# Mild, Selective Ru‐Catalyzed Deuteration Using D_2_O as a Deuterium Source

**DOI:** 10.1002/chem.201904927

**Published:** 2019-11-28

**Authors:** Pascal Eisele, Franziska Ullwer, Sven Scholz, Bernd Plietker

**Affiliations:** ^1^ Institut für Organische Chemie Universität Stuttgart Pfaffenwaldring 55 70569 Stuttgart Germany

**Keywords:** catalysis, C−H activation, deuterium, isotopes, ruthenium

## Abstract

A method for the selective deuteration of polyfunctional organic molecules using catalytic amounts of [RuCl_2_(PPh_3_)_3_] and D_2_O as a deuterium source is presented. Through variation of additives like CuI, KOH, and various amounts of zinc powder, orthogonal chemoselectivities in the deuteration process are observed. Mechanistic investigation indicates the presence of different, defined Ru‐complexes under the given specific conditions.

## Graphical abstract

**C−H activation**: A method for the selective deuteration of polyfunctional organic molecules using catalytic amounts of [RuCl_2_(PPh_3_)_3_] and D_2_O as a deuterium source is presented. Through variation of additives like CuI, KOH, and various amounts of zinc powder, orthogonal chemoselectivities in the deuteration process are observed (see scheme).

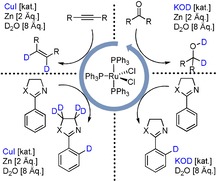

The exchange of hydrogen atoms for their isotopes deuterium or tritium is a common method for studies on biosynthesis but also metabolism.^1^ Moreover, the position selective deuteration is a mean to mask notoriously reactive C−H bonds against a fast oxidative metabolic degradation.^2^ The use of the kinetic isotope effect leads to a reduction of reactivity and hence to an improved bioavailabiltiy/‐stability of a pharmacophore in living organisms.^3^ On the other hand, the isotope effect can provide important insights into mechanistic issues.^1^, ^4^ There is thus much interest in the development of chemoselective deuteration methods.^5^ Often, the incorporation of deuterium or tritium occurs when using D_2_ or T_2_ gas, which is not unproblematic because of the hazard potential of these gases.^6^ The use of D_2_O (or T_2_O) for deuteration seems more feasible against this background.^7^

In the course of total syntheses and studies on possible metabolic degradation products, we needed a viable method for selective deuteration. Some time ago, we reported that the readily available Ru complex [RuCl_2_(PPh_3_)_3_] (**1**) catalyzes orthogonal‐chemoselective reductions of multifunctional substrates by using zinc and stoichiometric amounts of water in the presence of different cocatalysts.^8^ While the addition of CuI as a cocatalyst allows the selective reduction of alkynes to alkenes in the presence of carbonyl groups, the exchange of the cocatalyst reverses the sequence of reactivity. Alkynes are not reduced, and carbonyl groups are selectively converted into the corresponding alcohols.^8^ Based on these results and motivated by our search for a workable deuteration method, we initiated a research project in which we sought to exploit the cocatalyst‐dependent chemoselectivity trends of the Ru–Zn–H_2_O system for the development of selective isotope labeling. Herein we report the first results of this study, in which we demonstrate the selective deuteration of different polyfunctional organic substrates. NMR spectroscopic investigations were used to identify the in situ formed Ru catalysts **2**–**4** (Figure 1).

**Figure 1:**
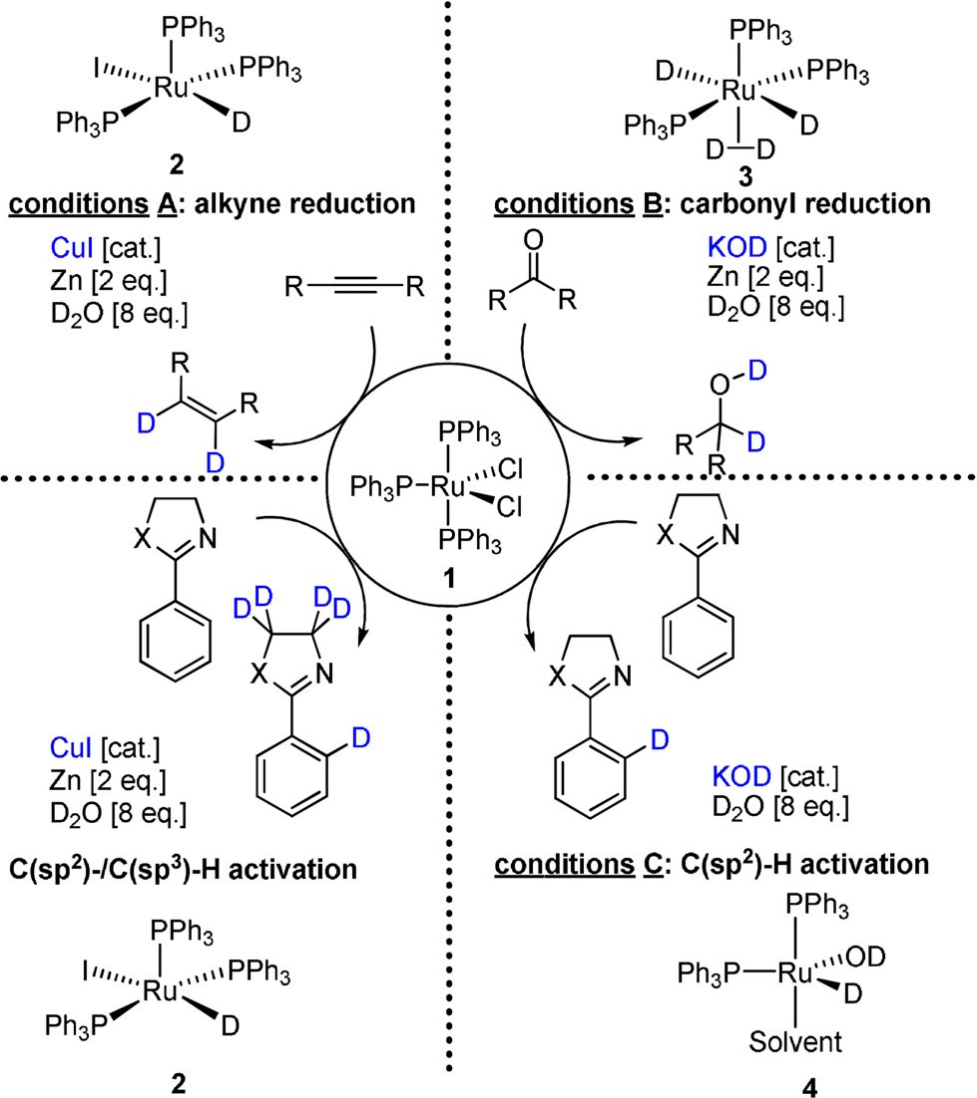
Additive‐dependent chemoselective Ru‐catalyzed deuterations.

The study presented here was based on in‐depth NMR spectroscopic studies on the influence of the additives KOH and CuI on the preacatalyst activation. The quintessence of this extensive work is summarized in Scheme 1. Consequently, the Ru−H species [RuHI(PPh_3_)_3_] (**2**) is formed in the presence of an excess of zinc in H_2_O using CuI as an additive starting from precatalyst [RuCl_2_(PPh_3_)_3_] (**1**) [Eq. (1), Scheme 1]. In contrast, the presence of KOH and zinc as an additive yields the Ru “superhydride” complex [Ru(H_2_)H_2_(PPh_3_)_3_] (**3**) [Eq. (3), Scheme 1]. In the absence of zinc, the Ru–OH complex **4** is formed from **1** in the presence of catalytic amounts of KOH [Eq. (2), Scheme 1]. These spectroscopic results provide a direct explanation for the observed chemoselectivities. For example, the activity of the structurally similar complex [RuHCl(PPh_3_)_3_] in the chemoselective reduction of olefins in the presence of carbonyl groups has been described in the literature.^9^ On the contrary, complex **3** is one of the most active Ru catalysts in the reduction of carbonyl groups and quasi inert to olefin/alkyne reductions.^10^

**Scheme 1:**
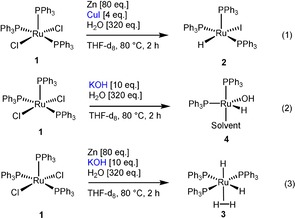
Activation of the precatalyst [RuCl_2_(PPh_3_)_3_] (**1**) through additives.

With these results in hand, the activation of D_2_O under the established conditions was studied by the reduction of diarylalkynes **5**–**7** and acetophenone derivatives **11**–**13** (Scheme 2). By addition of CuI, the *Z*‐bis‐deuterated olefins **8**–**10** could be isolated in good yields of 86 % at a deuteration degree of 85 %. Another 14 % corresponded to the *E*‐bis‐deuterated olefin; interestingly, the degree of deuteration on both olefinic carbon atoms was identical at about 85 %. Mixed H–D‐substituted olefins were undetectable. After a longer reaction time only *E*‐configured olefins **8**–**10** could be detected; a change in the degree of deuteration or overreduction was not observed [reaction conditions (A), Eq. (1), Scheme 2]. Upon addition of KOD [reaction conditions (B), Eq. (2), Scheme 2], no conversion was observed, whereas ketones **11**–**13**, upon addition of catalytic amounts of KOD, reacted cleanly to give the corresponding alcohols **14**–**16** [Eq. (3), Scheme 2]. The H–D exchange at the acidic α‐carbon atom occurs as a KOD‐catalyzed background reaction, and the addition of [RuCl_2_(PPh_3_)_3_] **1** was not necessary under these conditions. In the presence of CuI as an additive, however, no reduction of the carbonyl group and no deuteration at the α‐carbon atom was observed. Interestingly, under these conditions a selective deuteration of the two *ortho*‐carbon atoms in the aromatic moiety ketones **17**–**19** was observed [Eq. (4), Scheme 2].

**Scheme 2:**
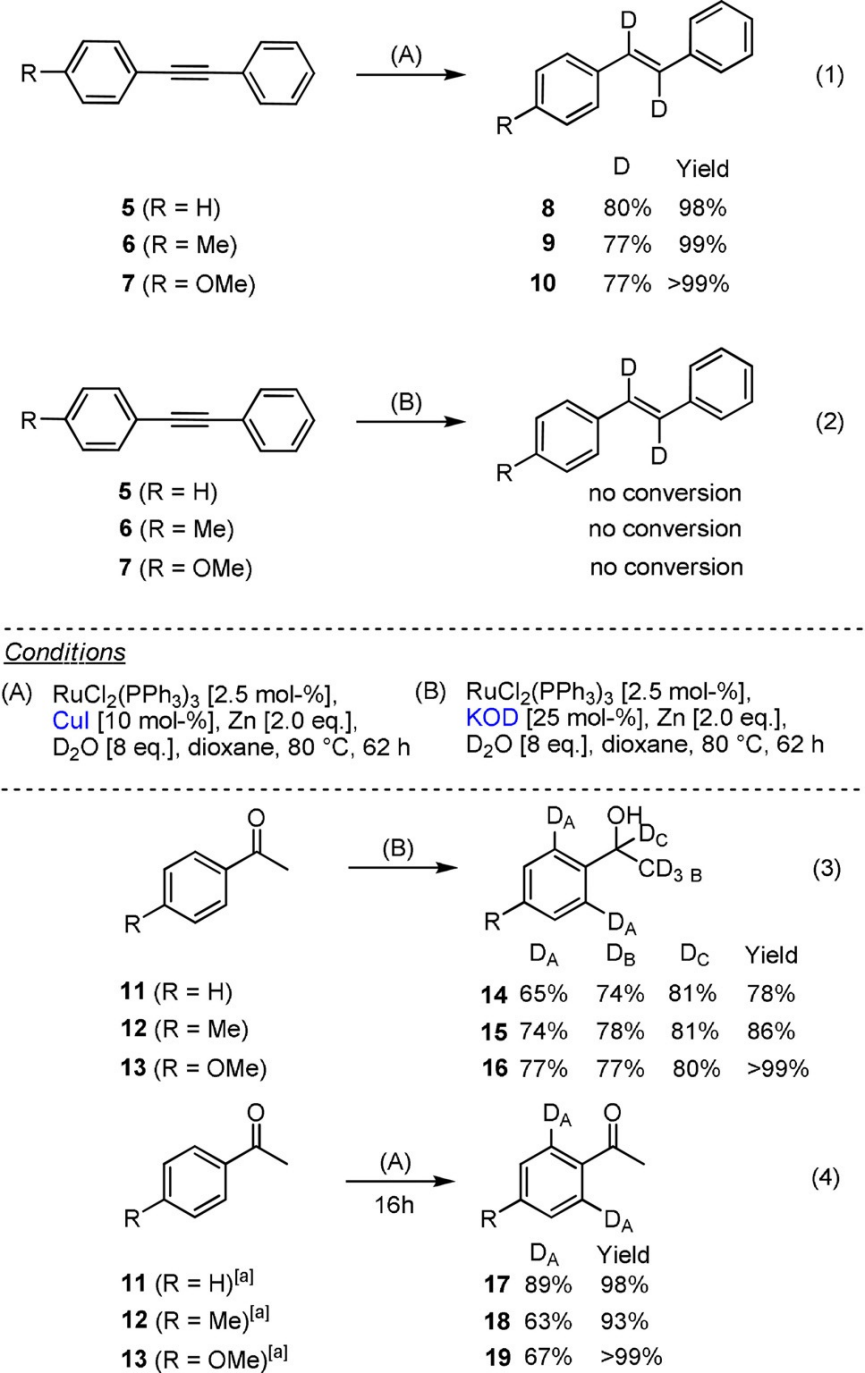
Additive‐directed chemoselective reductive deuteration of carbonyl compounds and alkynes. [a] 16 h reaction time.

Carbonyl groups are able to direct the *ortho*‐selective C−H activation through coordination of Ru catalysts. Since the groundbreaking work of Murai,^11^ a large number of Ru complexes have been developed that enable *ortho*‐C−H activation by a wide variety of directing groups under redox‐neutral^7^, ^12^ and oxidative^13^ conditions. The simple H–D exchange is formally a redox‐neutral transformation, which raises the question of the necessity of zinc as a reducing agent in such processes. To obtain a better overview of the scope of *ortho*‐deuteration, different catalyst‐directing groups were subsequently investigated with regard to their reactivity (Table 1). In fact, using catalytic amounts of [RuCl_2_(PPh_3_)_3_] (**1**) and KOD in the absence of stoichiometric amounts of zinc (conditions (C)) an efficient *ortho*‐selective C(sp^2^)–H–D exchange was possible. The use of catalytic amounts of [RuCl_2_(PPh_3_)_3_] (**1**) and CuI (conditions (A)), on the other hand, provided the expected deuterated aromatics only when using zinc.

**Table 1:** Ru‐catalyzed *ortho*‐deuteration of aromatics by using D_2_O as d‐surrogate after 16 h.^[a,b]^

Entry	Product				Entry	Product			
	Ru‐cat./KOD/D_2_O		Ru‐cat./CuI/Zn/D_2_O			Ru‐cat./KOD/D_2_O		Ru‐cat./CuI/Zn/D_2_O	
1	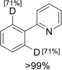	**20**	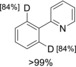	**20**	7	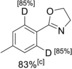	**27**	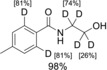	**28**
2	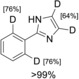	**21**	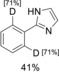	**21**	8	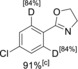	**29**	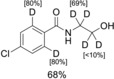	**30**
3	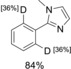	**22**	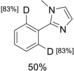	**22**	9	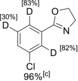	**31**	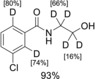	**32**
4	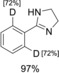	**23**	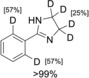	**23**	10^[c]^	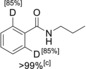	**33**	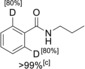	**33**
5	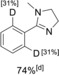	**24**	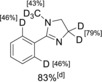	**24**	11	no reaction		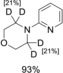	**34**
6	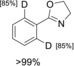	**25**	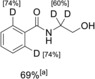	**26**	12^[e]^	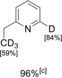	**35**	no reaction

[a] Conditions (A): substrate [0.5 mmol], [RuCl_2_(PPh_3_)_3_] [0.0125 mmol, 2.5 mol %], CuI [0.05 mmol, 10 mol‐%], zinc [1 mmol], D_2_O [4 mmol], dioxane [1 mL], 80 °C, 16 h. [b] Conditions (C): substrate [0.5 mmol], [RuCl_2_(PPh_3_)_3_] [0.0125 mmol, 2.5 mol %], KOD [0.125 mmol, 25 mol %], D_2_O [4 mmol], dioxane [1 mL], 80 °C, 16 h. [c] 62 h reaction time. [d] in [D_8_]THF. [e] in the presence of zinc powder.

Depending on the additive, significant differences in the deuteration were observed. While the *ortho*‐deuteration of C(sp^2^)−H bonds using catalytic amounts of KOD was highly selective, catalytic depletion of CuI led both to C(sp^2^)−H‐ and C (sp^3^)−H bond deuteration plus in some cases ring opening of the directing group, for example, oxazolidines. Since C(sp^3^)−H‐deuteration of benzoic acid propylamide **33** with the addition of both KOD and CuI led to almost identical results (entry 10, Table 1), we assume that C(sp^3^)−H‐deuteration occurs prior to the opening of the oxazolidinone. Importantly, no dehalogenation reactions were observed under either reaction conditions.^14^ Control experiments indicated that upon using CuI or KOD in the presence of stoichiometric amounts of zinc in situ generation of D_2_ from D_2_O is likely.^15^ However, since the *ortho*‐selective H–D exchange occurs with addition of catalytic amounts of KOD even without zinc, a direct deuteration from D_2_O can be assumed under these conditions.

With these results in hand, we turned to the deuteration of more complex substrates (Table 2). Both *p*‐ and *m*‐alkynylpyridyl‐substituted aromatics **36** and **37** were tested under the established conditions (entries 1 and 2, Table 2). The use of catalytic amounts of KOD both in the presence and absence of zinc occurred with no reduction of the CC triple bond but selective CH deuteration in the *ortho*‐position to the pyridyl substituent. In the presence of catalytic amounts of CuI, a semireduction of the alkynes to the olefins **41** and **42** plus *ortho*‐deuteration was observed. The initially formed *cis*‐configured olefins rearrange with longer reaction times into the *trans*‐products. Starting from arylalkynyl aryl ketones **38** and **39**, only deuteration of the carbonyl‐bound methyl group was observed in the presence of catalytic amounts of KOD. Interestingly, no *ortho*‐deuterations were detected (entries 3 and 4, Table 2). In analogy to substrates **36** and **37**, the use of catalytic amounts of CuI and stoichiometric amounts of zinc led to a selective semireduction of alkynes **38** and **39** plus *ortho*‐deuteration to give the corresponding olefins **43** and **44**. The substitution pattern of the starting materials **36**–**39** had a significant impact on the *ortho*‐deuteration process. While in *para*‐substituted substrates **36** and **38** both *ortho*‐positions were equally deuterated (entries 1 and 3, Table 2), only the position *ortho* to the catalyst‐directing group was deuterated for *meta*‐substituted arylalkynes **37** and **39**. Both electronic as well as steric effects might account for this finding (entries 2 and 4, Table 2).

**Table 2:** Additive‐directed Ru‐catalyzed deuteration of polyfunctional substrates after 62 h reaction time.^[a,b]^

Entry	Substrate		Product			
			Ru‐cat./KOD/D_2_O		Ru‐cat./CuI/Zn/D_2_O	
1	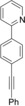	**36**	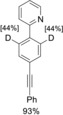	**36**	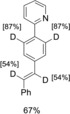	**41**
2	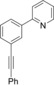	**37**	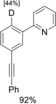	**37**	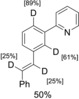	**42**
3	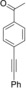	**38**	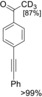	**38**	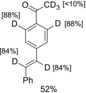	**43**
4	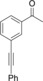	**39**	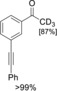	**39**	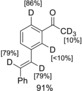	**44**
5^[c]^	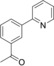	**40**	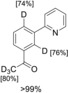	**40**	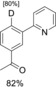	**40**

[a] Condition (A): substrate [0.5 mmol], [RuCl_2_(PPh_3_)_3_] [0.0125 mmol, 2.5 mol %], CuI [0.05 mmol, 10 mol %], zinc [1 mmol], D_2_O [4 mmol], dioxane [1 mL], 80 °C, 62 h. [b] Condition (C): substrate [0.5 mmol], RuCl_2_(PPh_3_)_3_ [0.0125 mmol, 2.5 mol %], KOD [0.125 mmol, 25 mol %], D_2_O [4 mmol], dioxane [1 mL], 80 °C, 62 h. [c] 16 h reaction time.

Finally, we tested both methods on complex drugs such as piribedil **45** or boscalid **46**. Fortunately, it was shown that both reaction conditions are also applicable to the selective deuteration of polyfunctional materials such as **45** and **46** [Eqs. (1)–(4), Scheme 3].

**Scheme 3:**
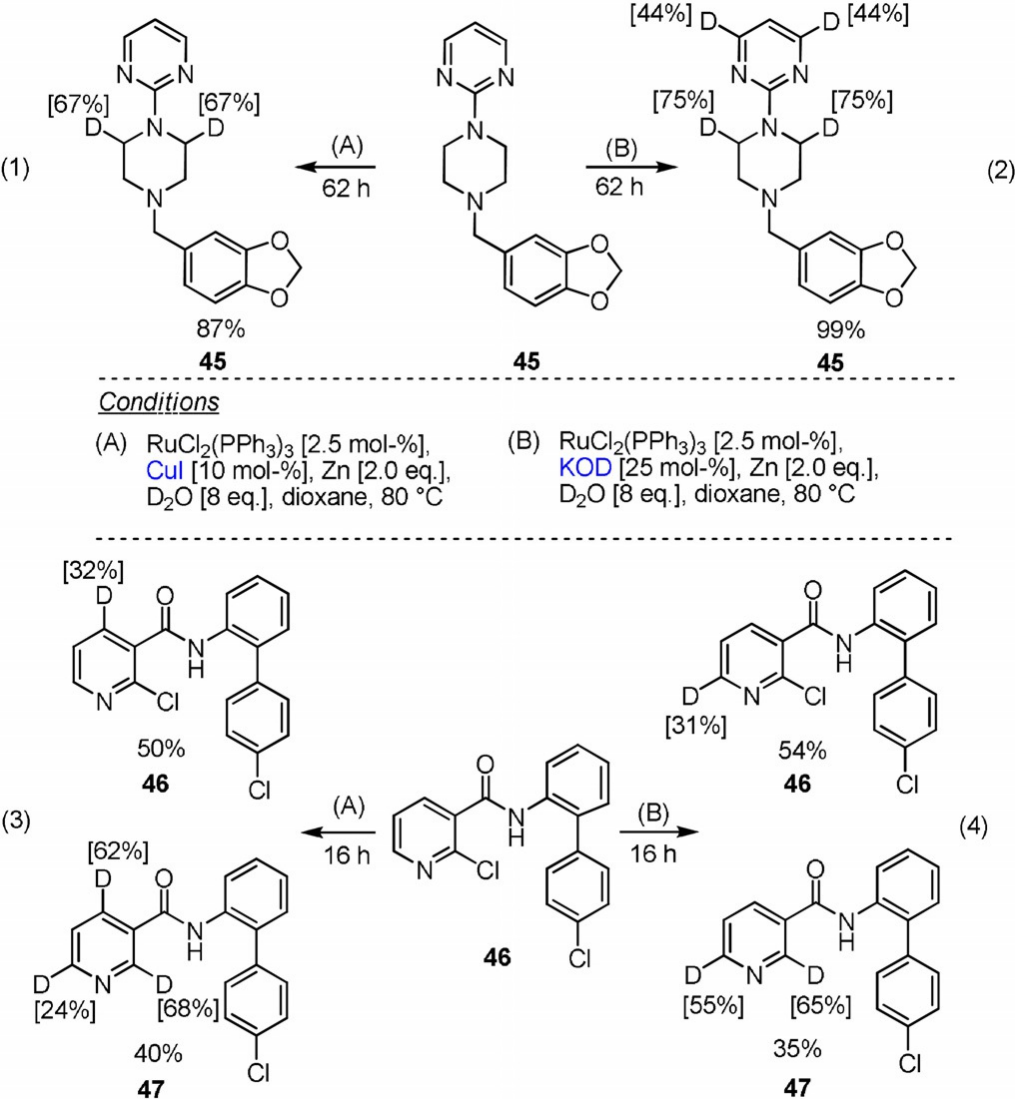
Additive‐directed Ru‐catalyzed deuteration of piribedil **45** and boscalid **46**.

Thus, in the presence of catalytic amounts of CuI (conditions (A), Scheme 3), a selective deuteration of the methylene group of the piperazine ring is observed [Eq. (1), Scheme 3]. The pyrimidine substituent obviously acts as a directing group. While under these conditions the pyrimidine ring is inert, we observed partial deuteration of the pyrimidine ring upon addition of catalytic amounts of KOD in addition to the H–D exchange at the methylene group of the piperazine [Eq. (2), Scheme 3]. It was found that by increasing the reaction time or by repeatedly reacting the deuterated product, higher degrees of deuteration can be obtained.^15^

The selectivity difference of our protocols is particularly evident in the reaction of boscalid **46** [Eqs. (3) and (4), Scheme 3]. Apparently, the carboxylic acid amide group at position 3 of the pyridine ring acts as a catalyst‐directing group, and in the presence of CuI directs the deuteration to the *ortho*‐positions of the pyridine ring [Eq. (3), Scheme 3]. In contrast, under KOD/ Zn conditions, the 6‐position of the pyridine ring is selectively deuterated [Eq. (4), Scheme 3]. In both cases, a partial Cl–D exchange is observed on the activated heteroaromatic moiety. In contrast, the second C−Cl bond in the unactivated aromatic remains unreactive under either conditions.

Herein, we present a preparatively simple method for the deuteration of functional organic molecules under mild conditions. Depending on the additive, the precatalyst [RuCl_2_(PPh_3_)_3_] **1** is converted into different defined Ru complexes **2**–**4** by using either CuI (cat.)/Zn, or KOD (cat.) or KOD (cat.)/Zn. Each of these complexes shows a different selectivity in H–D exchange reactions. D_2_O is used as the common deuterium source in all cases. When using zinc as an additive, the formation of D_2_ gas could be experimentally proven. Future work will aim to improve the present protocols, for example, through systematic variation of ligands, in order to amplify the selectivity trends.

## Conflict of interest

The authors declare no conflict of interest.

## Supporting information

As a service to our authors and readers, this journal provides supporting information supplied by the authors. Such materials are peer reviewed and may be re‐organized for online delivery, but are not copy‐edited or typeset. Technical support issues arising from supporting information (other than missing files) should be addressed to the authors.

Supplementary
