# Supplementary material for: Mild, Selective Ru‐Catalyzed Deuteration Using D2O as a Deuterium Source
Source: Chemistry. 2019 Nov 28;25(72):16550–4. doi: 10.1002/chem.201904927 (PMC6972544; doi:10.1002/chem.201904927)
Supplement: Supplementary file 1 — Supplementary [file CHEM-25-16550-s001.pdf]

CHEMISTRY
A European Journal

Supporting Information

Mild, Selective Ru-Catalyzed Deuteration Using D_2O as a Deuterium Source

Pascal Eisele[+], Franziska Ullwer[+], Sven Scholz, and Bernd Plietker*[a]

chem_201904927_sm_miscellaneous_information.pdf

Table of contents

1. General remarks 3
2. Synthesis of starting materials 4
3. Synthesis of reference compounds 32
3. Ru-catalyzed C-H-deuteration 43
3.1 General procedure for the deuteration following the CuI procedure (condition A) (GP-I). 43
3.2 General procedure for the deuteration following the KOD/Zn procedure (condition B) (GP-II) 43
3.3 General procedure for the deuteration following the KOD procedure (condition C) (GP-III) 43
3.4 General remarks on overview tables 43
3.5. Spectral data and overviews 44
3.5.1 Tolan 5 44
3.5.2 4-(phenylethynyl)toluene 6 49
3.5.3 4-(phenylethynyl)anisole 7 55
3.5.4 Acetophenone 11 61
3.5.5 4-Methylacetophenone 12 70
3.5.6 4-Methoxyacetophenone 13 75
3.5.7 2-Phenylpyridine 20 81
3.5.8 2-Phenylimidazole 21 88
3.5.9 *N*-methyl-2-phenyl-1H-imidazole 22 92
3.5.10 2-phenyl-2-imidazoline 23 95
3.5.11 4,5-dihydro-1-methyl-2-phenyl-1H-imidazole 24 97
3.5.12 2-phenyl-2-oxazoline 25 101
3.5.13 2-(4-methylphenyl)-4,5-dihydro-1.3-oxazole 27 104
3.5.14 2-(4-chlorophenyl)-4,5-dihydro-1.3-oxazole 29 108
3.5.15 2-(3-chlorophenyl)-4,5-dihydro-1.3-oxazole 31 112
3.5.16 N-(*n*-propyl)benzamide 33 119
3.5.17 2-(4-Morpholinyl)pyridine 34 124
3.5.18 2-Ethylpyridin 35 126
3.5.19 2-(4-(2-phenylethynyl)phenyl)pyridine 36 130
3.5.20 2-(3-(phenylethynyl)phenyl)pyridine 37 142
3.5.21 4-(phenylethinyl)acetophenone 38 156
3.5.22 1-(3-(2-phenylethynyl)phenyl)ethanone 39 164
3.5.23 1-(3-(pyridin-2-yl)phenyl)ethan-1-one 40 173
3.5.24 Piribedil 45 178

3.5.25 Boscalid 46 184
4. Synthesis of literature-known Ru-complexes 192
4.1 $Ru_2(H)_2(\mu\text{-}OH)_2(solvent)_2(PPh_3)_4$ 48 and $RuH(OH)(solvent)(PPh_3)_3$ 4[11] 193
4.2 $RuH_2(H_2)(PPh_3)_3$ 3[12] 194
4.3 $RuHI(PPh_3)_3$ 2 195
5. Mechanistic studies 197
5.1 KOH-protocol 197
5.2 KOH/Zn-protocol 201
5.2.1 Deuteration with $RuH_2(H_2)(PPh_3)_3$ 203
5.3 CuI-protocol 204
5.3.1 Deuteration with $RuHI(PPh_3)_3$ 2 206
5.3.2 Reduction with $RuHI(PPh_3)_3$ 2 206
5.4 Verification of D_2 gas formation 210
6. Literature 213

1. General remarks

All reactions and manipulations were performed under dry nitrogen by using standard Schlenk techniques. All solvents were purified prior to use. KOD was obtained as solution in D_2O (40 wt. % in D_2O, 98 atom % D) from Sigma-Aldrich (now Merck). $RuCl_2(PPh_3)_3$ (97%) was obtained from Sigma-Aldrich (now Merck). All other chemicals were purchased from Acros Organics, Sigma-Aldrich/Merck, Alfa Aesar, TCI, abcr or ChemPUR. NMR spectra were recorded on a Bruker Avance 300 spectrometer at 300 MHz (1H NMR), 75 MHz (13C NMR), a Bruker Ascend 400 spectrometer at 400 MHz (^{1}H NMR), 101 MHz (^{13}C NMR), a Bruker Avance 500 spectrometer at 500 MHz (^{1}H NMR), 126 MHz (^{13}C NMR), or a Bruker Avance 700 spectrometer at 700 MHz (^{1}H NMR), 176 MHz (^{13}C NMR). Chemical shifts are reported in ppm down field using tetramethylsilane or the signal of the deuterated solvent as an internal standard. Coupling constants *J* are given in Hz. The following abbreviations are used in the analysis of NMR spectra: s=singlet, d=doublet, t=triplet, q=quartet, hept=heptet, sept=septet. Combination of these abbreviations is applied whenever more than one coupling is observed. IR spectra were measured on a FT-IR spectrometer in an ATR mode. The intensity of the observed peaks is given in parenthesis: s=strong, m=medium,w=weak. Mass spectra were measured using electrospray ionization on a Bruker micrOTOF-Q. High performance liquid chromatography (HPLC) was performed using a Knauer K-501 pump, Knauer RI-detector K 2400 and a Macherey-Nagel VP250/21 Nucleodur 100-5 column.

E/*Z*-ratios and yields were determined by crude-NMR with internal standard. Degree of deuteration was determined by comparing the ^{1}H NMR integral of non-reactive protons with the deuterated proton signals. In order to identify the signals the pure compounds were measured and displayed unless the compounds were not stable which was the case for some of the (*Z*)-isomers of the obtained alkenes. In that case signals were compared with literature.

Signals of non-reactive protons are marked in blue whereas reactive positions are marked in red. Deuteration is given in percent next to the corresponding position. If the deuteration degree is lower than 10% the numbers were not added to the position in the molecule as we consider the measurement error in the NMR to be too significant for that value.

For volatile and very polar substrates the solvent 1,4-dioxane was replaced by THF-d_8 in order to measure NMRs without prior work-up.

2. Synthesis of starting materials

4-(phenylethinyl)acetophenone S1

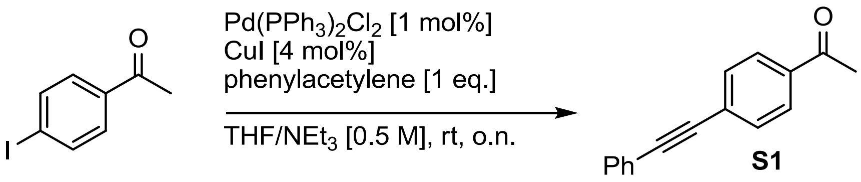

4-(phenylethinyl)acetophenone S1[1]

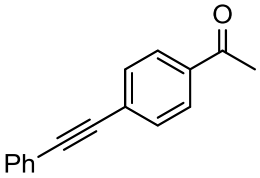

4-Iodoacetophenone (1.72 g, 7.0 mmol, 1.00 eq.), $Pd(PPh_3)_2Cl_2$ (49.1 mg, 0.07 mmol, 1 mol%), CuI (53.3 mg, 0.28 mmol, 4 mol%) and phenylacetylene (0.77 ml, 7 mmol, 1 eq.) were dissolved in THF (7 ml) and triethylamine (7 ml). After stirring over night at room temperature H_2O (17.5 ml) was added and the reaction mixture was extracted with diethylether (4 x 17.5 ml). The combined organic layers were dried over anhydrous Na_2SO_4 and concentrated under reduced pressure. The residue was purified by column chromatography on silica gel (petroleum ether/EtOAc – 10/1).

Yield: 1.24 g (5.6 mmol, 80%).

Physical State: colourless solid.

R_f Value: 0.26 (petroleum ether/EtOAc – 10/1).

^{1}H NMR (Avance 300 MHz, $CDCl_3$) δ 7.99 – 7.90 (m, 2H), 7.65 – 7.58 (m, 2H), 7.58 – 7.50 (m, 2H), 7.43 – 7.34 (m, 3H), 2.62 (s, 3H) ppm.

^{13}C NMR (Avance 101 MHz, $CDCl_3$) δ 197.3, 136.2, 131.8, 131.7, 128.8, 128.5, 128.3, 128.2, 122.7, 92.7, 88.6, 26.6 ppm.

IR (ATR, in $CDCl_3$) ν 2218 (m), 1676 (s), 1601 (m), 1553 (m), 1485 (m), 1442 (m), 1433 (m), 1423 (m), 1404 (m), 1359 (m), 1262 (m), 1180 (m), 1142 (m), 1108 (m) cm^{-1}.

HRMS (EI, m/z) calcd. for $C_{16}H_{12}O$: 220.0888, found: 220.0890.

^{1}H- and ^{13}C-NMR spectra of S1

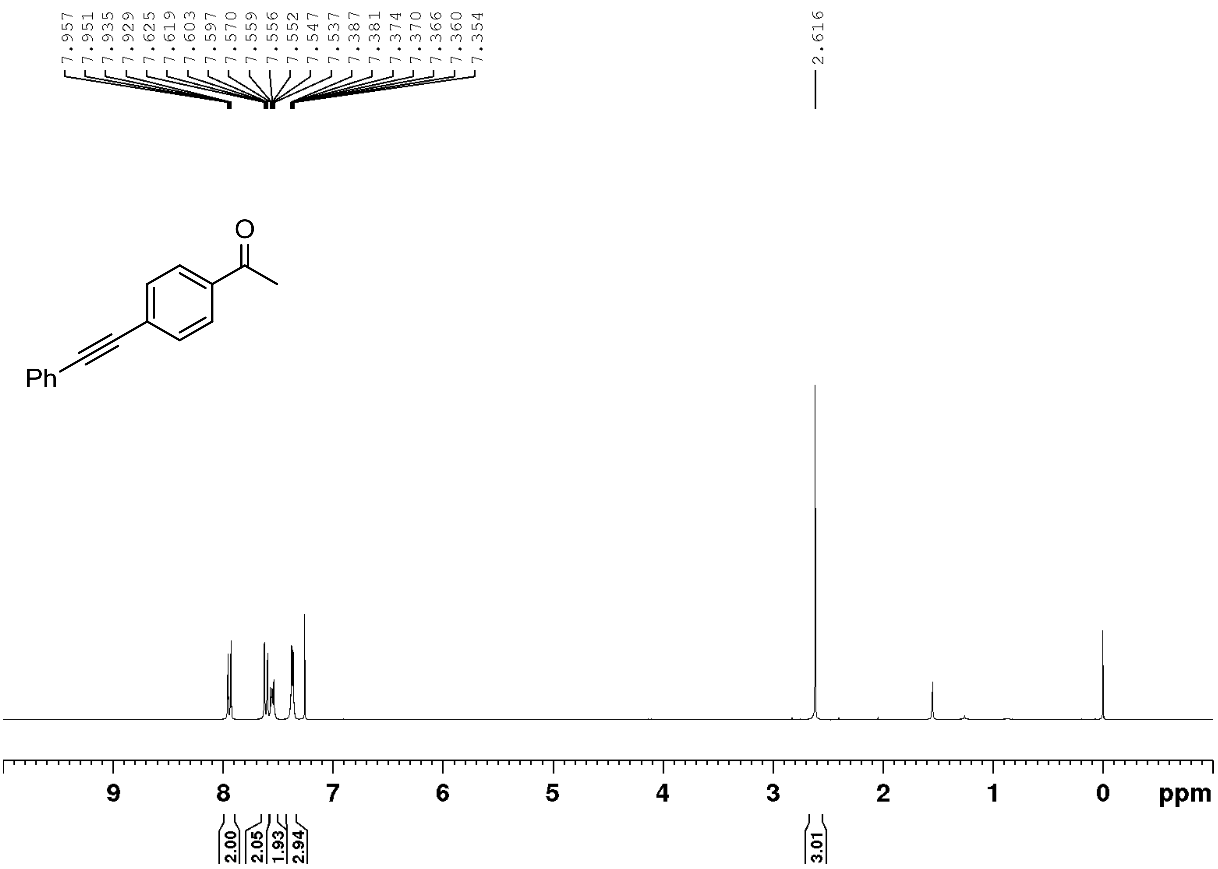

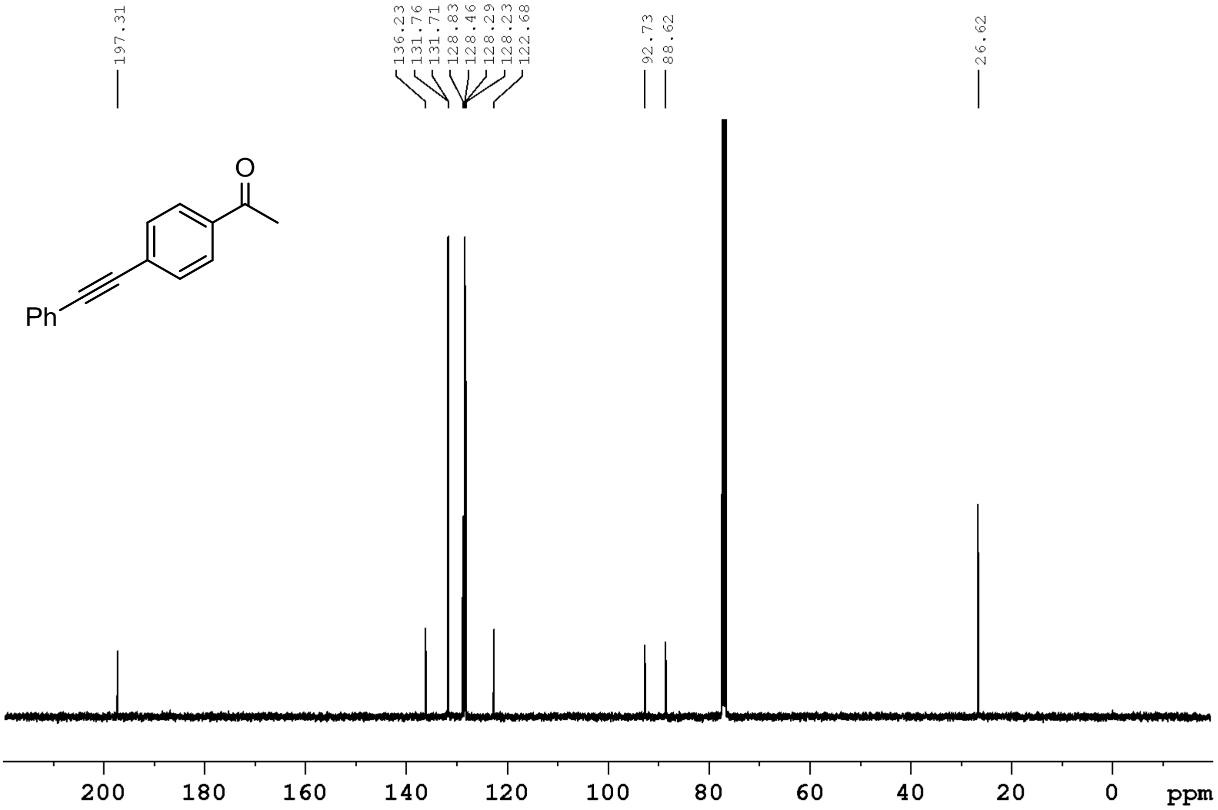

2-(4-(2-phenylethynyl)phenyl)pyridine S2

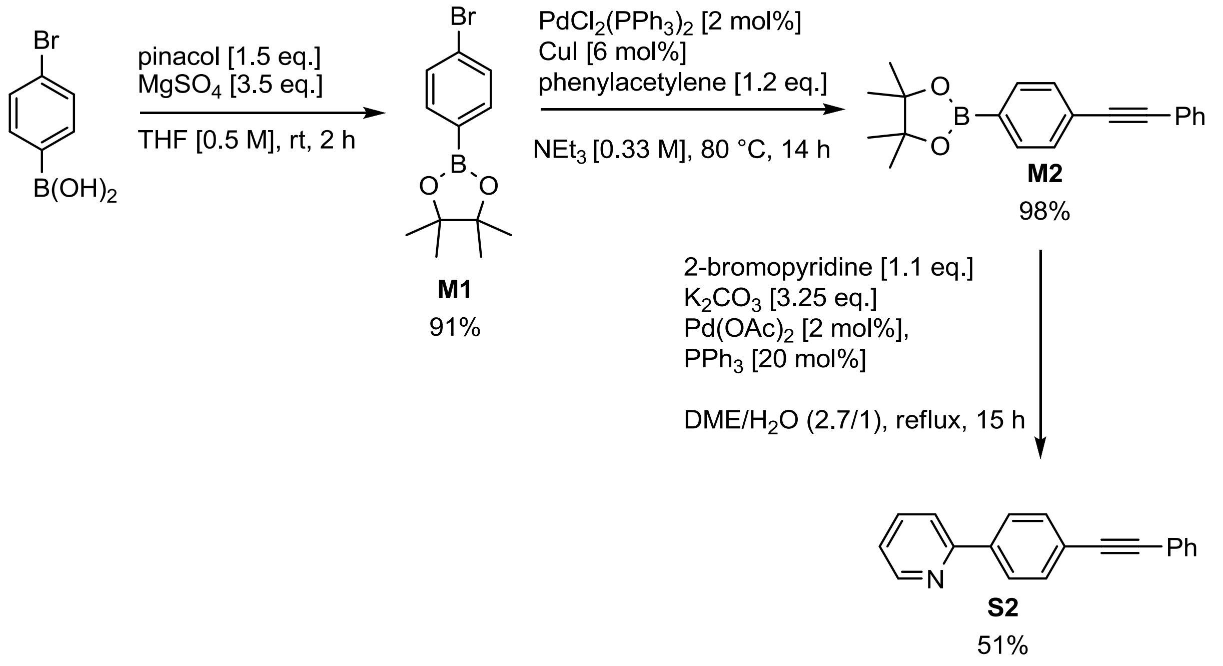

***p*-bromophenylboronic acid pinacol ester M1**

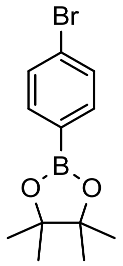

4-Bromophenyl boronic acid (2.01 g, 10 mmol, 1.0 eq.), pinacol (1.773 g, 15 mmol, 1.5 eq.) and $MgSO_4$ (4.213 g, 35 mmol, 3.5 eq.) were dissolved in THF (20 ml) and stirred for 2 h at room temperature. The crude mixture was filtered, purified over a pad of silica and concentrated under reduced pressure.

Yield: 2.58 g (9.1 mmol, 91%).

Physical State: colourless solid.

R_f Value: 0.58 (petroleum ether/EtOAc – 40/1).

^{1}H NMR (Avance 400 MHz, $CDCl_3$) δ 7.66 (d, J = 8.0 Hz, 2H), 7.50 (d, J = 8.1 Hz, 2H), 1.34 (s, 12H) ppm.

^{13}C NMR (Avance 101 MHz, $CDCl_3$) δ 136.3, 131.0, 126.2, 84.0, 24.9 ppm.

IR (ATR, in $CDCl_3$) ν 2978 (m), 2931 (w), 1588 (s), 1558 (w), 1467 (w), 1389 (m), 1355 (s), 1324 (m), 1142 (s), 1087 (s), 1012 (s) cm^{-1}.

HRMS (EI, m/z) calcd. for $C_{12}H_{16}BBrO_2$: 282.0429, found: 282.0427.

4,4,5,5-tetramethyl-2-[4-(2-phenylethynyl)phenyl]-1,3,2-dioxaborolane M2[2]

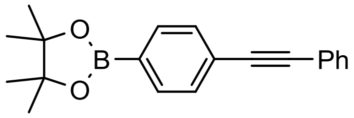

4-Bromophenylboronic acid pinacol ester **M1** (2.524 g, 8.92 mmol, 1.0 eq.), $PdCl_2(PPh_3)_2$ (125.2 mg, 0.18 mmol, 2 mol%) and CuI (101.9 mg, 0.54 mmol, 6 mol%) were dissolved in NEt_3 (26.8 ml). After stirring at room temperature for 5 min phenylacetylene (1.18 ml, 10.7 mmol, 1.2 eq.) was added and the mixture was stirred 22 h at 80 °C. The crude mixture was filtered through a pad of celite (eluent: EtOAc), quenched with NH_4Cl solution and extracted with EtOAc. The combined organic layers were dried over anhydrous Na_2SO_4 and concentrated under reduced pressure. The residue was purified by flash column chromatography on silica gel (petroleum ether → EtOAc).

Yield: 2.67 g (8.8 mmol, 98%).

Physical State: colourless solid.

R_f Value: 0.22 (petroleum ether/EtOAc – 40/1).

^{1}H NMR (Avance 400 MHz, $CDCl_3$) δ 7.78 (d, *J* = 8.2 Hz, 2H), 7.55 – 7.51 (m, 4H), 7.37 – 7.32 (m, 3H), 1.35 (s, 12H) ppm.

^{13}C NMR (Avance 101 MHz, $CDCl_3$) δ 134.6, 131.7, 130.8, 128.4, 126.0, 123.2, 90.7, 89.6, 84.0, 24.9 ppm.

IR (ATR, in $CDCl_3$) ν 2978 (w), 1607 (m), 1488 (w), 1443 (w), 1397 (m), 1359 (s), 1323 (m), 1261 (w), 1213 (w), 1141 (m); 1087 (m), 1019 (w) cm^{-1}.

HRMS (EI, m/z) calcd. for $C_{20}H_{21}BO_2$: 304.1638, found: 304.1638.

2-(4-(2-phenylethynyl)phenyl)pyridine S2[3]

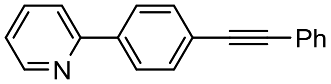

The substrate **M2** (0.913 g, 3 mmol, 1.0 eq.), 2-bromopyridine (0.53 g, 3.3 mmol, 1.1 eq.) and K_2CO_3 (1.35 g, 9.75 mmol, 3.25 eq.) were dissolved in dimethoxyethane (27 ml) and H_2O (10 ml). After degassing with N_2 for 30 min, $Pd(OAc)_2$ (33.7 mg. 0.15 mmol, 5 mol%) and PPh_3 (157.4 mg, 0,6 mmol, 20 mol%) were added and the mixture was refluxed for 15 h. DCM was added and the organic layer was dried over anhydrous Na_2SO_4 and purified by flash column chromatography on silica gel (eluent: DCM) and precipitated from hot EtOH (55 ml).

Yield: 0.39 g (1.5 mmol, 51%).

Physical State: cream-coloured solid.

R_f Value: 0.15 (petroleum ether/EtOAc – 40/1).

^{1}H NMR (Avance 400 MHz, CD_2Cl_2) δ 8.65 – 8.55 (m, 1H), 7.99 – 7.92 (m, 2H), 7.73 – 7.67 (m, 2H), 7.58 – 7.53 (m, 2H), 7.50 – 7.45 (m, 2H), 7.33 – 7.25 (m, 3H), 7.20 – 7.13 (m, 1H) ppm.

^{13}C NMR (Avance 101 MHz, CD_2Cl_2) δ 156.2, 149.8, 139.1, 136.8, 131.9, 131.6, 128.4, 126.7, 123.7, 123.1, 122.4, 120.3, 90.5, 89.1 ppm.

IR (ATR, in CD_2Cl_2) ν 3077 (w), 3051 (w), 3005 (w), 1605 (w), 1585 (s), 1571 (m), 1552 (w), 1513 (w), 1486 (m), 1462 (s), 1434 (s), 1403 (w), 1391 (w), 1311 (w), 1293 (w), 1262 (w), 1237 (w), 1187 (w), 1177 (w), 1154 (m), 1107 (w), 1096 (w), 1070 (w), 1059 (w), 1028 (w), 1014 (w) cm^{-1}.

HRMS (ESI, m/z) calcd. for $C_{19}H_{13}N$: 255.1048, found: 255.1053.

^{1}H- and ^{13}C-NMR spectra of S2

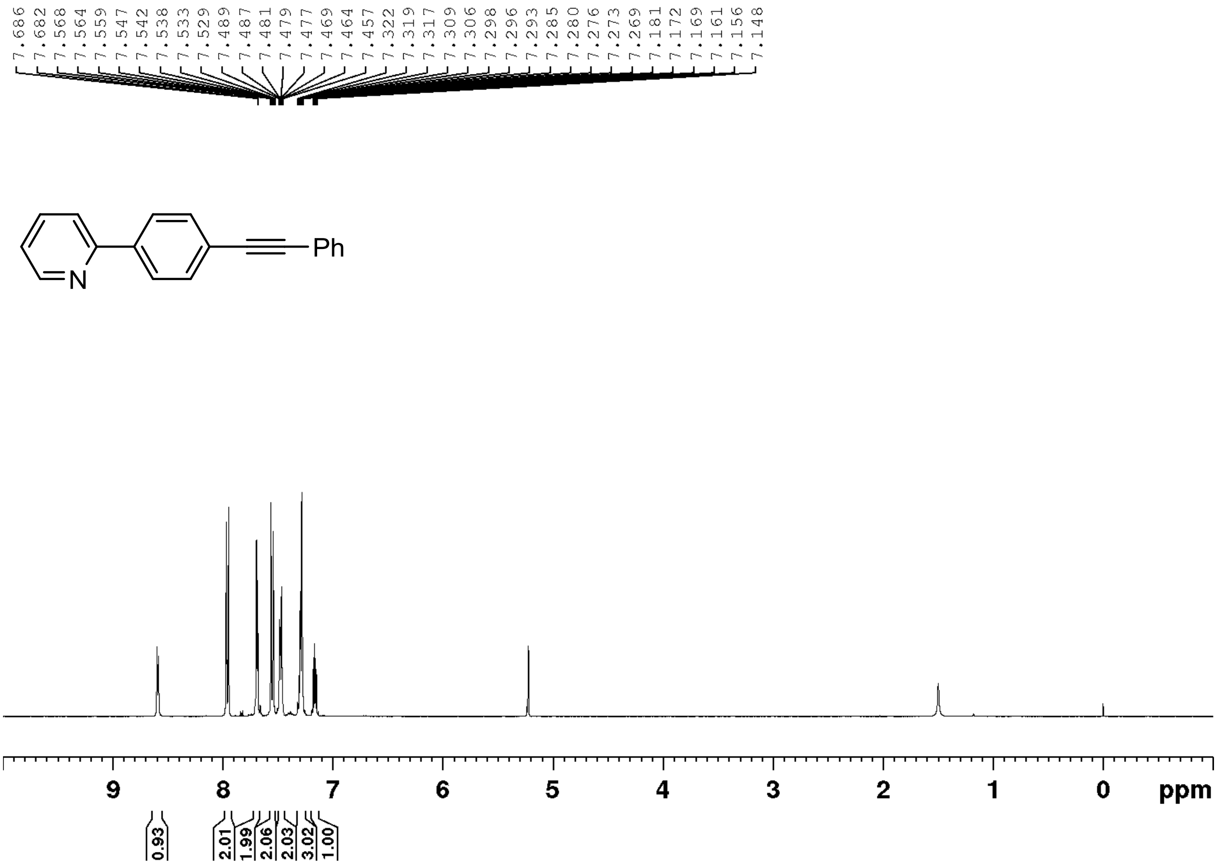

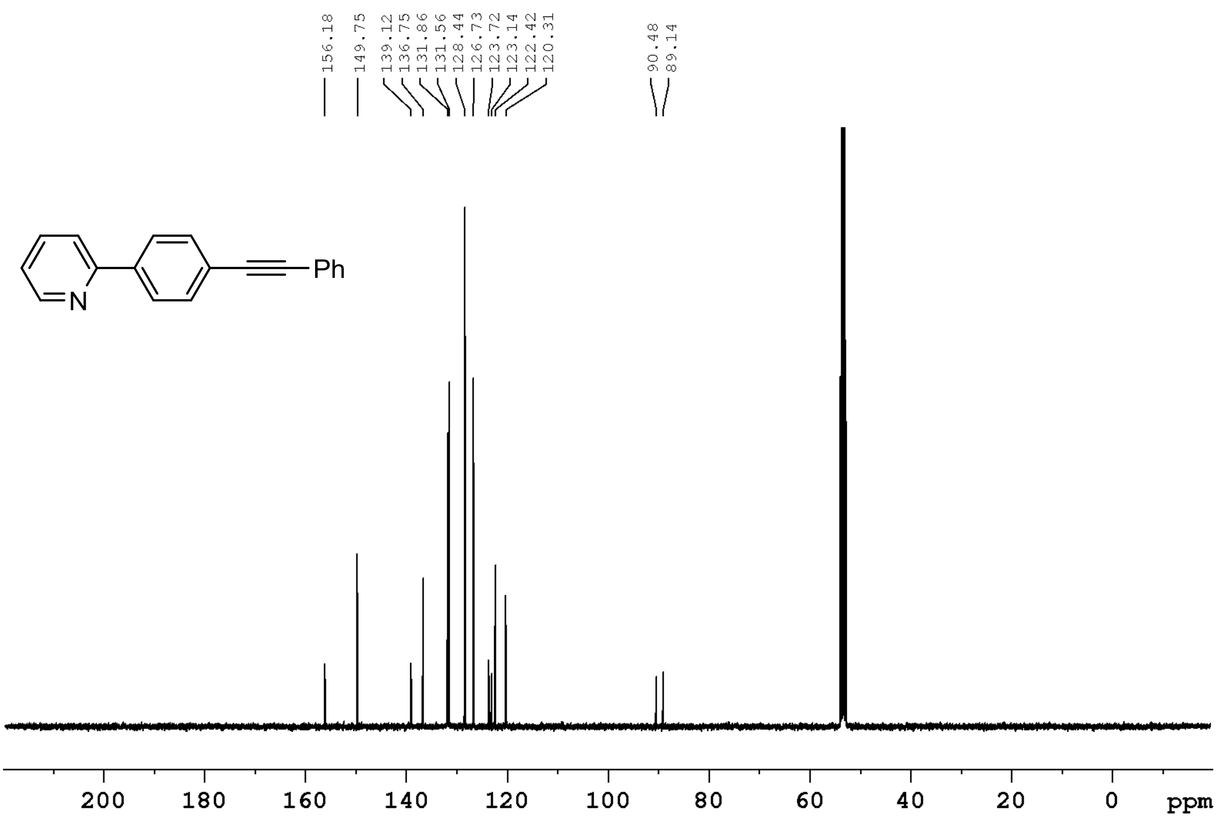

2-(3-(phenylethynyl)phenyl)pyridine S3

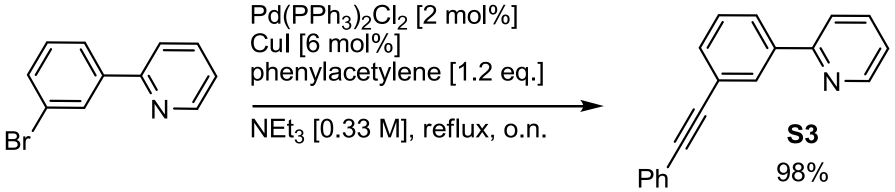

2-(3-(phenylethynyl)phenyl)pyridine S3

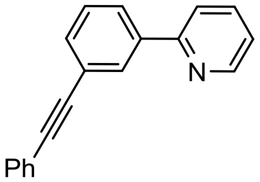

2-(3-Bromophenyl)pyridine (1.17 g, 5 mmol, 1 eq.), $Pd(PPh_3)_2Cl_2$ (70.2 mg, 0.1 mmol, 2 mol%) and CuI (57.1 mg, 0.3 mmol, 6 mol%) were dissolved in triethylamine (15 ml) and stirred for 5 min. Afterwards phenylacetylene (0.66 ml, 6 mmol, 1 eq) was added and the mixture was refluxed over night. A saturated NH_4Cl solution was added and the mixture was extracted with Et_2O, dried over anhydrous Na_2SO_4 and concentrated under reduced pressure. The crude product was purified by flash column chromatography on silica gel (petroleum ether/EtOAc – 40/1→10/1→5/1).

Yield: 1.25 g (4.9 mmol, 98%).

Physical State: red-brown oil.

R_f Value: 0.31 (petroleum ether/EtOAc – 10/1).

^{1}H NMR (Avance 400 MHz, $CDCl_3$) δ 8.71 (d, *J* = 4.76 Hz, 1H), 8.19 (t, *J* = 1.54 Hz, 1H), 7.98 (dt, *J* = 7.82, 2.86, 1H), 7.81- 7.72 (m, 2H), 7.62 – 7.50 (m, 3H), 7.46 (t, *J* = 7.74, 1H), 7.40 – 7.30 (m, 3H), 7.29 – 7.22 (m, 1H) ppm.

^{13}C NMR (Avance 101 MHz, $CDCl_3$) δ 156.6, 149.8, 139.6, 136.9, 132.0, 131.7, 130.2, 128.8, 128.4, 128.3, 126.8, 123.9, 123.3, 122.5, 120.6, 89.6, 89.3 ppm.

IR (ATR, in $CDCl_3$) ν 3056 (w), 1601 (w), 1585 (m), 1565 (w), 1493 (w), 1470 (w), 1461 (m), 1443 (w), 1433 (w), 1405 (w), 1324 (w), 1295 (w), 1271 (w), 1153 (w), 1069 (w), 1026 (w) cm^{-1}.

HRMS (ESI, m/z) calcd. for $C_{19}H_{13}N+H^+$: 256.1121, found: 256.1122.

^{1}H- and ^{13}C-NMR spectra of S3

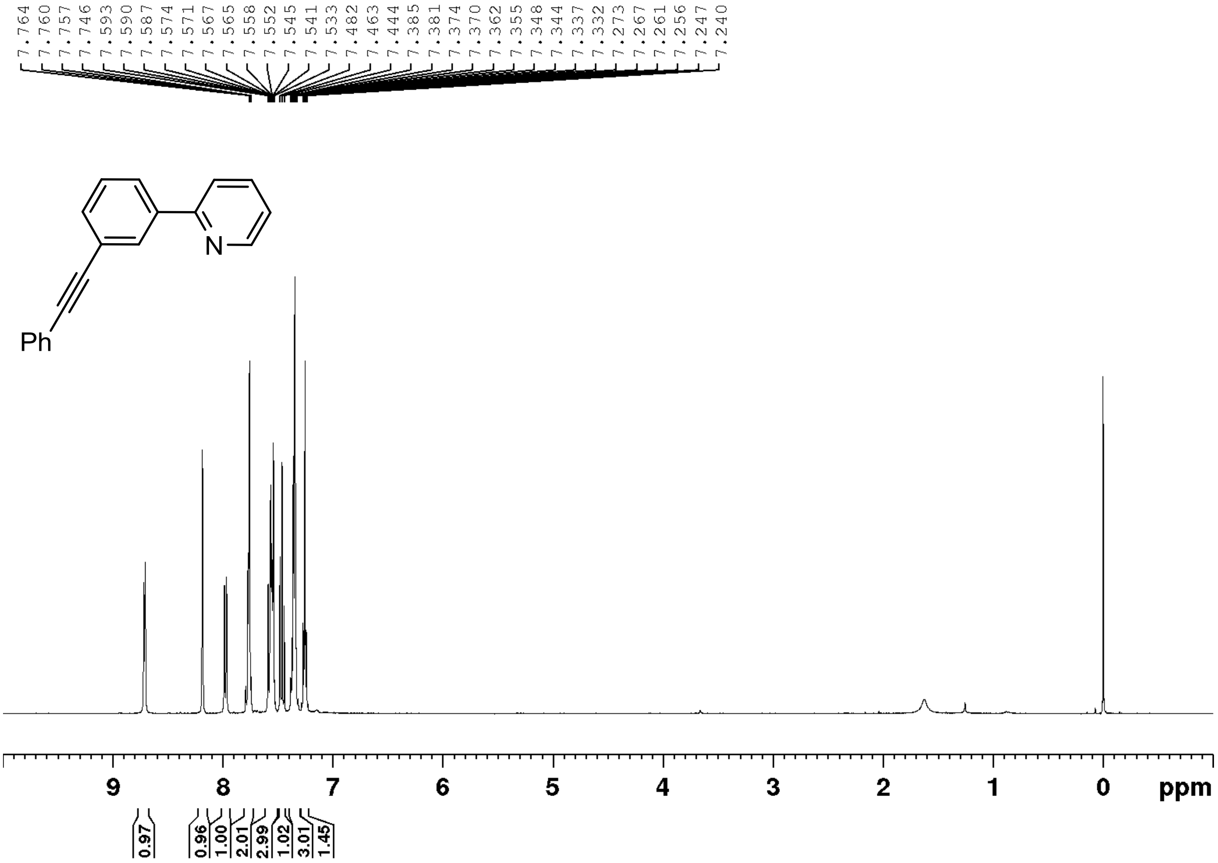

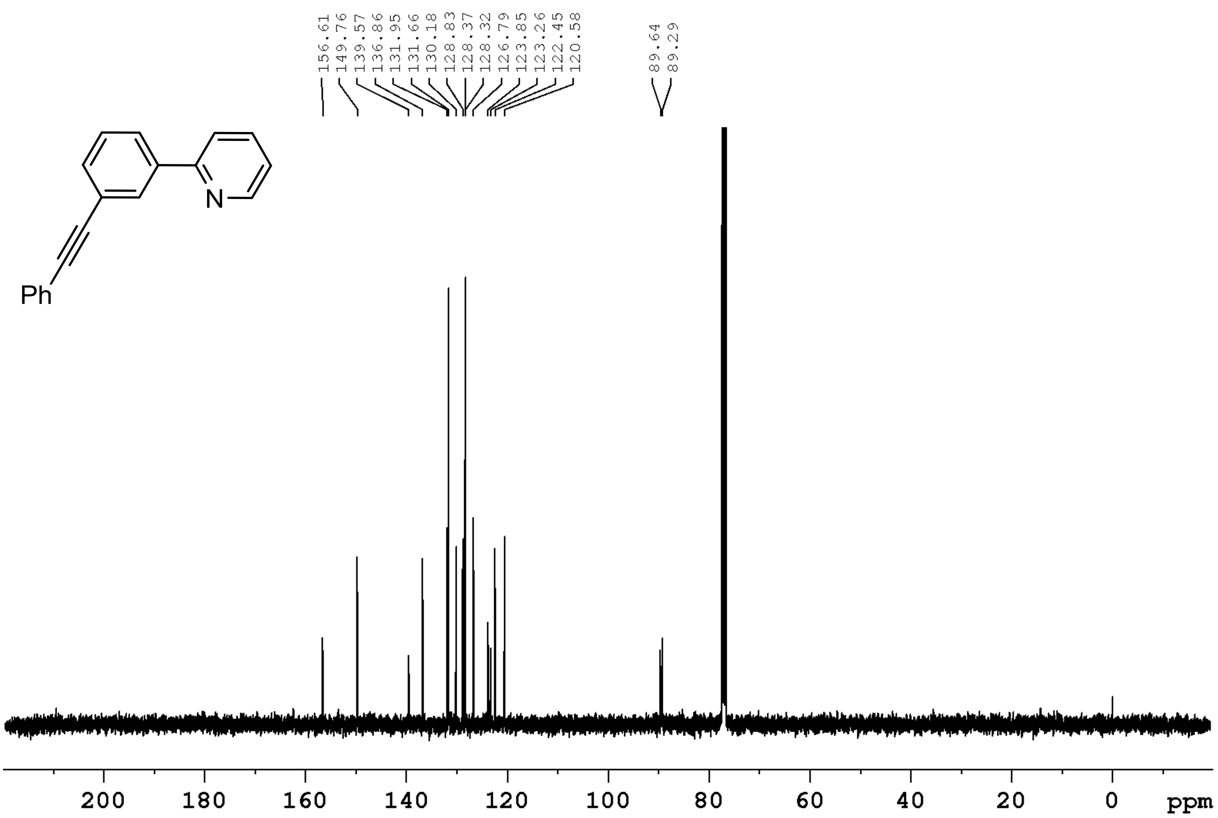

1-(3-(pyridin-2-yl)phenyl)ethan-1-one S4

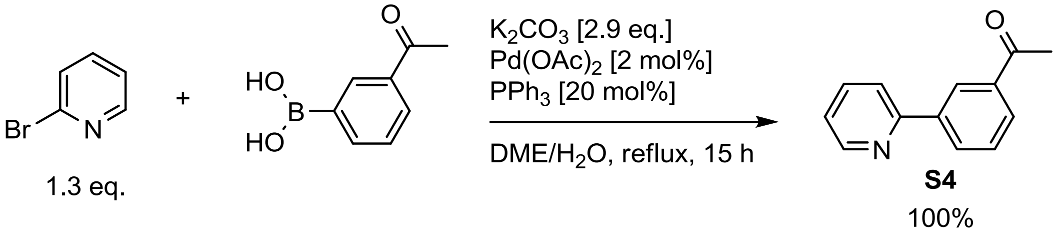

1-(3-(pyridin-2-yl)phenyl)ethan-1-one S4[3]

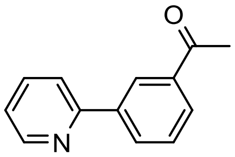

3-Acetylphenylboronic acid (0.98 g, 6 mmol, 1.35 eq.), 2-bromopyridine (0.44 ml, 4.5 mmol, 1. eq.) and K_2CO_3 (1.80 g, 13 mmol, 2.9 eq.) were dissolved in dimethoxyethane (36 ml) and H_2O (13 ml). After degassing with N_2 for 30 min, $Pd(OAc)_2$ (44.9 mg. 0.2 mmol, 5 mol%) and PPh_3 (209.8 mg, 0.8 mmol, 20 mol%) were added and the mixture was refluxed for 15 h. DCM was added and the organic layer was dried over anhydrous Na_2SO_4 and purified by column chromatography on silica gel (petroleum ether/EtOAc – 3/1→2/1).

Yield: 0.89 g (4.5 mmol, 100%).

Physical State: red-brown oil.

R_f Value: 0.24 (petroleum ether/EtOAc – 2/1).

^{1}H NMR (Avance 400 MHz, $CDCl_3$) δ 8.77 – 8.68 (m, 1H), 8.64 – 8.55 (m, 1H), 8.27 – 8.17 (m, 1H), 8.06 – 7.97 (m, 1H), 7.84 – 7.74 (m, 2H), 7.63 – 7.53 (m, 1H), 7.33 – 7.24 (m, 1H), 2.68 (s, 3H) ppm.

^{13}C NMR (Avance 101 MHz, $CDCl_3$) δ 198.0, 156.4, 149.8, 139.9, 137.7, 136.9, 131.5, 129.1, 128.7, 126.8, 122.6, 120.7, 26.8 ppm.

IR (ATR, in $CDCl_3$) ν 3064 (w), 3005 (w), 1680 (s), 1584 (m), 1566 (m), 1488 (w) , 1462 (m), 1433 (m), 1414 (m), 1356 (m), 1297 (m), 1270 (w), 1231 (s), 1175 (w), 1154 (w), 1083 (w), 1066 (w), 1041 (w), 1020 (w) cm^{-1}.

HRMS (ESI, m/z) calcd. for $C_{13}H_{11}NO+H^+$: 198.0913, found: 198.0913.

^{1}H- and ^{13}C-NMR spectra of S4

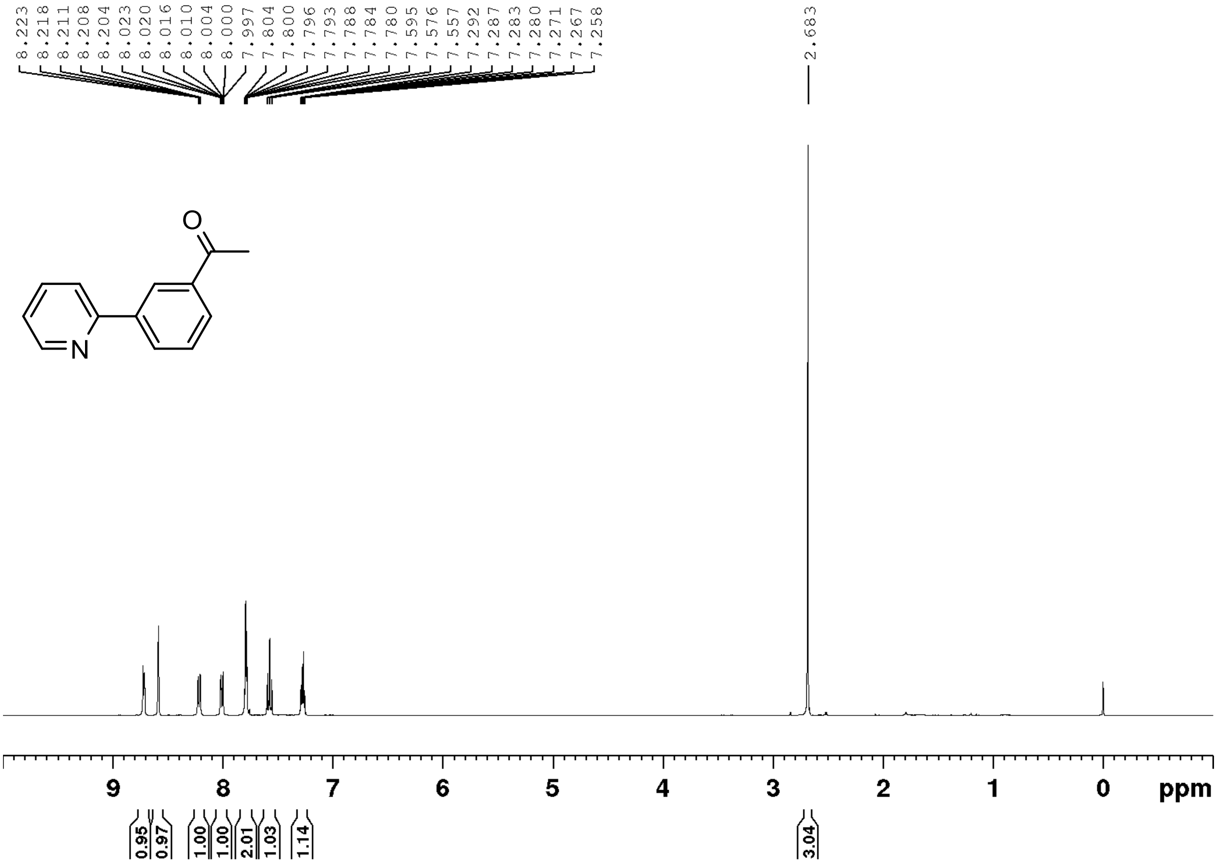

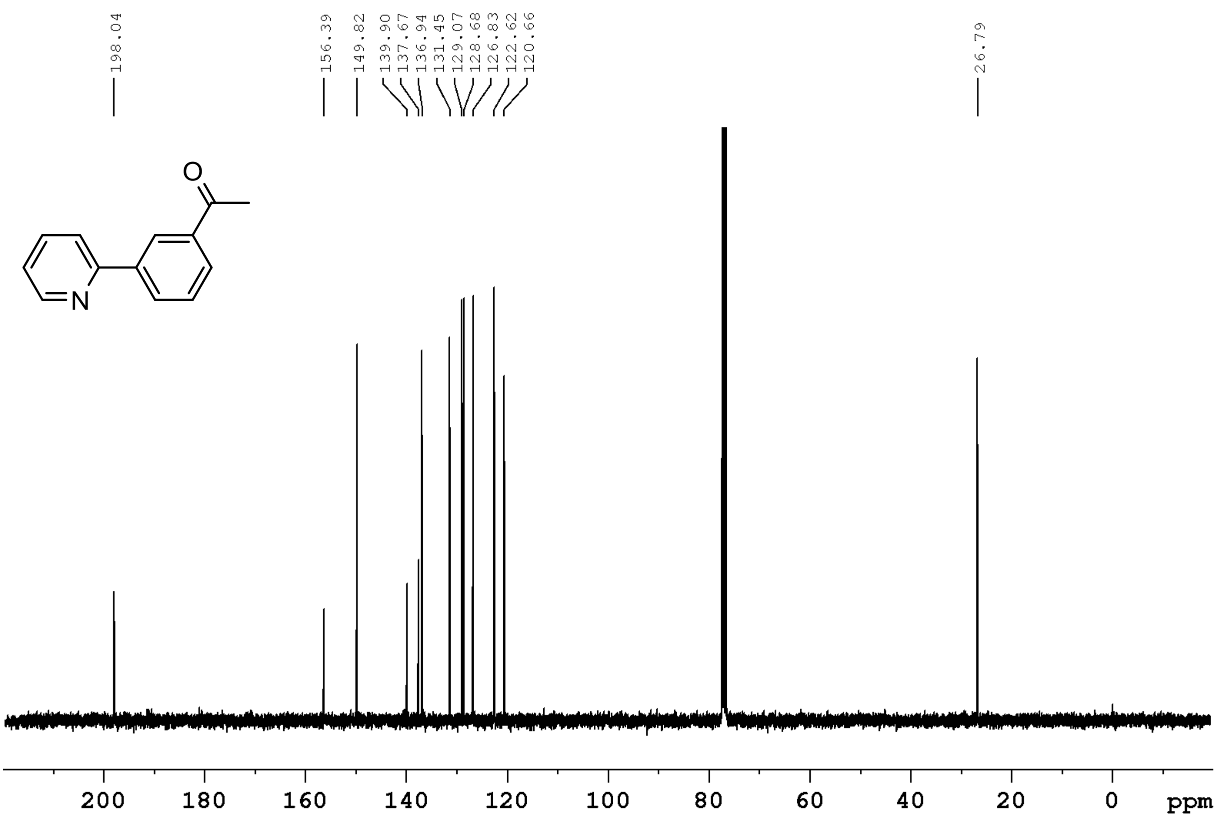

4-(phenylethynyl)toluene S5

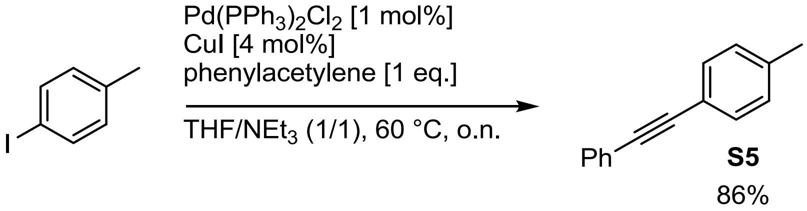

4-(phenylethynyl)toluene S5[1]

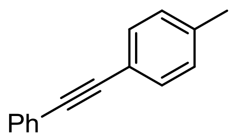

4-Tolyl iodide (2.18 g, 10 mmol, 1 eq.), $Pd(PPh_3)_2Cl_2$ (70.2 mg, 0.1 mmol, 1 mol%), CuI (76.2 mg, 0.4 mmol, 4 mol%) and phenylacetylene (1.1 ml, 10 mmol, 1 eq.) were dissolved in THF (10 ml) and triethylamine (10 ml). After stirring over night at 60 °C demin. H_2O (25 ml) was added and the reaction mixture was extracted with diethylether (4 x 25 ml). The combined organic layers were washed with brine, dried over anhydrous Na_2SO_4 and concentrated under reduced pressure. The residue was purified by column chromatography on silica gel (petroleum ether/EtOAc – PE→40/1).

Yield: 1.66 g (8.6 mmol, 86%).

Physical State: colourless solid.

R_f Value: 0.50 (petroleum ether).

^{1}H NMR (Avance 400 MHz, $CDCl_3$) δ 7.55 – 7.49 (m, 2H), 7.45 – 7.40 (m, 2H), 7.37 – 7.30 (m, 3H), 7.18 – 7.12 (m, 2H), 2.37 (s, 3H) ppm.

^{13}C NMR (Avance 101 MHz, $CDCl_3$) δ 138.4, 131.6, 131.5, 129.1, 128.3, 128.1, 123.5, 120.2, 89.6, 88.7, 21.5 ppm.

IR (ATR, in $CDCl_3$) ν 3080 (w), 3051 (w), 3030 (w), 2919 (w), 2861 (w), 2735 (w), 2216 (w), 1594 (m), 1571 (w), 1509 (s), 1485 (m), 1440 (m), 1310 (w), 1211 (w), 1181 (w), 1106 (w), 1070 (w), 1018 (w) cm^{-1}.

HRMS (EI, m/z) calcd. for $C_{15}H_{12}$: 192.0939, found: 192.0937.

^{1}H- and ^{13}C-NMR spectra of S5

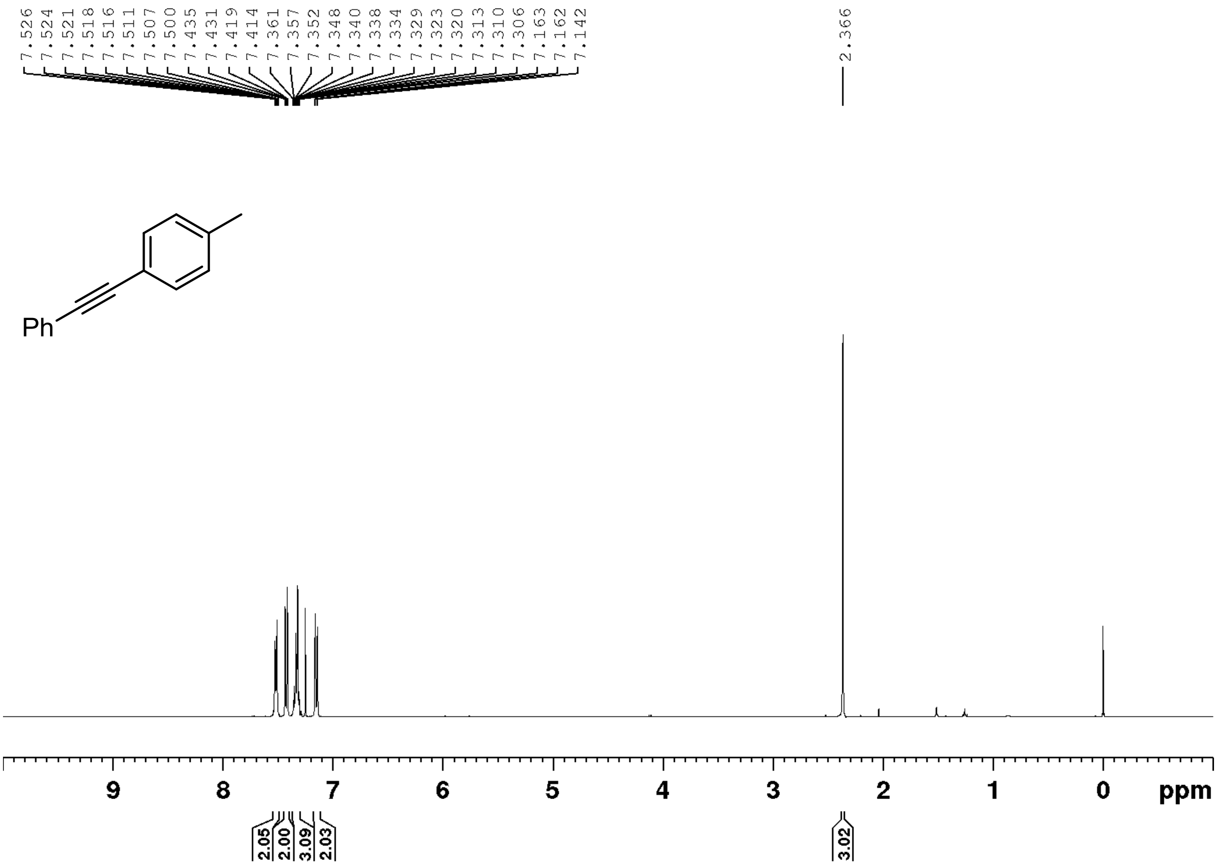

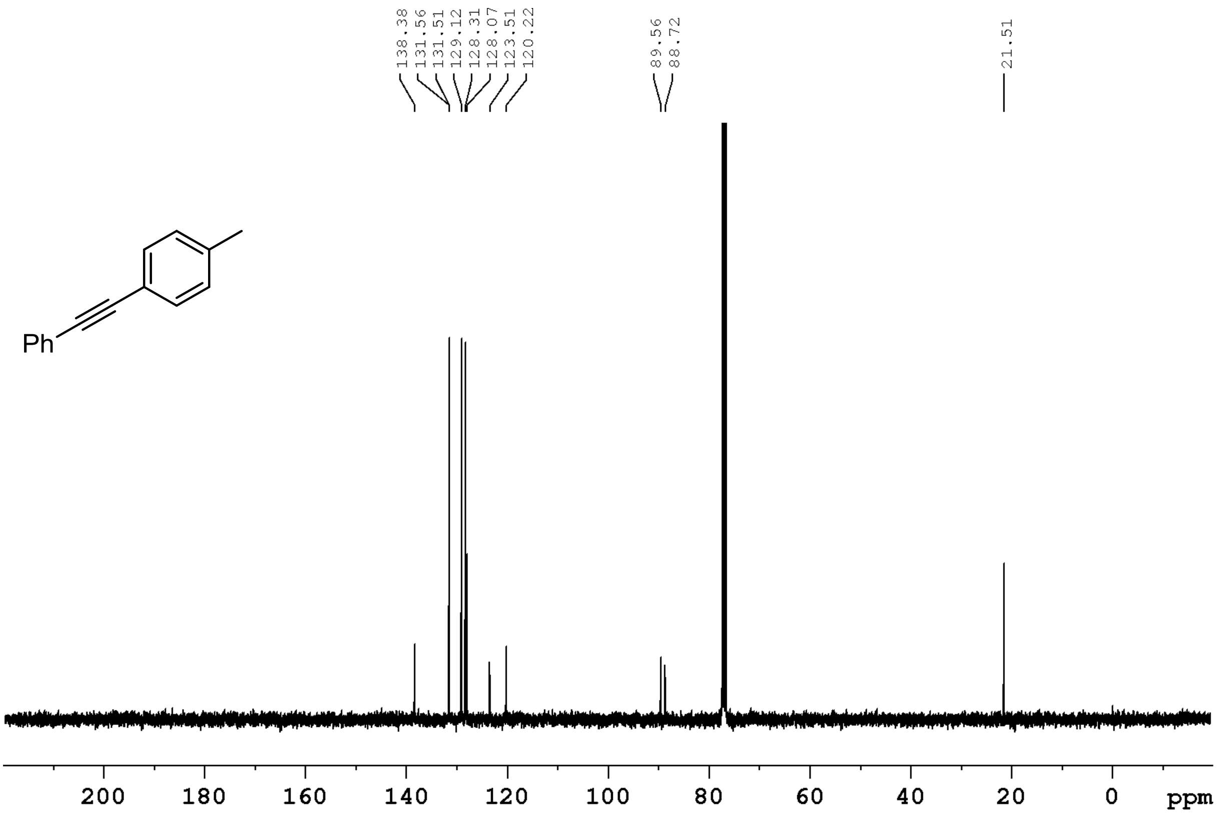

3-(phenylethynyl)acetophenone S6

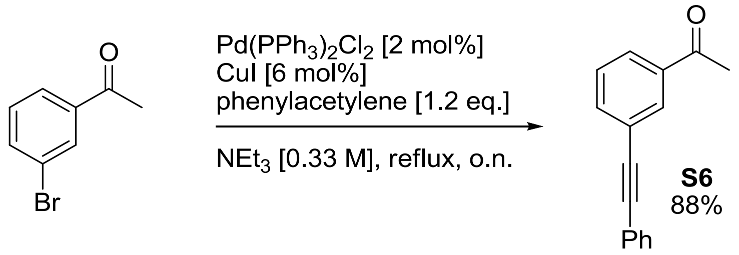

3-(phenylethynyl)acetophenone S6

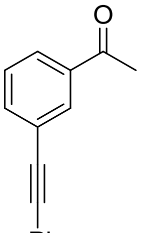

3-Bromoacetophenone (1.990 g, 10 mmol, 1 eq.), $Pd(PPh_3)_2Cl_2$ (140.4 mg, 0.2 mmol, 0.02 eq.) and copper iodide (114.3 mg, 0.6 mmol, 0.06 eq.) were mixed with triethylamine (30 ml) and stirred for 5 min. Phenylacetylene (1.32 ml, 12 mmol, 1.2 eq.) was added and the mixture was refluxed for 15 h. NH_4Cl solution (10 ml) was added and the mixture was extracted with Et_2O (3 x 20 ml). The combined organic layers were dried over anhydrous Na_2SO_4 and

concentrated under reduced pressure. The crude product was purified by column chromatography on silica gel (petroleum ether/EtOAc – 10/1).

Yield: 1.344 g (8.82 mmol, 88%).

Physical State: pale brown crystals.

R_f Value: 0.41 (petroleum ether/EtOAc – 10/1).

^{1}H NMR (Avance 300 MHz, $CDCl_3$) δ 8.11 (t, *J* = 1.7 Hz, 1H), 7.92 (dt, *J* = 7.7, 1.4 Hz, 1H), 7.72 (dt, *J* = 7.7, 1.4 Hz, 1H), 7.58 – 7.53 (m, 2H), 7.46 (t, *J* = 8.0 Hz, 1H), 7.40 – 7.34 (m, 3H), 2.63 (s, 3H) ppm.

^{13}C NMR (Avance 125 MHz, $CDCl_3$) δ 197.5, 137.4, 136.0, 131.8, 131.7, 128.9, 128.8, 128.6, 128.0, 124.1, 122.9, 90.6, 88.4, 26.8 ppm.

IR (ATR, in $CDCl_3$) ν 3062 (w), 1686 (s), 1600 (w), 1573 (w), 1492 (w), 1422 (w), 1357 (m), 1315 (w), 1280 (w), 1244 (s) cm^{-1}.

MS (ESI, m/z): calcd for $[C_{16}H_{12}O+Na]^+$: 243.0780, found: 243.0795.

^{1}H- and ^{13}C-NMR spectra of S6

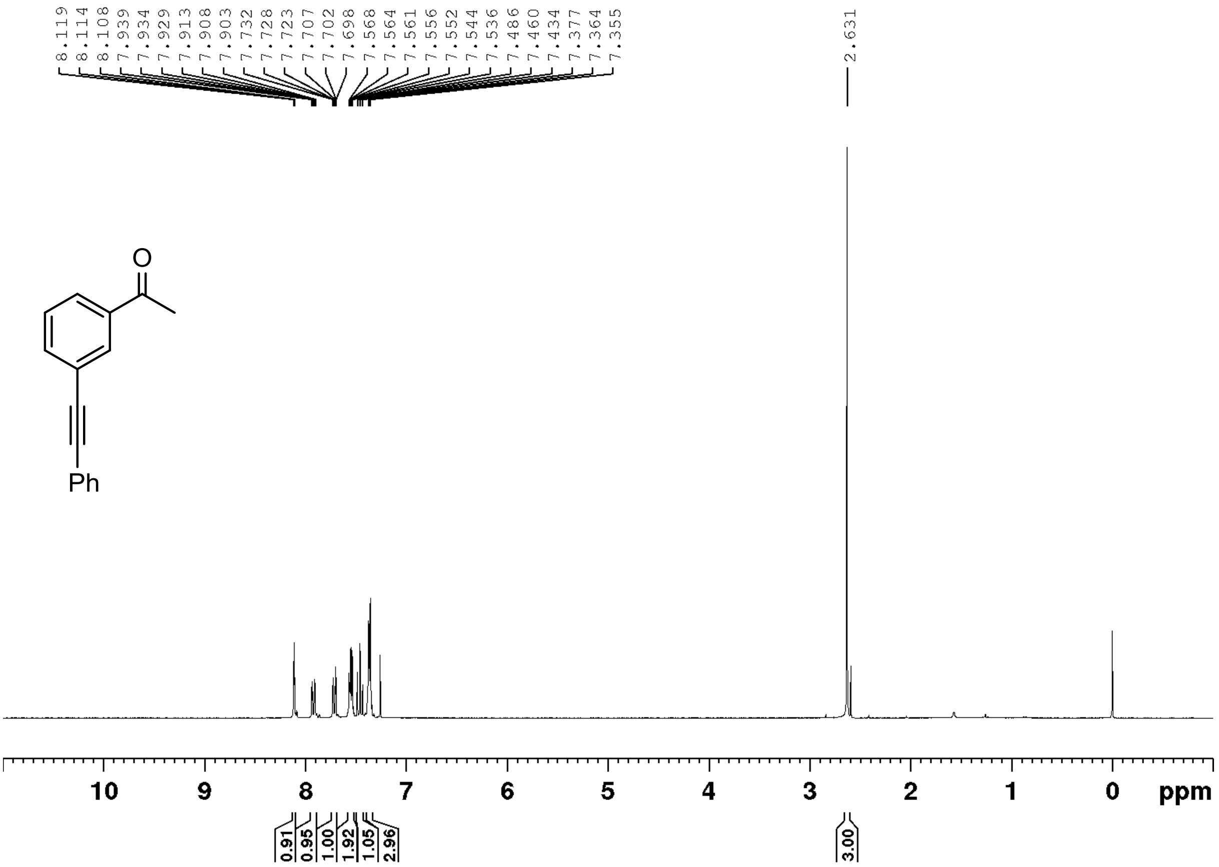

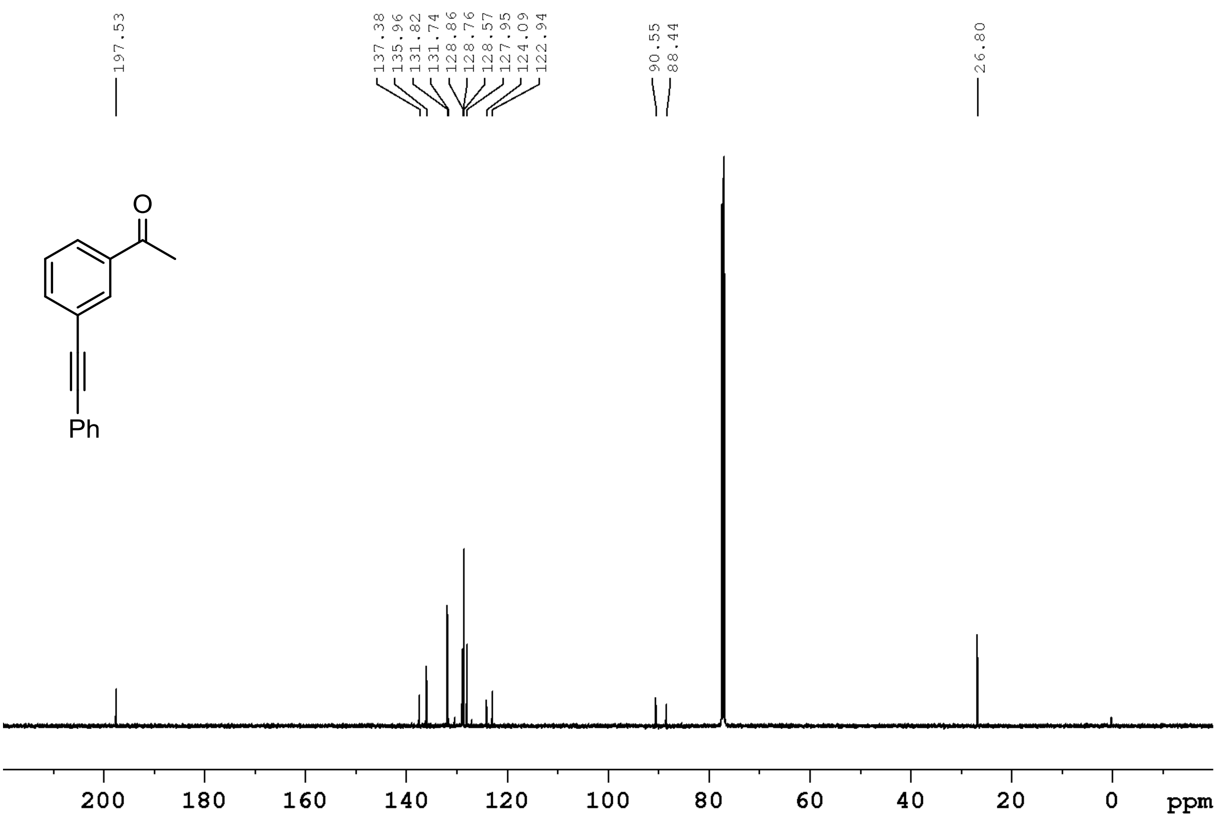

4-(phenylethynyl)anisole S7

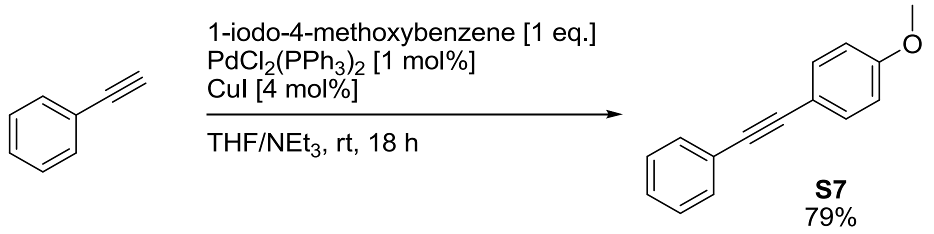

4-(phenylethynyl)anisole S7[1]

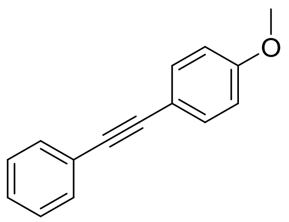

$Pd(PPh_3)_2Cl_2$ (70.2 mg, 0.1 mmol, 0.01 eq.) and copper iodide (76.2 mg, 0.4 mmol, 0.04 eq.) are mixed with THF (10 ml). Phenylacetylene (1.1 ml, 10 mmol, 1 eq.) and 1-iodo-4-methoxybenzene (2.340 g, 10 mmol, 1 eq.) are added. After addition of NEt_3 the reaction mixture was stirred at room temperature for 16 h. After addition of water (25 ml) the aqueous phase was extracted with Et_2O (4 x 25 ml). The combined organic layers were washed with

brine (25 ml), dried over anhydrous Na_2SO_4 and concentrated under reduced pressure. The crude product was purified by column chromatography on silica gel (petroleum ether/EtOAc – 90/1 → 40/1).

Yield: 1.640 g (7.87 mmol, 79%).

Physical State: yellow solid.

R_f Value: 0.6 (petroleum ether/EtOAc – 10/1).

^{1}H NMR (Avance 400 MHz, $CDCl_3$) δ 7.54 – 7.44 (m, 4H), 7.36 – 7.29 (m, 3H), 6.87 (d, *J* = 8.9 Hz, 2H), 3.82 (s, 3H) ppm.

^{13}C NMR (Avance 101 MHz, $CDCl_3$) δ 159.8, 133.2, 131.6, 128.4, 128.1, 123.8, 115.6, 114.2, 89.5, 88.2, 55.5 ppm.

IR (ATR, in $CDCl_3$) ν 3011 (w), 2214 (w), 1605 (m), 1593 (m), 1509 (s), 1440 (m), 1288 (m), 1249 (s), 1176 (m), 1027 (s) cm^{-1}.

MS (EI, 70 eV): *m/z* (%): 208 (100), 193 (46), 165 (35), 139 (8).

^{1}H- and ^{13}C-NMR spectra of S7

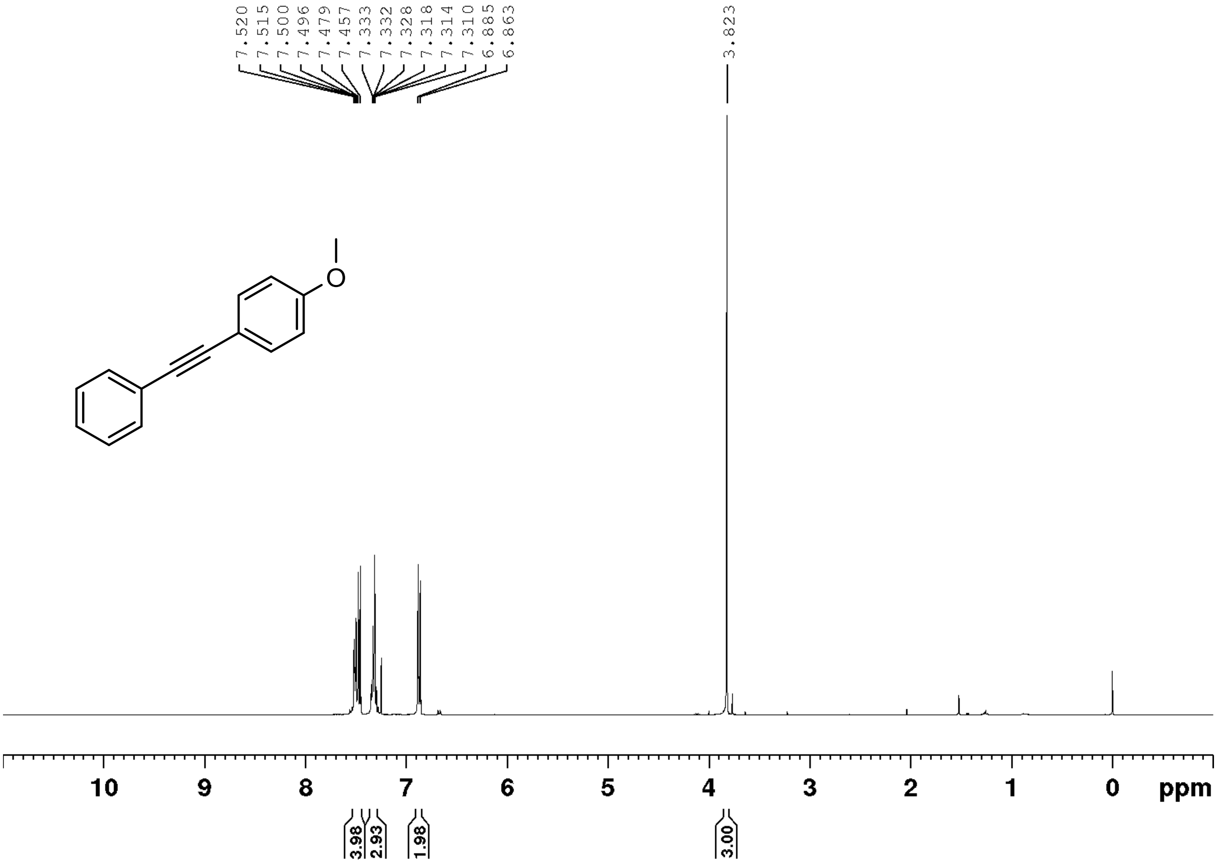

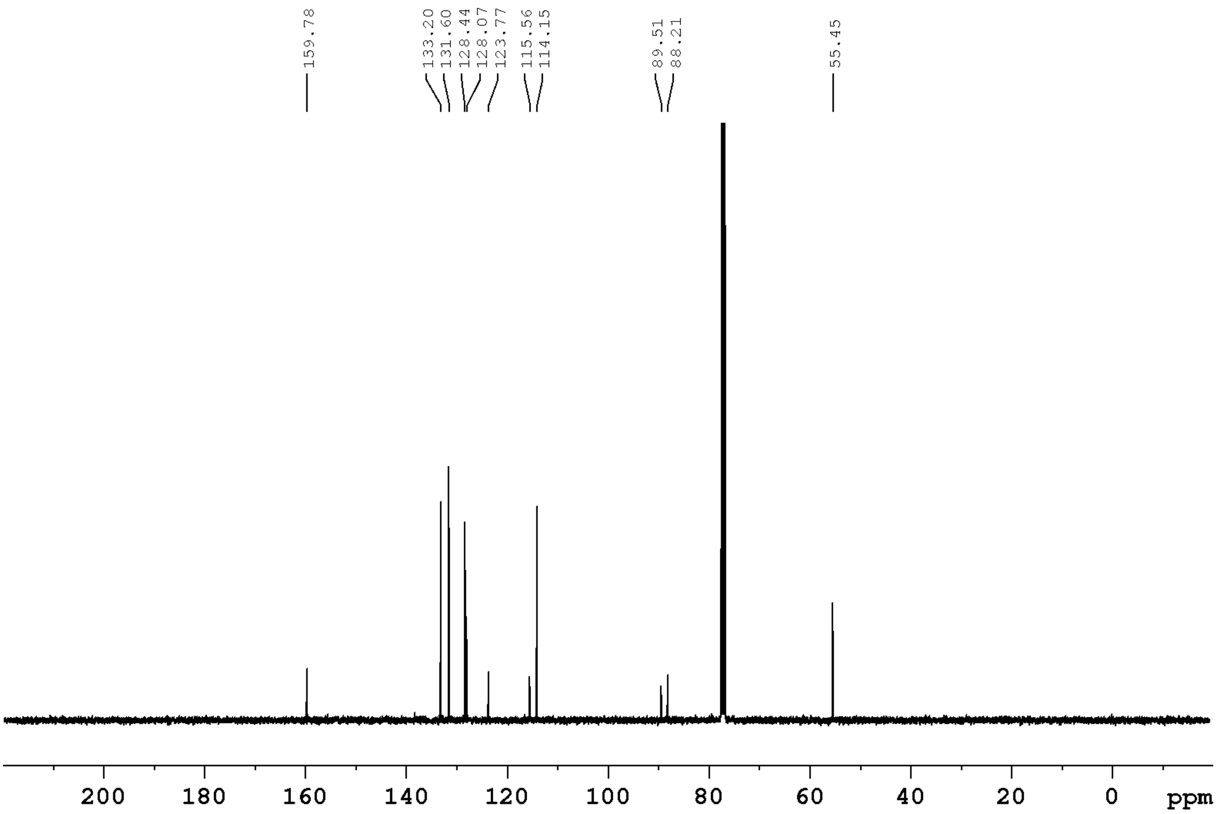

2-(4-methylphenyl)-4,5-dihydro-1,3-oxazole S8

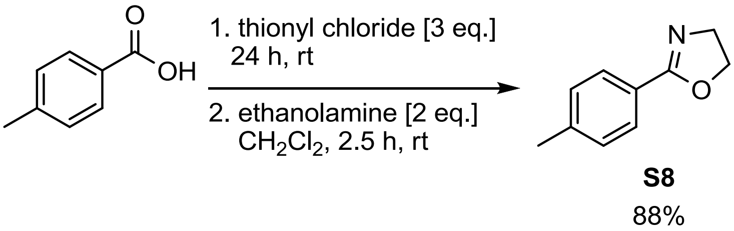

2-(4-methylphenyl)-4,5-dihydro-1,3-oxazole S8[4]

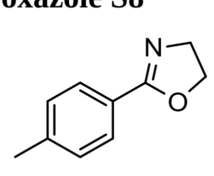

4-Methylbenzoic acid (2.723 g, 20 mmol, 1 eq.) and thionyl chloride (4.37 ml, 60 mmol, 3 eq.) were stirred for 24 h at room temperature. The excess thionyl chloride was removed by water jet pump. The crude product was dissolved in DCM (8.3 ml) and added dropwise at 0 °C to a mixture of ethanolamine (2.4 ml, 40 mmol, 2 eq.) in DCM (5 ml) and stirred for 2.5 h at room temperature. The reaction mixture was filtered and the filtrate was evaporated. The residue was stirred while thionyl chloride (3 ml, 40 mmol, 2 eq.) was added carefully. Afterwards diethylether (20 ml) was added to precipitate the oxazoline hydrochloride. The

product was isolated, neutralized by slow addition of a 20 % NaOH solution and extracted with diethylether, dried over anhydrous $MgSO_4$ and concentrated under reduced pressure.

Yield: 2.85 g (17.7 mmol, 88%).

Physical State: colourless solid.

^{1}H NMR (Avance 400 MHz, $CDCl_3$) δ7.83 (d, *J* = 8.2 Hz, 2H), 7.21 (d, *J* = 7.9, 2H), 4.41 (t, J = 9.4, 2H), 4.04 (t, J = 9.5, 2H), 2.39 (s, 3H) ppm.

^{13}C NMR (Avance 101 MHz, $CDCl_3$) δ 164.7, 141.6, 129.0, 128.1, 125.0, 67.5, 54.9, 21.5 ppm.

IR (ATR, in $CDCl_3$) ν 3035 (w), 2973 (w), 2935 (w), 2904 (w), 2878 (w), 1647 (s), 1611 (m), 1517 (w), 1543 (w), 1514 (m), 1479 (w), 1448 (w), 1409 (w), 1357 (m), 1327 (w), 1311 (w), 1256 (m), 1195 (w), 1183 (w), 116 (w), 1066 (s), 1020 (m) cm^{-1}.

HRMS (ESI, m/z) calcd. for $C_{10}H_{11}NO+H^+$: 162.0913, found: 162.0927.

^{1}H- and ^{13}C-NMR spectra of S8

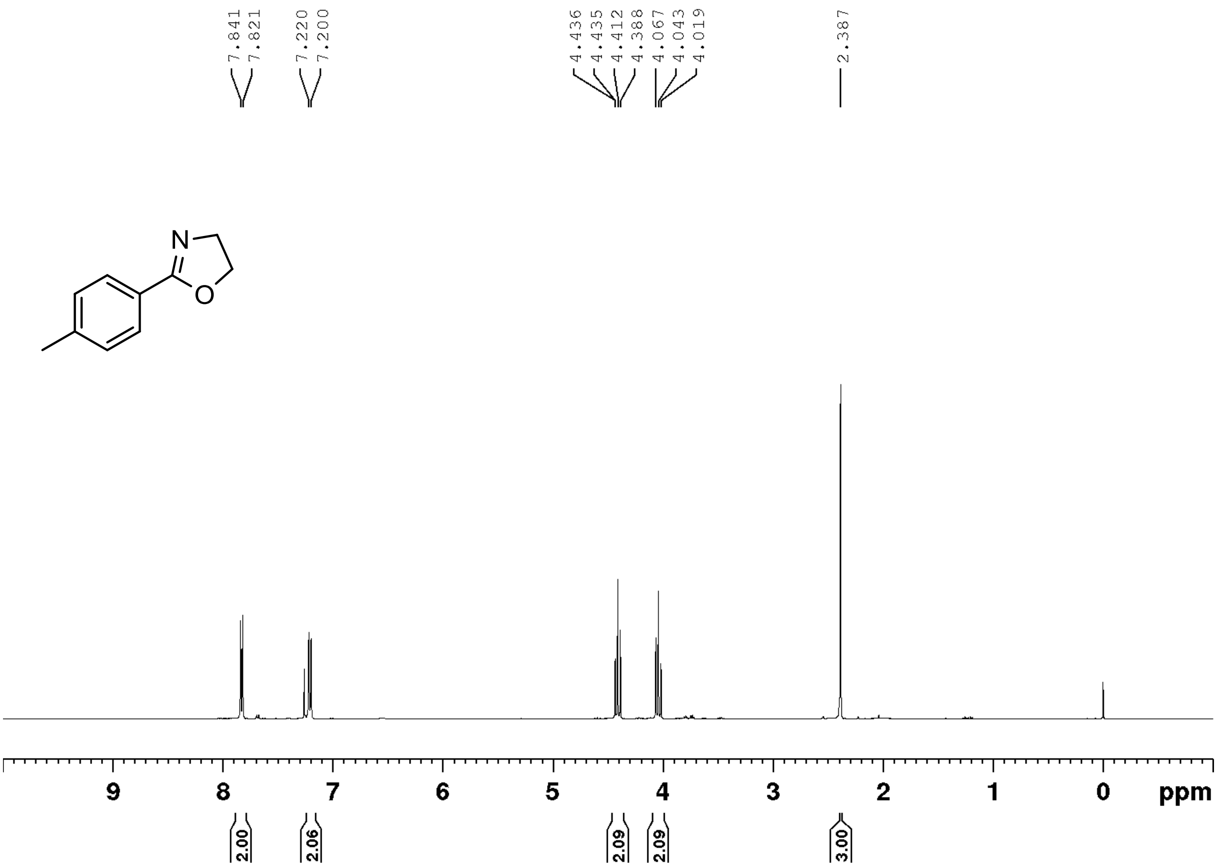

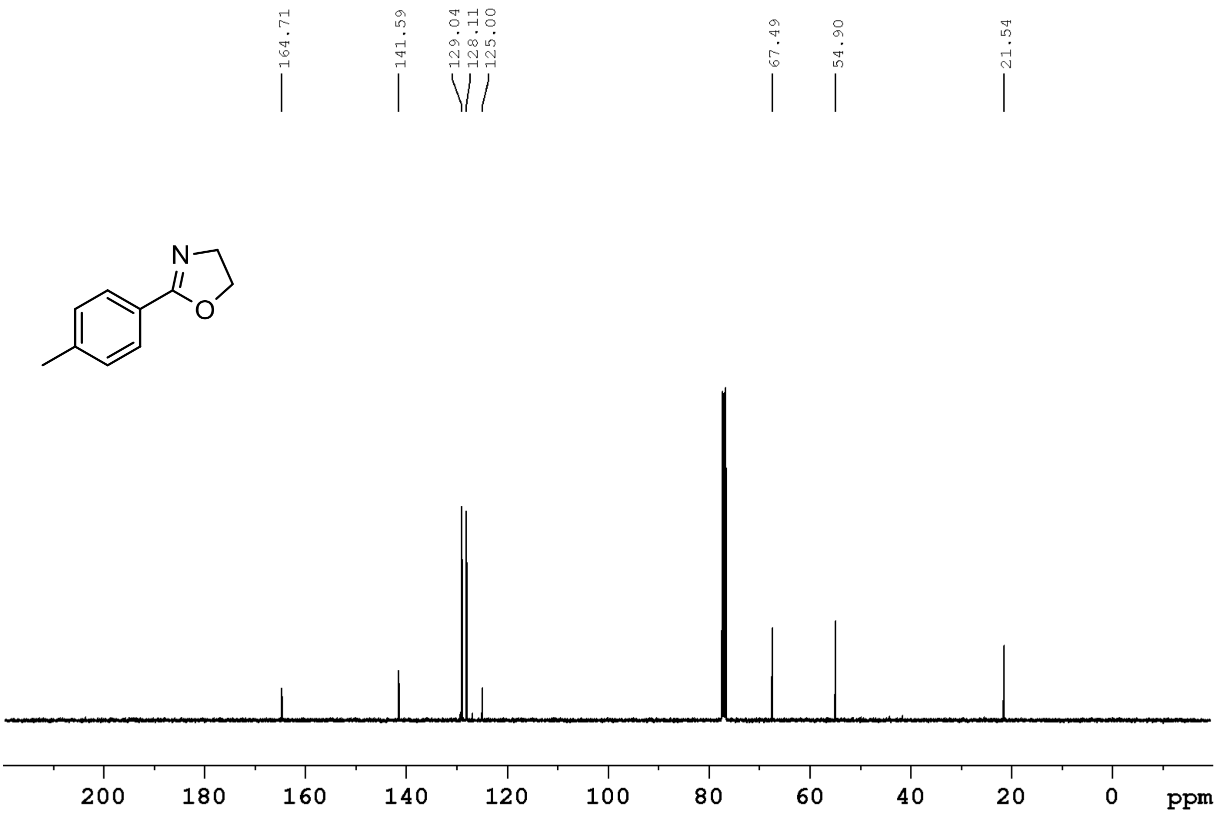

2-(4-chlorophenyl)-4,5-dihydro-1,3-oxazole S9

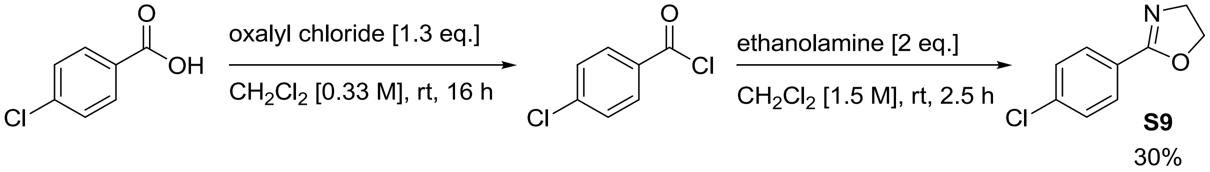

2-(4-chlorophenyl)-4,5-dihydro-1,3-oxazole S9[4]

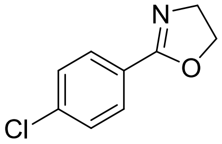

4-Chlorobenzoic acid (3.131 g, 20 mmol, 1 eq.) was dissolved in DCM (60 ml) and 5 drops of DMF were added. The reaction mixture was cooled to 0 °C and stirred for 5 min. Oxalyl chloride (2.23 ml, 26 mmol, 1.3 eq.) was slowly added and the reaction mixture was stirred overnight at room temperature. The solvent was removed under reduced pressure. The crude product was dissolved in DCM (8 ml) and slowly added to a solution of ethanolamine (2.40 ml, 40 mmol, 2 eq.) in DCM (5 ml) at 0 °C. The reaction mixture was stirred for 2.5 h at room temperature. The precipitation was filtered off and the filtrate was concentrated under reduced pressure. The crude product was treated with thionyl chloride (2.91 ml, 40 mmol, 2 eq.) and

Et_2O was added to precipitate the crude product. The filter cake was treated with 20% NaOH solution and extracted with Et_2O (3 x 20 ml). The combined organic layers were dried over anhydrous $MgSO_4$ and concentrated under reduced pressure to obtain the product as yellow crystals without further purification.

Yield: 1.074 g (5.91 mmol, 30% over 2 steps).

Physical State: yellow crystals.

R_f Value: 0.34 (petroleum ether/EtOAc – 1/1).

^{1}H NMR (Avance 300 MHz, $CDCl_3$) δ 7.88 (dt, *J* = 8.8, 2.0 Hz, 2H), 7.39 (dt, *J* = 9.1, 2.1 Hz, 2H), 4.44 (td, *J* = 9.5, 0.7 Hz, 2H), 4.06 (td, *J* = 9.5, 0.8 Hz, 2H) ppm.

^{13}C NMR (Avance 101 MHz, $CDCl_3$) δ 163.9, 137.5, 129.6, 128.7, 126.4, 67.9, 55.1 ppm.

IR (ATR, in $CDCl_3$) ν 2978 (w), 2937 (w), 2882 (w), 1650 (s), 1598 (m), 1490 (m), 1405 (m), 1361 (m), 1260 (m), 1177 (w), 1090 (m), 1066 (s), 1014 (m) cm^{-1}.

HRMS (ESI, m/z) calcd. for $C_9H_8ClNO+H^+$: 182.0367, found:182.0377.

^{1}H- and ^{13}C-NMR spectra of S9

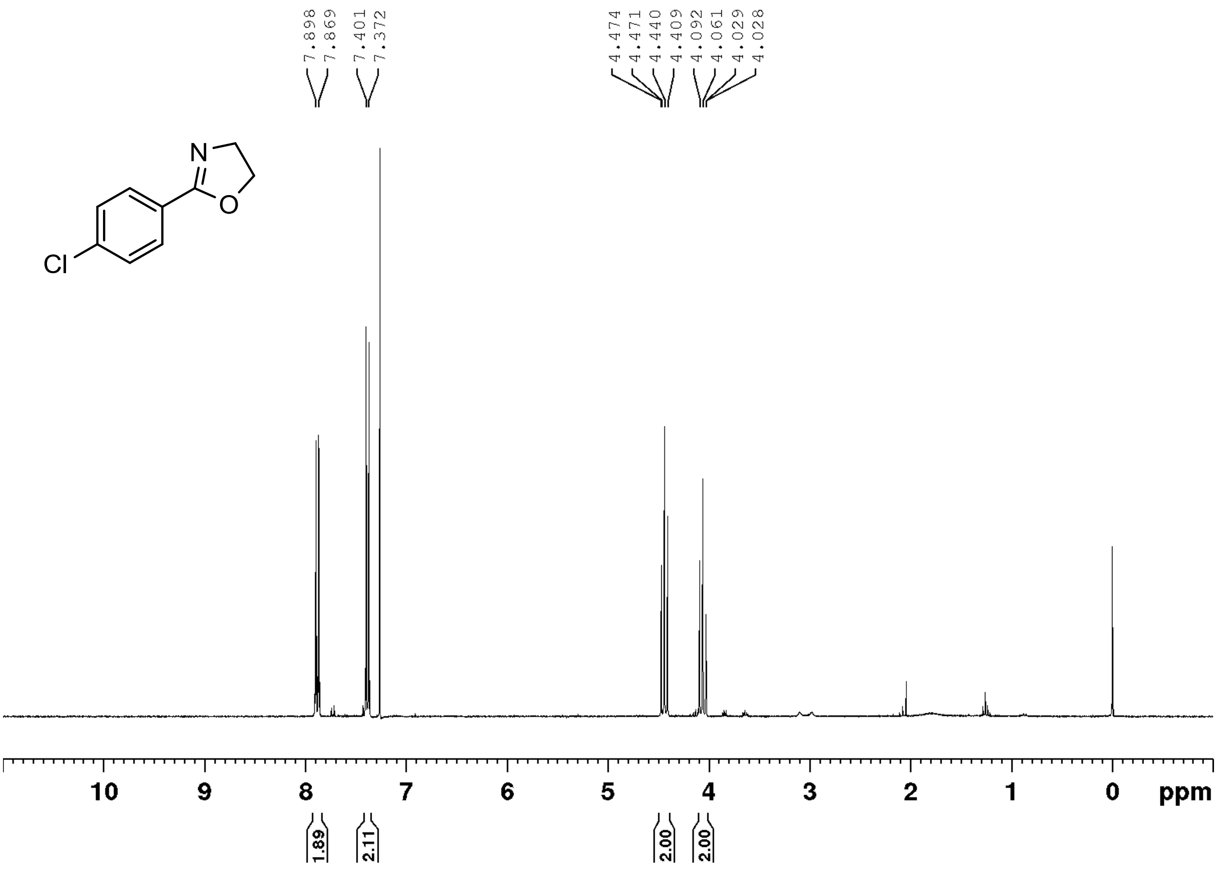

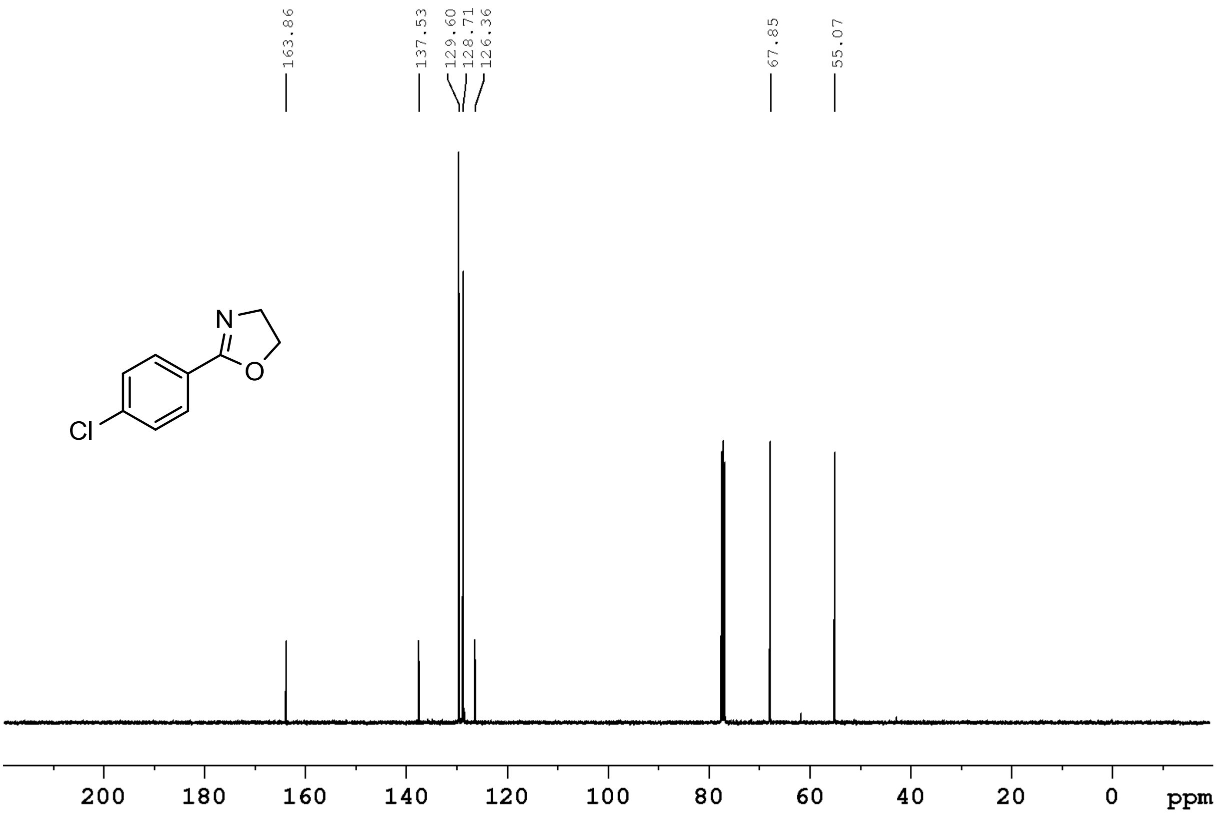

2-(3-chlorophenyl)-4,5-dihydro-1,3-oxazole S10

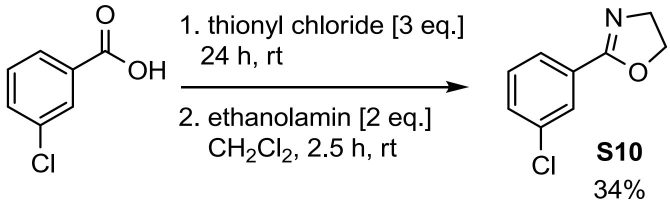

2-(3-chlorophenyl)-4,5-dihydro-1,3-oxazole S10[4]

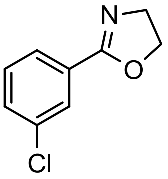

3-Chlorobenzoic acid (1.047 g, 10 mmol, 1 eq.) and thionyl chloride (2.18 ml, 30 mmol, 3 eq.) were stirred for 24 h at room temperature. The excess thionyl chloride was removed by water jet pump. The crude product was dissolved in DCM (4.15 ml) and added dropwise at 0 °C to a mixture of ethanolamine (1.2 ml, 20 mmol, 1 eq.) in DCM (2.5 ml) and stirred for 2.5 h at room temperature. The reaction mixture was filtered and the filtrate was evaporated. The residue was stirred while thionyl chloride (1.5 ml, 20 mmol, 2 eq.) was added carefully. Afterwards diethylether (20 ml) was added to precipitate the oxazolin hydrochloride. The

product was isolated, neutralized by slow addition of a 20 % NaOH solution and extracted with diethylether, dried over anhydrous $MgSO_4$ and concentrated under reduced pressure. The residue was purified by column chromatography on silica gel (petroleum ether/EtOAc – 3/1→1/1).

Yield: 0.61 g (3.4 mmol, 34%).

Physical State: colourless solid.

R_f Value: 0.22 (petroleum ether/EtOAc – 3/1).

^{1}H NMR (Avance 400 MHz, $CDCl_3$) δ 7.99 – 7.90 (m, 1H), 7.87 – 7.79 (m, 1H), 7.48 – 7.40 (m, 1H), 7.38 – 7.30 (m, 1H), 4.44 (t, *J* = 9.5 Hz, 2H), 4.07 (t, *J* = 9.6 Hz, 2H) ppm.

^{13}C NMR (Avance 101 MHz, $CDCl_3$) δ 163.5, 134.4, 131.3, 129.6, 129.5, 128.3, 126.3, 67.8, 55.0 ppm.

IR (ATR, in $CDCl_3$) ν 3069 (w), 2975 (w), 2935 (w), 2904 (w), 2878 (w), 1649 (s), 1599 (w), 1573 (m), 1480 (m), 1432 (m), 1356 (m), 1328 (w), 1297 (w), 1272 (w), 1253 (s), 1195 (w), 1162 (w), 1103 (w), 1078 (m), 1062 (s) cm^{-1}.

HRMS (ESI, m/z) calcd. for $C_9H_8ClNO+H^+$: 182.0367, found: 182.0366.

^{1}H- and ^{13}C-NMR spectra of S10

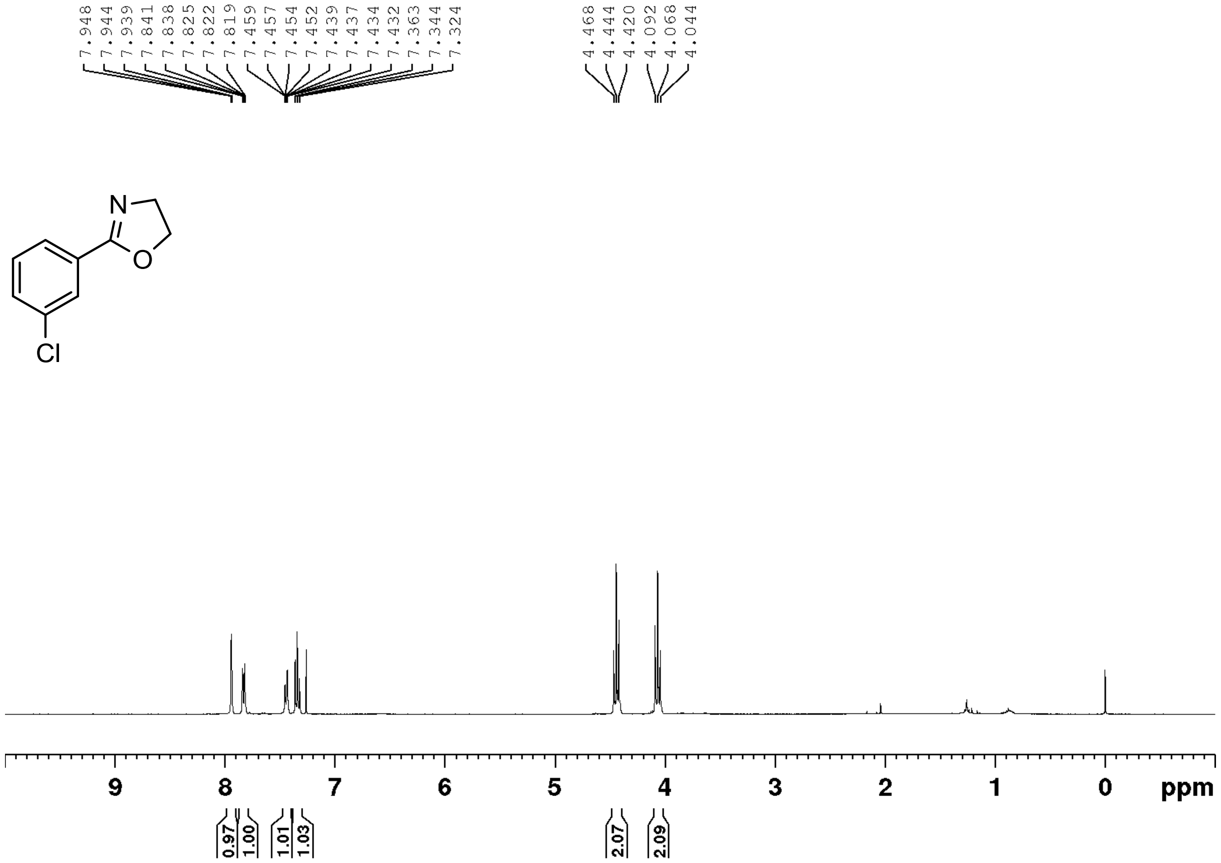

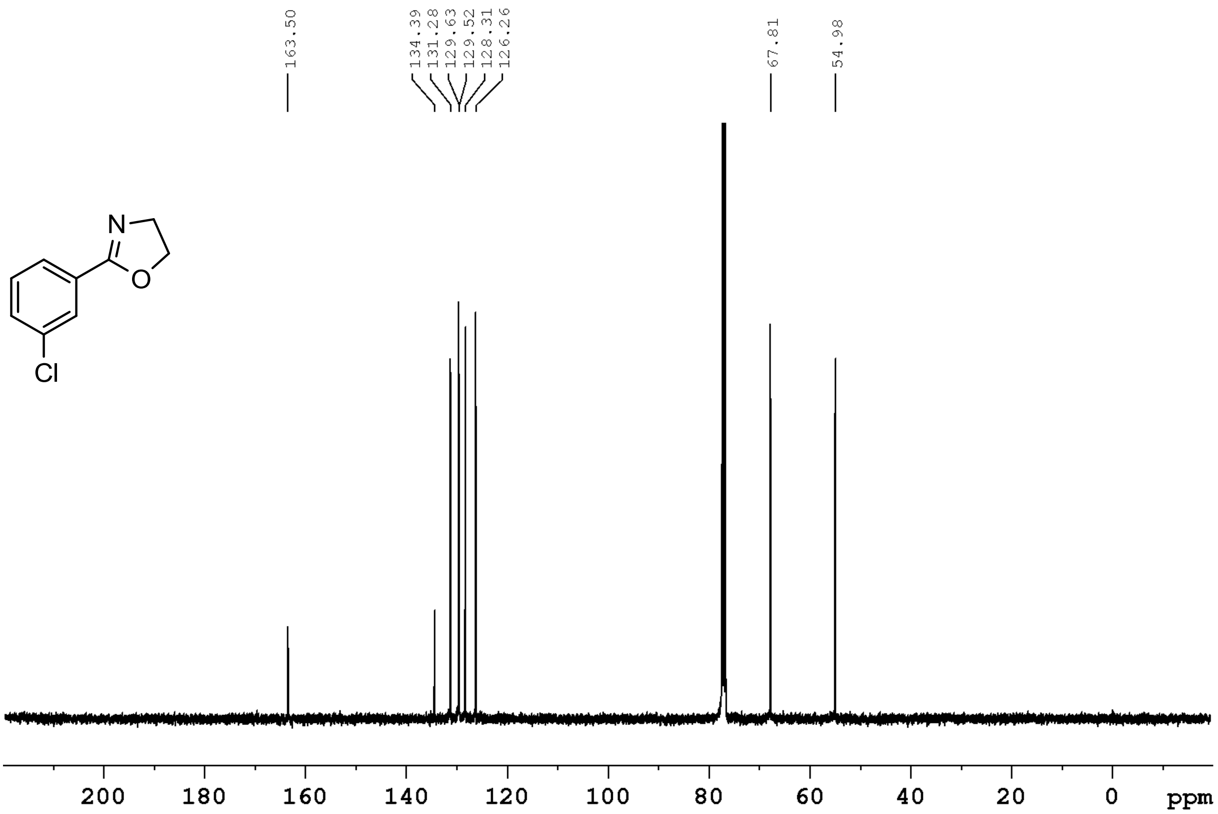

2-(4-methylphenyl)-1,3-dioxolane S11

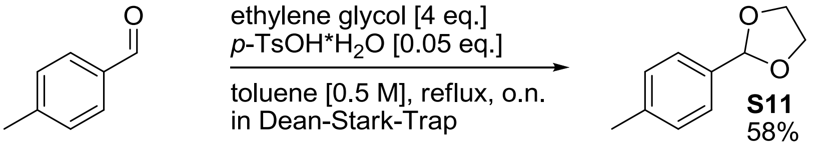

2-(4-methylphenyl)-1,3-dioxolane S11[5]

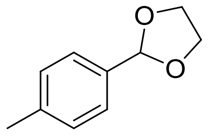

4-Methylbenzaldehyde (0.83 ml, 7 mmol, 1 eq.) and *p*-toluenesulfonic acid monohydrate (66.6 mg, 0.35 mmol, 0.05 eq.) were dissolved in toluene (15 ml). Ethylene glycol (1.57 ml) was added and the mixture was refluxed over night in a Dean–Stark apparatus. The reaction mixture was cooled to room temperature and a sat. $NaHCO_3$-solution (10 ml) was added. The aqueous phase was extracted with Et_2O (3 x 10 ml) and the combined organic phase was washed with brine (10 ml), dried over anhydrous Na_2SO_4 and concentrated under reduced pressure. The crude product was purified by column chromatography on silica gel (petroleum ether/EtOAc – 10/1→5/1).

Yield: 671 mg (4.08 mmol, 58%).

Physical State: colourless oil.

R_f Value: 0.21 (petroleum ether/EtOAc – 10/1).

^{1}H NMR (Avance 400 MHz, $CDCl_3$) δ 7.36 (d, *J* = 7.8 Hz, 2H), 7.18 (d, *J* = 7.8 Hz, 2H), 5.78 (s, 1H), 4.12 (t, *J* = 7.0 Hz, 2H), 4.02 (t, *J* = 7.0 Hz, 2H), 2.35 (s, 3H) ppm.

^{13}C NMR (Avance 101 MHz, $CDCl_3$) δ 139.1, 135.1, 129.2, 126.5, 103.9, 65.4, 21.4 ppm.

IR (ATR, in $CDCl_3$) ν 3371 (b), 2883 (w), 1702 (m), 1687 (m), 1604 (w), 1386 (m), 1208 (m), 1168 (w), 1077 (s), 1021 (m) cm^{-1}.

HRMS (ESI, m/z) calcd. for $C_{10}H_{12}O_2$-H^+: 163.0759, found: 163.0757.

^{1}H- and ^{13}C-NMR spectra of S11

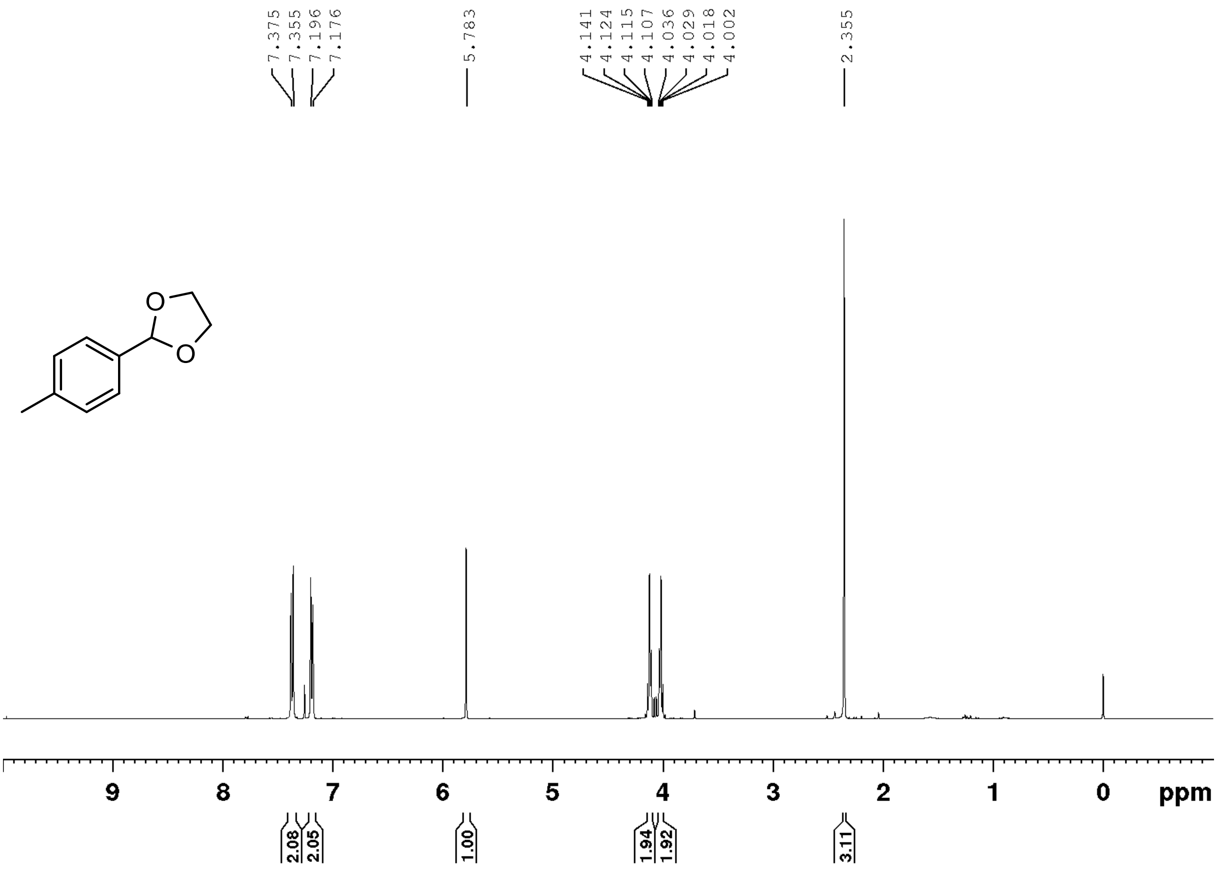

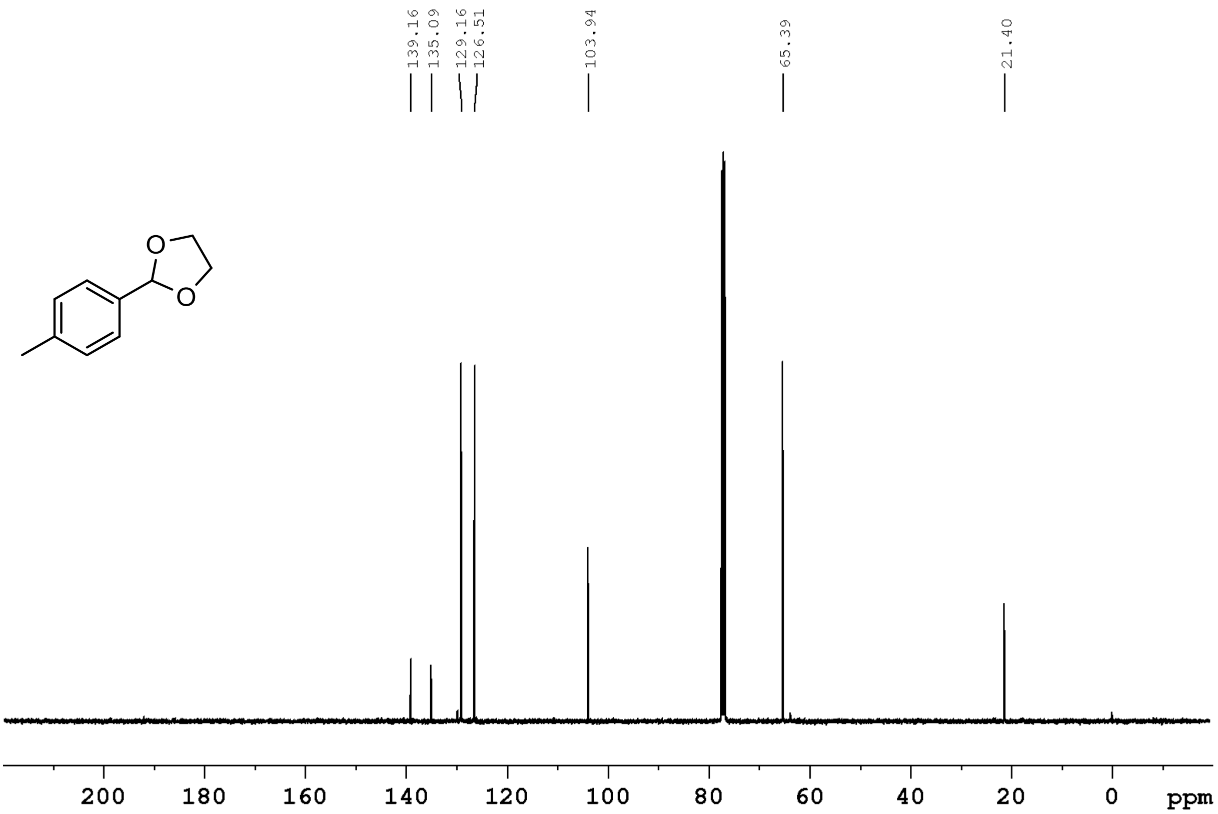

2-(4-methylphenyl)-1,3-dioxolane S12

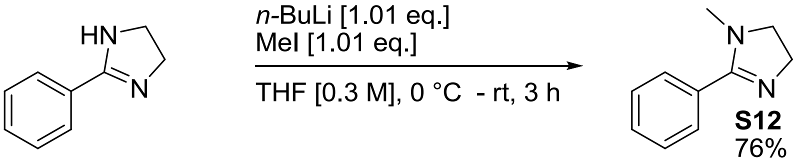

2-(4-methylphenyl)-1,3-dioxolane S12[6]

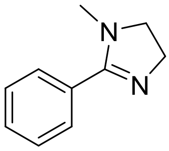

2-Phenyl-2-imidazoline (731 mg, 5 mmol, 1 eq.) was dissolved in THF (17 ml) and cooled to -78 °C. *n*-Butyllithium (3.16 ml, 1.6 M in Hexane, 5.05 mmol, 1.01 eq.) was added dropwise and the mixture was stirred for 45 min. Methyl iodide (0.32 ml, 5.05 mmol, 1.01 eq.) was added and the reaction was stirred at room temperature for 5 h. The solvent was evaporated under reduced pressure. The residue was dissolved in DCM (10 ml) and washed with water (3 x 10 ml). The organic phase was dried over anhydrous $MgSO_4$ and concentrated under reduced pressure. The crude product was purified by colum chromatography on silica gel ($DCM/MeOH/NEt_3$ - 60/5/1).

Yield: 611 mg (3.81 mmol, 76%).

Physical State: colourless oil.

R_f Value: 0.14 (DCM/MeOH/NEt_3 - 60/5/1).

^{1}H NMR (Avance 400 MHz, $CDCl_3$) δ 7.57 – 7.52 (m, 2H), 7.43 – 7.37 (m, 3H), 3.88 (t, *J* = 9.9 Hz, 2H), 4.46 (t, *J* = 9.9 Hz, 2H), 2.81 (s, 3H) ppm.

^{13}C NMR (Avance 101 MHz, $CDCl_3$) δ 168.3, 131.1, 130.0, 128.5, 128.3, 54.2, 53.0, 36.6 ppm.

IR (ATR, in $CDCl_3$) ν 2828 (w), 2861 (w), 1611 (m), 1594 (s), 1447 (w), 1330 (w), 1276 (s), 1184 (w), 1060 (s), 1025 (m) cm^{-1}.

HRMS (ESI, m/z) calcd. for $C_{10}H_{12}N_2+H^+$: 161.1073, found: 161.1084.

^{1}H- and ^{13}C-NMR spectra of S12

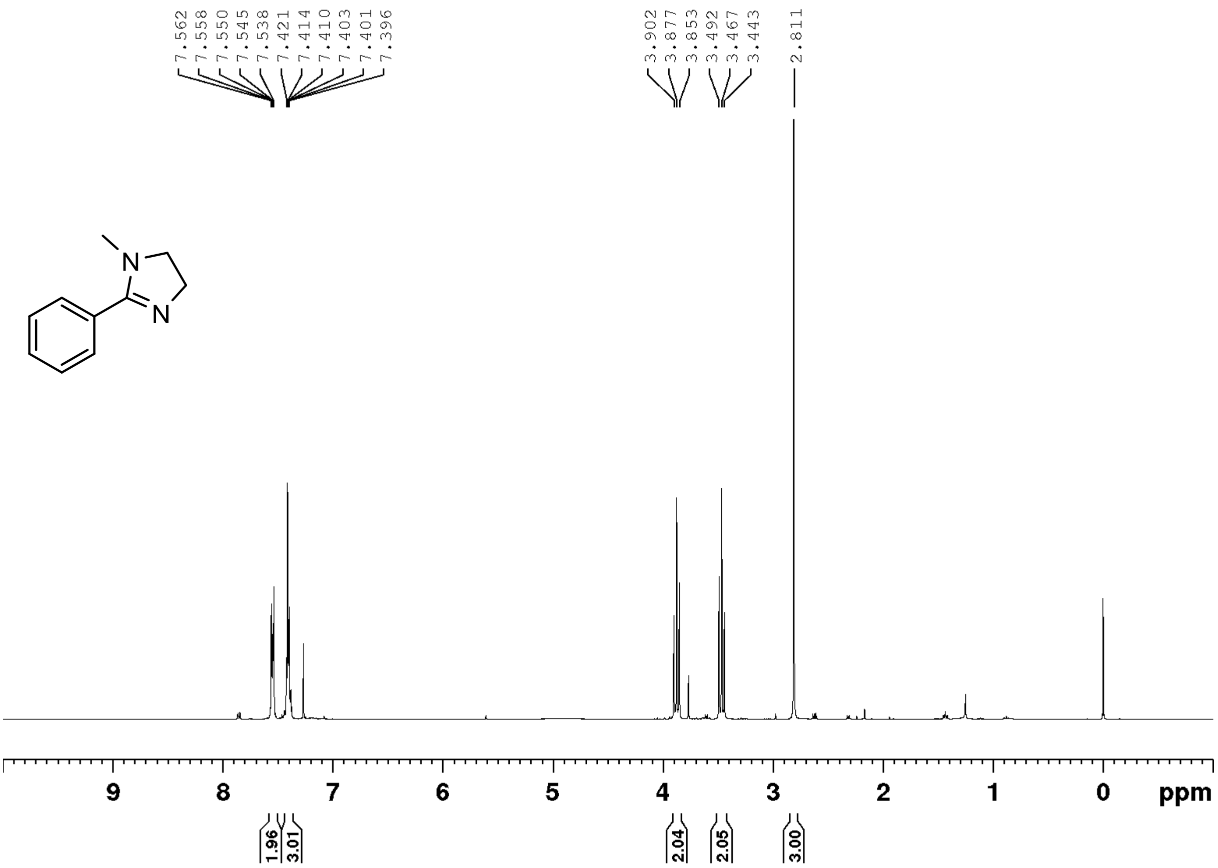

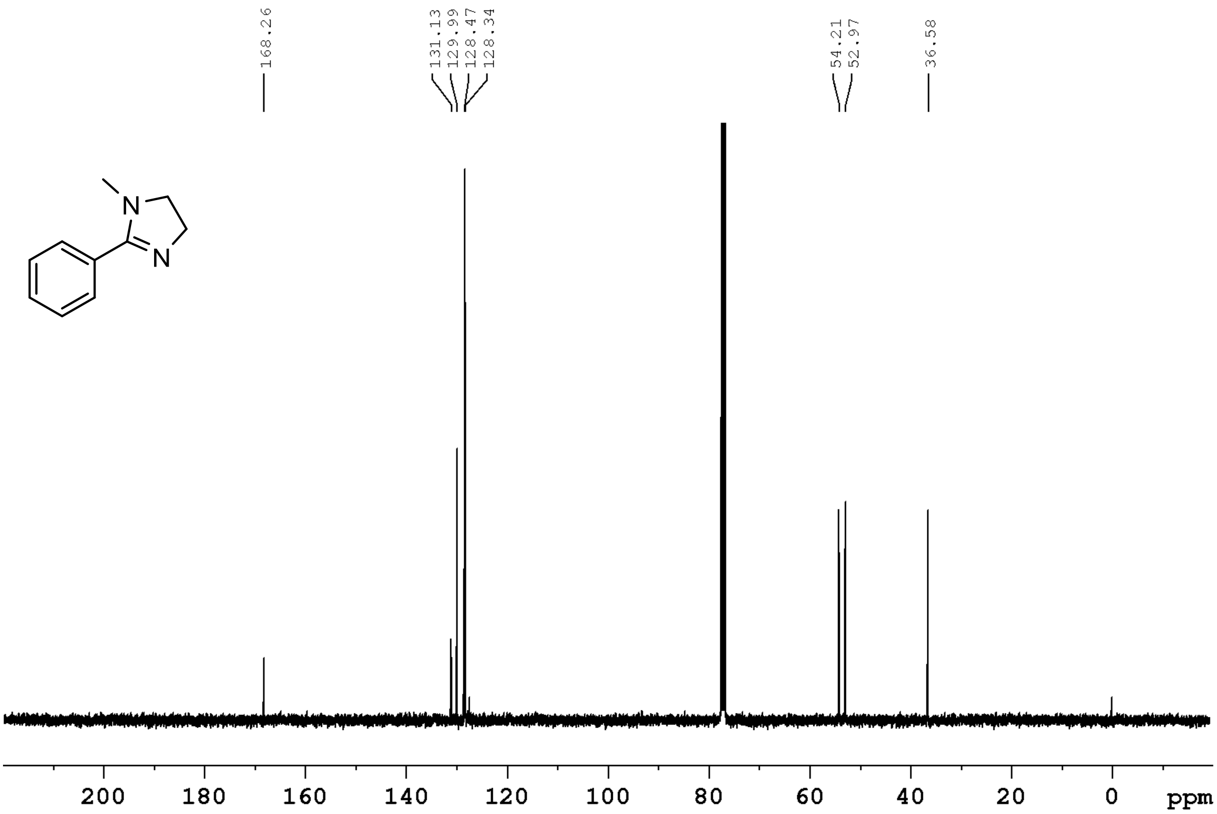

N-methyl-2-phenyl-1H-imidazole S13

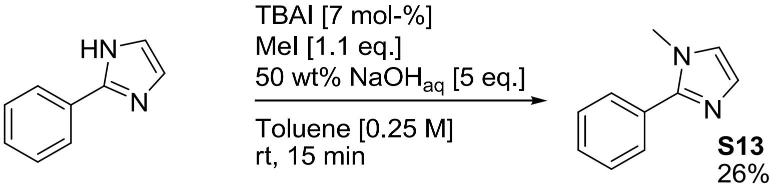

N-methyl-2-phenyl-1H-imidazole S13[7]

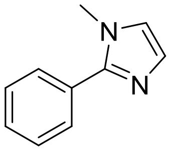

2-Phenylimidazole (721 mg, 5 mmol, 1 eq.) was dissolved in toluene (25 ml) and TBAI (129 mg, 0.35 mmol, 0.07 eq) was added. An aqueous NaOH solution (25 ml, 50 wt%, 5 eq.) was added and the biphasic system was stirred vigorously. MeI (0.32 ml, 5.05 mmol, 1.1 eq.) was added and the mixture was stirred for 15 min at room temperature. After the adding of additional toluene (20 ml) and water (20 ml) the mixture was extracted with DCM (3x 20 ml). The combined organic phase was dried over anhydrous $MgSO_4$ and the solvent was evaporated under reduced pressure. The crude product was purified by column chromatography on silica gel (EtOAc → EtOAc/MeOH 100:5).

Yield: 209 mg (1.32 mmol, 26%).

Physical State: orange oil.

R_f Value: 0.30 (EtOAc).

^{1}H NMR (Avance 400 MHz, $CDCl_3$) δ 7.65 – 7.61 (m, 2H), 7.48 – 7.38 (m, 3H), 7.12 (d, *J* = 1.3 Hz, 1H), 6.97 (d, *J* = 1.3 Hz, 1H), 3.74 (s, 3H) ppm.

^{13}C NMR (Avance 101 MHz, $CDCl_3$) δ 148.0, 130.7, 128.8, 128.8, 128.6, 128.5, 122.4, 34.6 ppm.

IR (ATR, in $CDCl_3$) ν 1499 (w), 1475 (m), 1405 (m), 1279 (m), 1139 (w), 1075 (w), 1019 (w) cm^{-1}.

HRMS (ESI, m/z) calcd. for $C_{10}H_{10}N_2$+H^+: 159.0917, found: 159.0932.

^{1}H- and ^{13}C-NMR spectra of S13

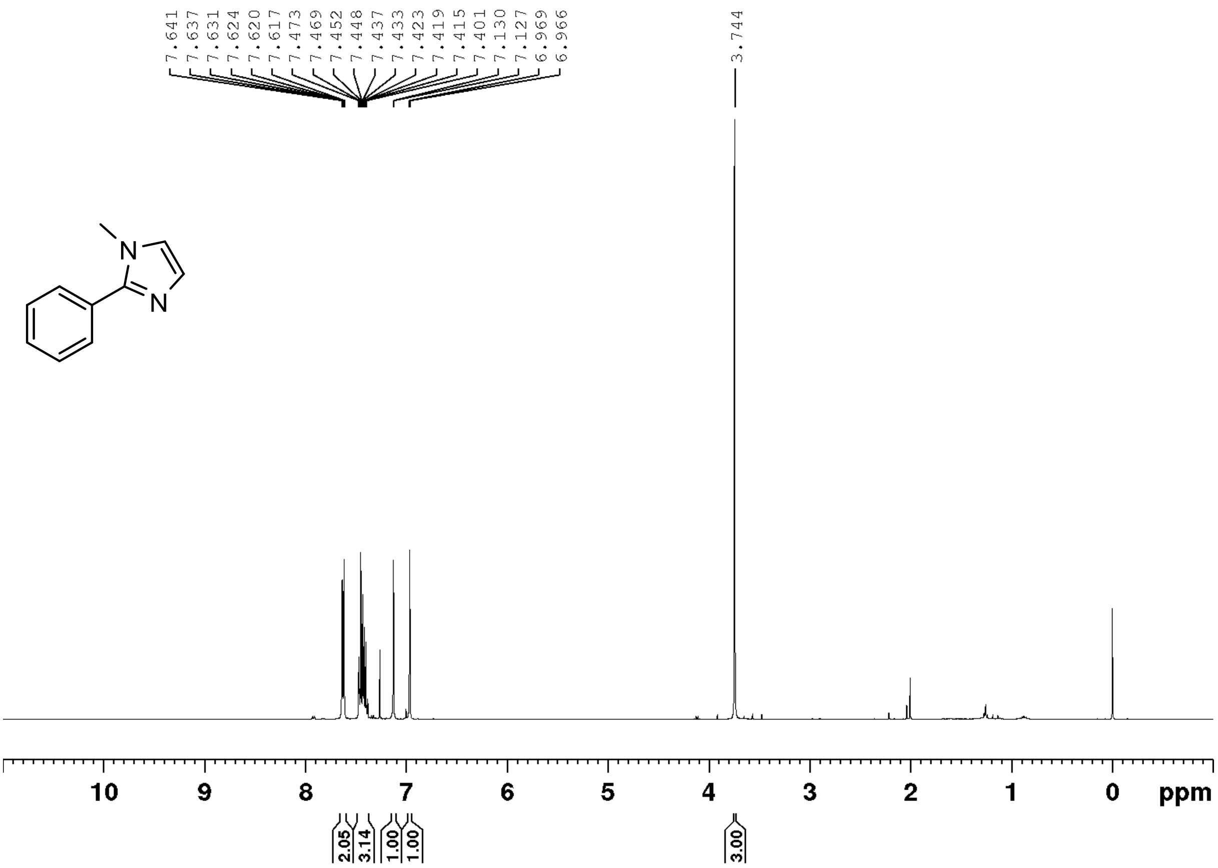

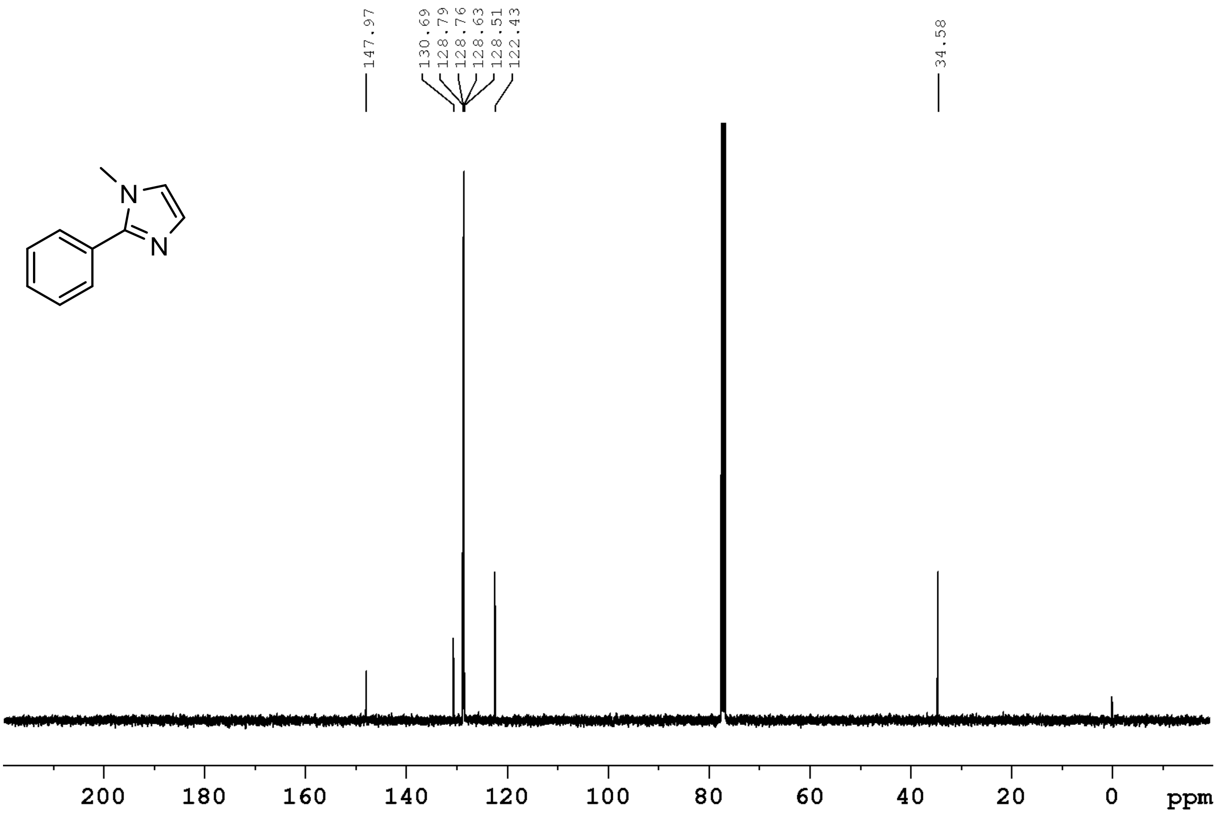

3. Synthesis of reference compounds

2-(3-chlorophenyl)-4,5-dihydro-1,3-oxazole R1

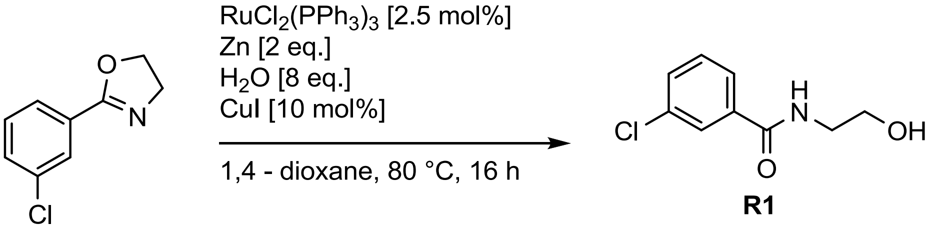

2-(3-chlorophenyl)-4,5-dihydro-1,3-oxazole R1

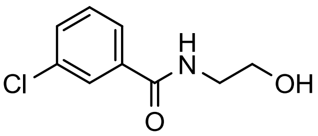

According to **GP-I**: **S10** (90.8 mg, 0.5 mmol, 1 eq.), $RuCl_2(PPh_3)_3$ (12.0 mg, 0.0125 mmol, 2.5 mol%), Zn (65.4 mg, 1 mmol, 2 eq.), CuI (9.5 mg, 0.05 mmol, 10 mol%) and H_2O (80 µl, 4 mmol, 8 eq.) were dissolved in dry 1,4-dioxane (1 ml) and stirred for 16 h at 80 °C. The product was cristallized in chloroform.

Physical State: colourless solid.

^{1}H NMR (Avance 400 MHz, DMSO) δ 8.55 (t, *J* = 4.9 Hz, 1H), 7.90 (t, *J* = 1.8 Hz, 1H), 7.81 (dt, *J* = 7.8, 1.2 Hz) 7.62 – 7.56 (m, 1H), 7.54 – 7.46 (m, 1H), 4.72 (t, *J* = 5.4, 1H), 3.56 – 3.47 (m, 2H), 3.36 – 3.29 (m, 2H) ppm.

^{13}C NMR (Avance 75 MHz, DMSO) δ 164.8, 136.5, 133.1, 130.9, 130.2, 127.0, 125.9, 59.6, 42.2 ppm.

IR (ATR, neat) ν 3376 (m), 3309 (m), 3086 (w), 3063 (w), 2940 (m), 2876 (w), 2713 (w), 1629 (s), 1599 (m), 1547 (s), 1473 (m), 1459 (m), 1433 (m), 1398 (w), 1376 (m), 1363 (m), 1322 (m), 1218 (m), 1166 (w), 1094 (m), 1063 (s), 1045 (m), 1000 (m) cm^{-1}.

HRMS (ESI, m/z) calcd. for $C_9H_{10}ClNO_2+Na^+$: 222.0292, found: 222.0295.

^{1}H- and ^{13}C-NMR spectra of R1

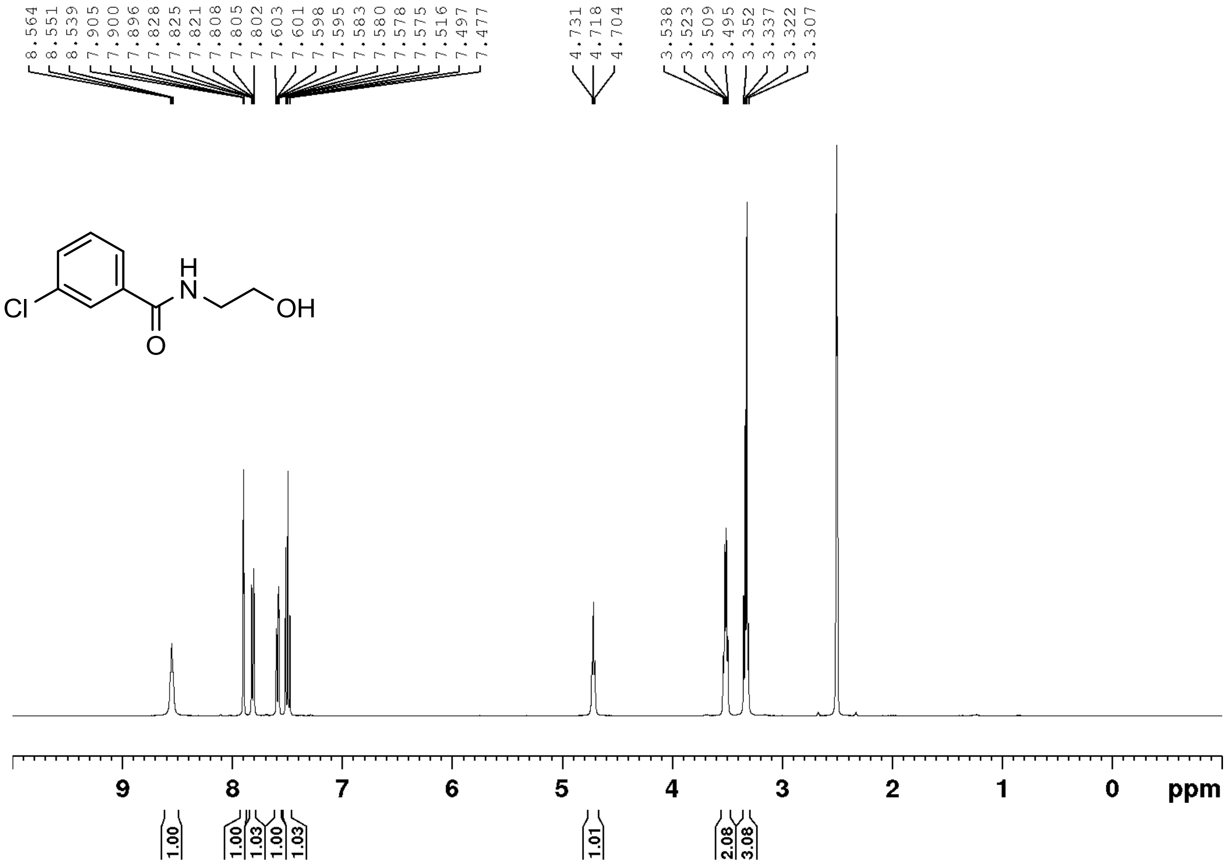

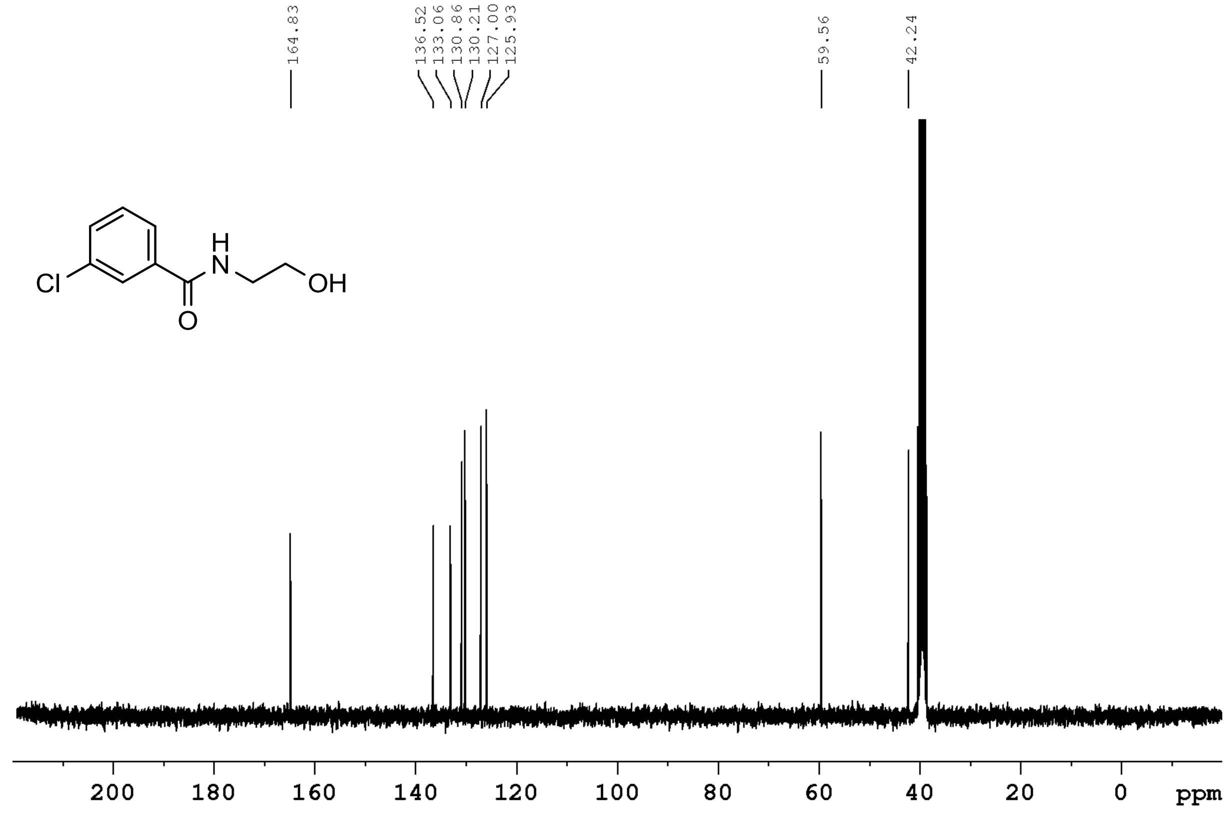

1-[3-(2-pyridyl)phenyl]ethanol R2

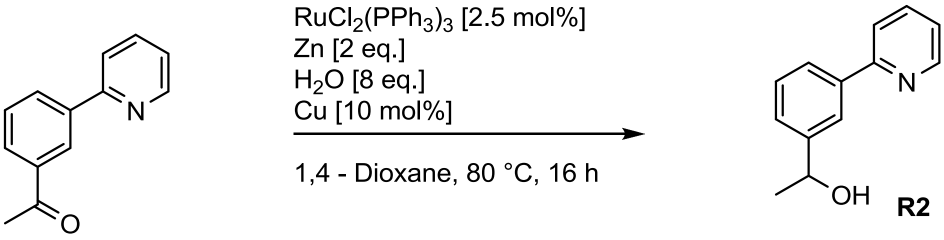

1-[3-(2-pyridyl)phenyl]ethanol R2

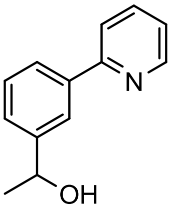

According to **GP-I**: **S4** (98.6 mg, 0.5 mmol, 1 eq.), $RuCl_2(PPh_3)_3$ (12.0 mg, 0.0125 mmol, 2.5 mol%), Zn (65.4 mg, 1 mmol, 2 eq.), CuI (9.5 mg, 0.05 mmol, 10 mol%) and H_2O (80 µl, 4 mmol, 8 eq.) were dissolved in dry 1,4-dioxane (1 ml) and stirred for 16 h at 80 °C and purified by column chromatography (petroleum ether/EtOAc – 3/1).

Yield: 45.1 mg (0.23 mmol, 45%).

Physical State: colourless liquid.

R_f Value: 0.14 (petroleum ether/EtOAc – 3/1).

^{1}H NMR (Avance 400 MHz, CD_2Cl_2) δ 8.52 (d, J = 4.56 Hz, 1H), 7.90 (s, 1H), 7.78 – 7.71 (m, 1H), 7.68 – 7.57 (m, 2H), 7.35 – 7.26 (m, 2H), 7.16 – 7.06 (m, 1H), 4.53 (q, J = 6.47 Hz, 1H), 2.79 (s, 1H), 1.38 (d, J = 6.48 Hz, 3H) ppm.

^{13}C NMR (Avance 75 MHz, CD_2Cl_2) δ 157.6, 149.9, 147.3, 139.8, 137.2, 129.1, 126.4, 126.1, 124.4, 122.6, 120.9, 70.4, 25.6 ppm.

IR (ATR, in CD_2Cl_2) ν 3301 (broad), 3058 (w), 2969 (w), 2925 (w), 2867 (w), 1584 (m), 1565 (m), 1462 (m), 1434 (m), 1416 (m), 1367 (w), 1292 (w), 1188 (m), 1153 (w), 1108 (w), 1070 (m), 1012 (w) cm^{-1}.

HRMS (ESI, m/z) calcd. for $C_{13}H_{13}NO+H^+$: 200.1070, found: 200.1068.

^{1}H- and ^{13}C-NMR spectra of R2

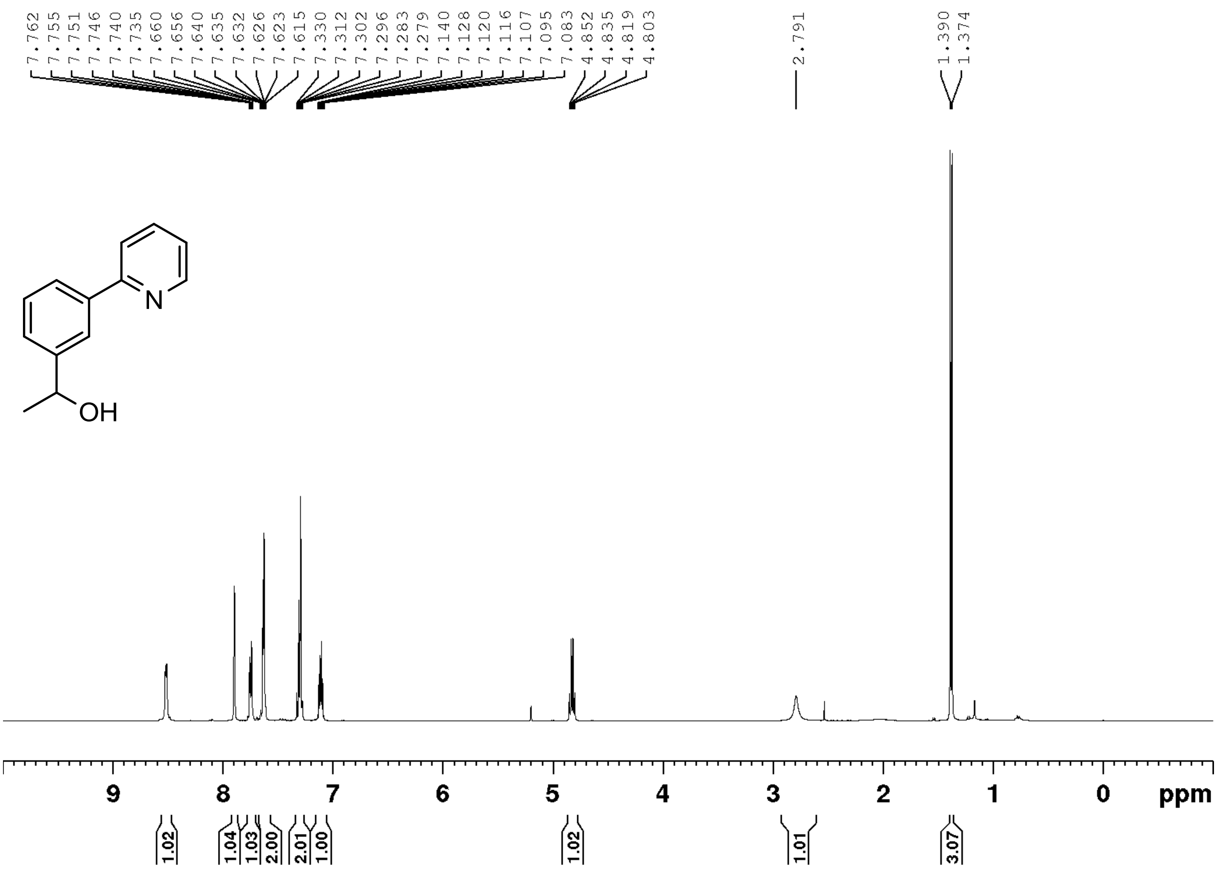

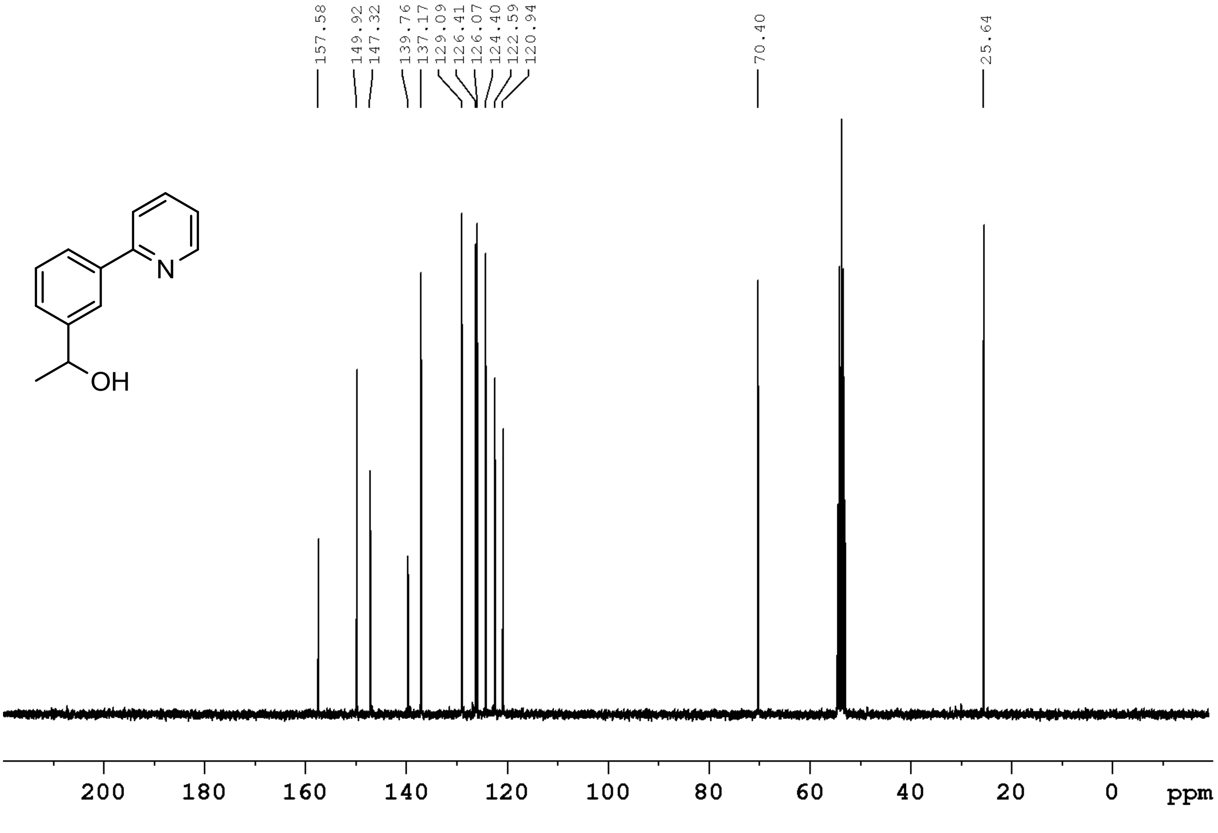

(*E*/*Z*)-2-(3-styrylphenyl)pyridine

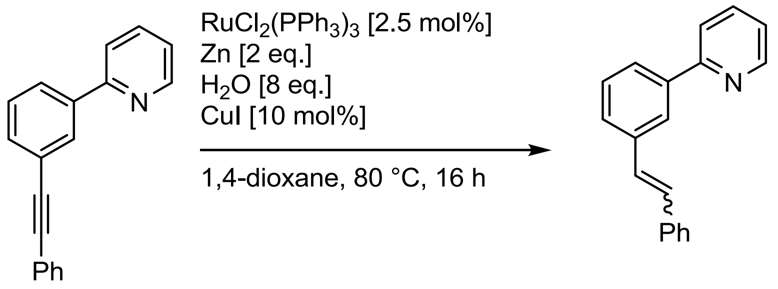

(*Z*)-2-(3-styrylphenyl)pyridine R3

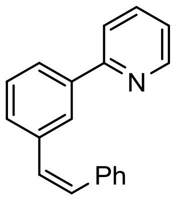

According to **GP-I**: **S3** (127.7 mg, 0.5 mmol, 1 eq.), $RuCl_2(PPh_3)_3$ (12.0 mg, 0.0125 mmol, 2.5 mol%), Zn (65.4 mg, 1 mmol, 2 eq.), CuI (9.5 mg, 0.05 mmol, 10 mol%) and H_2O (80 µl, 4 mmol, 8 eq.) were dissolved in dry 1,4-dioxane (1 ml) and stirred for 16 h at 80 °C. The product purified by column chromatography (petroleum ether/EtOAc – 20/1).

Physical State: colourless liquid.

R_f Value: 0.22 (petroleum ether/EtOAc – 20/1).

^{1}H NMR (Avance 400 MHz, CD_2Cl_2) δ 8.61 – 8.47 (m, 1H), 7.83 – 7.75 (m, 2H), 7.63 – 7.56 (m, 2H), 7.47 – 7.42 (m, 1H), 7.25 – 7.16 (m, 4H), 7.16 – 7.07 (m, 4H), 6.65 – 6.53 (m, 2H) ppm.

^{13}C NMR (Avance 176 MHz, CD_2Cl_2) δ 156.9, 149.6, 139.4, 137.7, 137.3, 136.6, 130.6, 130.1, 129.3, 128.9, 128.6, 128.3, 127.4, 127.2, 125.6, 122.2, 120.2 ppm.

IR (ATR, in CD_2Cl_2) ν 3052 (w), 3007 (w), 1953 (w), 1888 (w), 1698 (w), 1583 (m), 1563 (m), 1491 (w), 1471 (w), 1461 (m), 1432 (m), 1390 (w), 1297 (w), 1240 (w), 1195 (w), 1179 (w), 1152 (w), 1073 (w), 1043 (w), 1028 (w) cm^{-1}.

HRMS (ESI, m/z) calcd. for $C_{19}H_{15}N+H^+$: 258.1277, found: 258.1274.

^{1}H- and ^{13}C-NMR spectra of R3

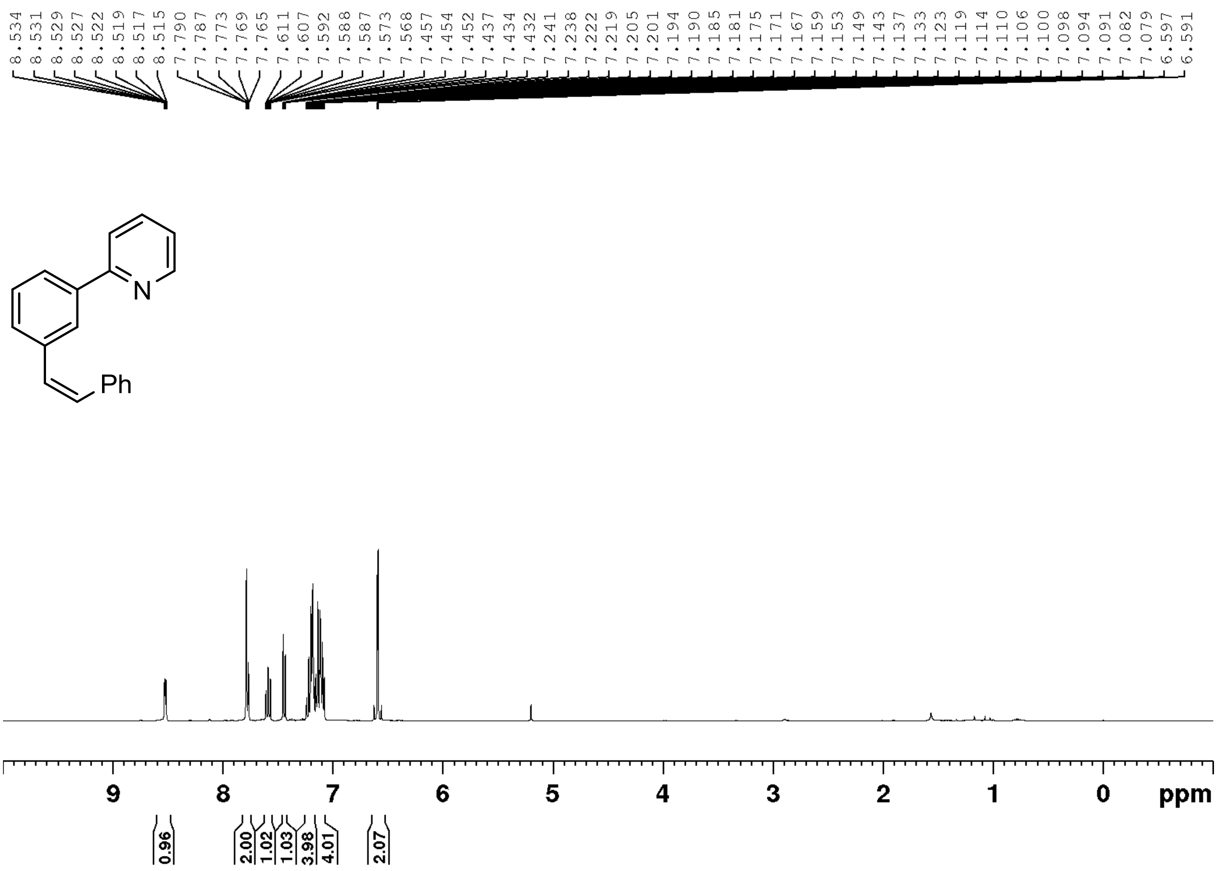

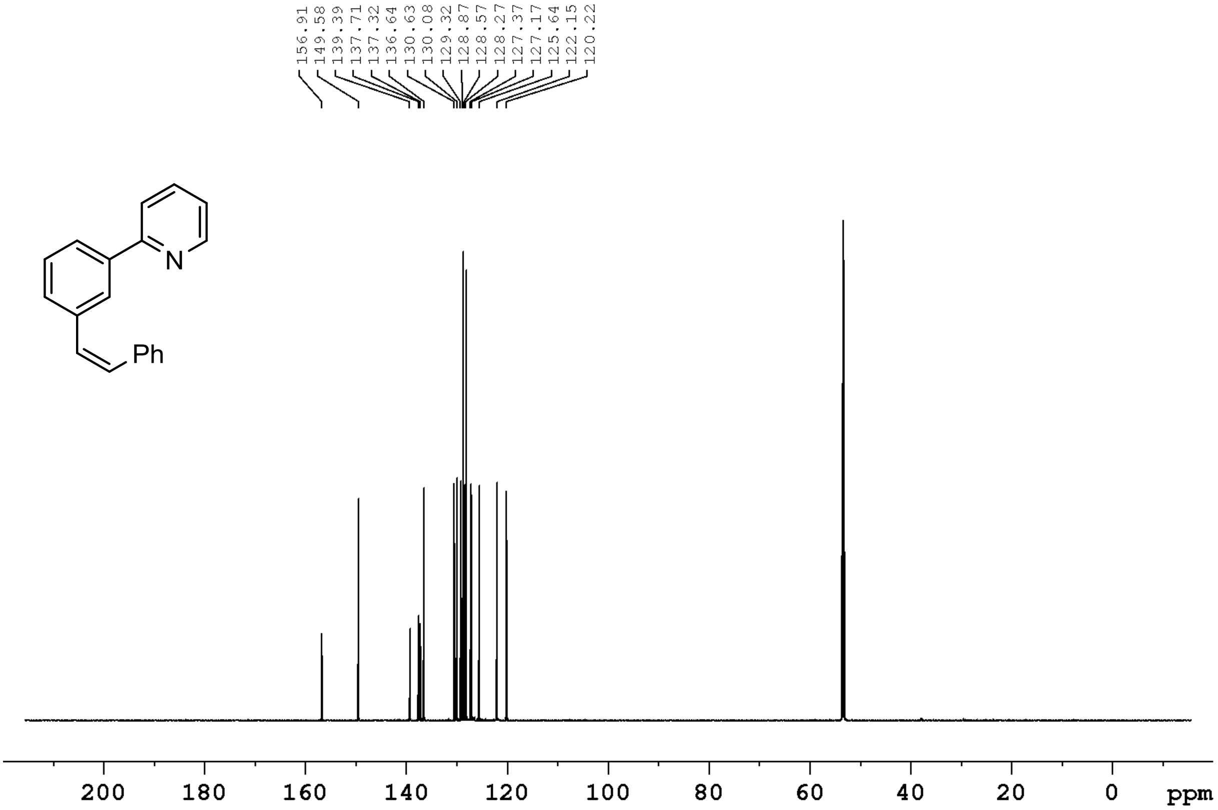

(*E*)-2-(3-styrylphenyl)pyridine R4

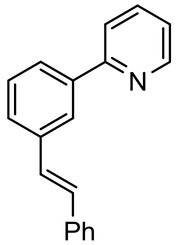

According to **GP-I**: **S3** (127.7 mg, 0.5 mmol, 1 eq.), $RuCl_2(PPh_3)_3$ (12.0 mg, 0.0125 mmol, 2.5 mol%), Zn (65.4 mg, 1 mmol, 2 eq.), CuI (9.5 mg, 0.05 mmol, 10 mol%) and H_2O (80 µl, 4 mmol, 8 eq.) were dissolved in dry 1,4-dioxane (1 ml) and stirred for 16 h at 80 °C. The product purified by column chromatography (petroleum ether/EtOAc – 20/1).

Physical State: colourless solid.

R_f Value: 0.17 (petroleum ether/EtOAc – 20/1).

^{1}H NMR (Avance 400 MHz, CD_2Cl_2) δ 8.73 – 8.67 (m, 1H), 8.23 (t, *J* = 1.6 Hz, 1H), 7.91 (dt, *J* = 7.8, 1.16 Hz, 1H), 7.82 – 7.74 (m, 2H), 7.62 – 7.54 (m, 3H), 7.50 – 7.45 (m, 1H), 7.41 – 7.35 (m, 2H), 7.32 – 7.22 (m, 4H) ppm.

^{13}C NMR (Avance 126 MHz, CD_2Cl_2) δ 157.3, 150.0, 140.2, 138.2, 137.7, 137.1, 129.4, 129.4, 129.1, 128.8, 128.1, 127.3, 126.9, 126.5, 125.4, 122.7, 120.8 ppm.

IR (ATR, in CD_2Cl_2) ν 3056 (w), 3024 (w), 1949 (w), 1878 (w), 1806 (w), 1584 (m), 1565 (w), 1493 (w), 1461 (m), 1446 (w), 1433 (w), 1413 (w), 1300 (w), 1274 (w), 1247 (w), 1214 (w), 1180 (w), 1153 (w), 1073 (w), 1044 (w), 1028 (w) cm^{-1}.

HRMS (ESI, m/z) calcd. for $C_{19}H_{15}N+H^+$: 258.1277, found: 258.1274.

^{1}H- and ^{13}C-NMR spectra of R4

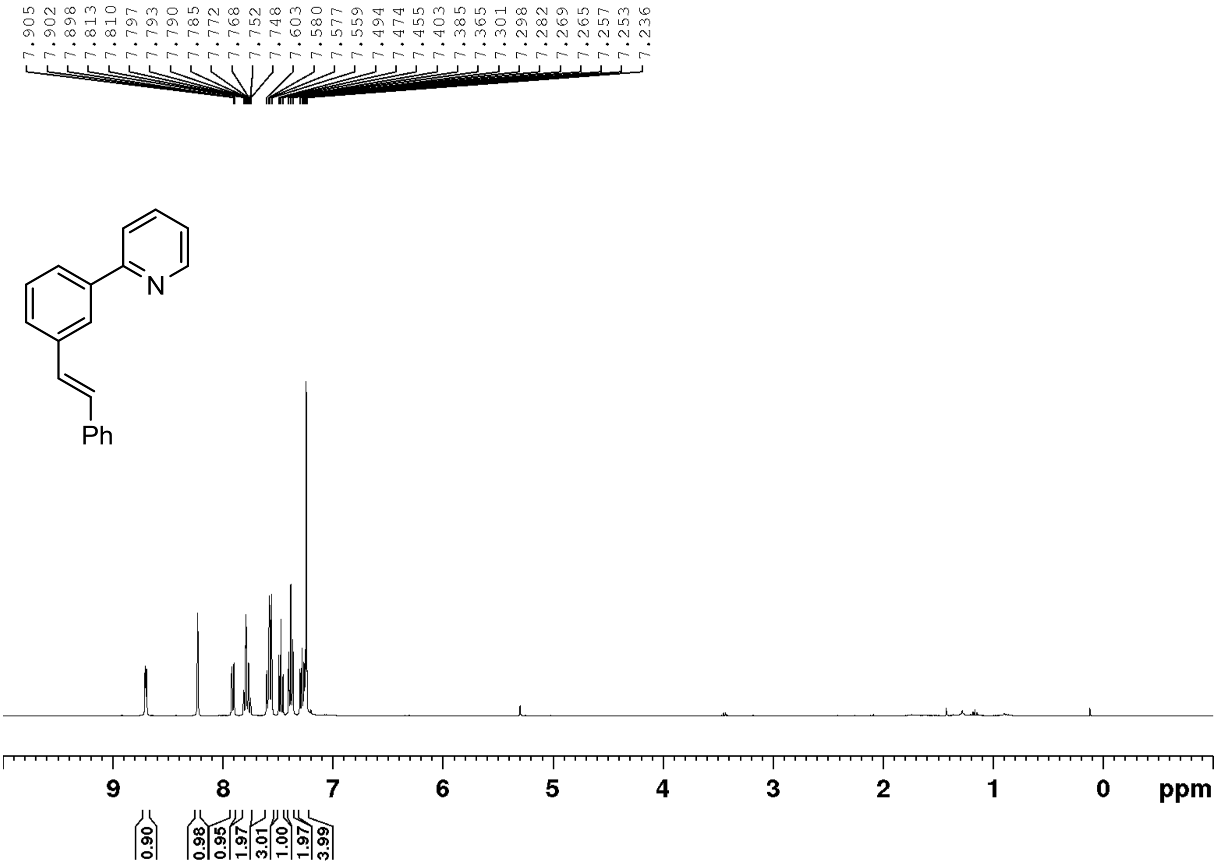

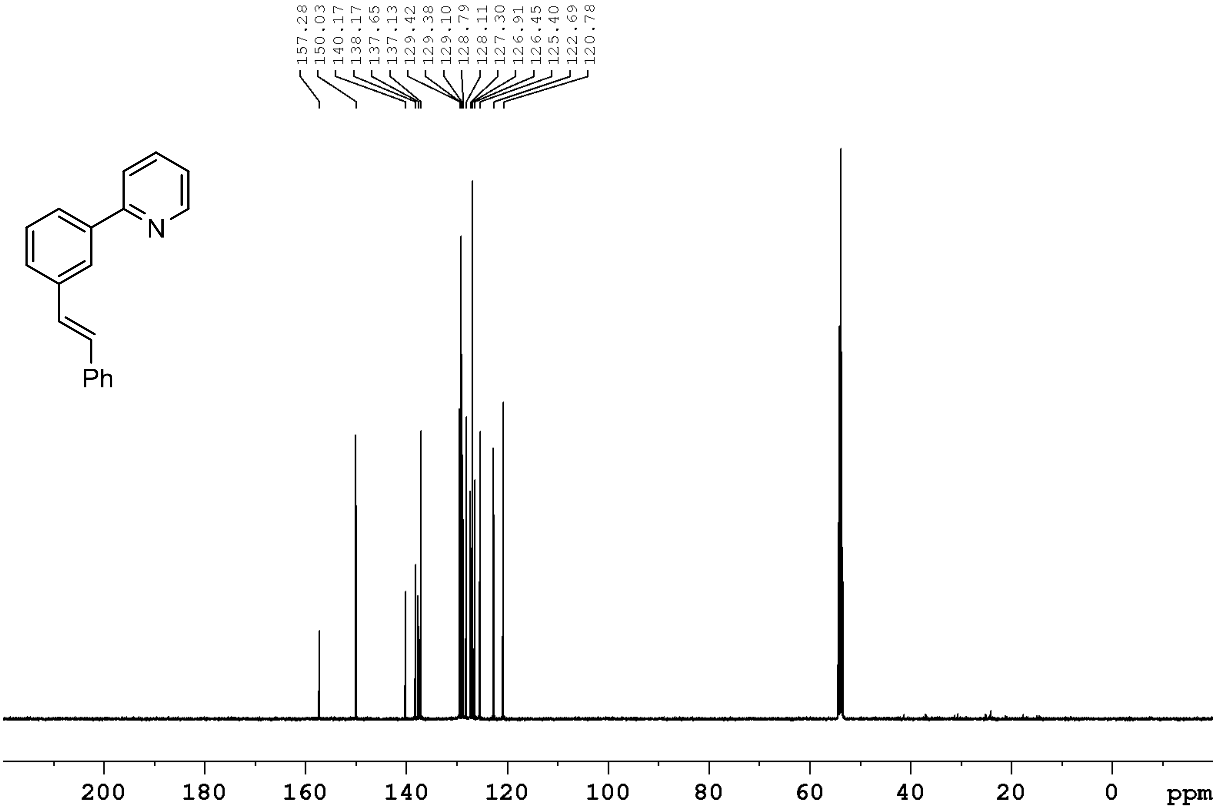

N-(*n*-propyl)benzamide R5

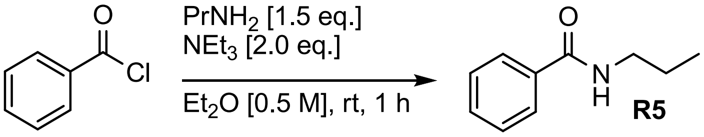

N-(*n*-propyl)benzamide R5

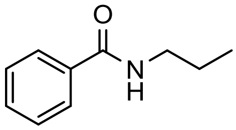

n-Propylamine (0.62 ml, 7.5 mmol, 1.5 eq.), benzoyl chloride (0.58 ml, 5 mmol, 1.0 eq.) and NEt_3 (1.4 ml, 10 mmol, 2 eq.) were dissolved in dry Et_2O (40 ml). The reaction mixture was stirred at room temperature for 1 h. The mixture was extracted with Et_2O and purified by flash column chromatography (petroleum ether/EtOAc – 5/1 → 2/1).

Yield: 0.764 g (4.68 mmol, 94%).

Physical State: colourless solid.

R_f Value: 0.20 (petroleum ether/EtOAc – 5/1).

^{1}H NMR (Avance 400 MHz, $CDCl_3$) δ 7.76 (d, J = 7.96 Hz, 2H), 7.55 – 7.31 (m, 3H), 6.45 - 6.04 (m, 1H), 3.52 – 3.27 (m, 2H), 1.73 – 1.54 (m, 2H), 1.04 – 0.90 (m, 3H) ppm.

^{13}C NMR (Avance 101 MHz, $CDCl_3$) δ 167.6, 134.9, 131.3, 128.5, 126.8, 41.8, 22.9, 11.4 ppm.

IR (ATR, in $CDCl_3$) ν 3302 (m), 3084 (w), 2965 (w), 2933 (w), 2872 (w), 1632 (s), 1603 (w), 1577 (m), 1547 (s), 1493 (m), 1465 (w), 1449 (w), 1433 (w), 1374 (w), 1327 (m), 1315 (m), 1291 (m), 1246 (w), 1151 (w) cm^{-1}.

MS (ESI): *m/z* (%): for $C_{10}H_{13}NO+Na^+$ 186.1 (87), 164.1 (100), 122.1 (9), 105.0 (14).

^{1}H- and ^{13}C-NMR spectra of R5

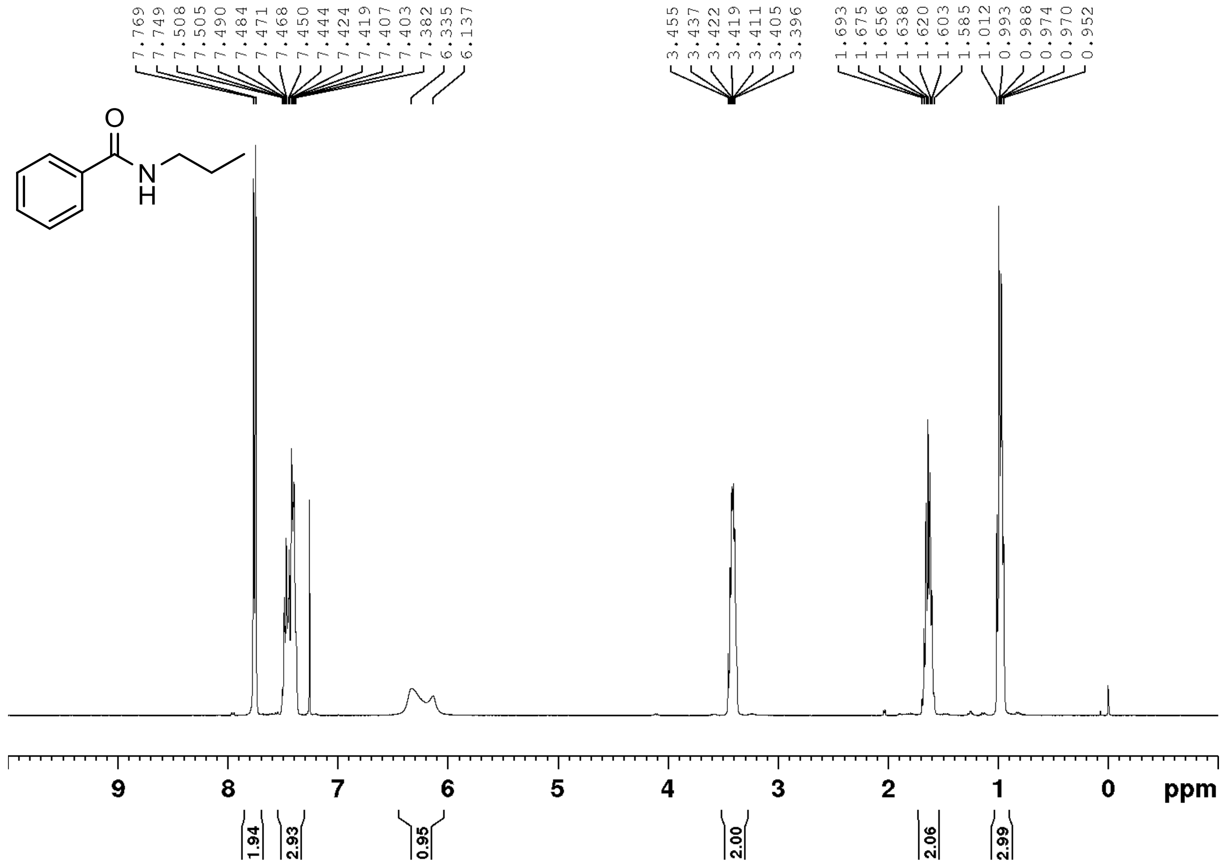
7.769
7.749
7.508
7.505
7.490
7.484
7.471
7.468
7.450
7.444
7.424
7.419
7.407
7.403
7.382
6.335
6.137
3.455
3.437
3.422
3.419
3.411
3.405
3.396
1.693
1.675
1.656
1.638
1.620
1.603
1.585
1.012
0.993
0.988
0.974
0.970
0.952
O
N
H
9
8
7
6
5
4
3
2
1
0
ppm
1.94
2.93
0.95
2.00
2.06
2.99

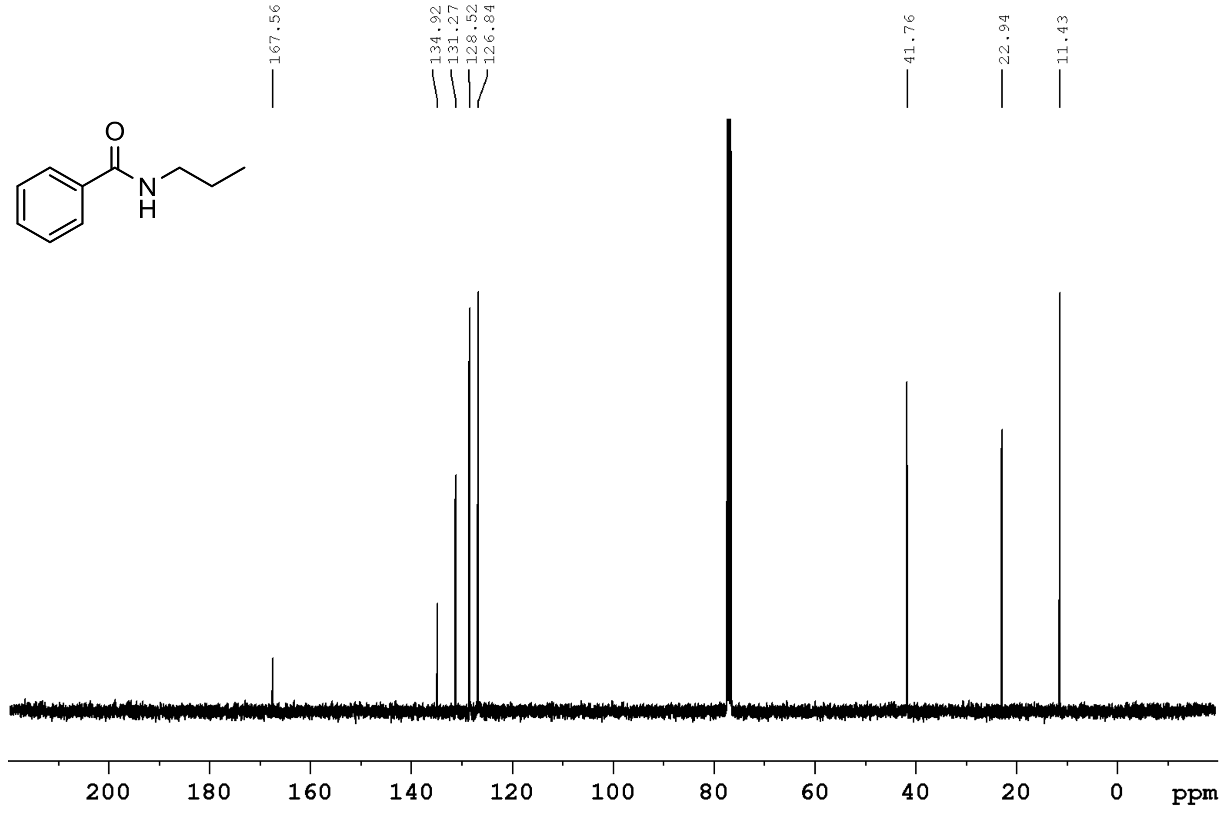
167.56
134.92
131.27
128.52
126.84
41.76
22.94
11.43
O
N
H
200
180
160
140
120
100
80
60
40
20
0
ppm

3. Ru-catalyzed C-H-deuteration

3.1 General procedure for the deuteration following the CuI procedure (condition A) (GP-I)

To a flame-dried schlenk tube was added $RuCl_2(PPh_3)_3$ (2.5 mol%), CuI (10 mol%) and Zinc (2 eq.) under nitrogen stream. After the addition of 1,4-dioxane (1 ml) the substrate (0.5 mmol) was added. D_2O (8 eq.) was added and the reaction mixture was stirred for the time given at 80 °C. For work-up the mixture was filtered over silica and ethyl acetate was used as eluant. The solvent was evaporated under reduced pressure and crude NMRs were measured with mesitylene (0.5 mmol) as internal standard.

3.2 General procedure for the deuteration following the KOD/Zn procedure (condition B) (GP-II)

To a flame-dried schlenk tube was added $RuCl_2(PPh_3)_3$ (2.5 mol%) and Zinc (2 eq.) under nitrogen stream. After the addition of 1,4-dioxane (1 ml) the substrate (0.5 mmol) was added. KOD in D_2O (25 mol%, 40 wt% in D_2O, 98 atom % D) and D_2O (8 eq.) were added. The reaction mixture was stirred for the time given at 80 °C. For work-up the mixture was filtered over silica and ethyl acetate was used as eluant. The solvent was evaporated under reduced pressure and crude NMRs were measured with mesitylene (0.5 mmol) as internal standard.

3.3 General procedure for the deuteration following the KOD procedure (condition C) (GP-III)

To a flame-dried schlenk tube was added $RuCl_2(PPh_3)_3$ (2.5 mol%) under nitrogen stream. After the addition of 1,4-dioxane (1 ml) the substrate (0.5 mmol) was added. KOD in D_2O (25 mol%, 40 wt% in D_2O, 98 atom % D) and D_2O (8 eq.) were added and the reaction mixture was stirred for the time given at 80 °C. For work-up the mixture was filtered over silica and ethyl acetate was used as eluant. The solvent was evaporated under reduced pressure and crude NMRs were measured with mesitylene (0.5 mmol) as internal standard.

3.4 General remarks on overview tables

All experiments performed on each substrate are condensed into a table displayed above the spectra. The following abbreviations were used:

- for no experimental data

n.o. for not observed.

n.d. for not determinable

3.5. Spectral data and overviews

3.5.1 Tolan 5

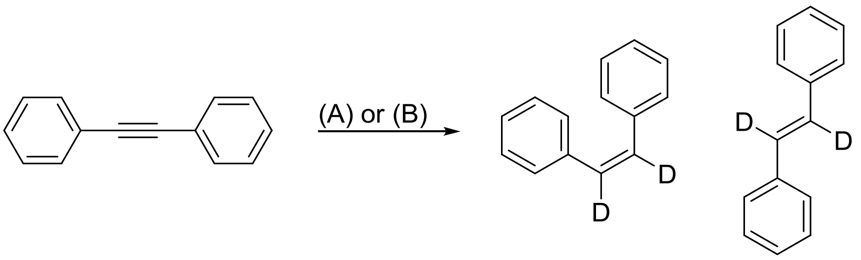

I II

		I (*Z*)		II (*E*)	
	t	D_A	Yield	D_A	Yield
cond. (A)	16 h	80%	80%	70%	13%
	62 h	n.o.	n.o.	80%	98%
cond. (B)	16 h	-	-	-	-
	62 h	-	-	-	-

(*Z*/*E*)-Stilben 8 (I and II)

^{1}H-NMR (400 MHz, CD_2Cl_2) Spectra of pure compound:

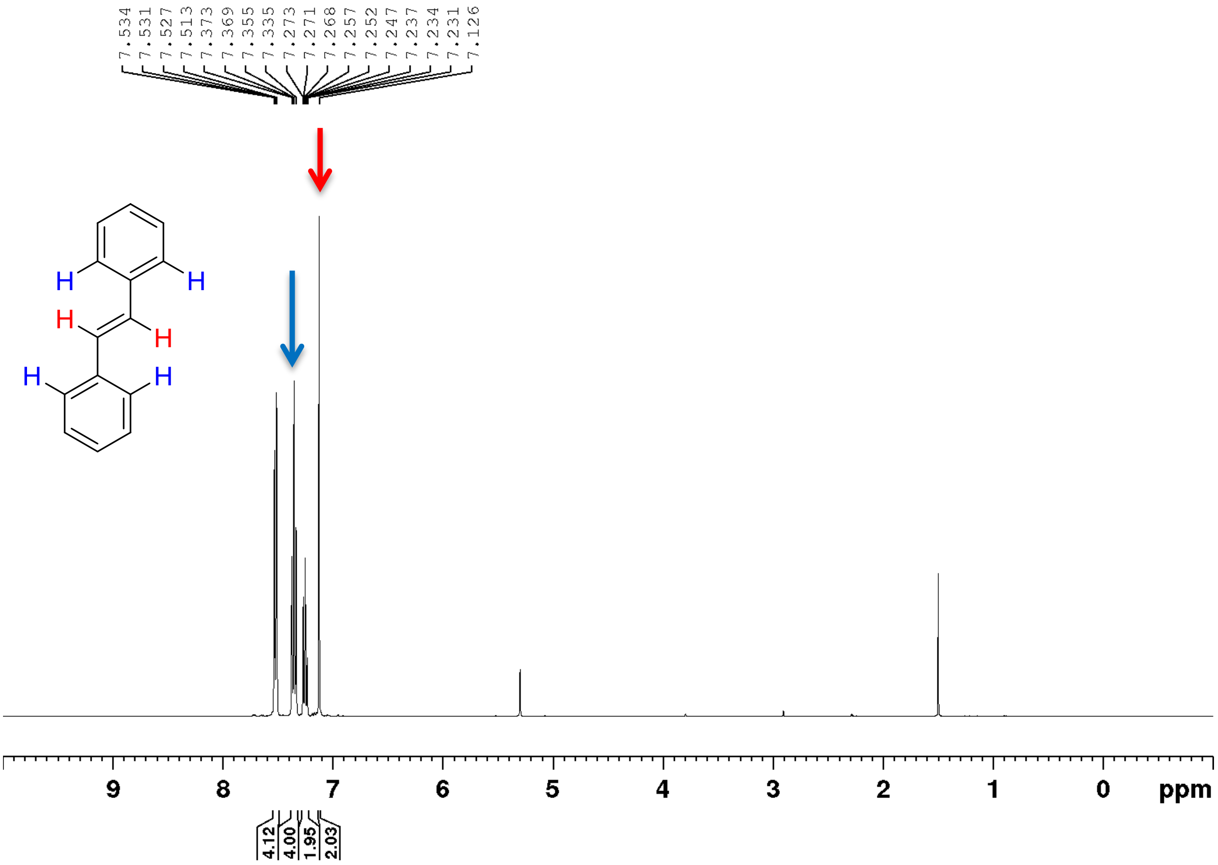

Enlargement of relevant area:

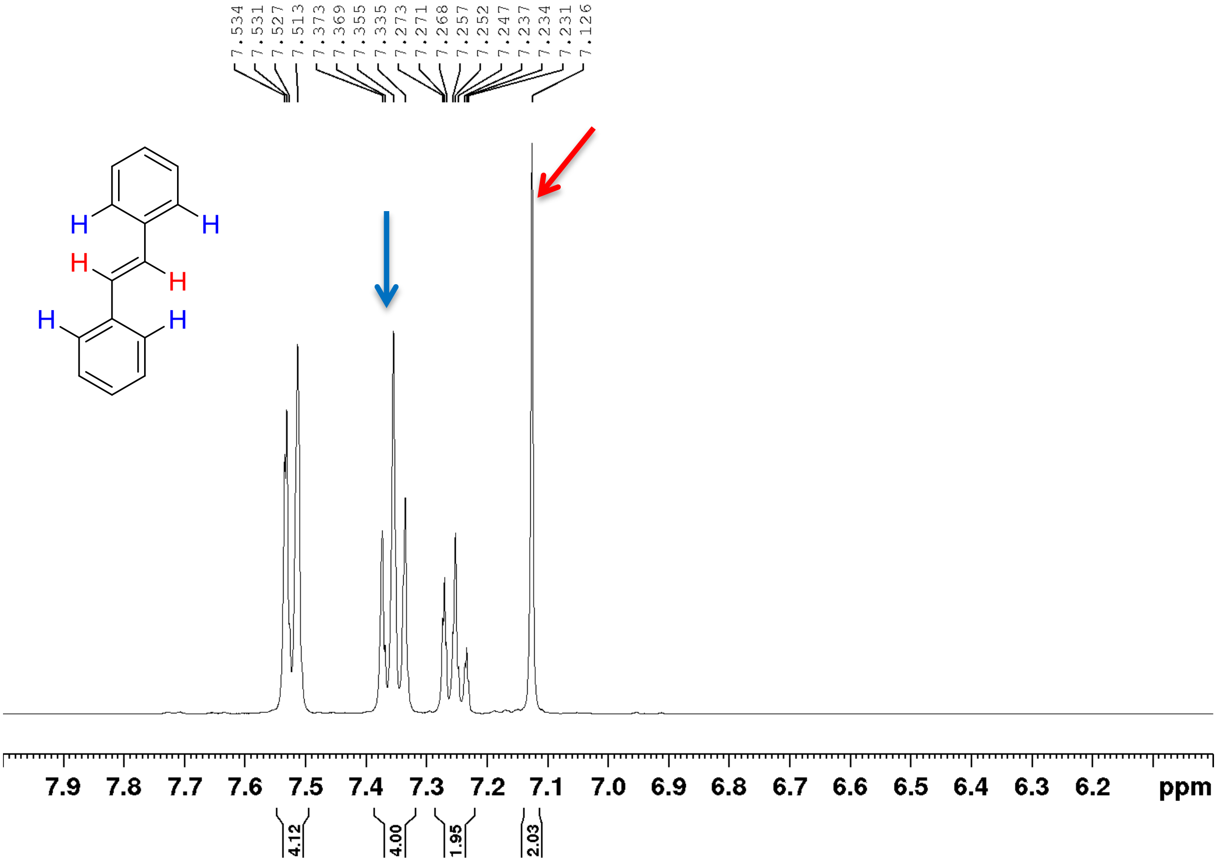

^{1}H-NMR (400 MHz, CD_2Cl_2, mesitylene) Spectra of deuterated compound **8** following the CuI procedure for 16 h: (*Z*/*E*: 6:1)

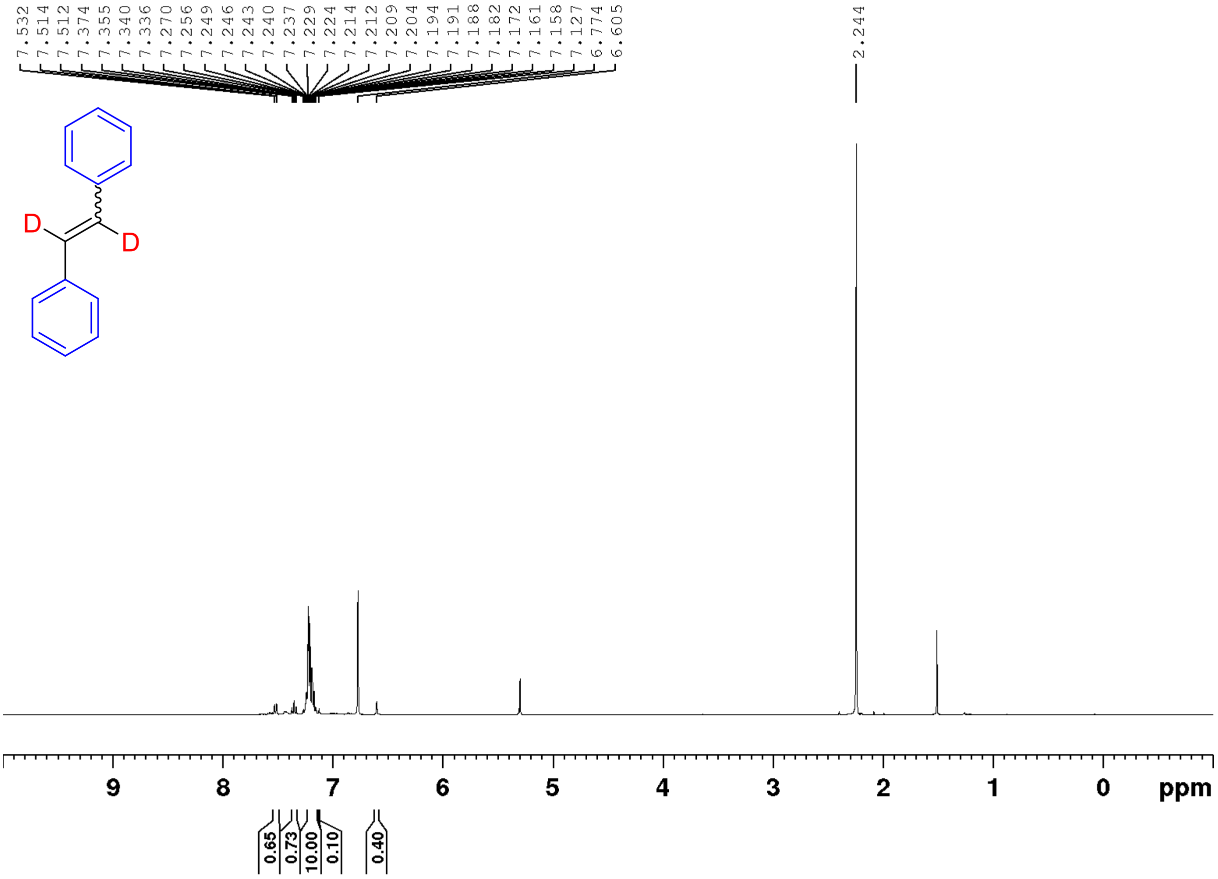

Enlargement of relevant area:

Yield of *Z*-Isomer[8]: 80%

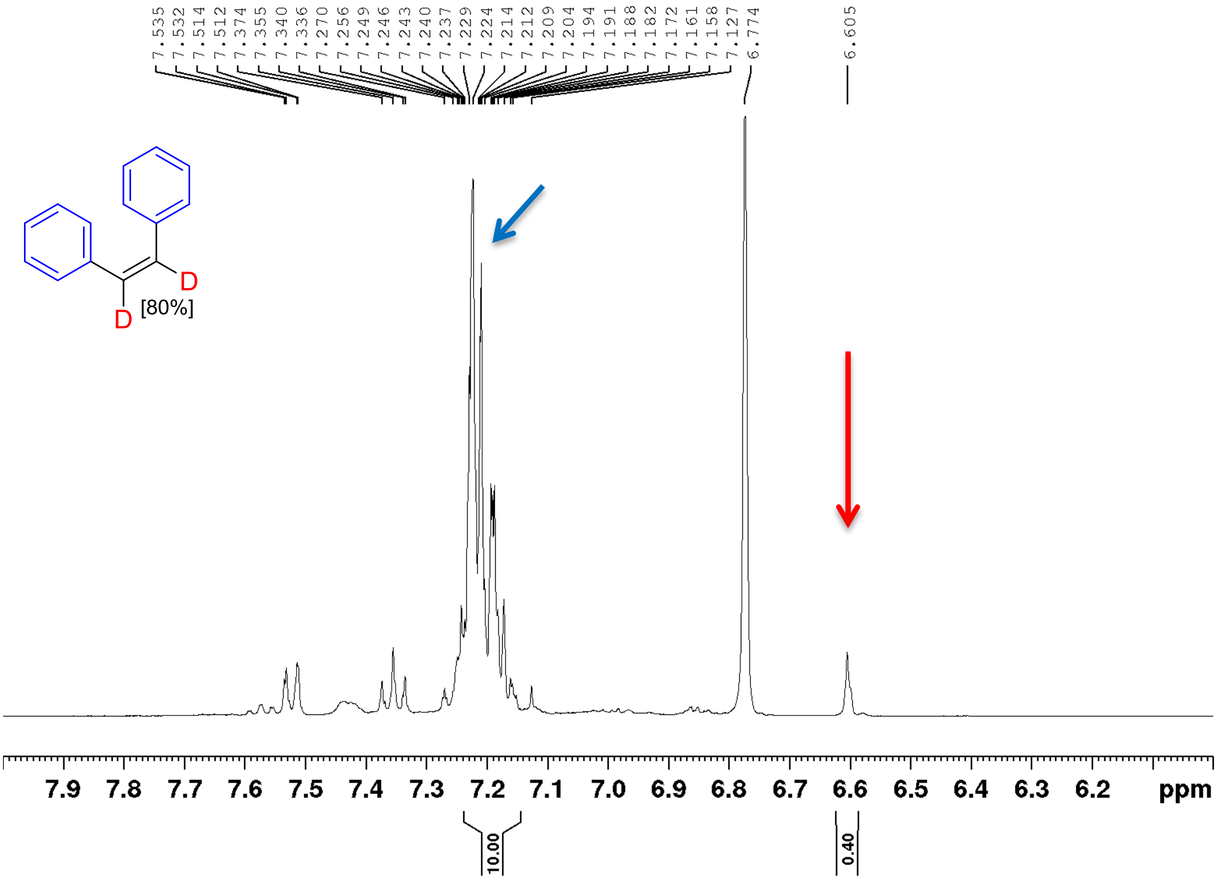

Yield of *E*-Isomer: 13%

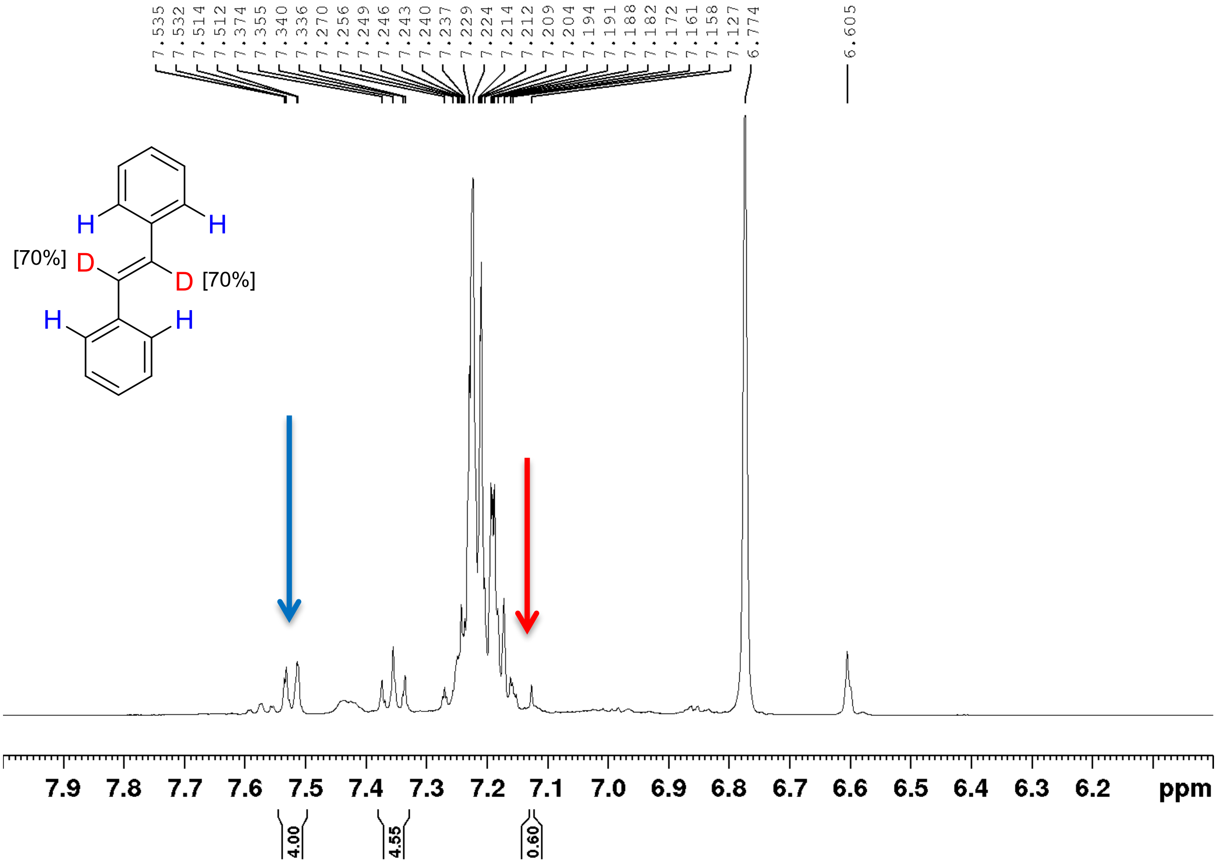

^{1}H-NMR (400 MHz, CD_2Cl_2, mesitylene) Spectra of deuterated compound **8** following the CuI procedure for 62 h: Yield: 98%

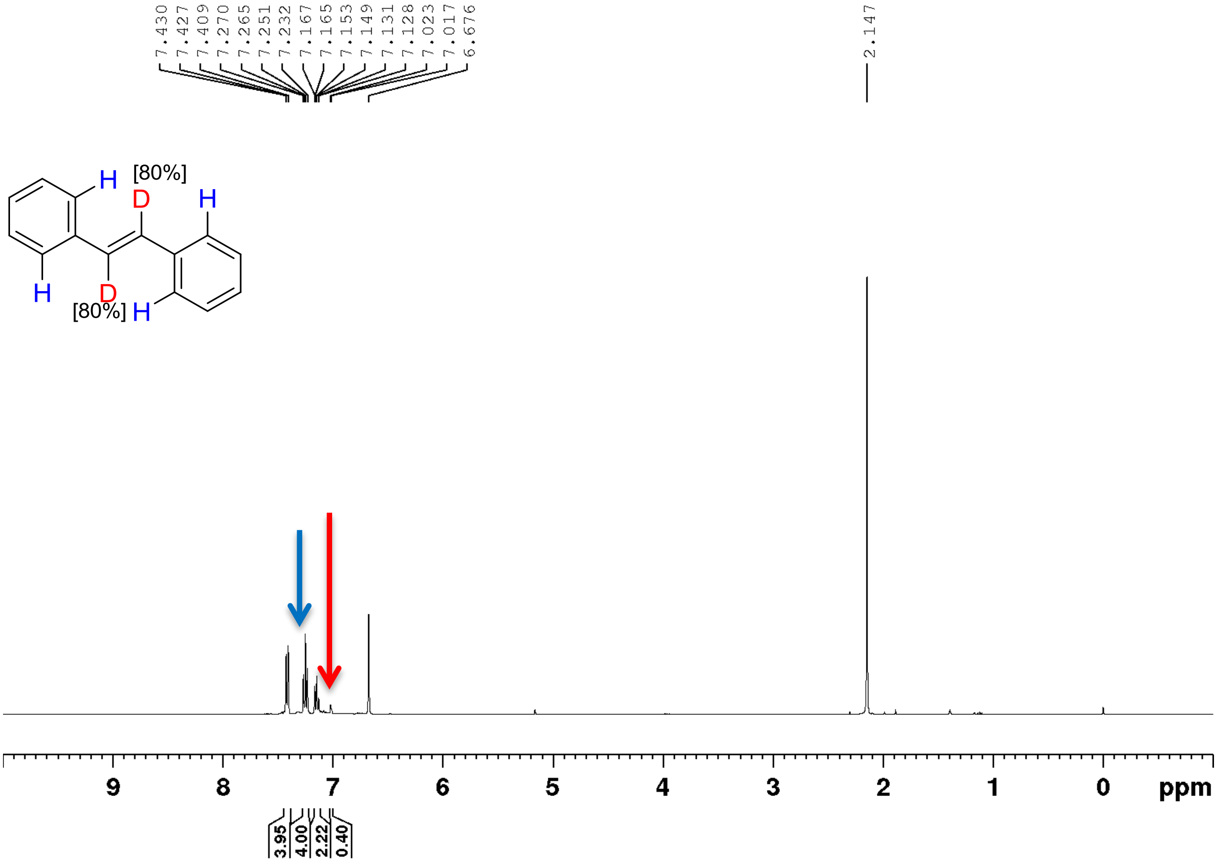

Enlargement of relevant area:

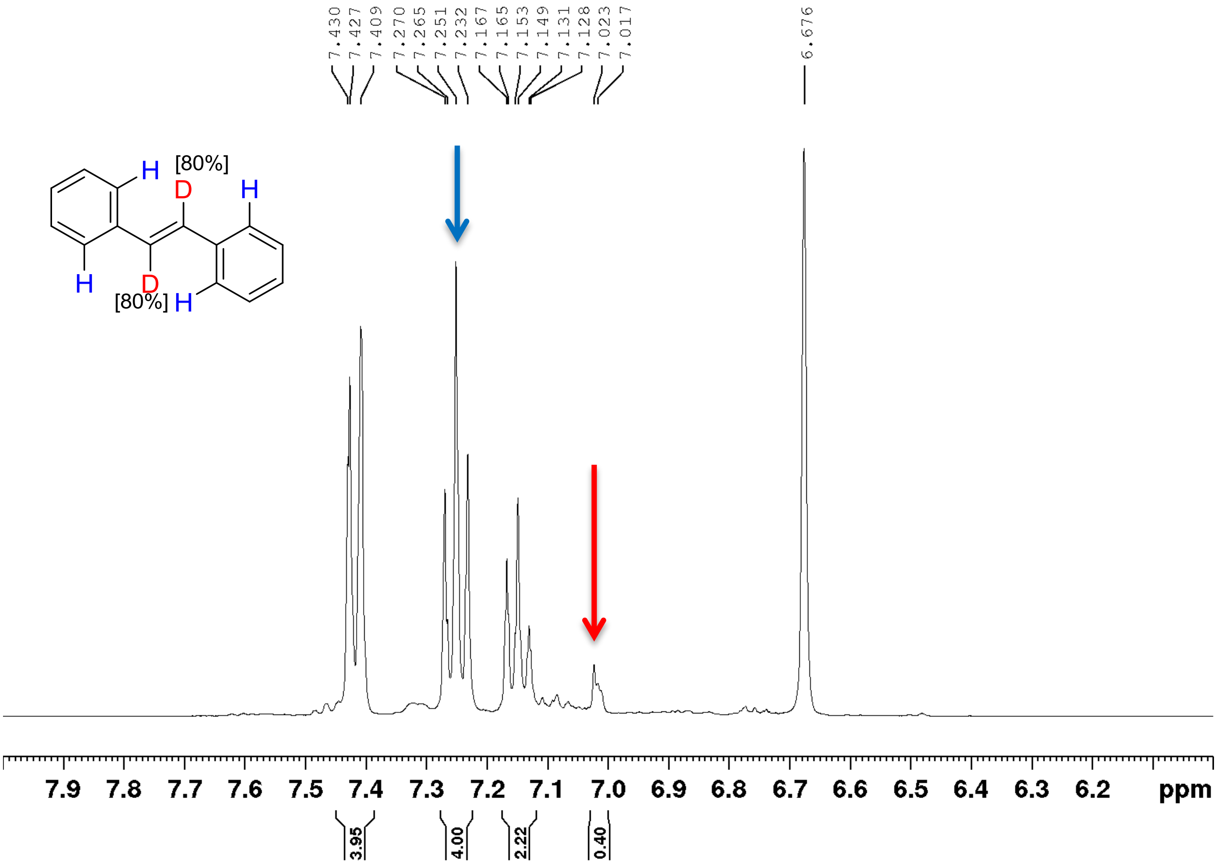

3.5.2 4-(phenylethynyl)toluene 6

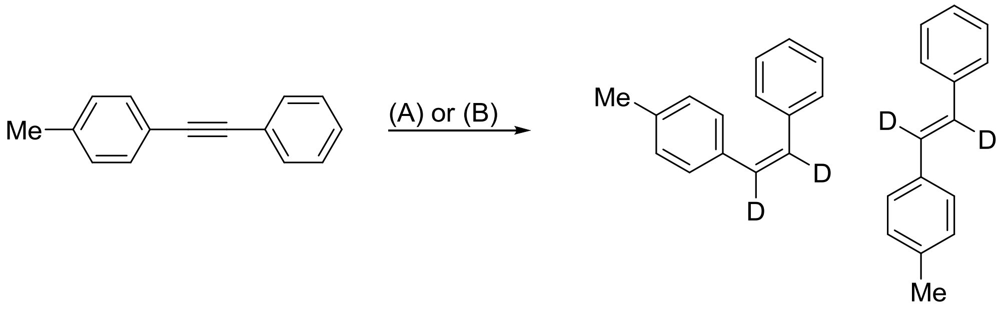

I **II**

		I (*Z*)		II (*E*)	
	t	D_A	Yield	D_A	Yield
cond. (A)	16 h	85%	86%	79%	7%
	62 h	n.o.	n.o.	77%	99%
cond. (B)	16 h	-	-	-	-
	62 h	-	-	-	-

(Z/*E*)-4-Methylstilbene 8 (I and II)

^{1}H-NMR (400 MHz, CD_2Cl_2) Spectra of pure compound **8**:

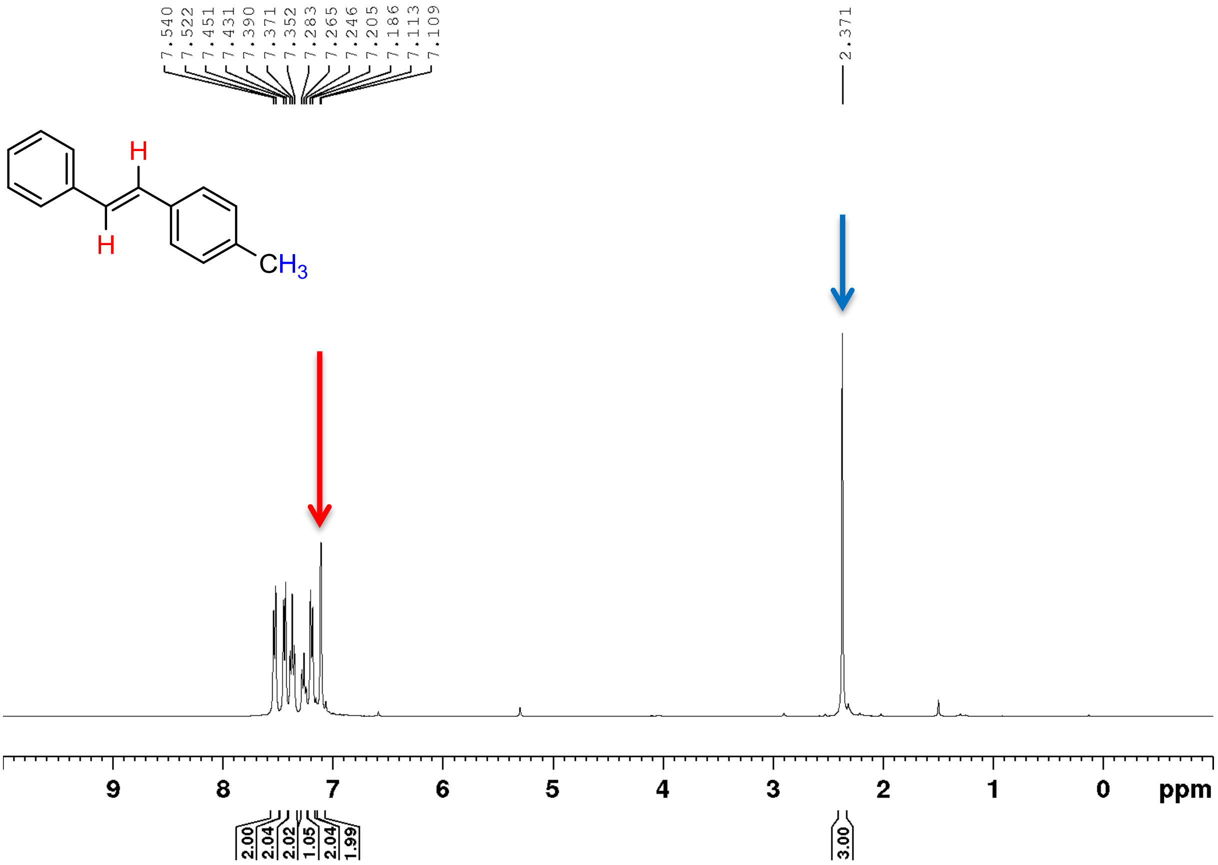

Enlargement of relevant area:

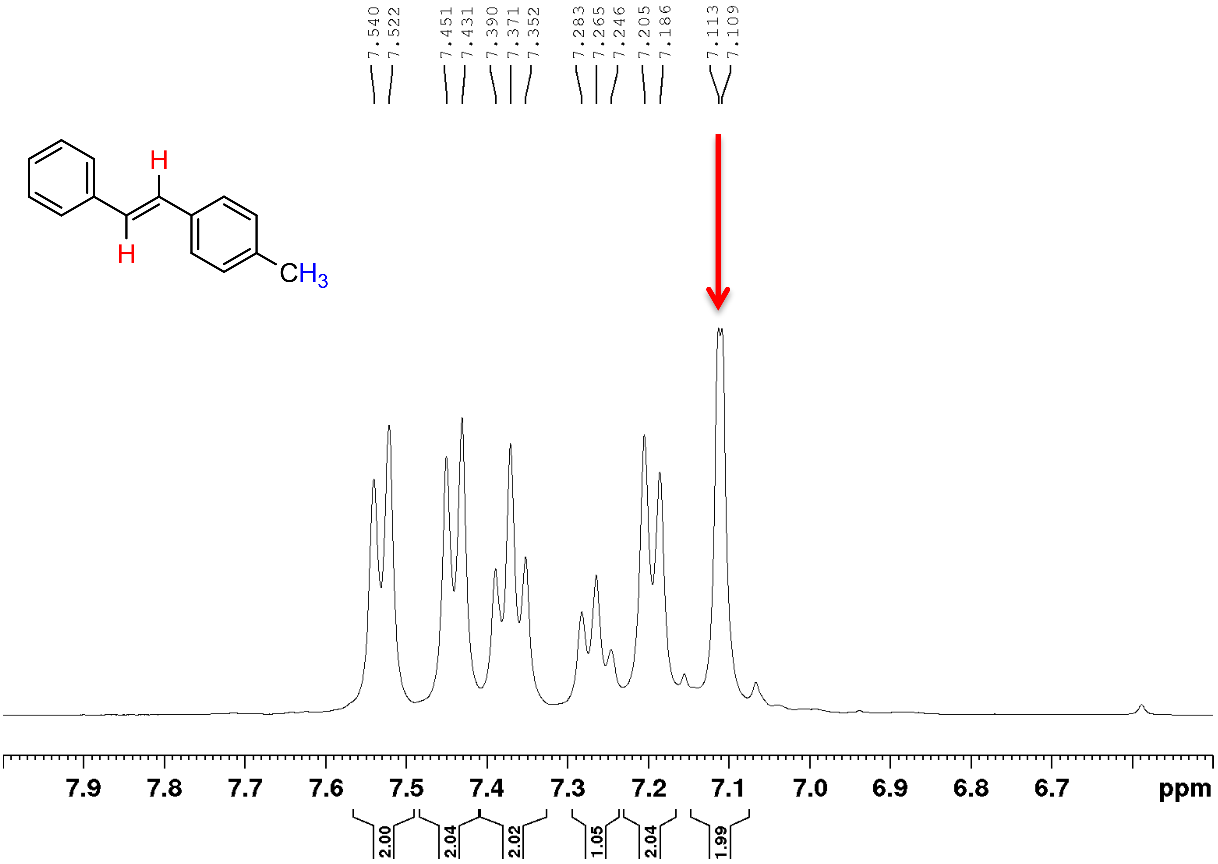

^{1}H-NMR (400 MHz, CD_2Cl_2, mesitylene) Spectra of crude deuterated mixture following the CuI procedure for 16 h: (*Z*/*E*: 12:1)

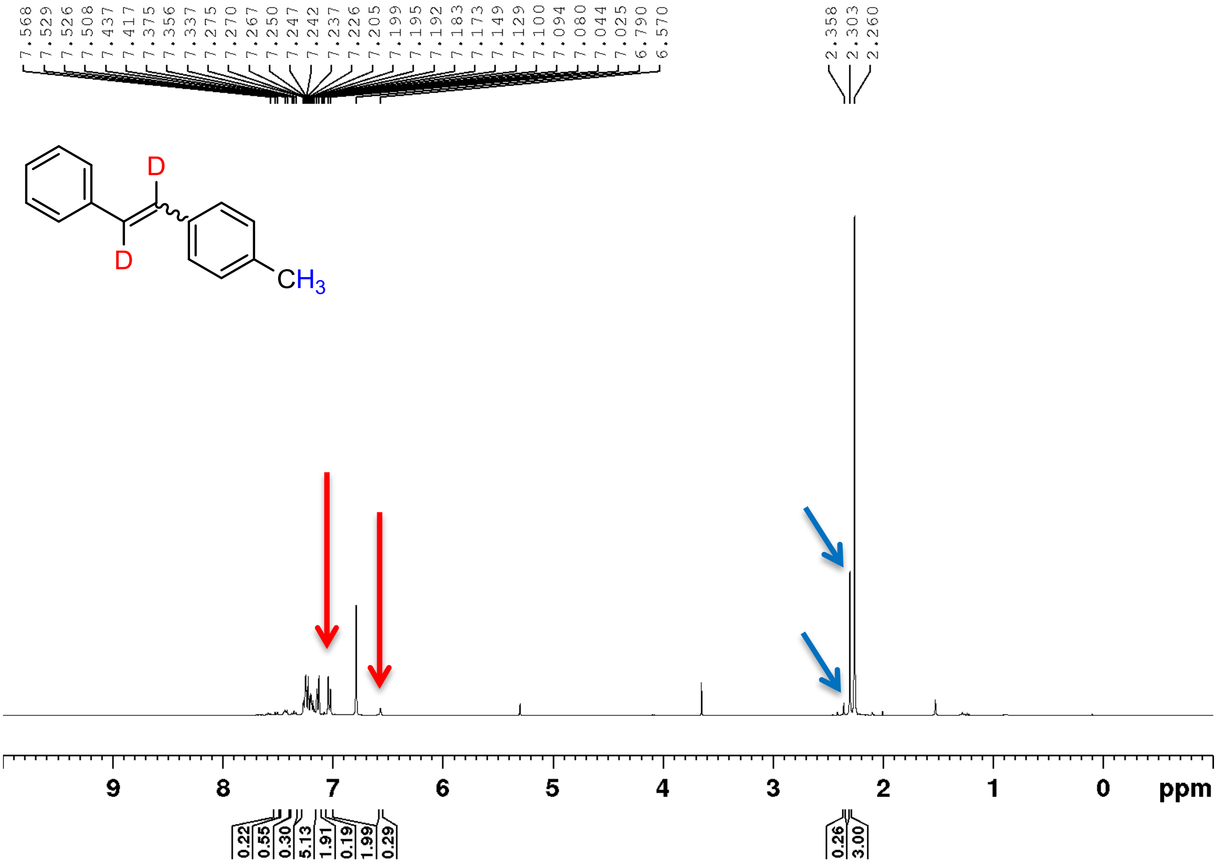

Enlargement of relevant area:

Yield of *Z*-Isomer[9]: 86%

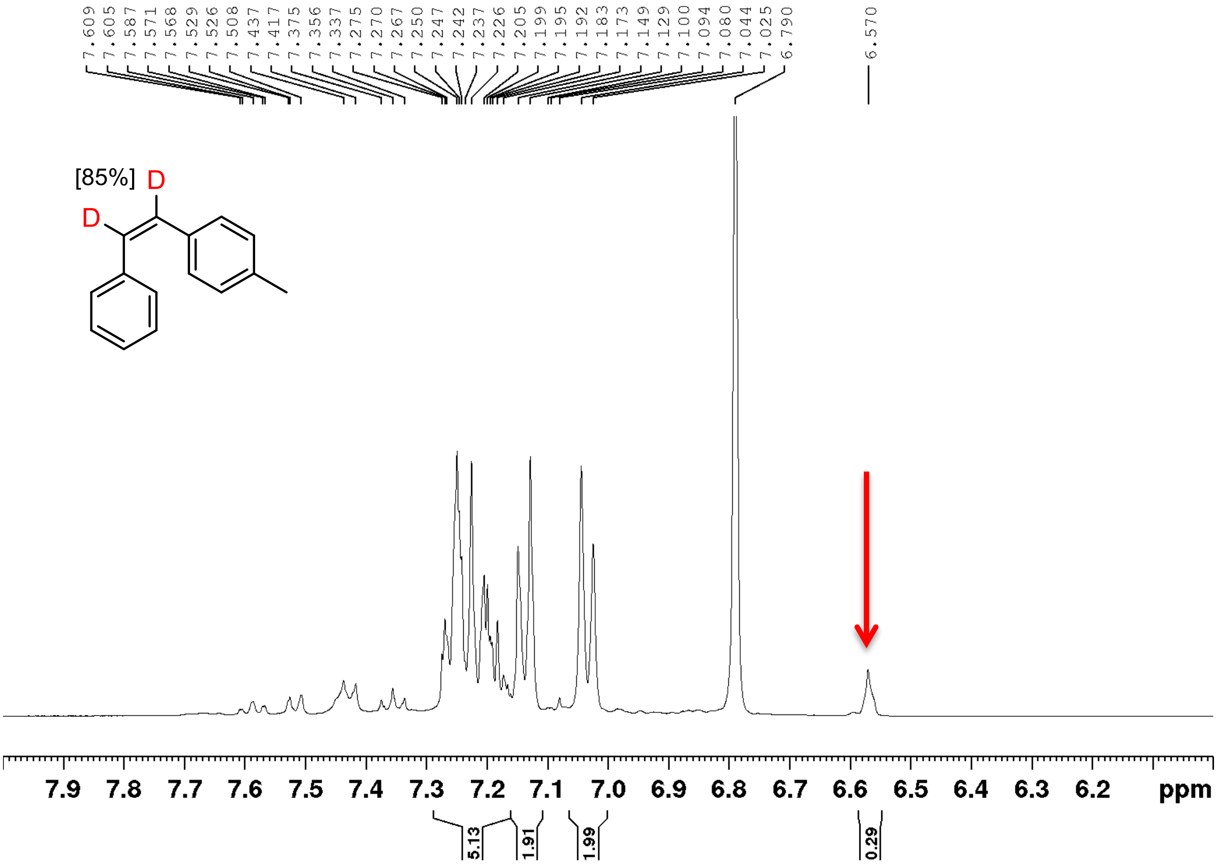

^{1}H-NMR (400 MHz, $CDCl_3$, mesitylene) after columnchromatographie:

Yield of *E*-Isomer before columnchromatographie: 7%

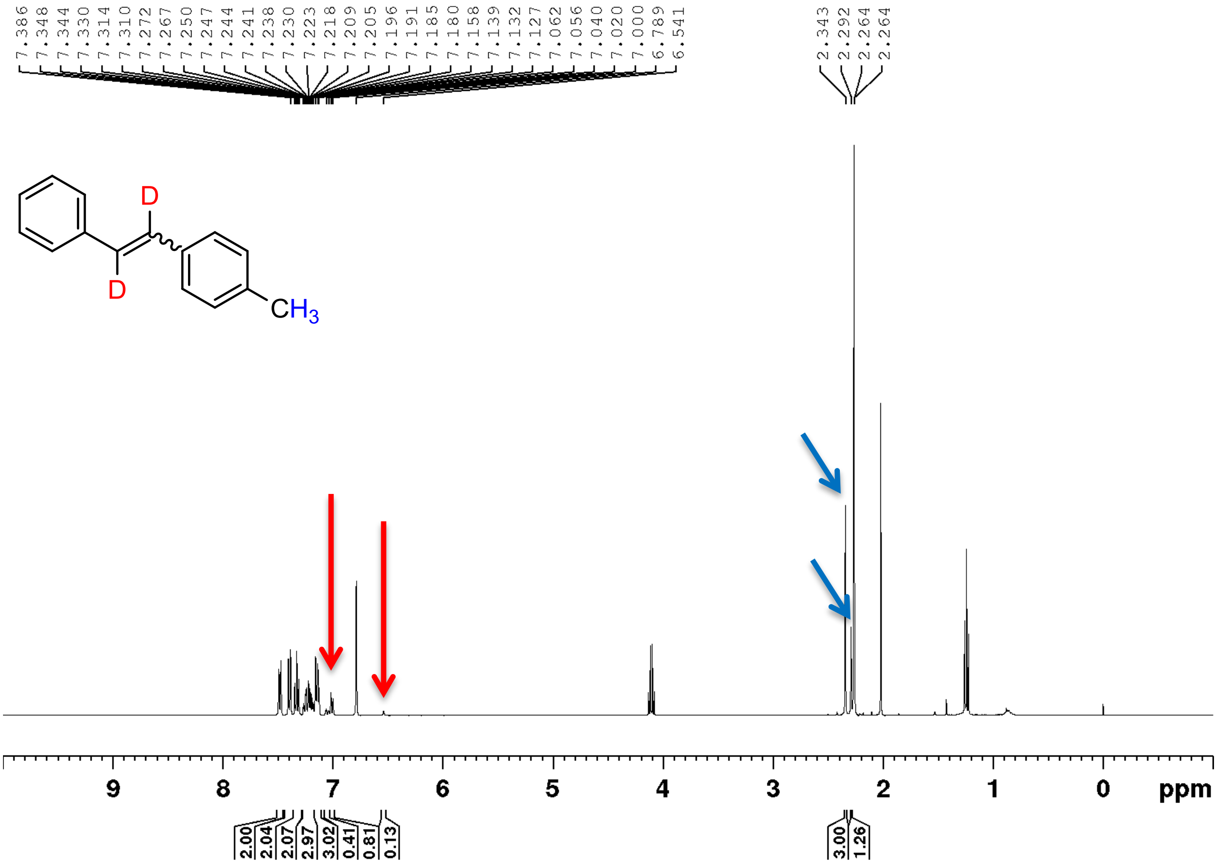

7.386 7.348 7.344 7.330 7.314 7.310 7.272 7.267 7.250 7.247 7.244 7.241 7.238 7.230 7.223 7.218 7.209 7.205 7.196 7.191 7.185 7.180 7.158 7.139 7.132 7.127 7.062 7.056 7.040 7.020 7.000 6.789 6.541
2.343 2.292 2.264 2.264
D
D
CH3
9 8 7 6 5 4 3 2 1 0 ppm
2.00 2.04 2.07 2.97 3.02 0.41 0.81 0.13
3.00 1.26

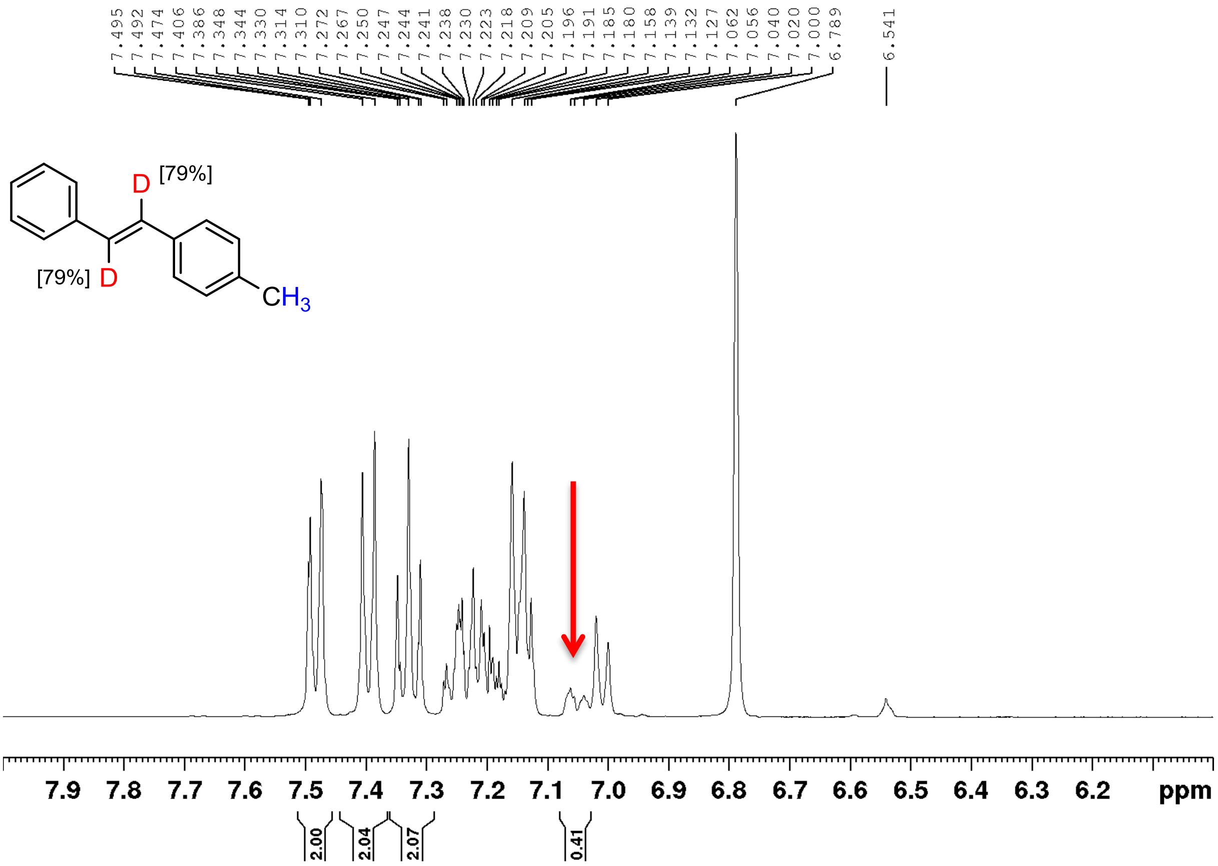

7.495 7.492 7.474 7.406 7.386 7.348 7.344 7.330 7.314 7.310 7.272 7.267 7.250 7.247 7.244 7.241 7.238 7.230 7.223 7.218 7.209 7.205 7.196 7.191 7.185 7.180 7.158 7.139 7.132 7.127 7.062 7.056 7.040 7.020 7.000 6.789
6.541
D [79%]
[79%] D
CH3
7.9 7.8 7.7 7.6 7.5 7.4 7.3 7.2 7.1 7.0 6.9 6.8 6.7 6.6 6.5 6.4 6.3 6.2 ppm
2.00 2.04 2.07 0.41

^{1}H-NMR (400 MHz, CD_2Cl_2, mesitylene) Spectra of deuterated compound following the CuI procedure for 62 h: Yield: 99%

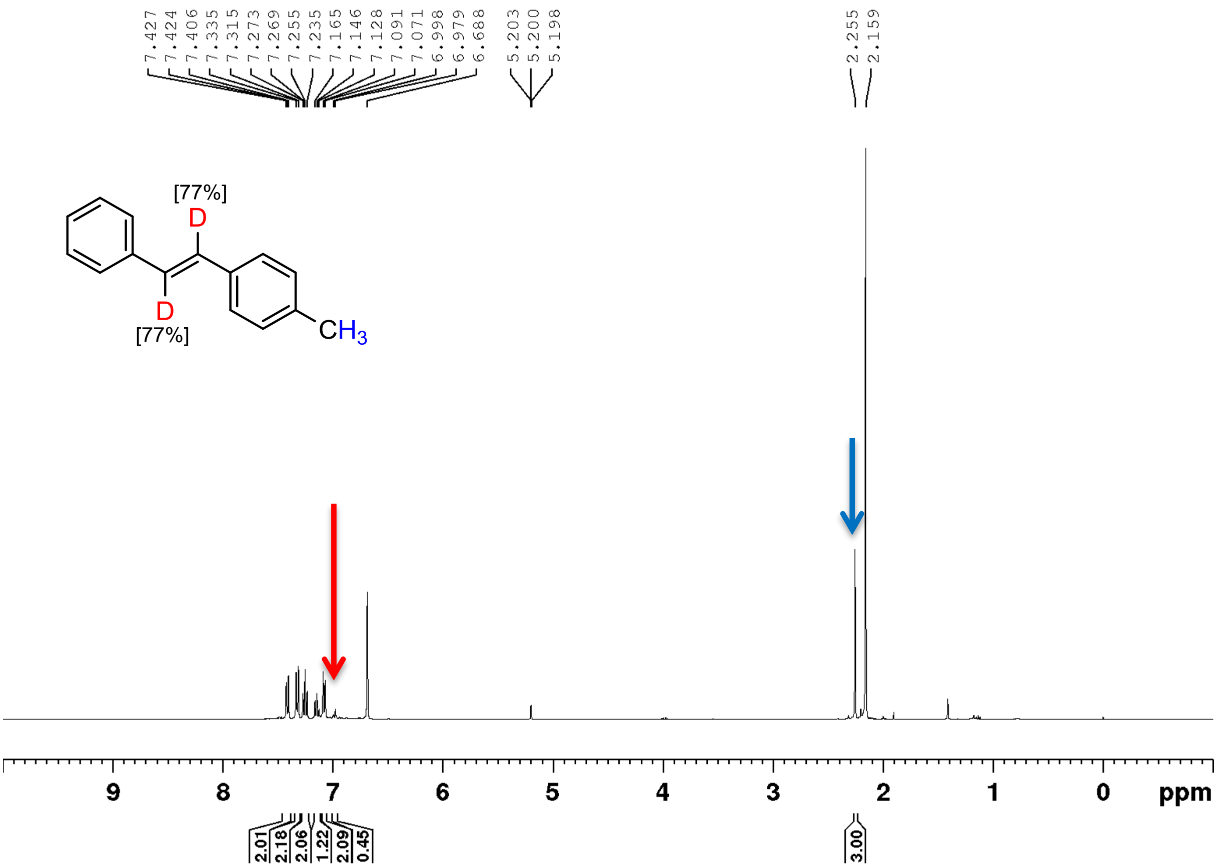

Enlargement of relevant area:

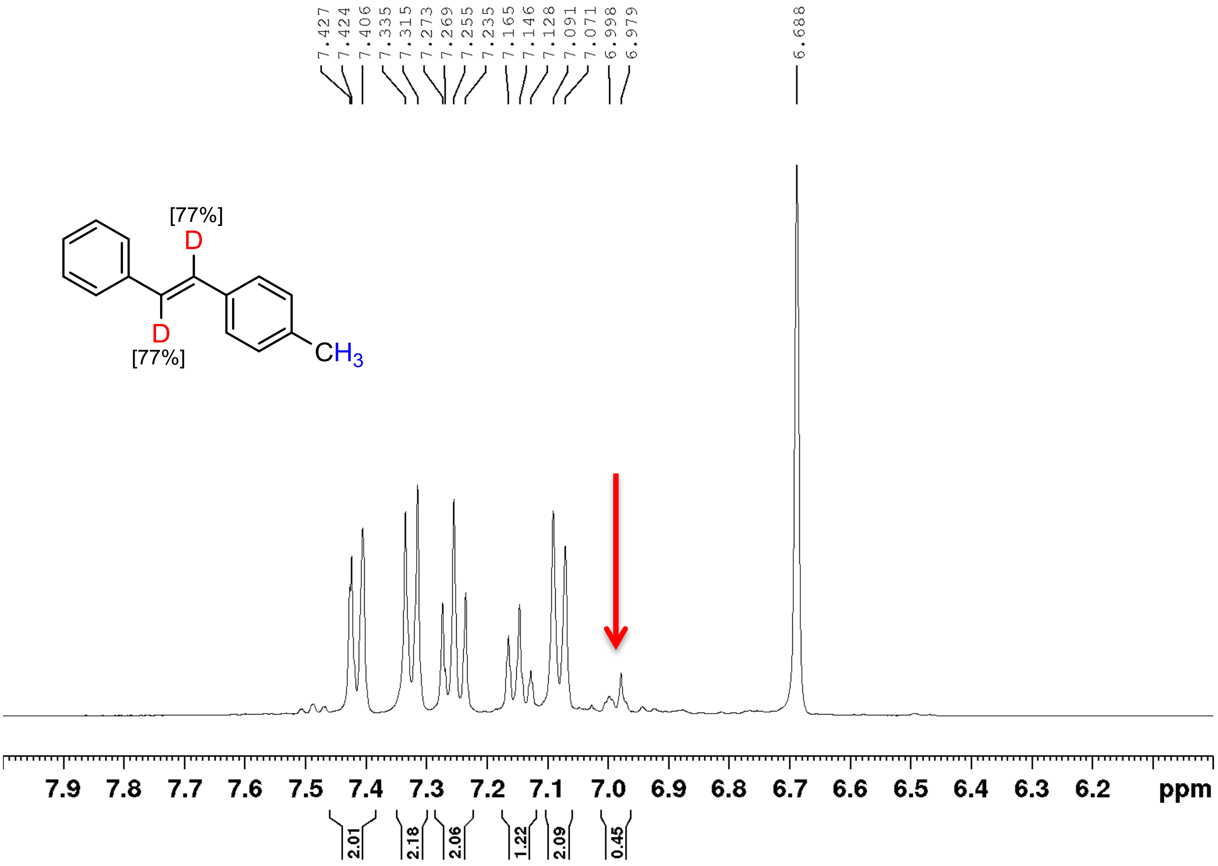

3.5.3 4-(phenylethynyl)anisole 7

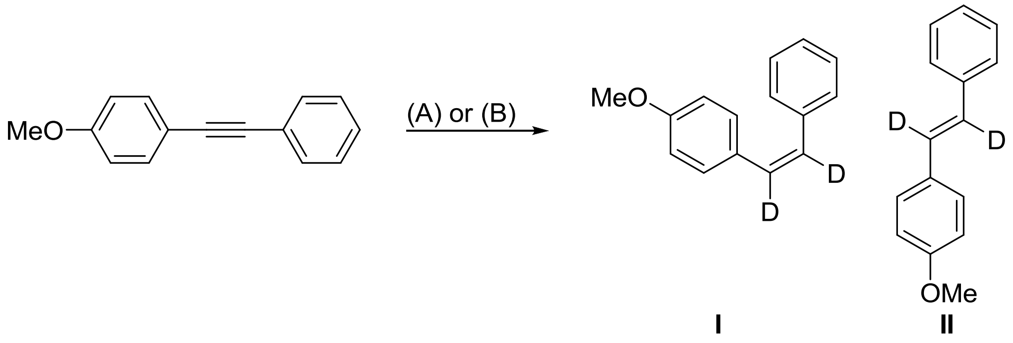

		I (*Z*)		II (*E*)	
	t	D_A	Yield	D_A	Yield
cond. (A)	16 h	86%	37%	75%	57%
	62 h	n.o.	n.o.	77%	>99%
cond. (B)	16 h	-	-	-	-
	62 h	-	-	-	-

4-(phenylethynyl)anisole 10 (I and II)

^{1}H-NMR (400 MHz, CD_2Cl_2) Spectra of pure compound **10**:

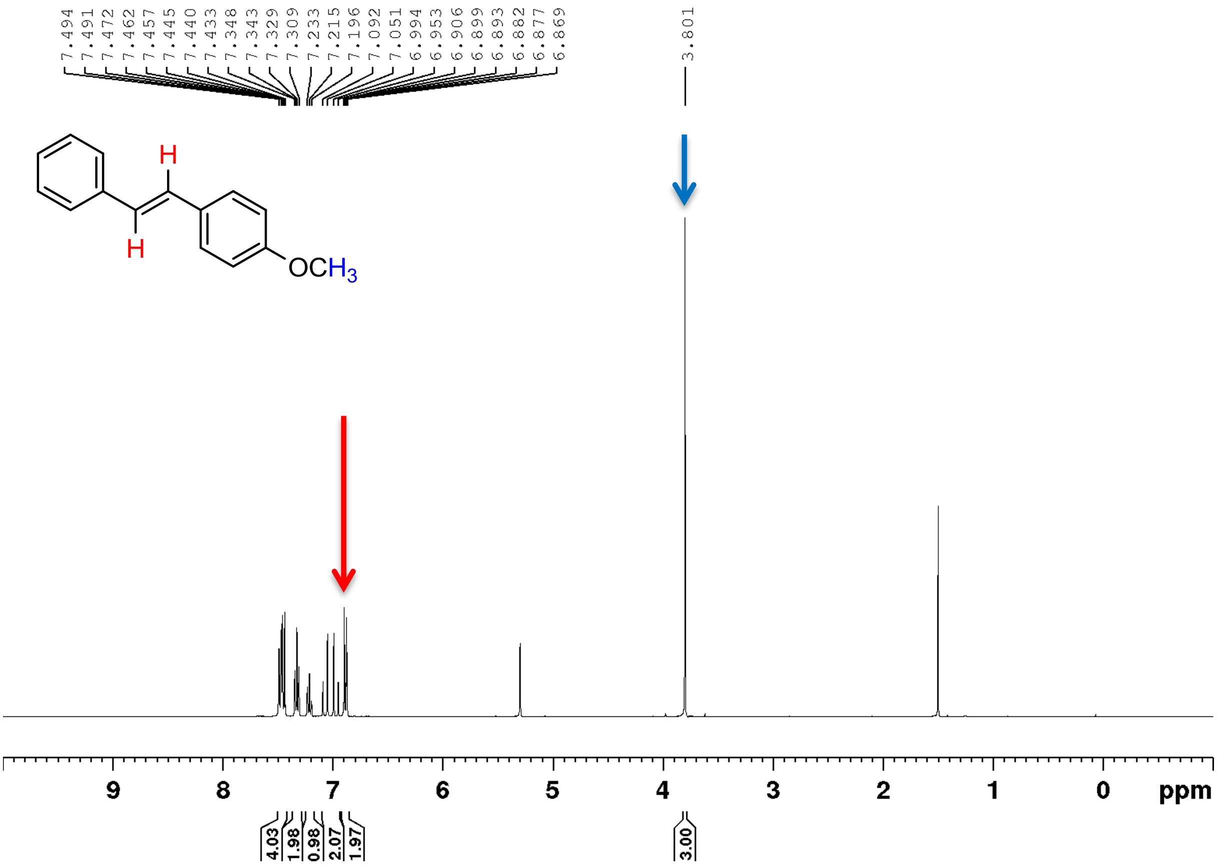

Enlargement of relevant area:

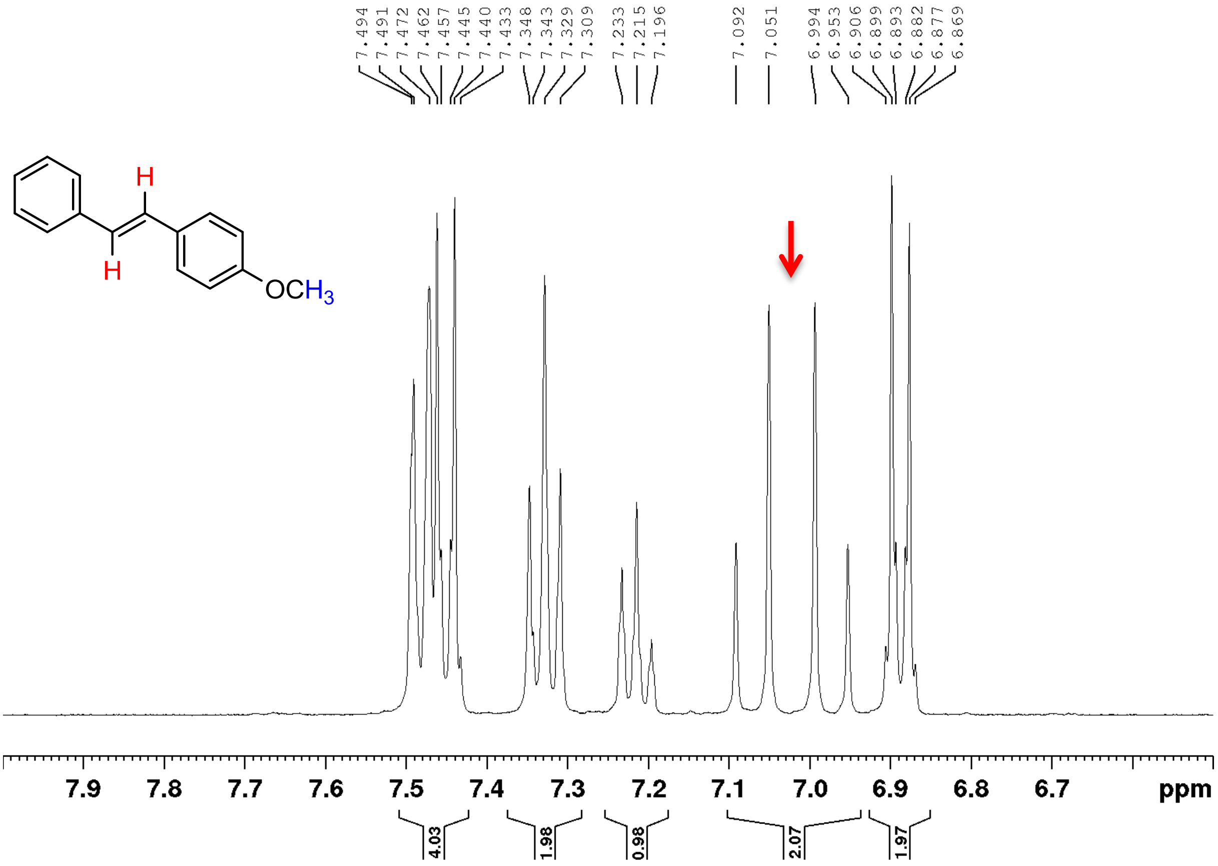

^{1}H-NMR (400 MHz, CD_2Cl_2, mesitylene) Spectra of deuterated compound **10** following the CuI procedure for 16 h: (*Z*/*E*: 1:1.5)

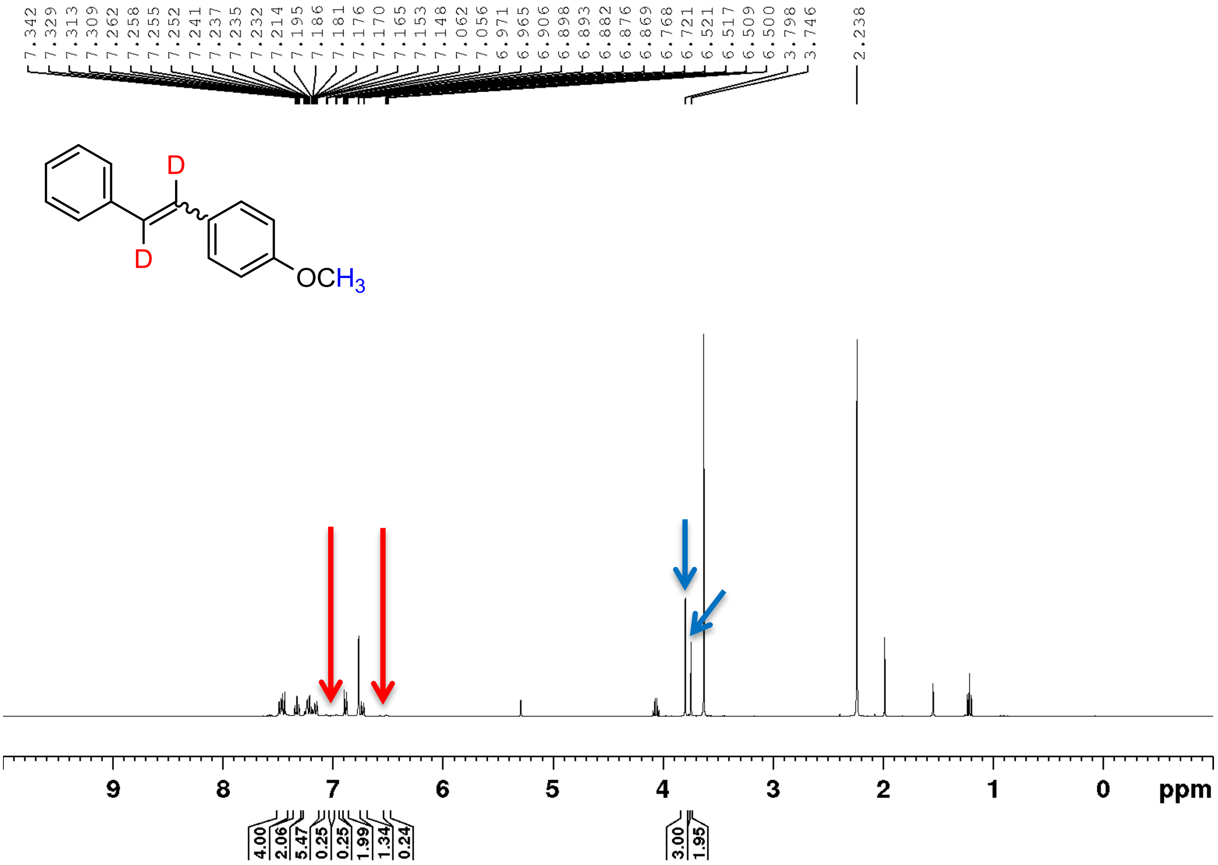

Enlargement of relevant area:

Yield of *Z*-Isomer[9]: 37%

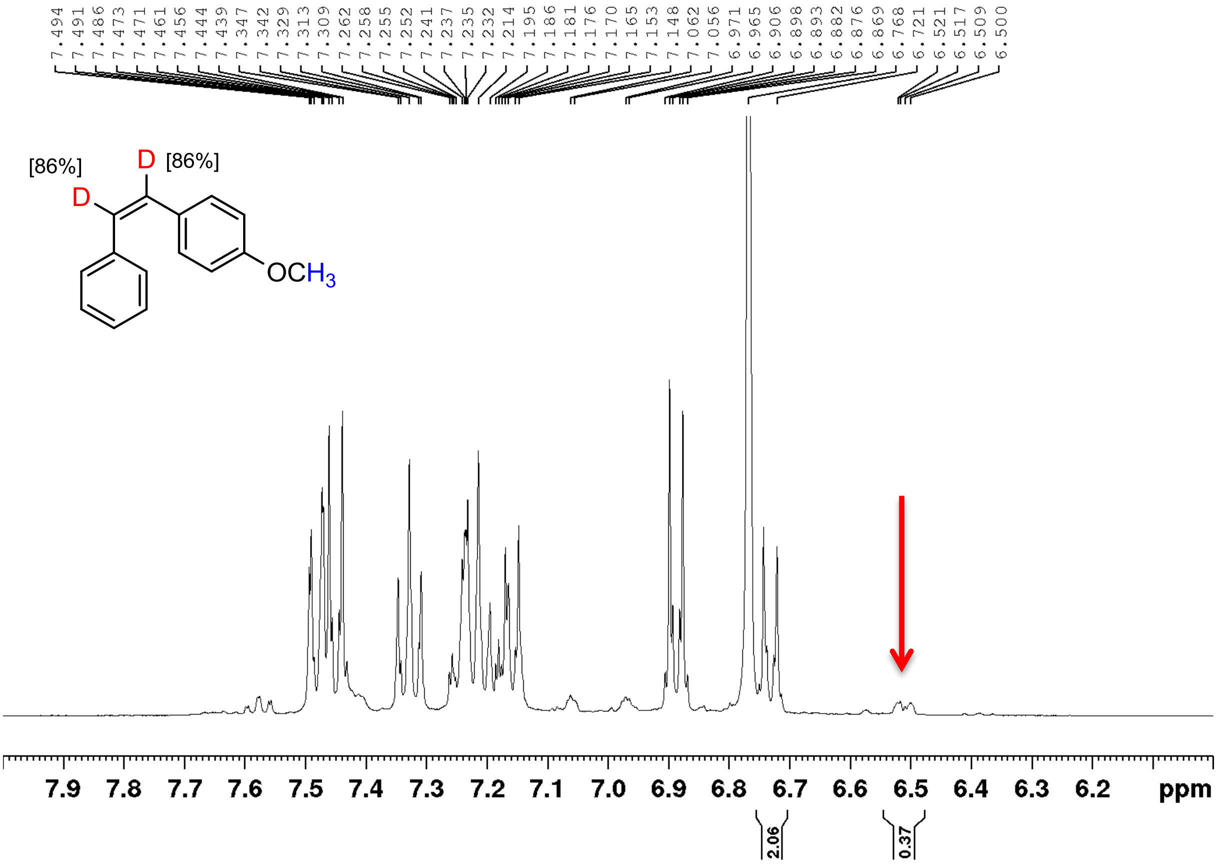

Yield of *E*-Isomer: 57%

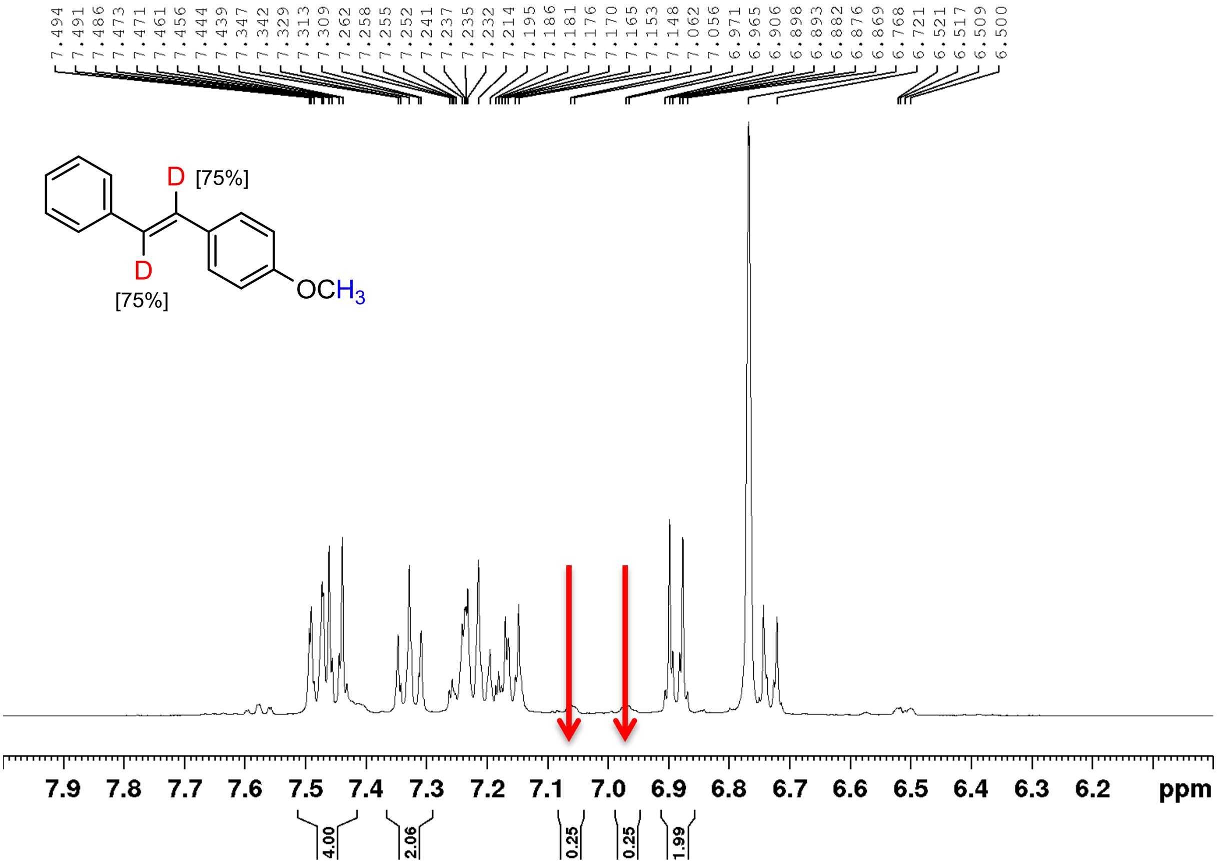

^{1}H-NMR (400 MHz, CD_2Cl_2, mesitylene) Spectra of deuterated compound **10** following the CuI procedure for 62 h: Yield: >99%

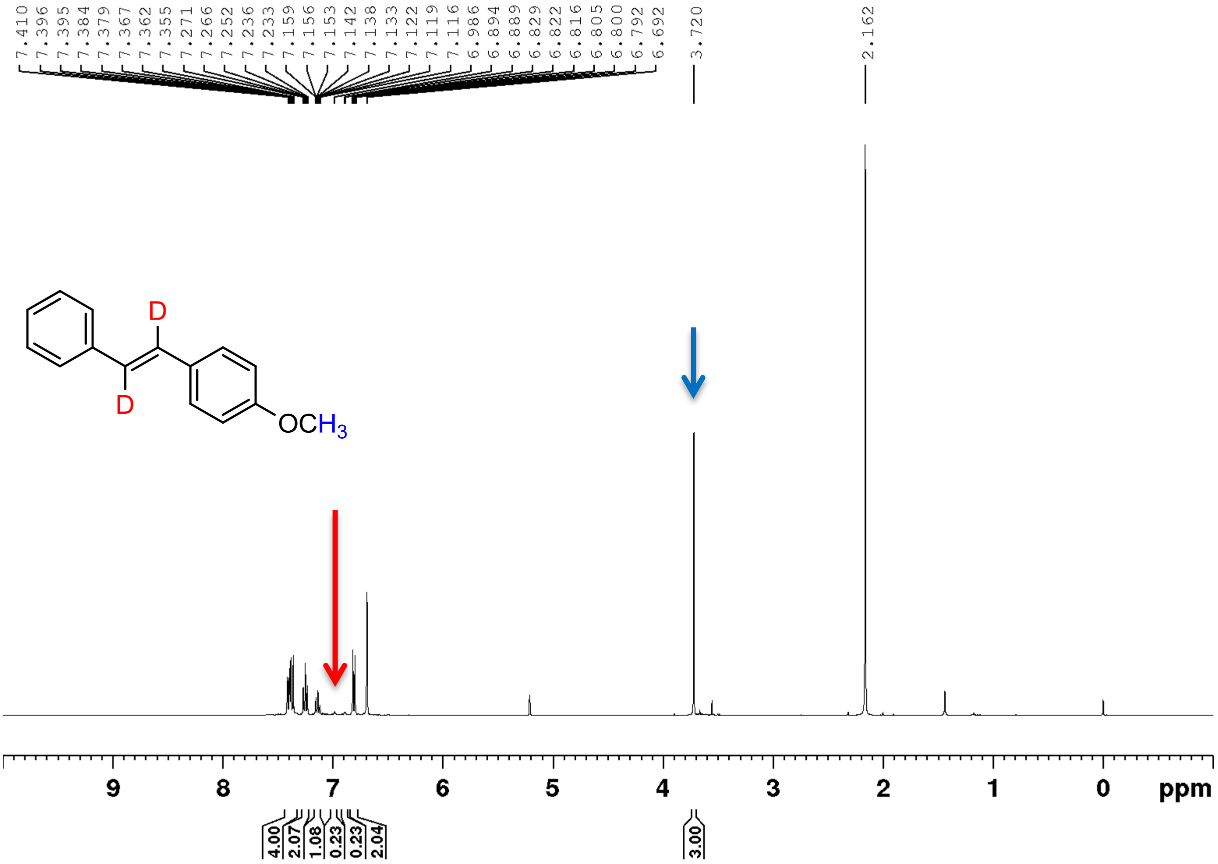

Enlargement of relevant area:

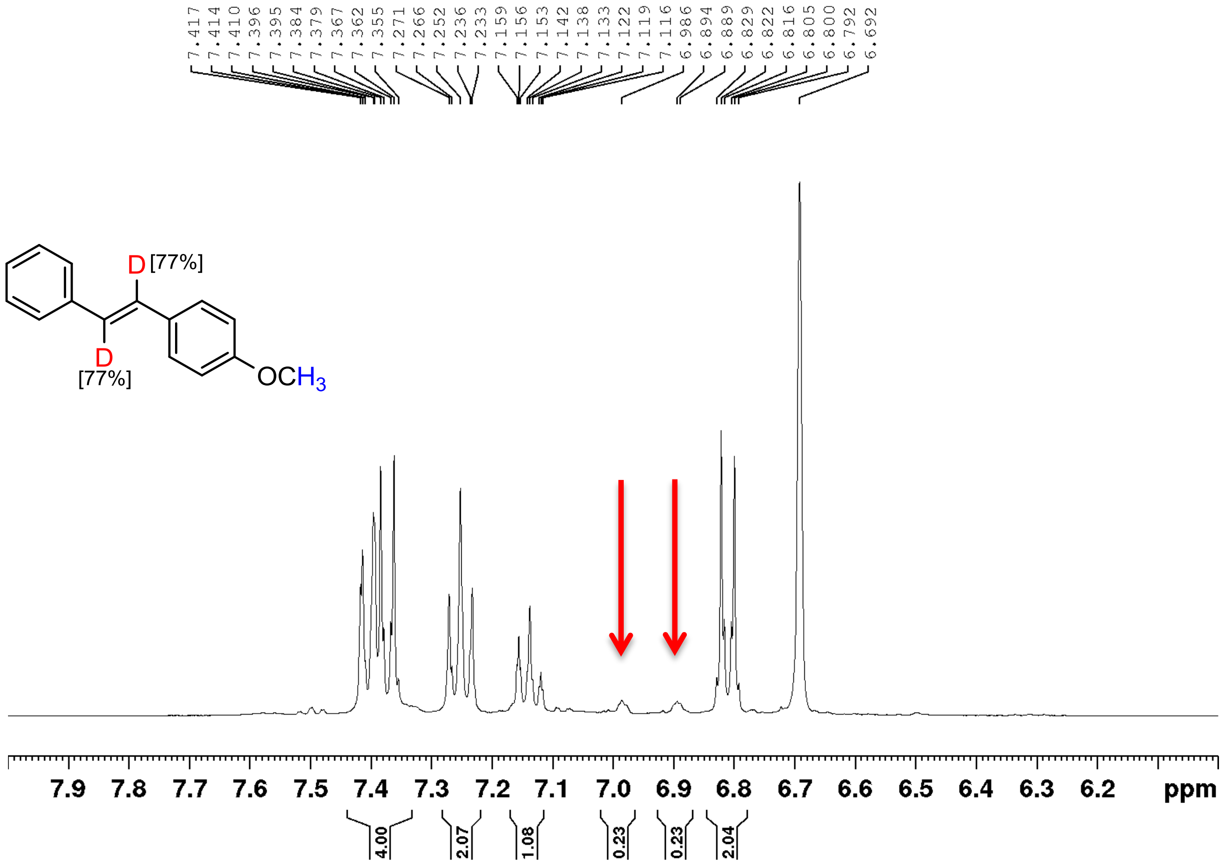

3.5.4 Acetophenone 11

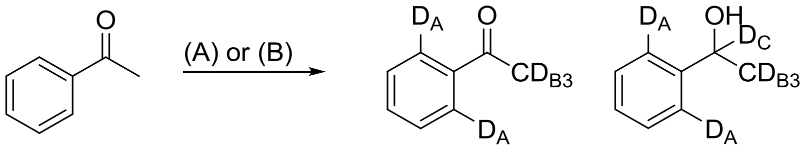

I **II**

	I					II			
	t	D_A	D_B	D_C	Yield	D_A	D_B	D_C	Yield
cond. (A)	16 h	89%	n.o.	n.o.	98%	n.o.	n.o.	n.o.	n.o.
cond. (B)	16 h	50%	79%	n.o.	25%	51%	75%	69%	45%
	62 h	n.o.	n.o.	n.o.	n.o.	65%	74%	81%	78%

Acetophenone 11 (I)

^{1}H-NMR (400 MHz, $CDCl_3$) Spectra of pure compound **11**:

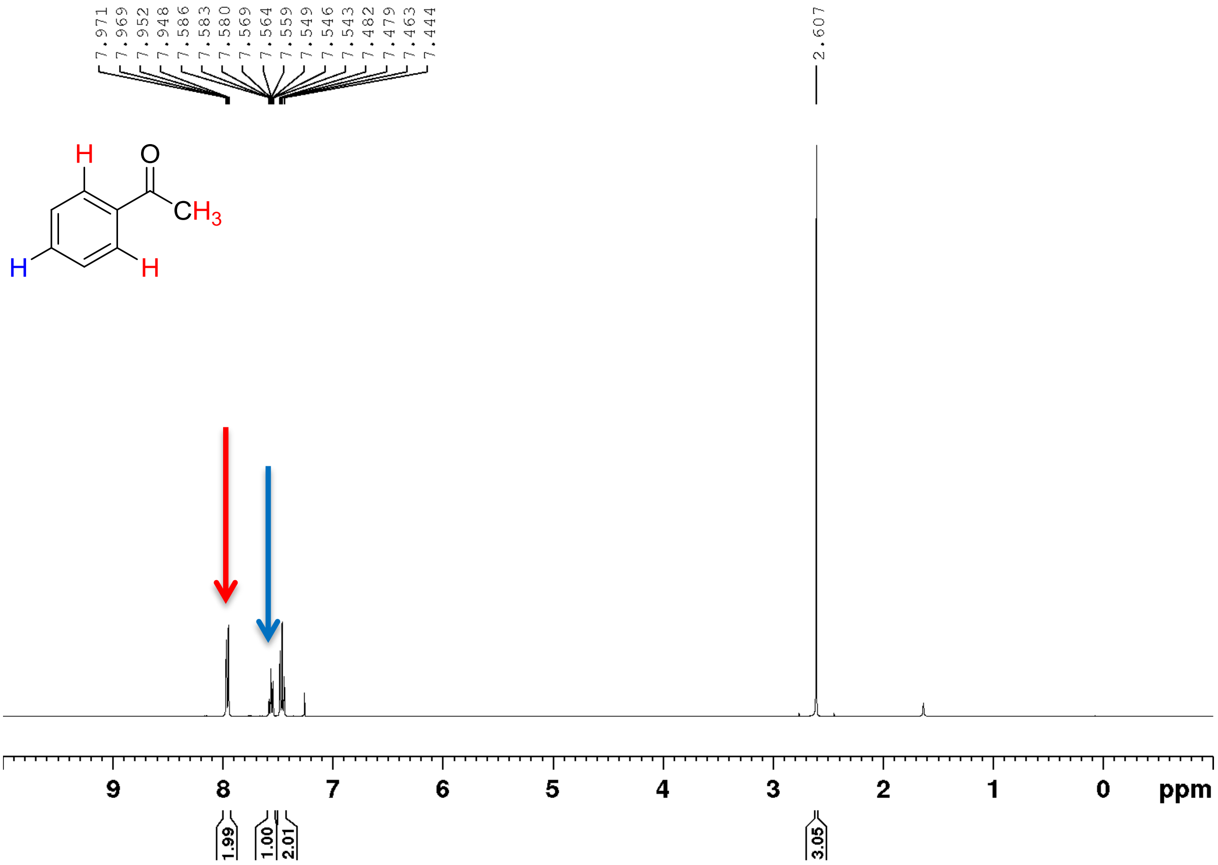

Enlargement of relevant area:

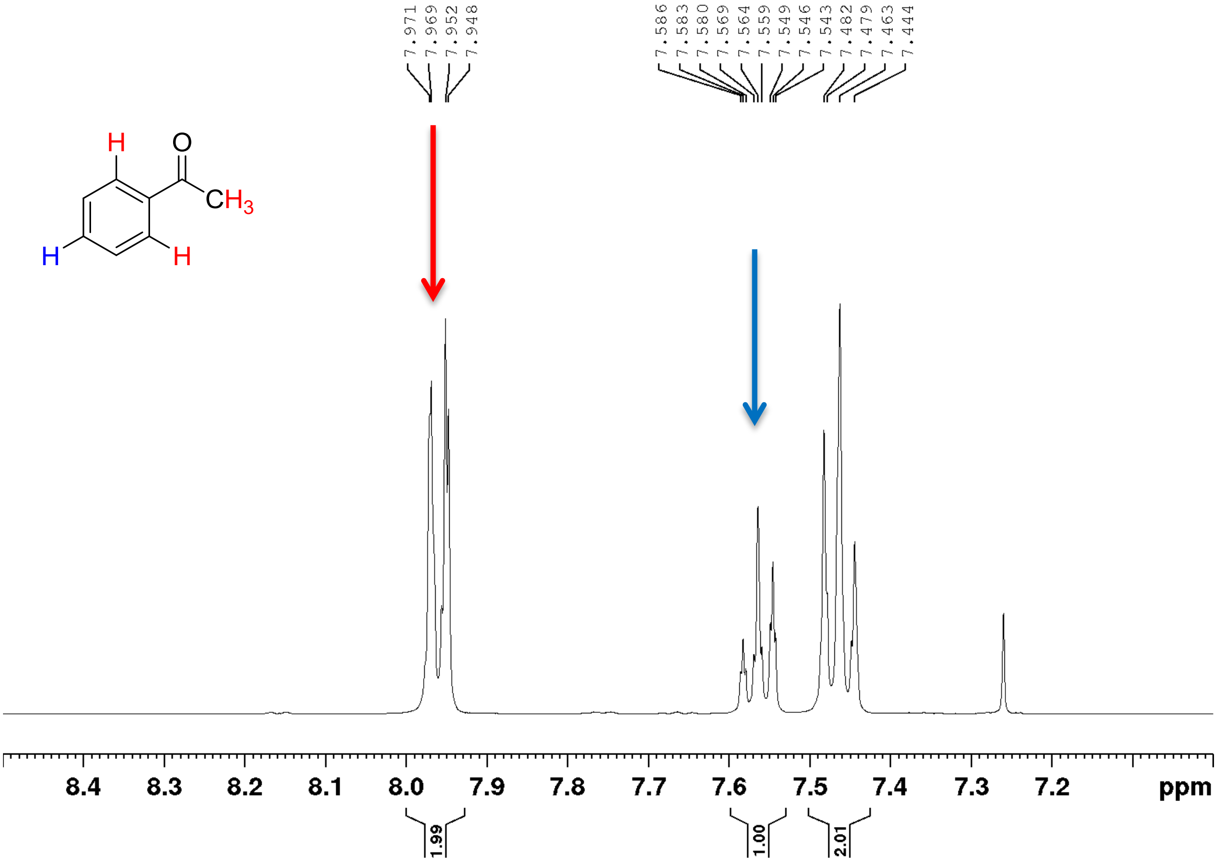

^{1}H-NMR (400 MHz, CD_2Cl_2) Spectra of pure compound **11**:

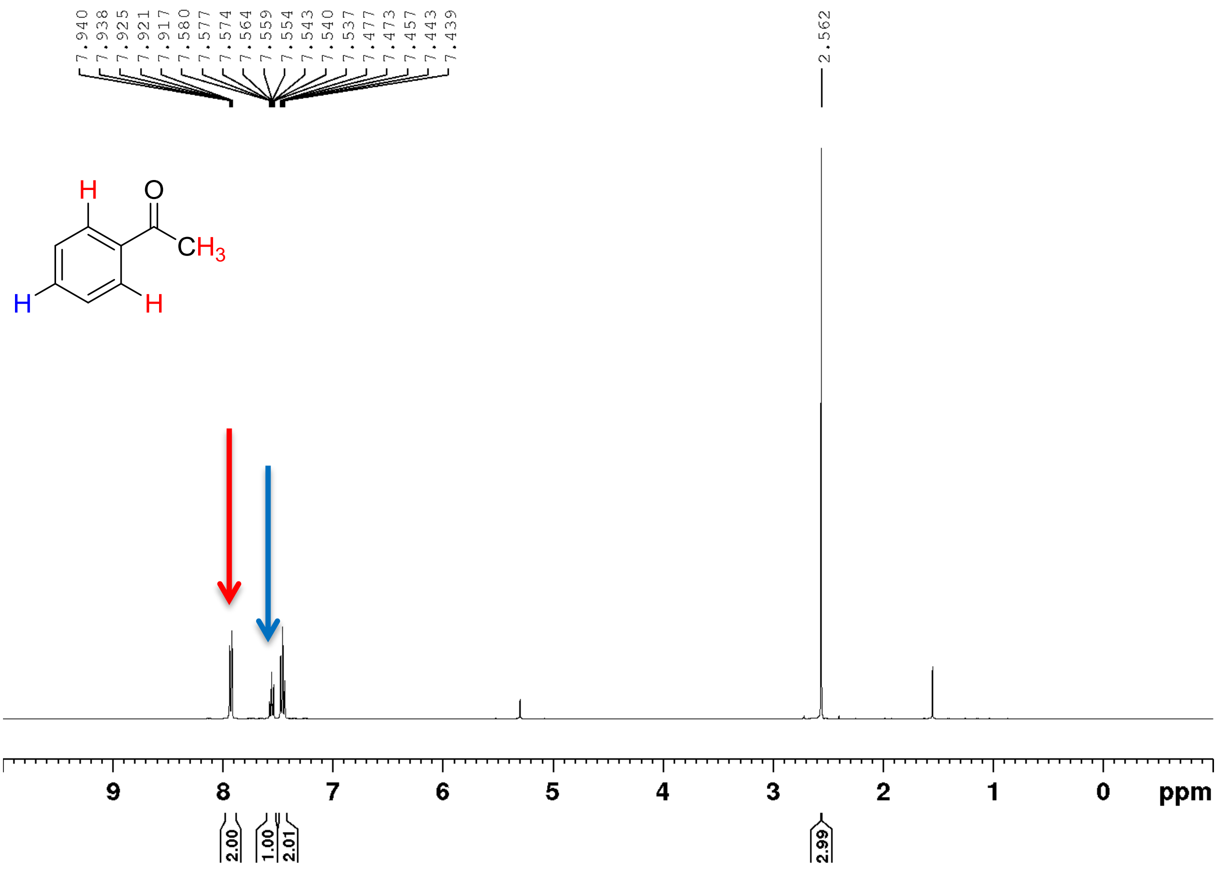

Enlargement of relevant area:

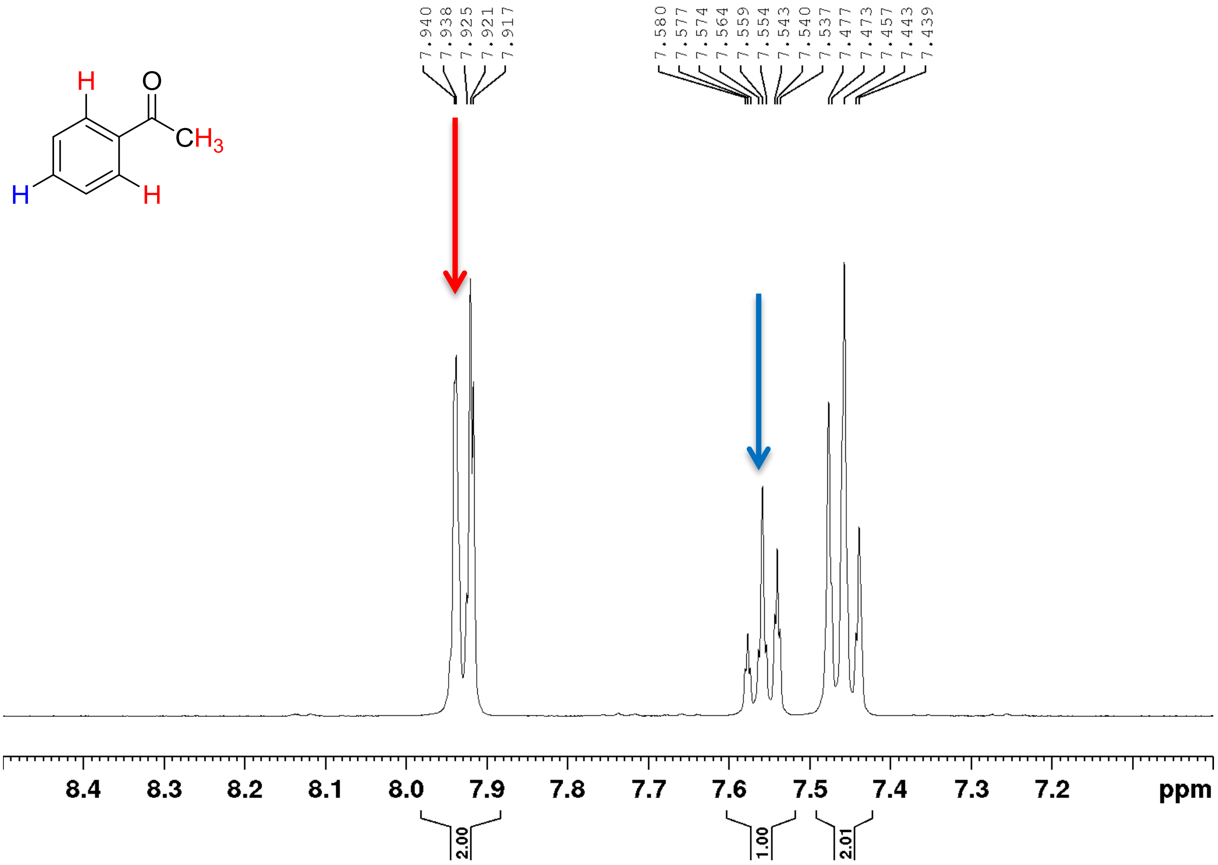

^{1}H-NMR (400 MHz, $CDCl_3$) Spectra of deuterated compound **11** following the KOD/Zn procedure for 16 h: Yield: 25%

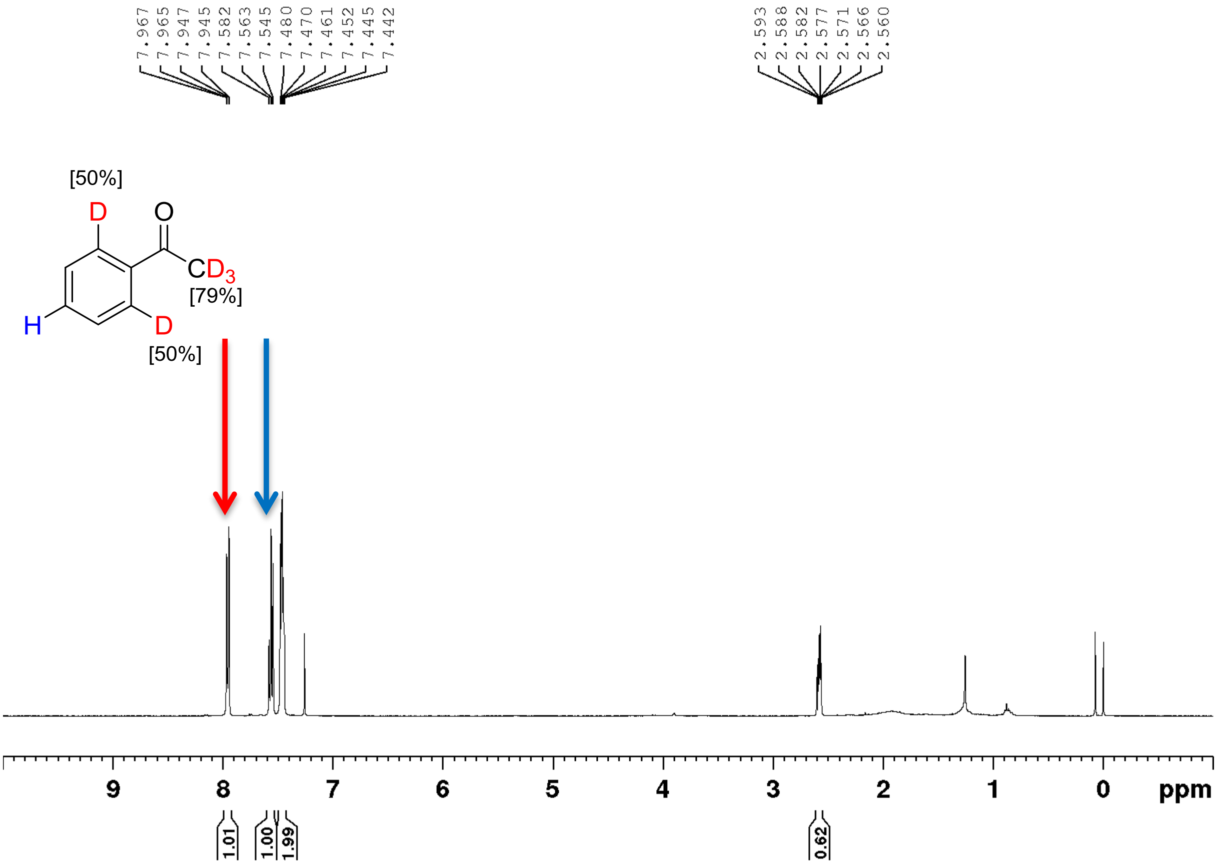

^{1}H-NMR (400 MHz, CD_2Cl_2, mesitylene) Spectra of deuterated compound **11** following the CuI procedure for 16 h: Yield: 98%

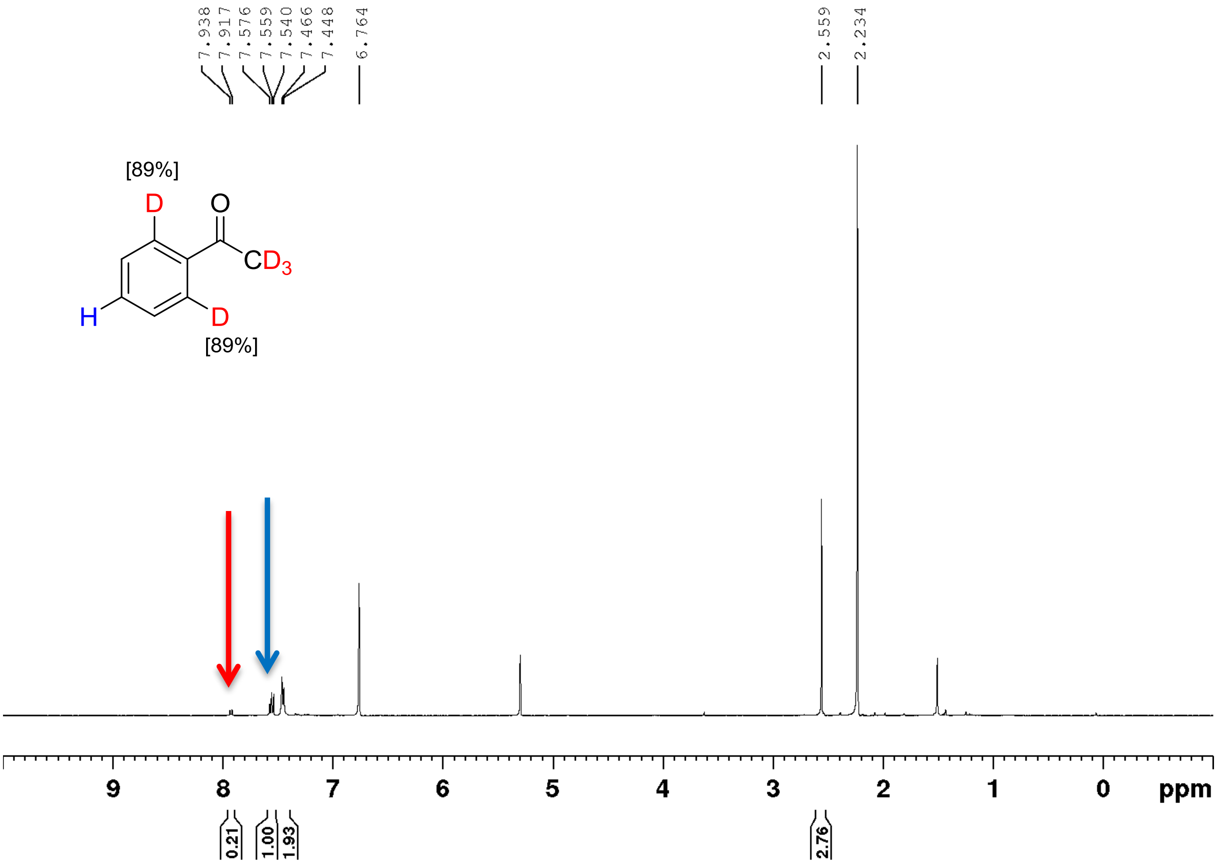

Enlargement of relevant area:

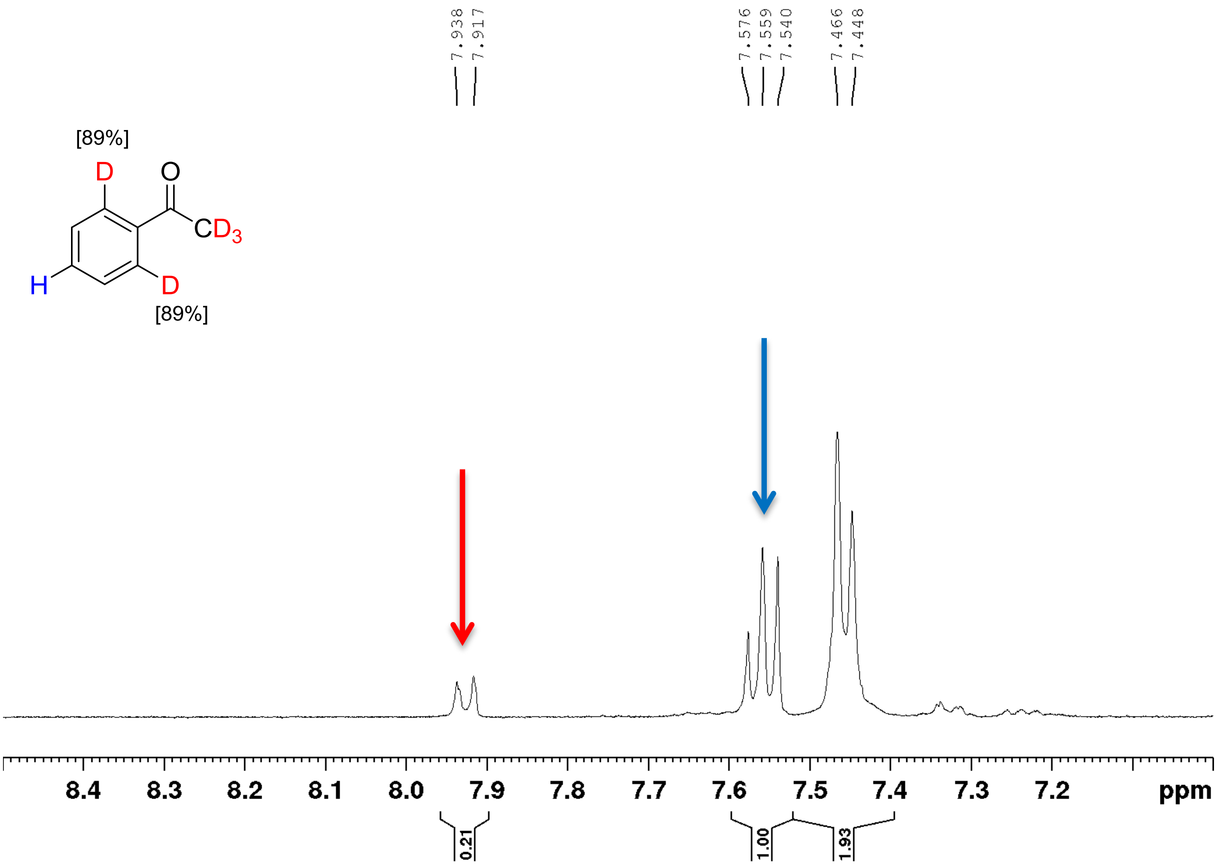

1-Phenylethanol 14

^{1}H-NMR (400 MHz, THF-d_8) Spectra of reduced substrate **14:**

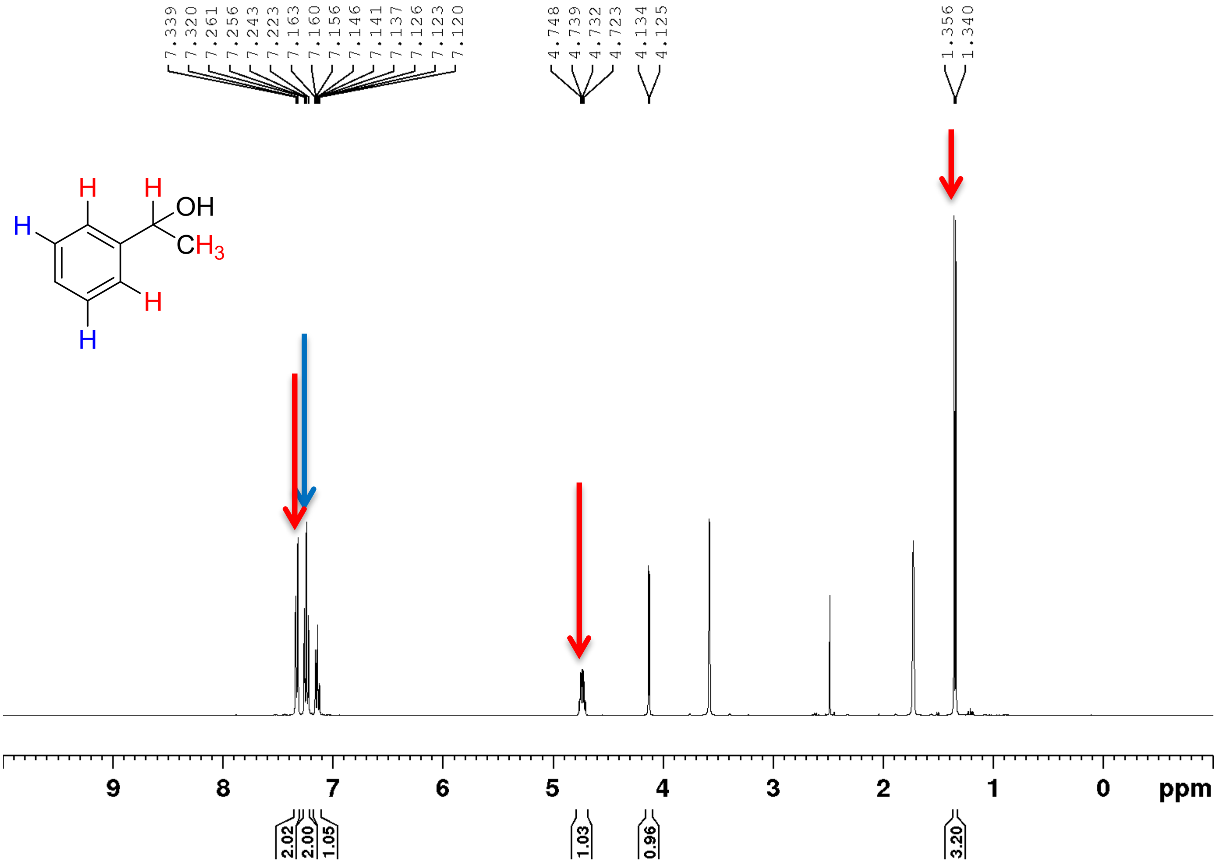

^{1}H-NMR (400 MHz, THF-d_8) Spectra of deuterated compound **14** following the KOD/Zn procedure for 16 h: Yield: 45%

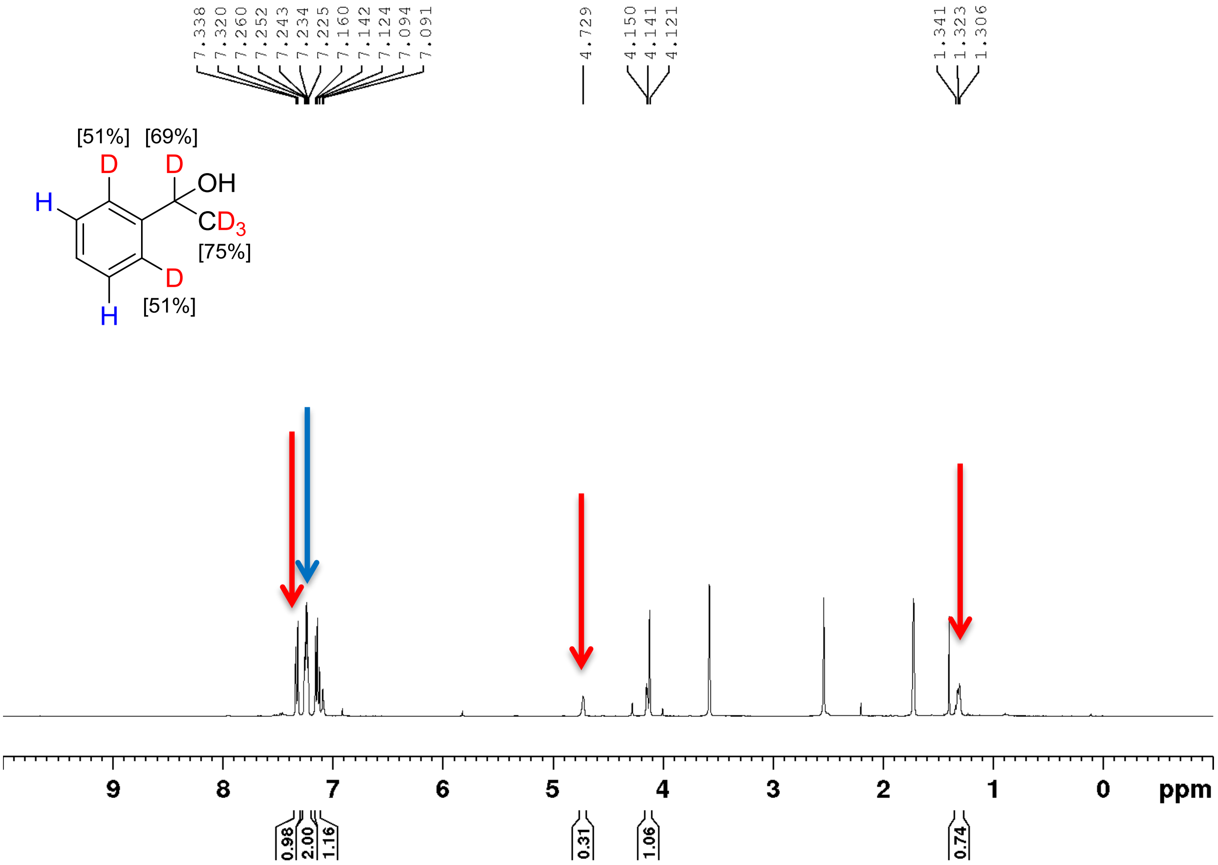

Enlargement of relevant area:

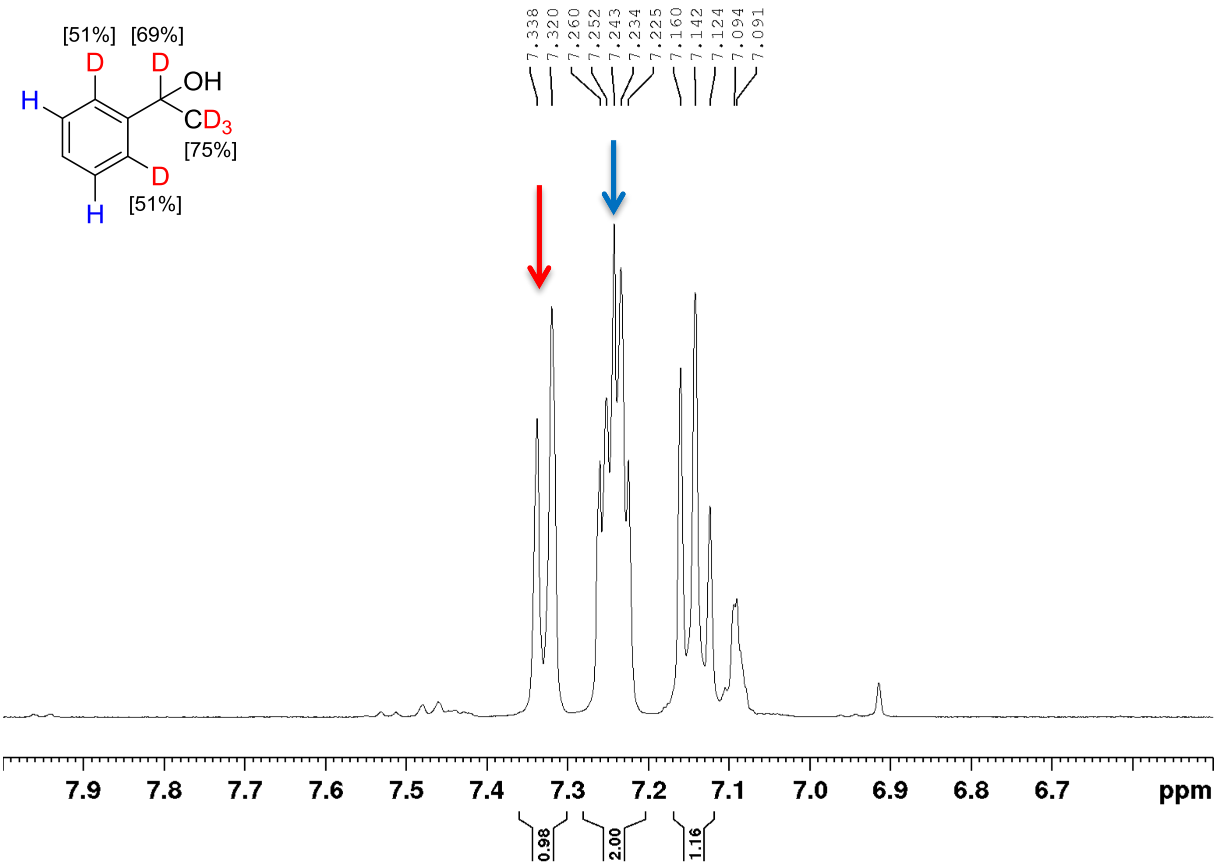

^{1}H-NMR (400 MHz, THF-d_8) Spectra of deuterated compound **14** following the KOD/Zn procedure for 62 h: Yield: 78%.

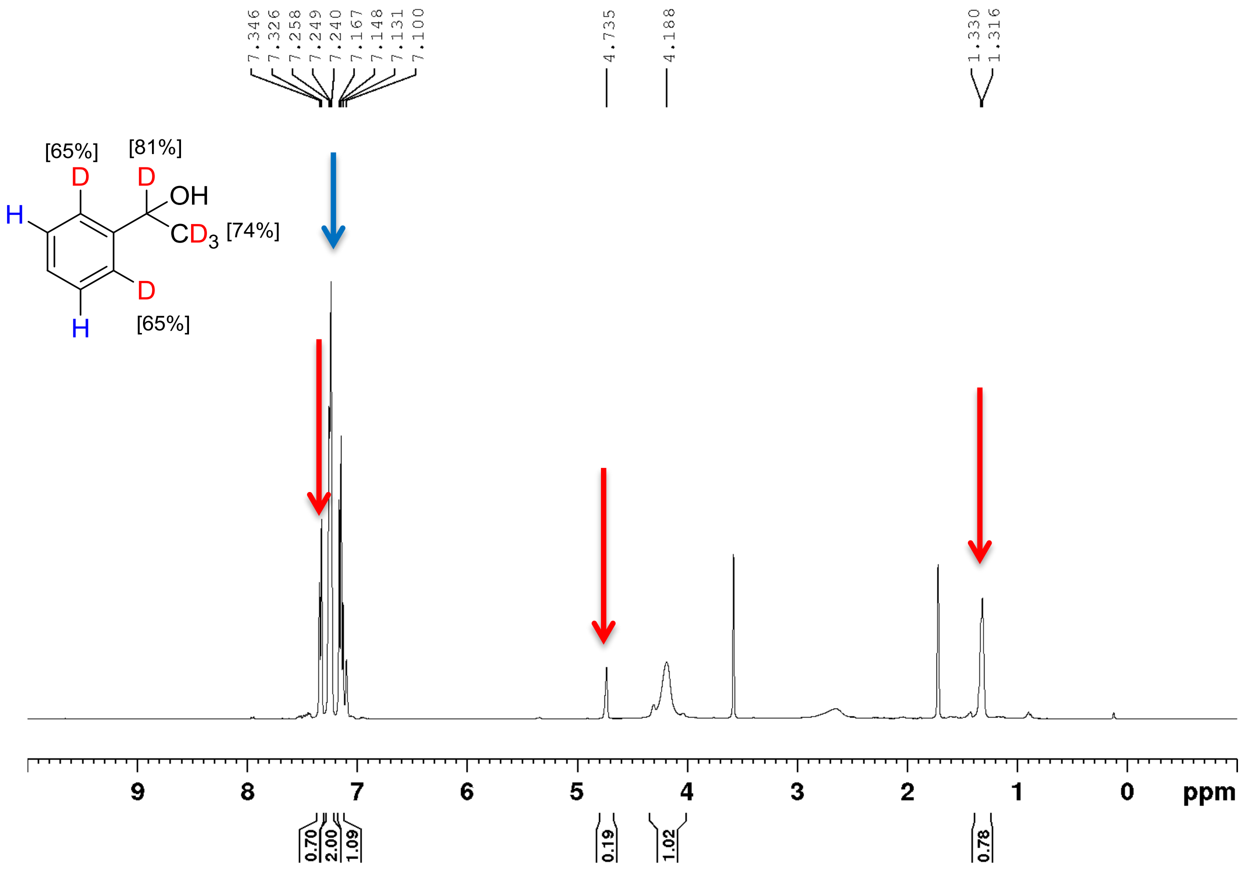

Enlargement of relevant area:

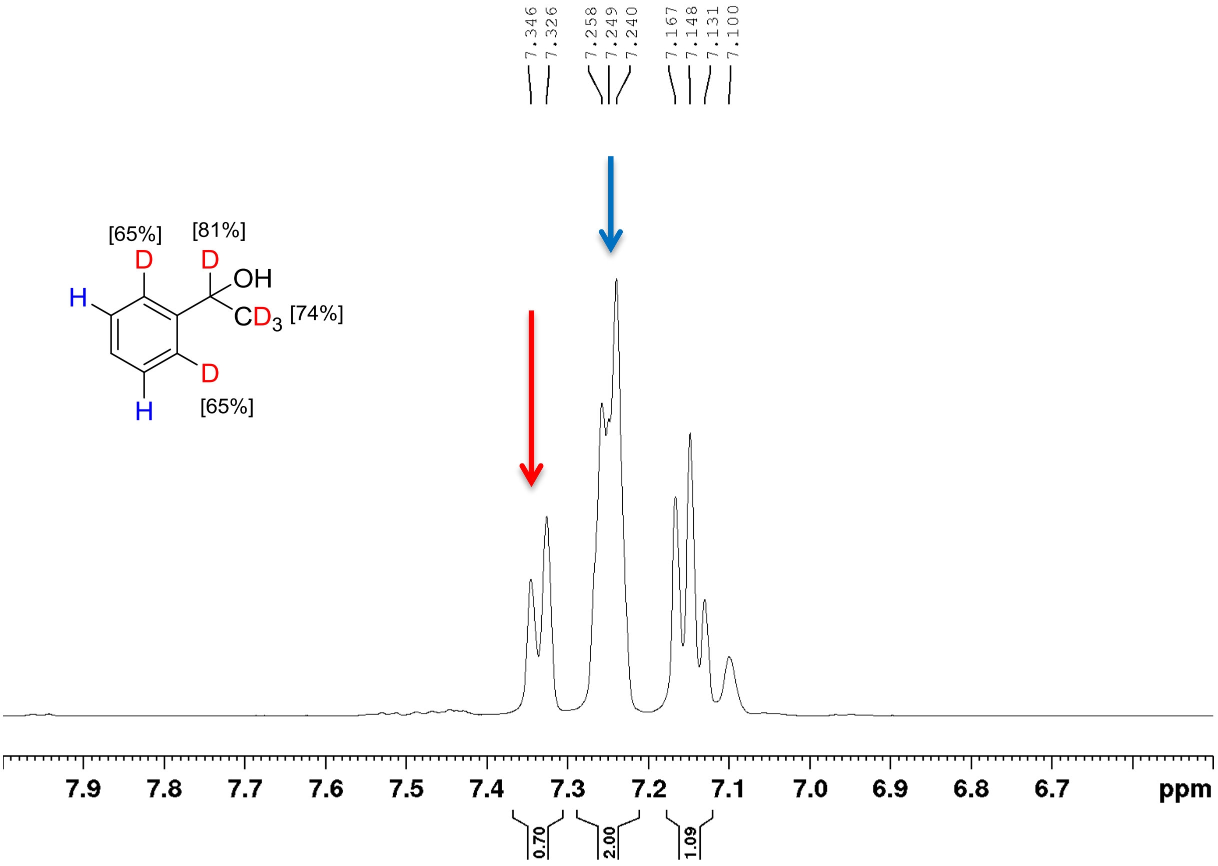

3.5.5 4-Methylacetophenone 12

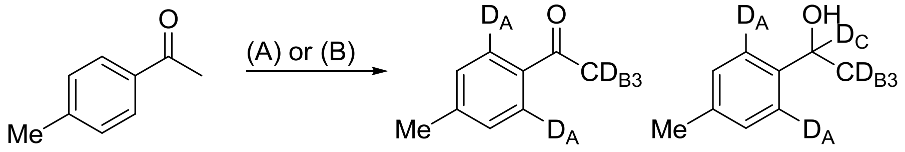

I II

		I				II			
	t	D_A	D_B	D_C	Yield	D_A	D_B	D_C	Yield
cond. (A)	16 h	63%	n.o.	n.o.	93%	n.o.	n.o.	n.o.	n.o.
	62 h	-	-	-	-	-	-	-	-
cond. (B)	16 h	69%	81%	n.o.	49%	66%	80%	71%	47%
	62 h	n.o.	n.o.	n.o.	n.o.	74%	78%	81%	86%

4-Methylacetophenone 12 (I)

^{1}H-NMR (400 MHz, CD_2Cl_2) Spectra of pure compound **12**:

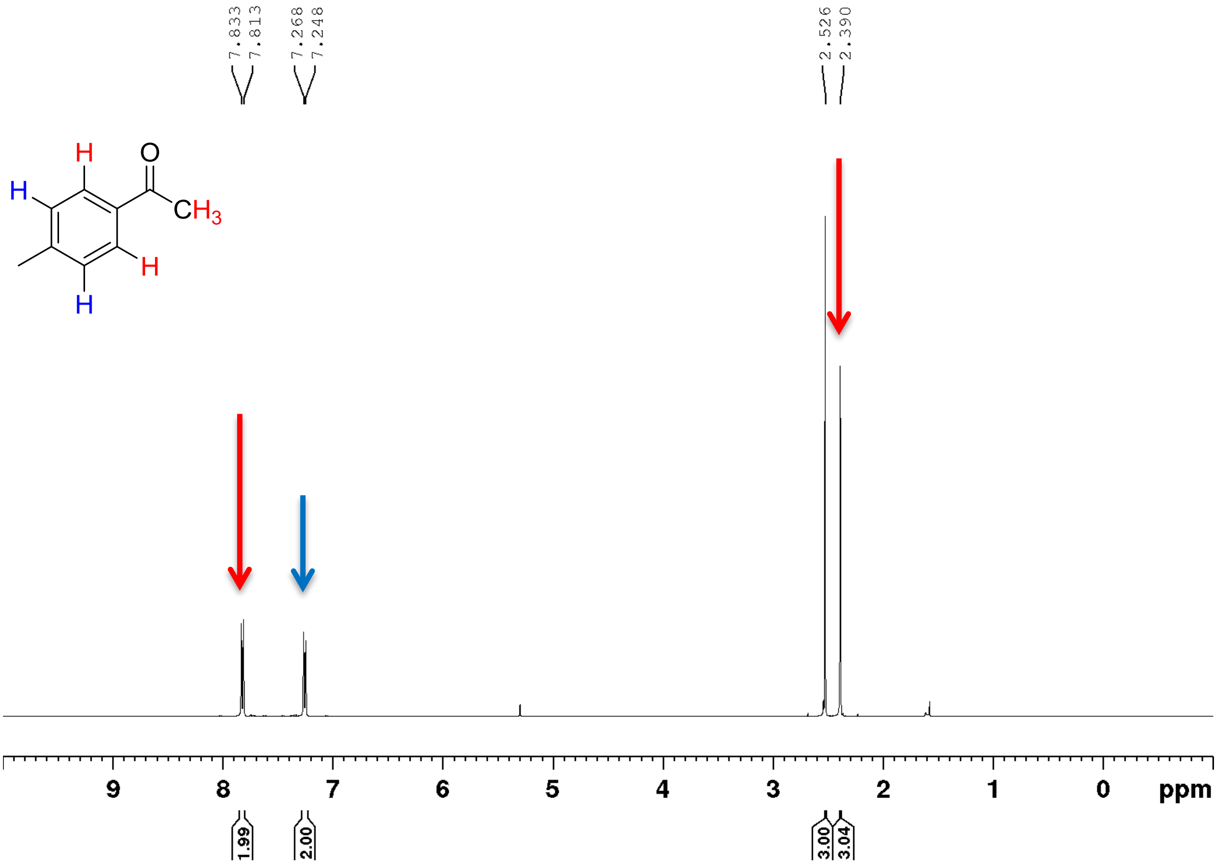

^{1}H-NMR (400 MHz, CD_2Cl_2, mesitylene) Spectra of deuterated compound **12** following the CuI procedure for 16 h**:** Yield: 93%

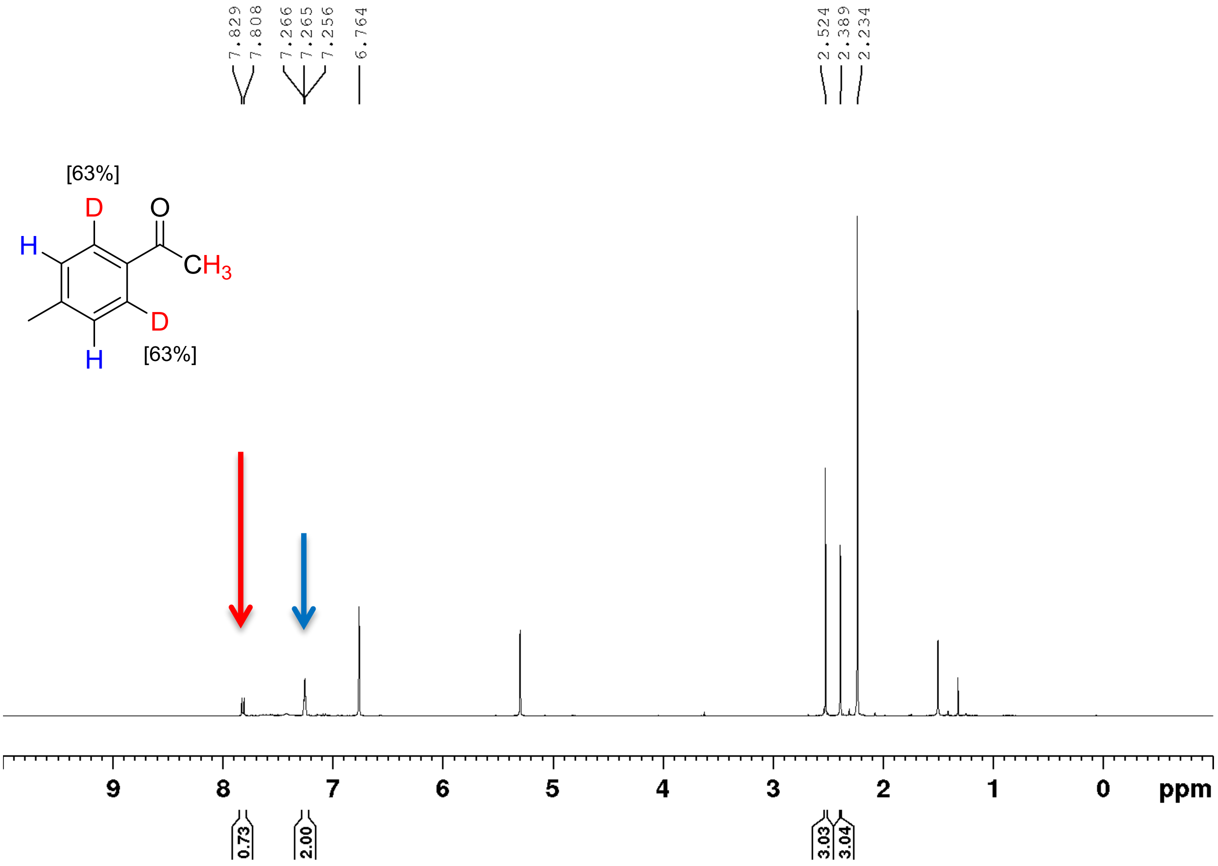

1-(4-Methylphenyl)ethanol 15 (II)

^{1}H-NMR (400 MHz, $CDCl_3$) Spectra of pure compound **15**:

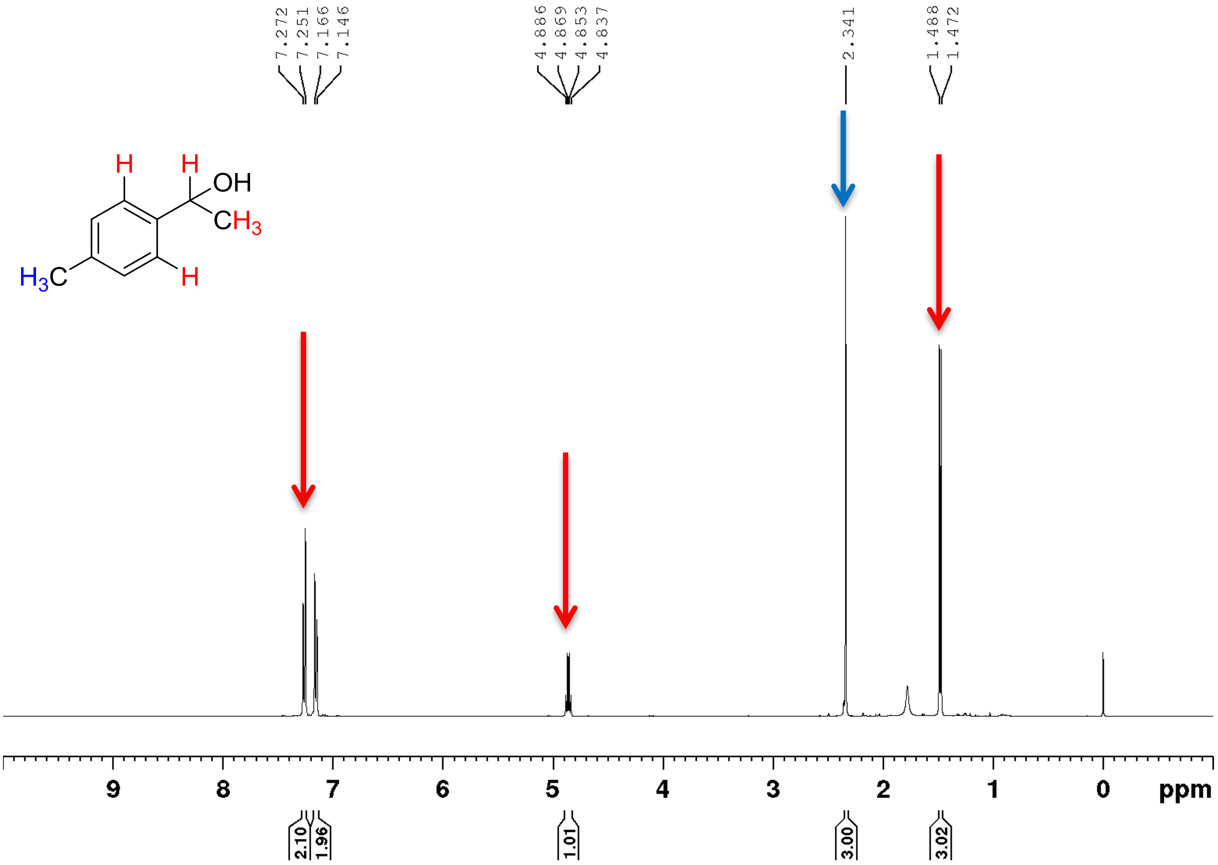

^{1}H-NMR (400 MHz, CD_2Cl_2) Spectra of deuterated compound **15** following the KOD/Zn procedure for 16 h: Yield: 47%

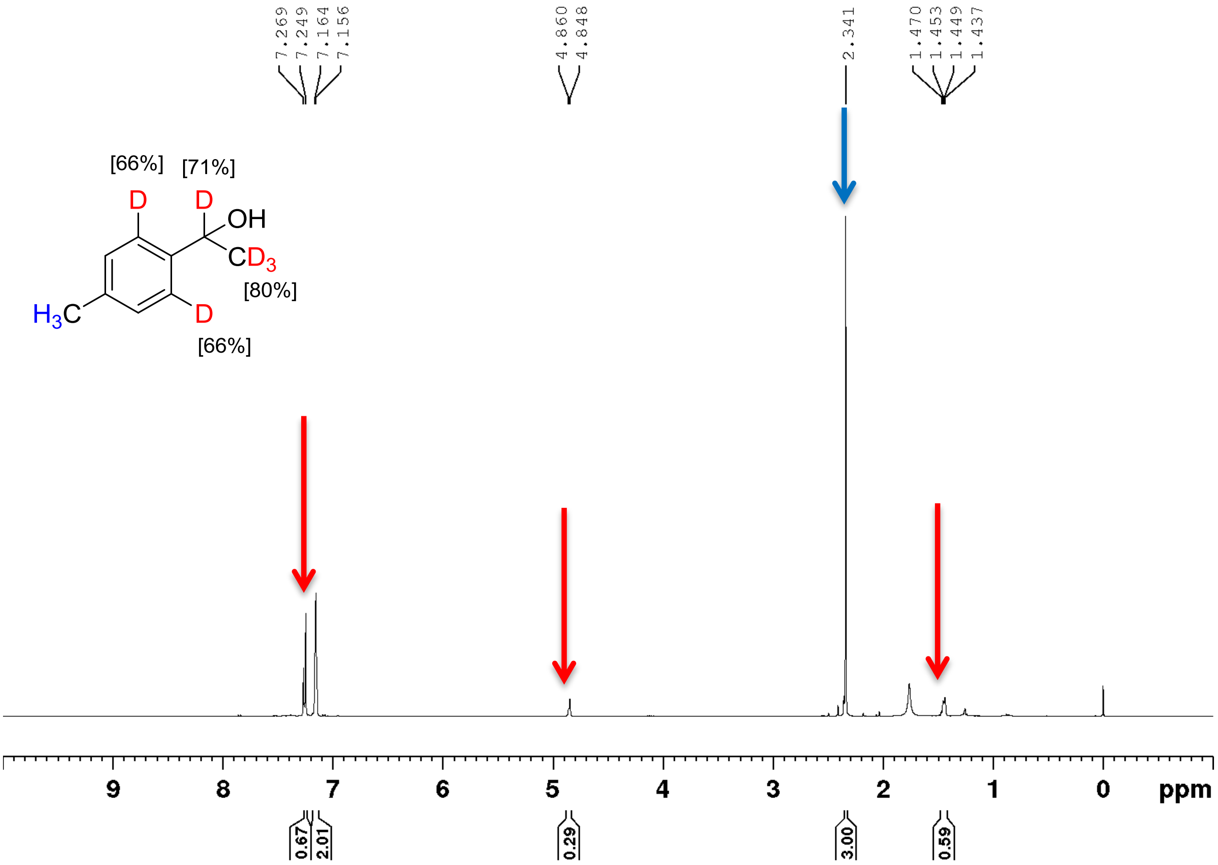

^{1}H-NMR (400 MHz, CD_2Cl_2, mesitylene) Spectra of deuterated compound **15** following the KOD/Zn procedure for 62 h: Yield: 86%

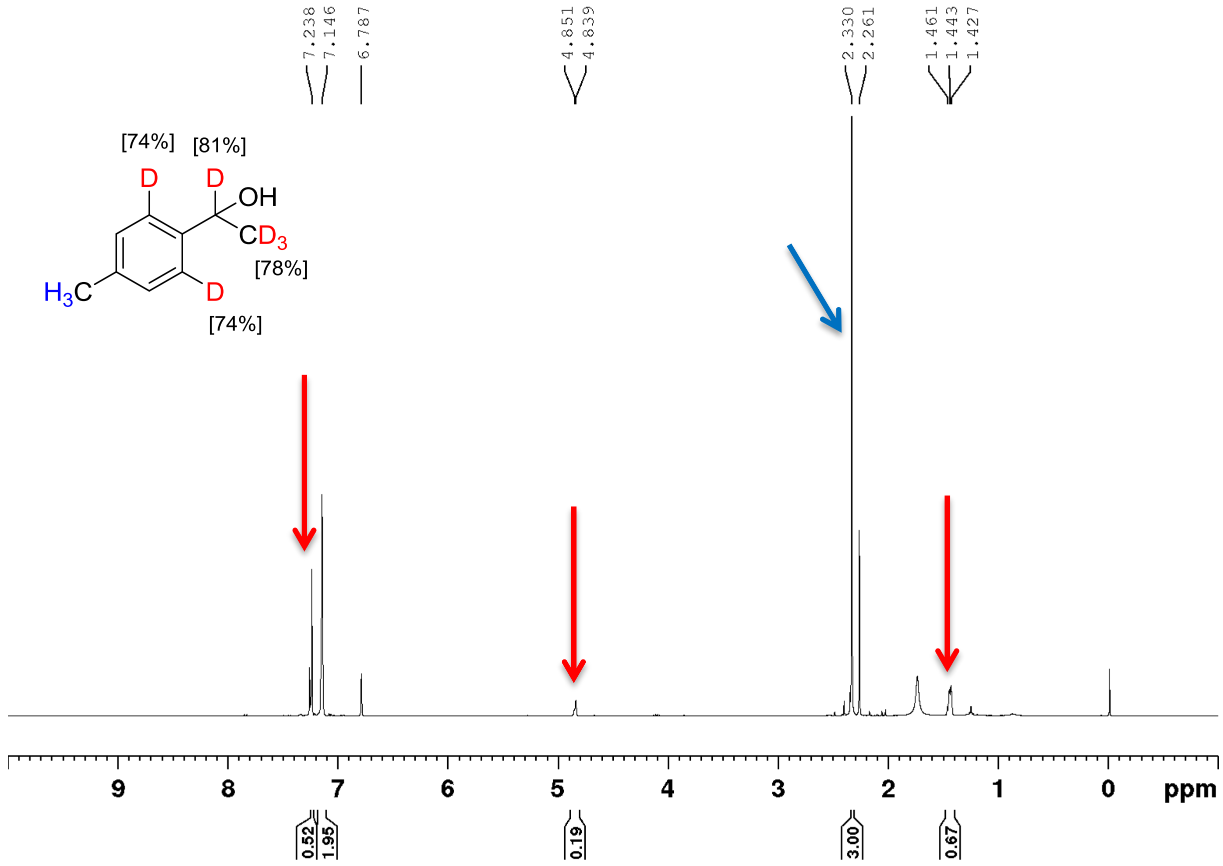

3.5.6 4-Methoxyacetophenone 13

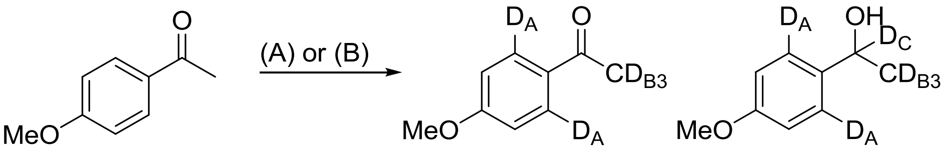

I **II**

	I					II			
	t	D_A	D_B	D_C	Yield	D_A	D_B	D_C	Yield
cond. (A)	16 h	67%	n.o.	n.o.	>99%	n.o.	n.o.	n.o.	n.o.
	62 h	-	-	-	-	-	-	-	-
cond. (B)	16 h	69%	81%	n.o.	49%	70%	79%	77%	41%
	62 h	-	-	-	-	77%	77%	80%	>99%

4-Methoxyacetophenone 13 (I)

^{1}H-NMR (400 MHz, CD_2Cl_2) Spectra of pure compound **13**:

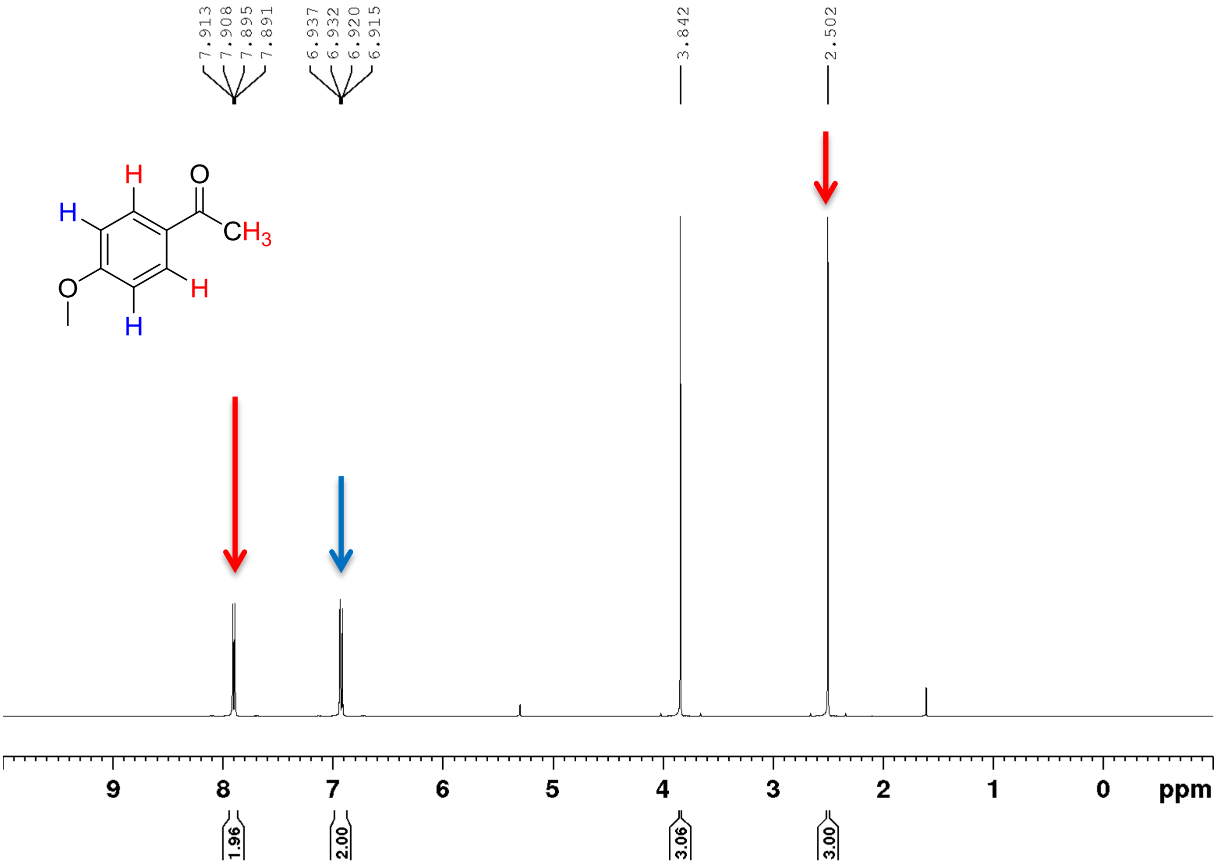

^{1}H-NMR (400 MHz, CD_2Cl_2, mesitylene) Spectra of deuterated compound **13** following the CuI procedure for 16 h: Yield: >99%

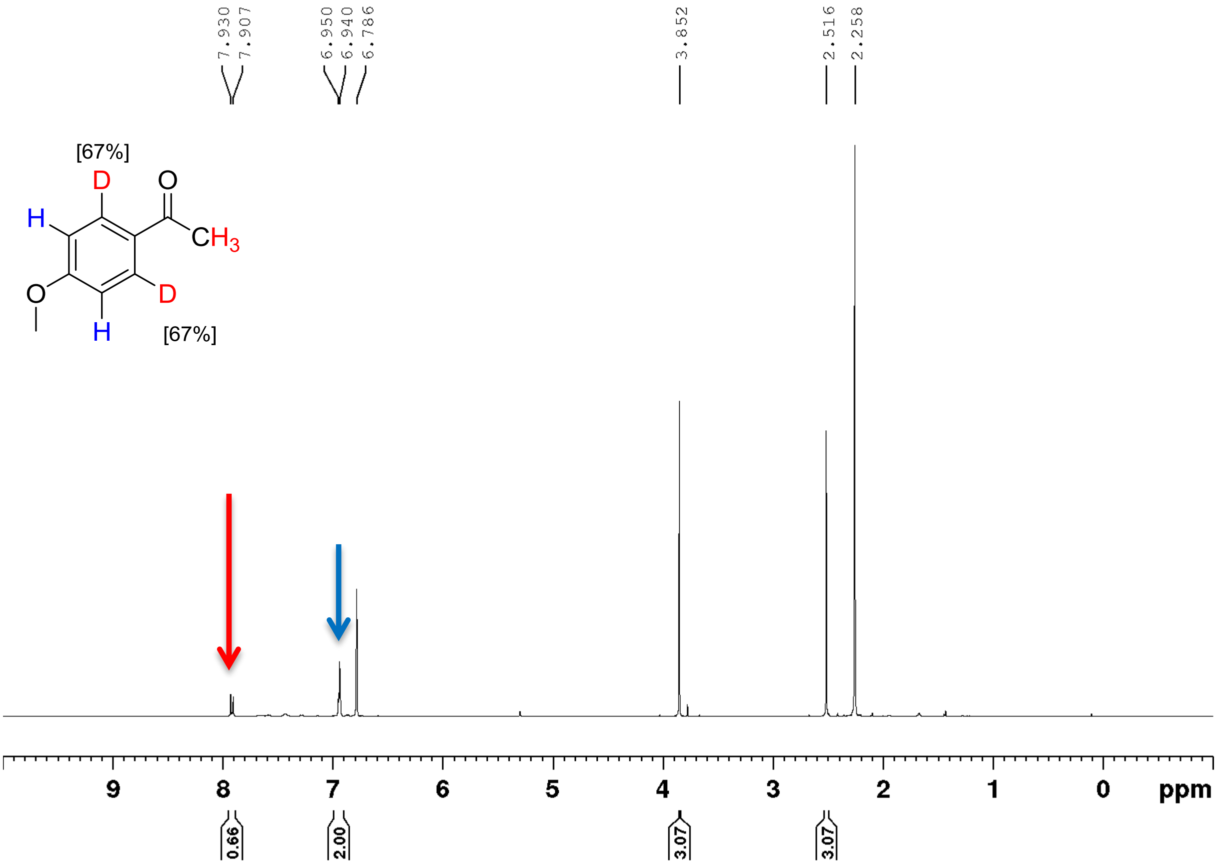

^{1}H-NMR (400 MHz, $CDCl_3$) Spectra of deuterated compound **13** in CD_2Cl_2 following the KOD/Zn procedure for 16 h: Yield: 49%

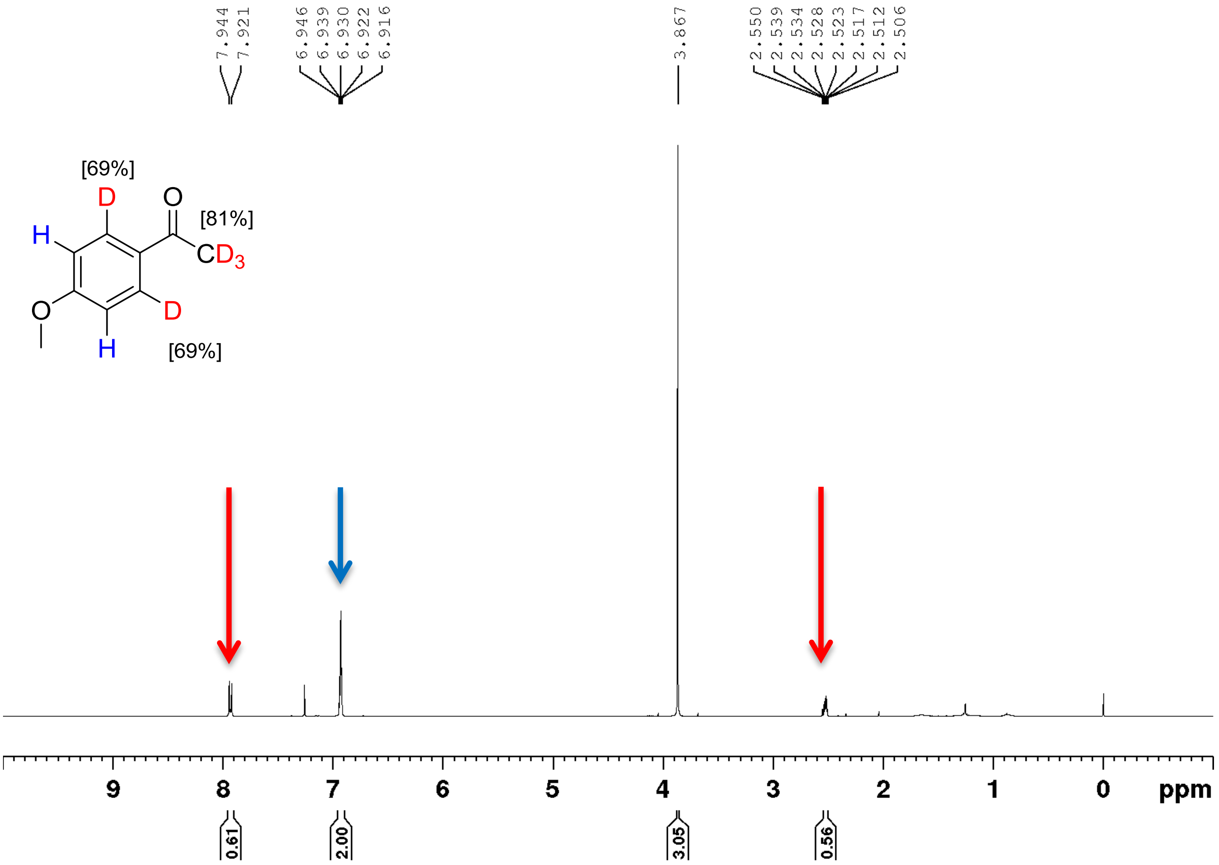

1-(4’-methoxyphenyl)ethanol 16 (II)

^{1}H-NMR (400 MHz, $CDCl_3$) Spectra of pure compound **16**:

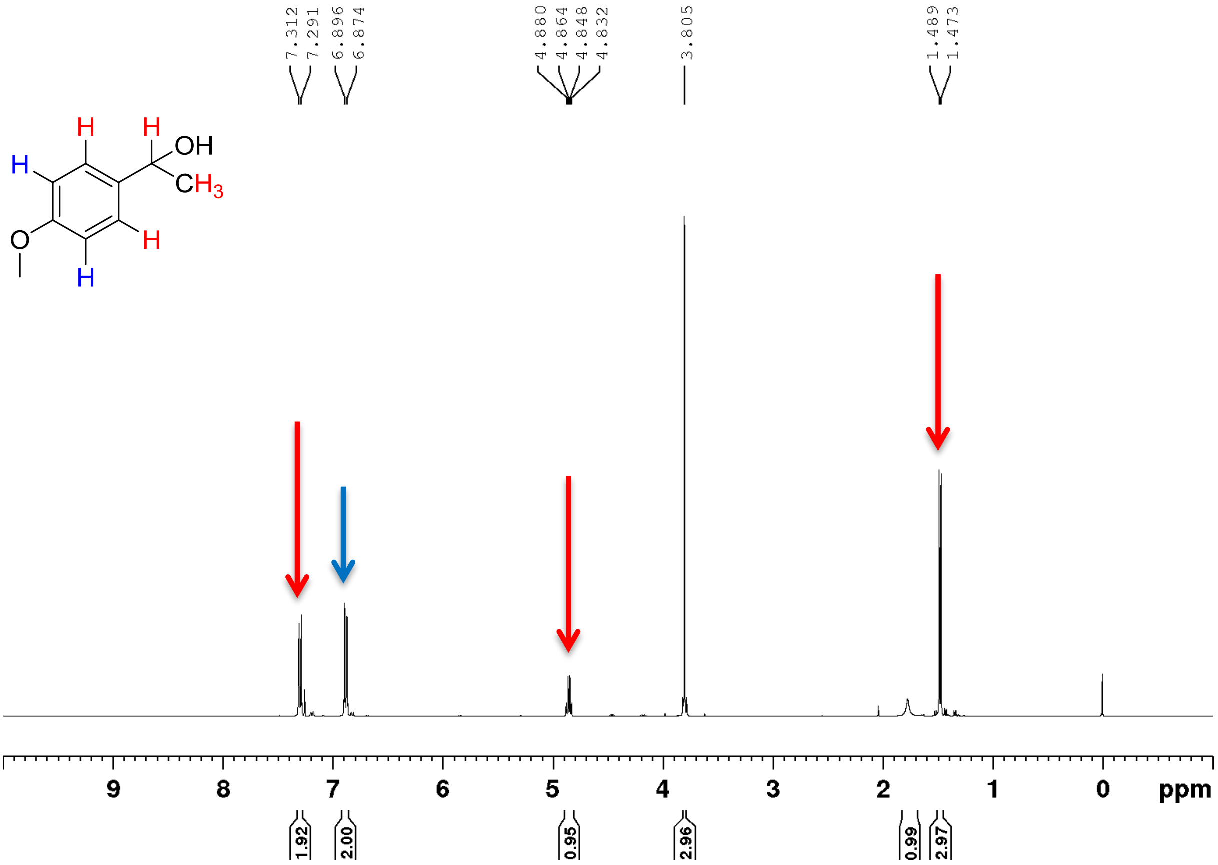

^{1}H-NMR (400 MHz, $CDCl_3$) Spectra of deuterated compound **16** following the KOD/Zn procedure for 16 h: Yield: 41%

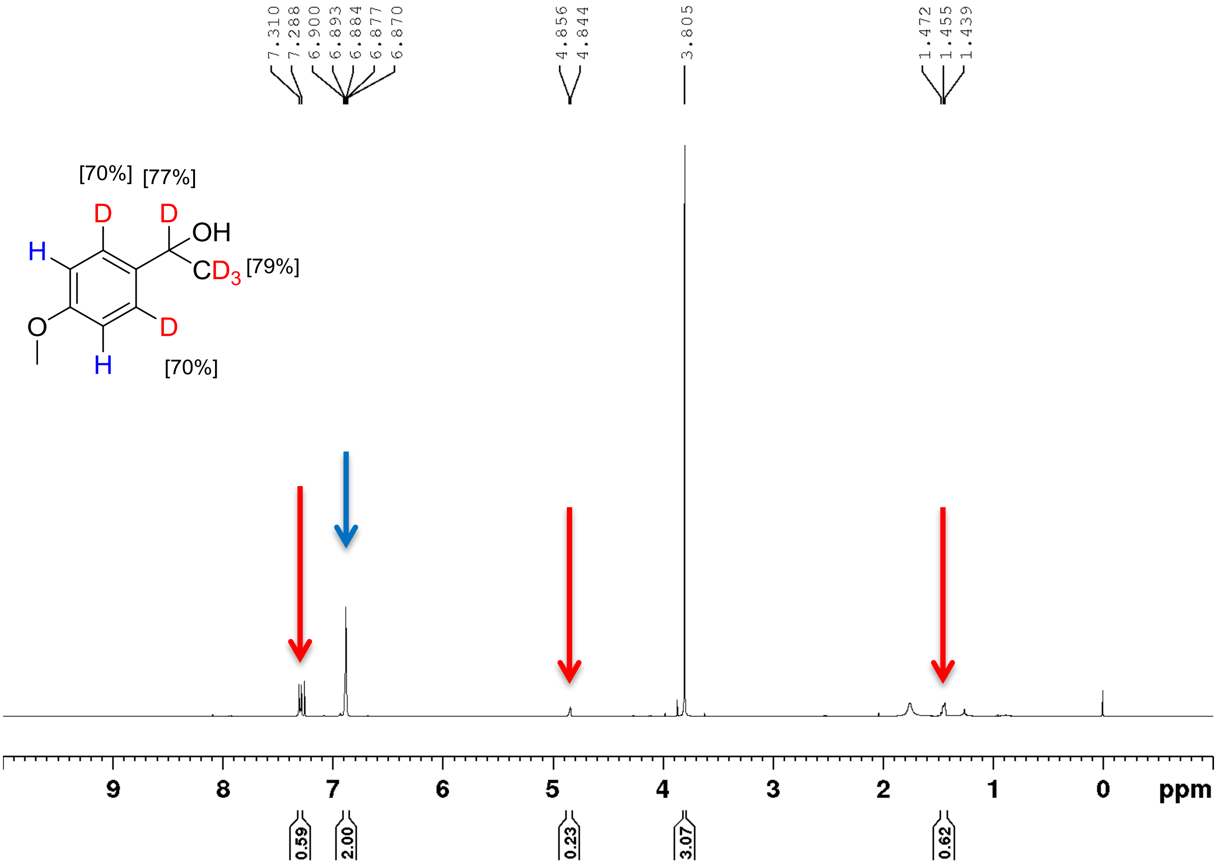

^{1}H-NMR (400 MHz, $CDCl_3$) Spectra of deuterated compound **16** following the KOD/Zn procedure for 62 h: Yield: >99%

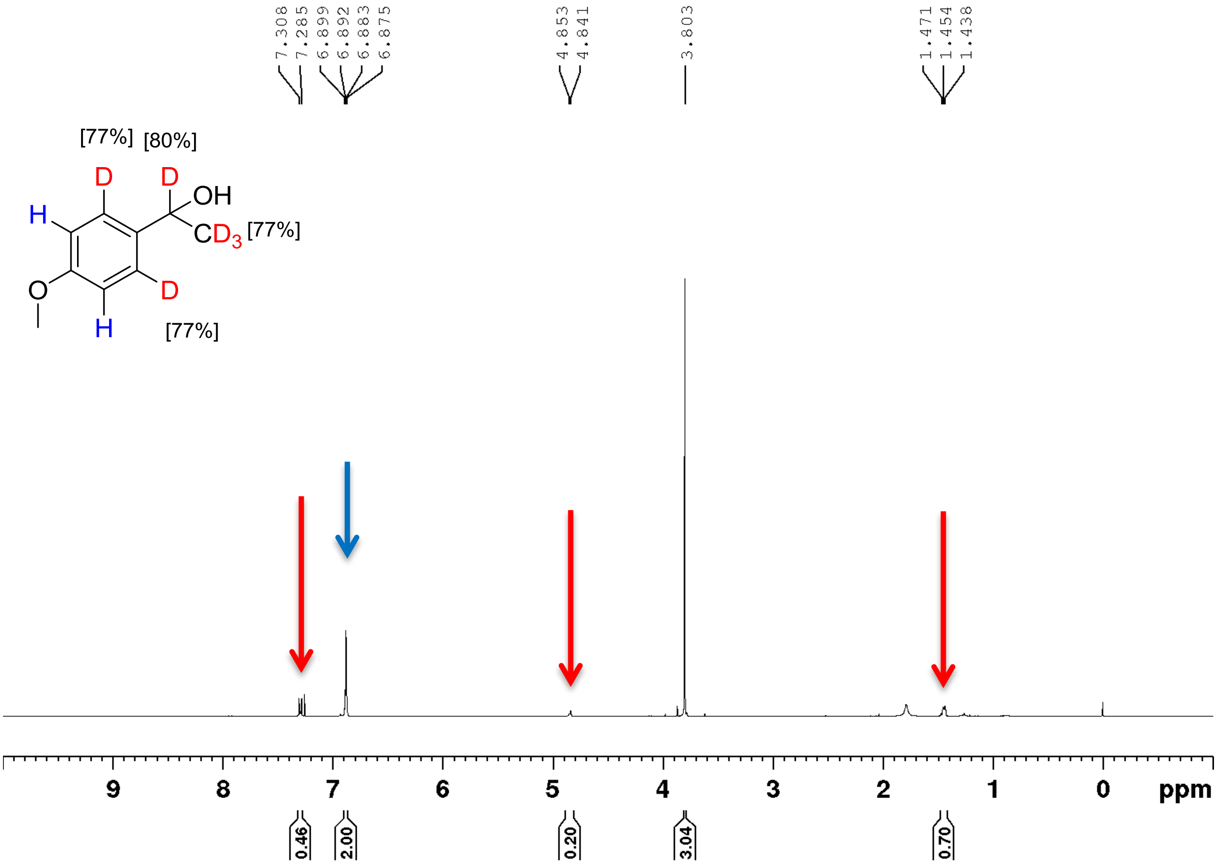

3.5.7 2-Phenylpyridine 20

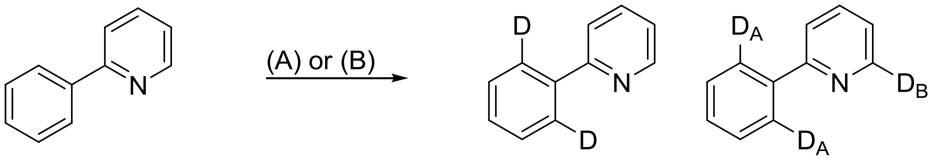

I II

	t	D_A	D_B	Yield
cond. (A)	16 h	84%	n.o.	>99%
	62 h	-	-	-
cond. (C)	16 h	71%	n.o.	>99%
cond. (B)	16 h	67%	20%	>99%

^{1}H-NMR (400 MHz, $CDCl_3$) Spectra of pure compound **20**:

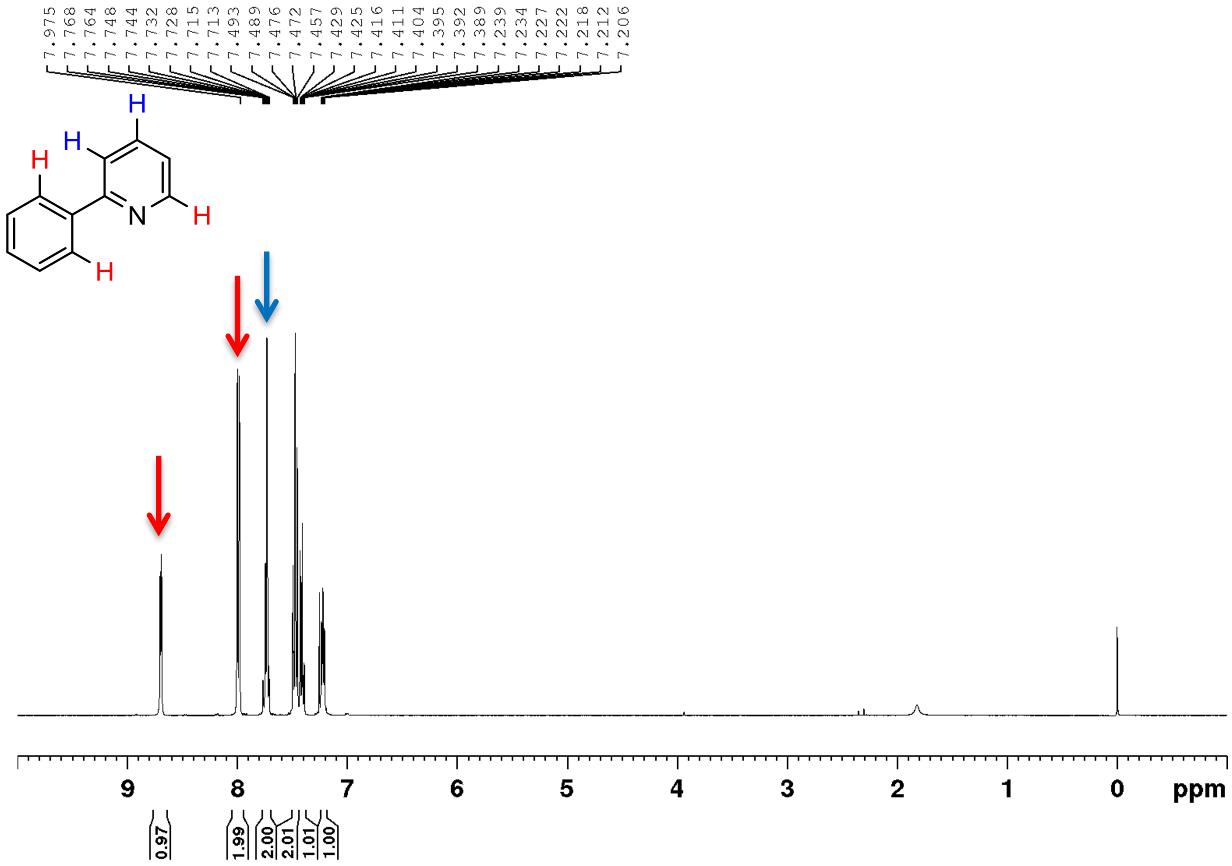

Enlargement of relevant area:

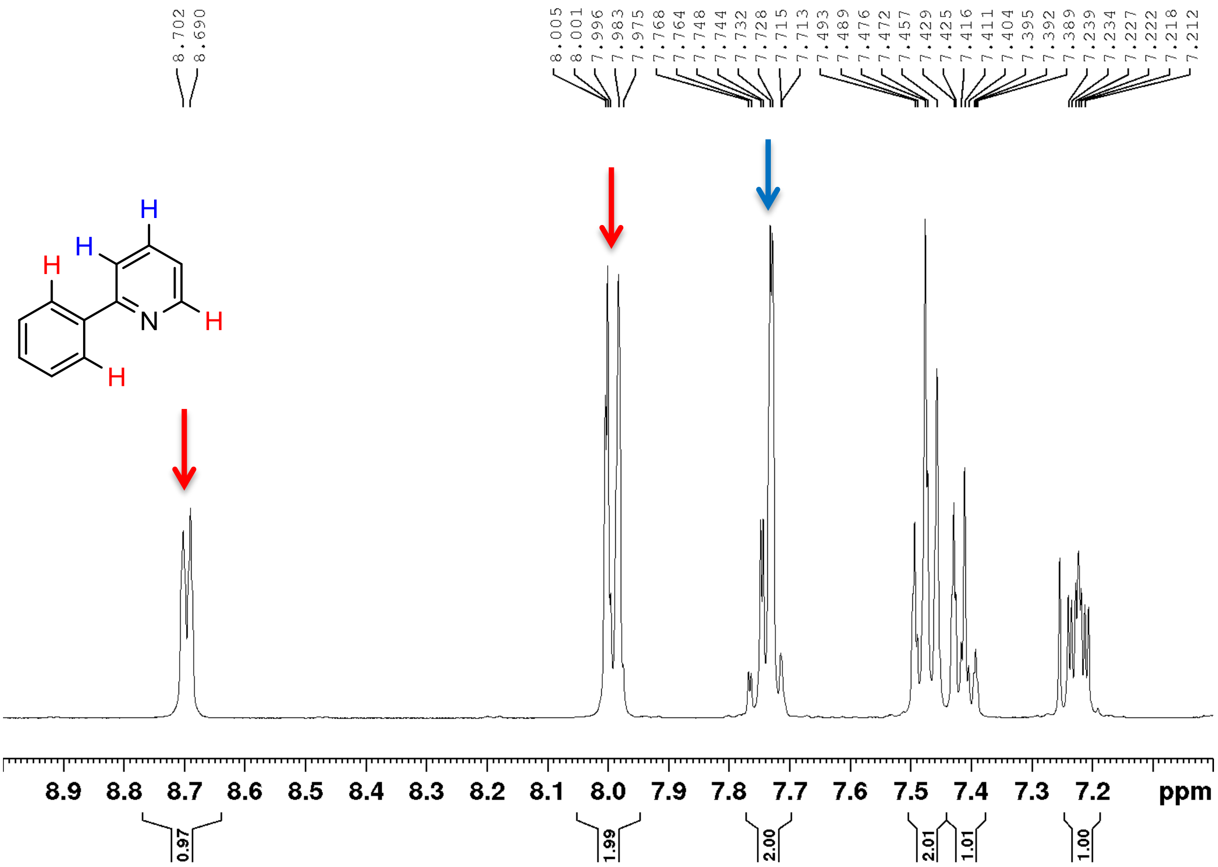

^{1}H-NMR (400 MHz, CD_2Cl_2) Spectra of pure compound **20**:

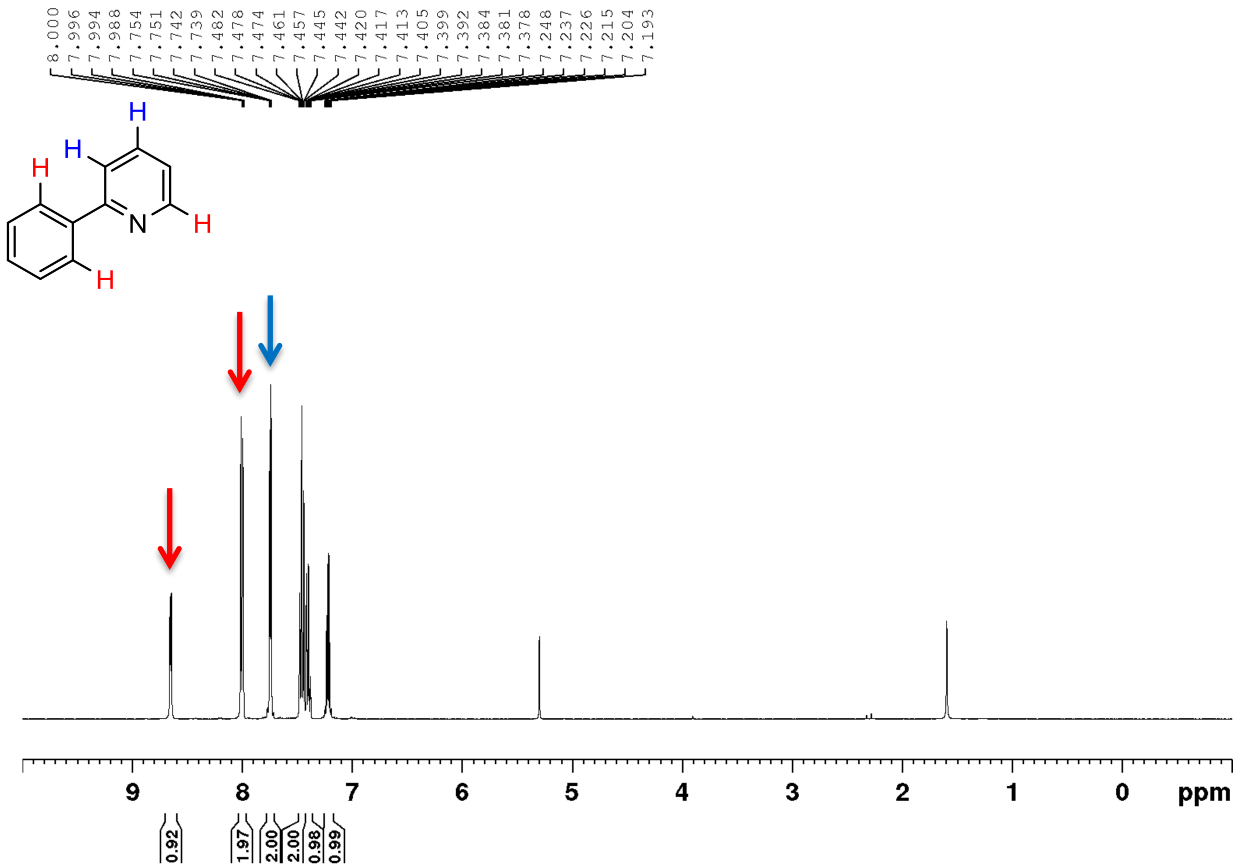

Enlargement of relevant area:

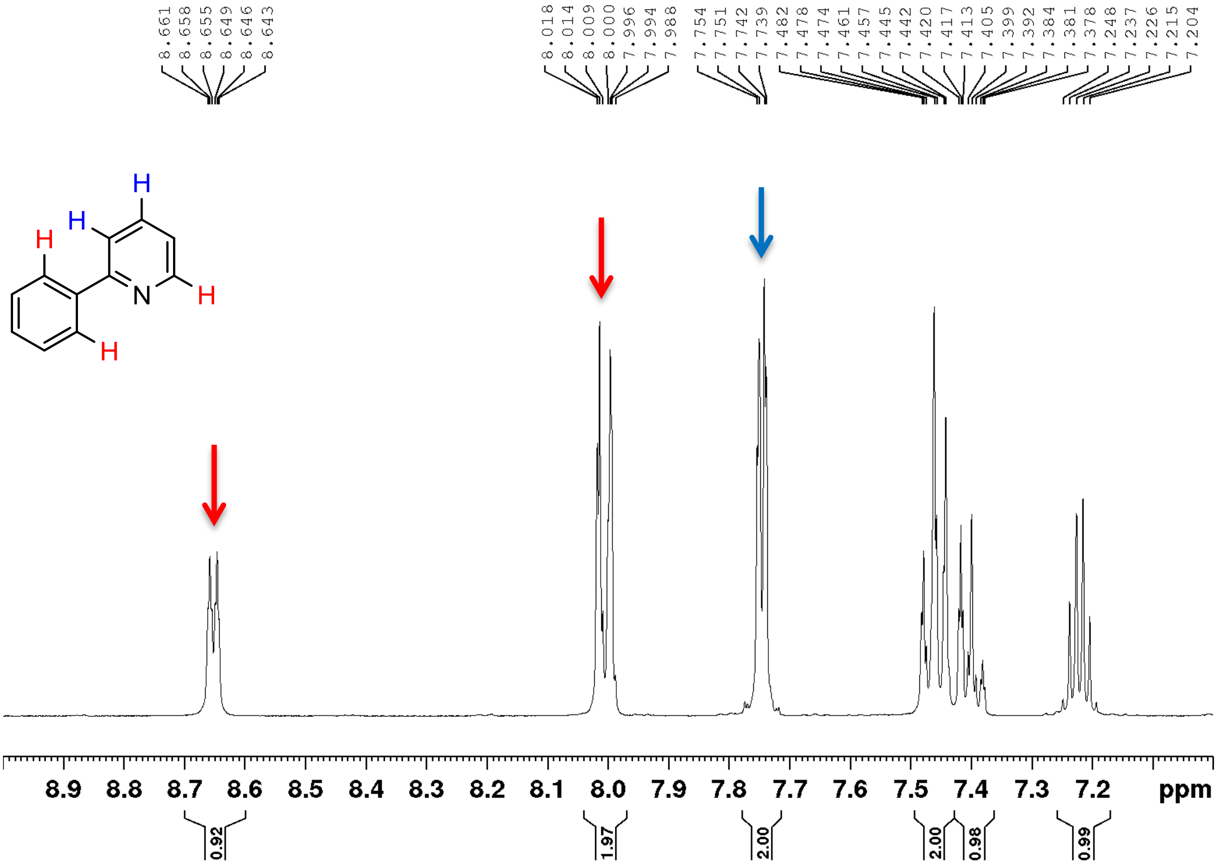

^{1}H-NMR (400 MHz, $CDCl_3$, mesitylene) Spectra of deuterated compound **20** following the KOD procedure for 16 h: Yield: >99%

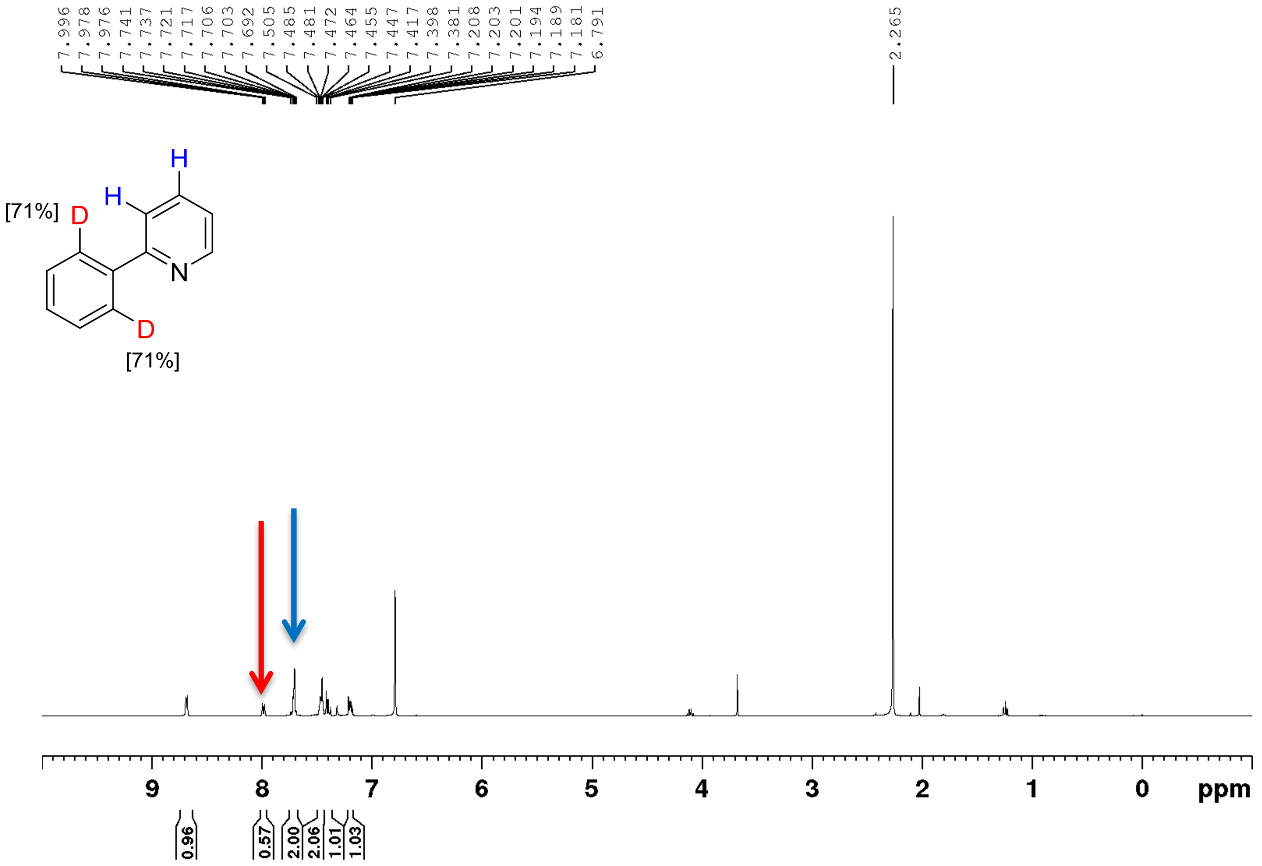

Enlargement of relevant area:

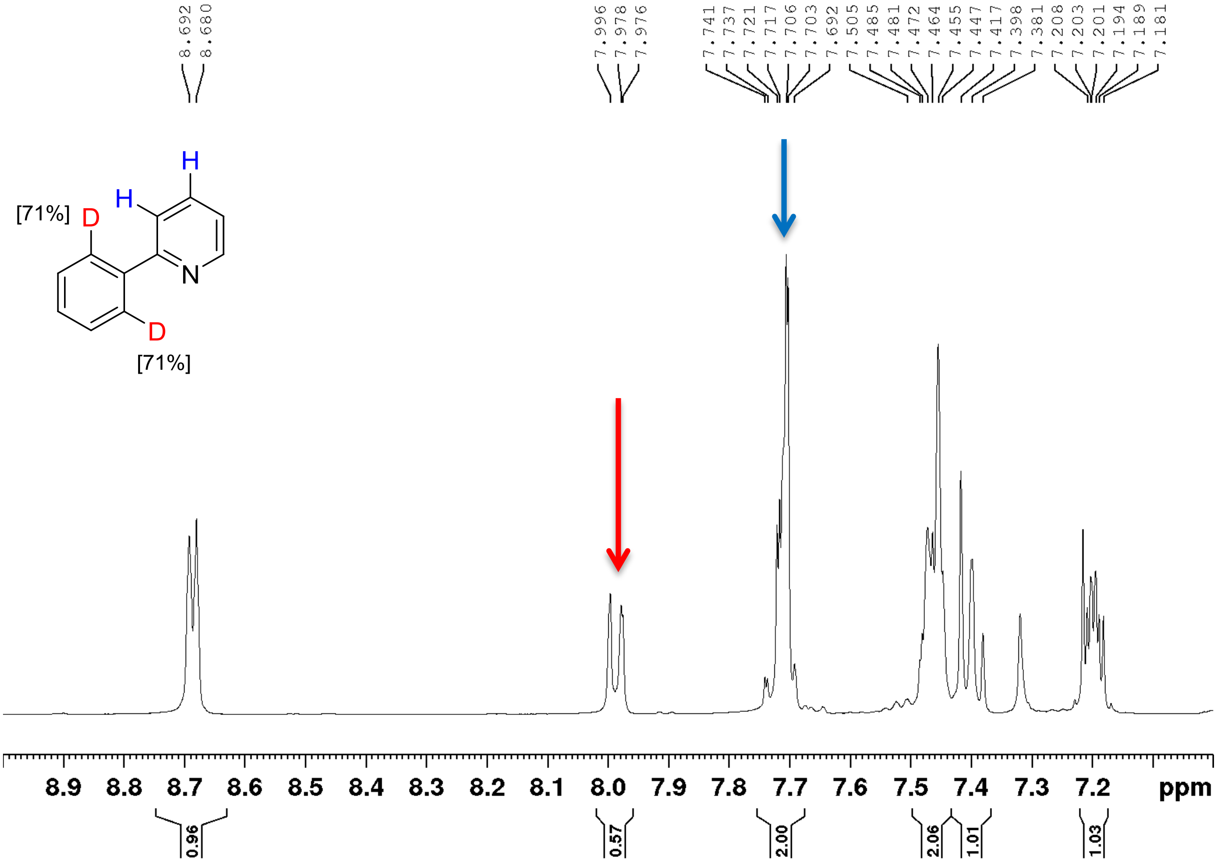

^{1}H-NMR (400 MHz, CD_2Cl_2, nitromethane) Spectra of deuterated compound **20** following the KOD/Zn procedure for 16 h: Yield: >99%

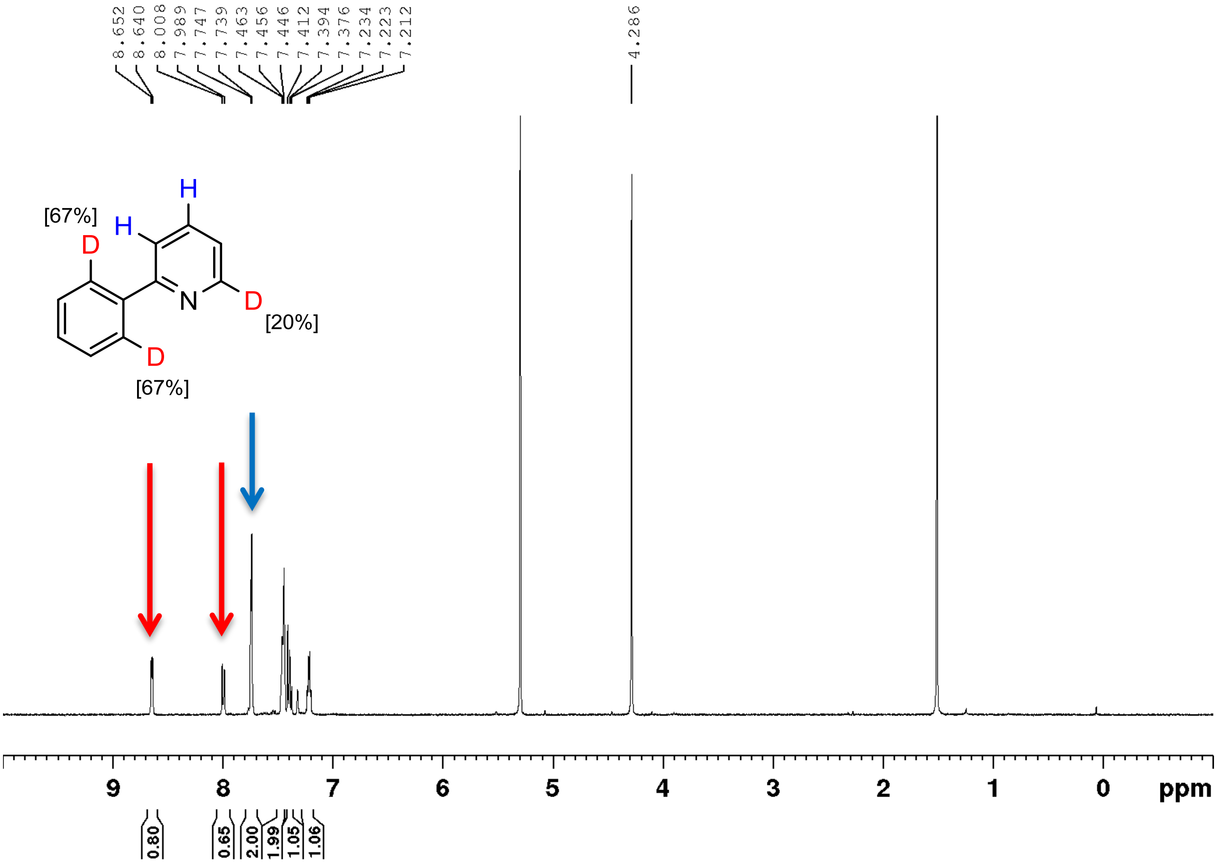

Enlargement of relevant area:

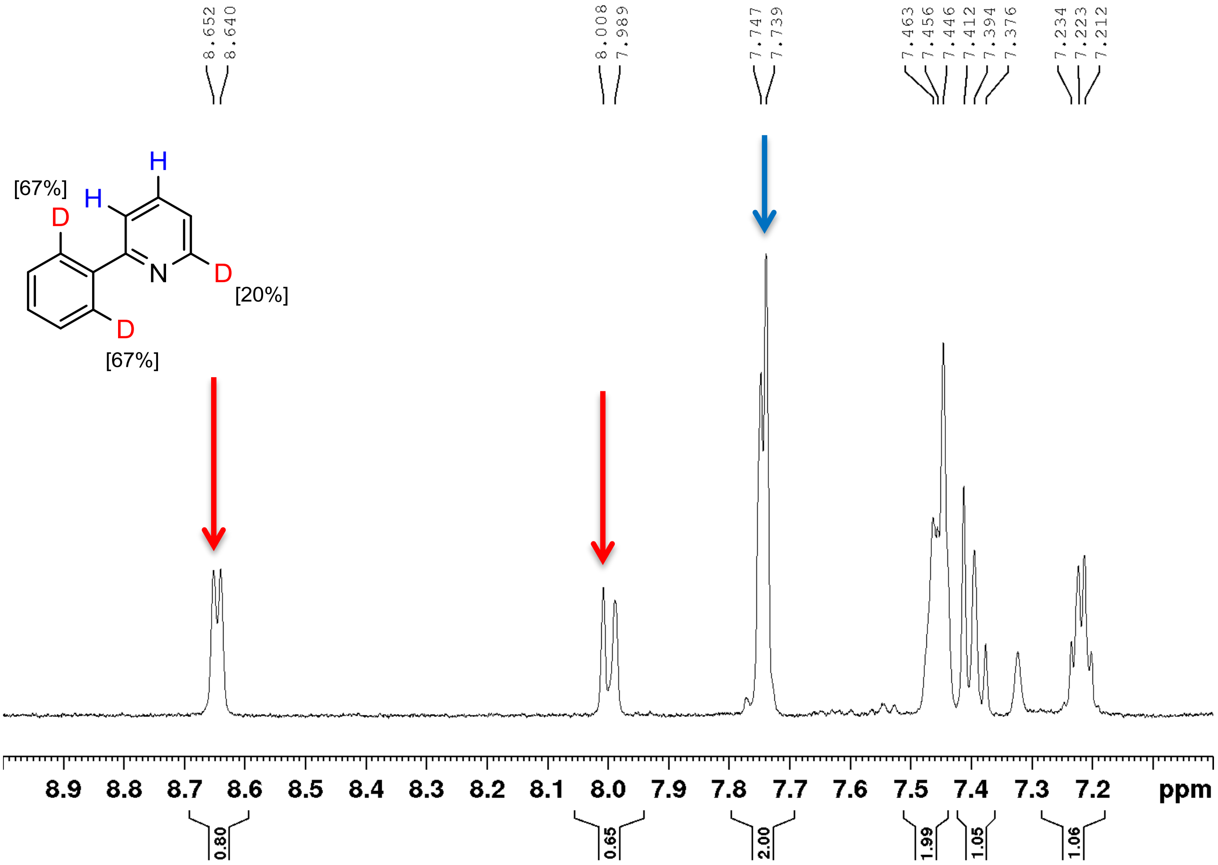

^{1}H-NMR (400 MHz, CD_2Cl_2, nitromethane) Spectra of deuterated compound **20** following the CuI procedure for 16 h: Yield: >99%

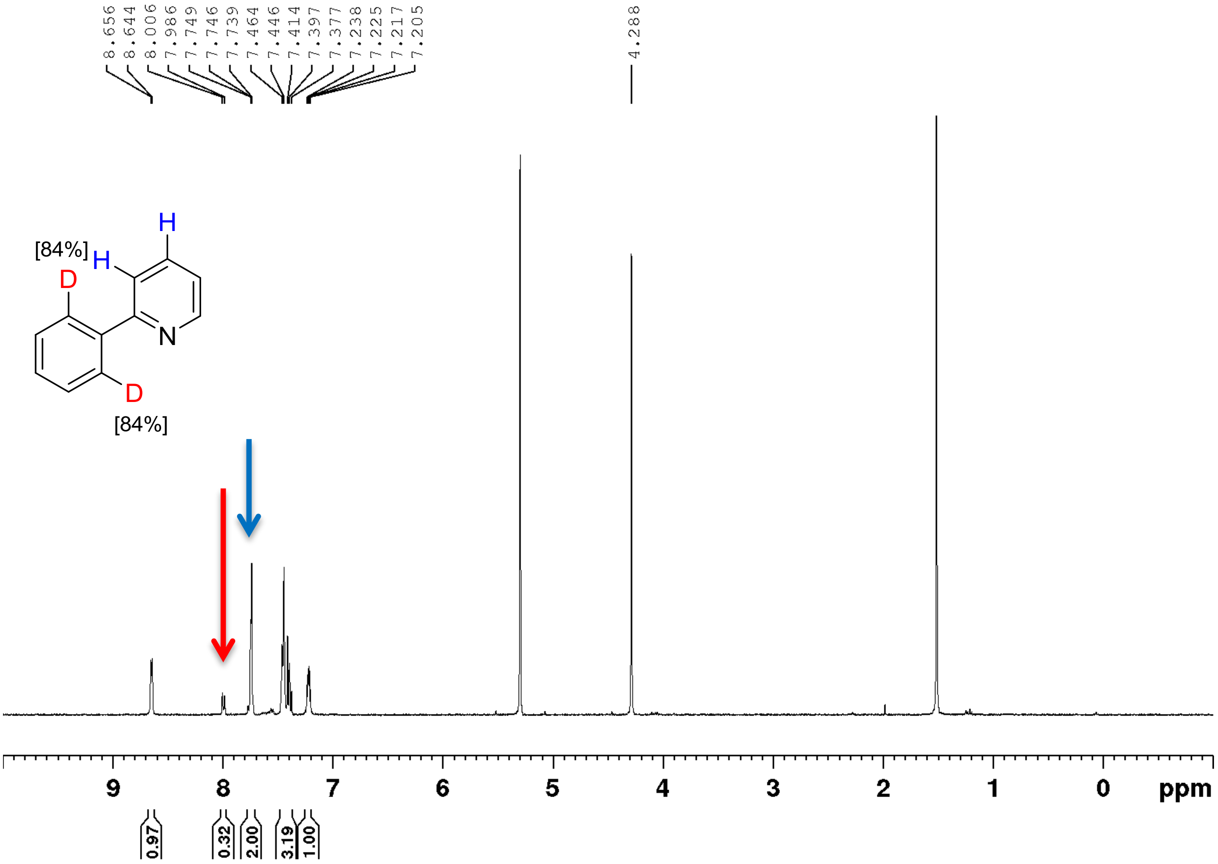

Enlargement of relevant area:

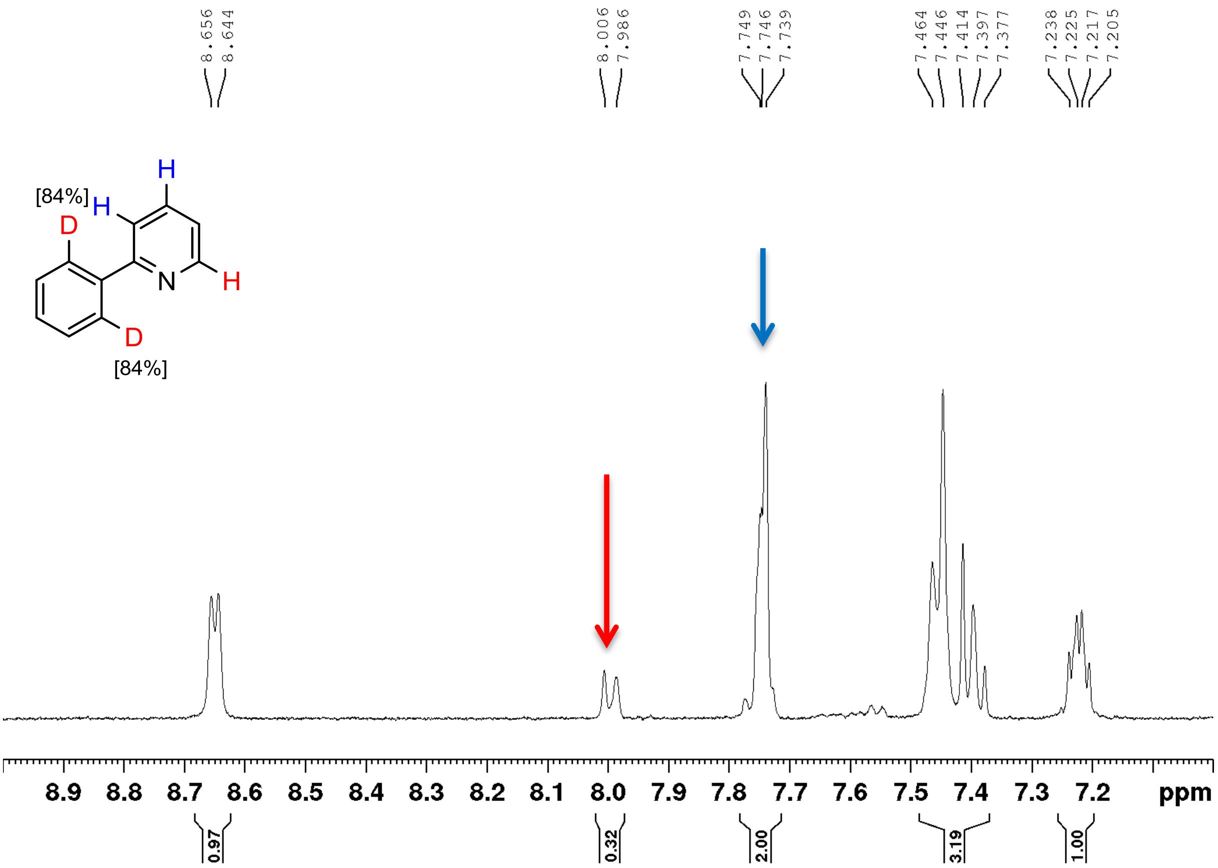

3.5.8 2-Phenylimidazole 21

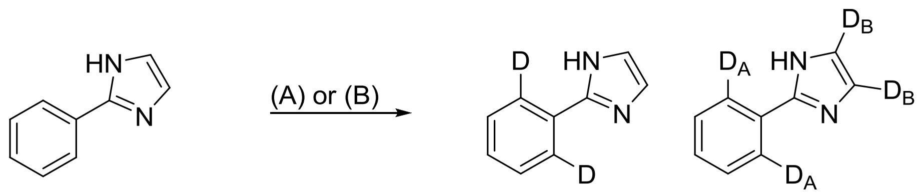

I **II**

	t	D_A	D_B	Yield
cond. (A)	16 h	71%	n.o.	41%
	62 h	-	-	-
cond. (C)	16 h	76%	64%	>99%
cond. (B)	16 h	-	-	-

2-Phenylimidazole 21 (I and II)

^{1}H-NMR (300 MHz, $CDCl_3$) Spectra of pure compound **21**:

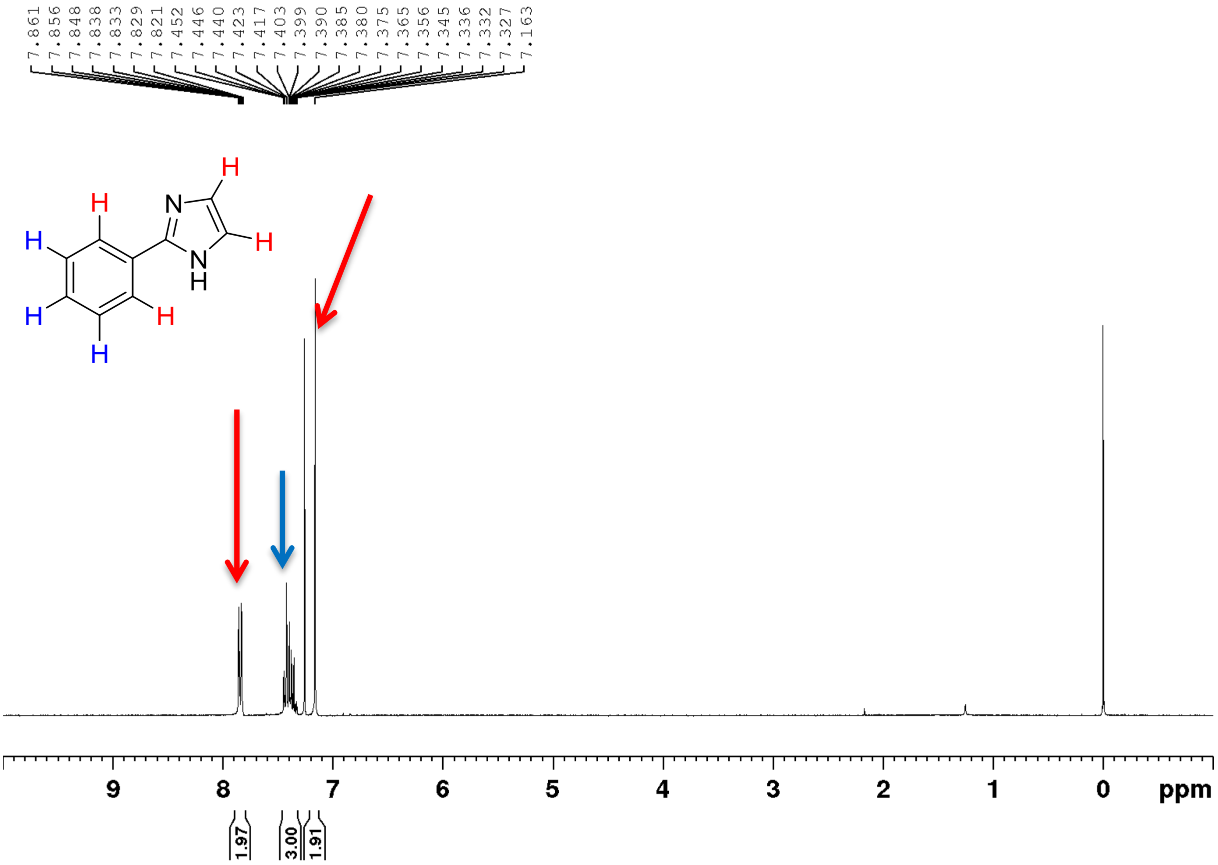

^{1}H-NMR (400 Hz, $CDCl_3$, nitromethane) Spectra of deuterated compound **21** following the KOD procedure for 16 h: Yield: >99%

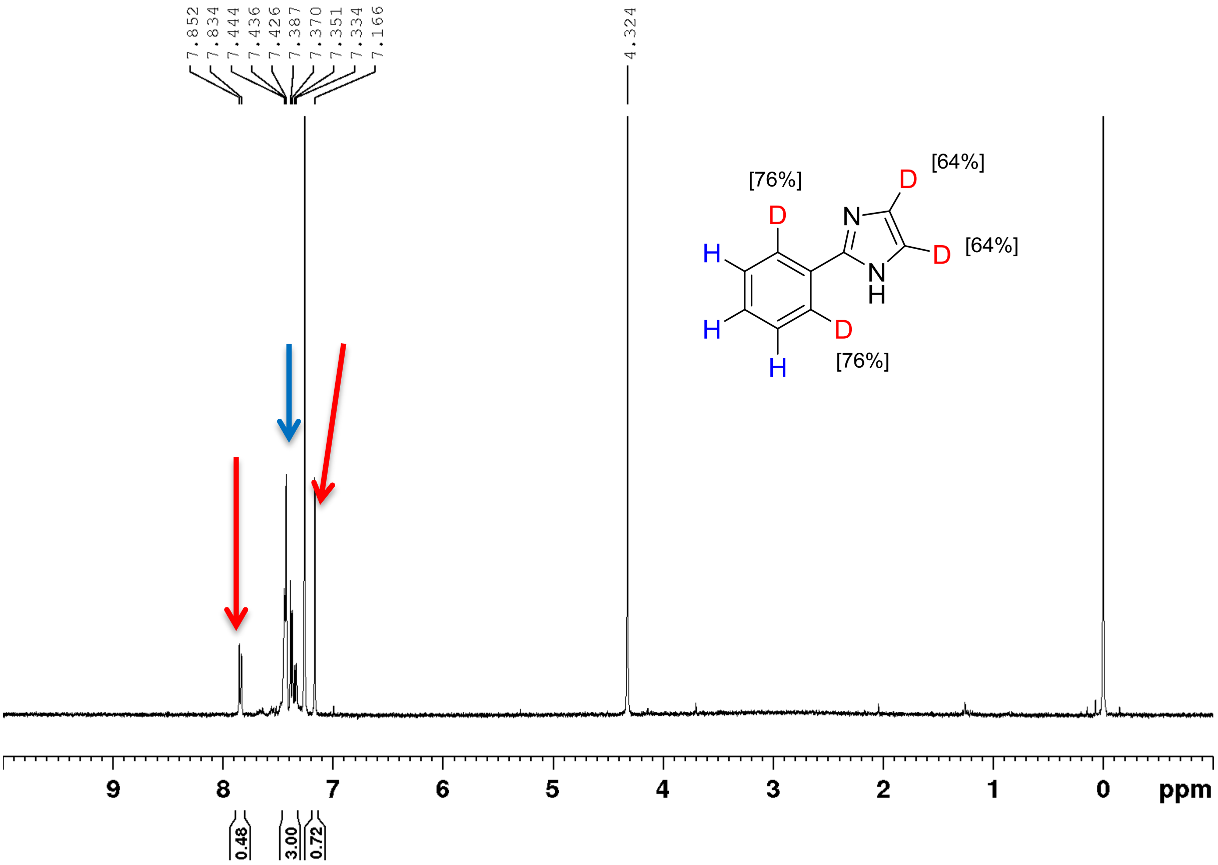

^{1}H-NMR (400 Hz, $CDCl_3$, nitromethane) Spectra of deuterated compound **21** following the CuI procedure for 16 h: Yield: 41%

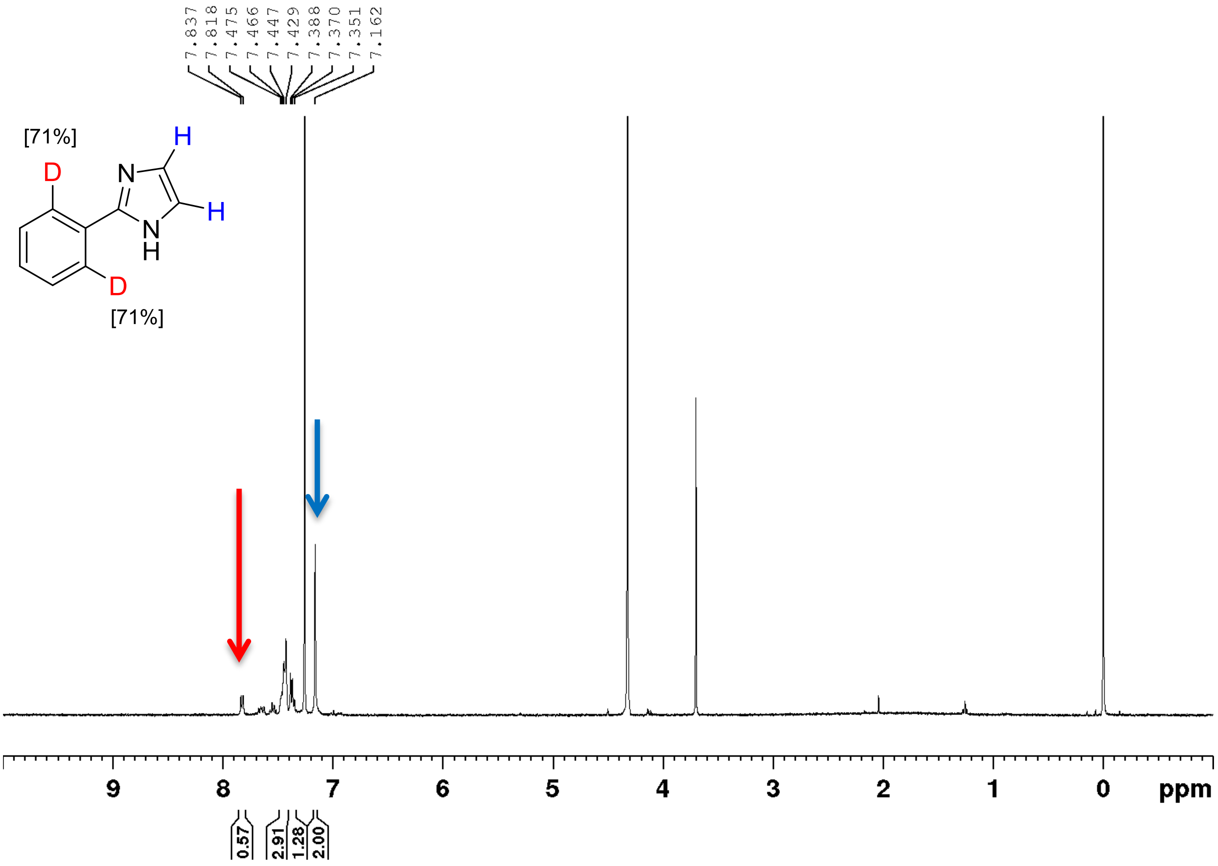

Enlargement of relevant area:

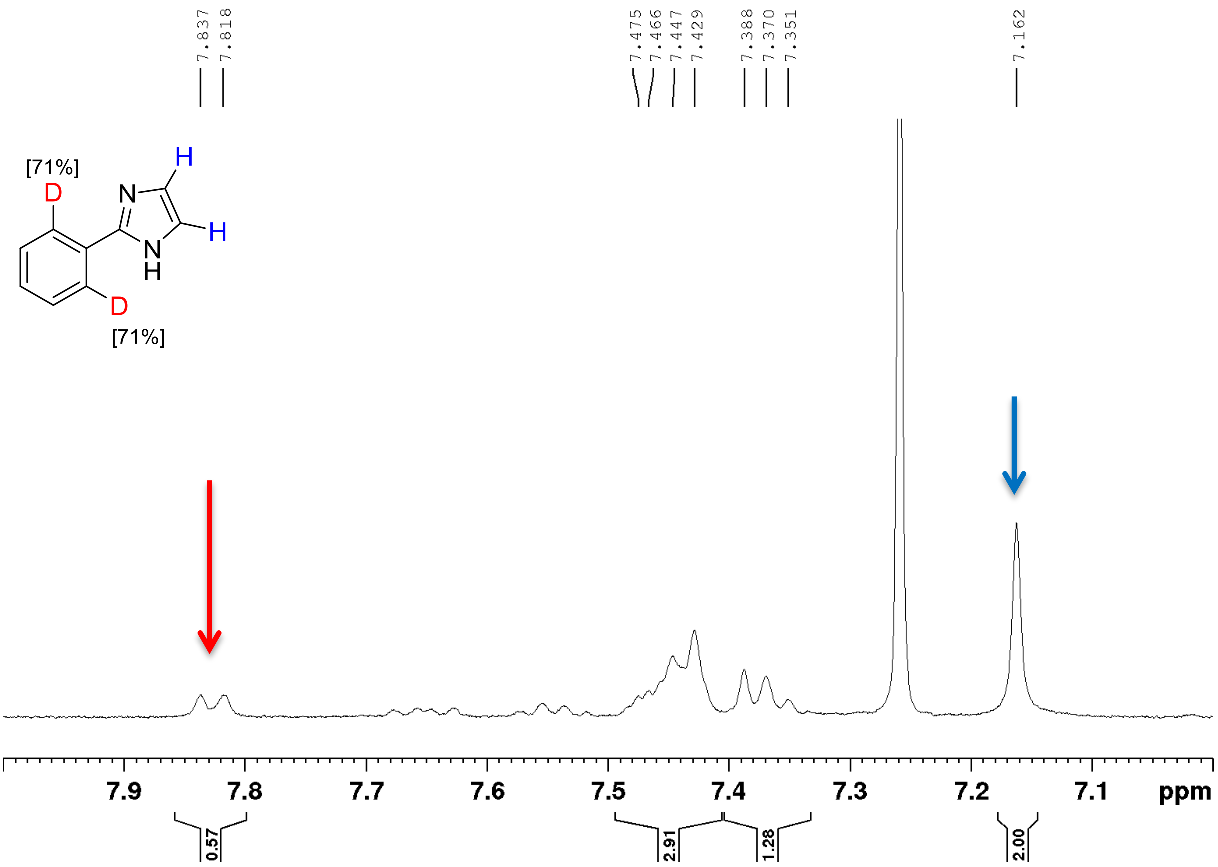

3.5.9 *N*-methyl-2-phenyl-1H-imidazole 22

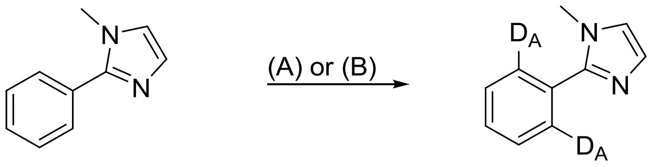

	t	D_A	Yield
cond. (A)	16 h	83%	50%
	62 h	-	-
cond. (C)	16 h	36%	84%
cond. (B)	16 h	-	-

^{1}H-NMR (300 MHz, $CDCl_3$) Spectra of pure compound **22**:

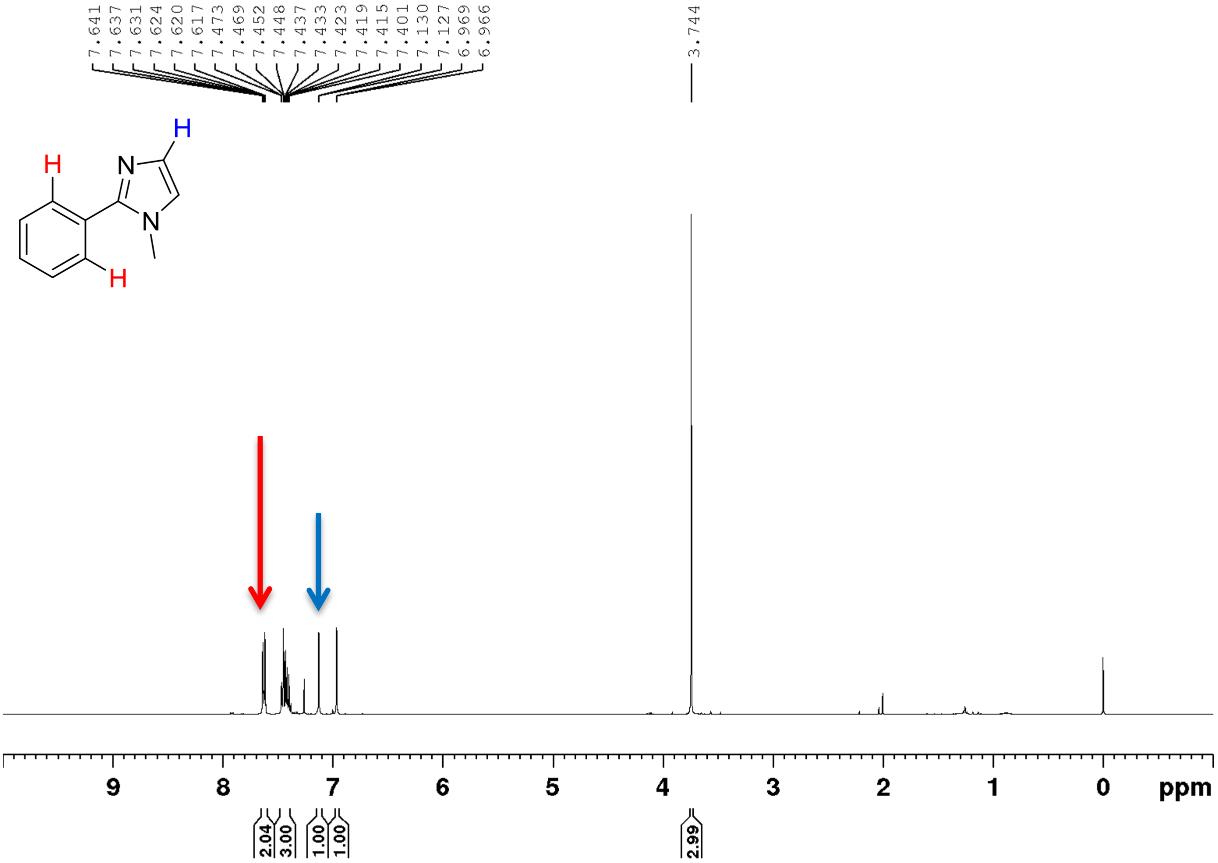

^{1}H-NMR (400 MHz, $CDCl_3$, mesitylene) Spectra of deuterated compound **22** following the KOD procedure for 16 h: Yield: 84%

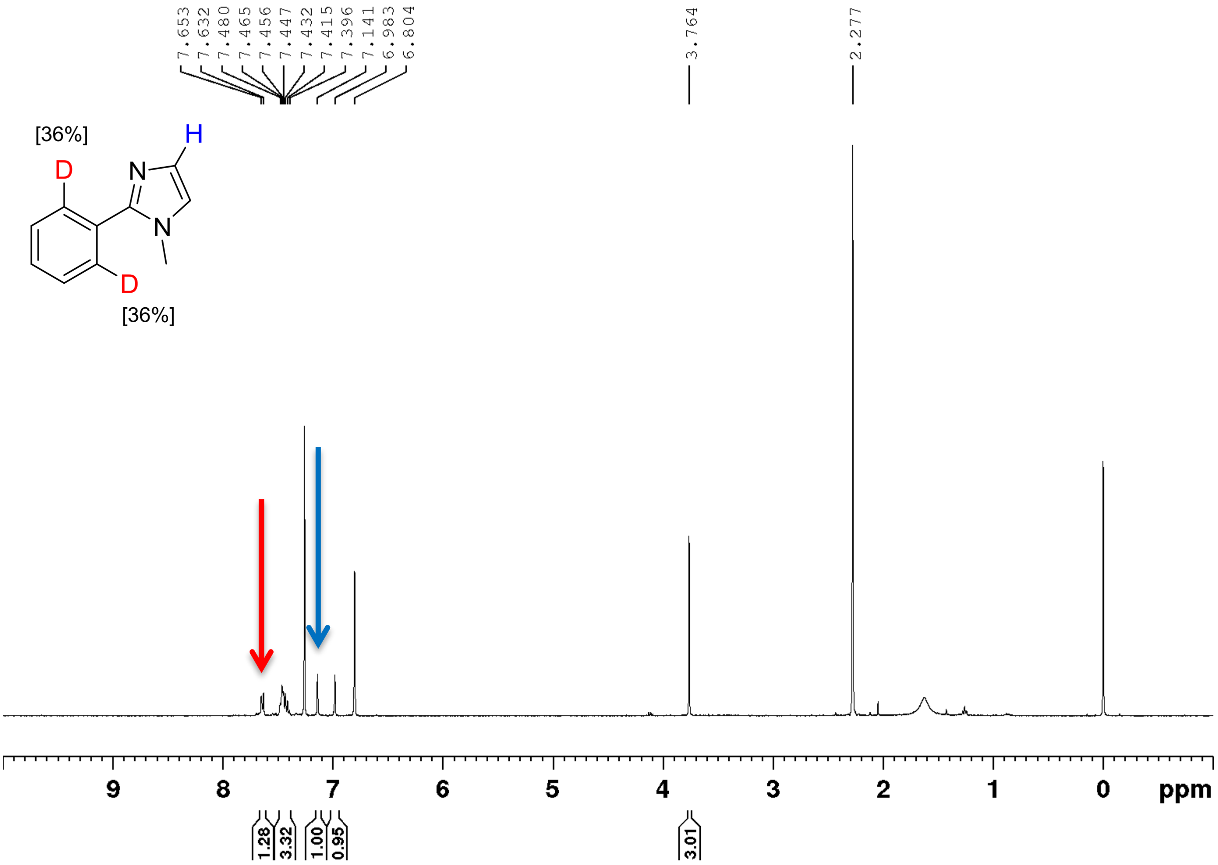

^{1}H-NMR (400 MHz, $CDCl_3$, mesitylene) Spectra of deuterated compound **22** following the CuI procedure for 16 h: Yield: 50%

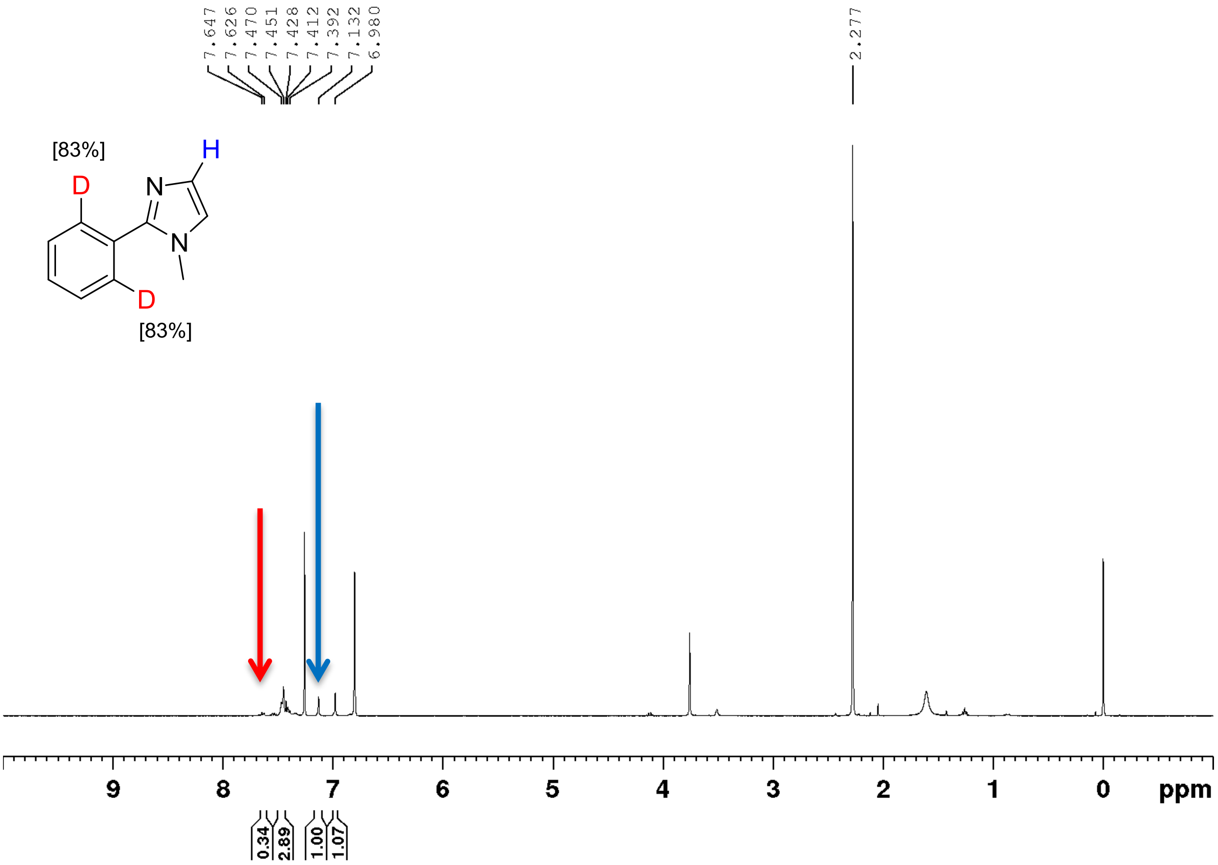

Enlargement of relevant area:

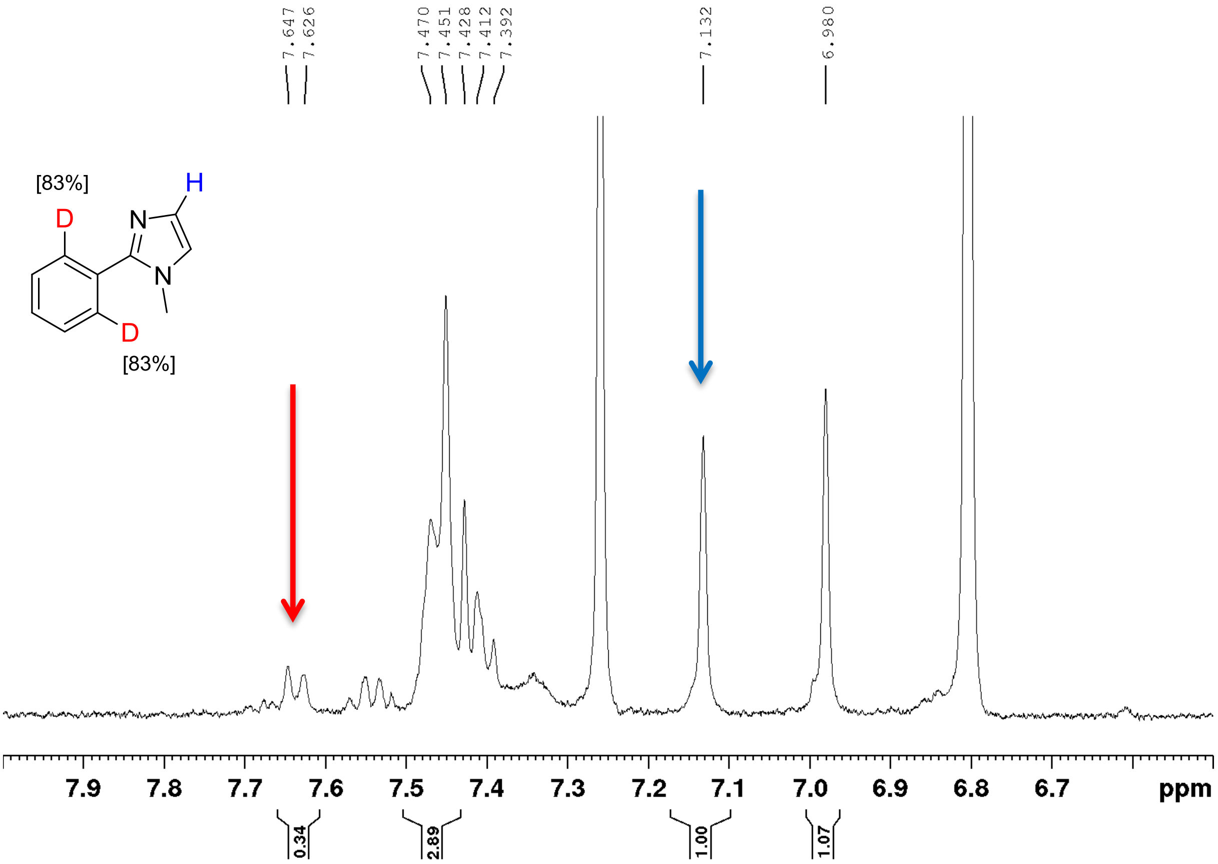

3.5.10 2-phenyl-2-imidazoline 23

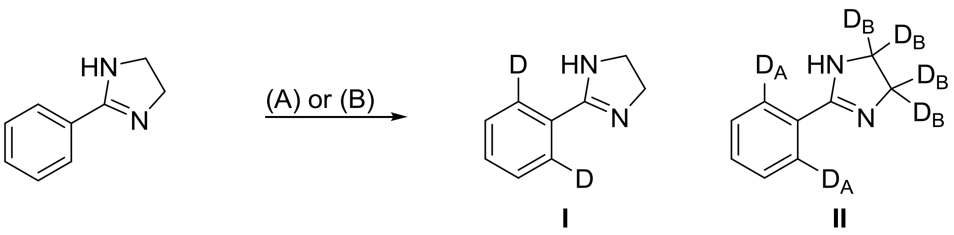

	t	D_A	D_B	Yield
cond. (A)	16 h	57%	25%	>99%
	62 h	-	-	-
cond. (C)	16 h	72%	n.o.	97%
cond. (B)	16 h	-	-	-

2-phenyl-2-imidazoline 23 (I and II)

^{1}H-NMR (400 MHz, THF-d_8) Spectra of pure compound **23**:

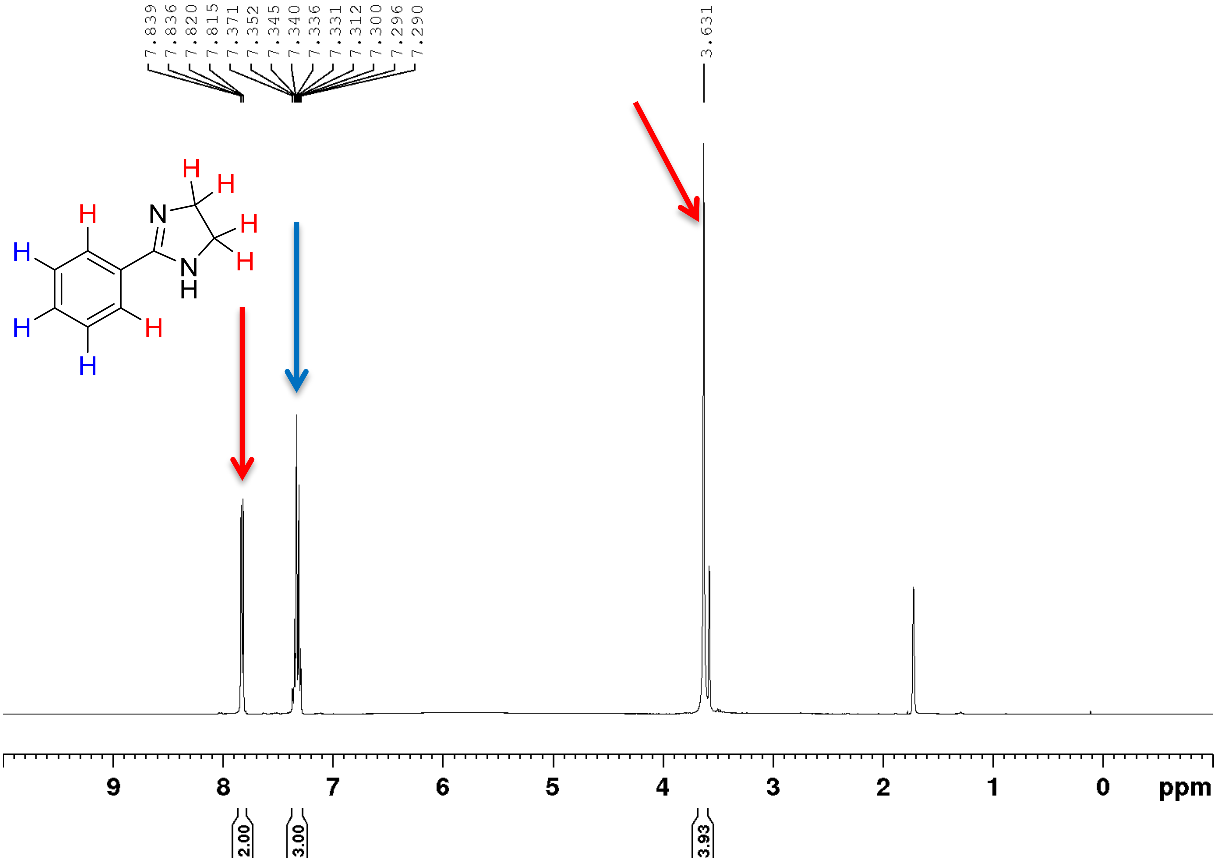

^{1}H-NMR (400 Hz, CD_2Cl_2, mesitylene) Spectra of deuterated compound **23** following the KOD procedure for 16 h: Yield: 97%

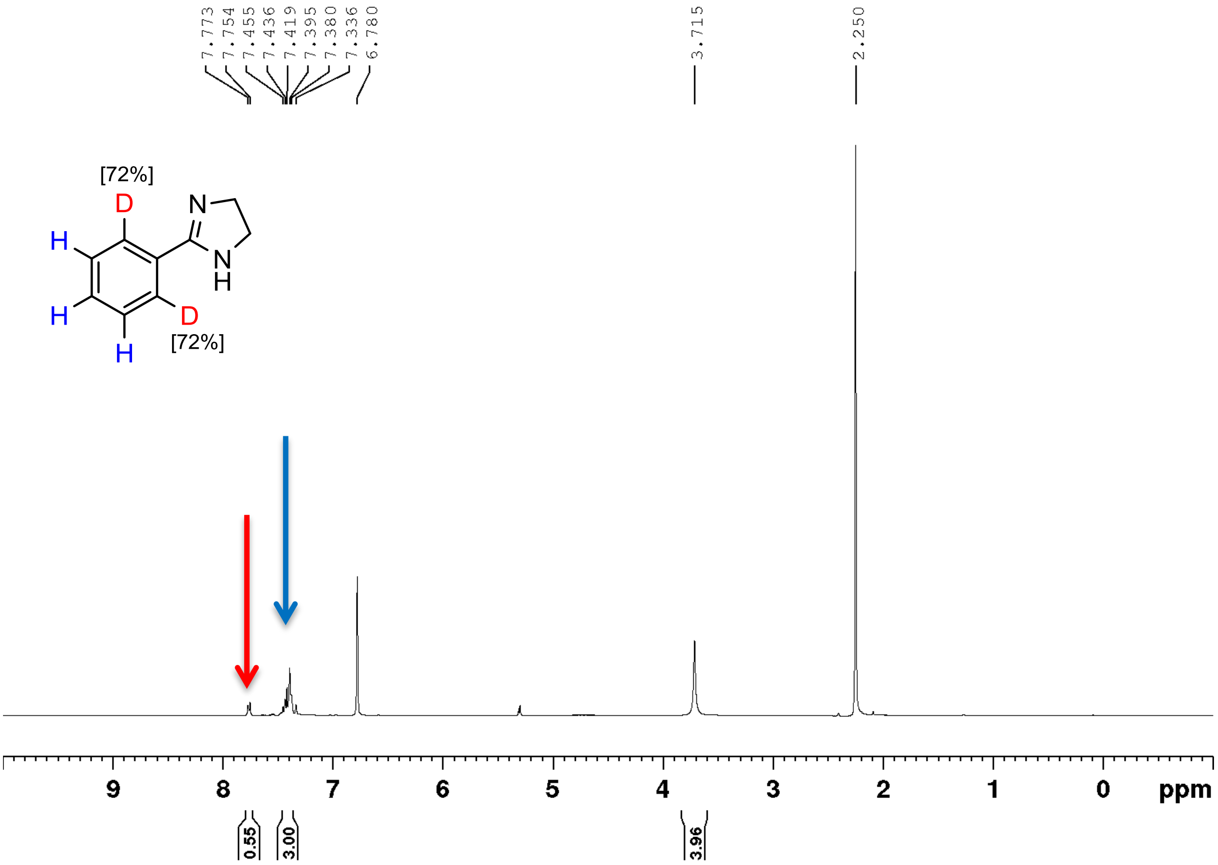

^{1}H-NMR (400 Hz, CD_2Cl_2, mesitylene) Spectra of deuterated compound **23** following the CuI procedure for 16 h: Yield: >99%

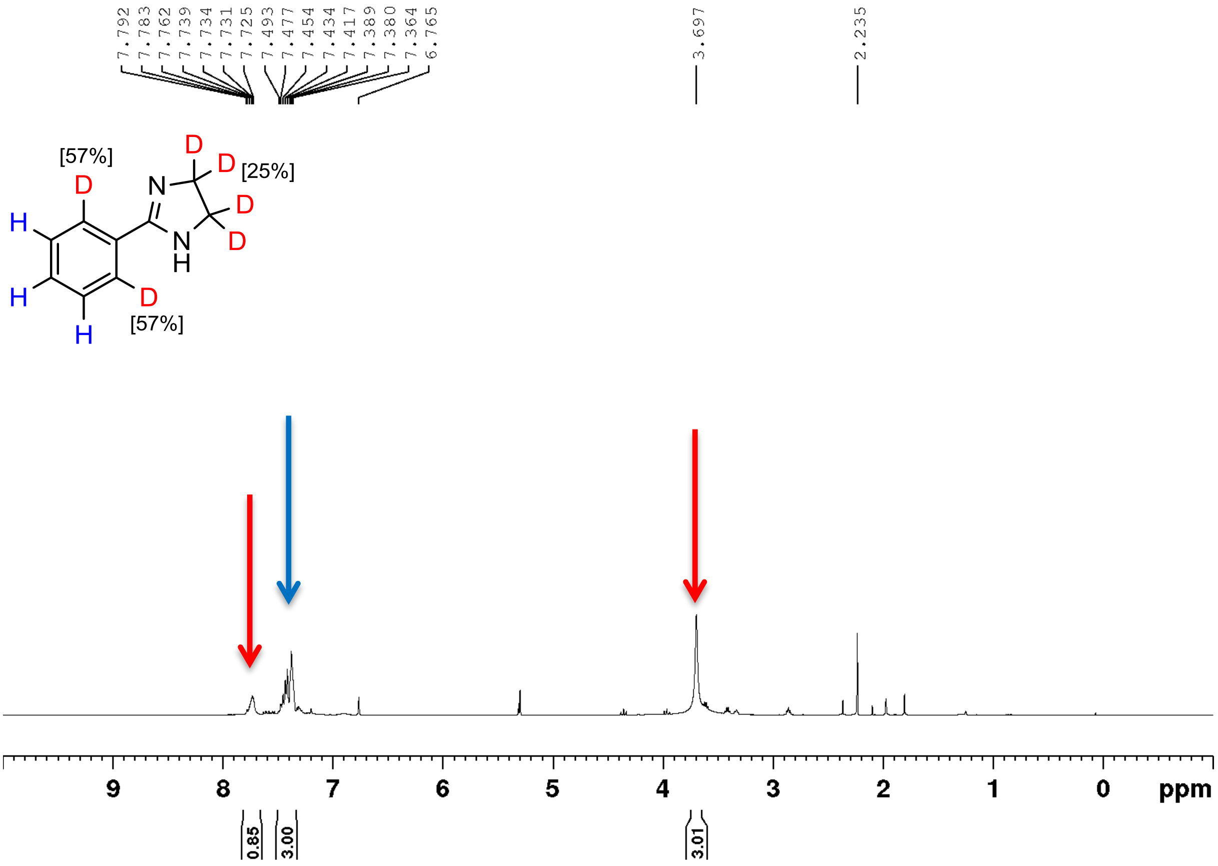

3.5.11 4,5-dihydro-1-methyl-2-phenyl-1H-imidazole 24

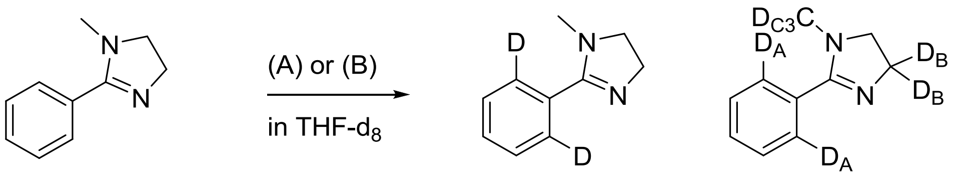

I | II

	t	D_A	D_B	D_C	Yield
cond. (A)	16 h	46%	79%	43%	83%
	62 h	-	-		-
cond. (C)	16 h	31%	n.o.	n.o.	74%
cond. (B)	16 h	-	-		-

4,5-dihydro-1-methyl-2-phenyl-1H-imidazole 24 (I and II)

^{1}H-NMR (300 MHz, CDCl$_3$) Spectra of pure compound **24**:

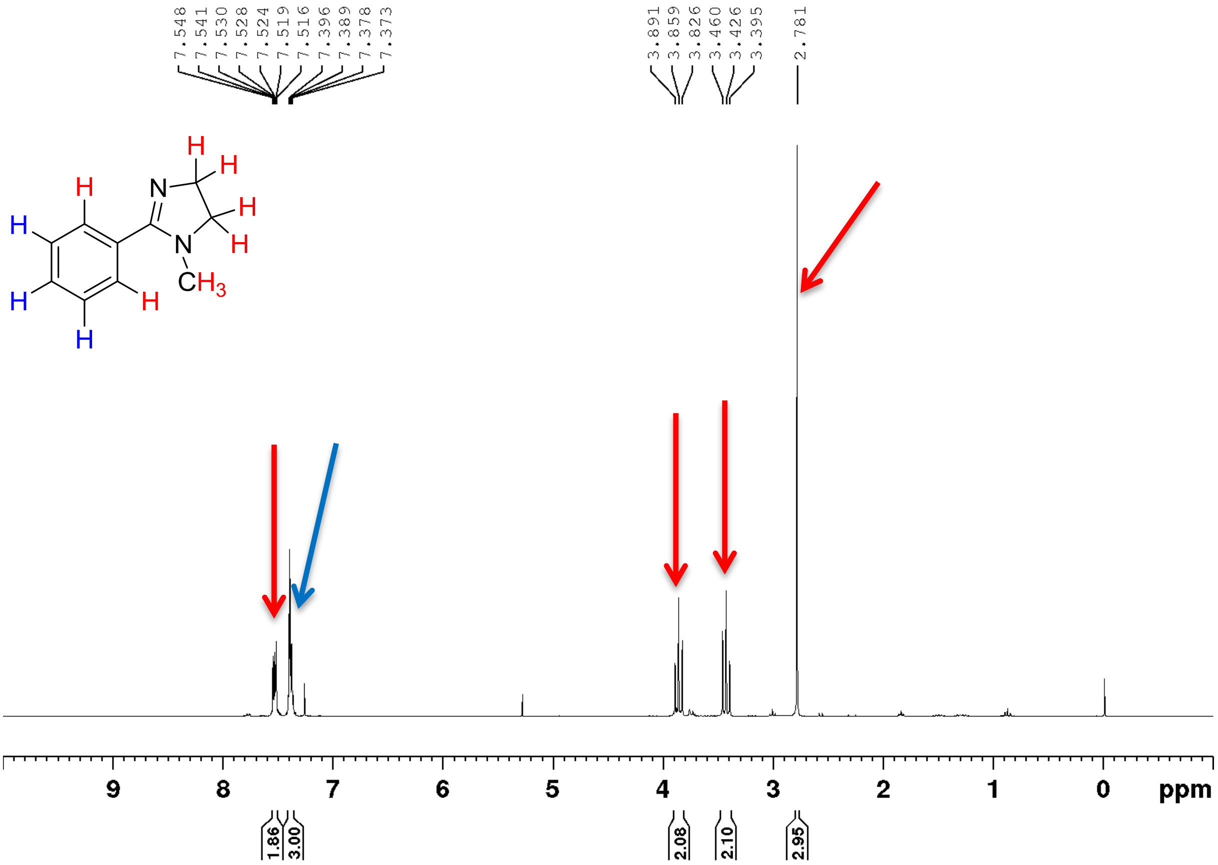

^{1}H-NMR (400 Hz, THF-d$_8$, nitromethane) Spectra of deuterated compound **24** following the KOD procedure for 16 h: Yield: 74%

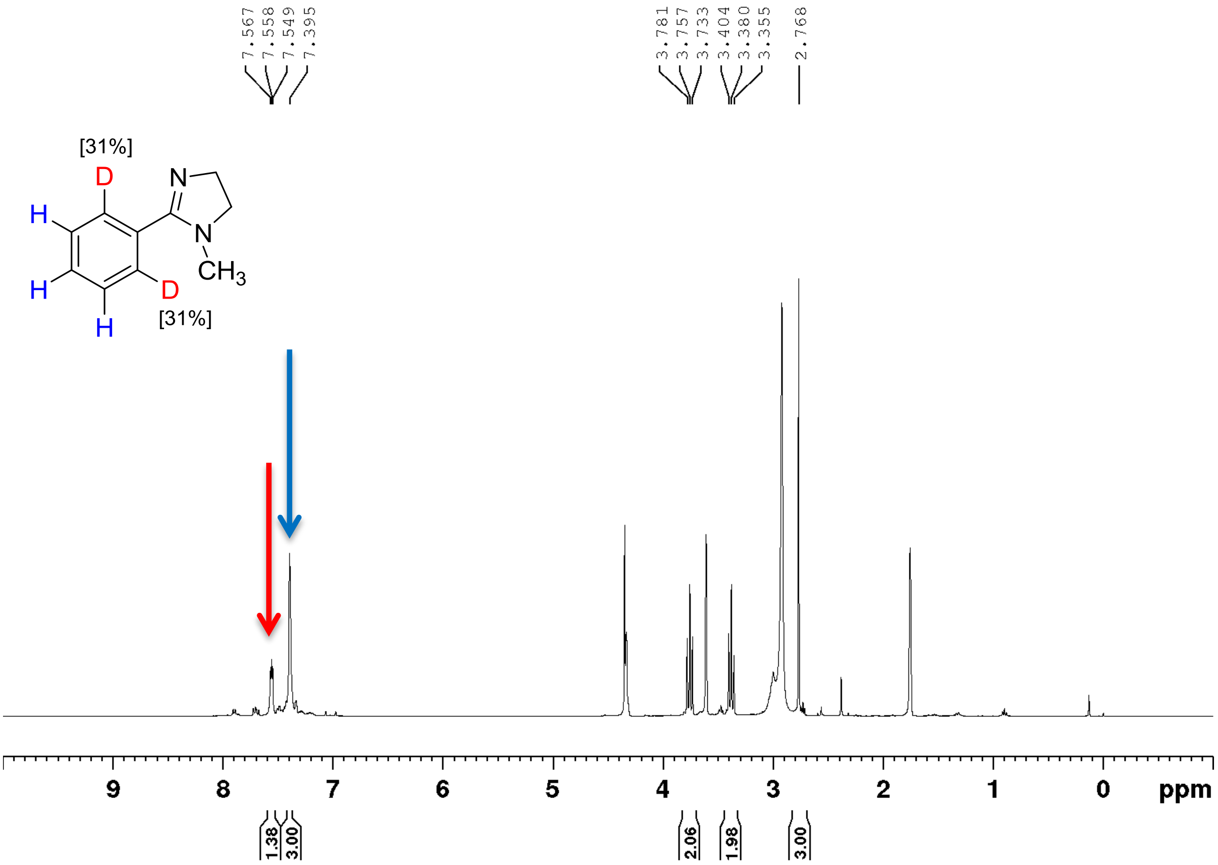

^{1}H-NMR (400 MHz, THF-d$_8$, mesitylene) Spectra of deuterated compound **24** following the CuI procedure for 16 h: Yield: 83%

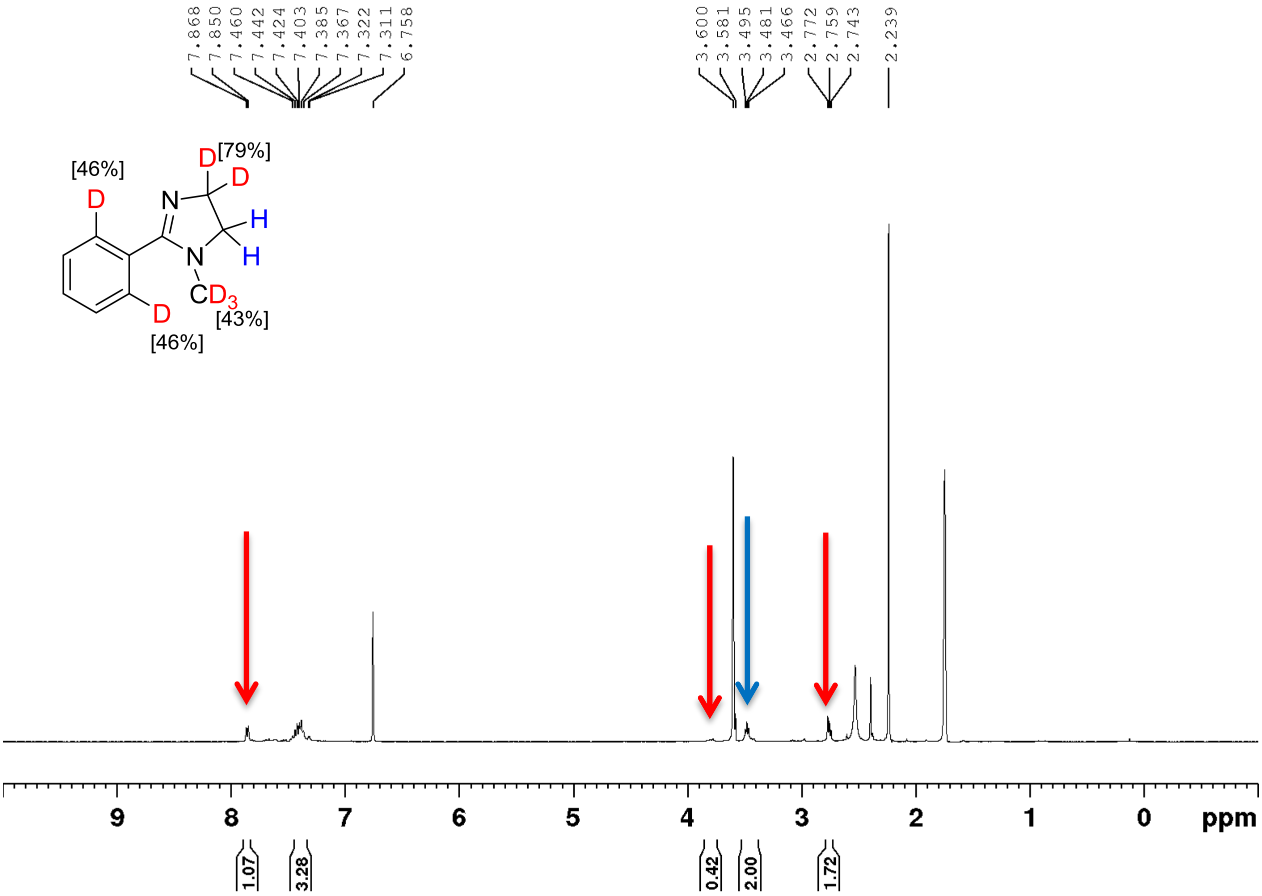

Enlargement of relevant area:

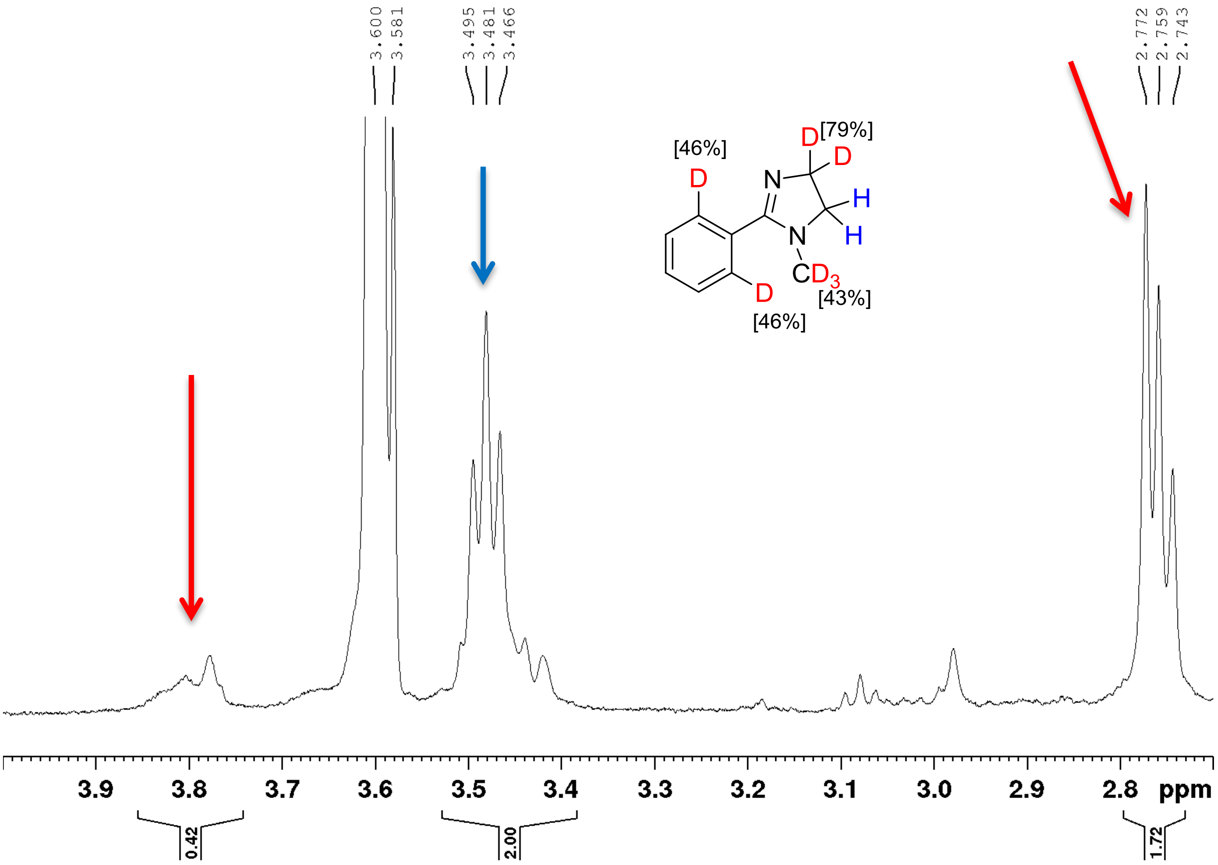

3.5.12 2-phenyl-2-oxazoline 25

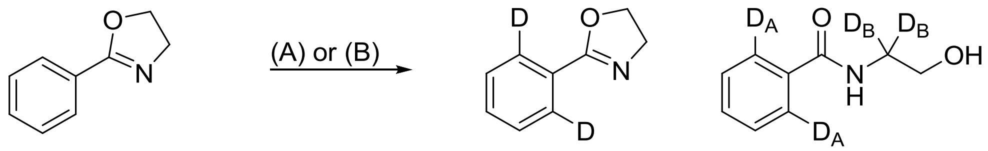

I II

	I				II		
	t	D_A	D_B	Yield	D_A	D_B	Yield
cond. (A)	16 h	-	-	-	-	-	-
	62 h	n.o.	n.o.	n.o.	74%	60%	69%
cond. (C)	16 h	85%	n.o.	>99%	n.o.	n.o.	n.o.
cond. (B)	62 h	-	-	-	-	-	-

2-phenyl-2-oxazoline 25 (I)

^{1}H-NMR (400 MHz, CD_2Cl_2) Spectra of pure compound **25**:

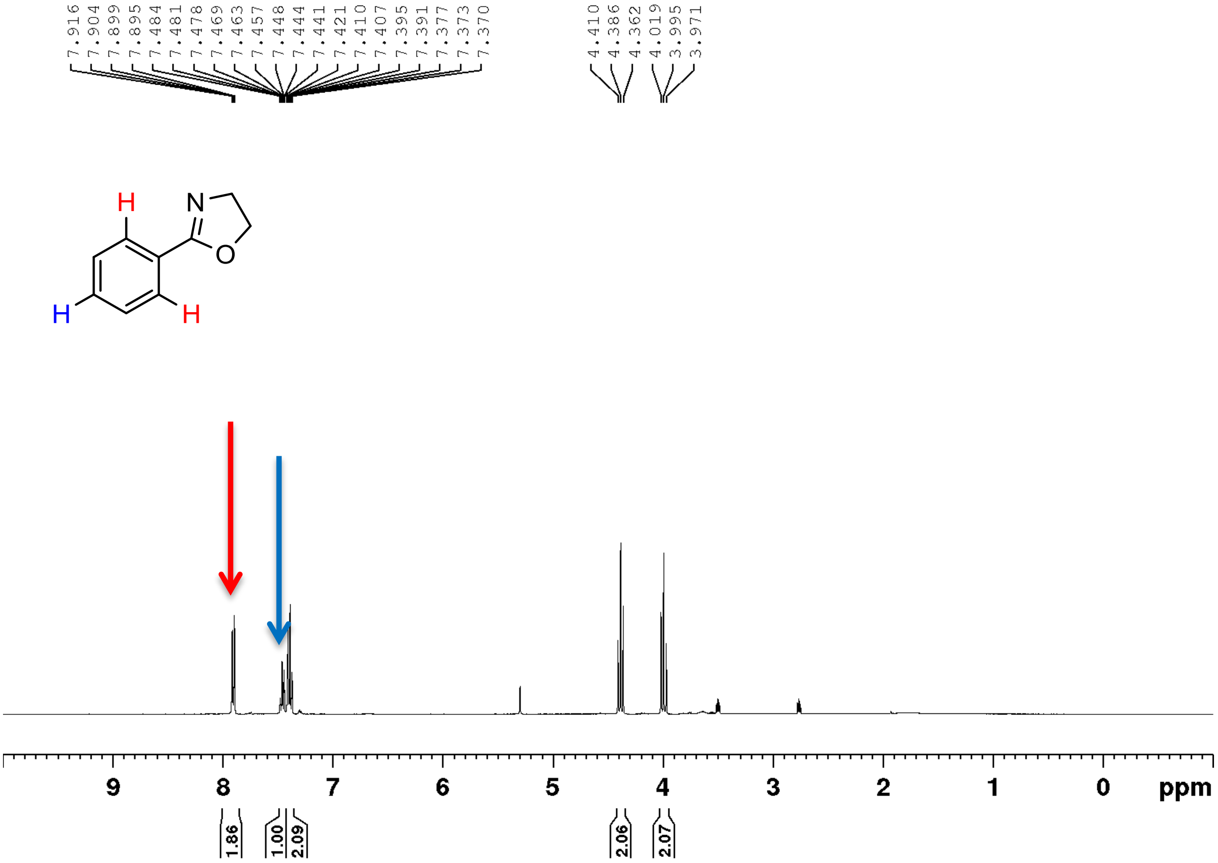

^{1}H-NMR (400 MHz, CD_2Cl_2, nitromethane) Spectra of deuterated compound **25** following the KOD procedure for 16 h: Yield: >99%

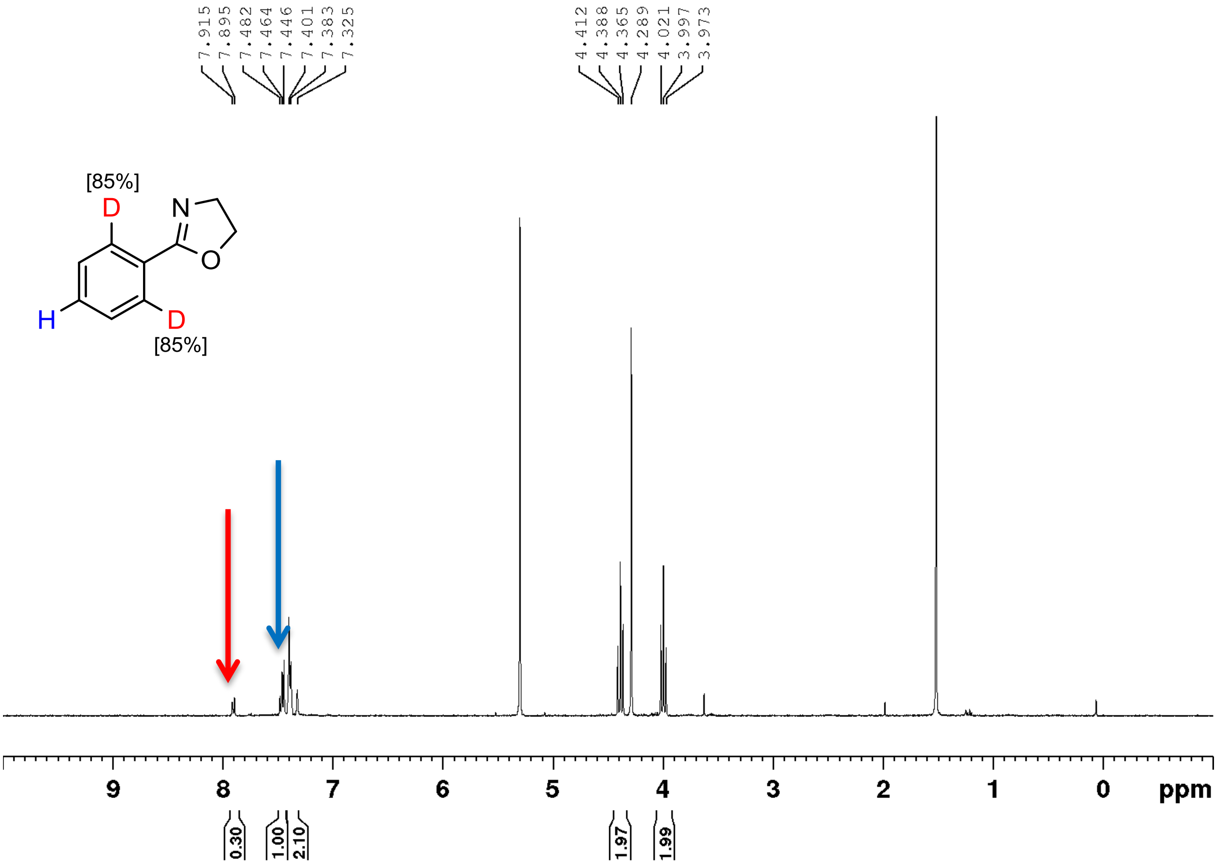

***N*-(2-hydroxyethyl)benzamide 26 (II)**

^{1}H-NMR (400 MHz, CD_2Cl_2, mesitylene) Spectra of pure compound **26**:

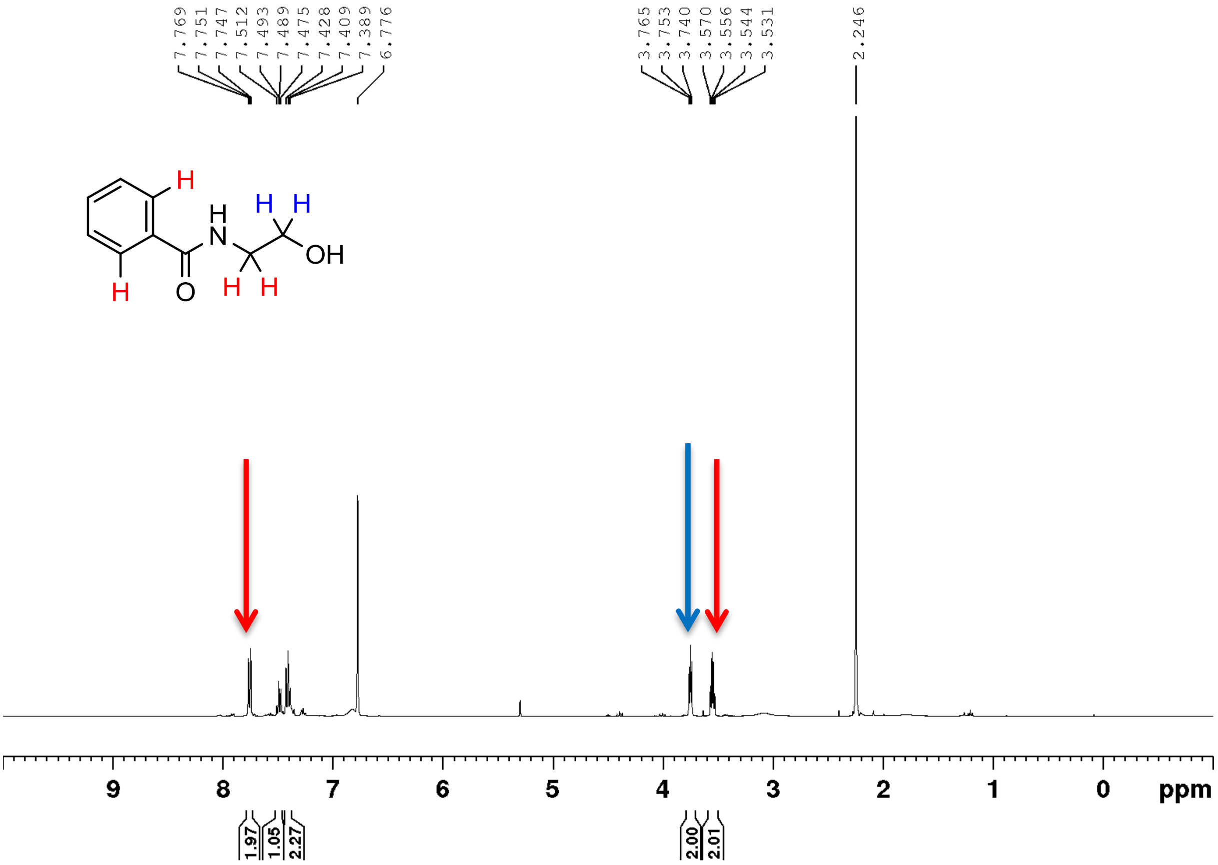

^{1}H-NMR (400 MHz, CD_2Cl_2, mesitylene) Spectra of deuterated compound **26** following the CuI procedure for 62 h: Yield: 69%

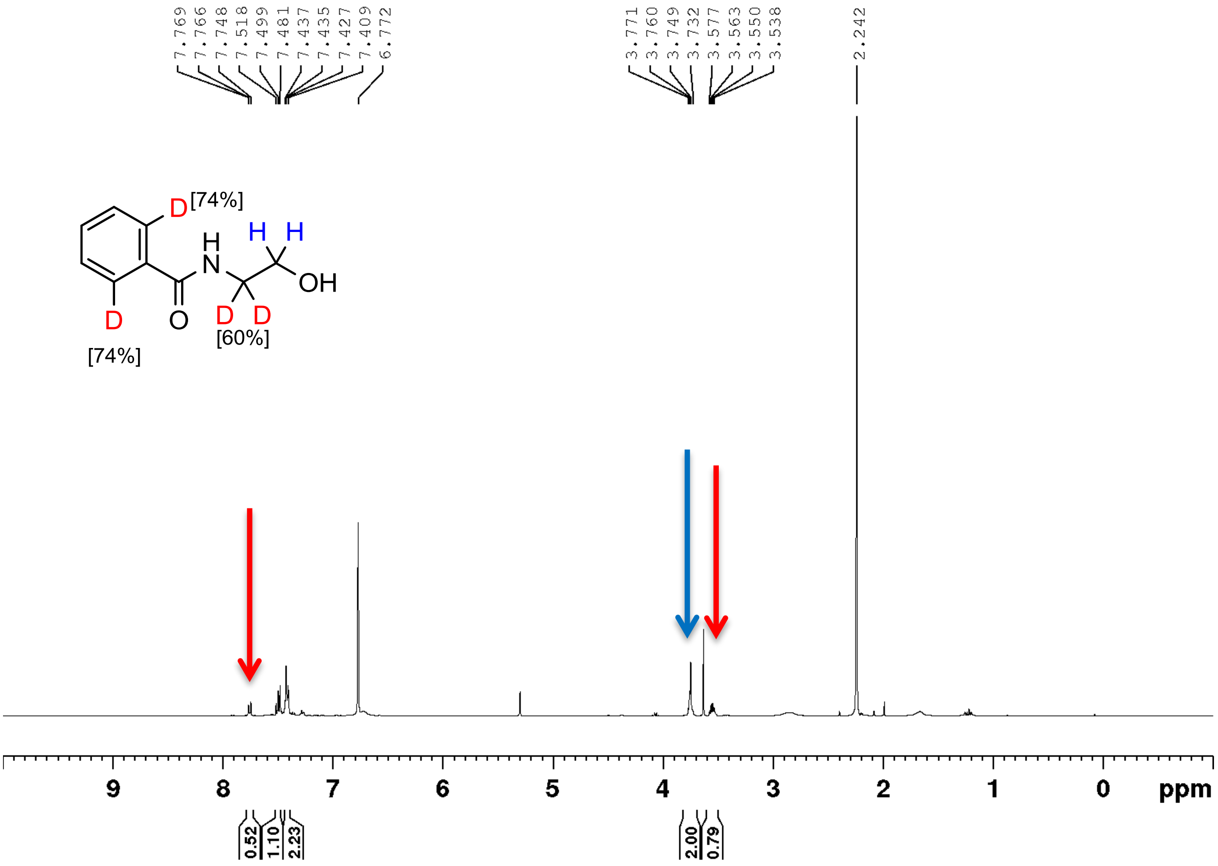

3.5.13 2-(4-methylphenyl)-4,5-dihydro-1.3-oxazole 27

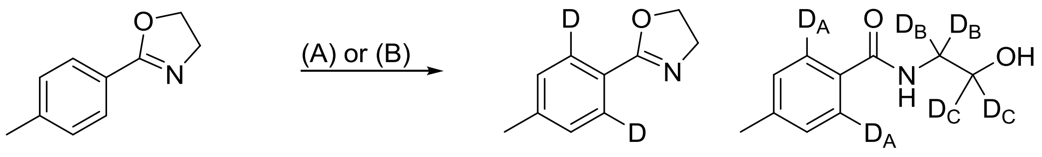

I **II**

		I				II			
	t	D_A	D_B	D_C	Yield	D_A	D_B	D_C	Yield
cond. (A)	16 h	n.o.	n.o.	n.o.	n.o.	81%	74%	26%	98%
	62 h	-	-	-	-	-	-	-	-
cond. (C)	16 h	22%	n.o.	n.o.	90%	n.o.	n.o.	n.o.	n.o.
	62 h	85%	n.o.	n.o.	83%	n.o.	n.o.	n.o.	n.o.

2-(4-methylphenyl)-4,5-dihydro-1.3-oxazole 27 (I)

^{1}H-NMR (400 MHz, $CDCl_3$) Spectra of pure compound **27**:

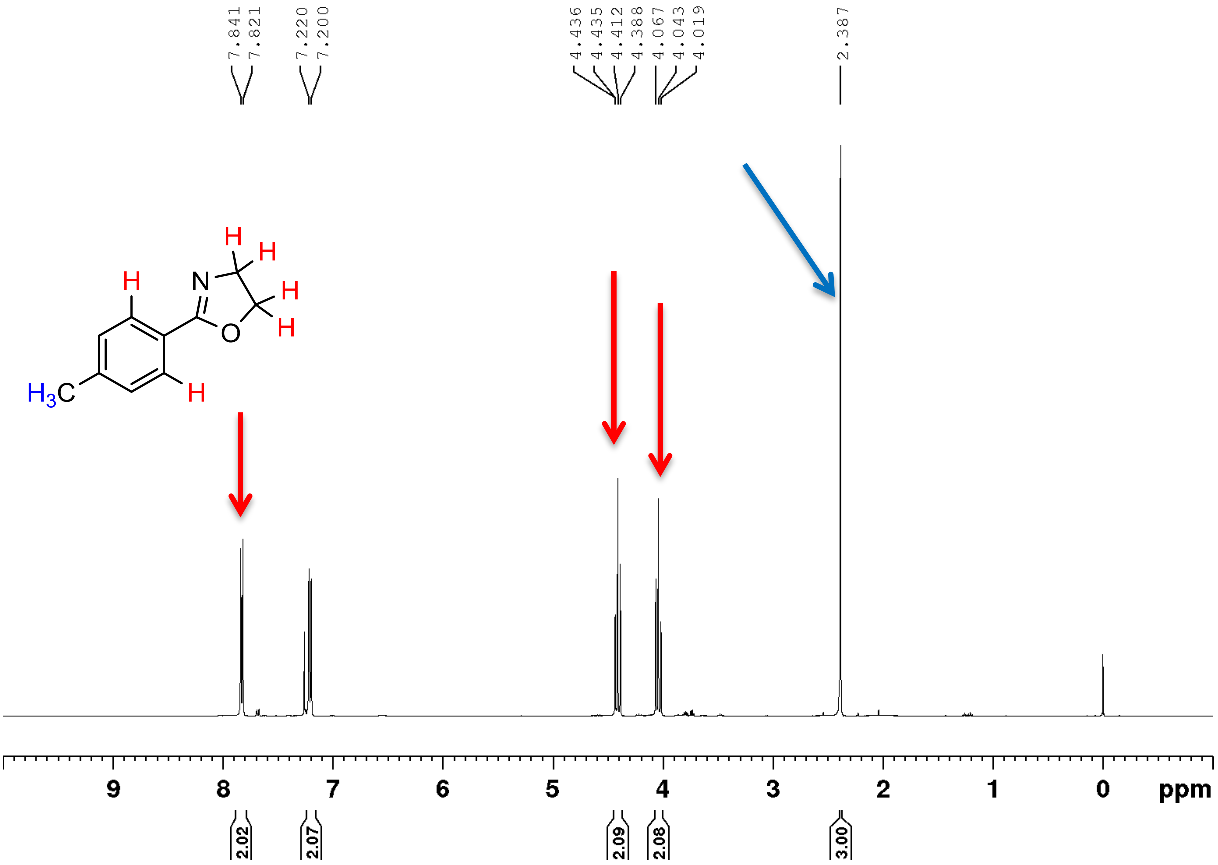

^{1}H-NMR (400 MHz, $CDCl_3$, mesitylene) Spectra of deuterated compound **27** following the KOD procedure for 16 h: Yield: 90%

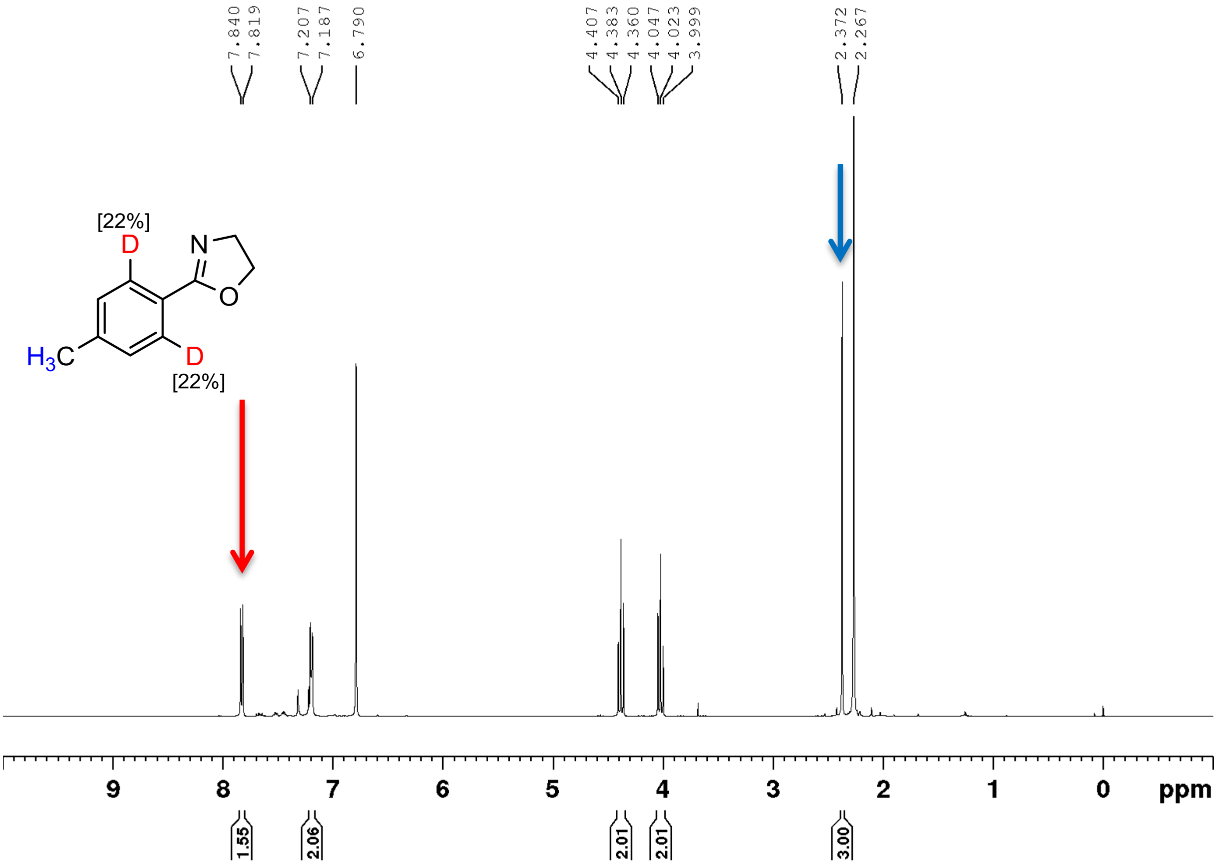

^{1}H-NMR (400 MHz, $CDCl_3$, mesitylene) Spectra of deuterated compound **27** following the KOD proceduren for 62 h: Yield: 83%

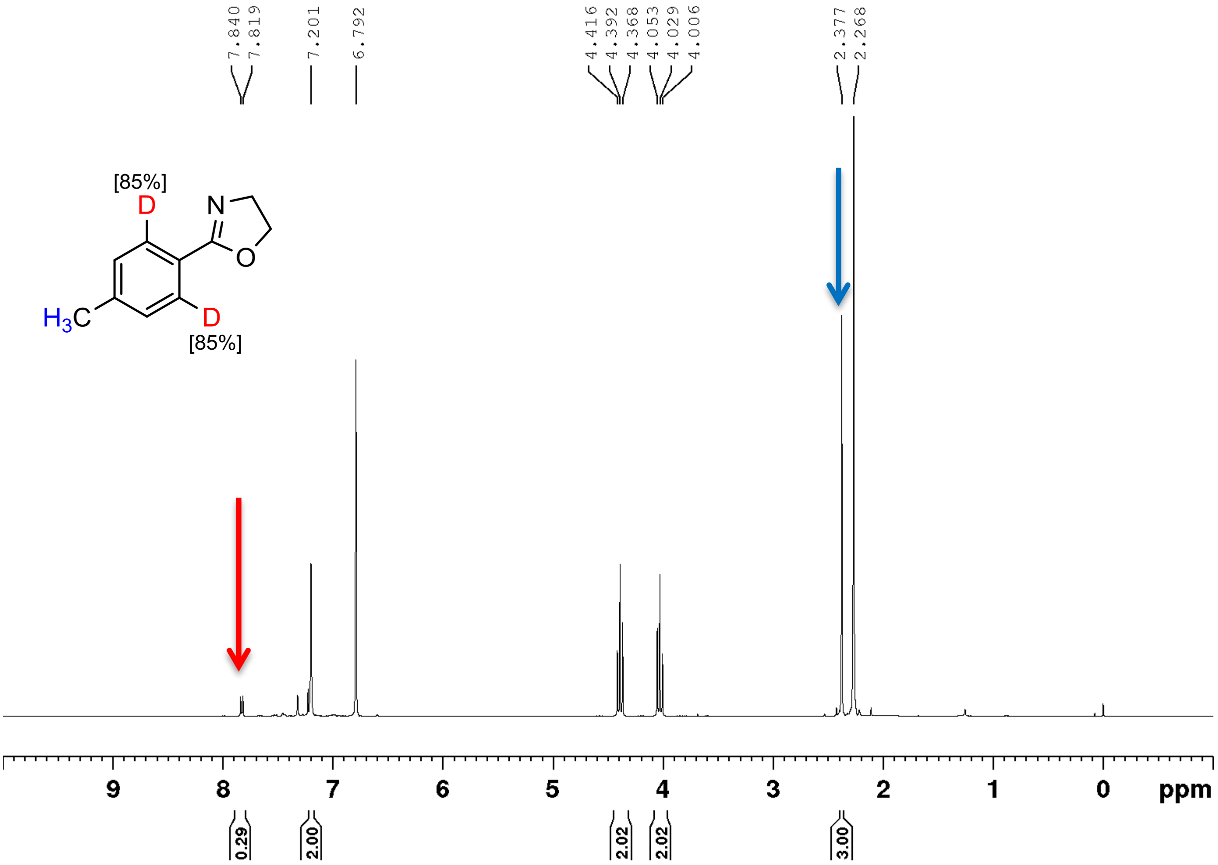

***N*-(2-Hydroxyethyl)-4-methylbenzamide 28[10] (II)**

^{1}H-NMR (400 MHz, $CDCl_3$, mesitylene) Spectra of deuterated compound **28** following the CuI procedure for 16 h: Yield: 98%

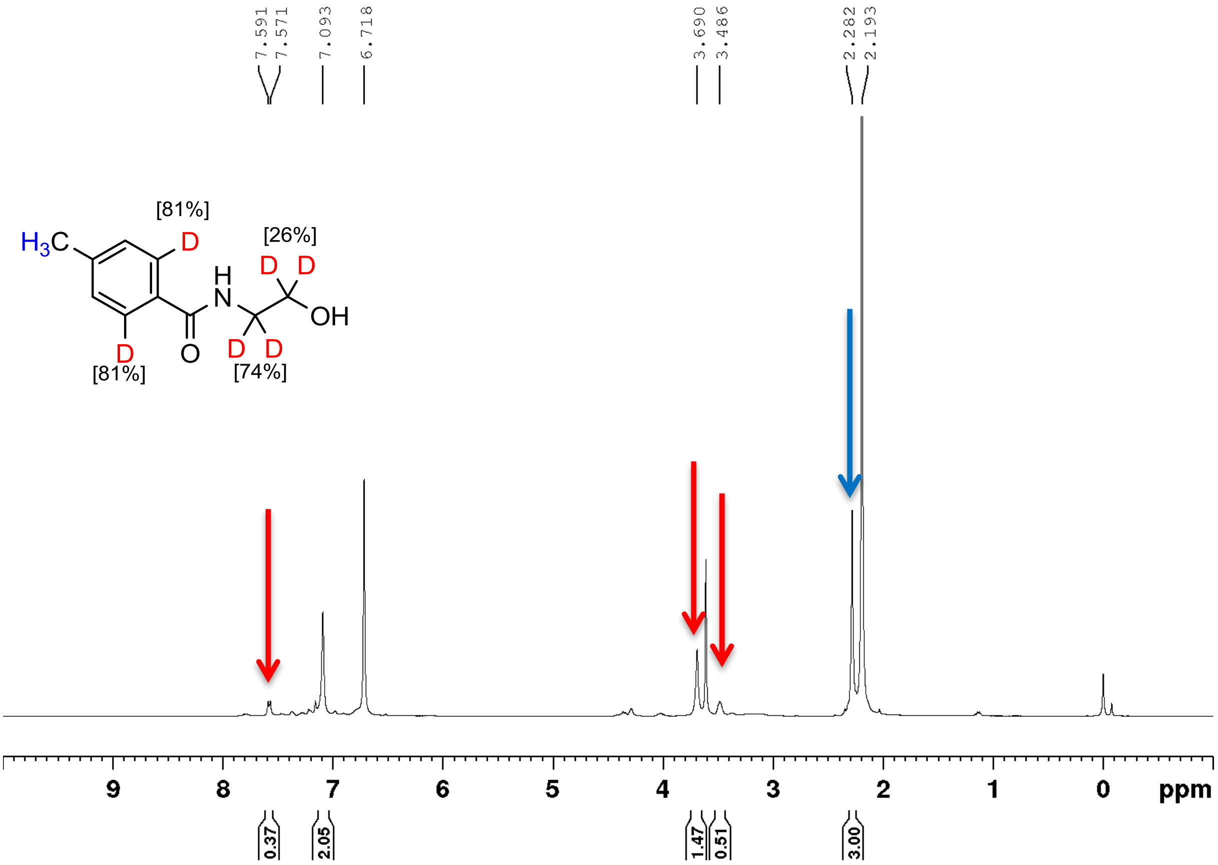

3.5.14 2-(4-chlorophenyl)-4,5-dihydro-1.3-oxazole 29

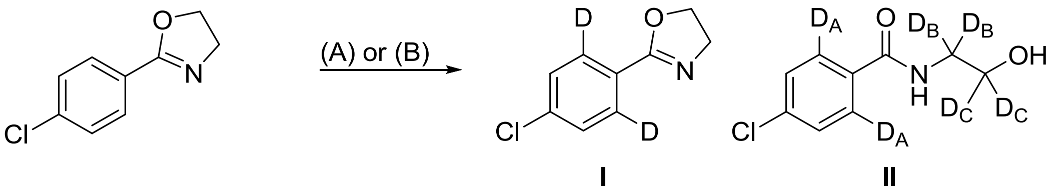

	I					II			
	t	D_A	D_B	D_C	Yield	D_A	D_B	D_C	Yield
cond. (A)	16 h	n.o.	n.o.	n.o.	n.o.	80%	69%	<10%	68%
	62 h	-	-	-	-	-	-	-	-
cond. (C)	16 h	<10%	n.o.	n.o.	95%	n.o.	n.o.	n.o.	n.o.
	62 h	84%	n.o.	n.o.	91%	n.o.	n.o.	n.o.	n.o.

2-(4-chlorophenyl)-4,5-dihydro-1.3-oxazole 29 (I)

^{1}H-NMR (400 MHz, $CDCl_3$) Spectra of pure compound **29**:

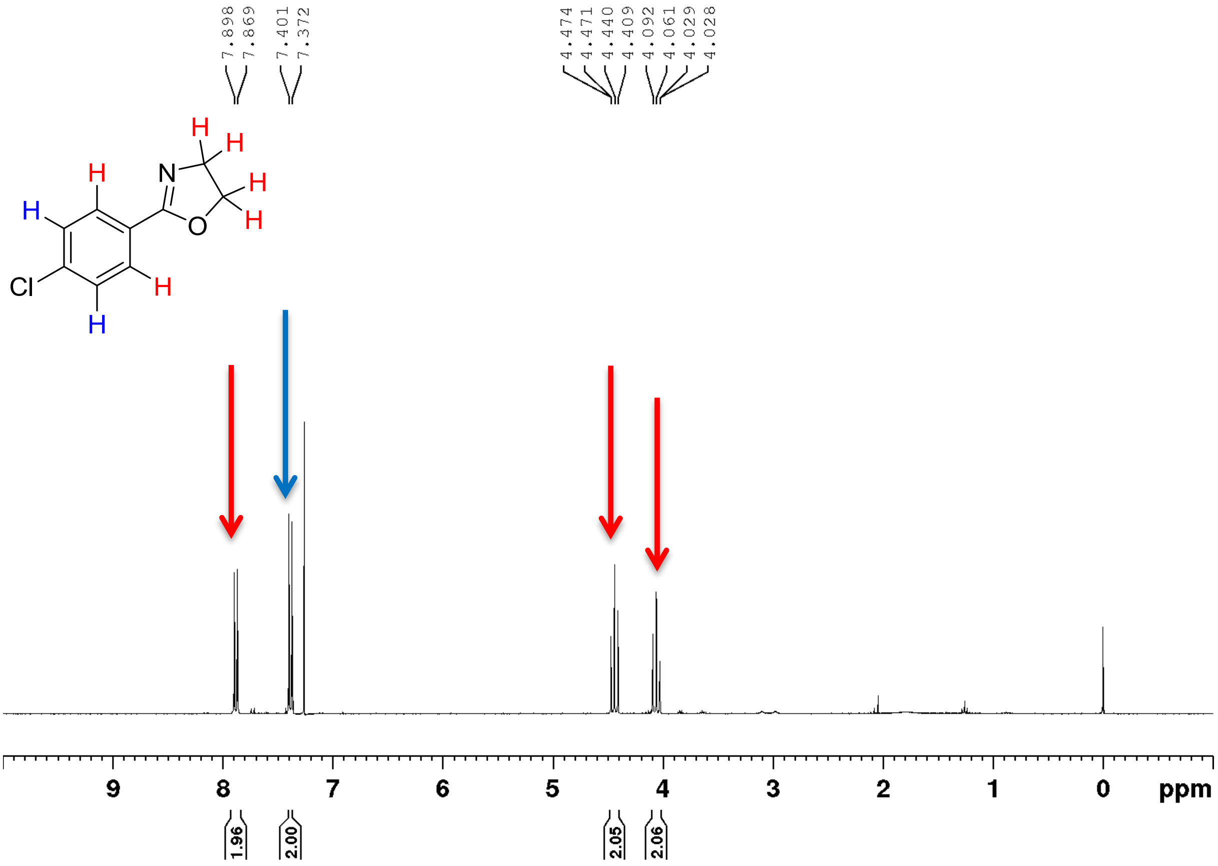

^{1}H-NMR (400 MHz, $CDCl_3$, mesitylene) Spectra of deuterated compound **29** following the KOD procedure for 16 h: Yield: 95%

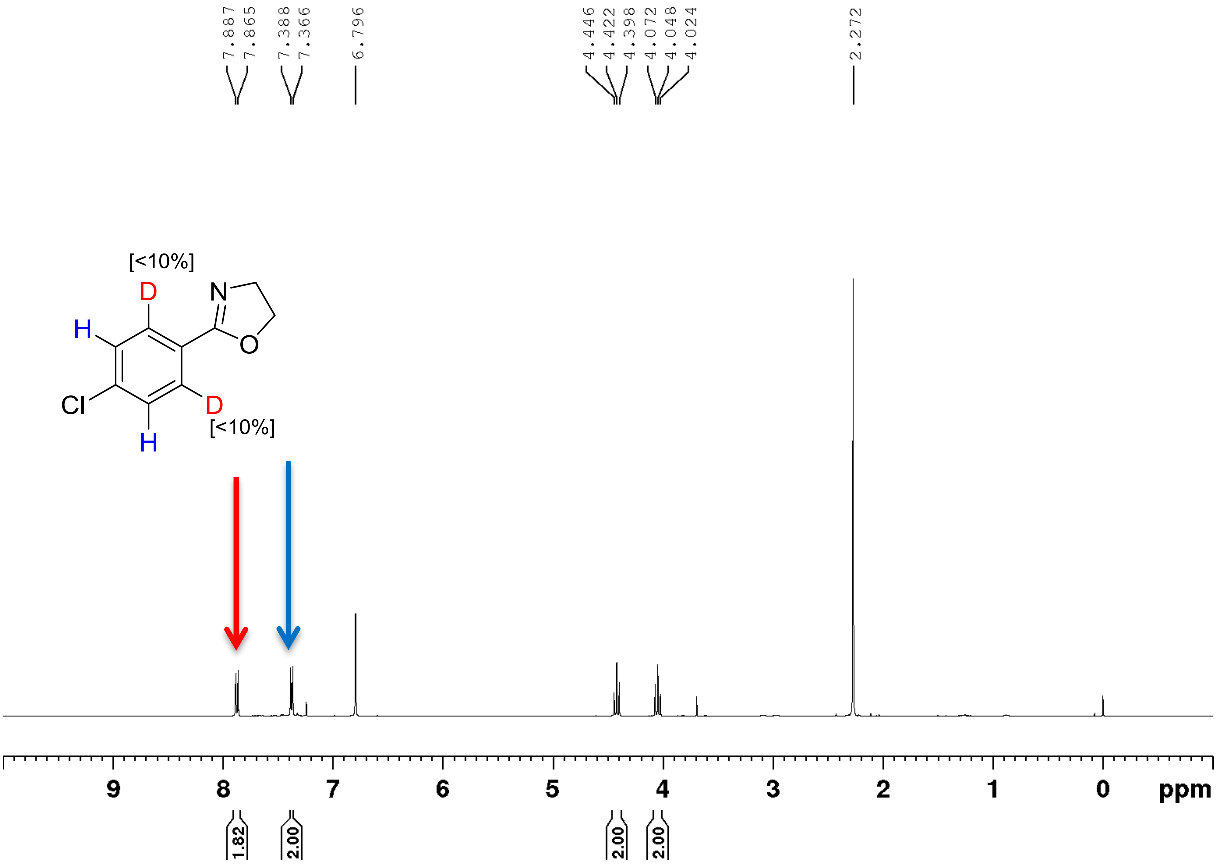

^{1}H-NMR (400 MHz, $CDCl_3$, mesitylene) Spectra of deuterated compound **29** following the KOD procedure for 62 h: Yield: 91%

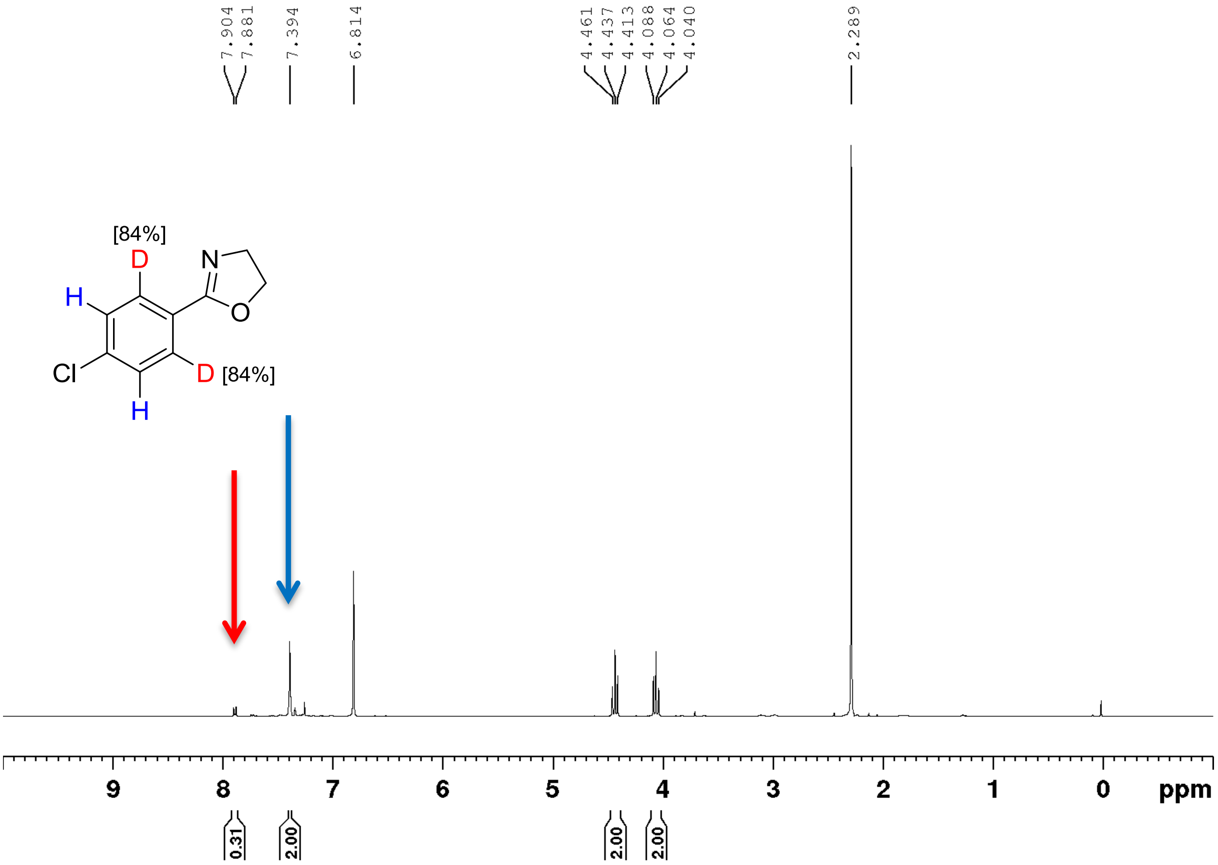

4-Chloro-*N*-(2-Hydroxyethyl)benzamide 30[10] (II)

^{1}H-NMR (400 MHz, $CDCl_3$, mesitylene) Spectra of deuterated compound **30** following the CuI procedure for 16 h: Yield: 68%

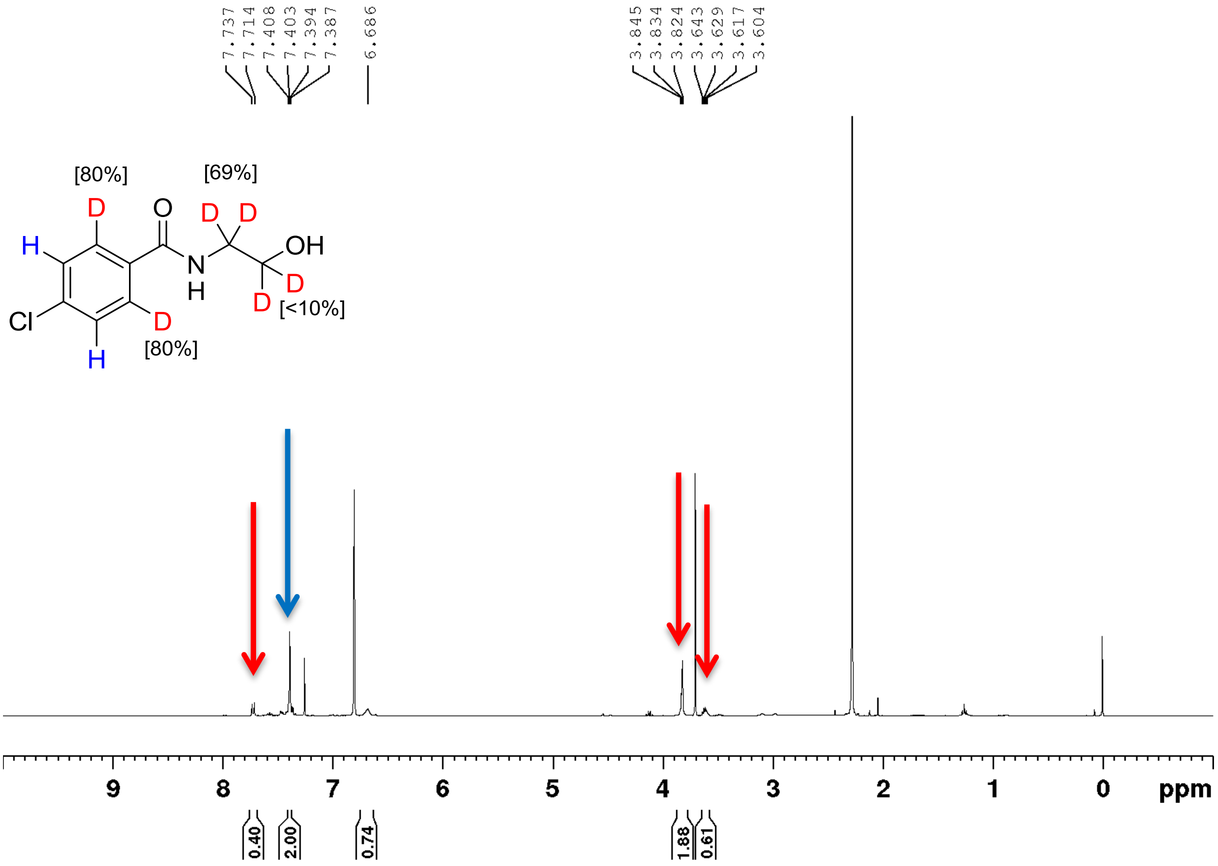

3.5.15 2-(3-chlorophenyl)-4,5-dihydro-1.3-oxazole 31

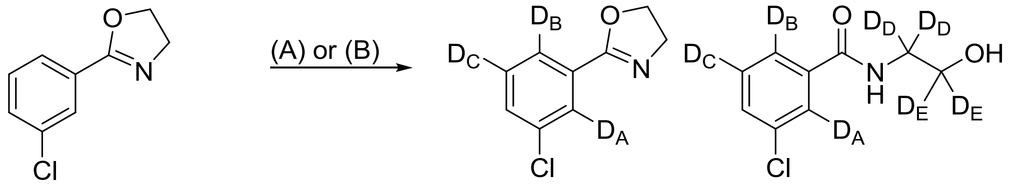

I II

	I							II					
	t	D_A	D_B	D_C	D_D	D_E	Yield	D_A	D_B	D_C	D_D	D_E	Yield
cond. (A)	16 h	n.o.	n.o.	n.o.	n.o.	n.o.	n.o.	74%	80%	n.o.	66%	16%	93%
	62 h	-	-	-	-	-	-	-	-	-	-	-	-
cond. (C)	16 h	24%	25%	n.o.	n.o.	n.o.	95%	n.o.	n.o.	n.o.	n.o.	n.o.	n.o.
	62 h	82%	83%	30%	n.o.	n.o.	96%	n.o.	n.o.	n.o.	n.o.	n.o.	n.o.

2-(3-chlorophenyl)-4,5-dihydro-1.3-oxazole 31 (I)
^{1}H-NMR (400 MHz, $CDCl_3$) Spectra of pure compound **31**:

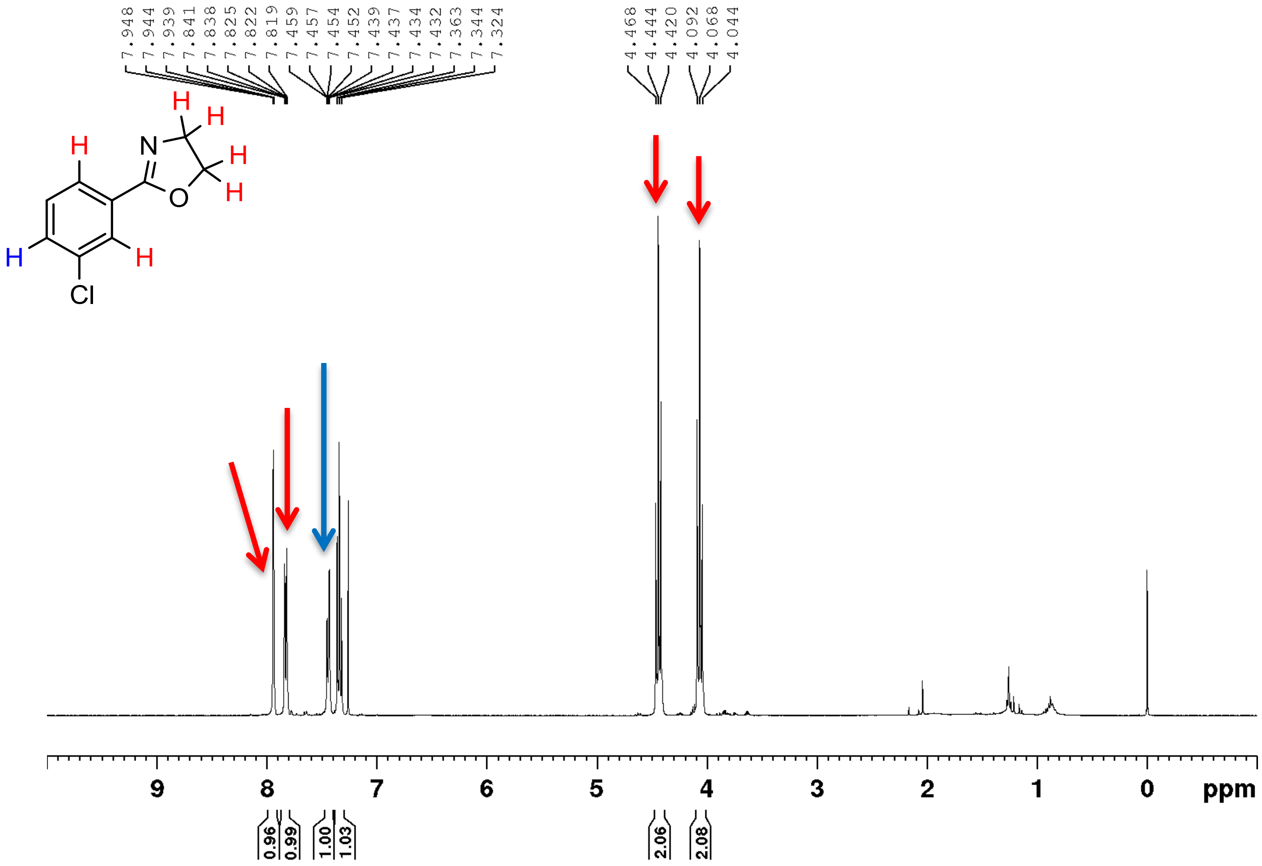

^{1}H-NMR (400 MHz, $CDCl_3$, mesitylene) Spectra of deuterated compound **31** following the KOD procedure for 16 h: Yield: 95%

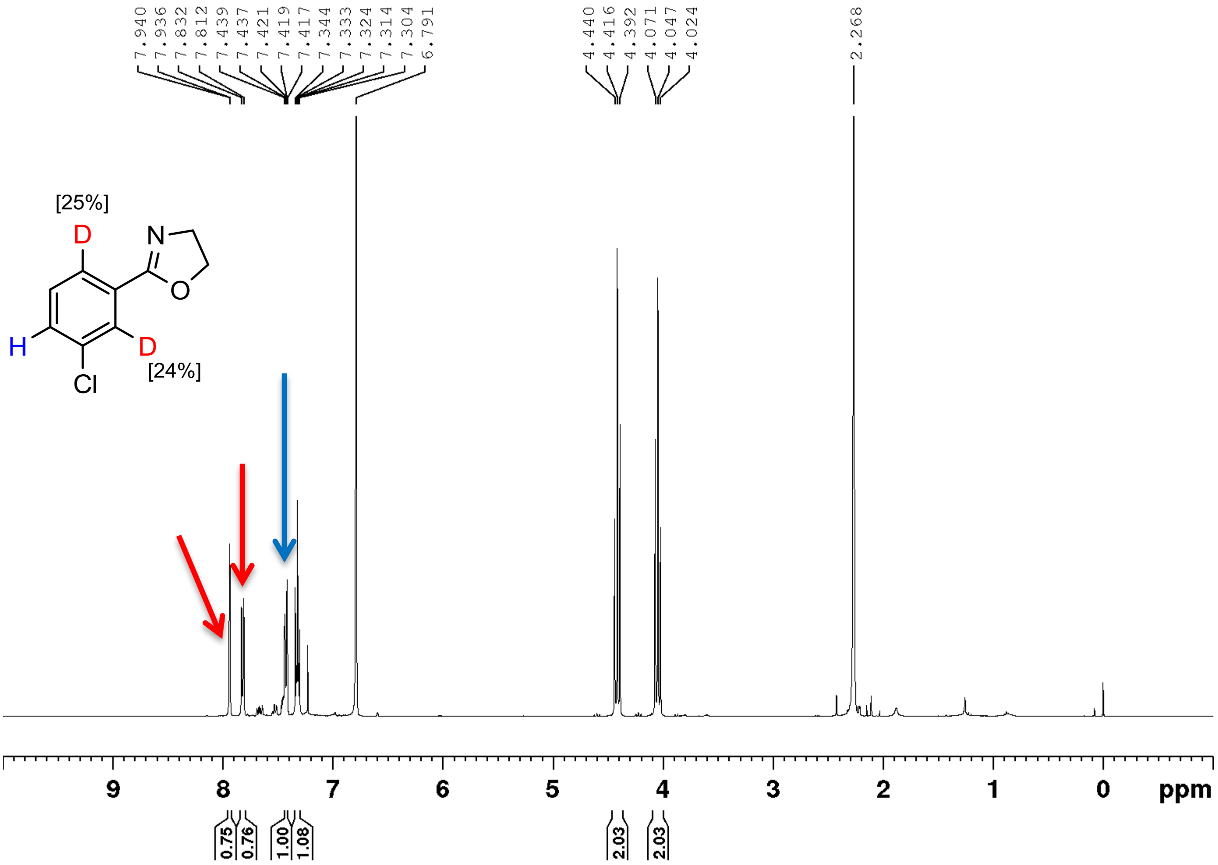

Enlargement of relevant area:

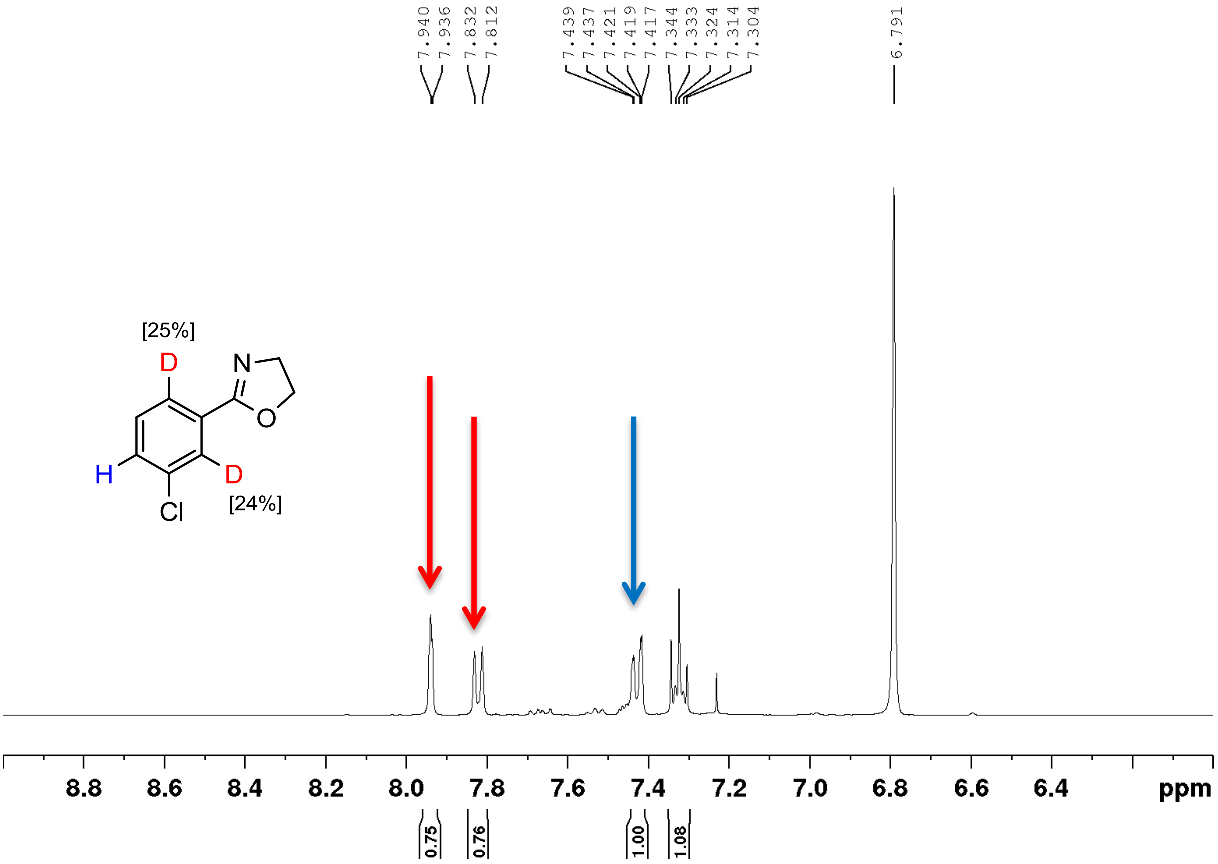

^{1}H-NMR (400 MHz, $CDCl_3$, mesitylene) Spectra of deuterated compound **31** following the KOD procedure for 62 h: Yield: 96%

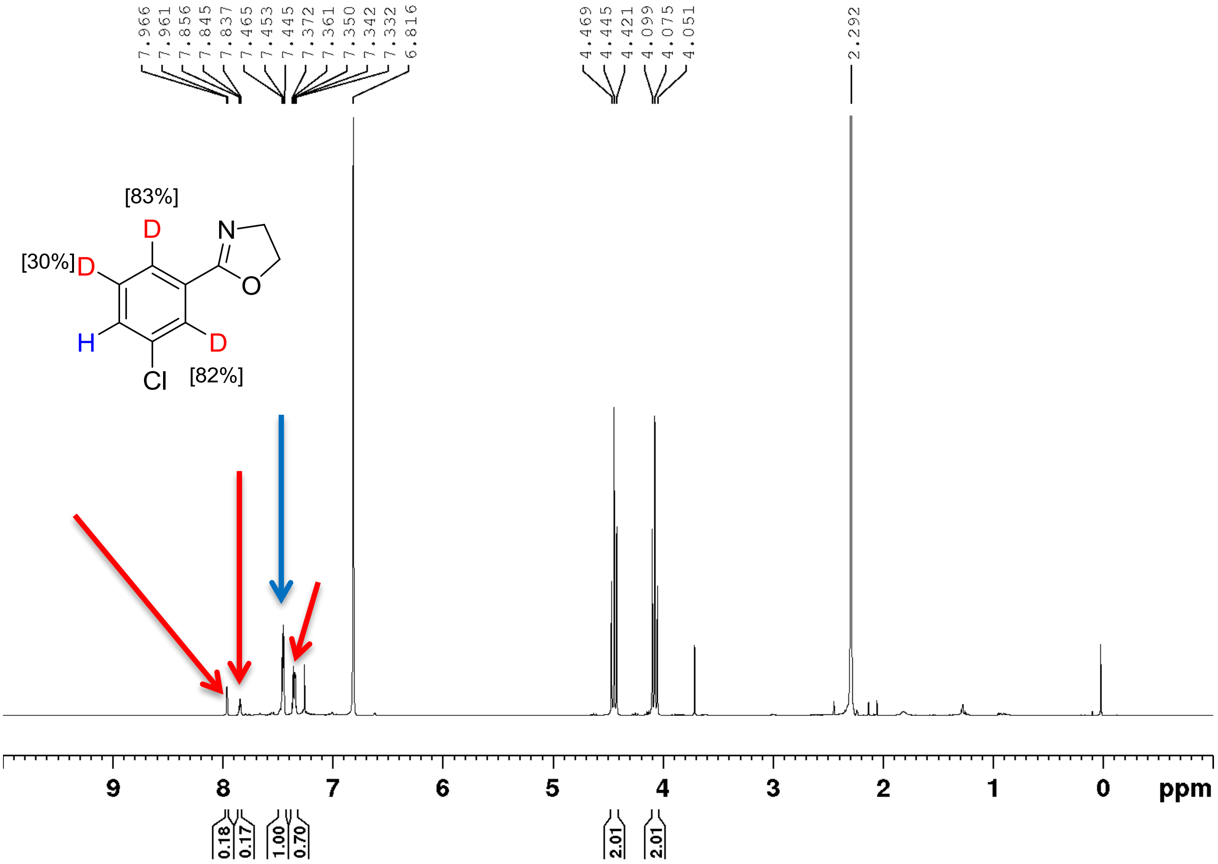

Enlargement of relevant area:

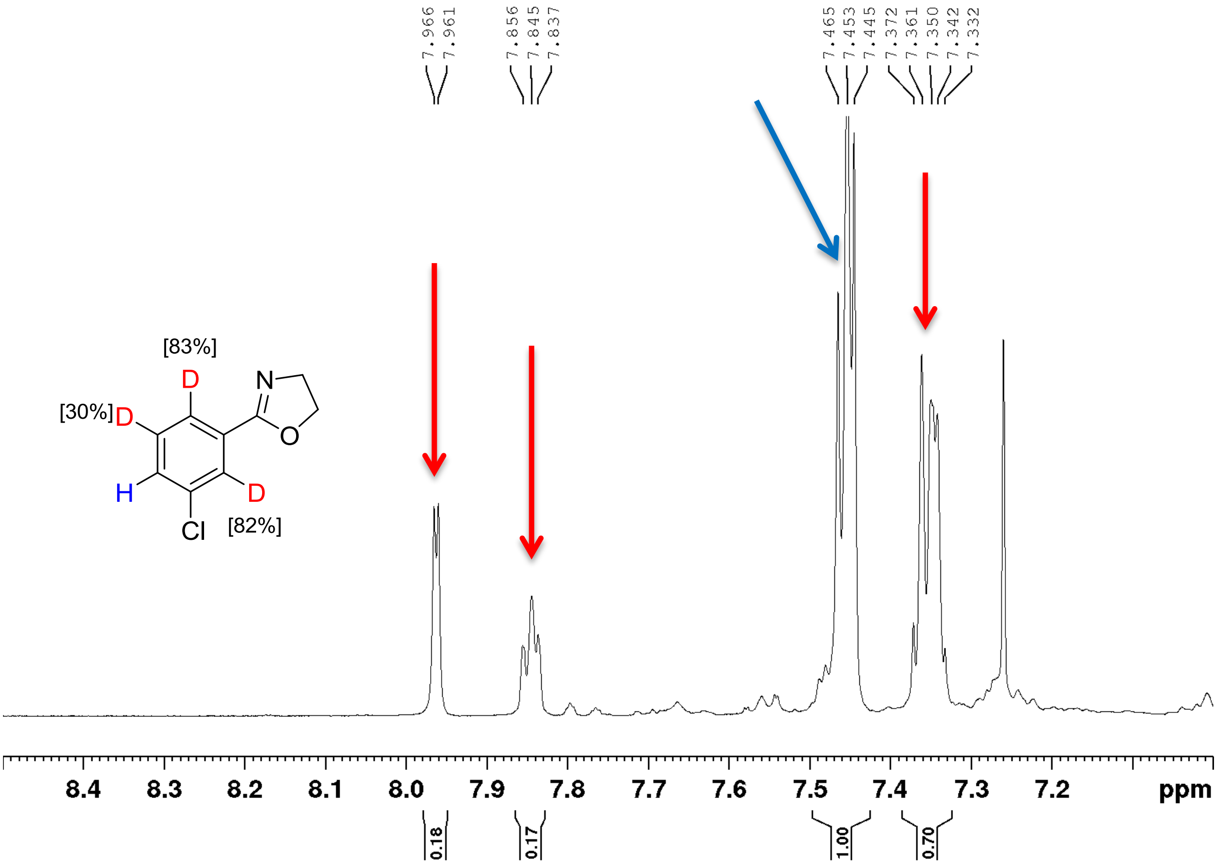

3-chloro-*N*-(2-hydroxyethyl)benzamide 32 (II)

^{1}H-NMR (400 MHz, $CDCl_3$) Spectra of pure compound **32**:

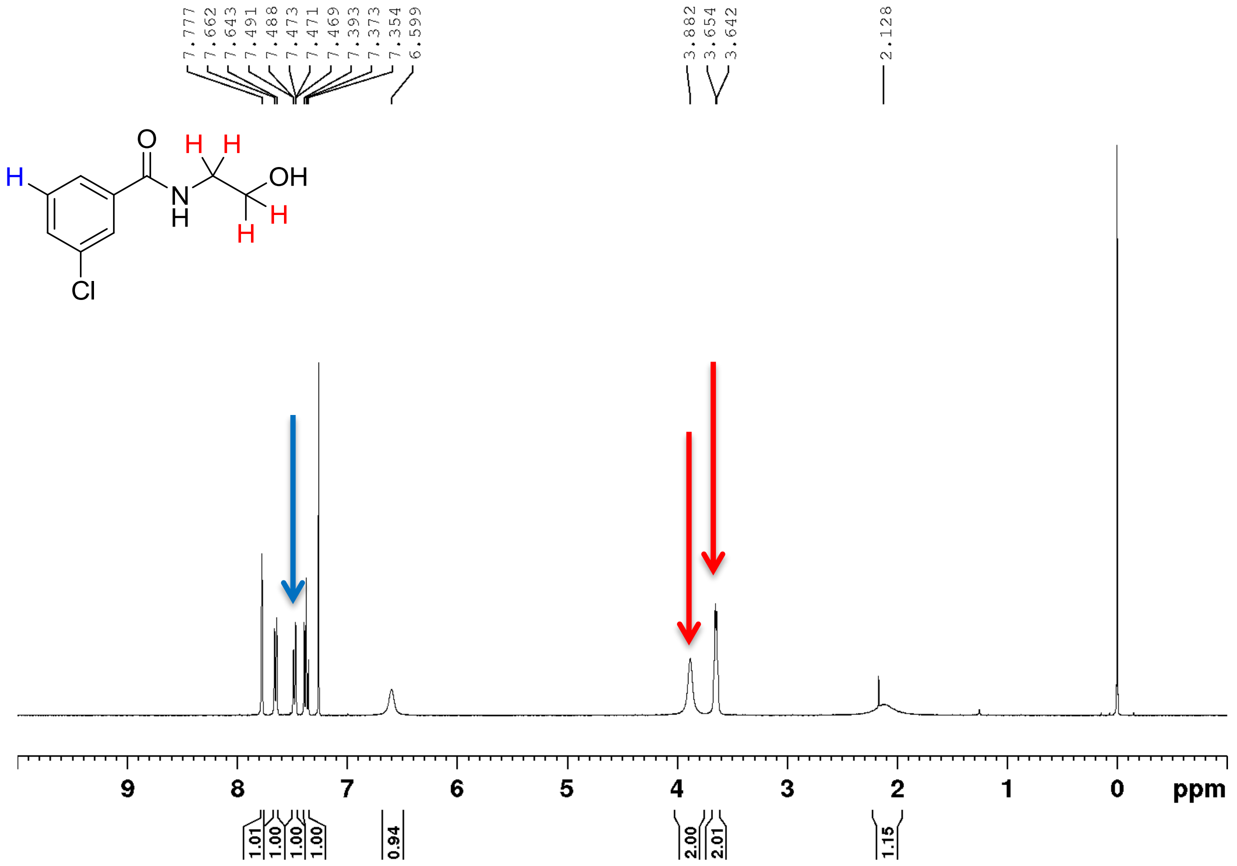

^{1}H-NMR (400 MHz, $CDCl_3$) Spectra of deuterated compound **32** following the CuI procedure for 16 h: Yield: 93%

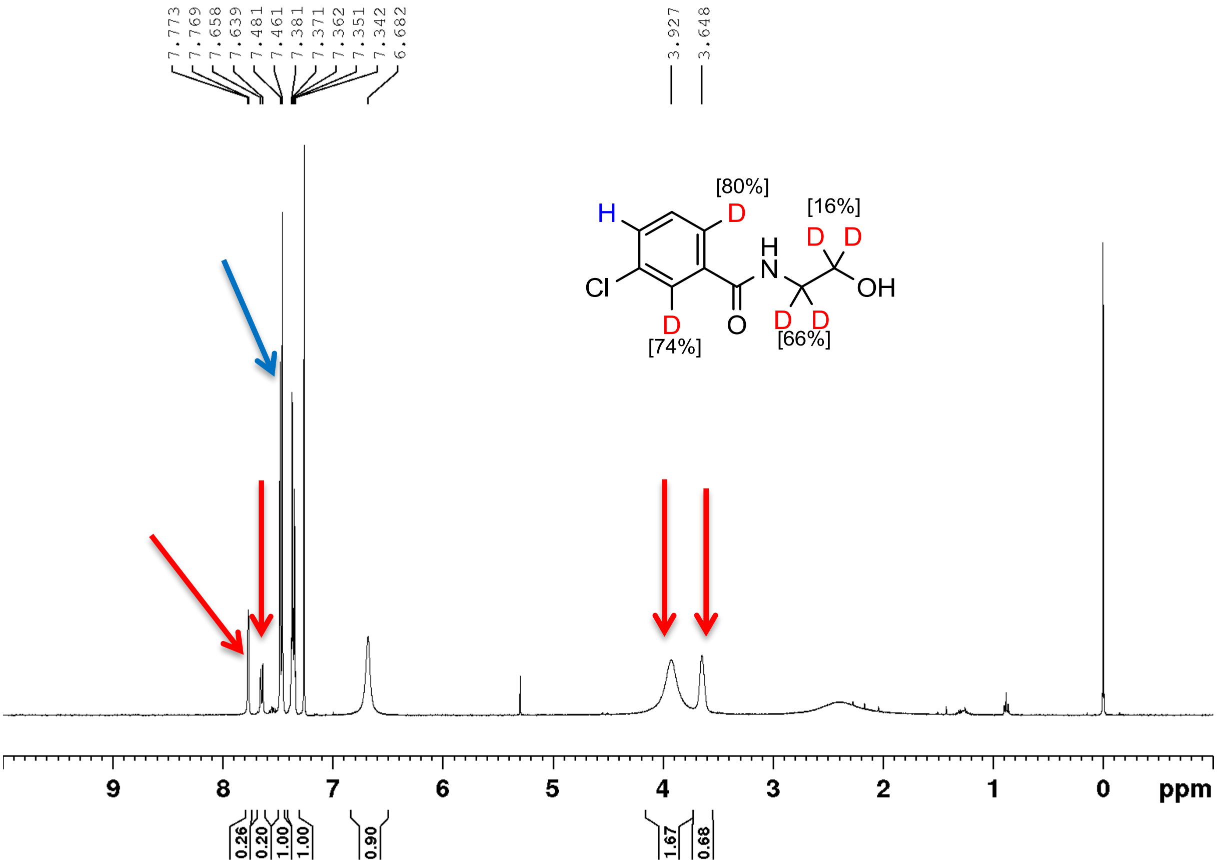

Enlargement of relevant area:

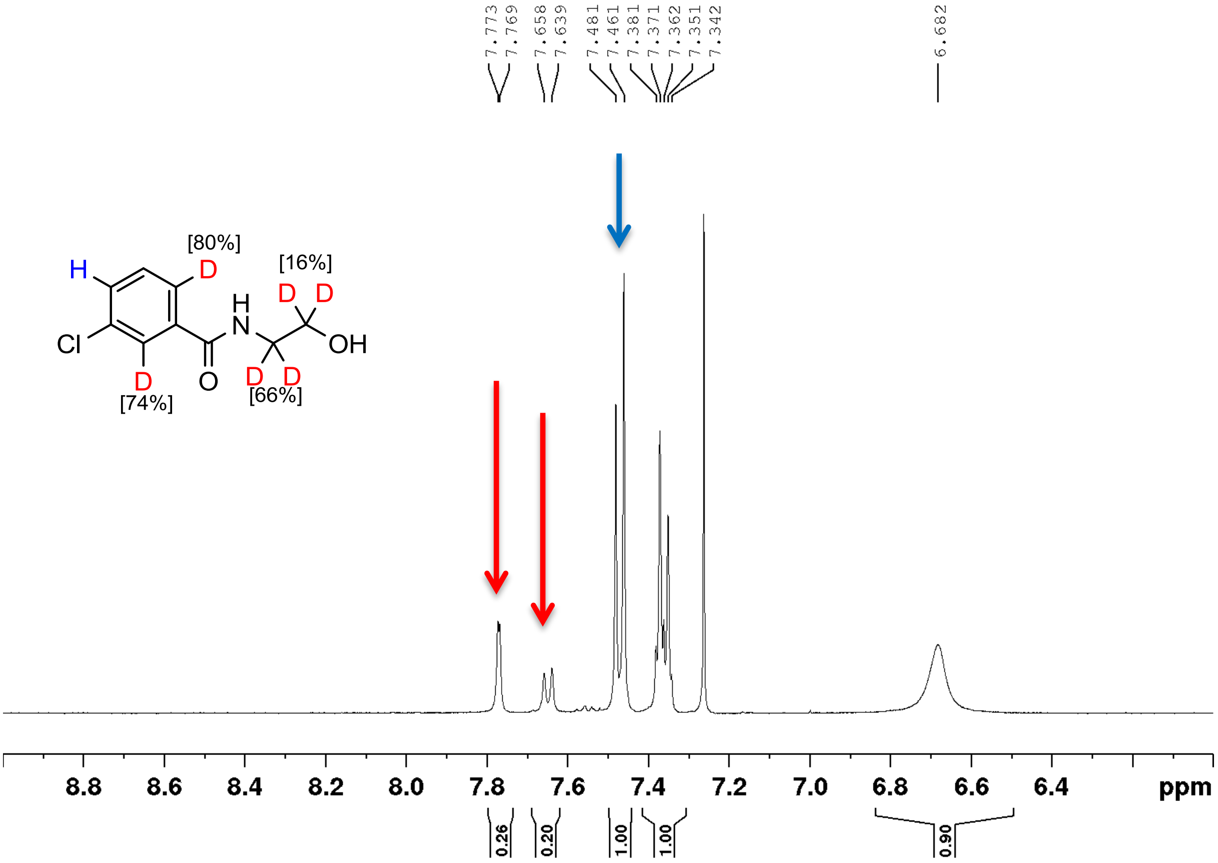

3.5.16 N-(*n*-propyl)benzamide 33

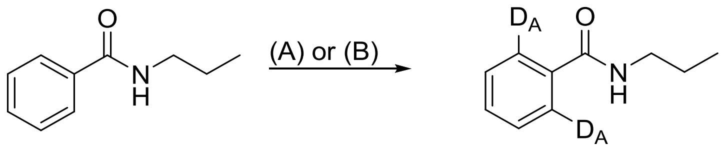

	t	D_A	Yield
cond. (A)	16 h	65%	>99%
	62 h	80%	>99%
cond. (B)	16 h	36%	>99%
	62 h	85%	>99%
cond. (C)	62 h	10%	>99%

N-(*n*-propyl)benzamide 33

^{1}H-NMR (400 MHz, $CDCl_3$) Spectra of pure compound **33**:

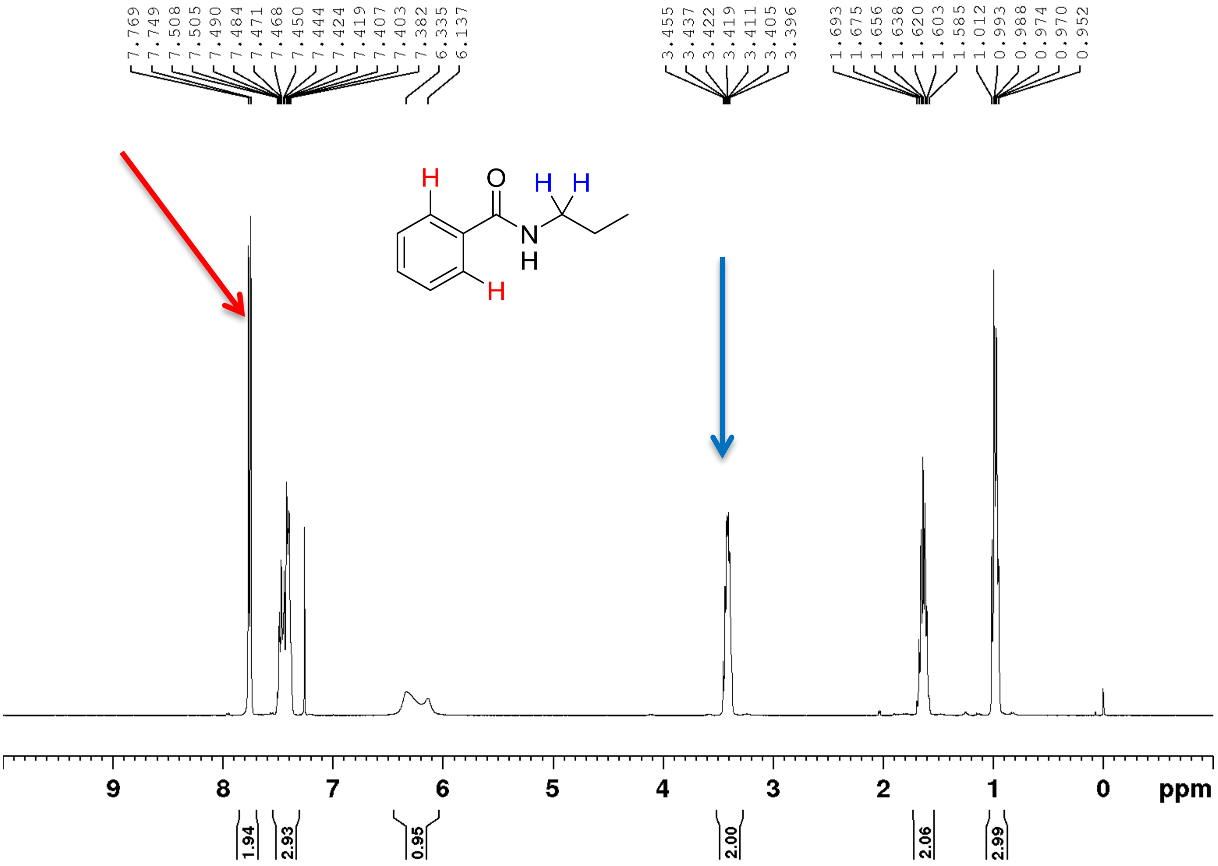

^{1}H-NMR (400 MHz, $CDCl_3$, mesitylene) Spectra of deuterated compound **33** following the KOD procedure for 62 h: Yield: >99%

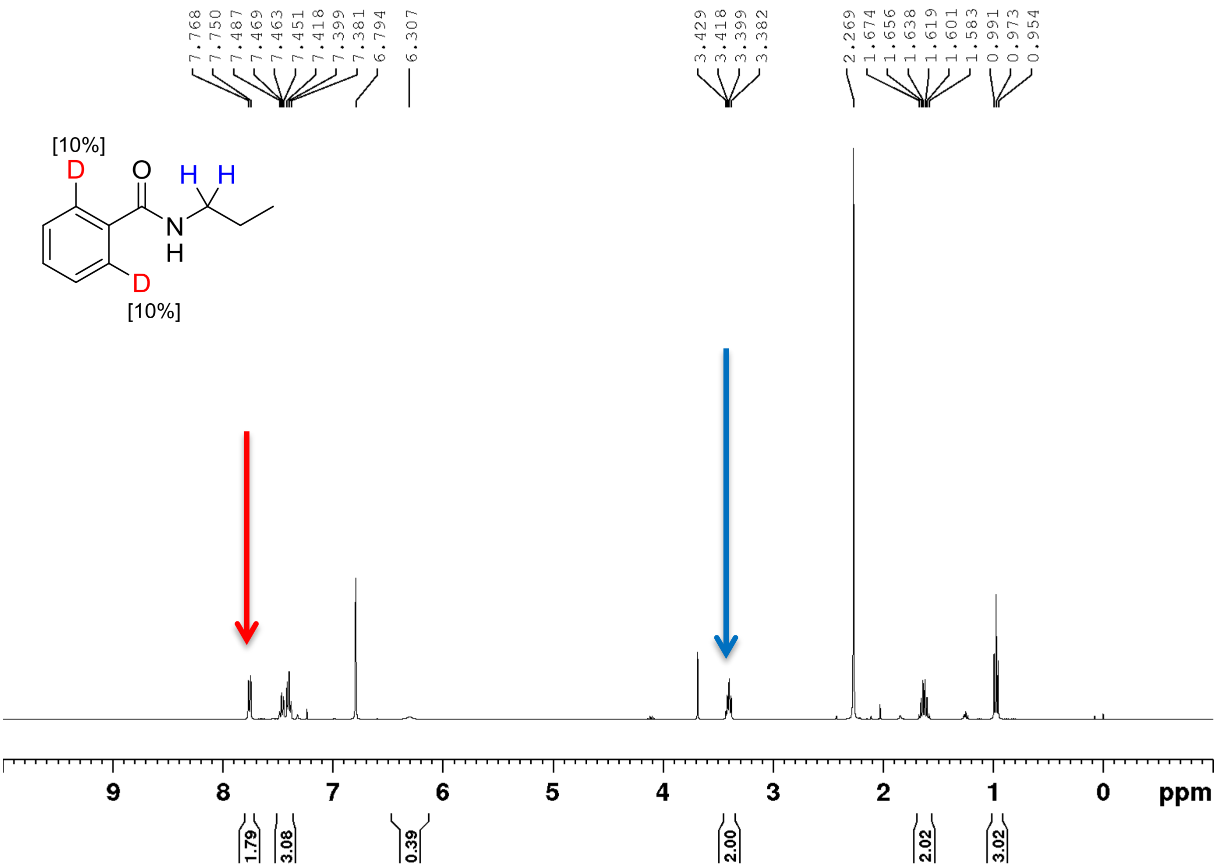

^{1}H-NMR (400 MHz, $CDCl_3$, mesitylene) Spectra of deuterated compound **33** following the KOD/Zn procedure for 16 h: Yield: >99%

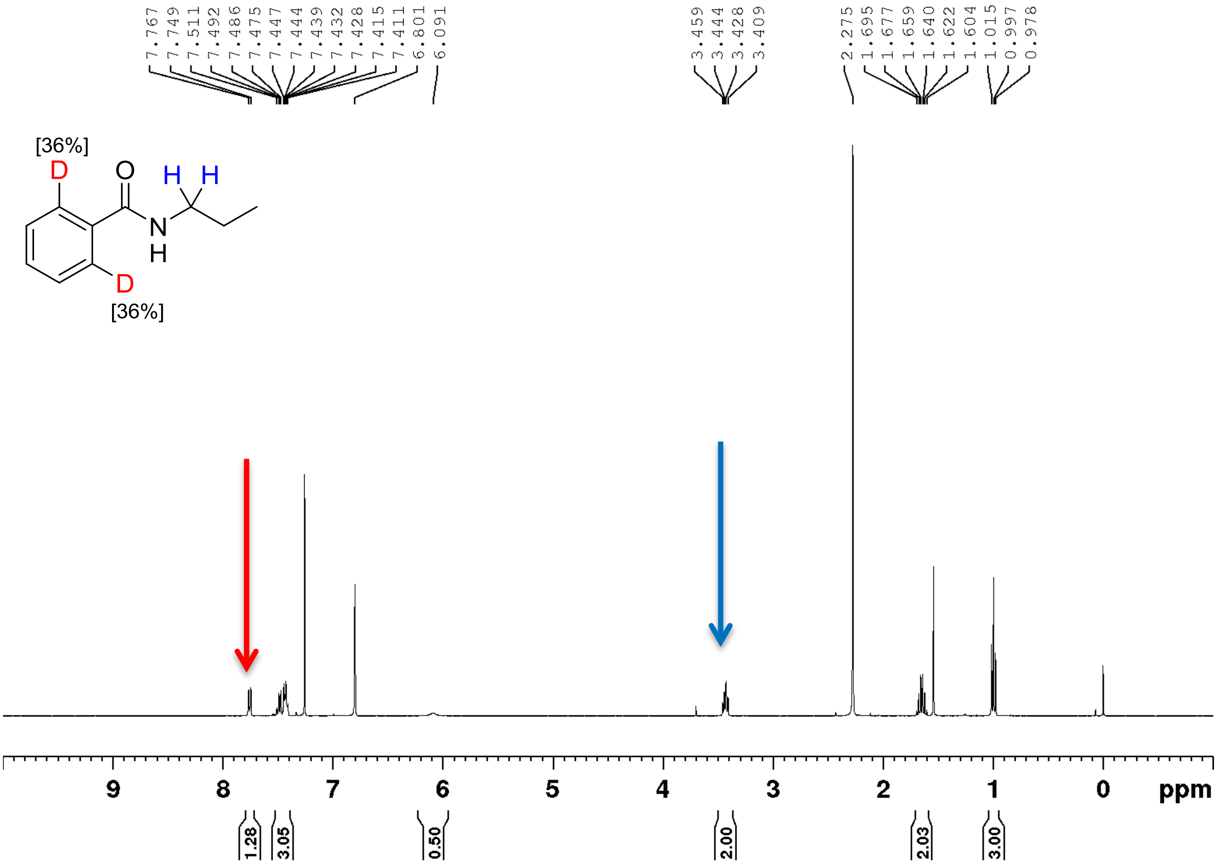

^{1}H-NMR (400 MHz, $CDCl_3$, mesitylene) Spectra of deuterated compound **33** following the KOD/Zn procedure for 62 h: Yield: >99%

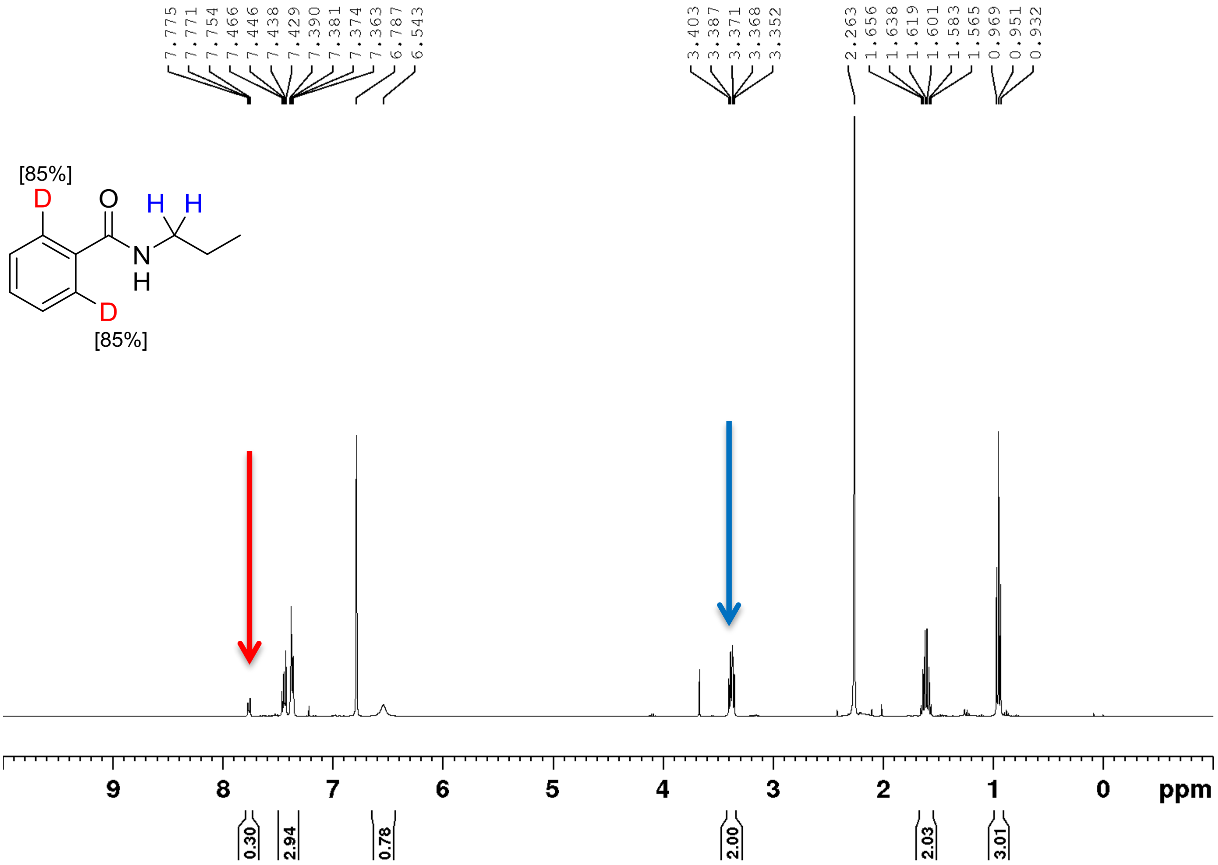

^{1}H-NMR (400 MHz, $CDCl_3$, mesitylene) Spectra of deuterated compound **33** following the CuI procedure for 16 h: Yield: >99%

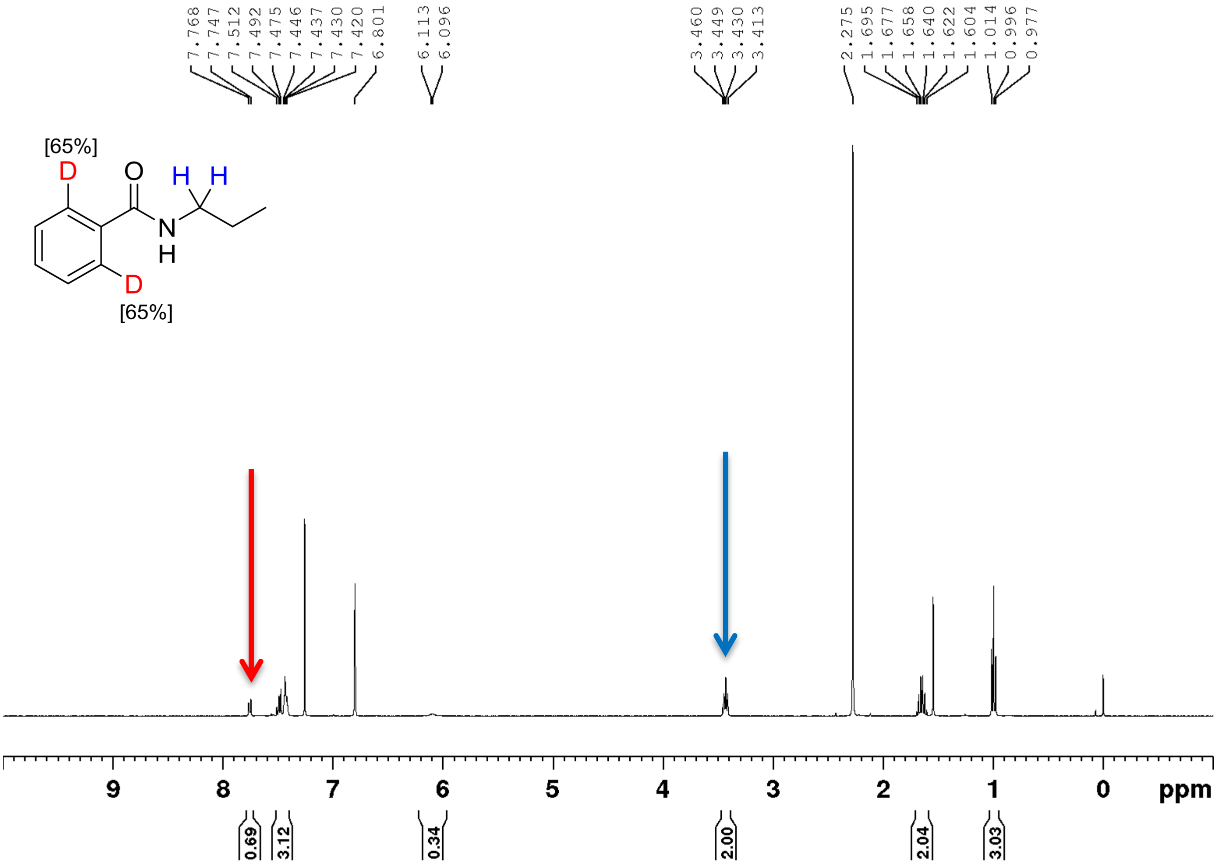

^{1}H-NMR (400 MHz, $CDCl_3$, mesitylene) Spectra of deuterated compound **33** following the CuI procedure for 62 h: Yield: >99%

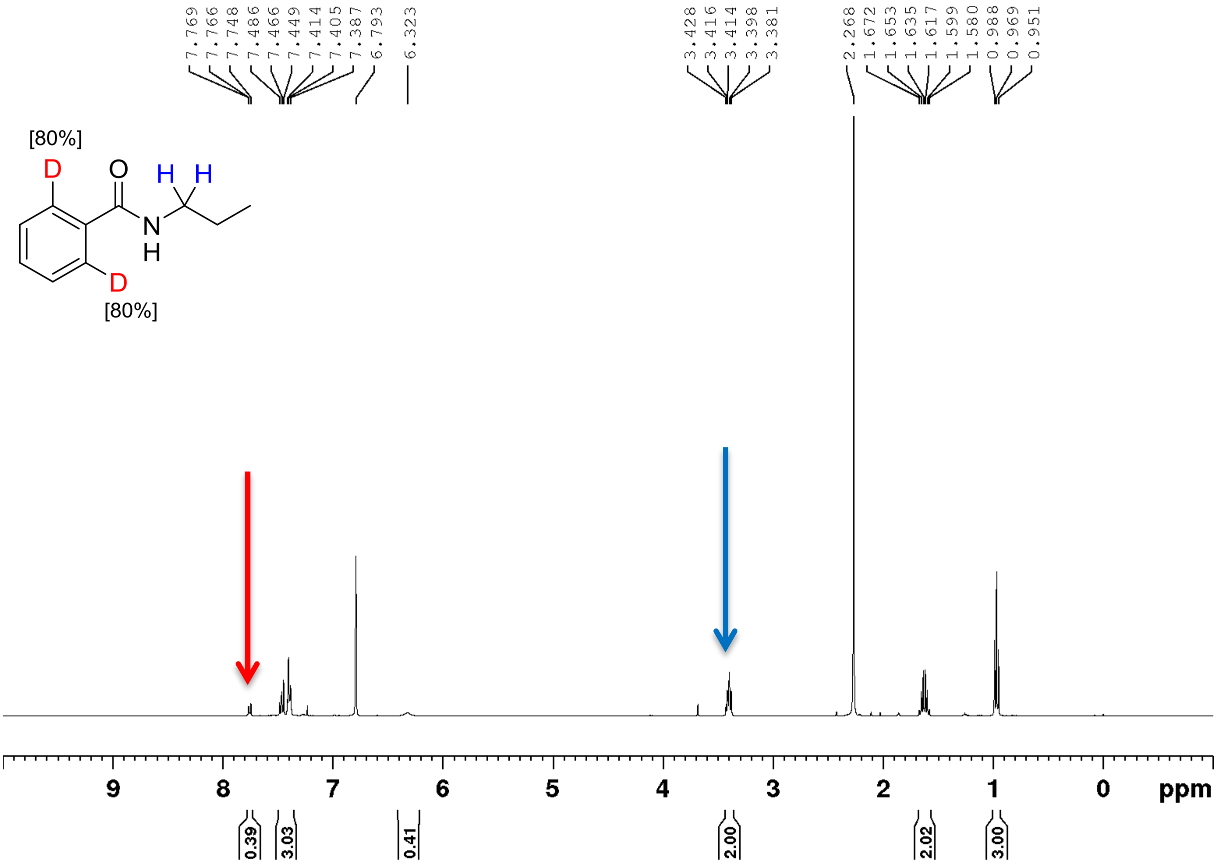

3.5.17 2-(4-Morpholinyl)pyridine 34

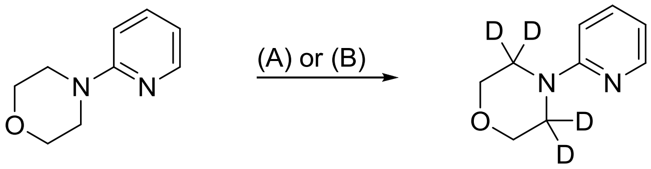

	t	D	Yield
cond. (A)	16 h	21%	93%
	62 h	-	-
cond. (C)	16 h	n.o.	n.o.
	62 h	-	-

2-(4-Morpholinyl)pyridine 34

^{1}H-NMR (300 MHz, $CDCl_3$) Spectra of pure compound **34**:

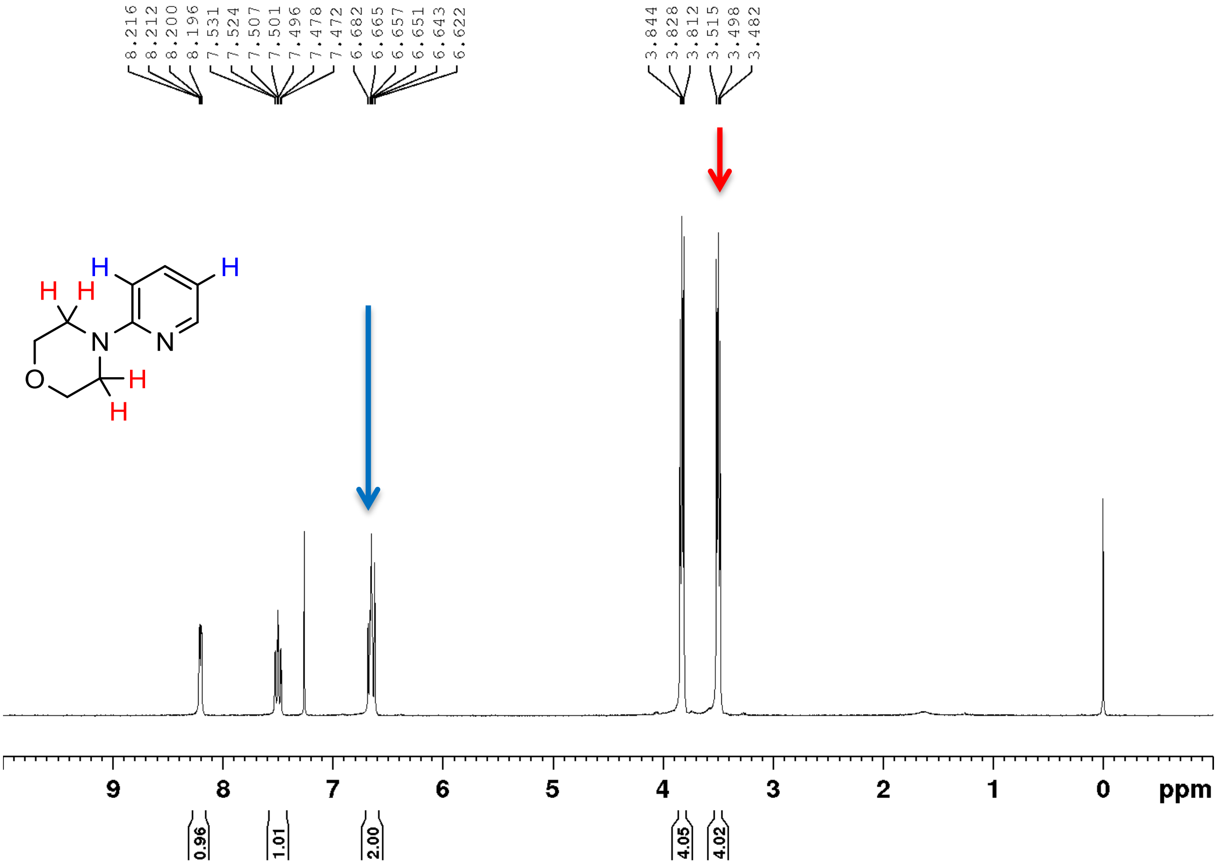

^{1}H-NMR (400 MHz, $CDCl_3$, mesitylene) Spectra of deuterated compound **34** following the CuI procedure for 16 h: Yield: 93%

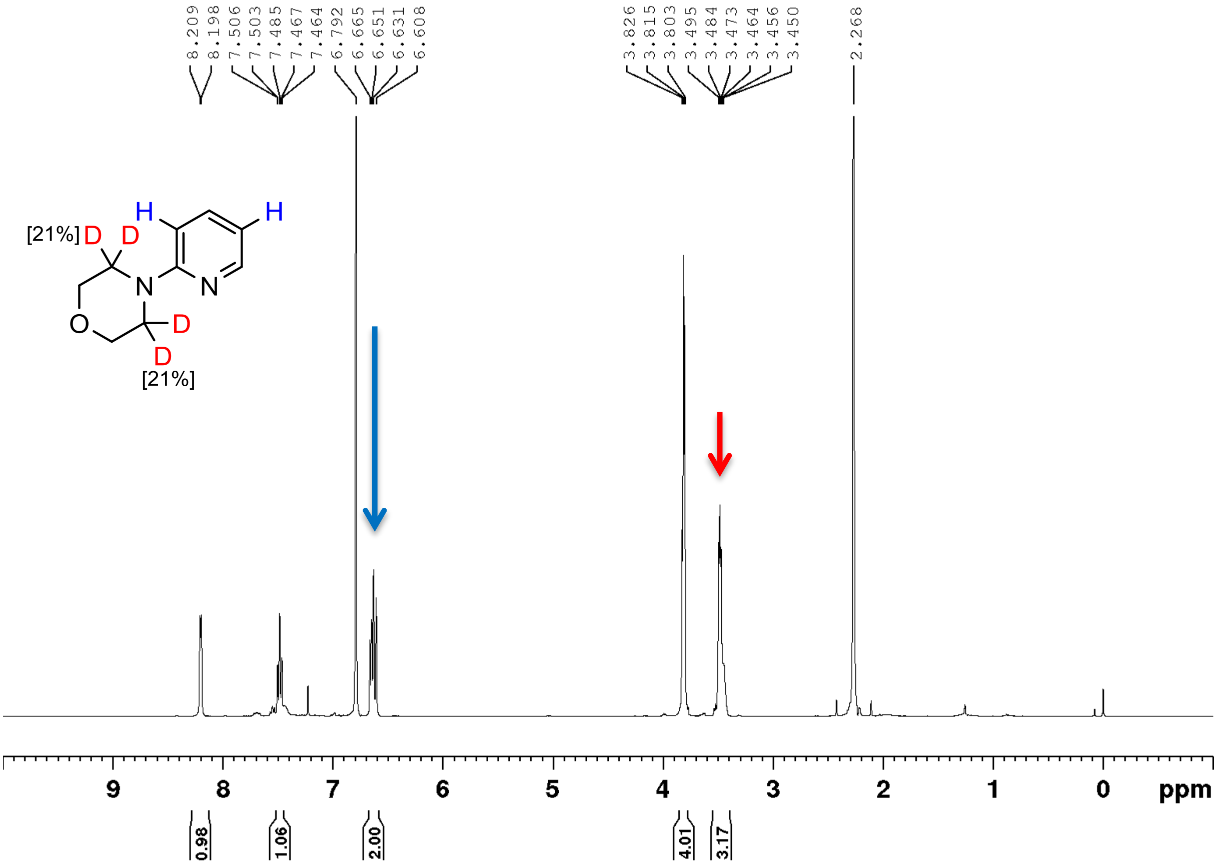

3.5.18 2-Ethylpyridin 35

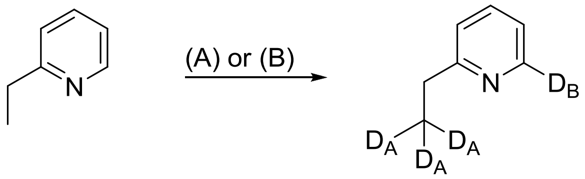

	t	D_A	D_B	Yield
cond. (A)	16 h	n.o.	n.o.	n.o.
	62 h	-	-	-
cond. (B)	16 h	37%	72%	<99%
	62 h	59%	84%	96%

2-Ethylpyridin 35

^{1}H-NMR (400 MHz, THF-d_8) Spectra of pure compound **35**:

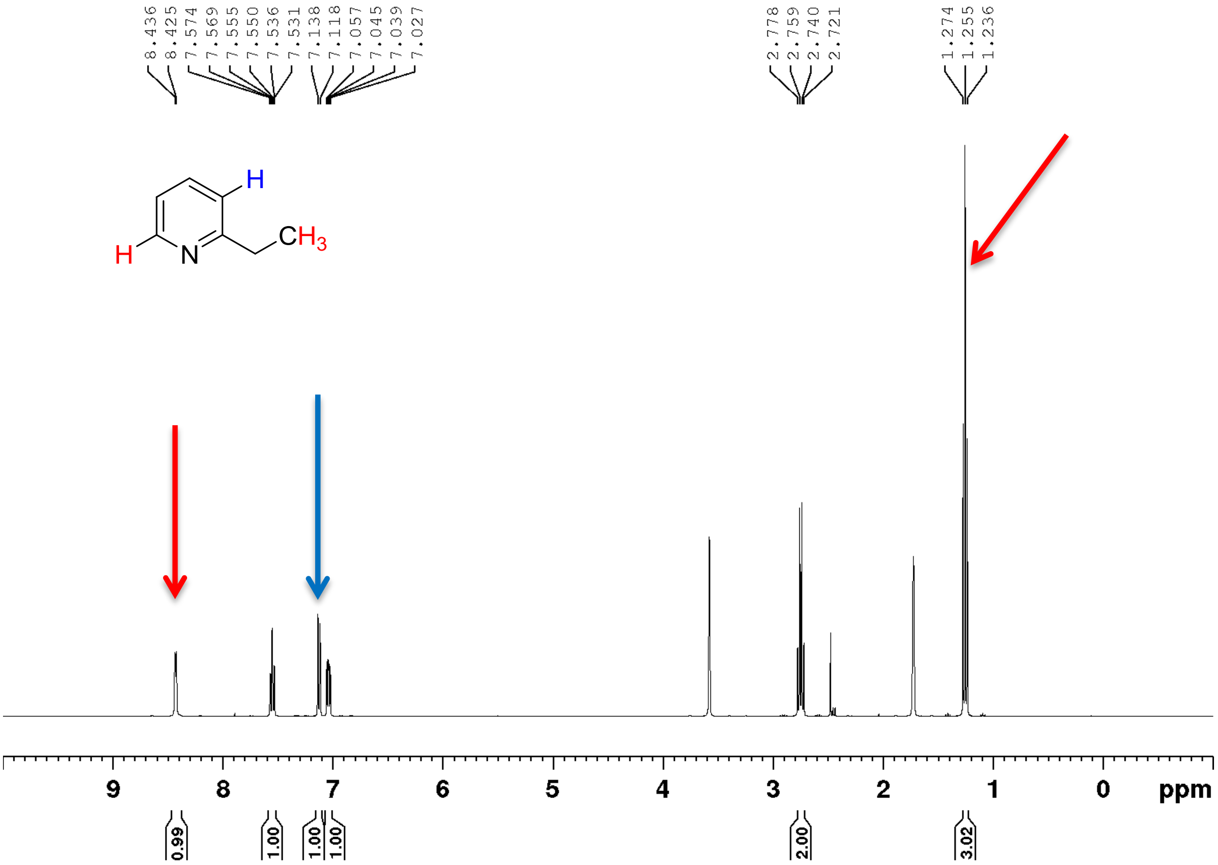

^{1}H-NMR (400 MHz, THF-d$_8$, mesitylene) Spectra of deuterated compound **35** following the KOD/Zn procedure for 16 h: Yield: >99%

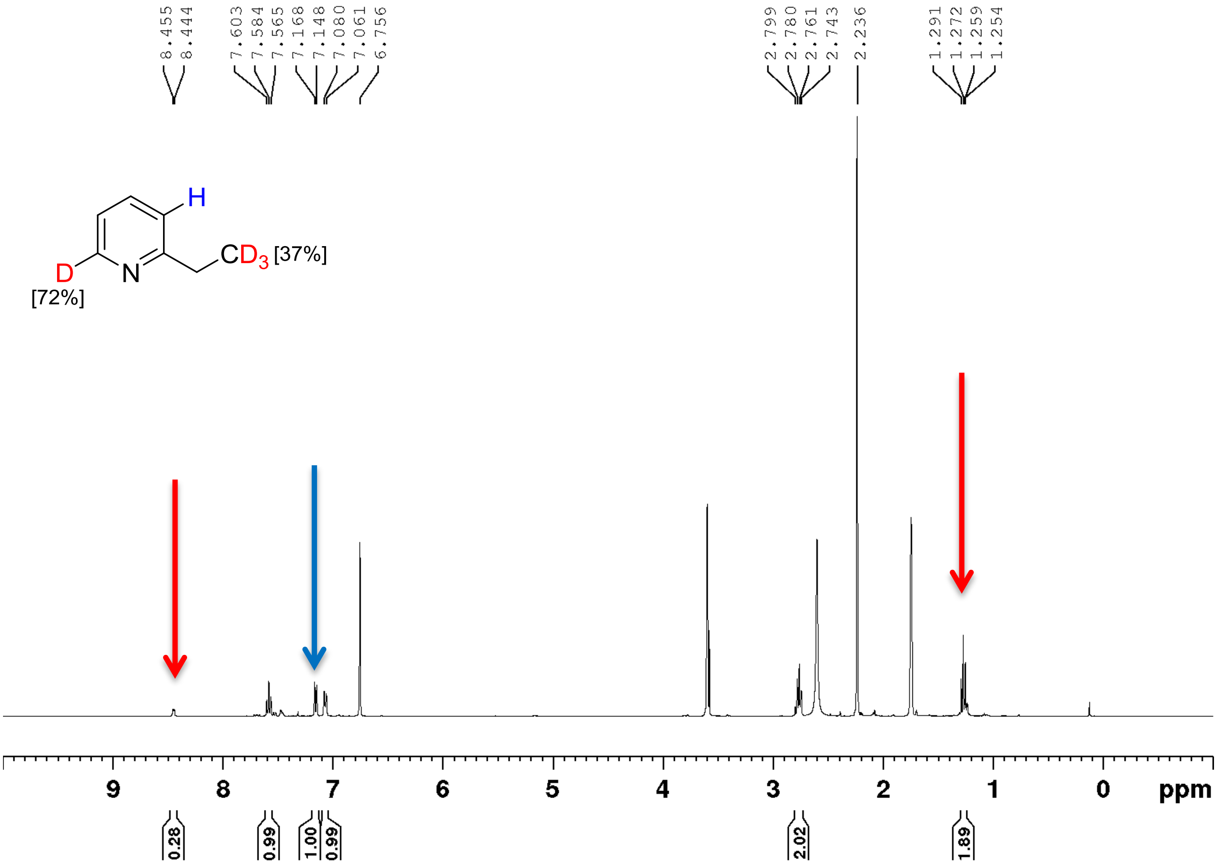

Enlargement of relevant area:

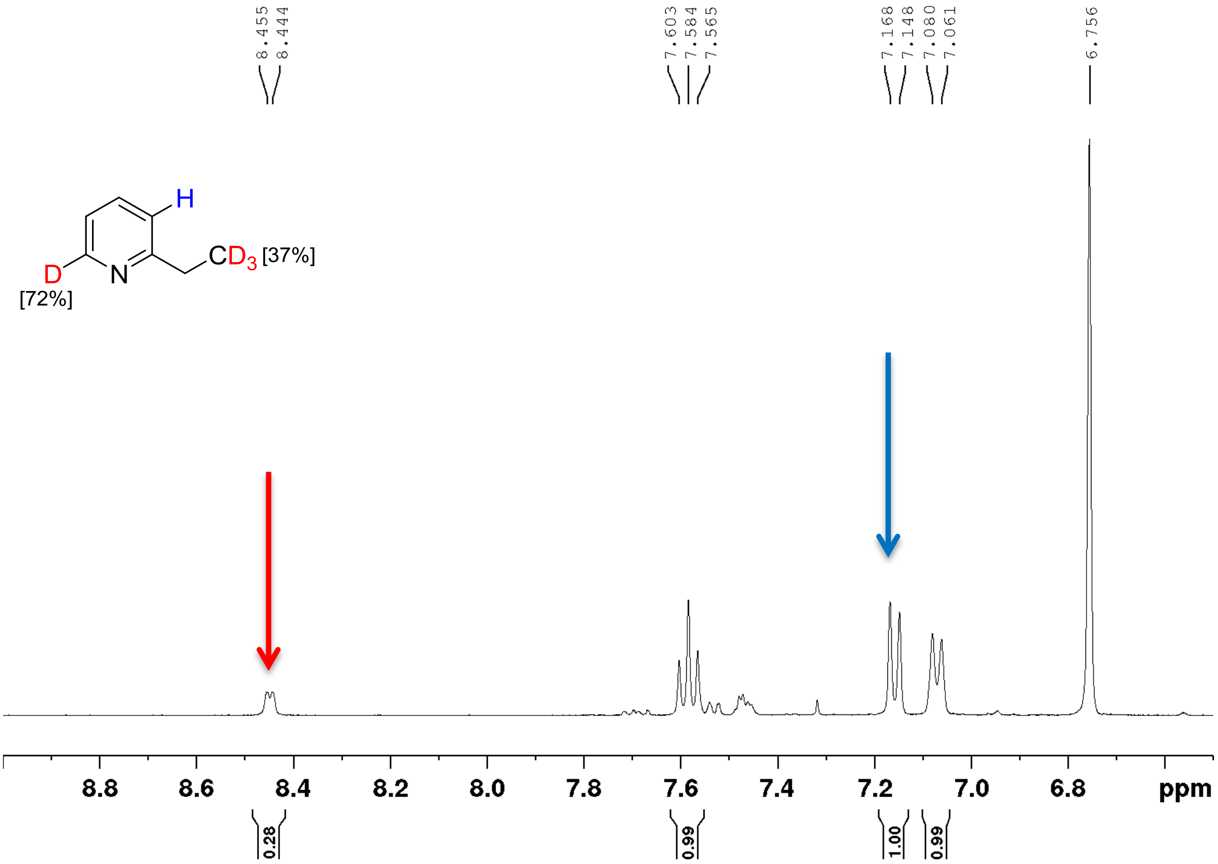

^{1}H-NMR (400 MHz, CD_2Cl_2, mesitylene) Spectra of deuterated compound **35** following the KOD/Zn procedure for 62 h: Yield: 96%

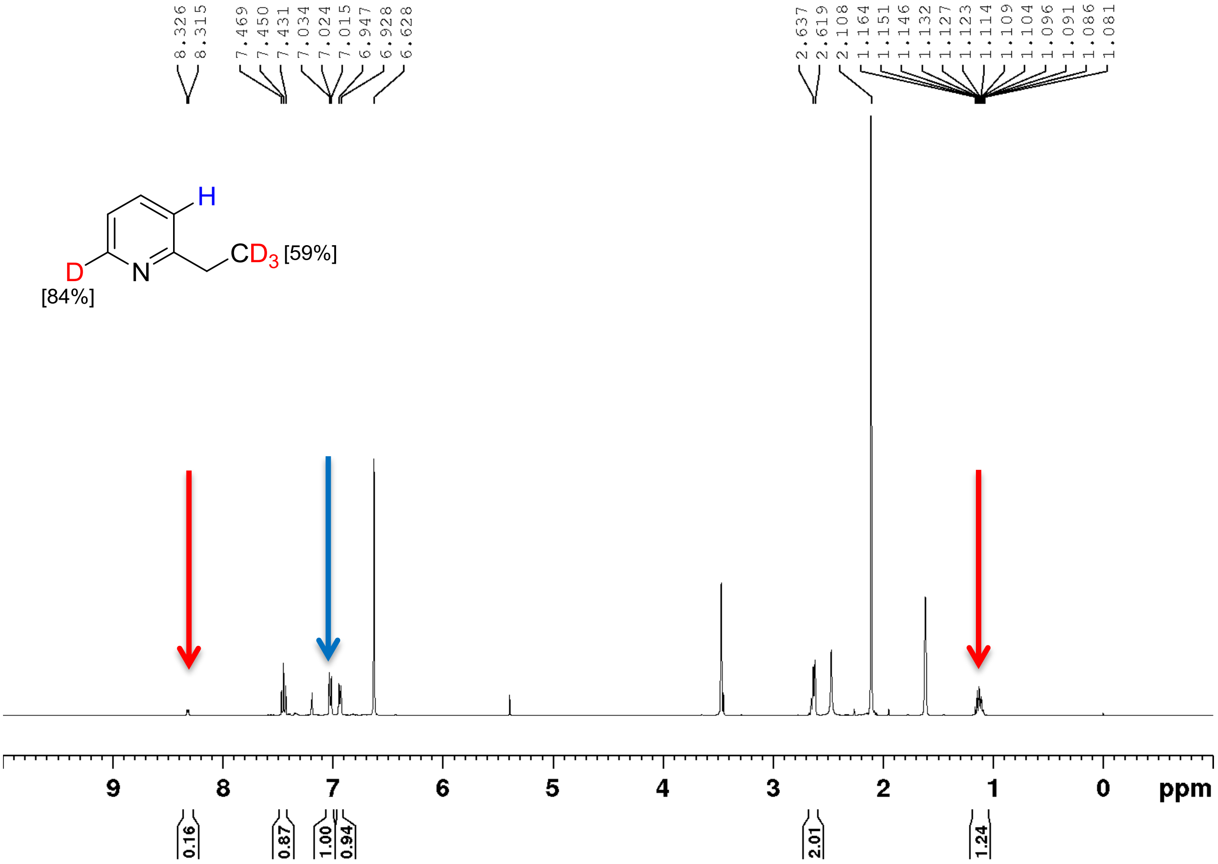

Enlargement of relevant area:

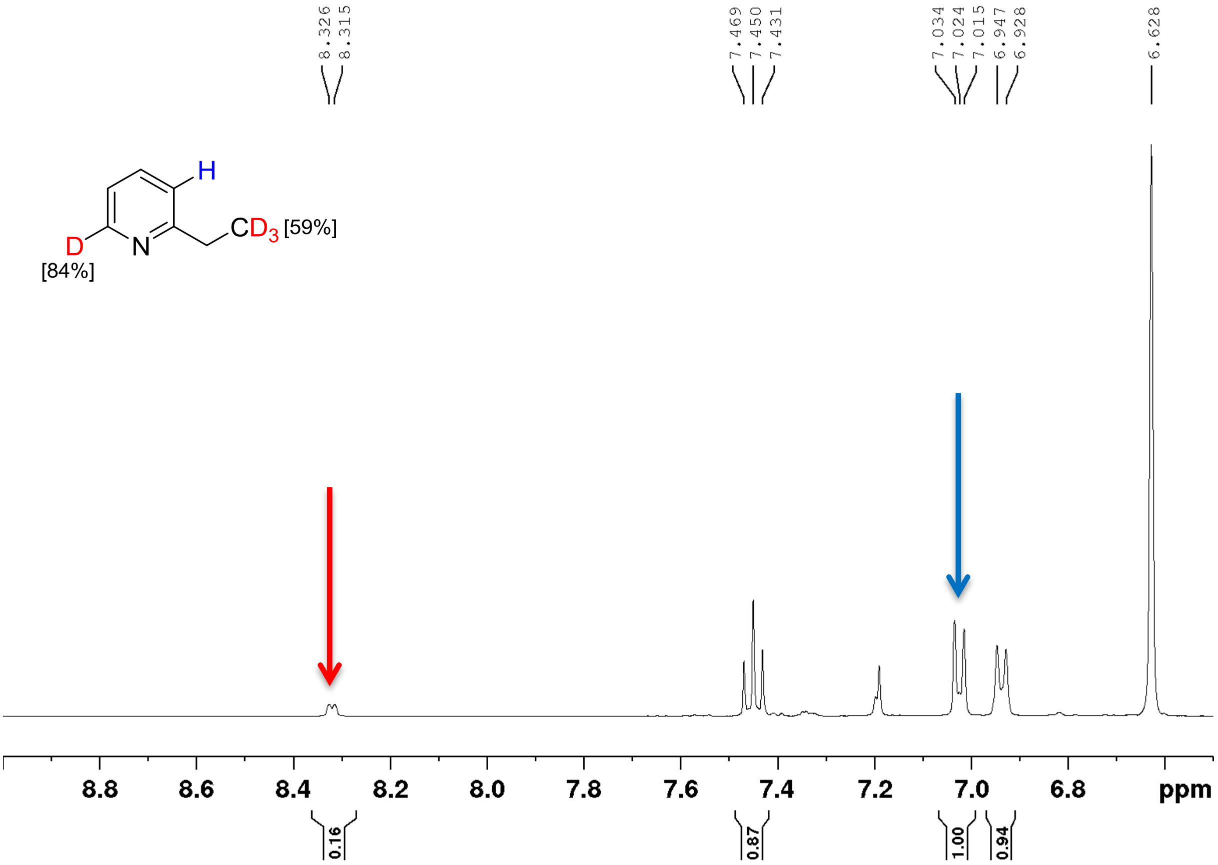

3.5.19 2-(4-(2-phenylethynyl)phenyl)pyridine 36

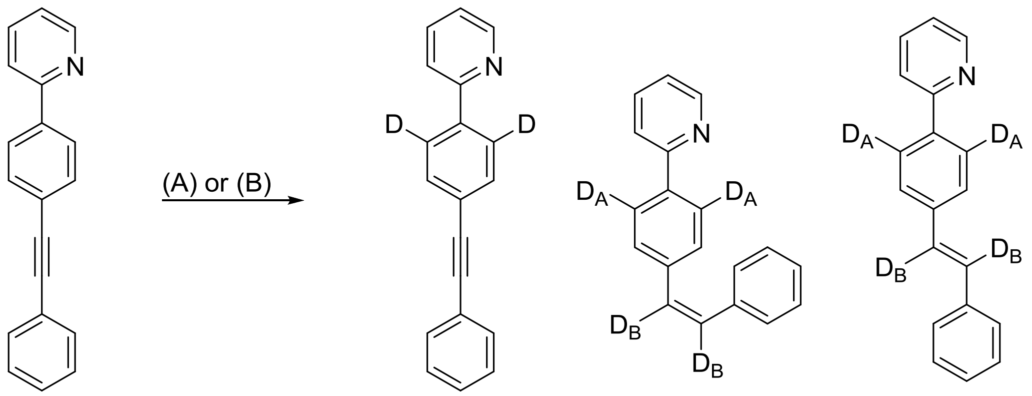

I II III

	I			II (*Z*)			III (*E*)		
	t	D_A	Yield	D_A	D_B	Yield	D_A	D_B	Yield
cond. (A)	16 h	n.o.	n.o.	67%	73%	32%	66%	n.d.	47%
	62 h	n.o.	n.o.	n.o.	n.o.	n.o.	87%	54%	67%
cond. (C)	16 h	n.o.	81%	n.o.	n.o.	n.o.	n.o.	n.o.	n.o.

	62 h	44%	93%	n.o.	n.o.	n.o.	n.o.	n.o.	n.o.

2-(4-(2-phenylethynyl)phenyl)pyridine 36 (I)
^{1}H-NMR (400 MHz, CD_2Cl_2) Spectra of pure compound **36**:

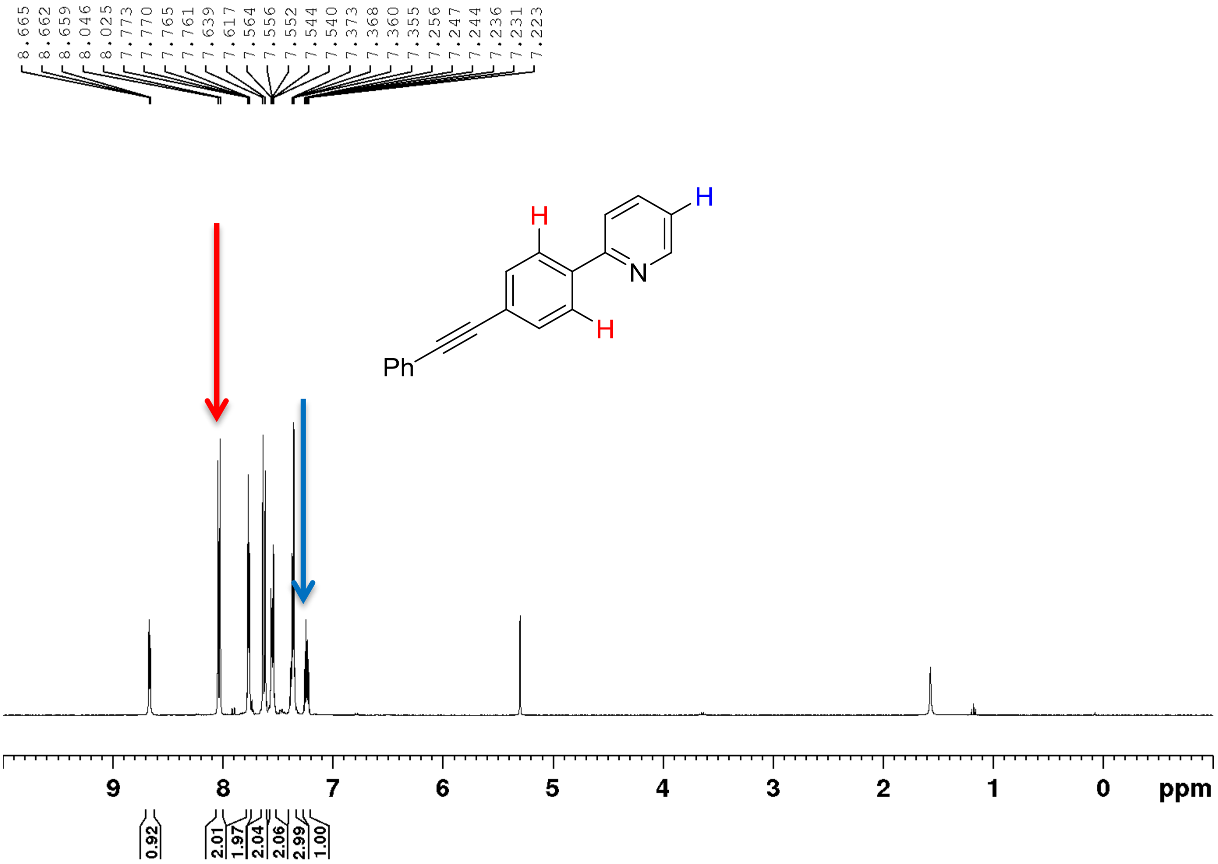

Enlargement of relevant area:

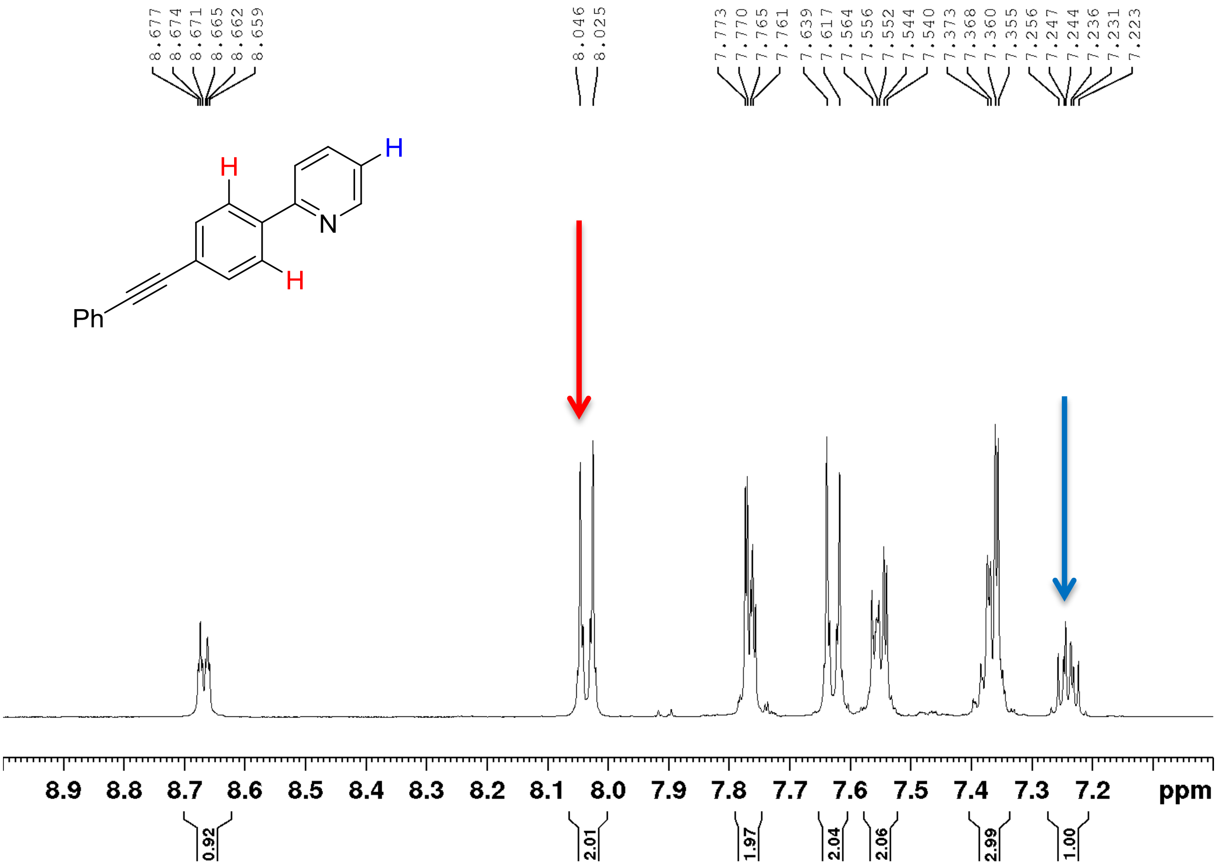

^{1}H-NMR (400 MHz, CD_2Cl_2, mesitylene) Spectra of deuterated compound **36** following the KOD procedure for 16 h: Yield: 81%

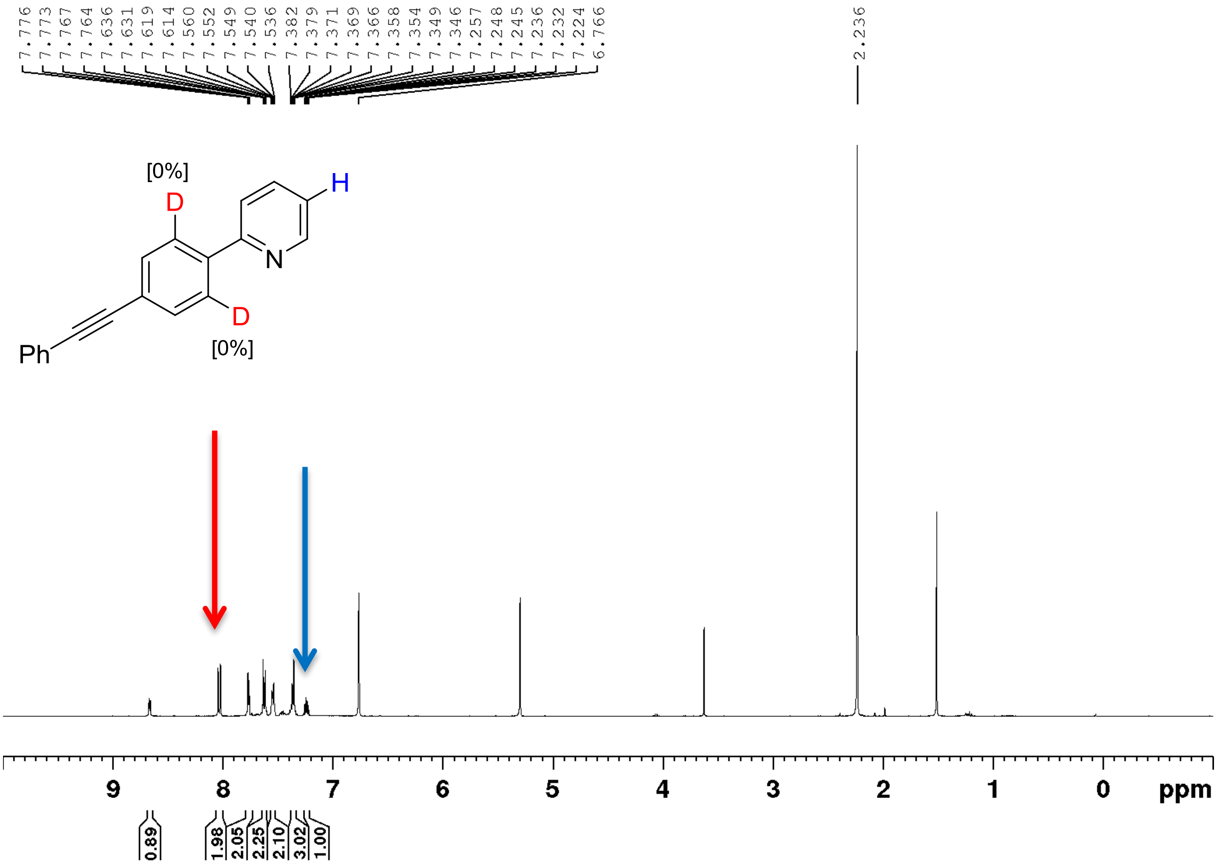

^{1}H-NMR (400 MHz, CD_2Cl_2, mesitylene) Spectra of deuterated compound **36** following the KOD procedure for 62 h: 93%

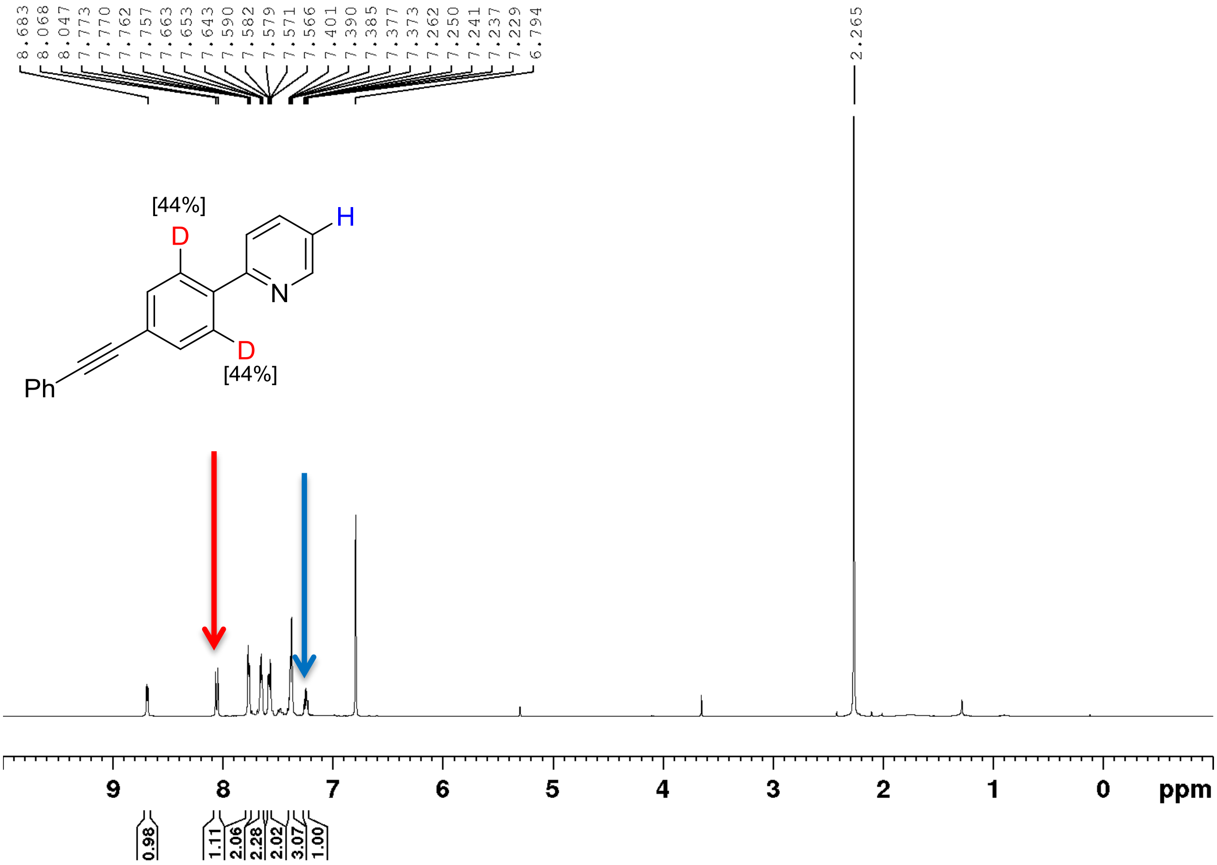

Enlargement of relevant area:

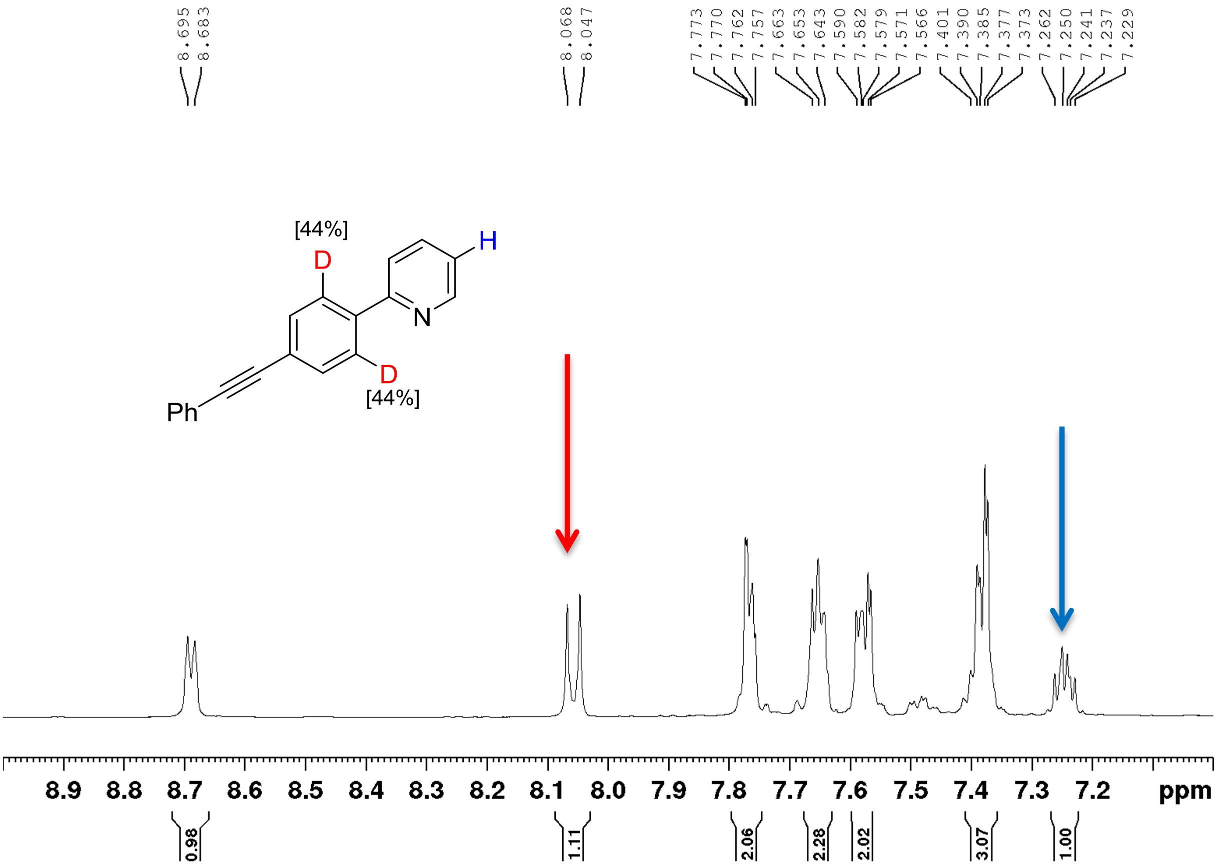

(*Z*)-2-(4-styrylphenyl)pyridine (II)

^{1}H-NMR (400 MHz, CD_2Cl_2) Spectra of pure compound:

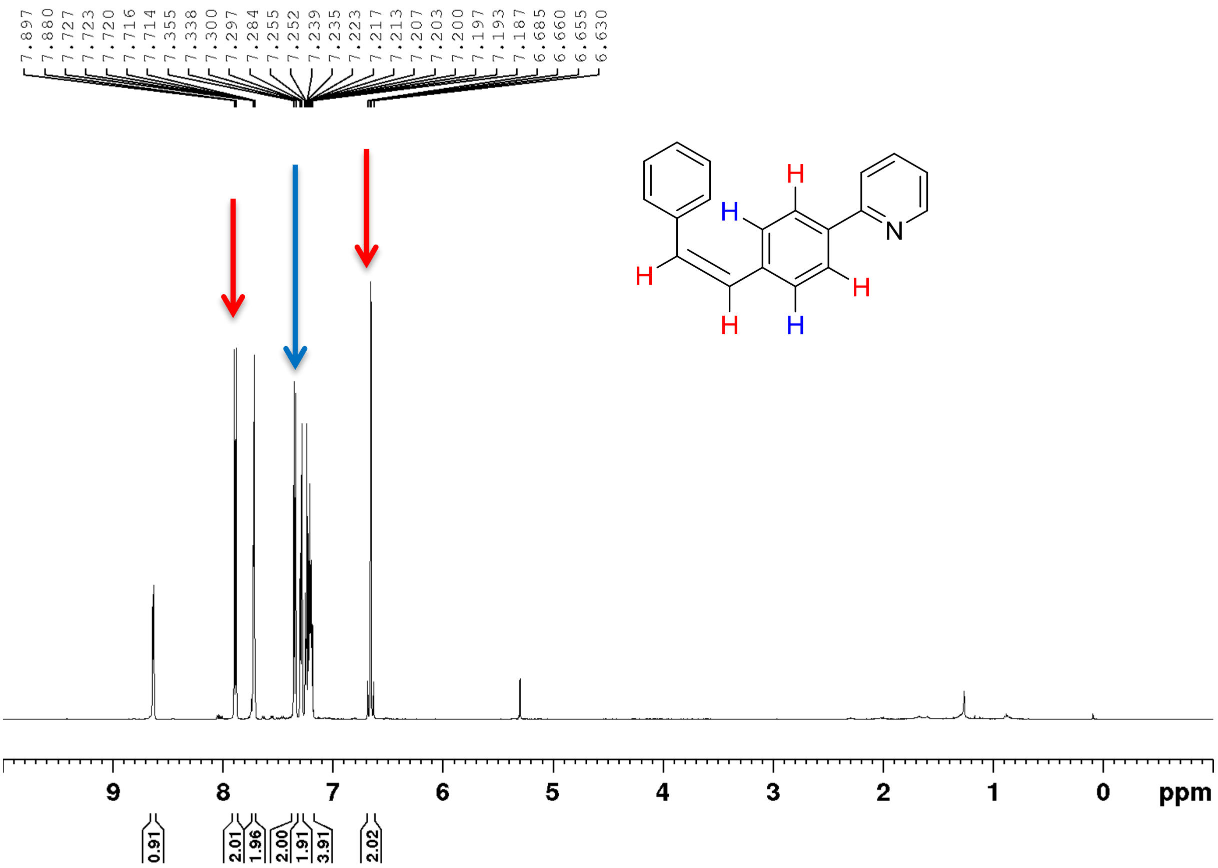

Enlargement of relevant area:

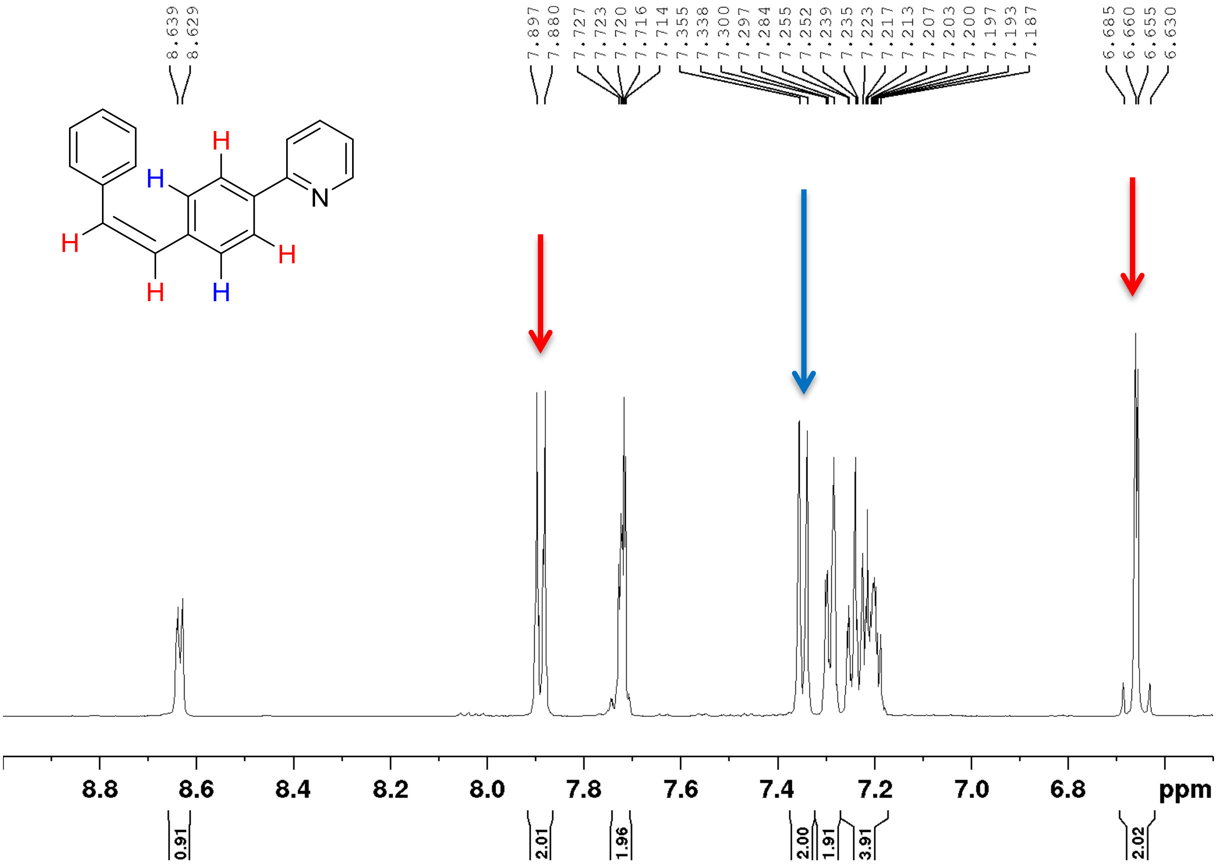

^{1}H-NMR (400 MHz, $CDCl_3$) Spectra of deuterated compound following the CuI procedure for 16 h: Yield: 32% (*Z*/*E*: 1:1.5)

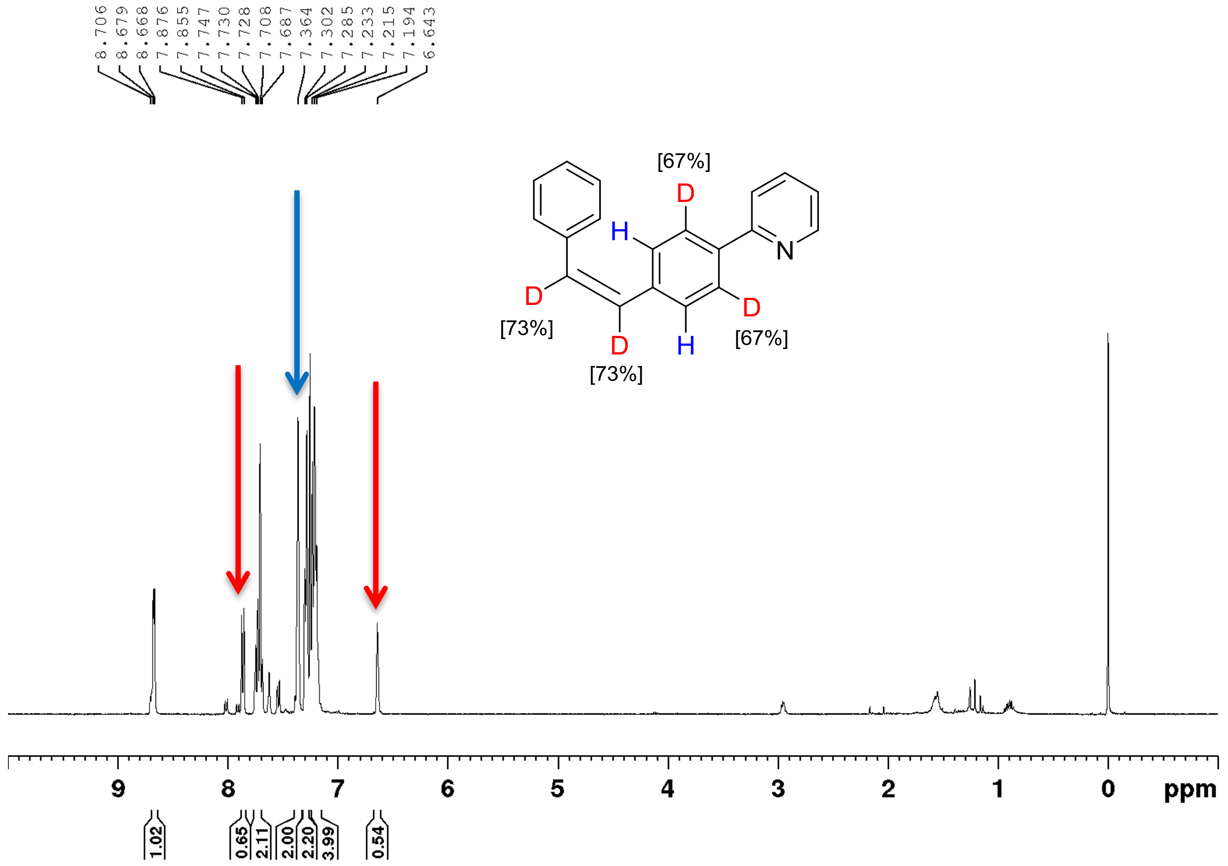

Enlagement of relevant area:

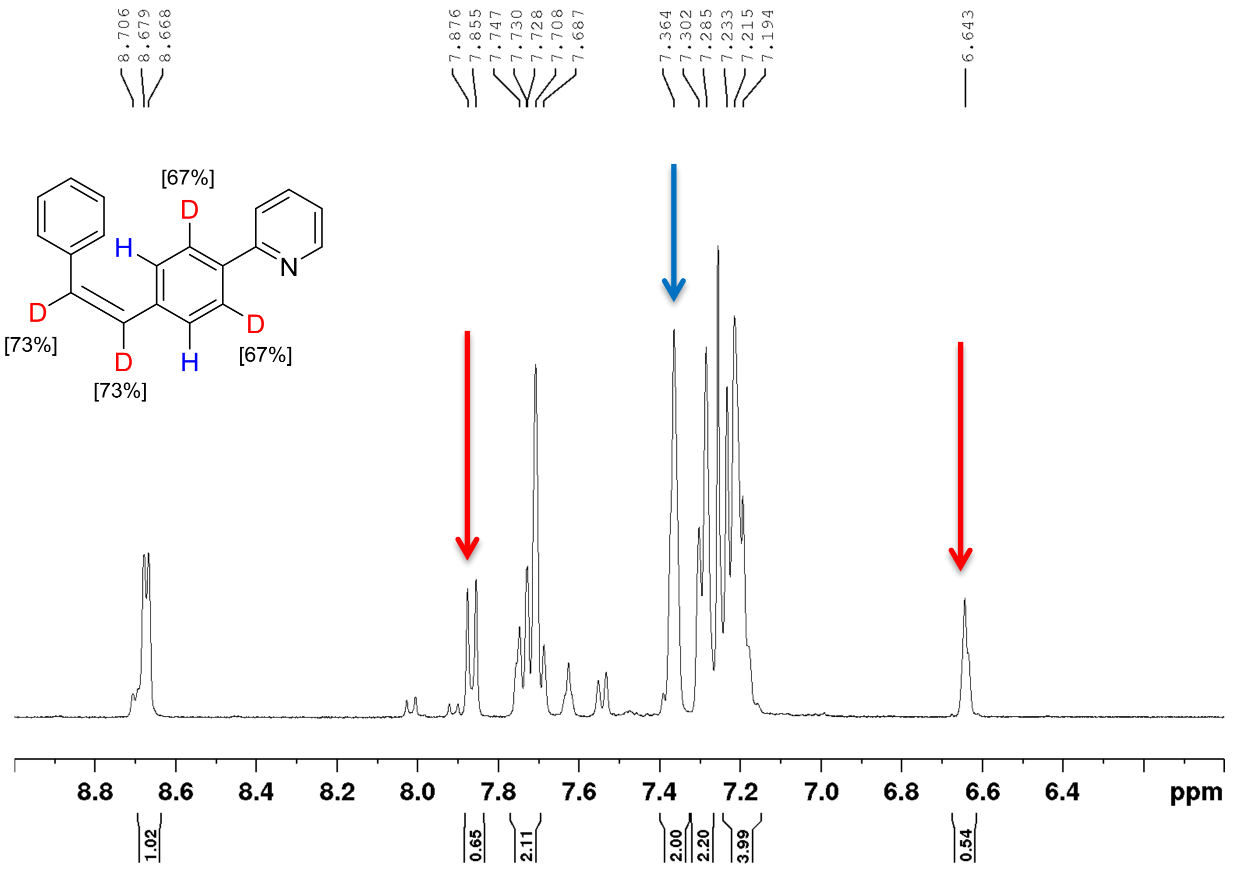

(*E*)-2-(4-styrylphenyl)pyridine 41 (III)

^{1}H-NMR (400 MHz, CD_2Cl_2) Spectra of pure compound **41**:

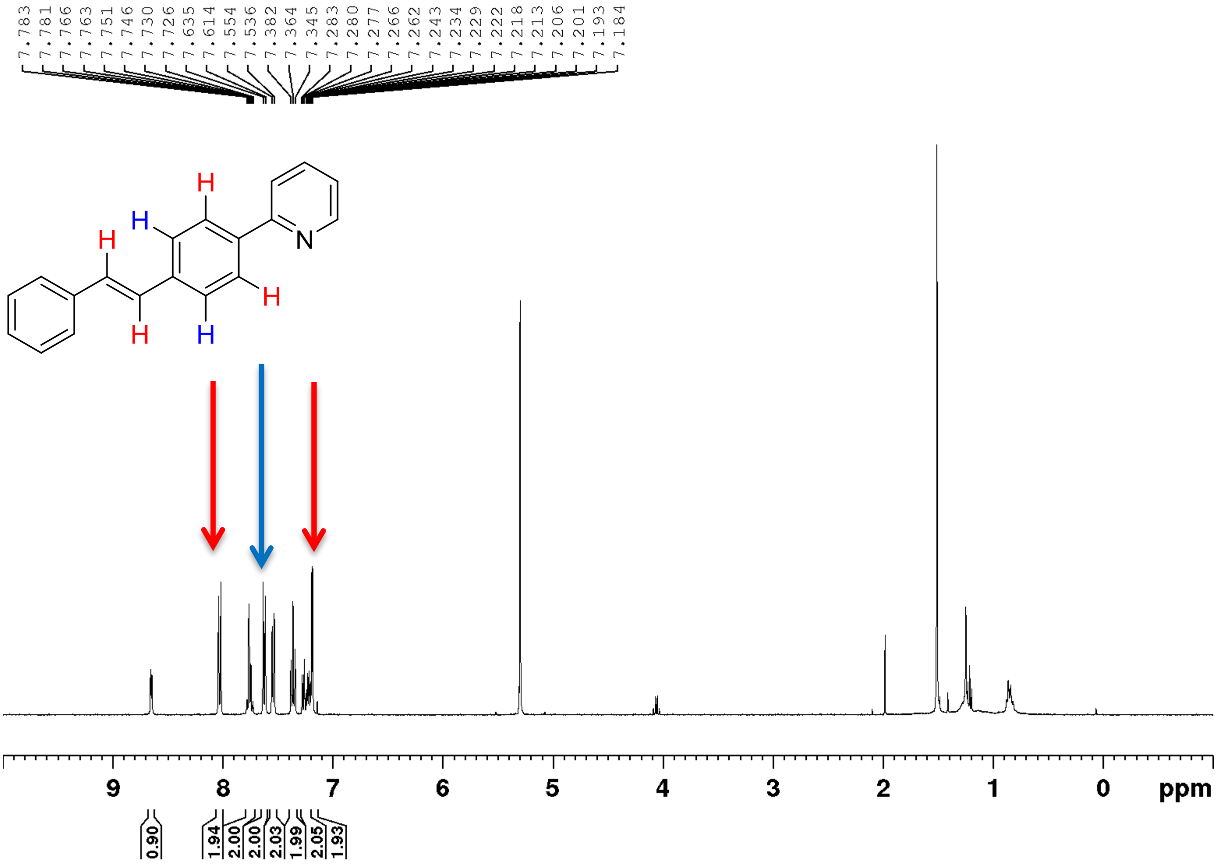

Enlargement of relevant area:

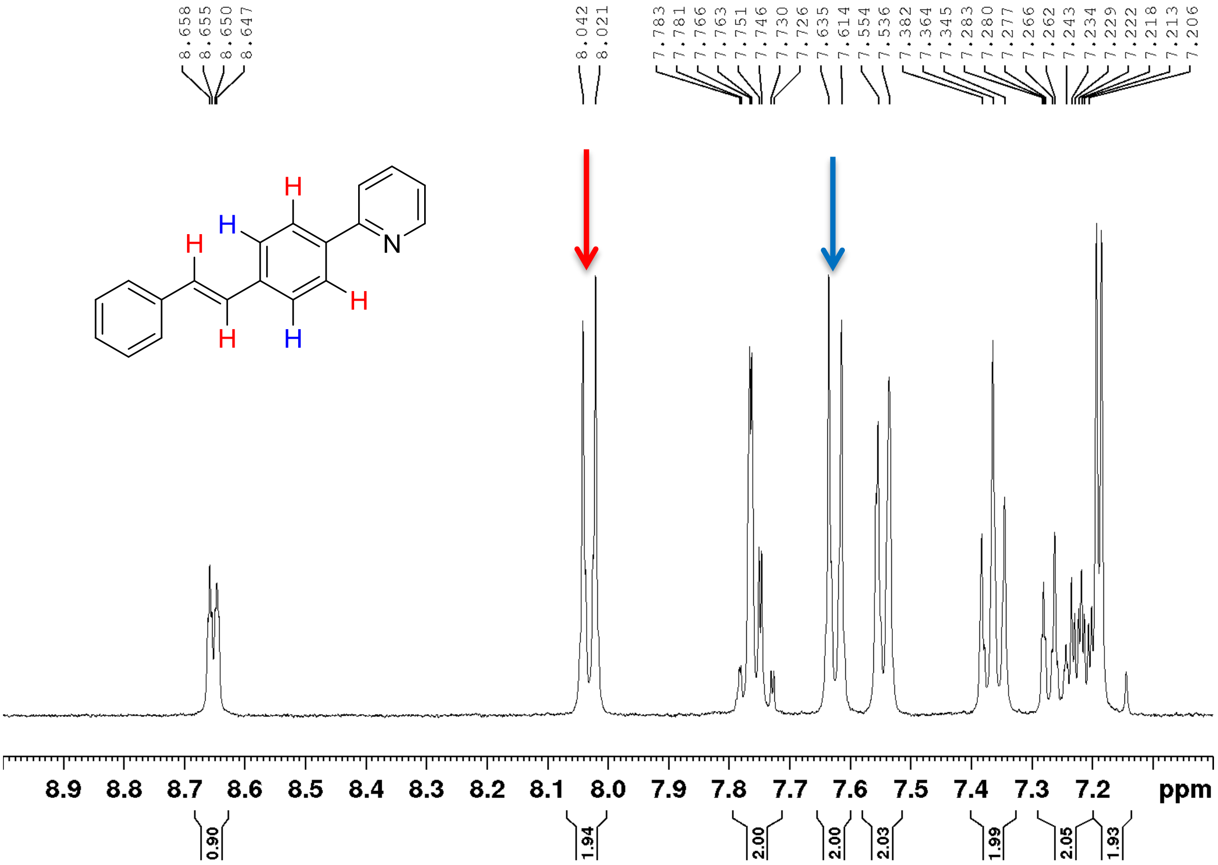

^{1}H-NMR (400 MHz, $CDCl_3$) Spectra of deuterated compound **41** following the CuI procedure for 16 h: Yield: 47%

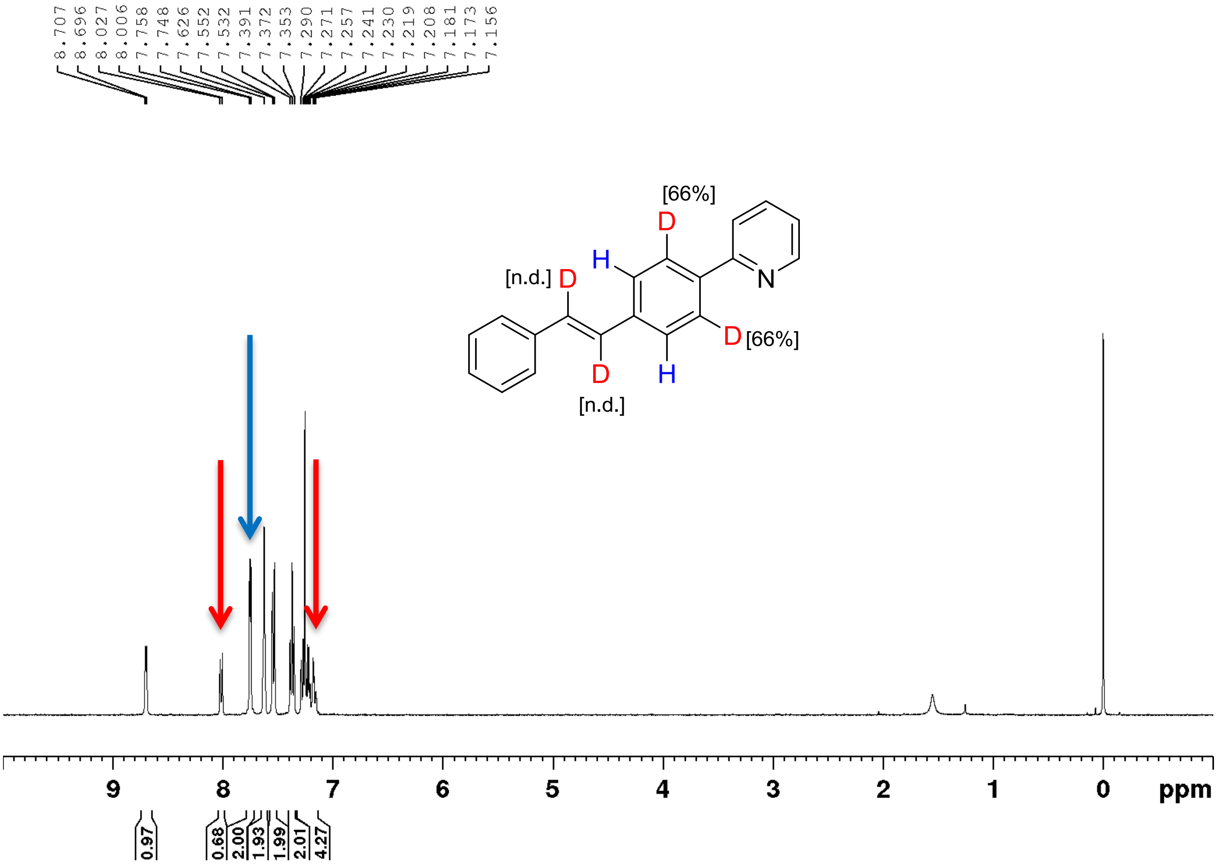

Enlargement of relevant area:

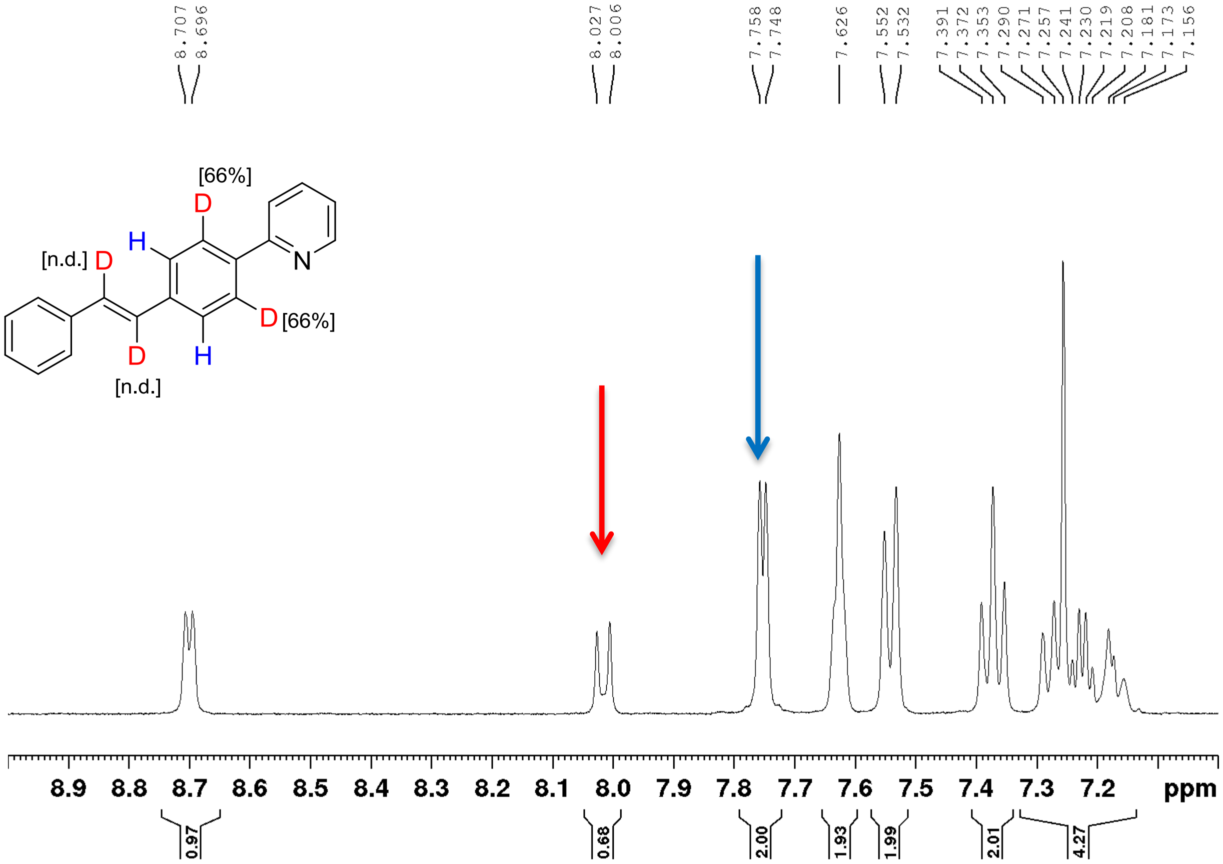

^{1}H-NMR (400 MHz, $CDCl_3$) Spectra of deuterated compound **41** following the CuI procedure for 62 h: Yield: 67%

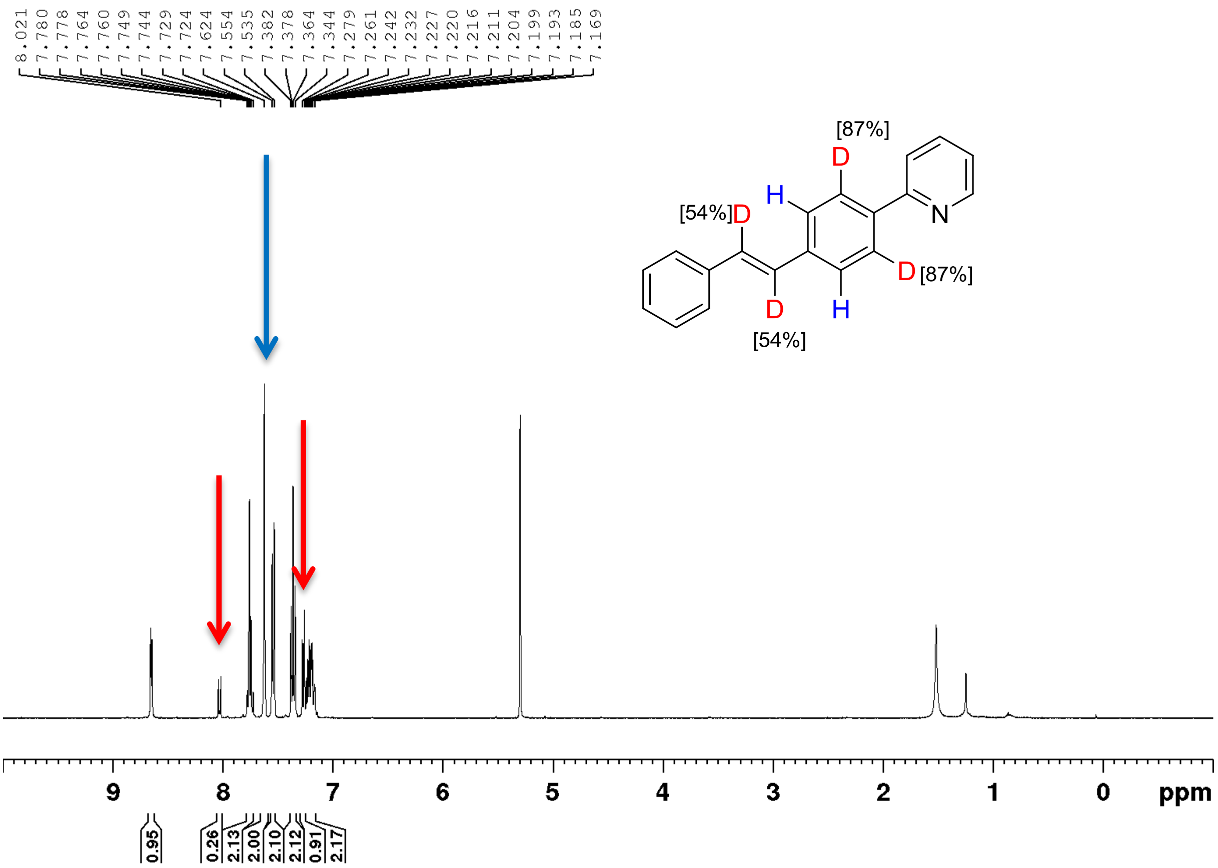

Enlargement of relevant area:

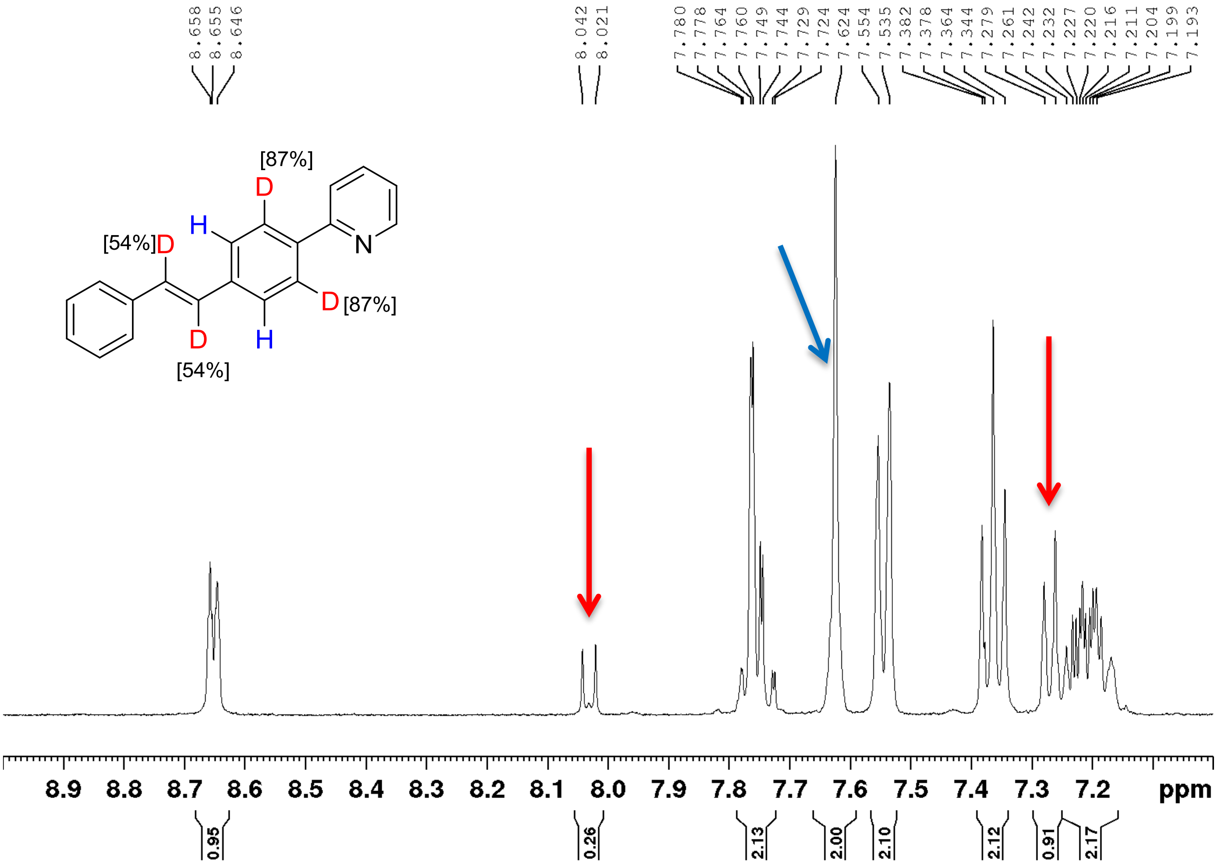

3.5.20 2-(3-(phenylethynyl)phenyl)pyridine 37

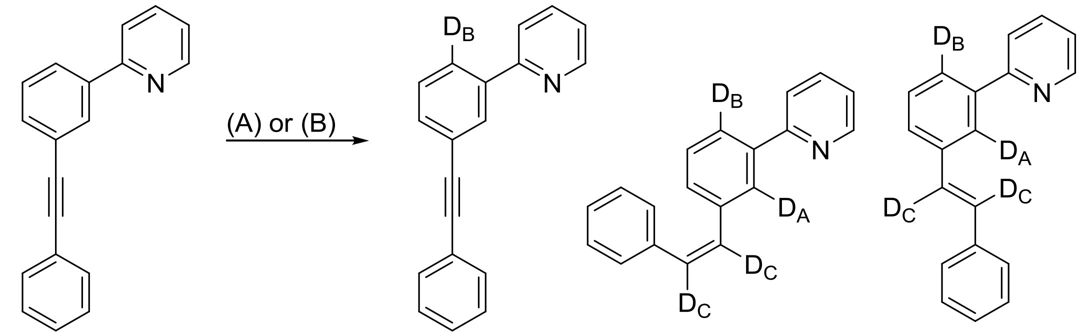

I **II** **III**

		**I**		**II** (*Z*)				**III** (*E*)			
	t	D_B	Yield	D_A	D_B	D_C	Yield	D_A	D_B	D_C	Yield
cond. (A)	16 h	n.o.	n.o.	34%	60%	66%	51%	39%	59%	24%	20%
	62 h	n.o.	n.o.	n.d.	n.d.	71%	20%	61%	89%	25%	50%
cond. (C)	16 h	n.o.	94%	n.o.	n.o.	n.o.	n.o.	n.o.	n.o.	n.o.	n.o.
	62 h	44%	92%	n.o.	n.o.	n.o.	n.o.	n.o.	n.o.	n.o.	n.o.

2-(3-(phenylethynyl)phenyl)pyridine 37 (I)

^{1}H-NMR (300 MHz, CD_2Cl_2) Spectra of pure compound **37**:

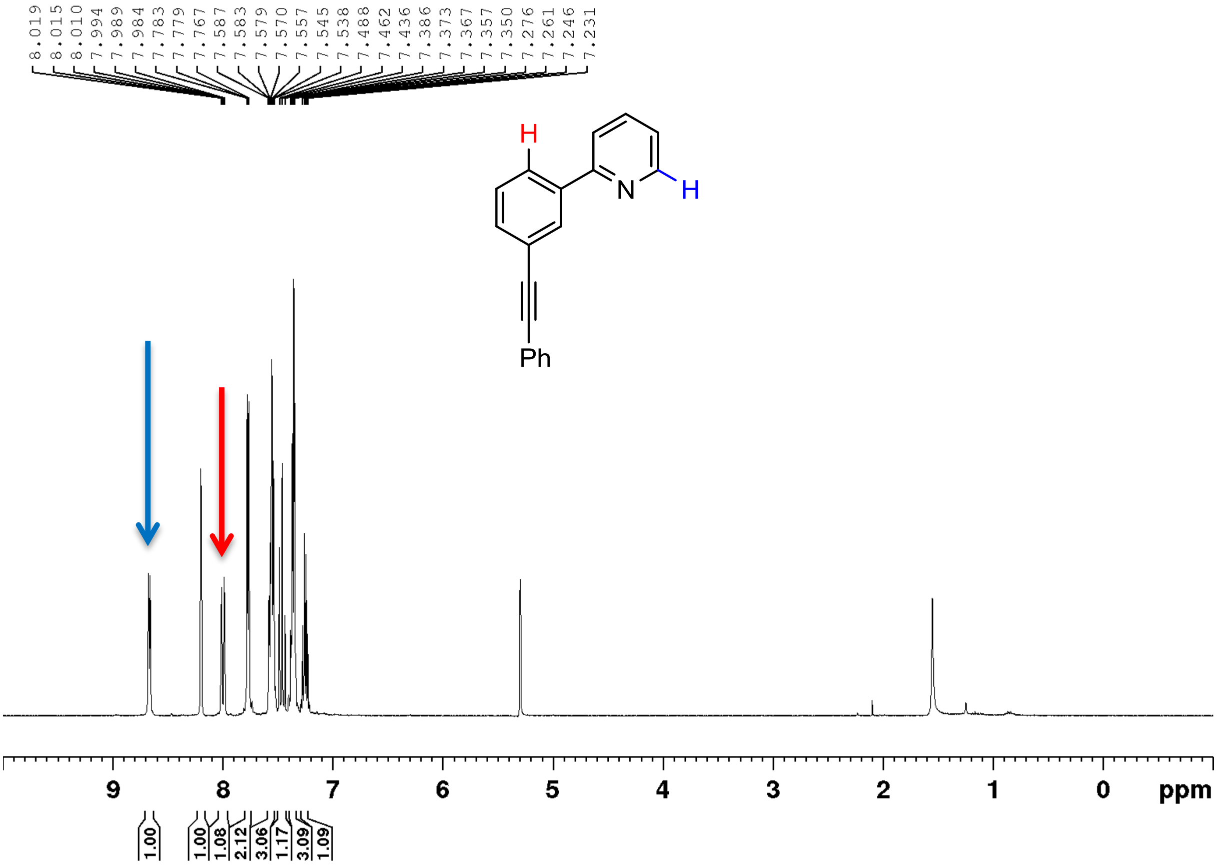

Enlargement of relevant area:

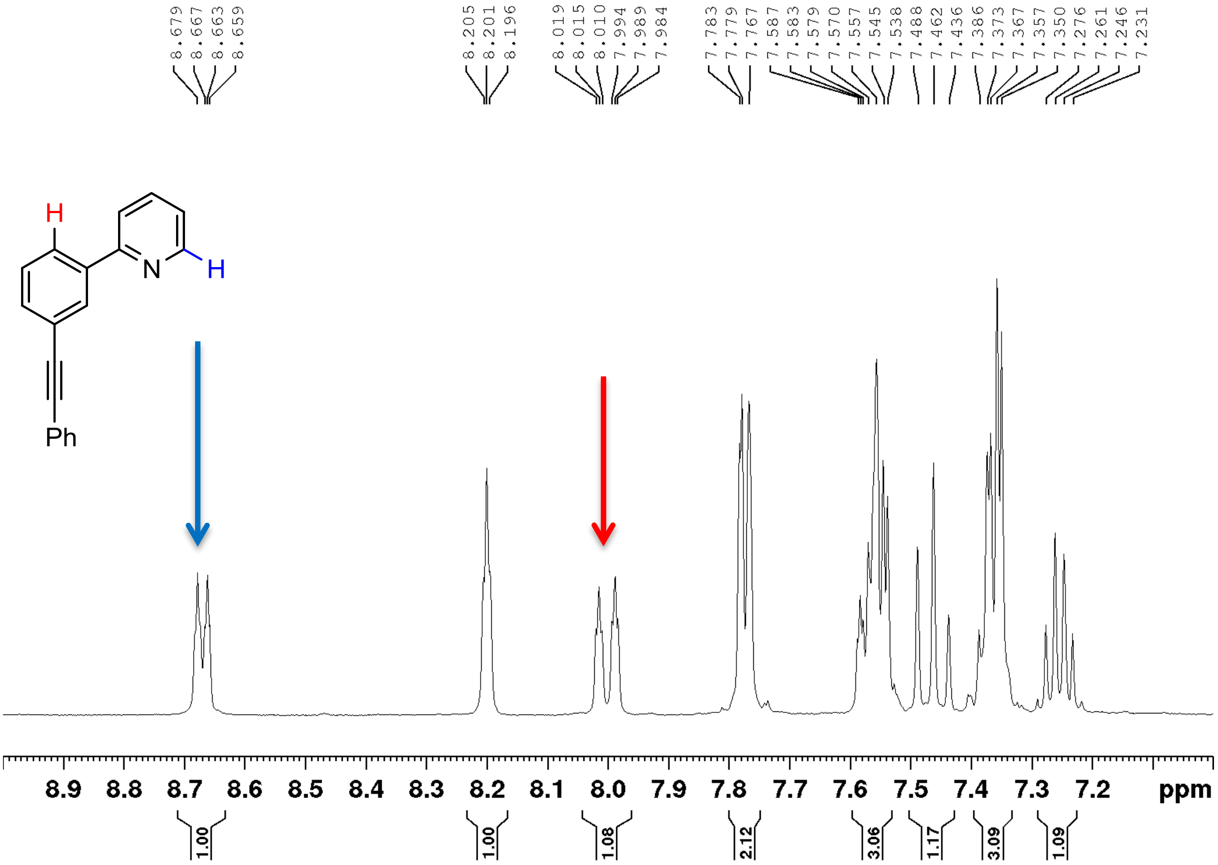

^{1}H-NMR (400 MHz, CD_2Cl_2, mesitylene) Spectra of deuterated **37** compound following the KOD procedure for 16 h: Yield: 94%

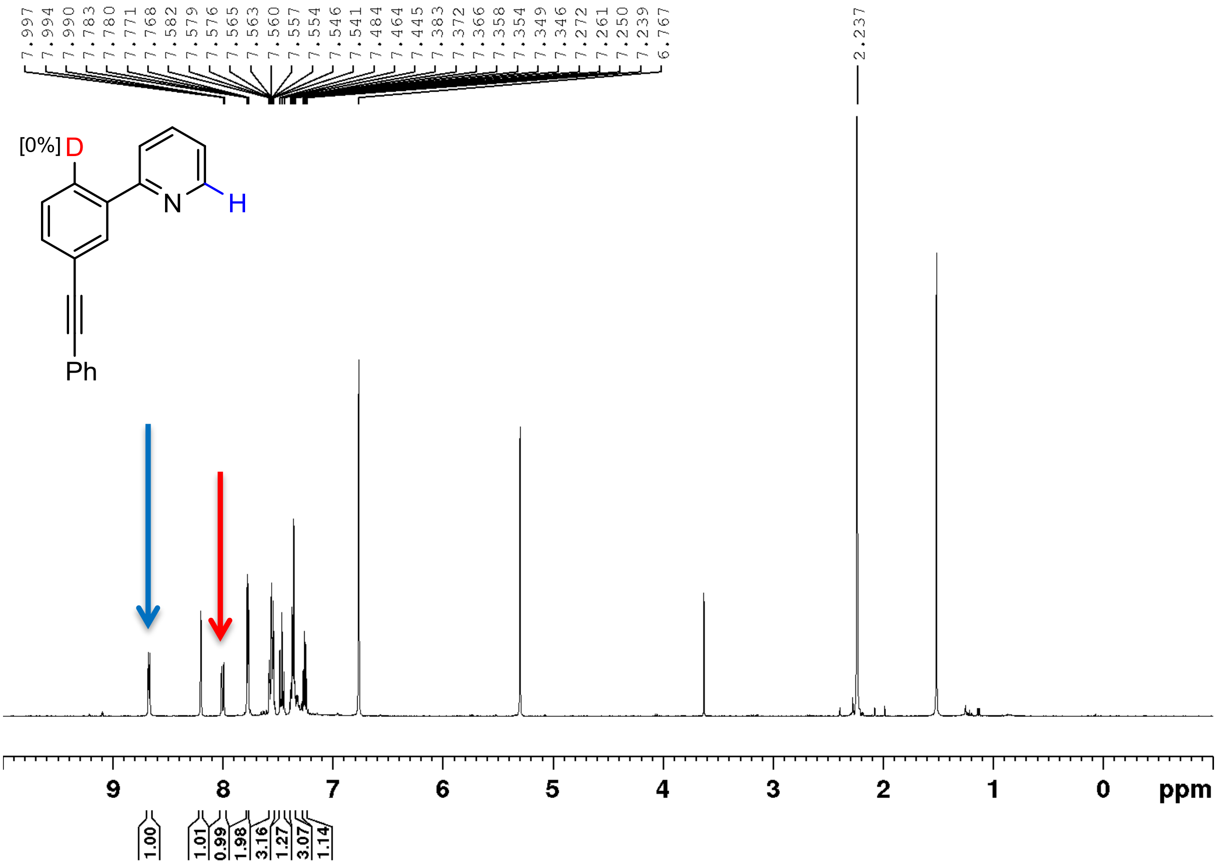

^{1}H-NMR (400 MHz, CD_2Cl_2, mesitylene) Spectra of deuterated **37** compound following the KOD procedure for 62 h: Yield: 92%

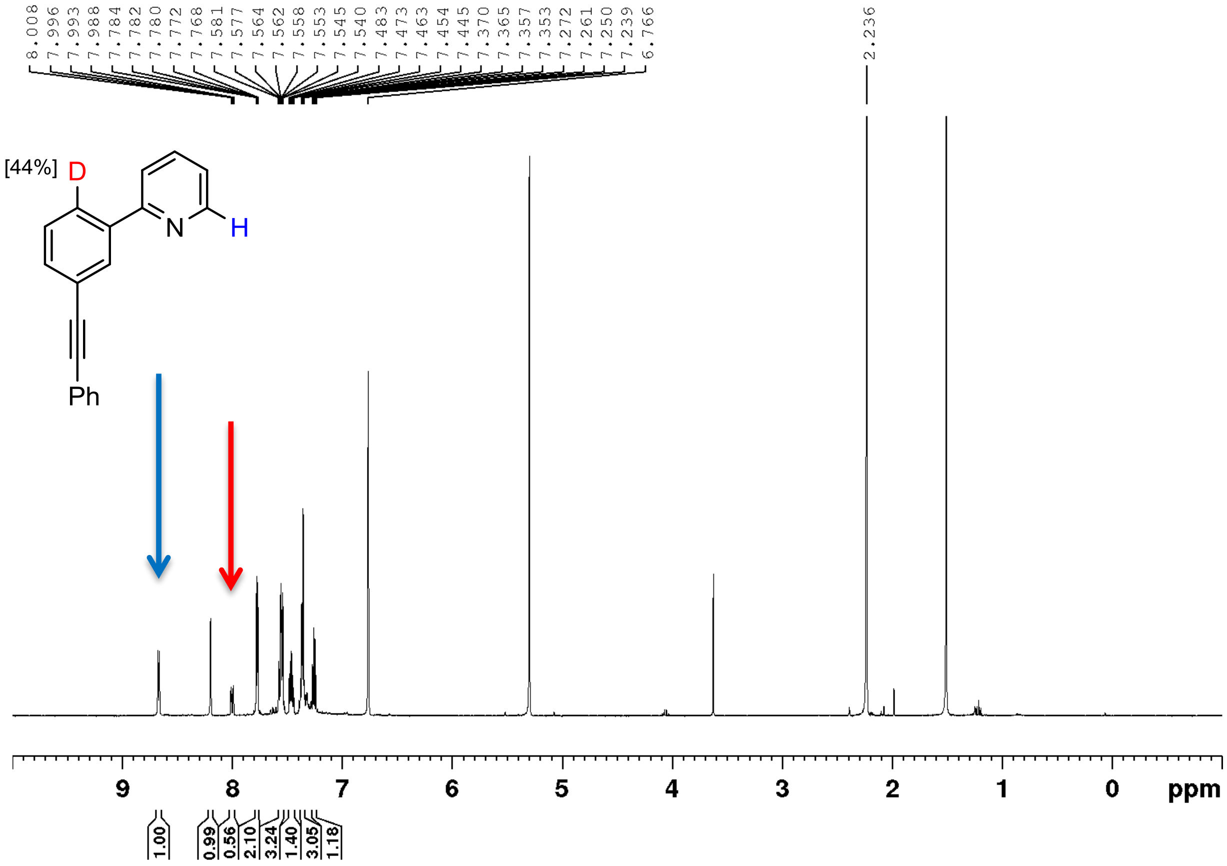

Enlargement of relevant area:

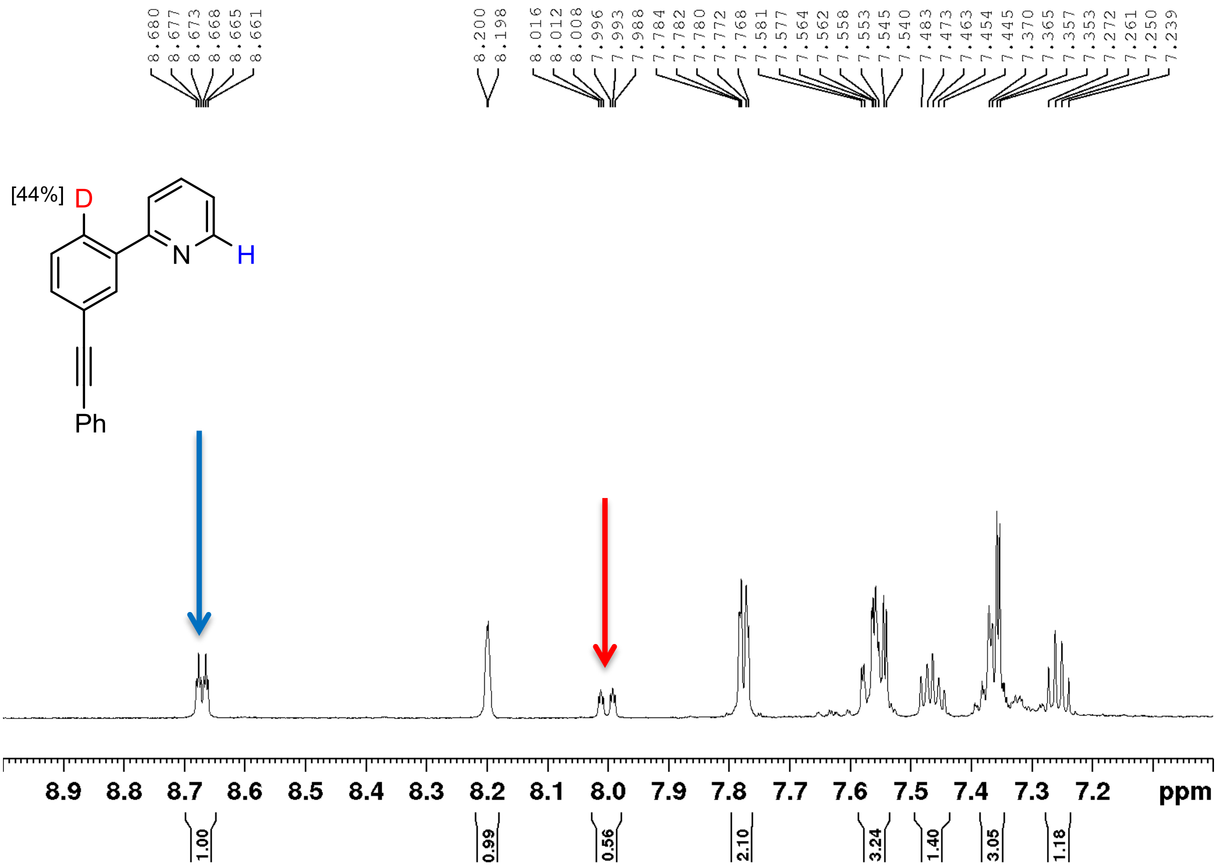

(*Z*)-2-(3-styrylphenyl)pyridine (II)

^{1}H-NMR (400 MHz, CD_2Cl_2) Spectra of pure compound:

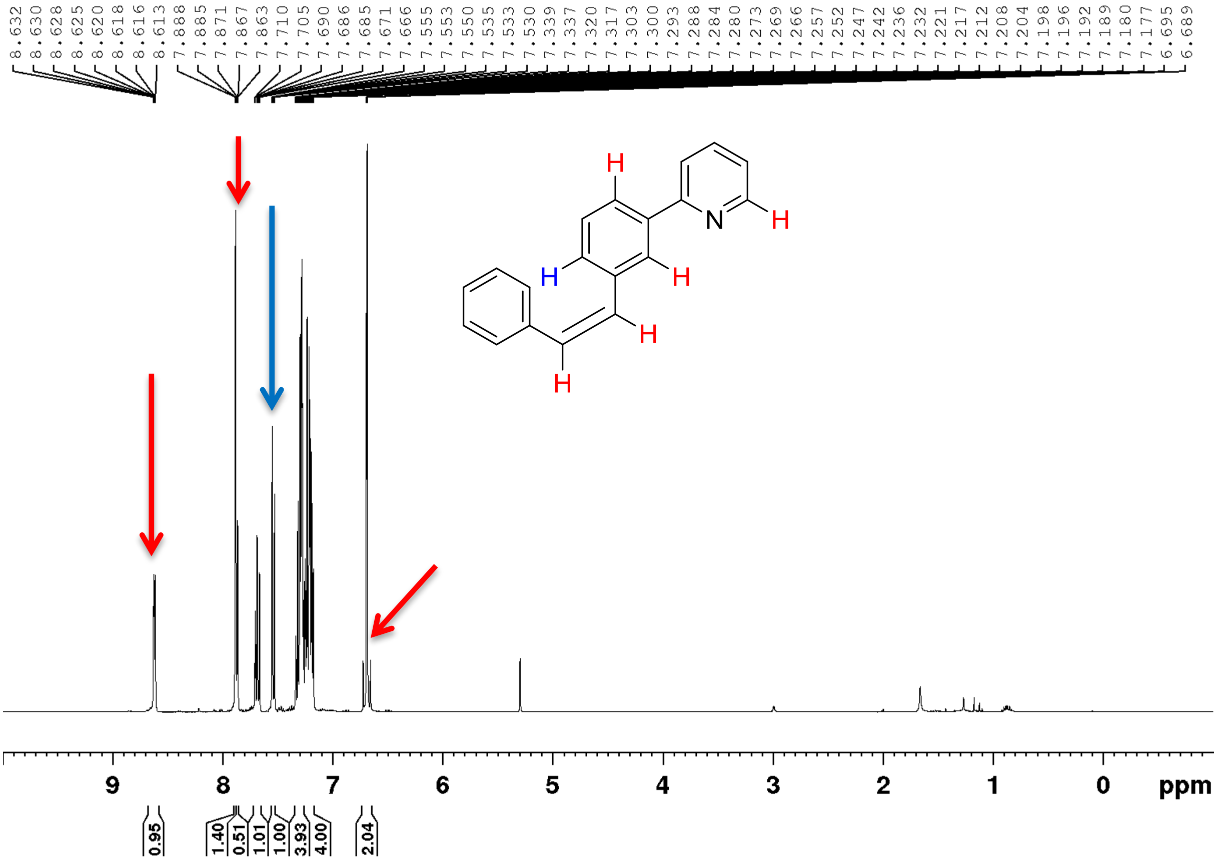

Enlargement of relevant area:

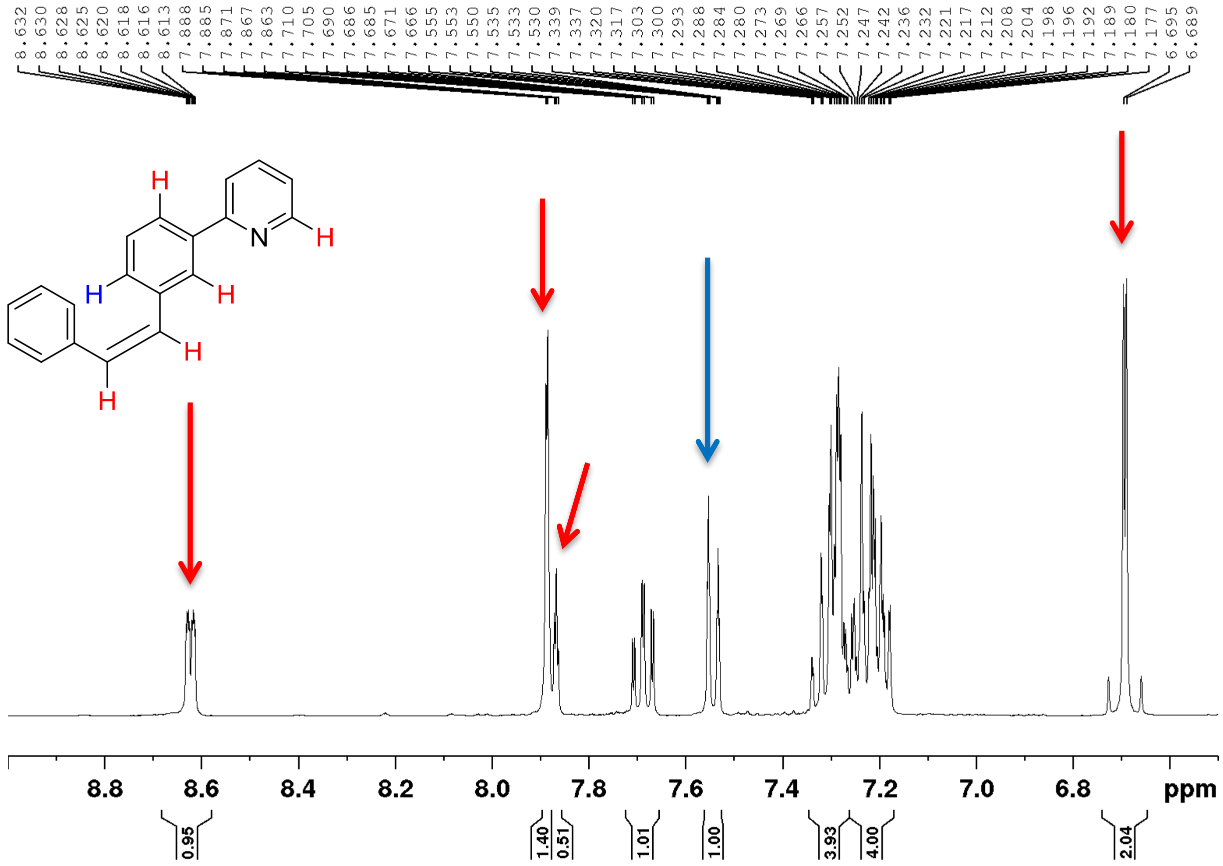

^{1}H-NMR (400 MHz, CD_2Cl_2) Spectra of deuterated compound following the CuI procedure for 16 h: Yield: 51% (*Z*/*E*: 2.5:1)

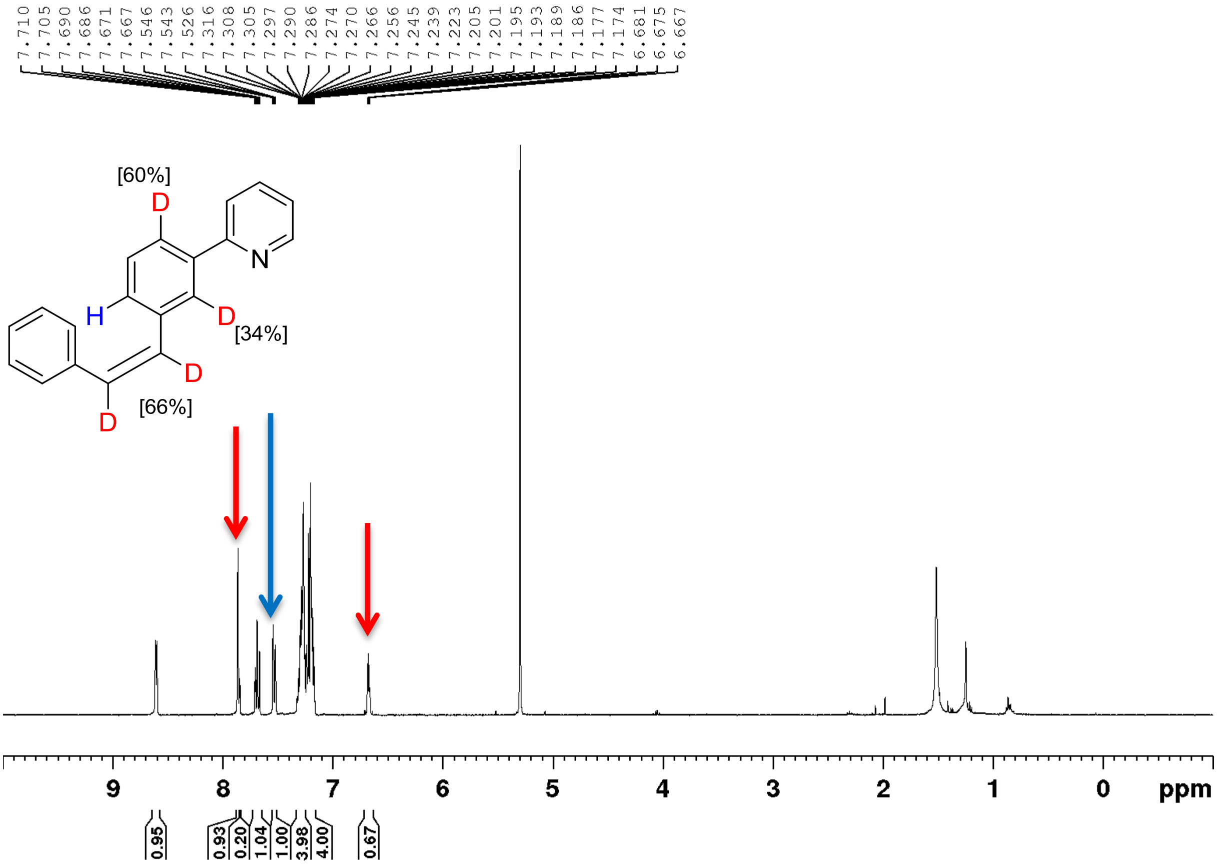

Enlargement of relevant area:

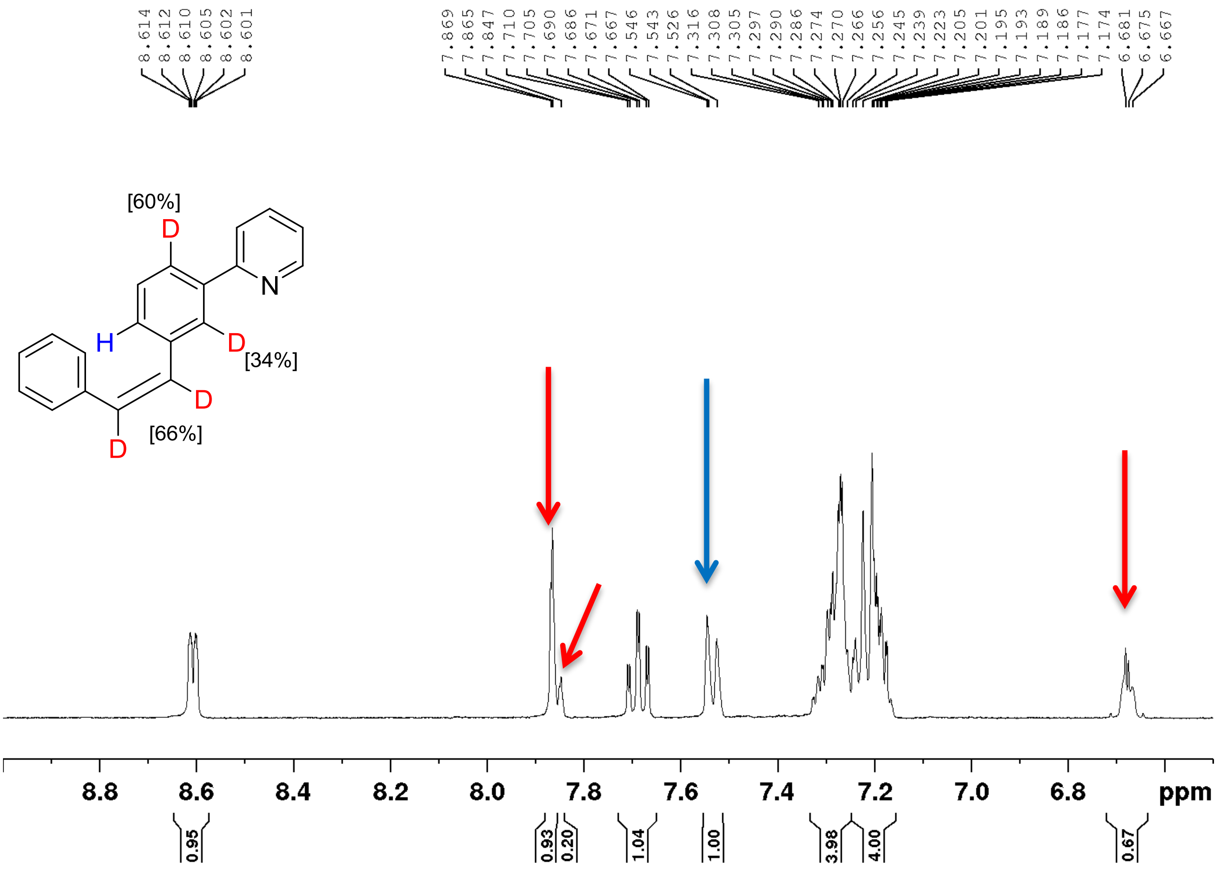

^{1}H-NMR (400 MHz, CD_2Cl_2) Spectra of deuterated compound following the CuI procedure for 62 h: Yield: 20%

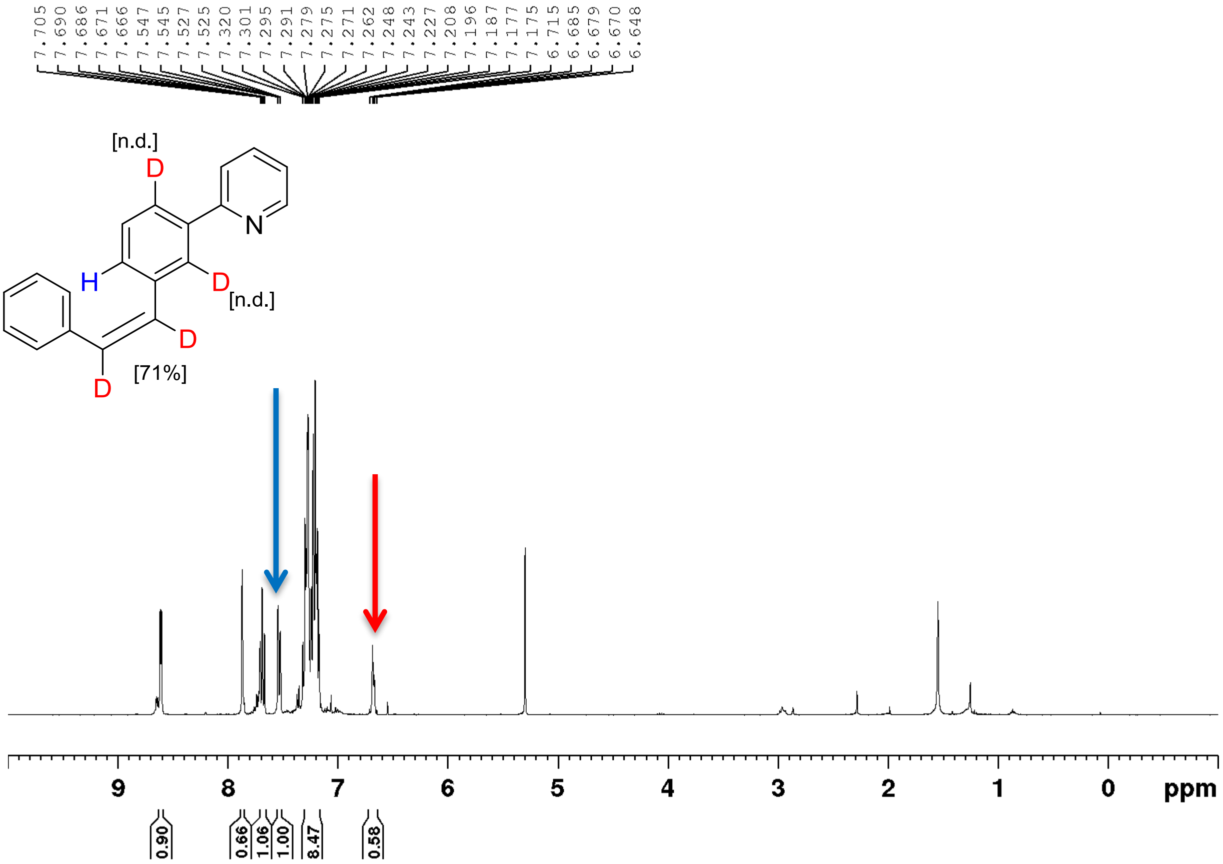

Enlargement of relevant area:

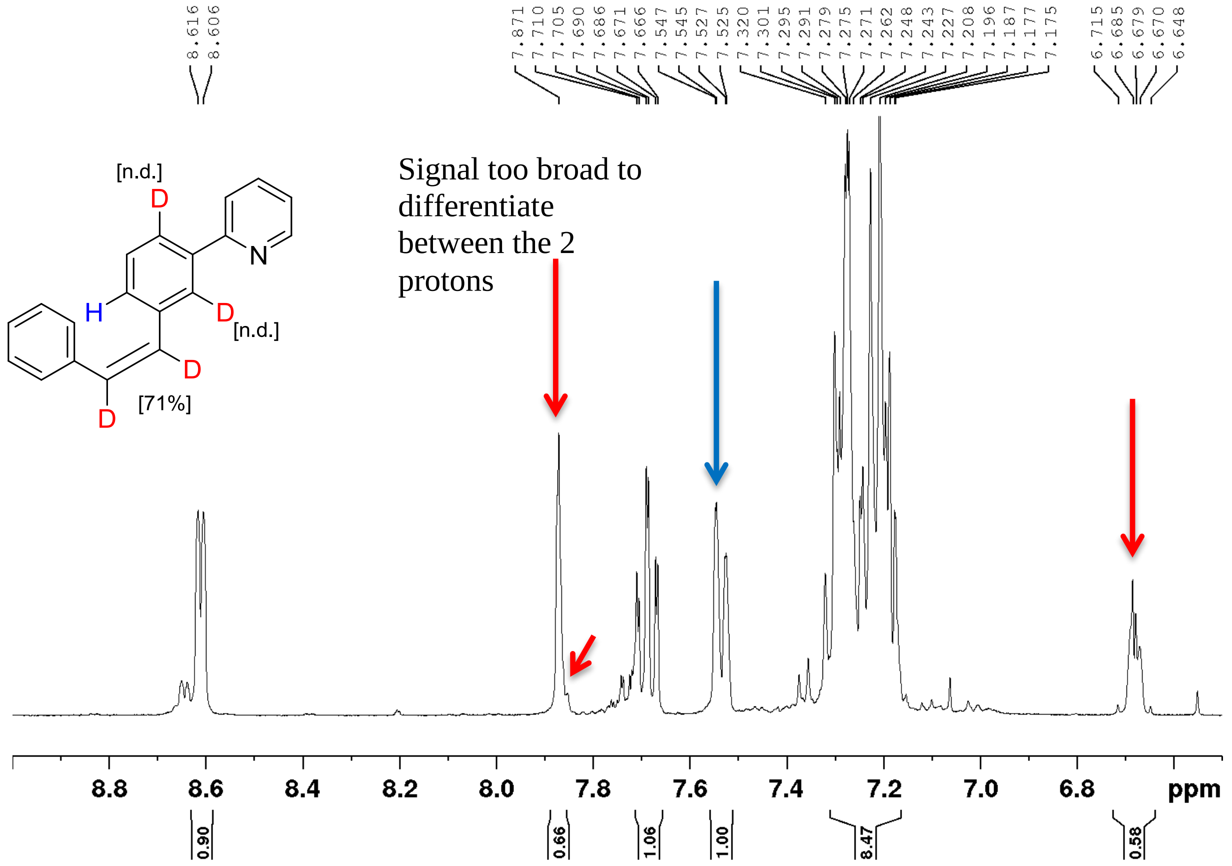

(*E*)-2-(3-styrylphenyl)pyridine 42 (III)

^{1}H-NMR (400 MHz, CD_2Cl_2) Spectra of pure compound **42**:

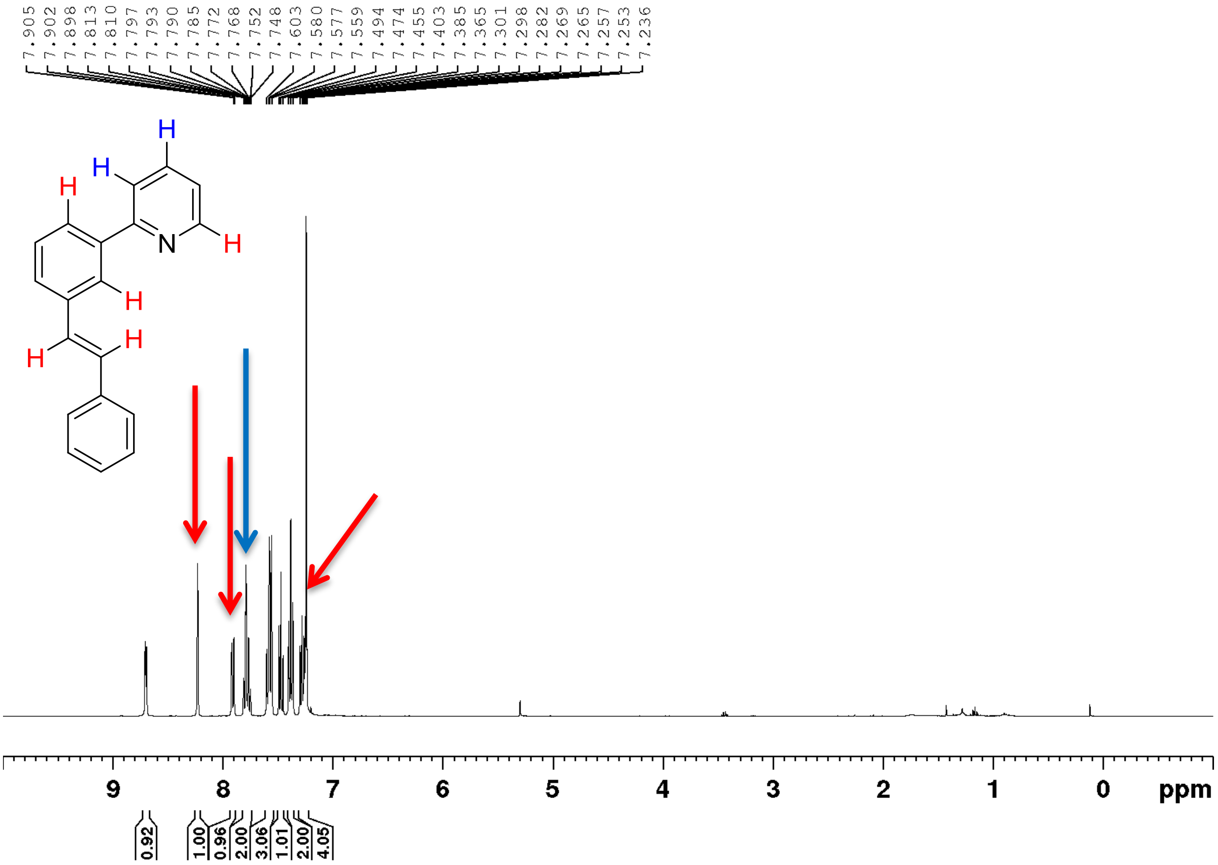

Enlargement of relevant area:

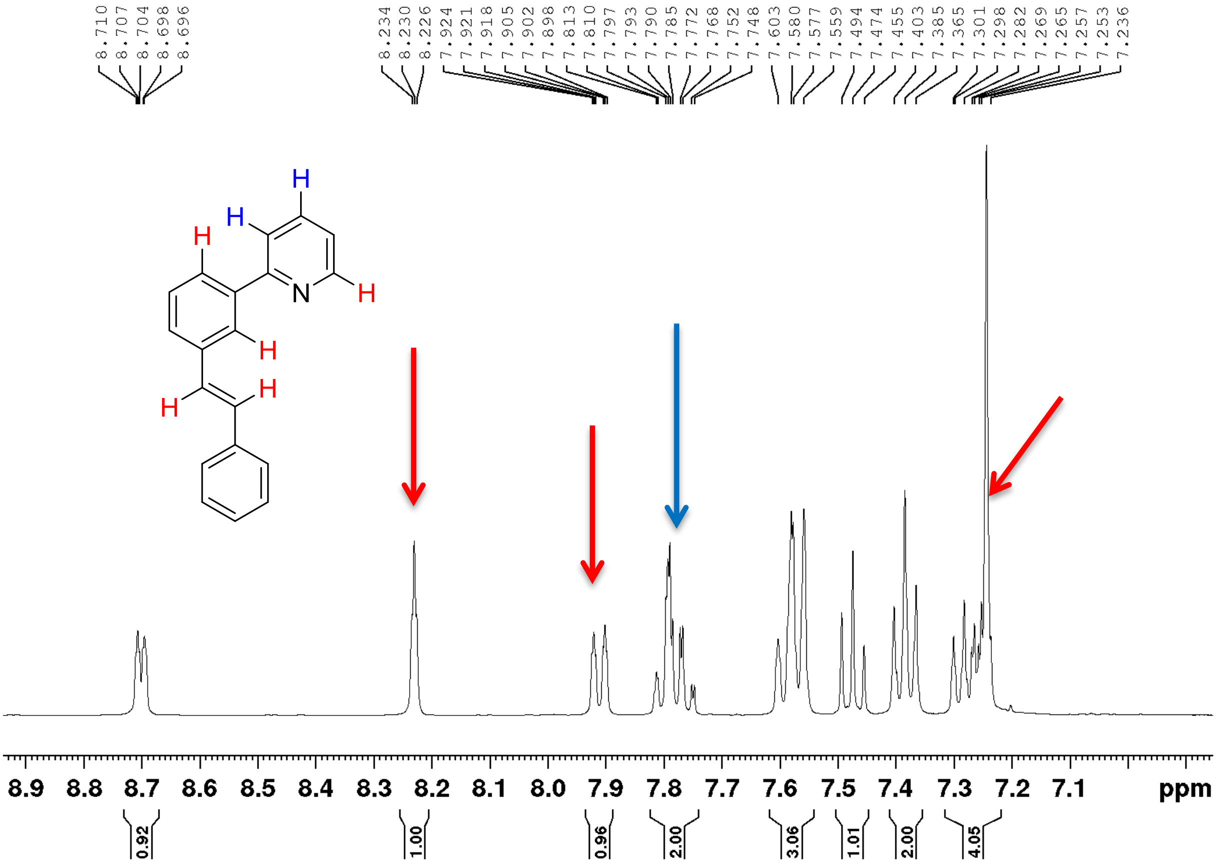

^{1}H-NMR (400 MHz, CD_2Cl_2, mesitylene) Spectra of deuterated compound **42** following the CuI procedure for 16 h: Yield: 20% (*Z*/*E*: 2.5:1)

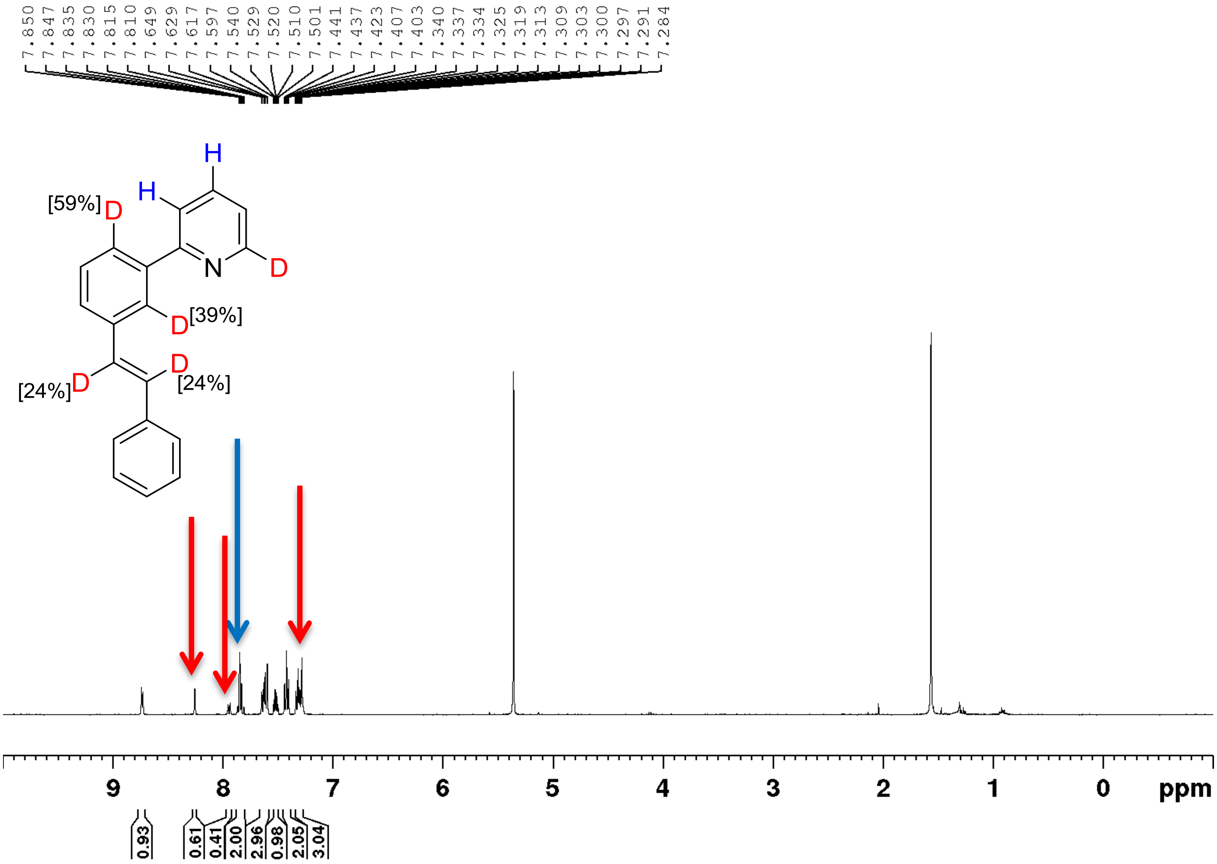

Enlargement of relevant area:

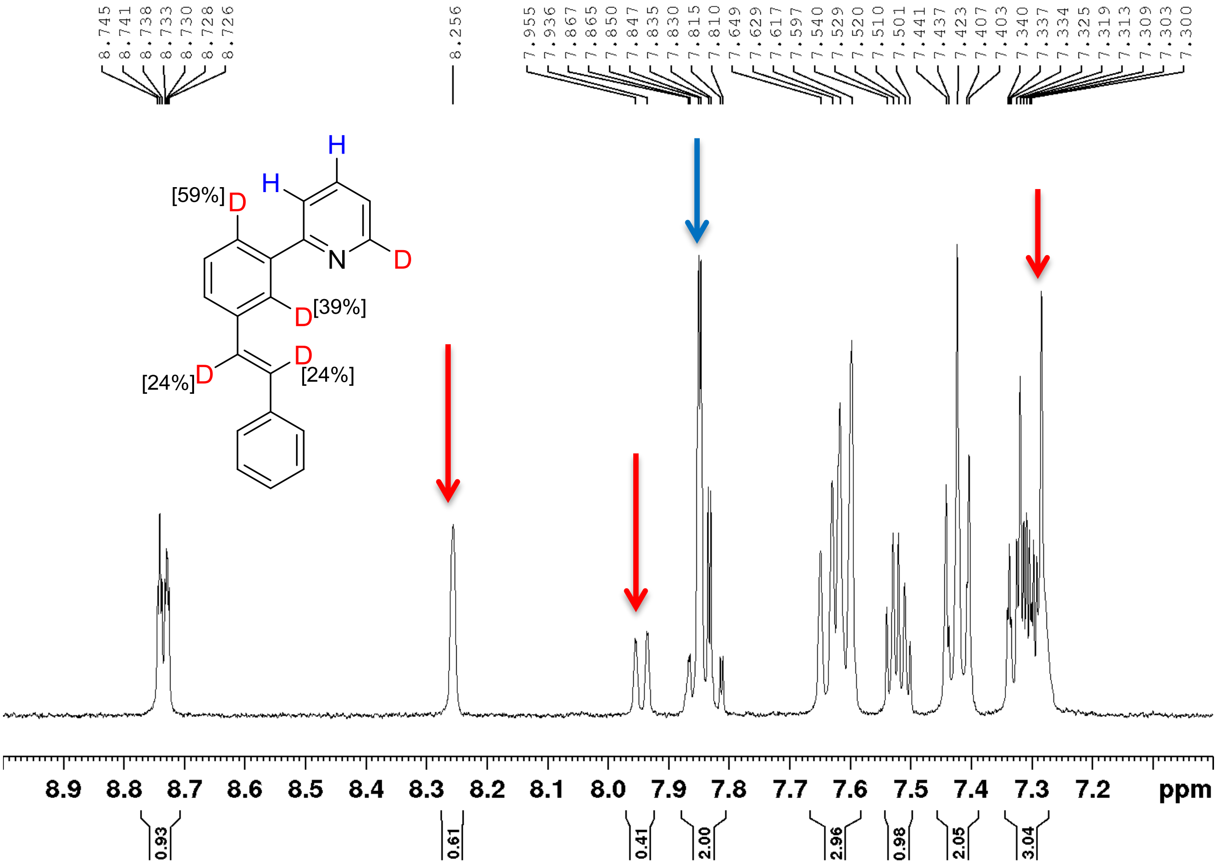

^{1}H-NMR (400 MHz, CD_2Cl_2, mesitylene) Spectra of deuterated compound **42** following the CuI procedure for 62 h: Yield: 50%

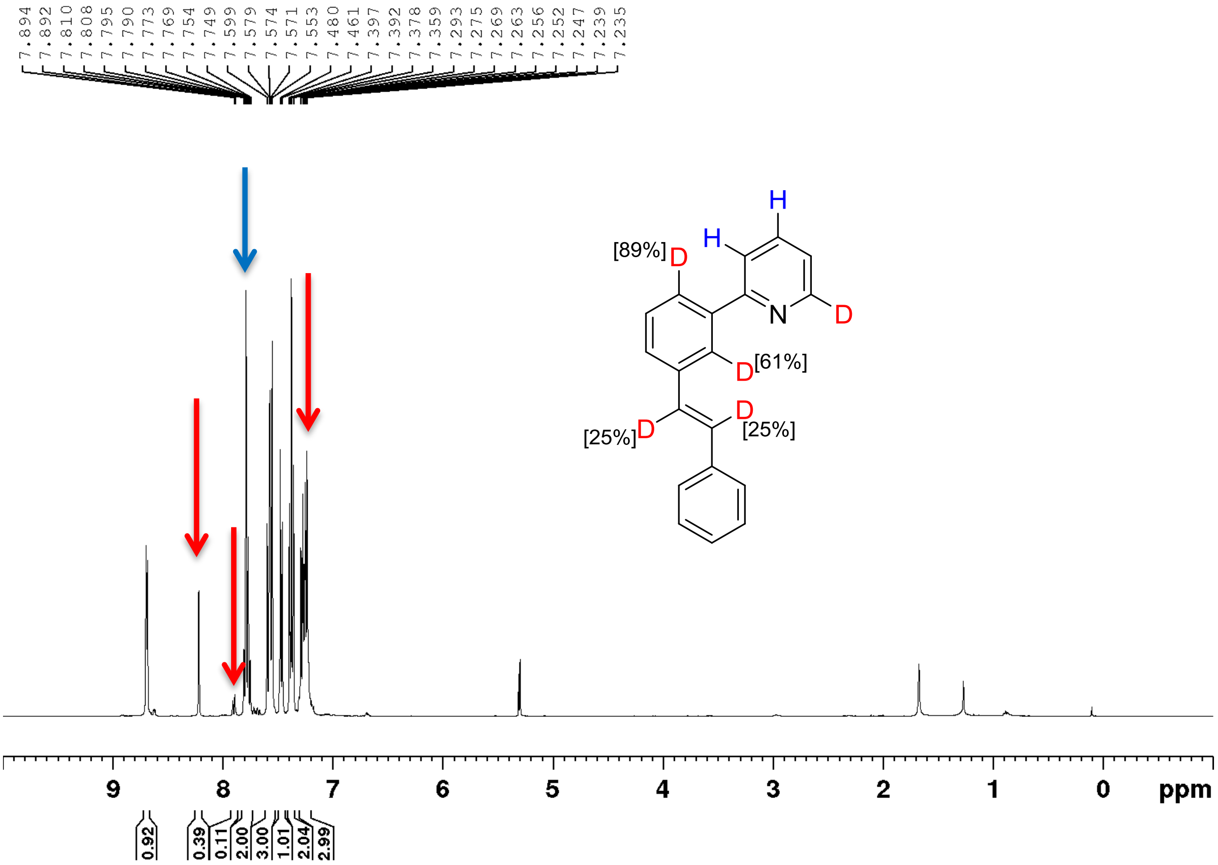

Enlargement of relevant area:

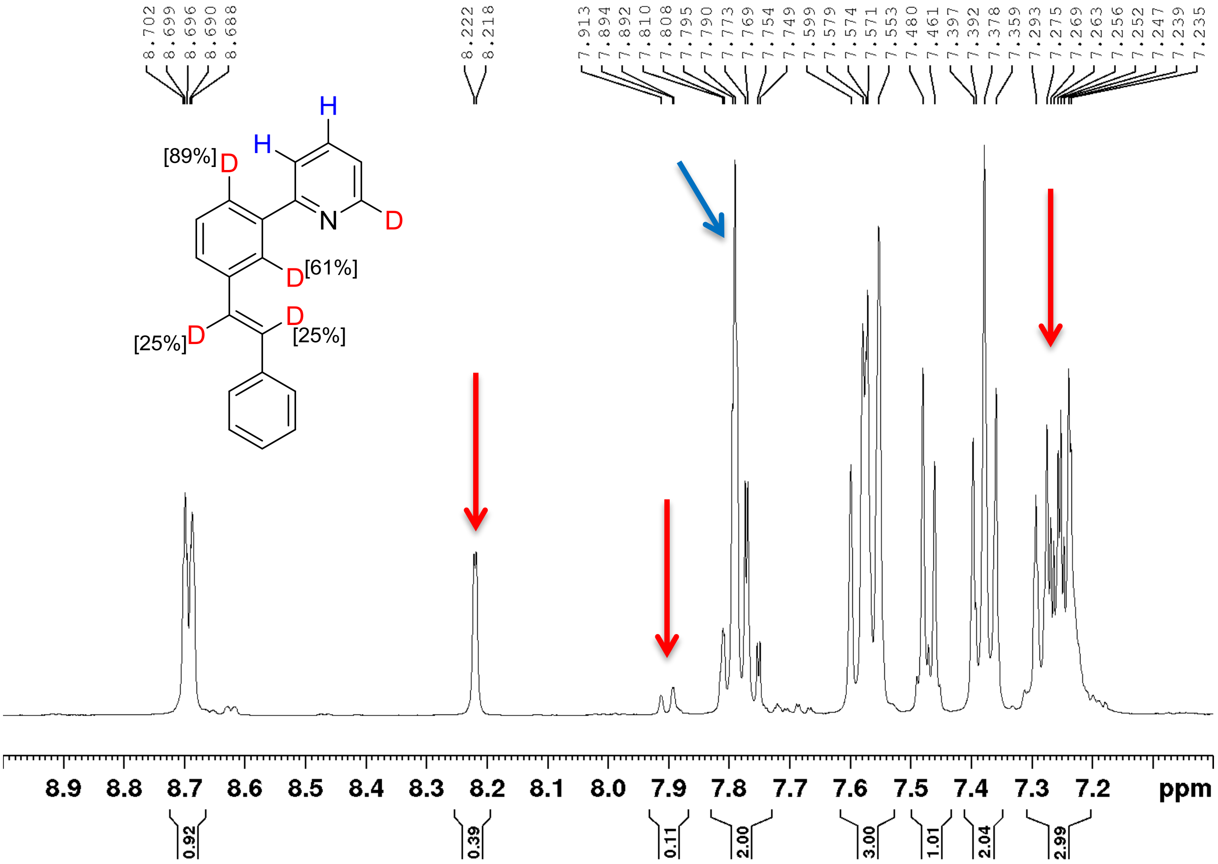

3.5.21 4-(phenylethinyl)acetophenone 38

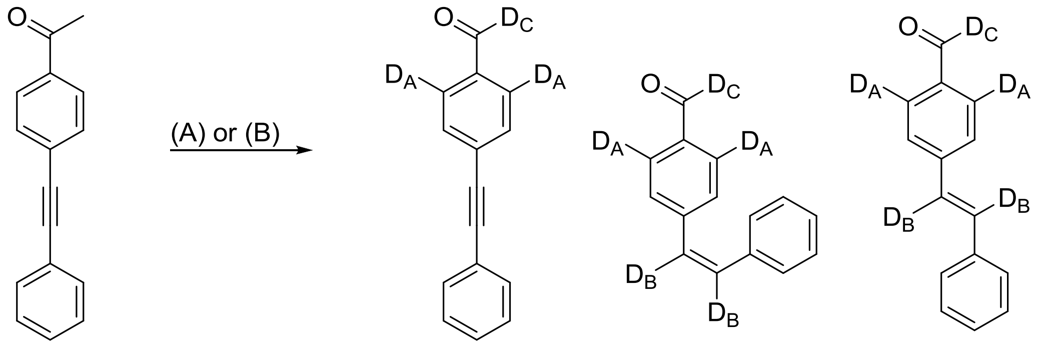

I II III

		I			II (*Z*)				III (*E*)			
	t	D_A	D_C	Yield	D_A	D_B	D_C	Yield	D_A	D_B	D_C	Yield
cond. (A)	16 h	n.o.	n.o.	n.o.	n.o.	82%	n.o.	42%	n.o.	63%	n.o.	13%
	62 h	n.o.	n.o.	n.o.	n.o.	n.d.	n.o.	n.o.	88%	84%	<10%	52%
cond. (C)	16 h	n.o.	87%	>99%	n.o.	n.o.	n.o.	n.o.	n.o.	n.o.	n.o.	n.o.
	62 h	n.o.	87%	>99%	n.o.	n.o.	n.o.	n.o.	n.o.	n.o.	n.o.	n.o.

4-(phenylethinyl)acetophenone 38 (I)

^{1}H-NMR (300 MHz, $CDCl_3$) Spectra of pure compound **38**:

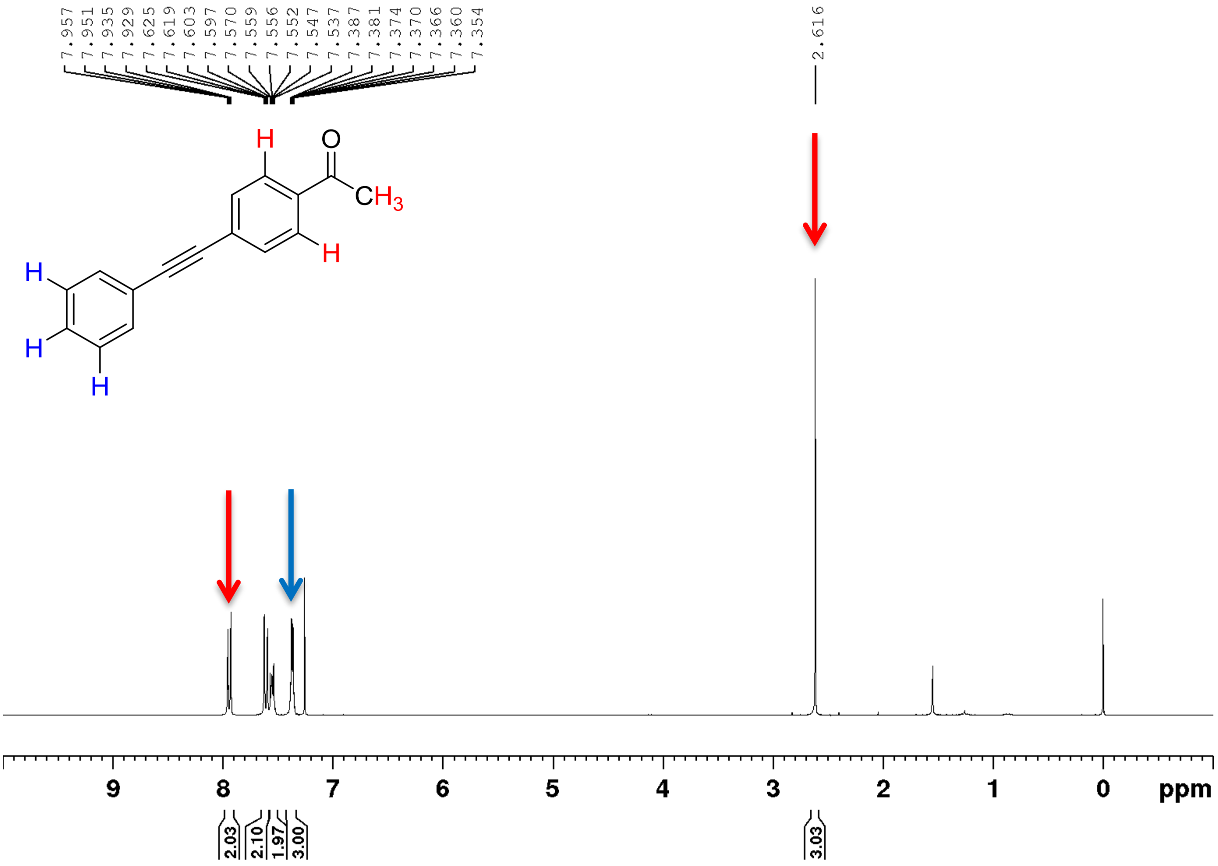

Enlargement of relevant area:

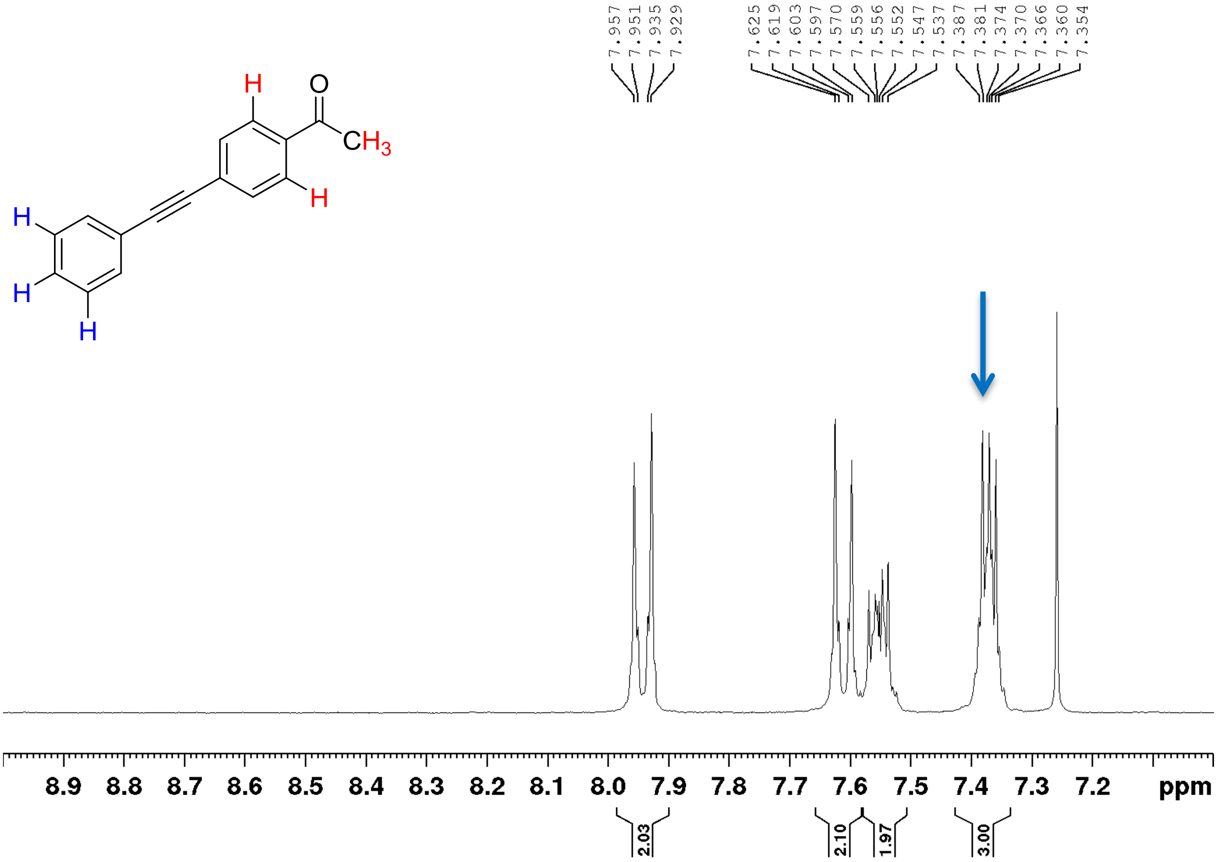

^{1}H-NMR (400 MHz, $CDCl_3$, nitromethan) Spectra of deuterated compound **38** following the KOD procedure for 16 h: Yield: >99%

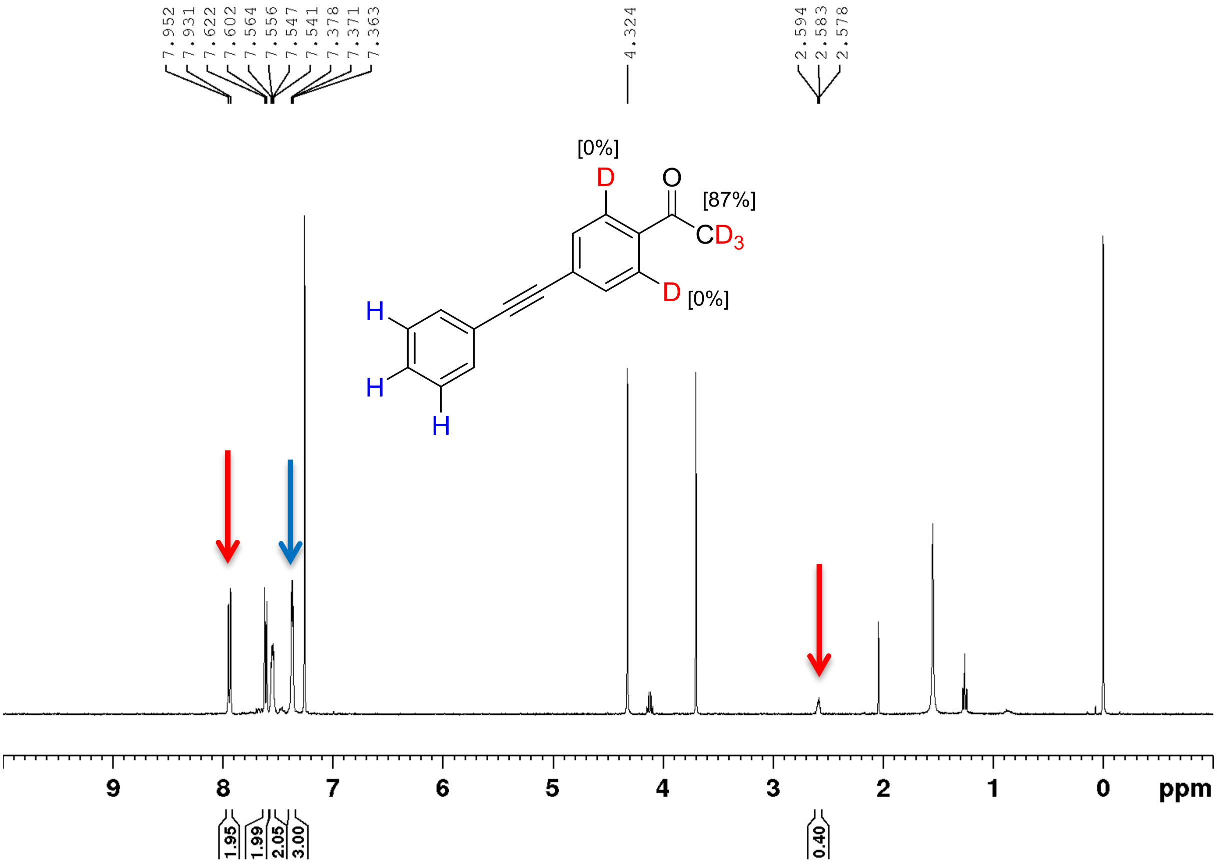

^{1}H-NMR (400 MHz, $CDCl_3$, mesitylene) Spectra of deuterated compound **38** following the KOD procedure for 62 h: Yield: >99%

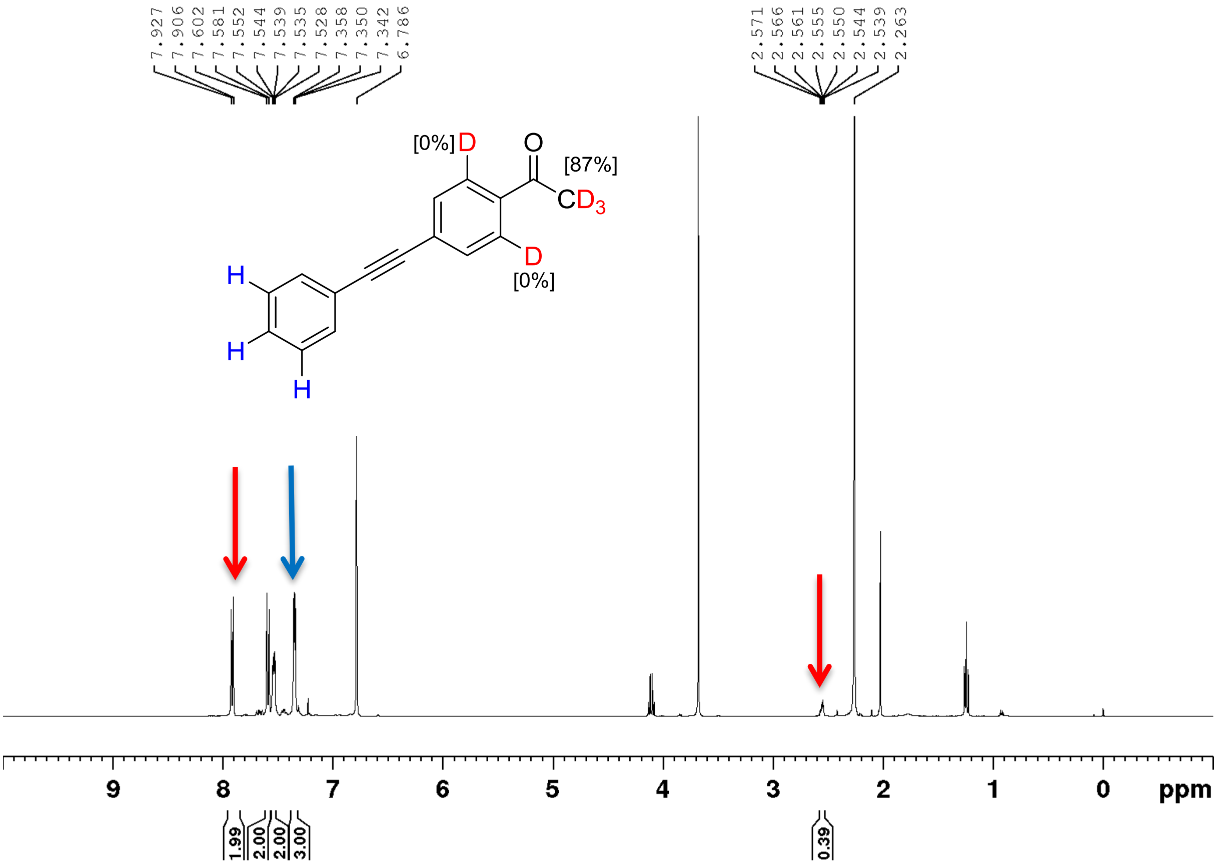

^{1}H-NMR (400 MHz, $CDCl_3$) Spectra of deuterated compound following the CuI procedure for 16 h: (*Z*/*E*: 3:1)

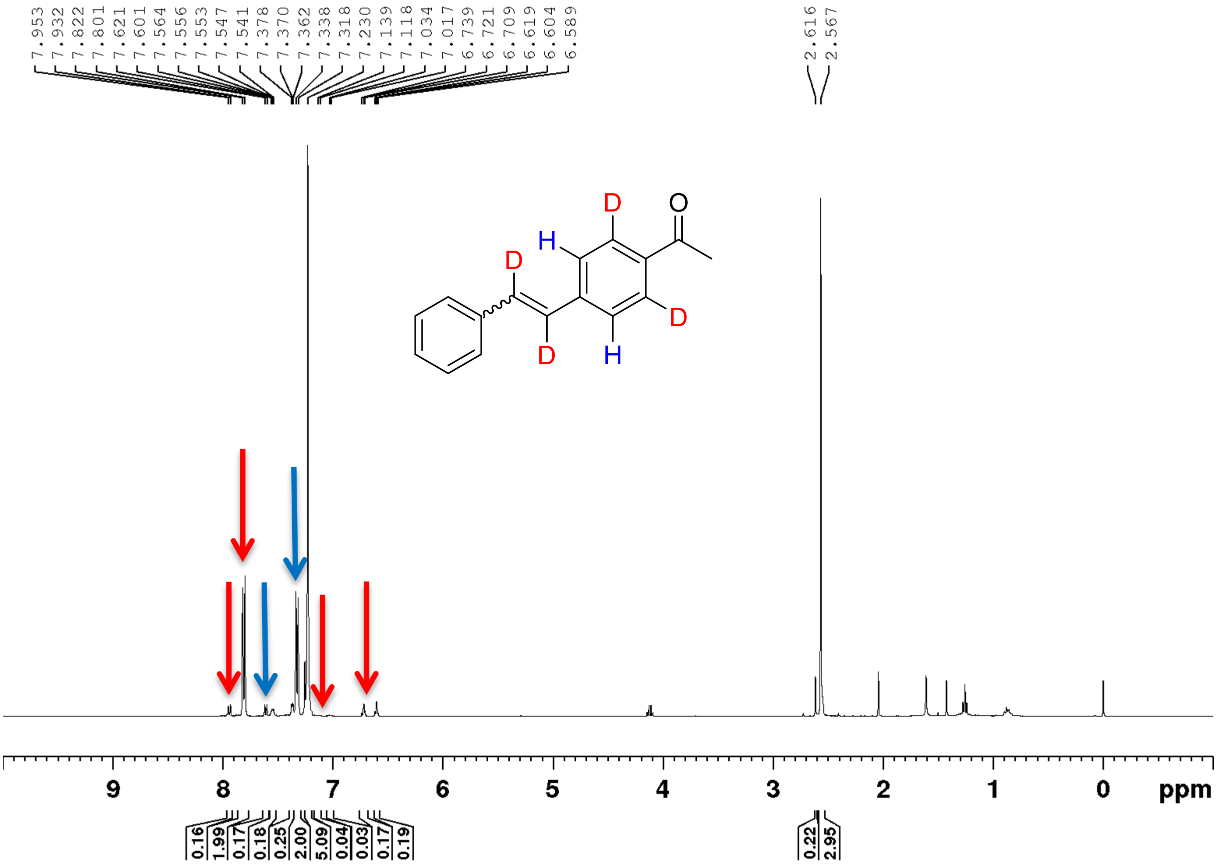

Enlargement of relevant area:

Yield of *Z*-Isomer (**II**)[1]: 42%

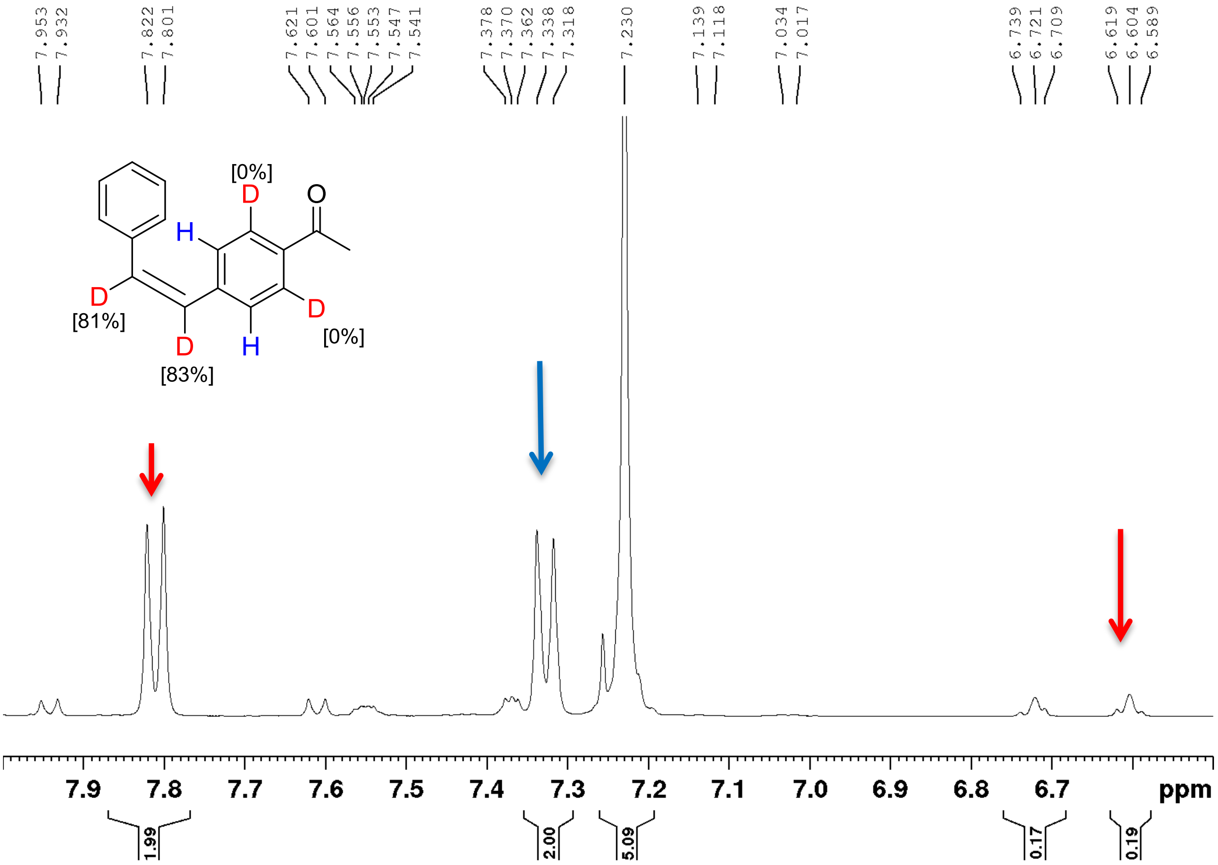

Yield of *E*-Isomer **43** (**III**): 13%

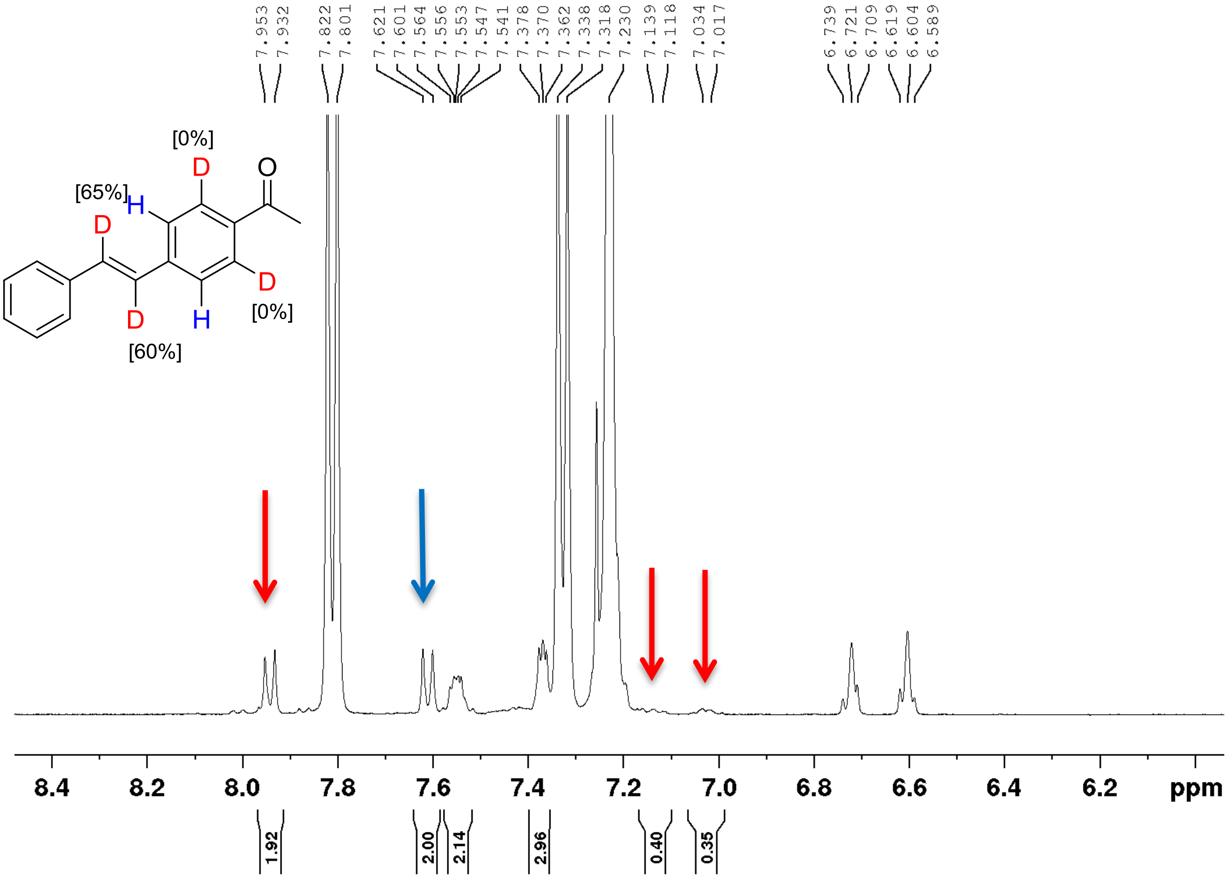

^{1}H-NMR (400 MHz, $CDCl_3$) Spectra of deuterated compound **43** following the CuI procedure for 62 h: Yield: 52%

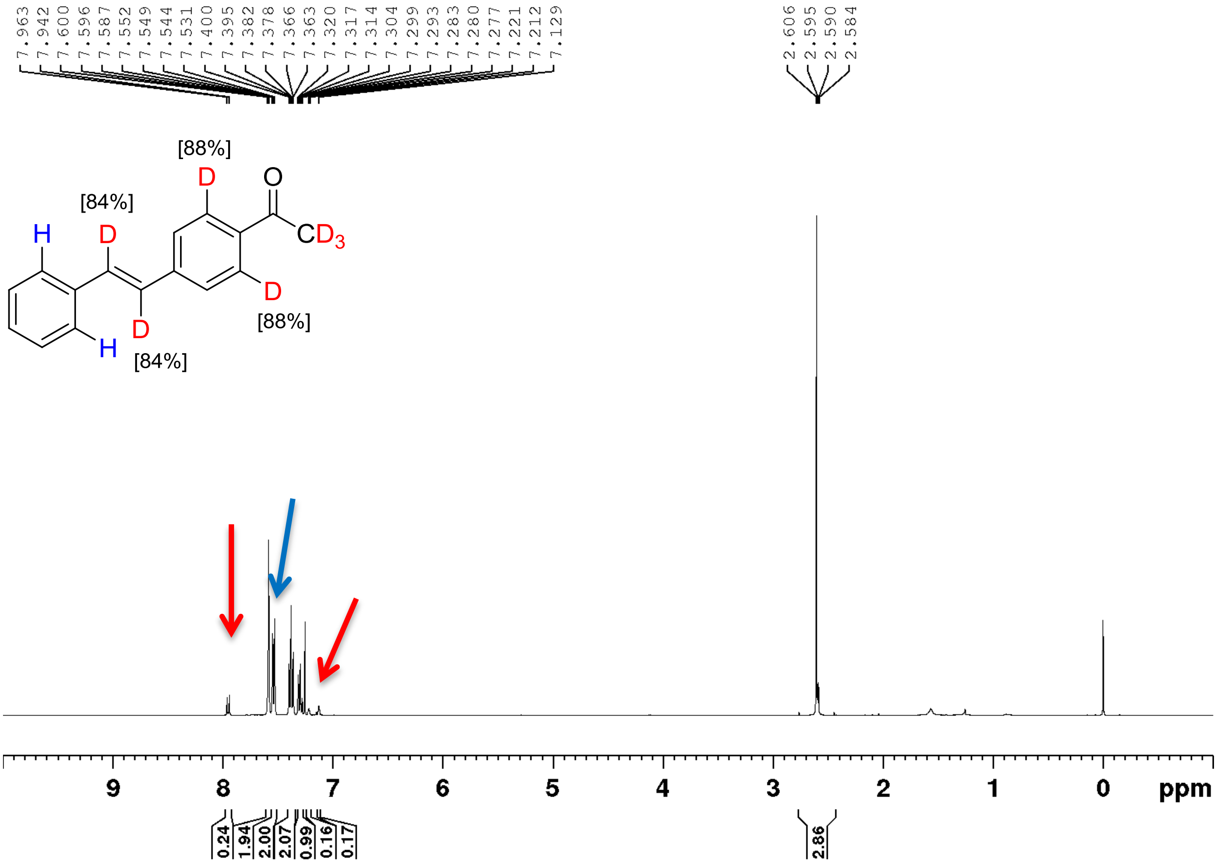

Enlargement of relevant area:

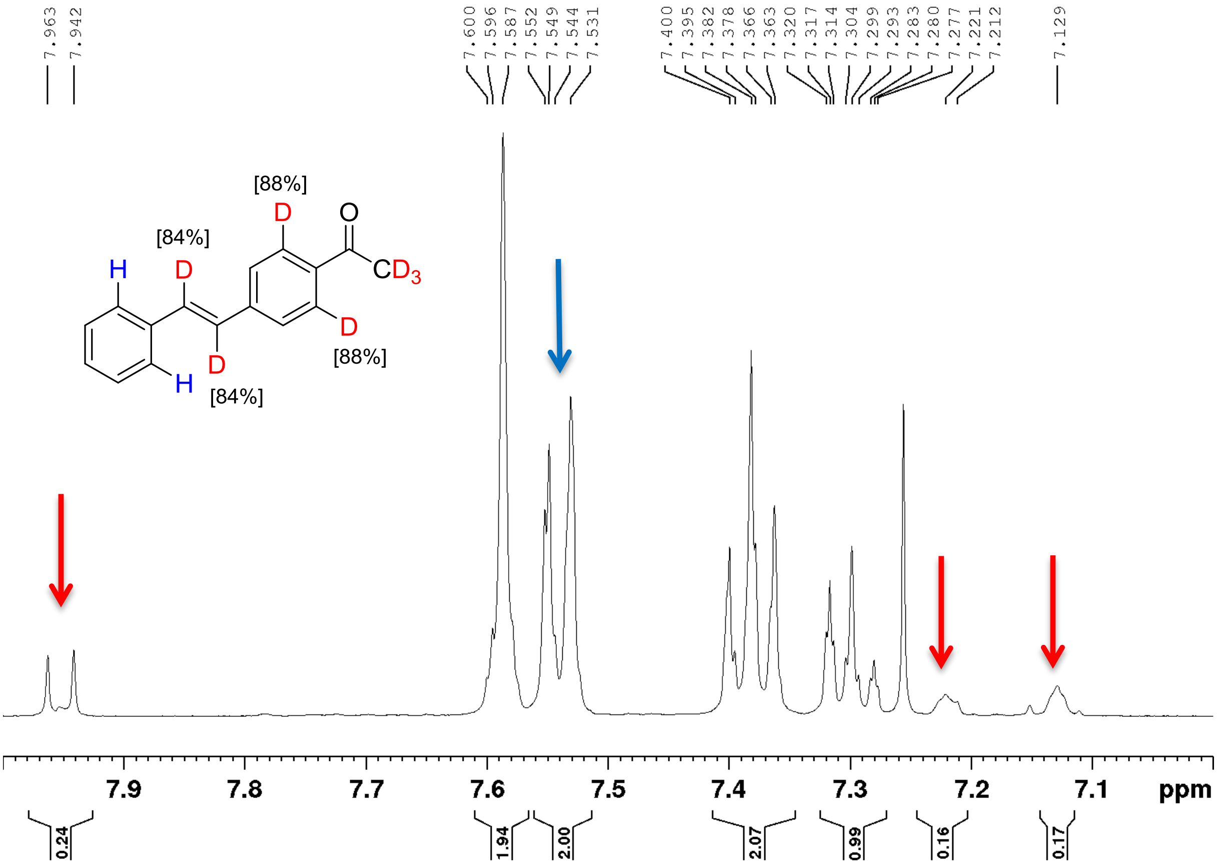
7.963
7.942
7.600
7.596
7.587
7.552
7.549
7.544
7.531
7.400
7.395
7.382
7.378
7.366
7.363
7.320
7.317
7.314
7.304
7.299
7.293
7.283
7.280
7.277
7.221
7.212
7.129
[88%]
[84%]
[88%]
[84%]
7.9
7.8
7.7
7.6
7.5
7.4
7.3
7.2
7.1
ppm
0.24
1.94
2.00
2.07
0.99
0.16
0.17

3.5.22 1-(3-(2-phenylethynyl)phenyl)ethanone 39

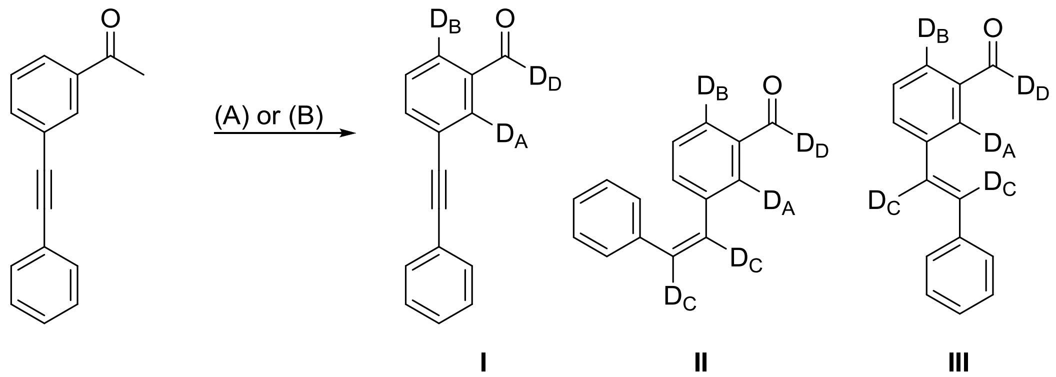

		I				II (*Z*)					III (*E*)				
	t	D_A	D_B	D_D	Yield	D_A	D_B	D_C	D_D	Yield	D_A	D_B	D_C	D_D	Yield
cond. (A)	16 h	n.o.	n.o.	n.o.	n.o.	n.o.	n.o.	83%	<10%	63%	n.o.	n.o.	n.o.	n.o.	n.o.
	62 h	n.o.	n.o.	n.o.	n.o.	n.o.	n.o.	n.o.	n.o.	n.o.	<10%	86%	79%	10%	91%
cond. (C)	16 h	n.o.	n.o.	84%	>99%	n.o.	n.o.	n.o.	n.o.	n.o.	n.o.	n.o.	n.o.	n.o.	n.o.
	62 h	n.o.	n.o.	87%	>99%	n.o.	n.o.	n.o.	n.o.	n.o.	n.o.	n.o.	n.o.	n.o.	n.o.

1-(3-(2-phenylethynyl)phenyl)ethanone 39 (I)

^{1}H-NMR (300 MHz, $CDCl_3$) Spectra of pure compound **39**:

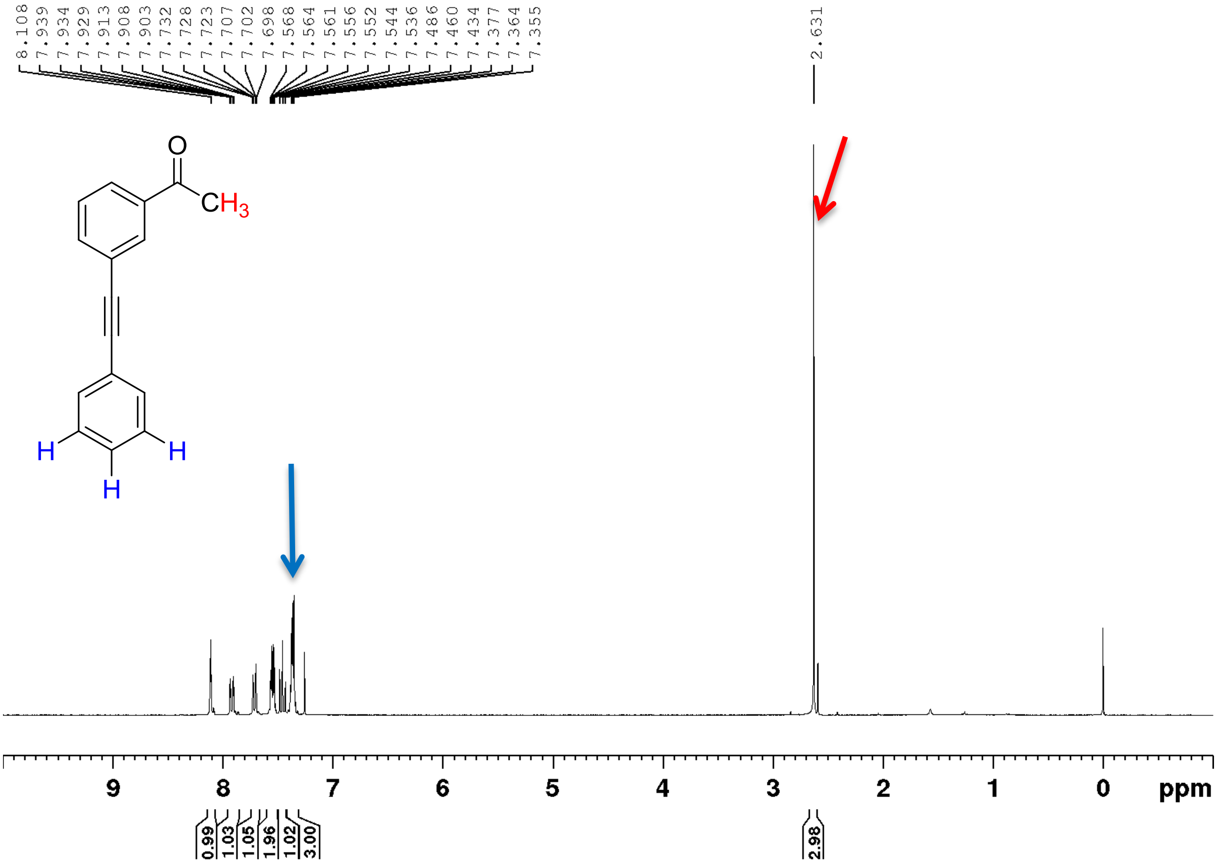

Enlargement of relevant area:

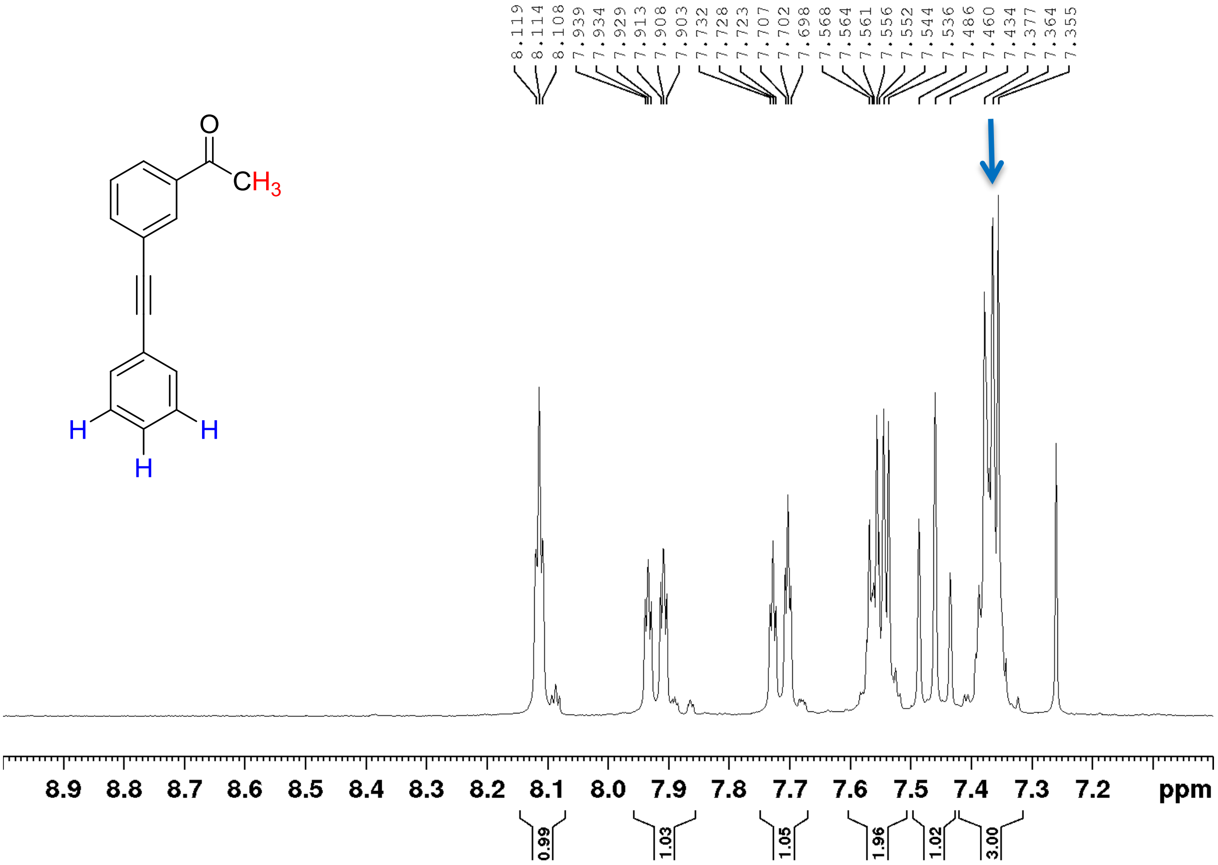

^{1}H-NMR (400 MHz, $CDCl_3$) Spectra of deuterated compound **39** following the KOD procedure for 16 h: Yield: >99%

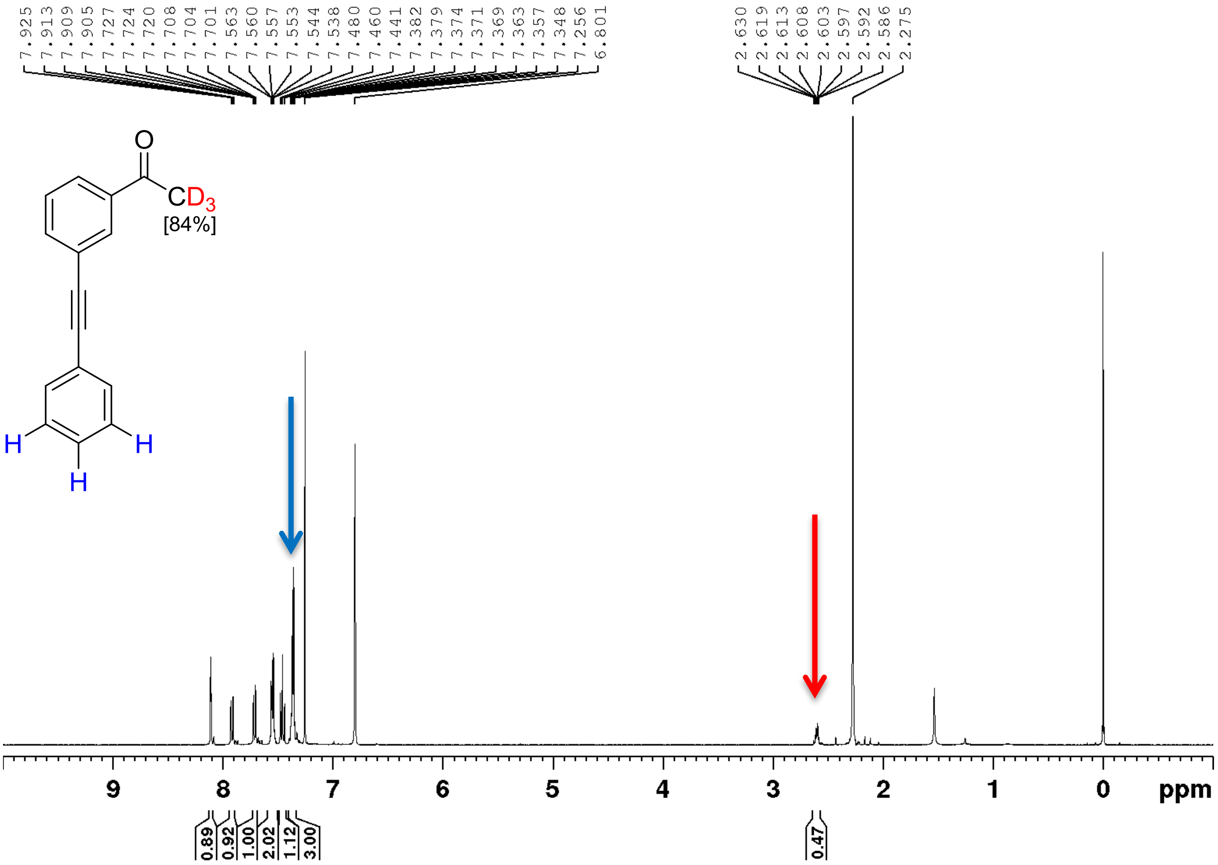

^{1}H-NMR (400 MHz, $CDCl_3$) Spectra of deuterated compound **39** following the KOD procedure for 62 h: Yield: >99%

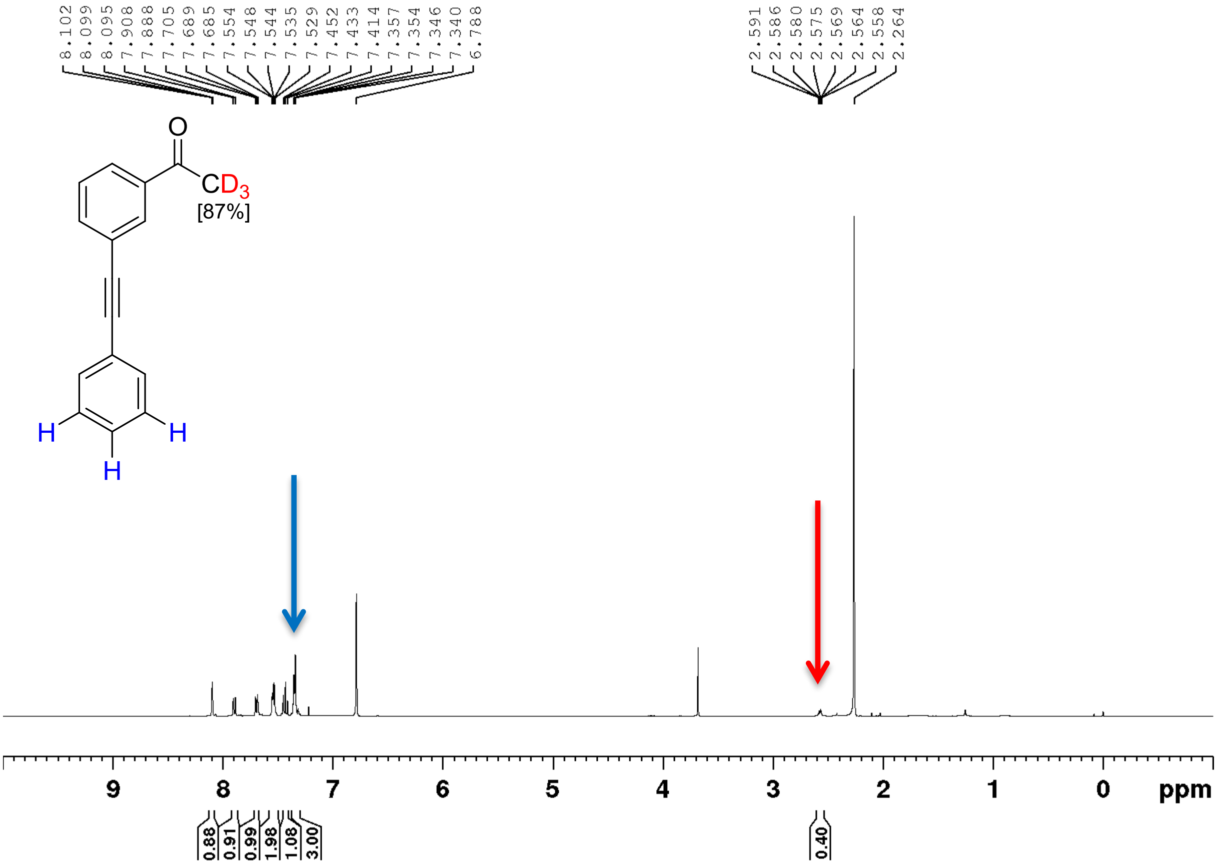

(*Z*)-1-(3-styrylphenyl)ethan-1-one (I)

^{1}H-NMR (400 MHz, CD_2Cl_2) Spectra of pure compound:

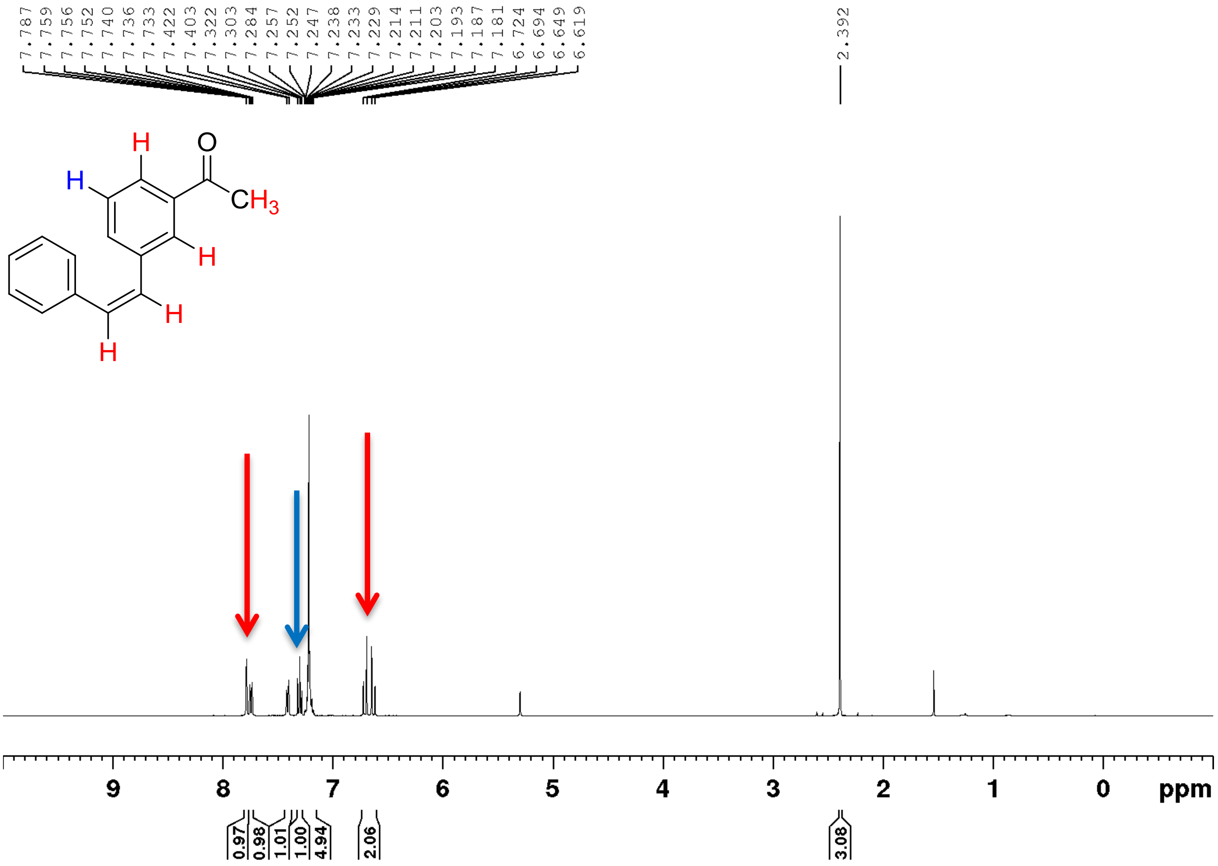

Enlargement of relevant area:

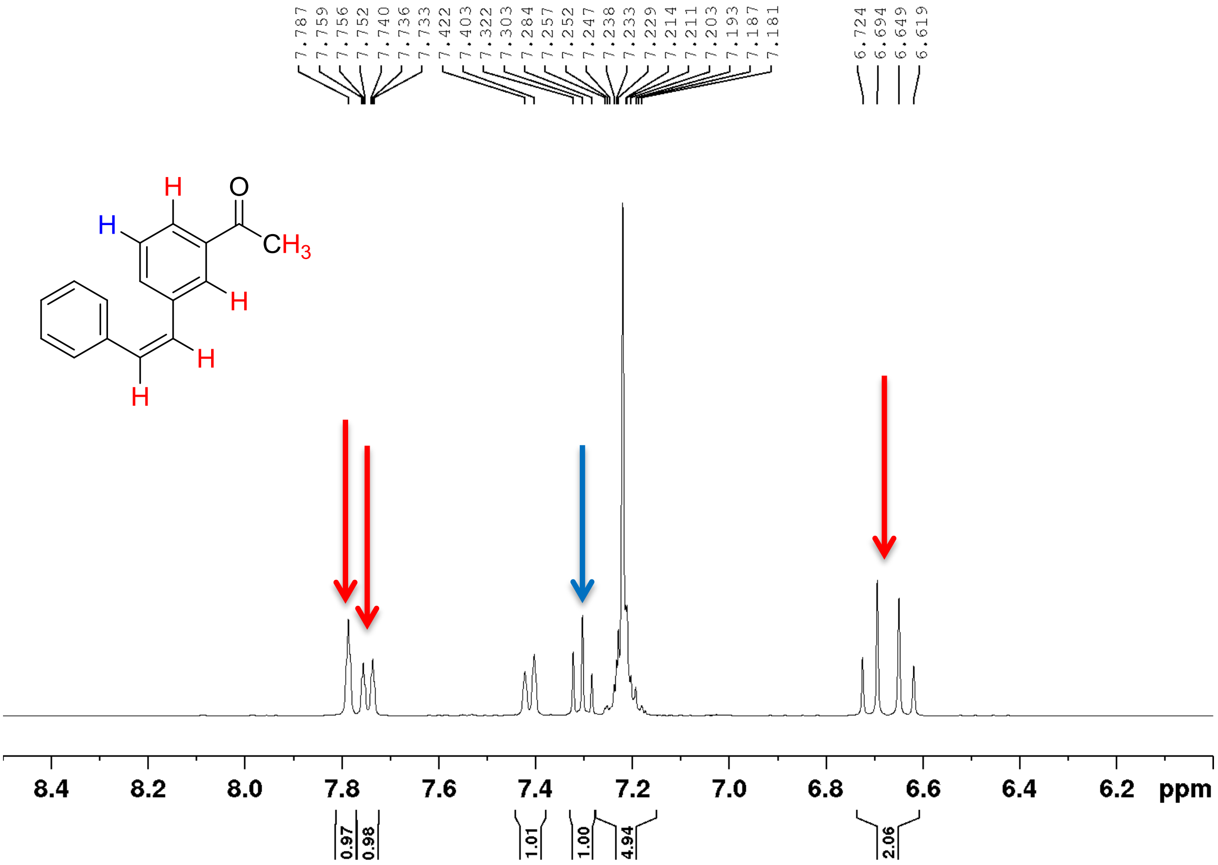

^{1}H-NMR (400 MHz, $CDCl_3$) Spectra of deuterated compound following the CuI procedure for 16 h: Yield: 63% (*Z*/*E*: 9:1)

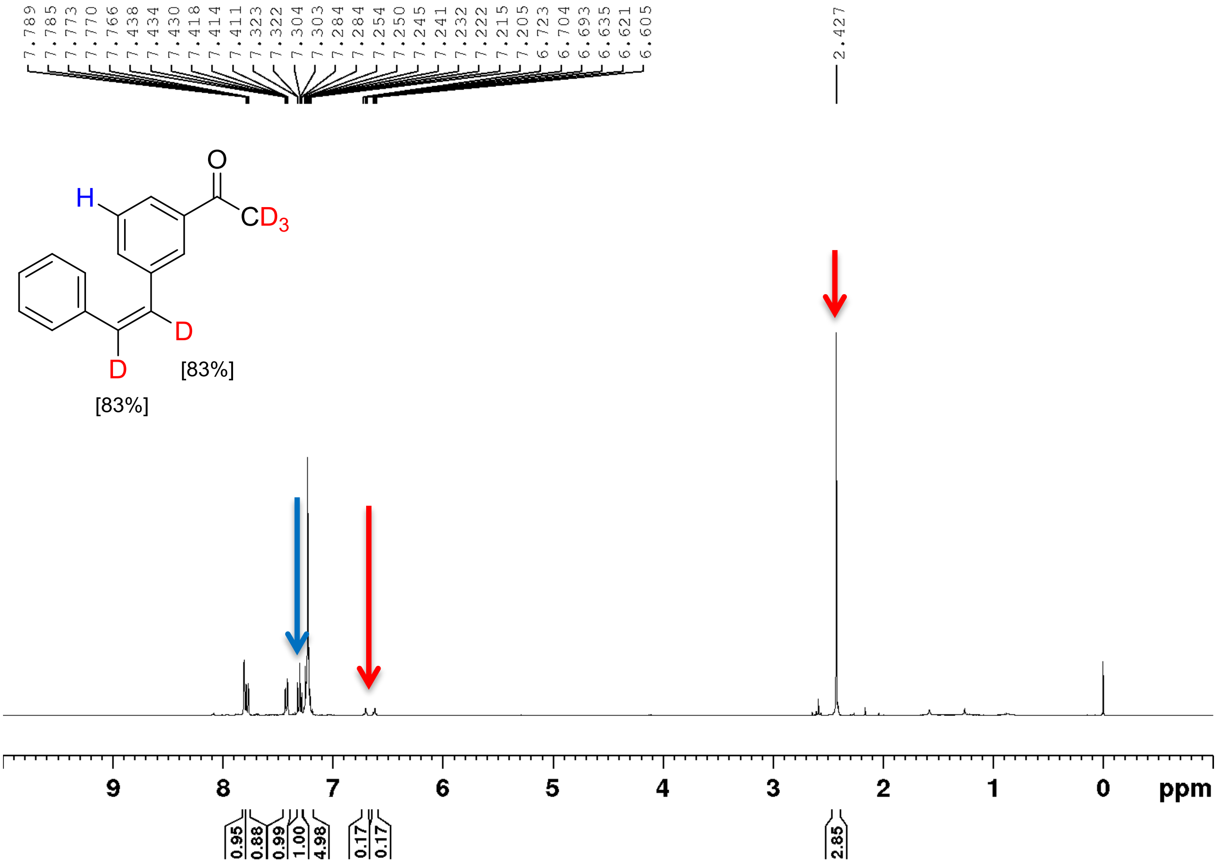

Enlargement of relevant area:

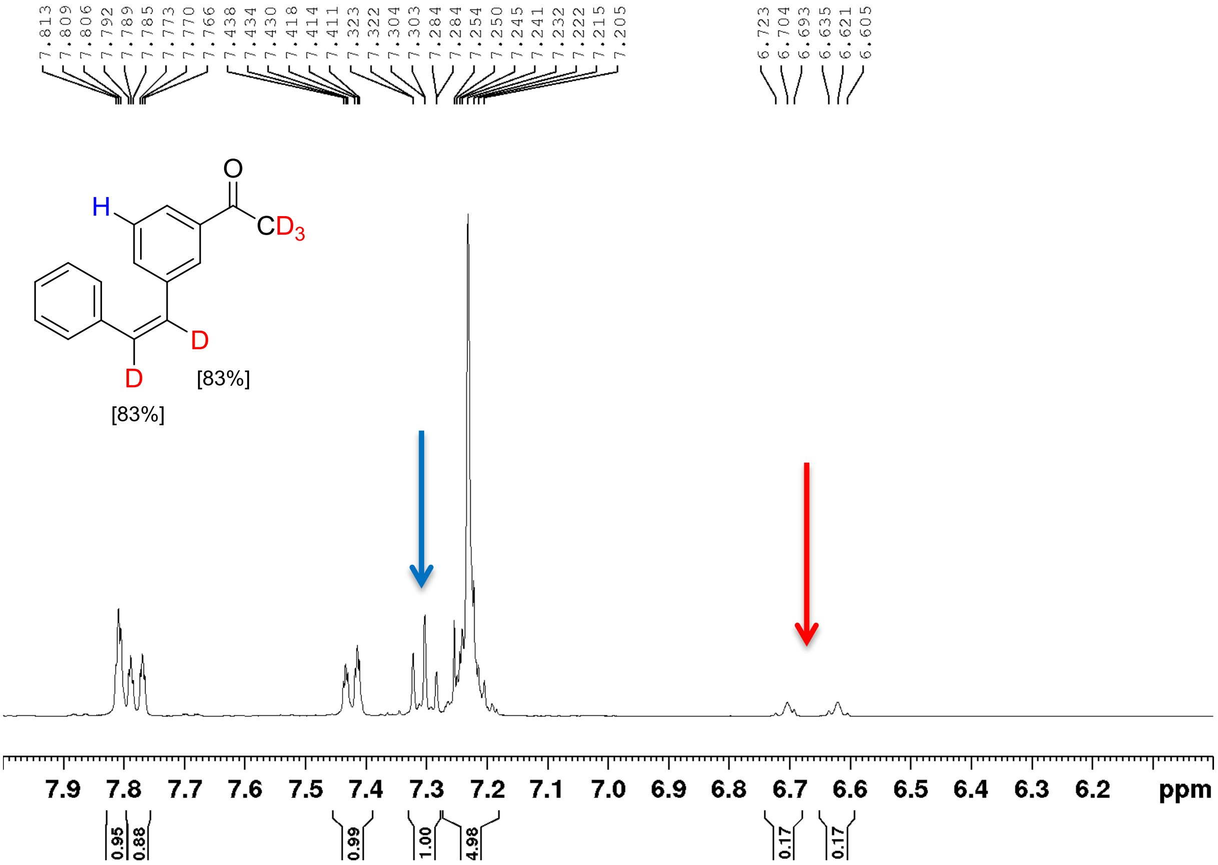

(*E*)-1-(3-styrylphenyl)ethan-1-one 44 (III)

^{1}H-NMR (400 MHz, CD_2Cl_2) Spectra of pure compound **44**:

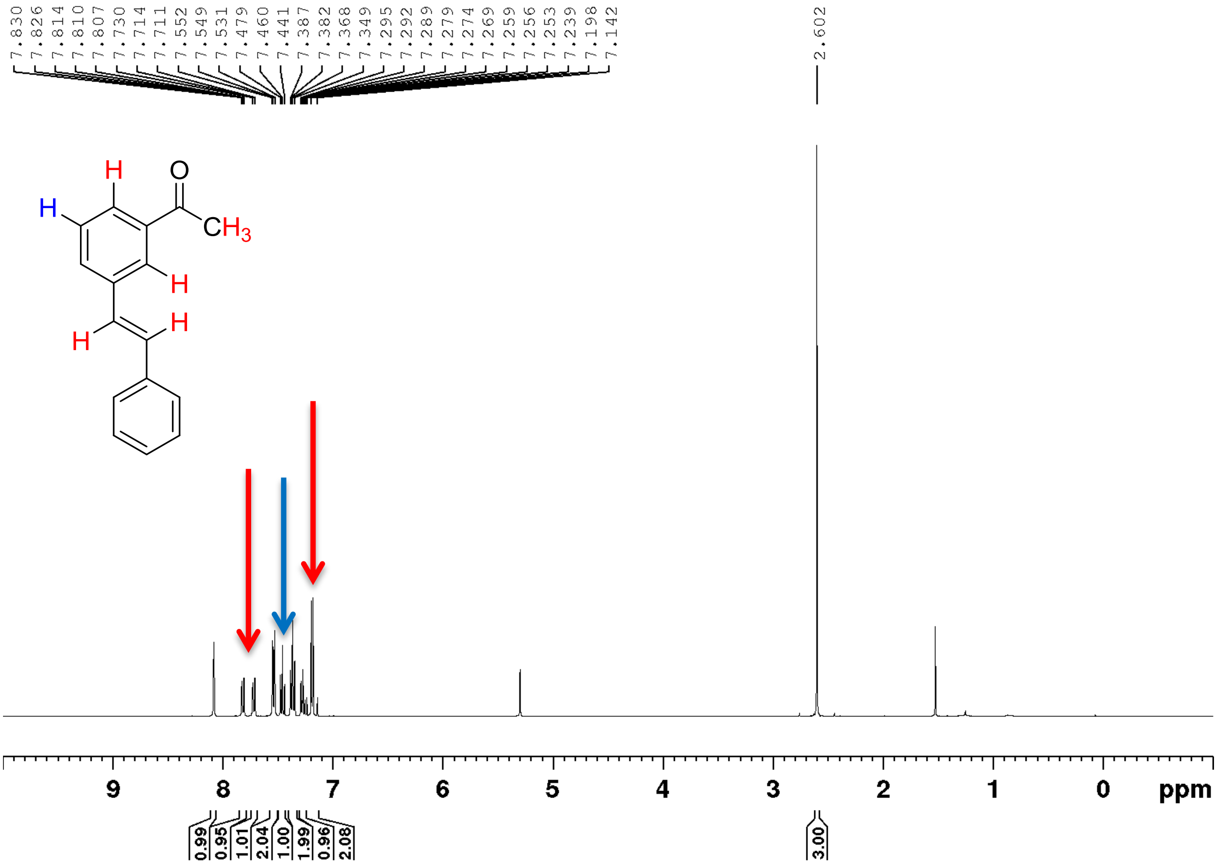

Enlargement of relevant area:

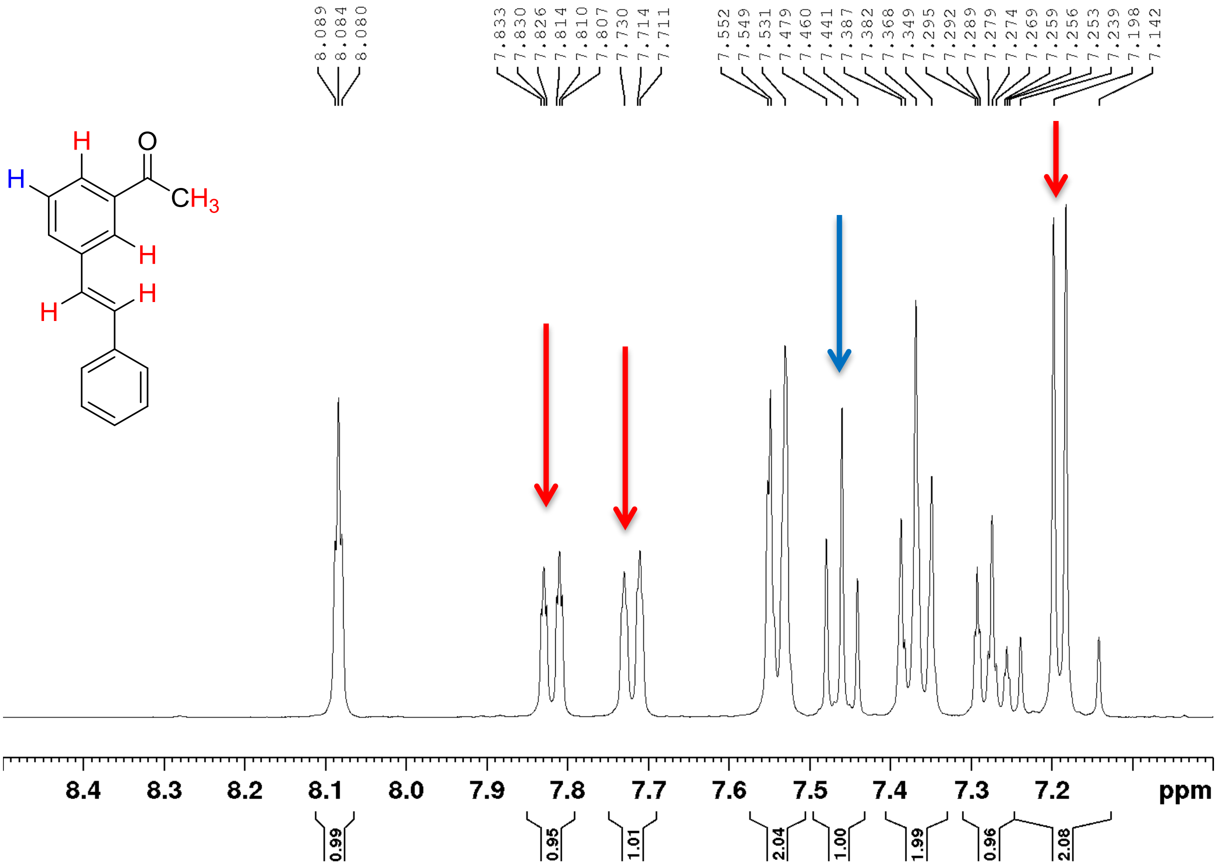

^{1}H-NMR (400 MHz, CD_2Cl_2) Spectra of deuterated compound **44** following the CuI procedure for 62 h: Yield: 91%

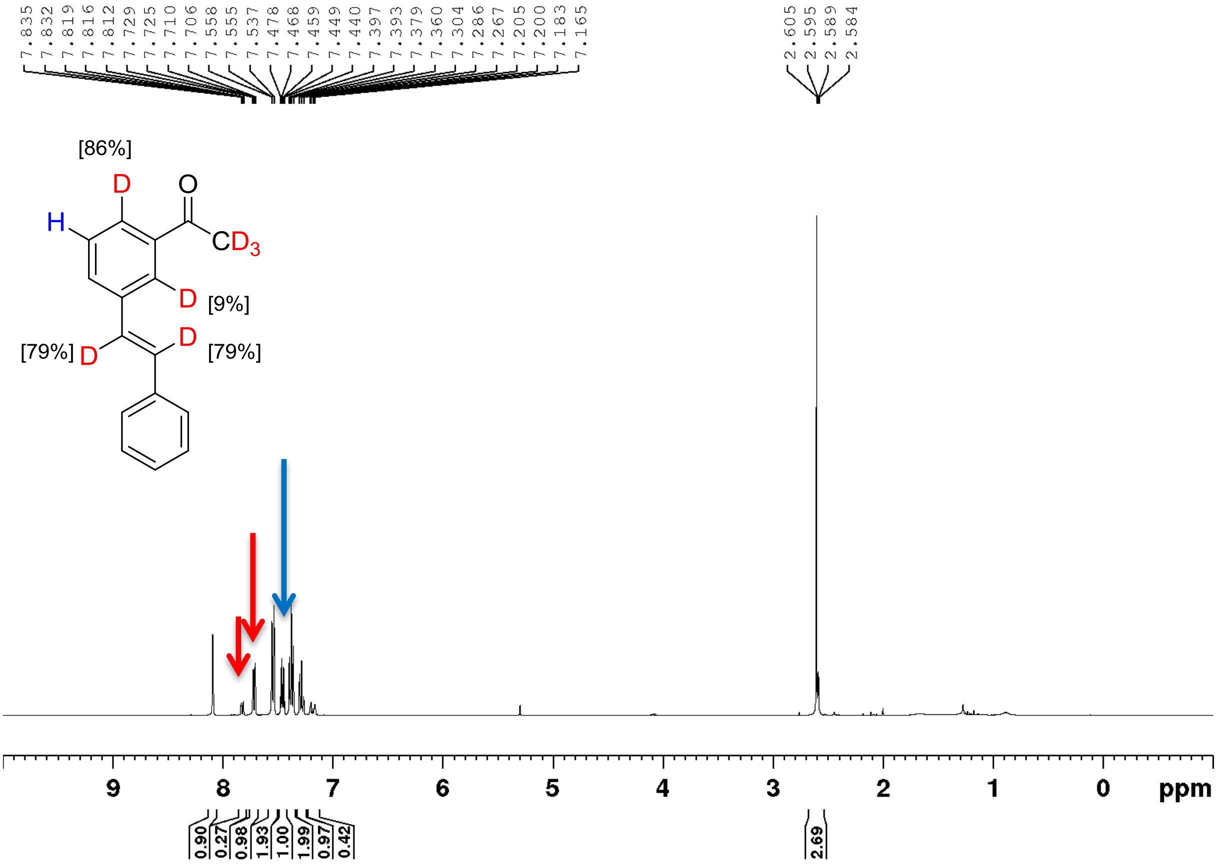

Enlargement of relevant area:

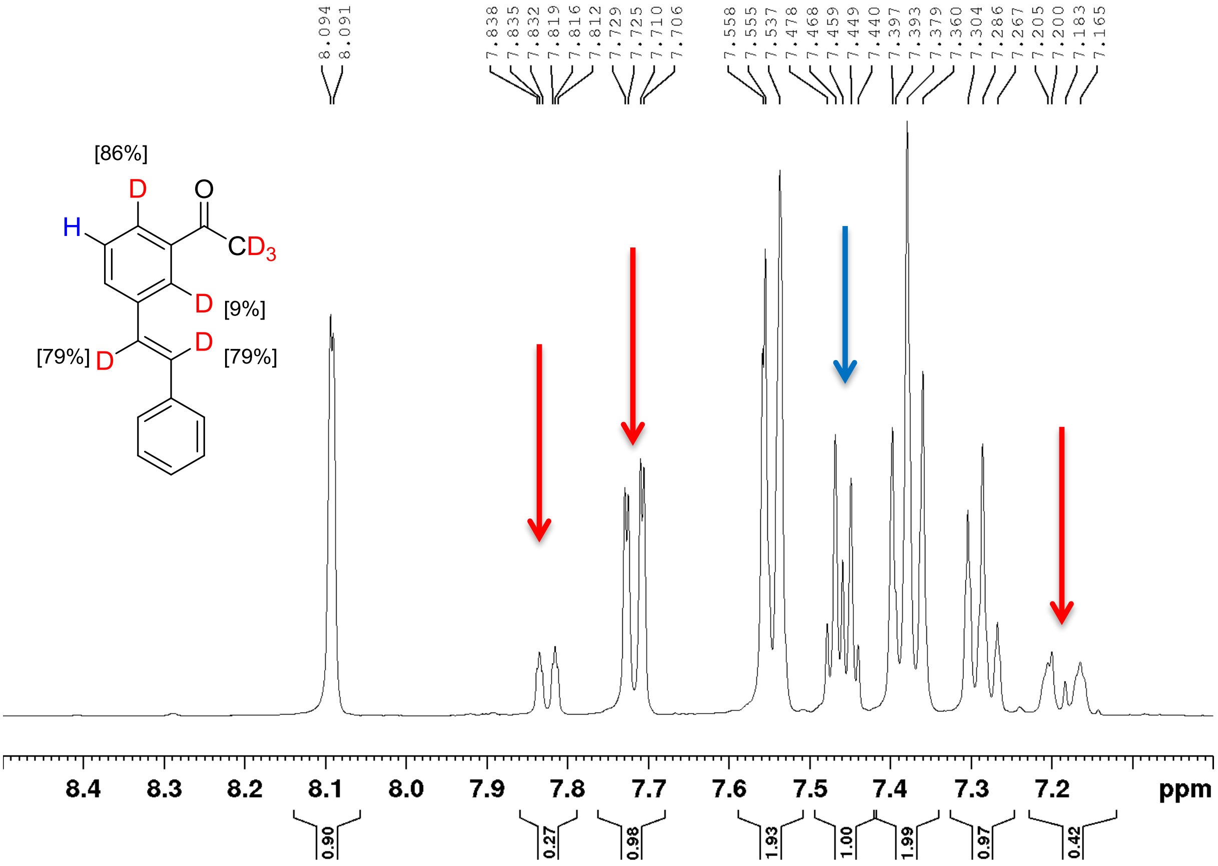

3.5.23 1-(3-(pyridin-2-yl)phenyl)ethan-1-one 40

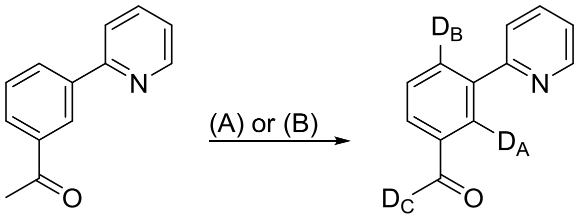

	t	D_A	D_B	D_D	Yield
cond. (A)	16 h	n.o.	80%	<10%	82%
	62 h	-	-	-	-
cond. (C)	16 h	76%	74%	80%	>99%
	62 h	-	-	-	-

1-(3-(pyridin-2-yl)phenyl)ethan-1-one 40

^{1}H-NMR (500 MHz, CD_2Cl_2) Spectra of pure compound **40**:

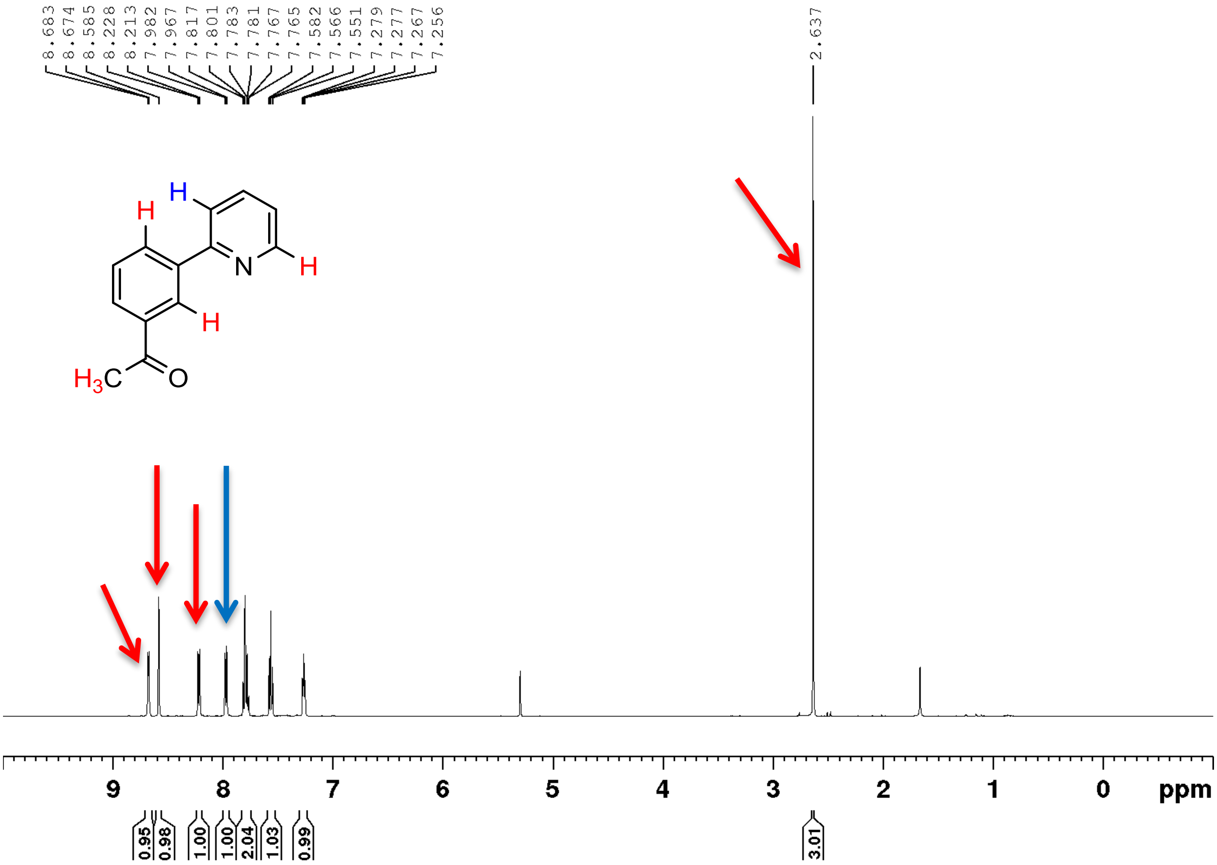

Enlargement of relevant area:

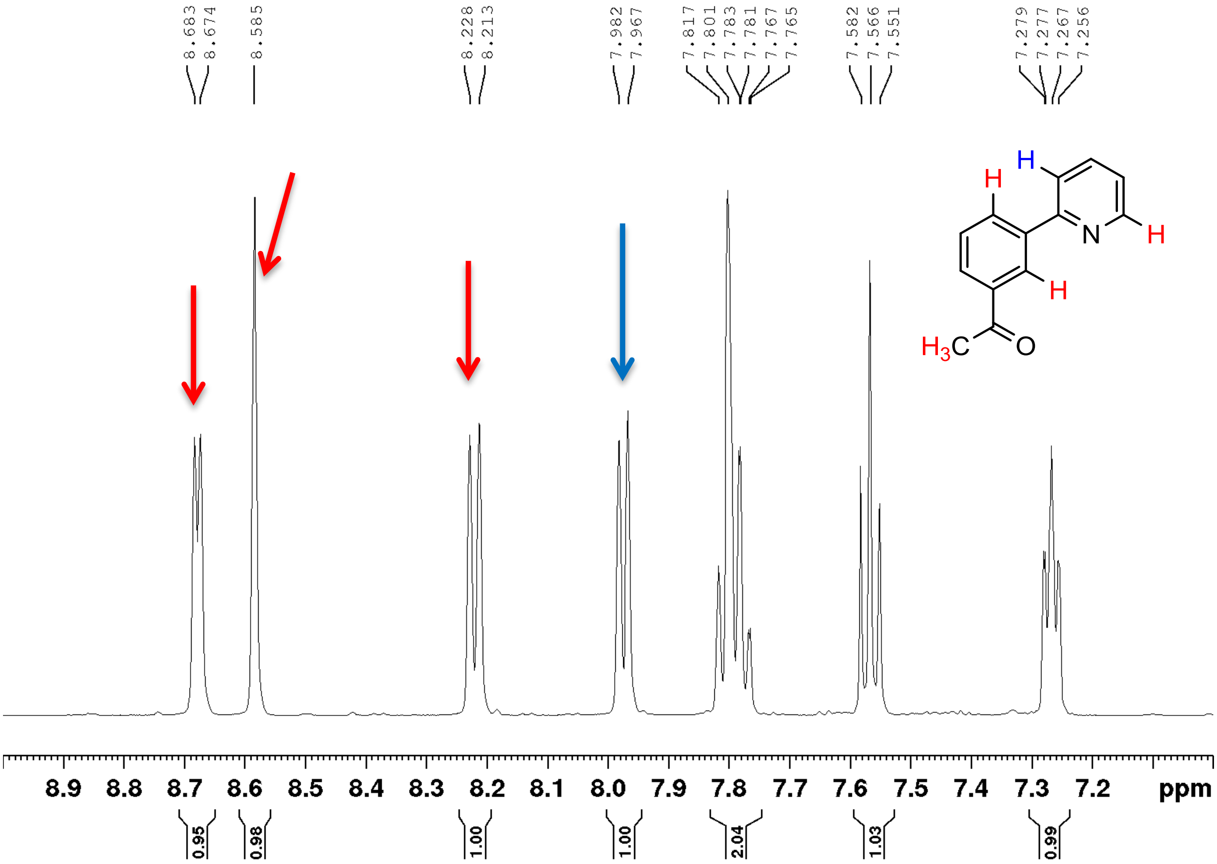

^{1}H-NMR (400 MHz, CD_2Cl_2, mesitylene) Spectra of deuterated compound **40** following the KOD procedure for 16 h: Yield: >99%

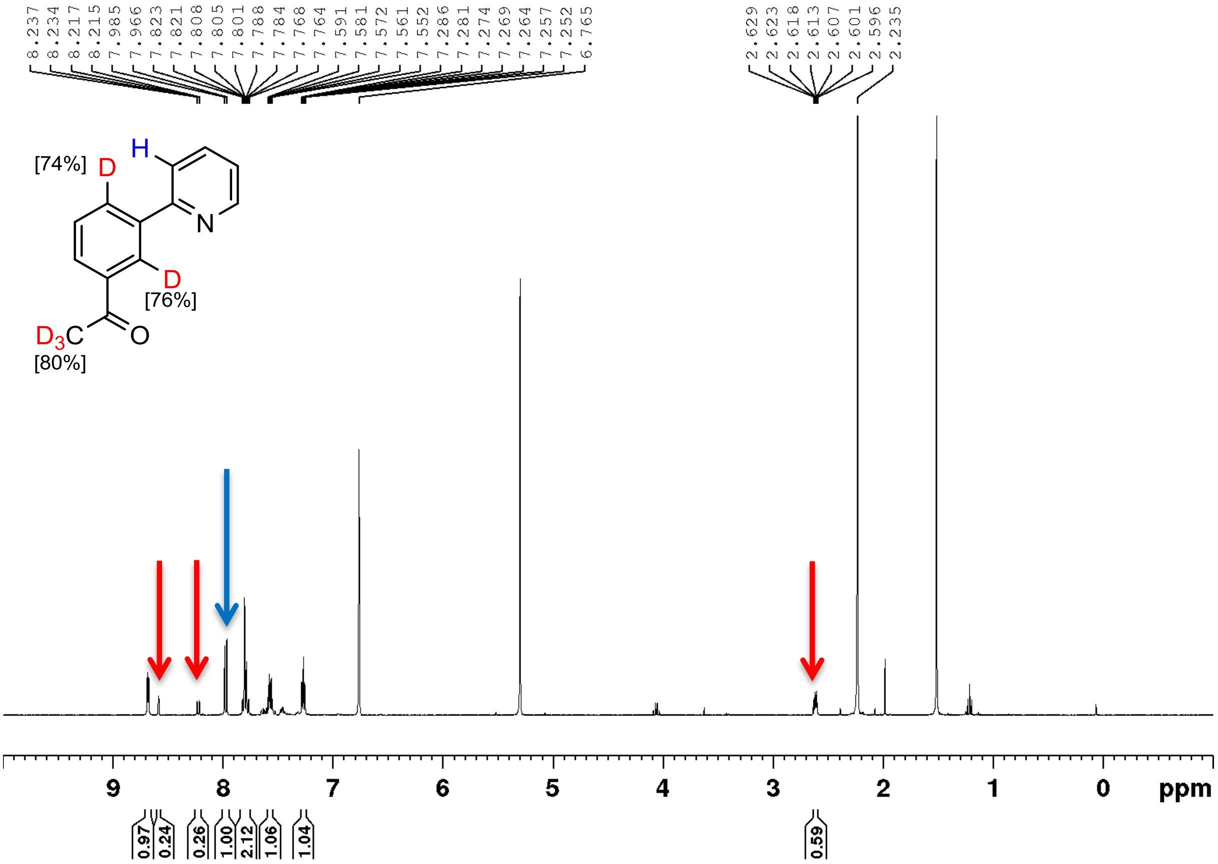

Enlargement of relevant area:

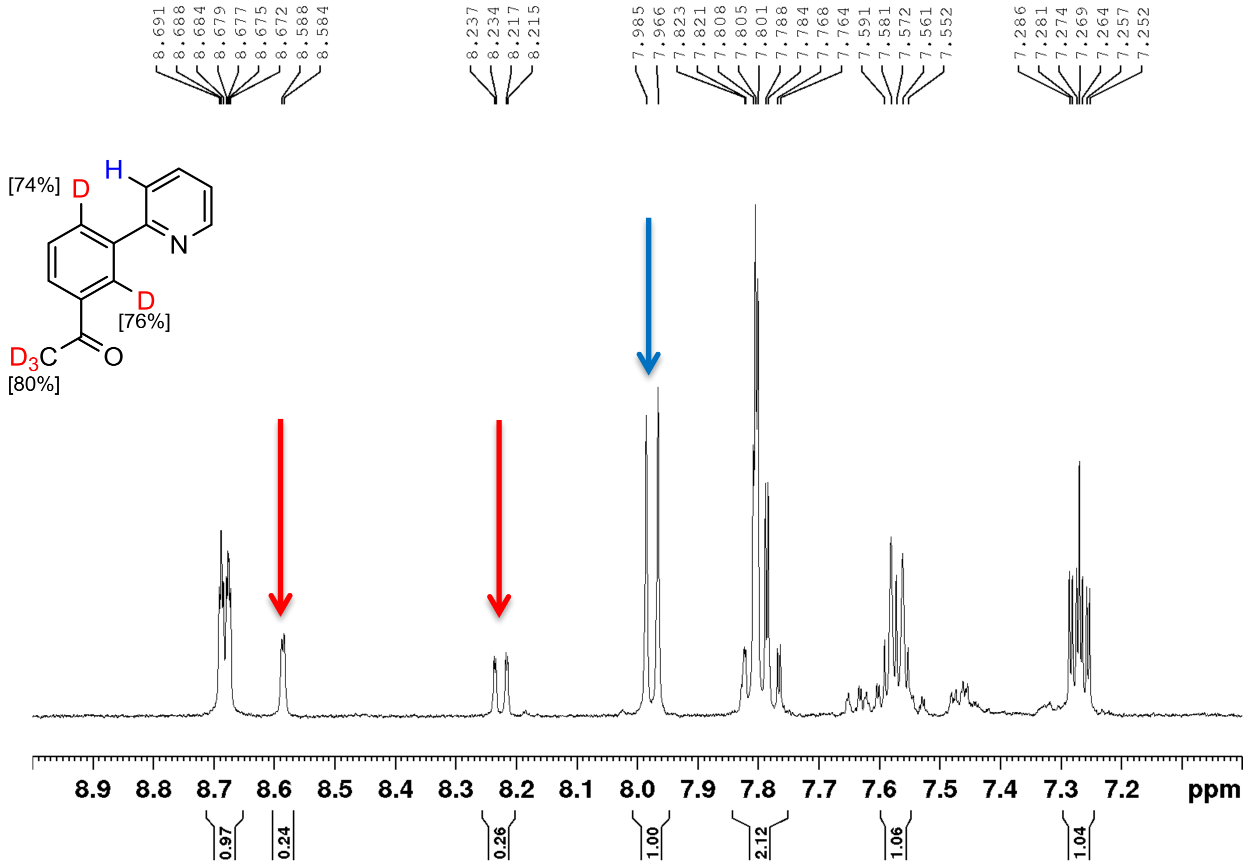

^{1}H-NMR (400 MHz, CD_2Cl_2, mesitylene) Spectra of deuterated compound **40** following the CuI procedure for 16 h: Yield: 82%

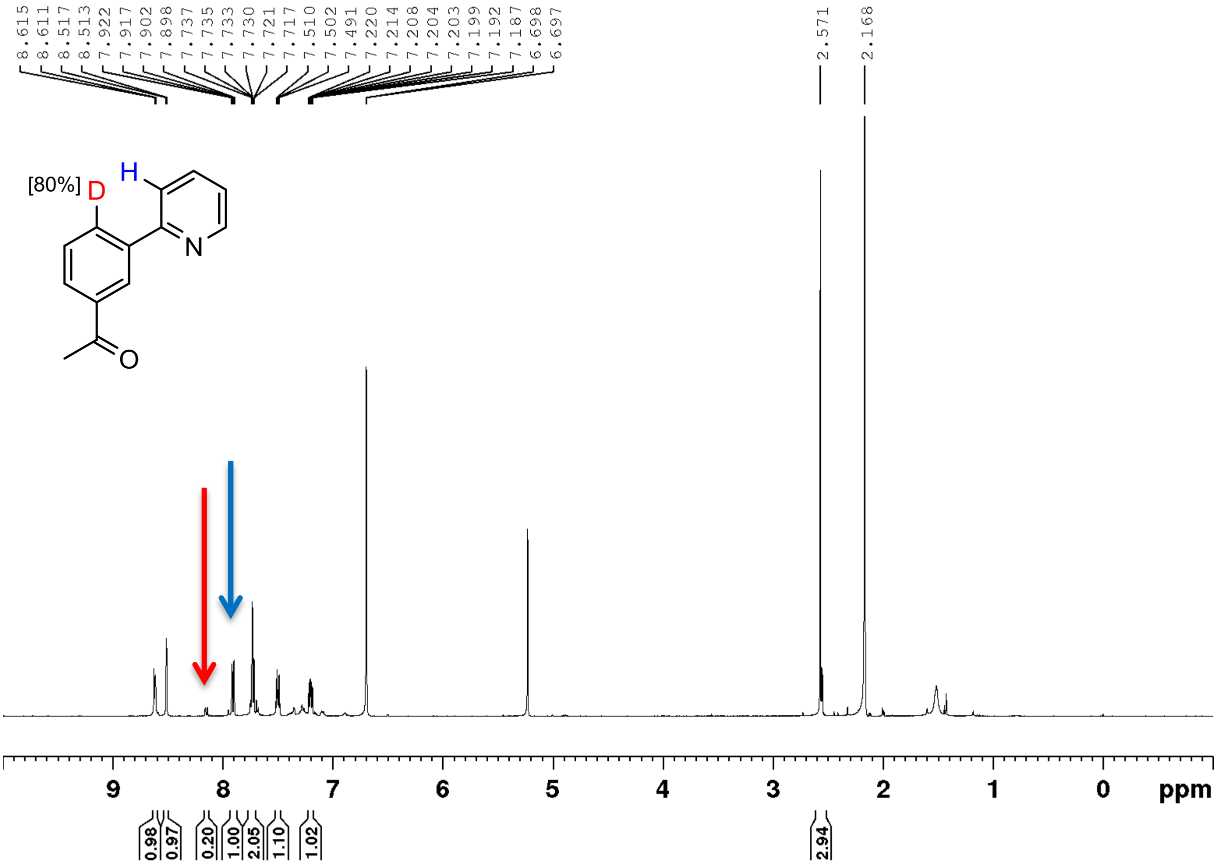

Enlargement of relevant area:

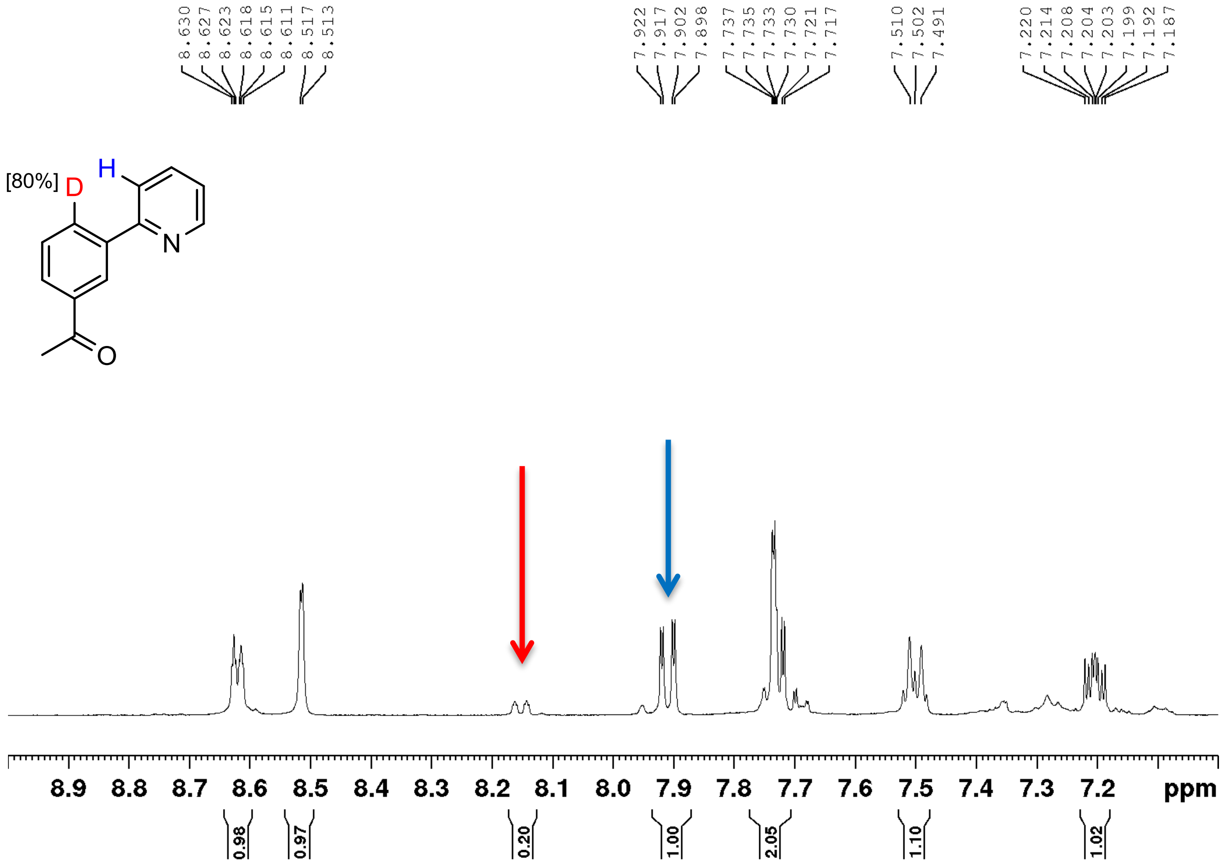

3.5.24 Piribedil 45

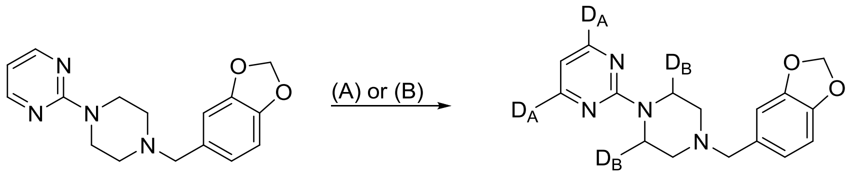

	t	D_A	D_B	Yield
cond. (A)	16 h	n.o.	49%	83%
	62 h	n.o.	67%	87%
	62 + 16 h	<10%	83%	49%
cond. (B)	16 h	21%	48%	>99%
	62 h	40%	73%	90%
	62 + 16 h	52%	80%	58%

2-(4-(benzo[d][1,3]dioxol-5-ylmethyl)piperazin-1-yl)pyrimidine, Piribedil 45 (I)

^{1}H-NMR (400 MHz, CD_2Cl_2) Spectra of pure compound **45**:

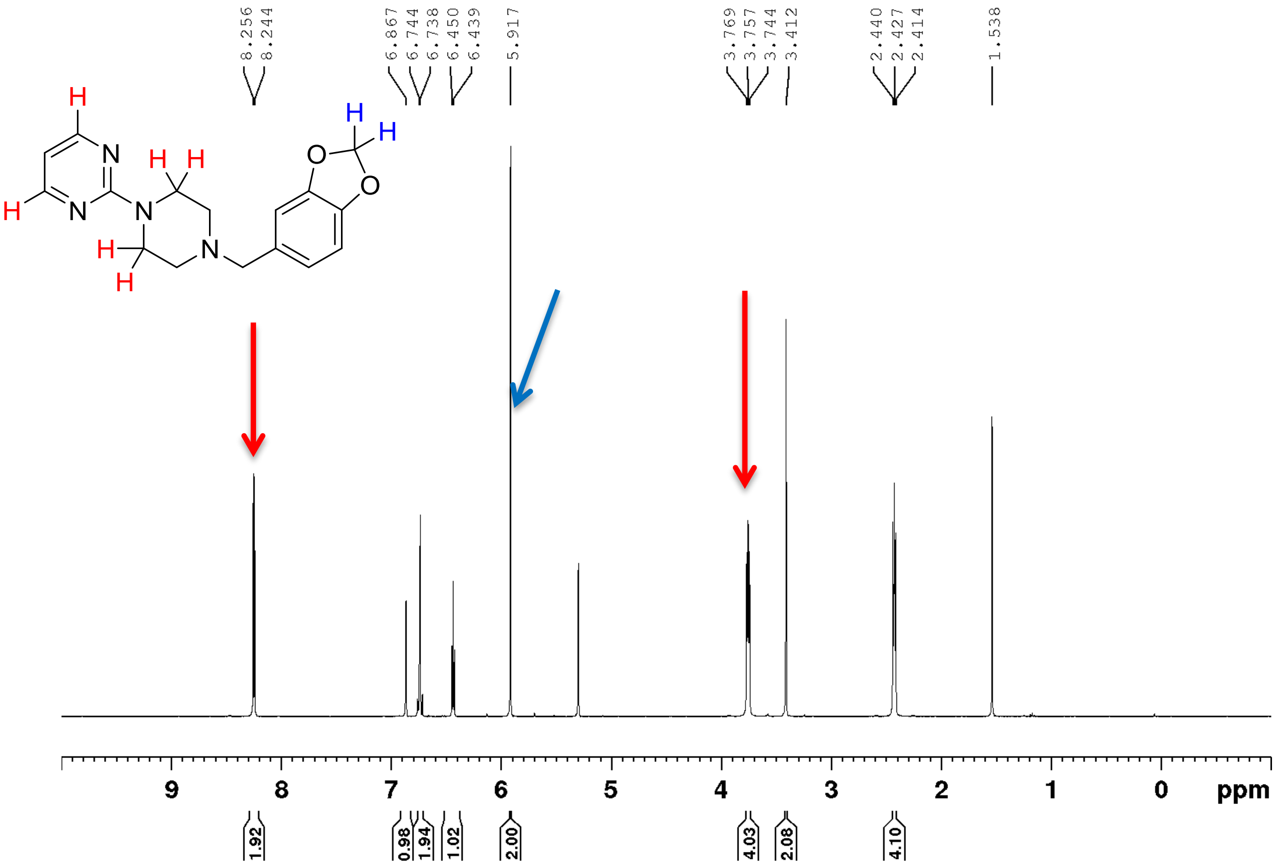

^{1}H-NMR (400 MHz, CD_2Cl_2) Spectra of deuterated compound **45** following the CuI procedure for 16 h: Yield: 83%.

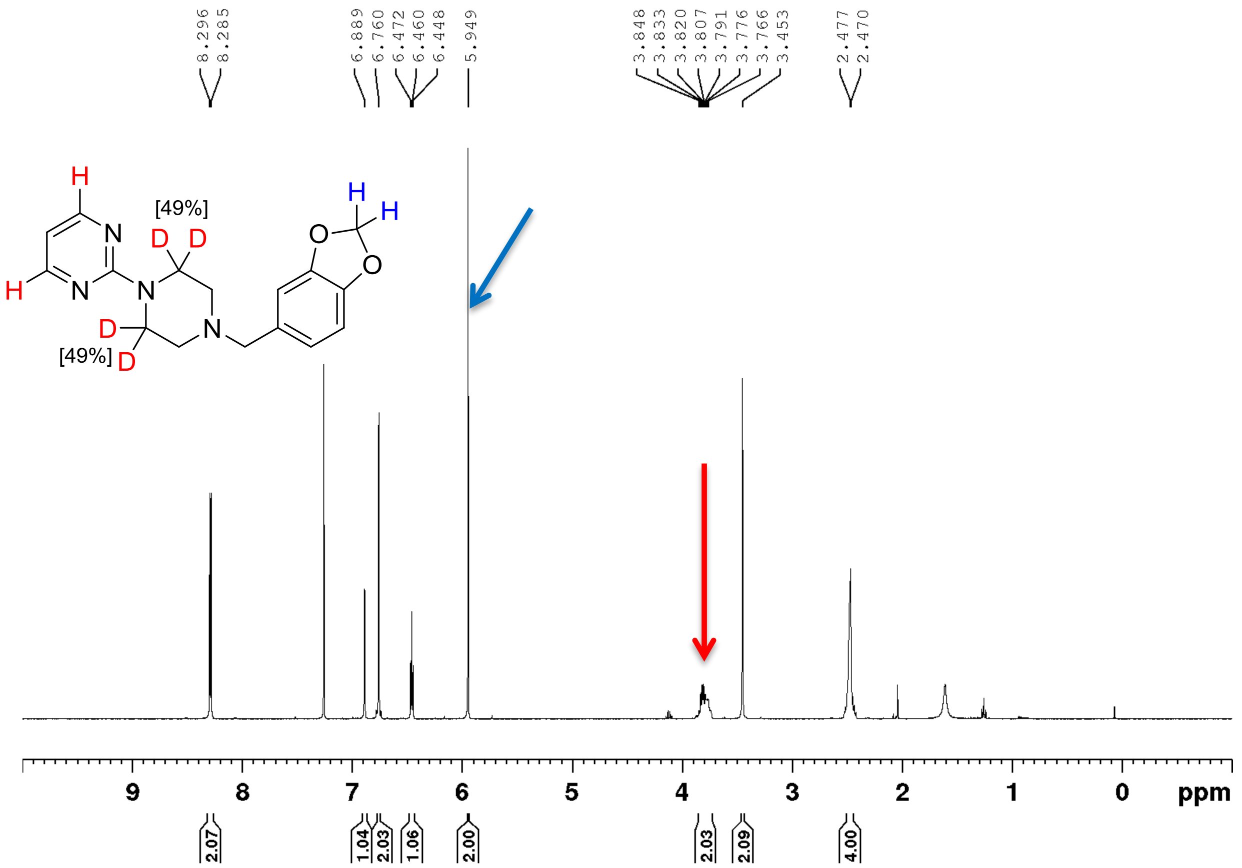

^{1}H-NMR (400 MHz, CD_2Cl_2) Spectra of deuterated compound **45** following the CuI procedure for 62 h: Yield: 87%.

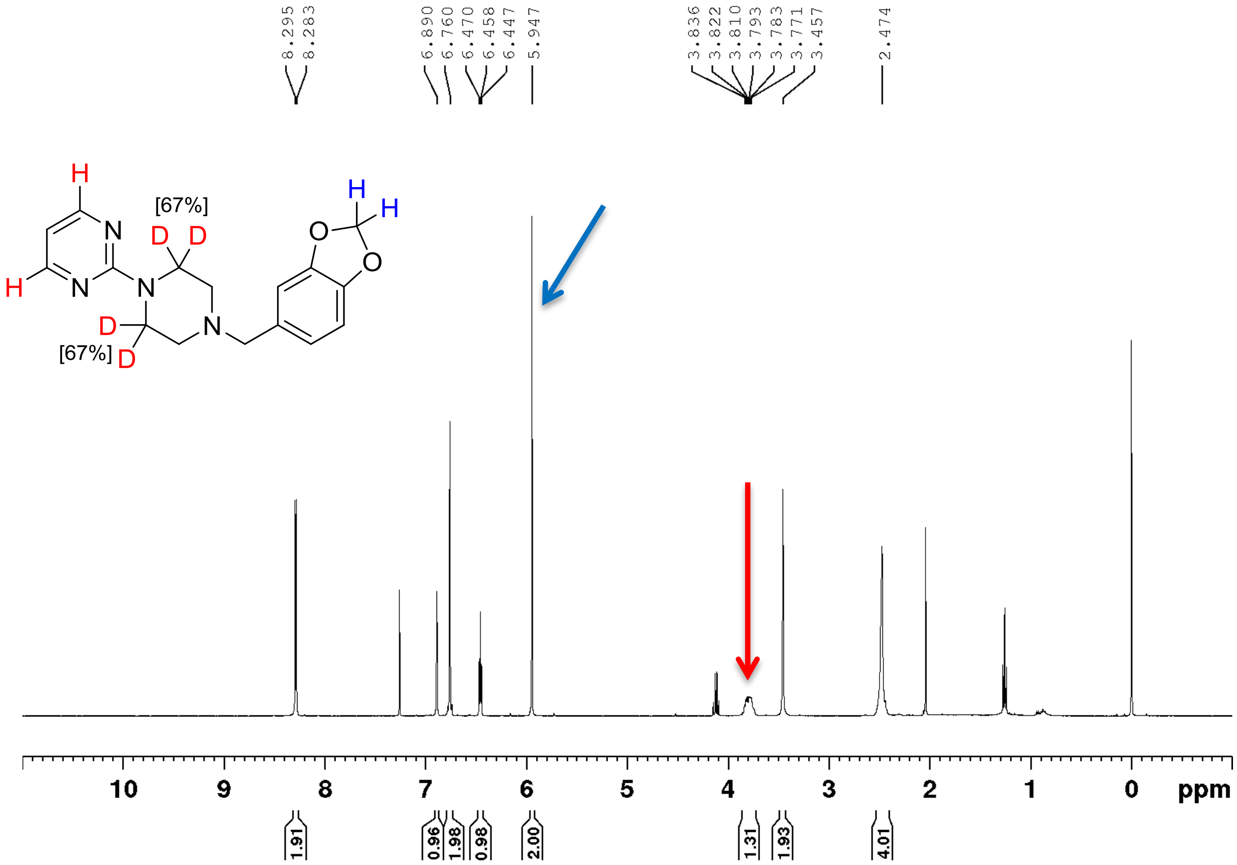

^{1}H-NMR (400 MHz, CD_2Cl_2) Spectra of deuterated compound **45** following the CuI procedure for 62 h and another subsequent run for 16 h: Yield: 49%.

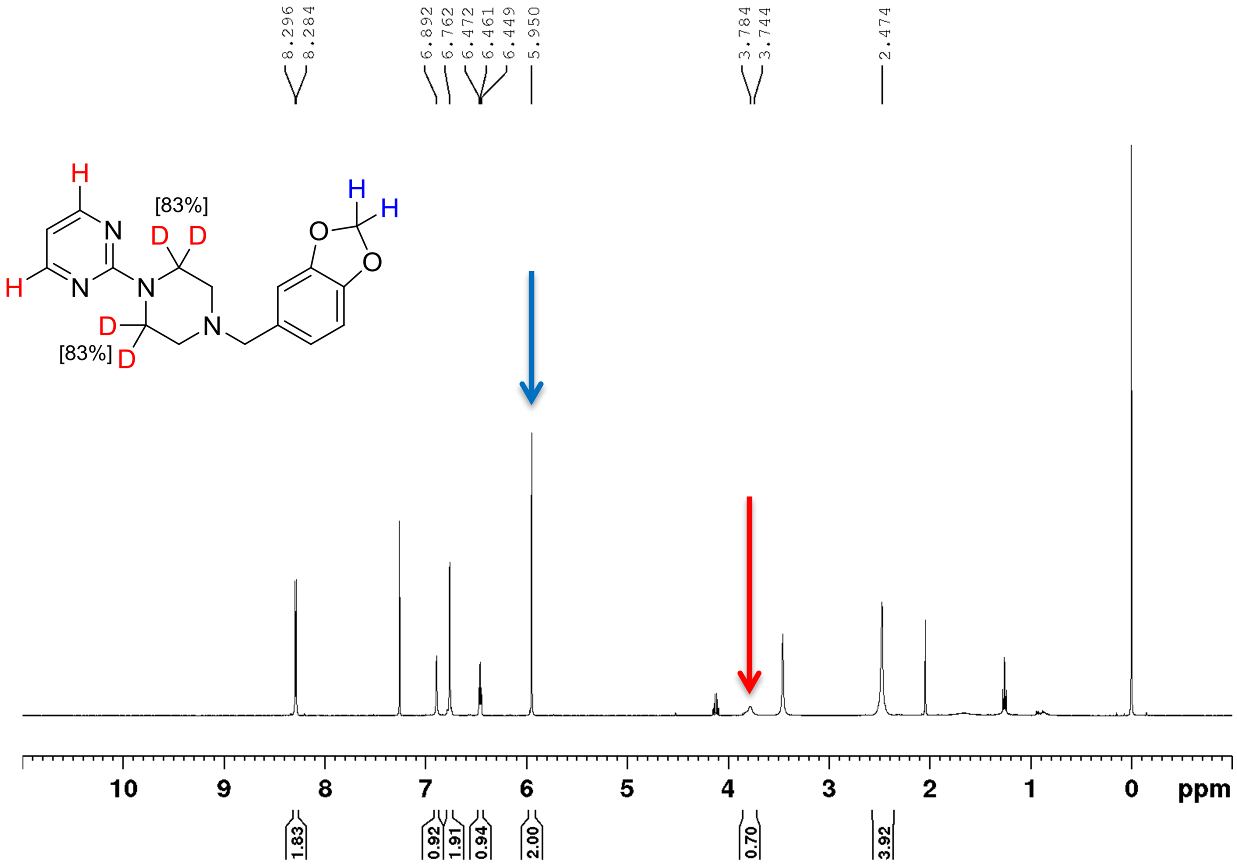

^{1}H-NMR (400 MHz, CD_2Cl_2) Spectra of deuterated compound **45** following the KOD/Zn procedure for 16 h: Yield: >99%

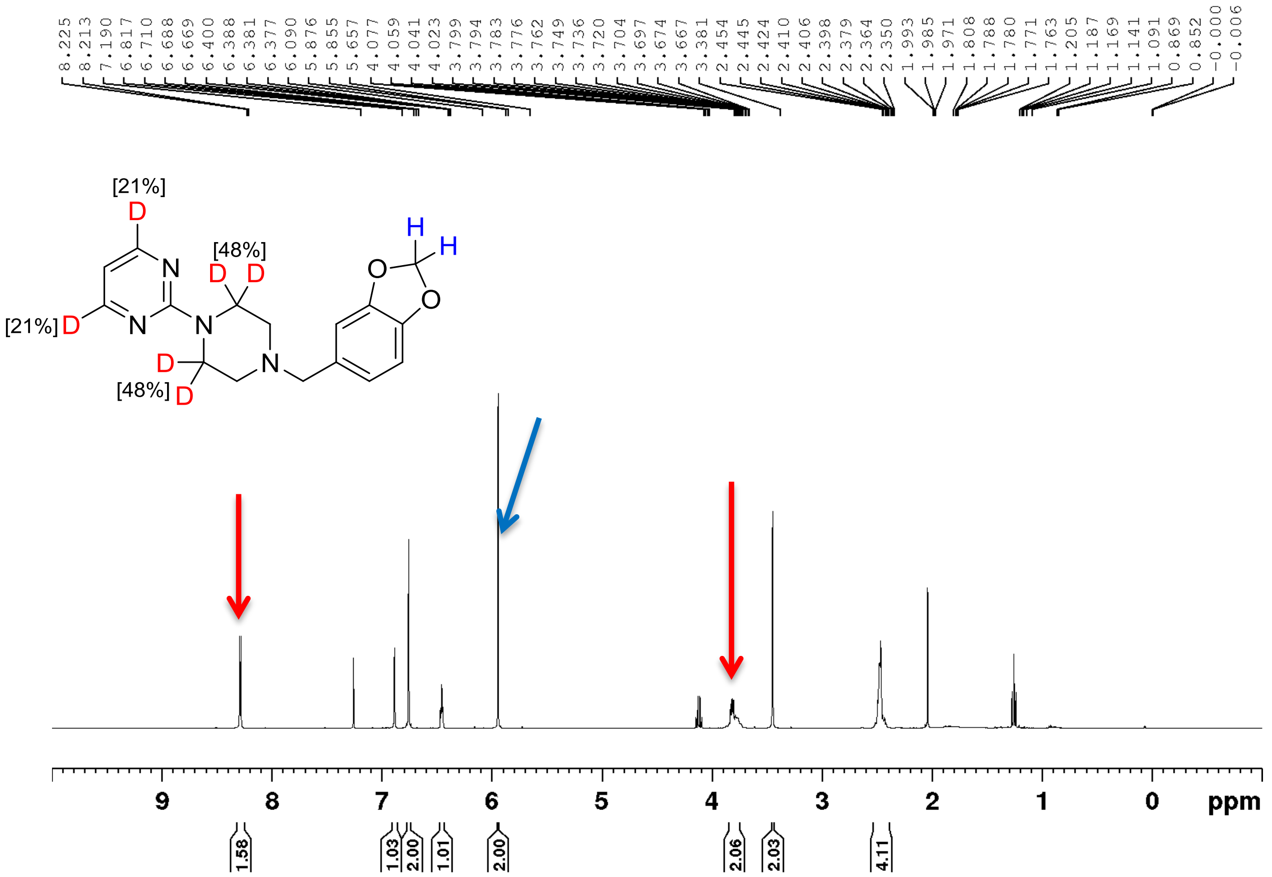

^{1}H-NMR (400 MHz, CD_2Cl_2) Spectra of deuterated compound **45** following the KOD/Zn procedure for 62 h: Yield: 90%

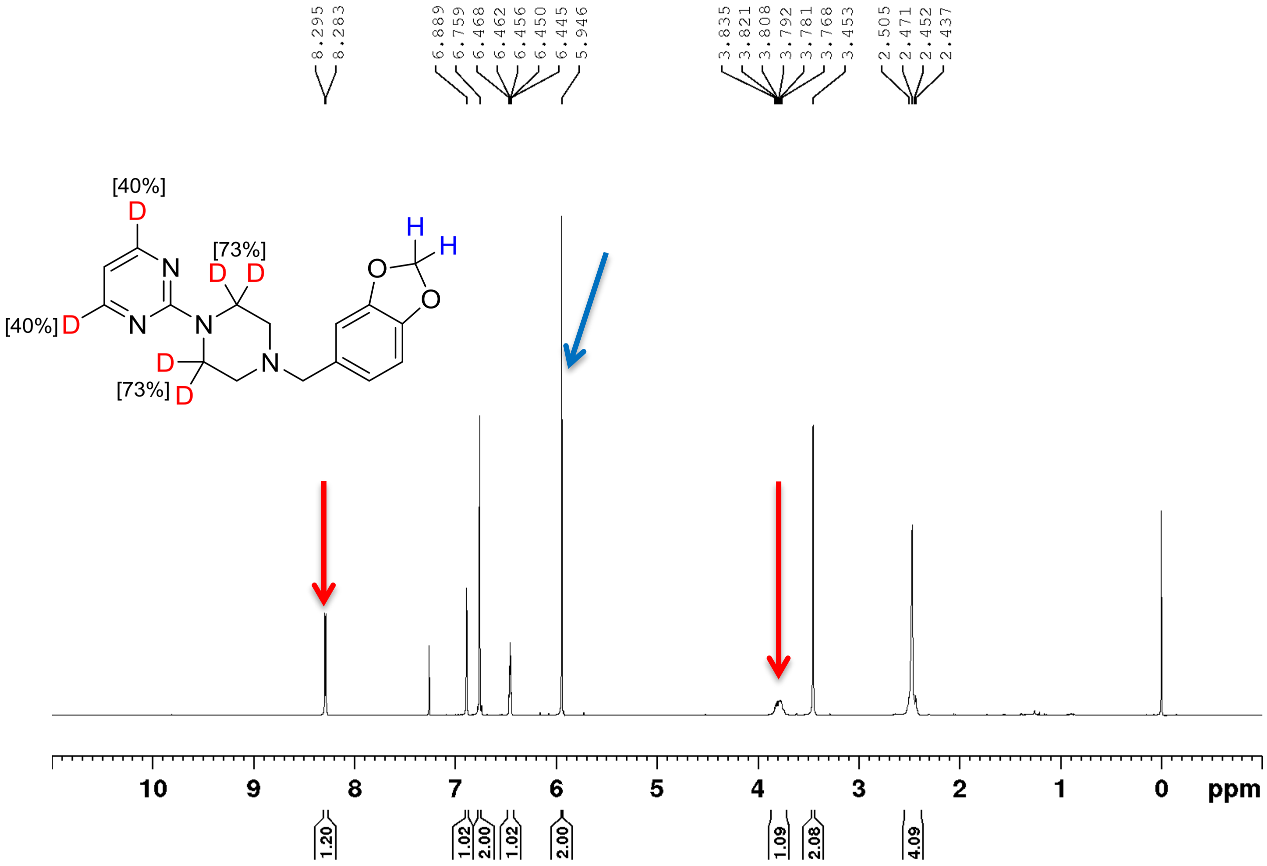

^{1}H-NMR (400 MHz, CD_2Cl_2) Spectra of deuterated compound **45** following the KOD/Zn procedure for 62 h and another subsequent run for 16 h: Yield: 58%

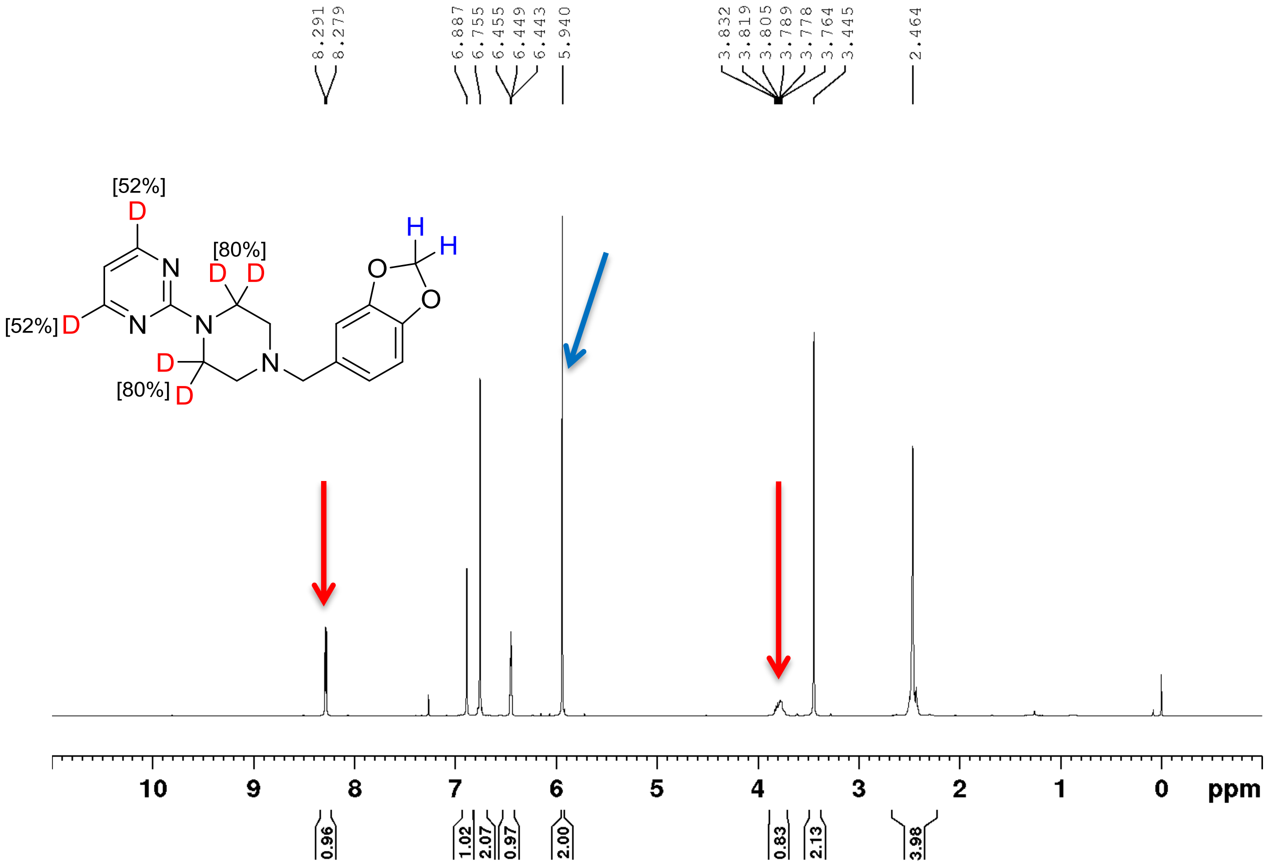

3.5.25 Boscalid 46

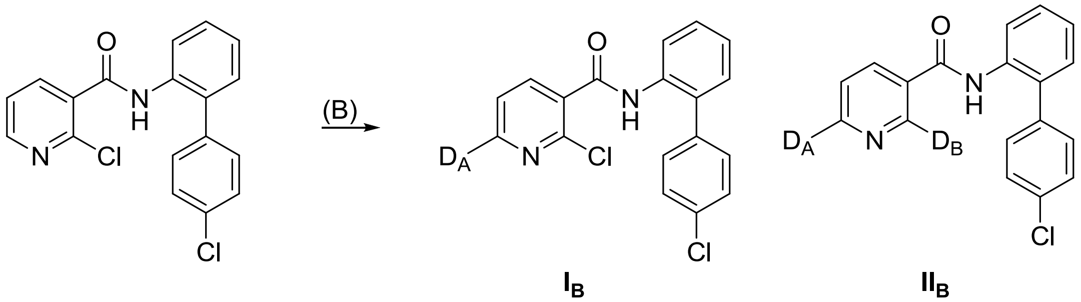

	I_B					II_B			
	t	D_A	D_B	D_C	Yield	D_A	D_B	D_C	Yield
cond. (B)	16 h	31%	n.o.	n.o.	54%	55%	65%	n.o.	35%

^{1}H-NMR (400 MHz, CD_2Cl_2) Spectra of pure compound **46**:

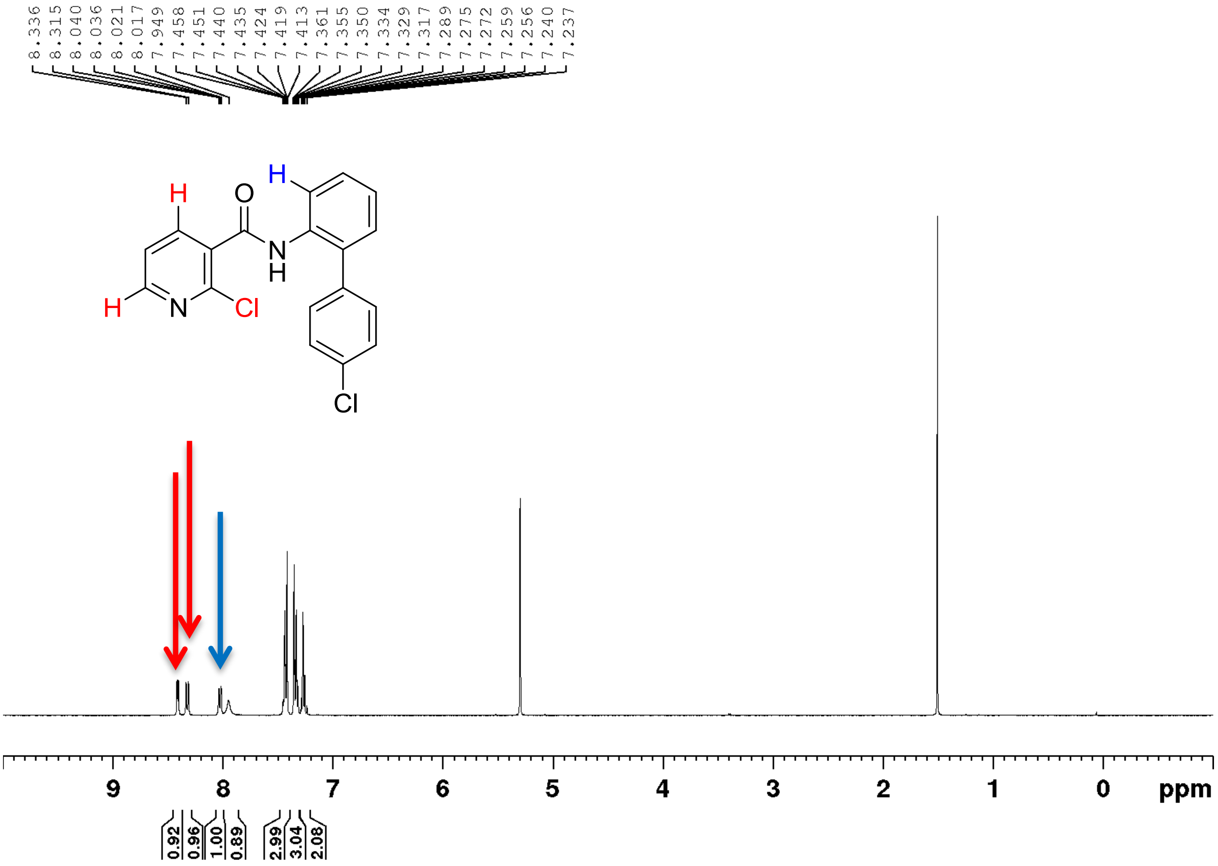

Enlargement of relevant area:

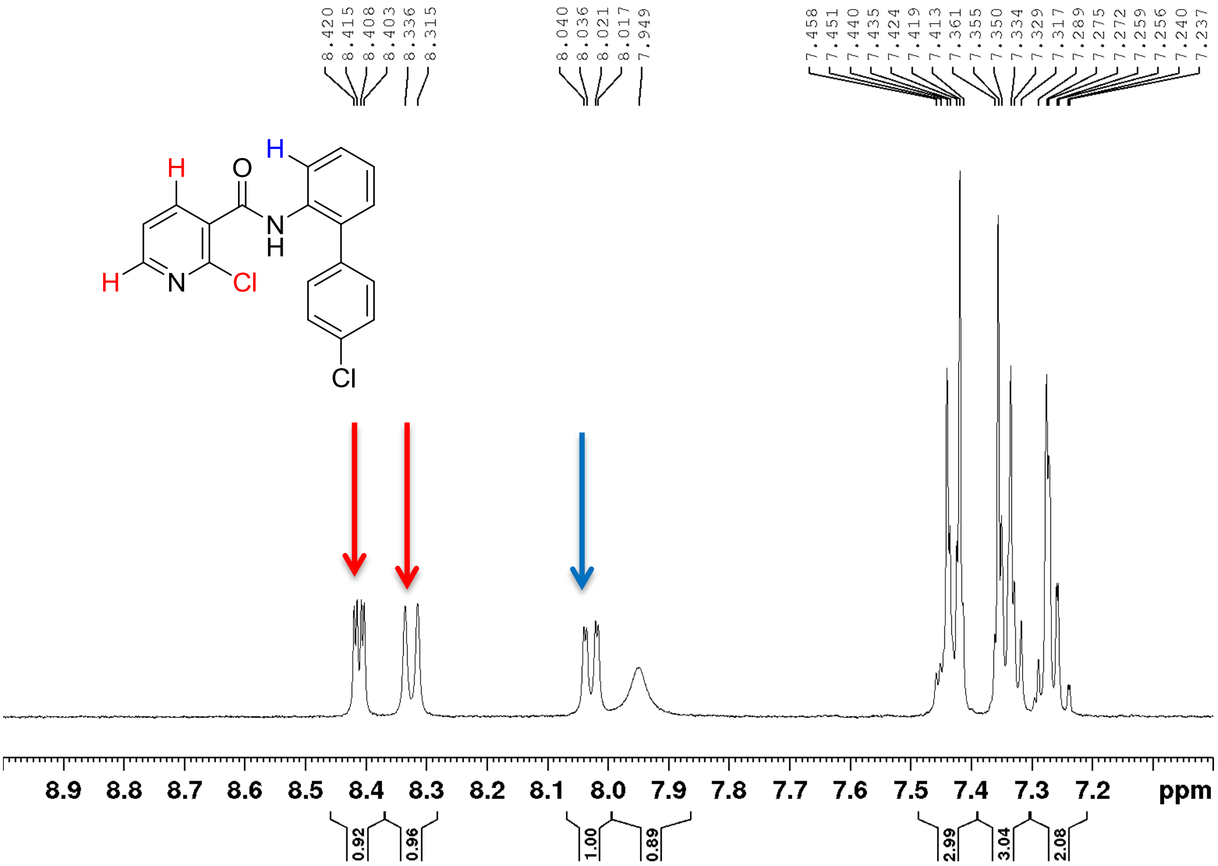

^{1}H-NMR (400 MHz, CD_2Cl_2) Spectra of deuterated compound 46 following the KOD/Zn procedure for 16 h: Yield: 54%

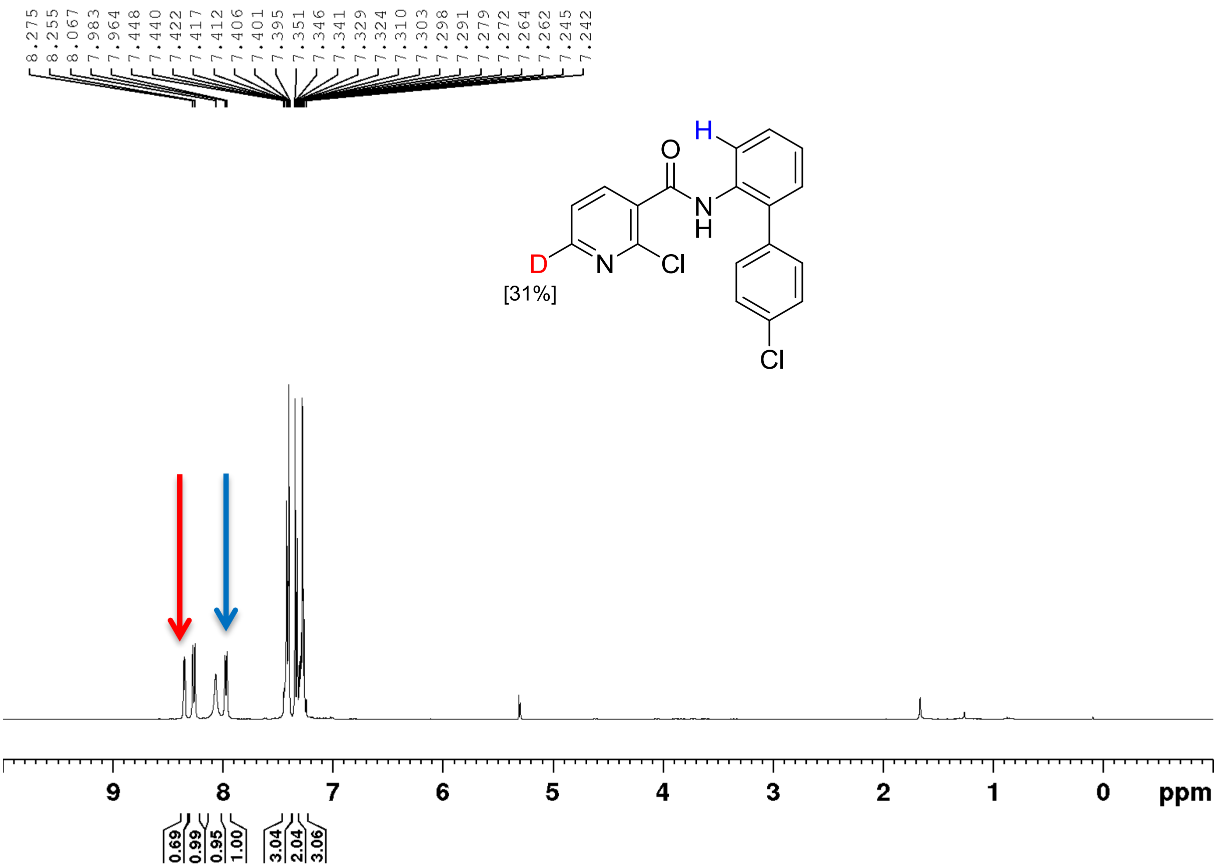

Enlargement of relevant area:

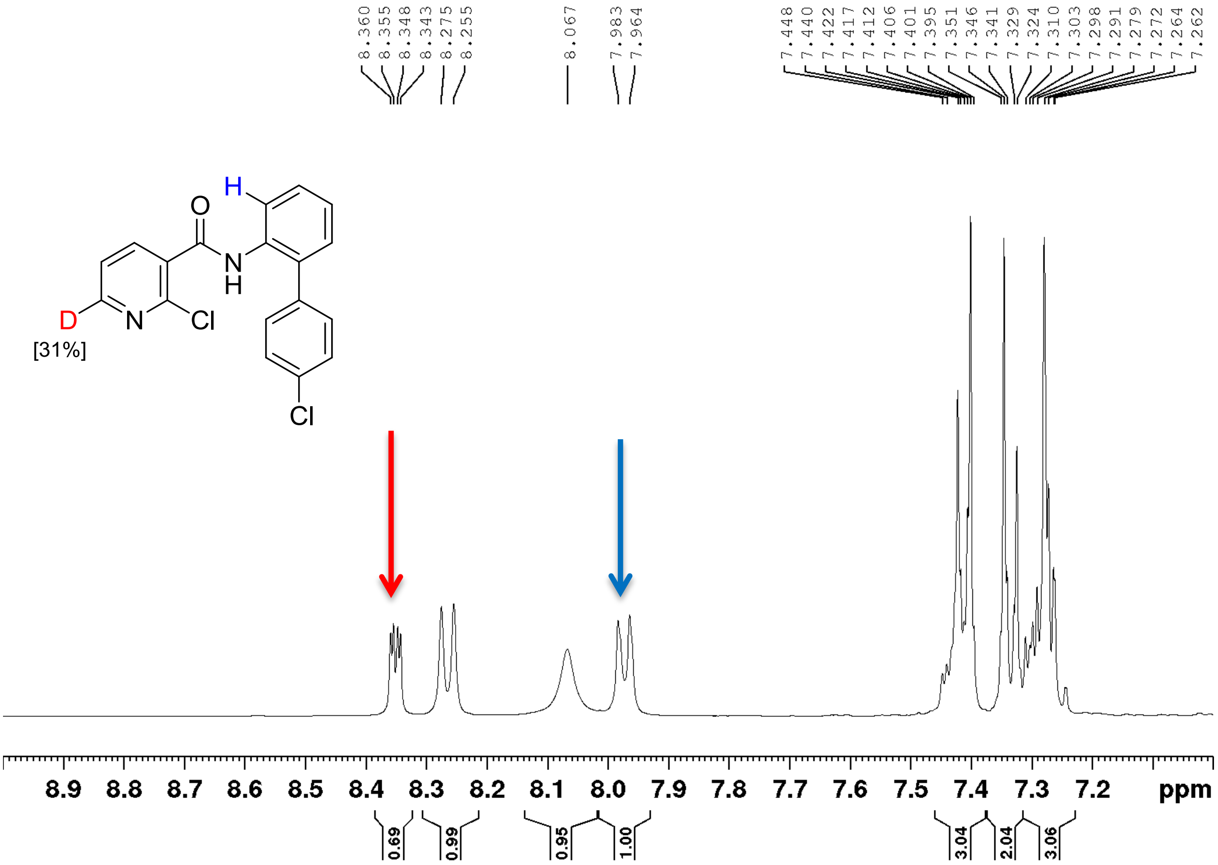

^{1}H-NMR (400 MHz, CD_2Cl_2) Spectra of deuterated compound **47** following the KOD/Zn procedure for 16 h: Yield: 35%

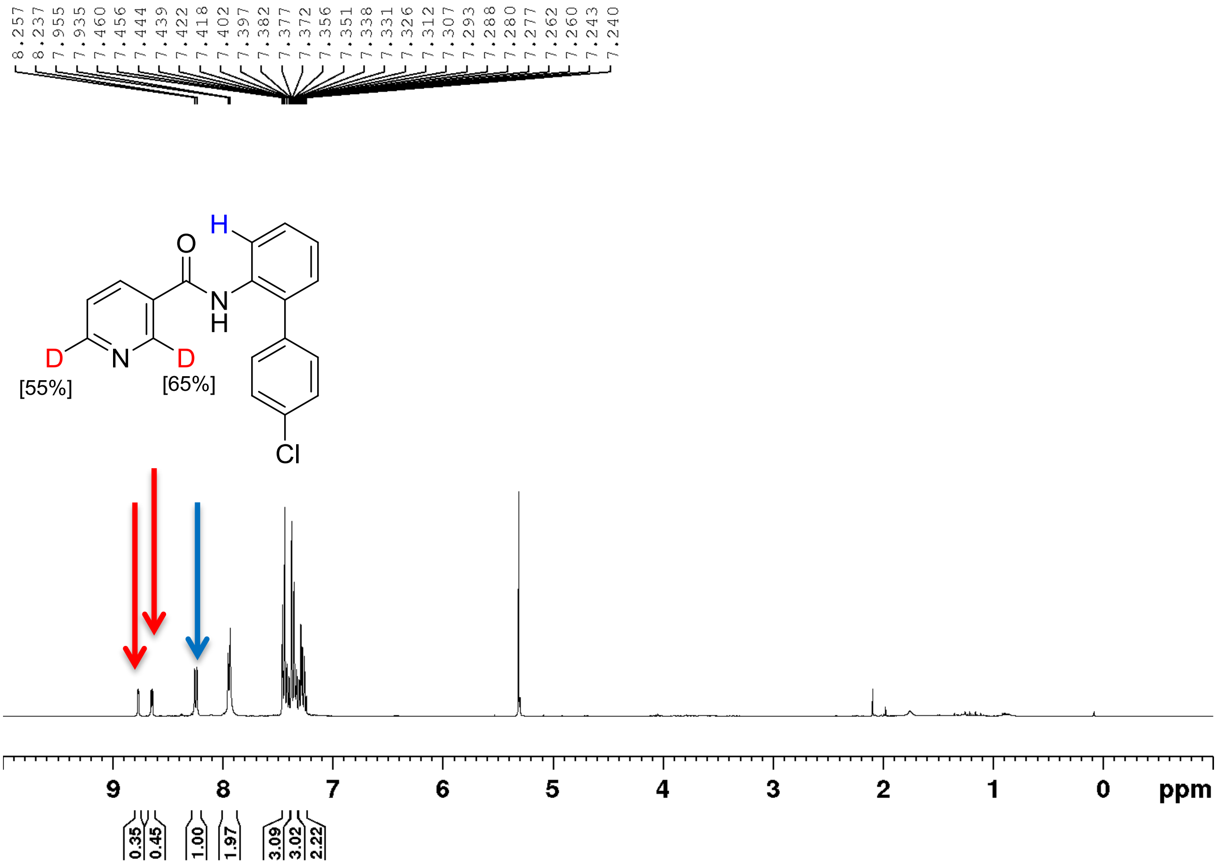

Enlargement of relevant area:

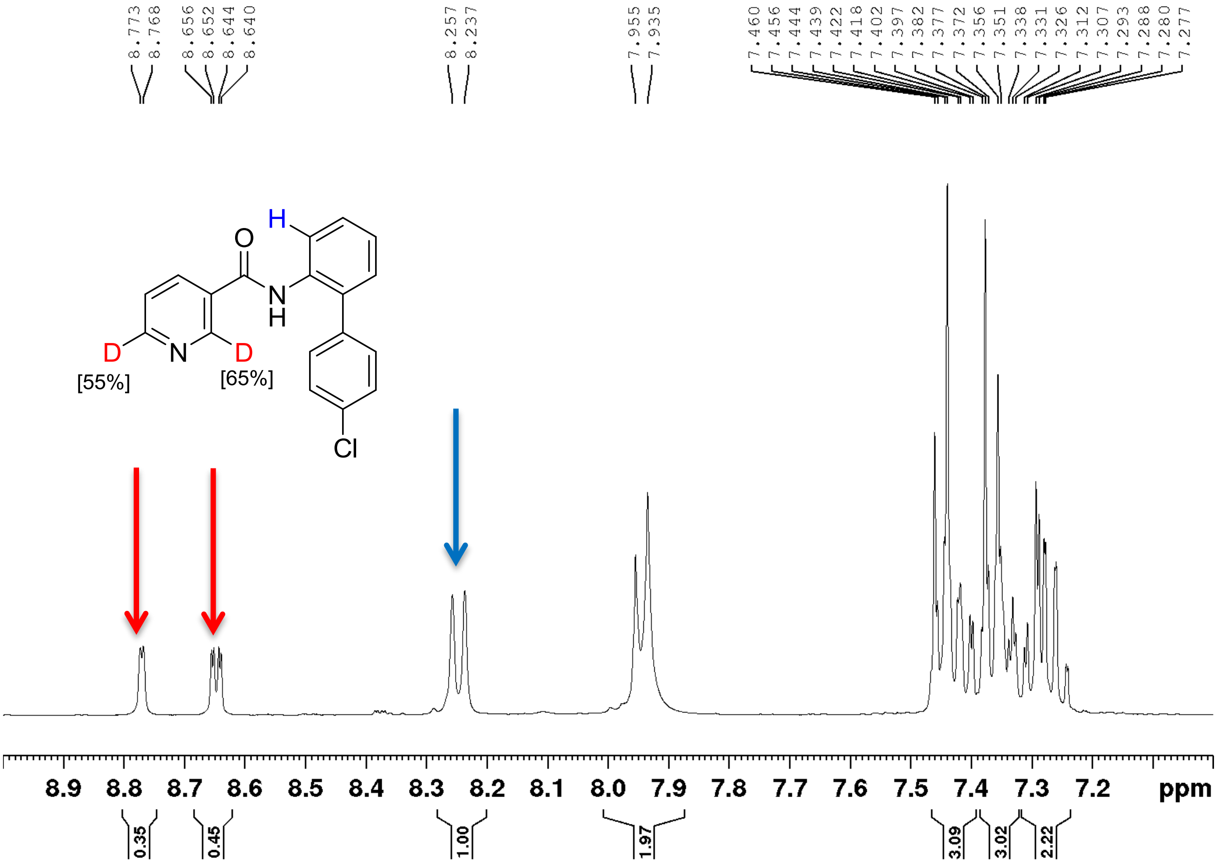

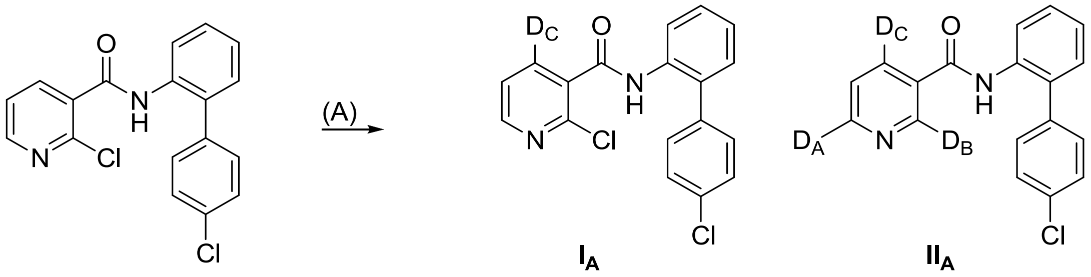

	I_A					II_A			
	t	D_A	D_B	D_C	Yield	D_A	D_B	D_C	Yield
cond. (A)	16 h	n.o.	n.o.	32%	50%	24%	68%	62%	40%

^{1}H-NMR (400 MHz, CD_2Cl_2) Spectra of deuterated compound **46** following the CuI procedure for 16 h: Yield: 50%.

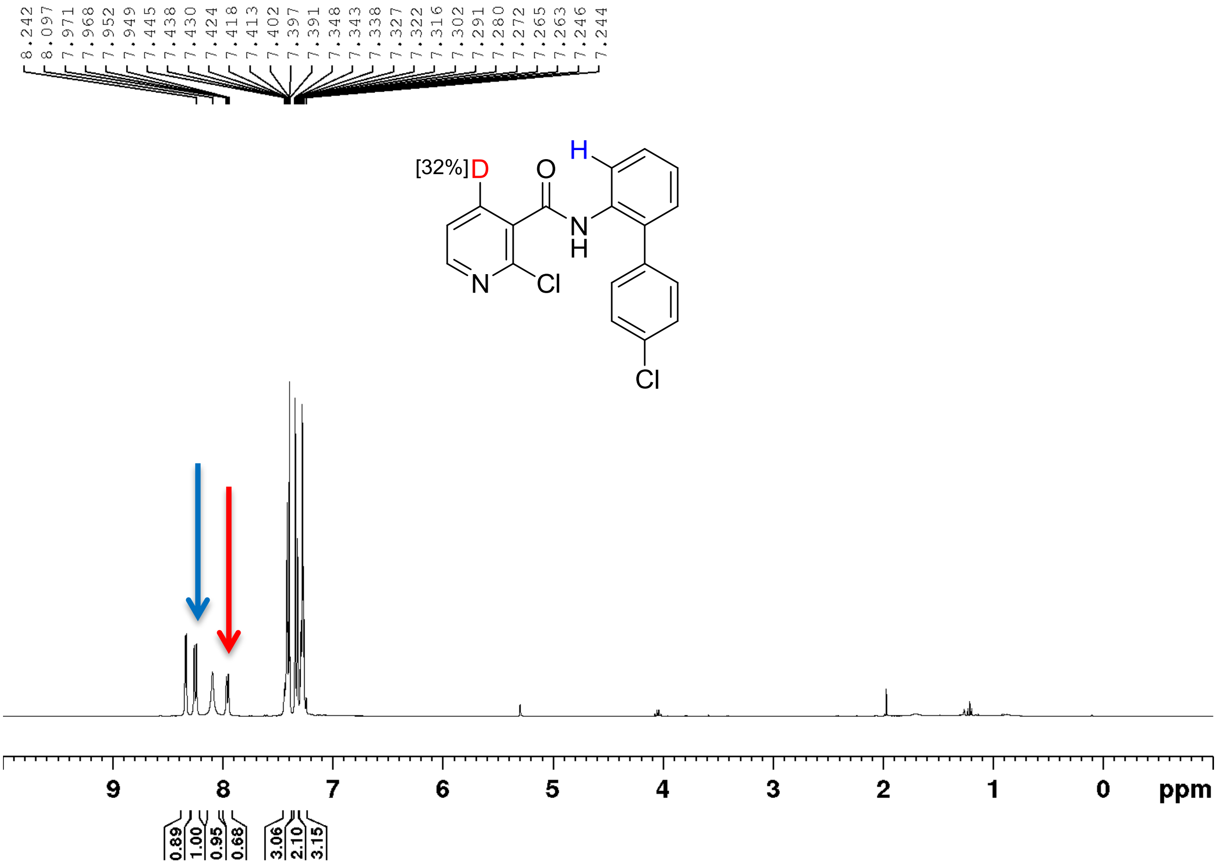

Enlargement of relevant area:

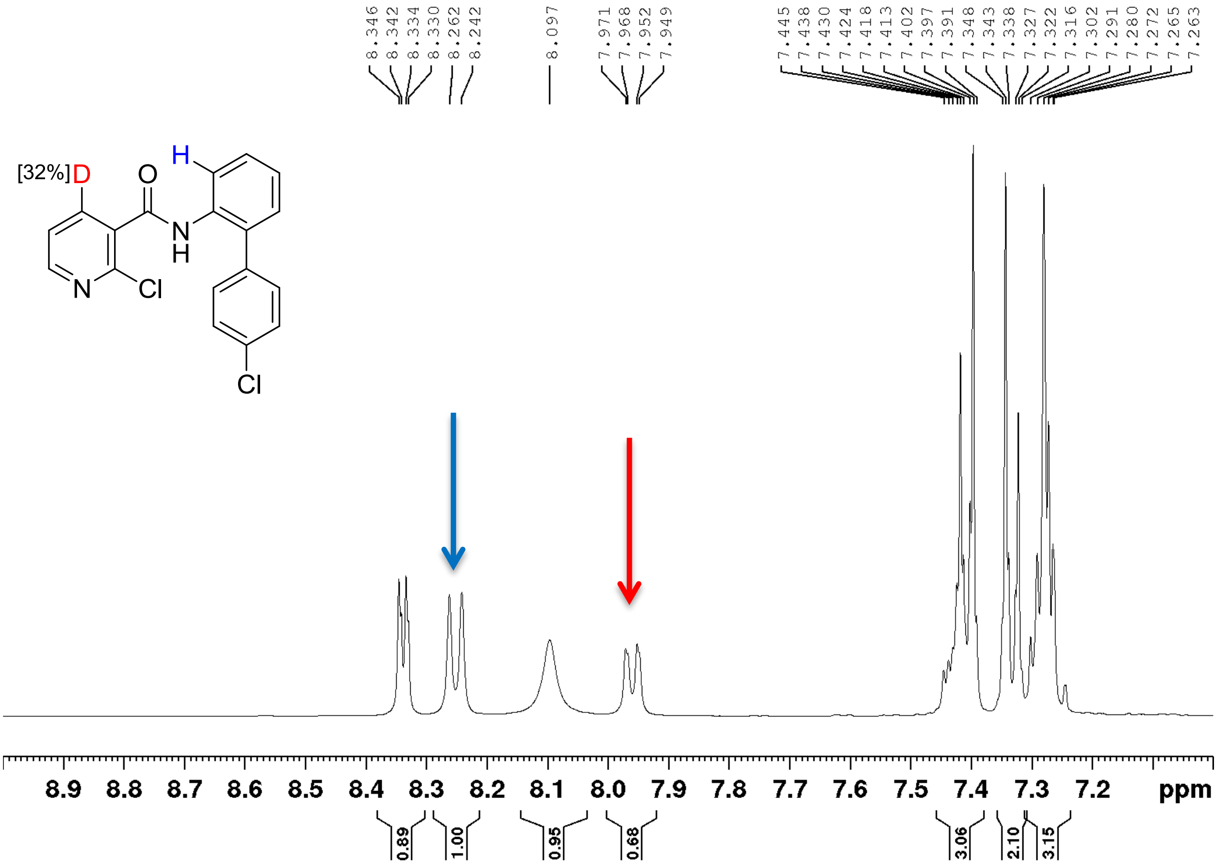

^{1}H-NMR (400 MHz, CD_2Cl_2) Spectra of deuterated compound **47** following the CuI procedure for 16 h: Yield: 40%.

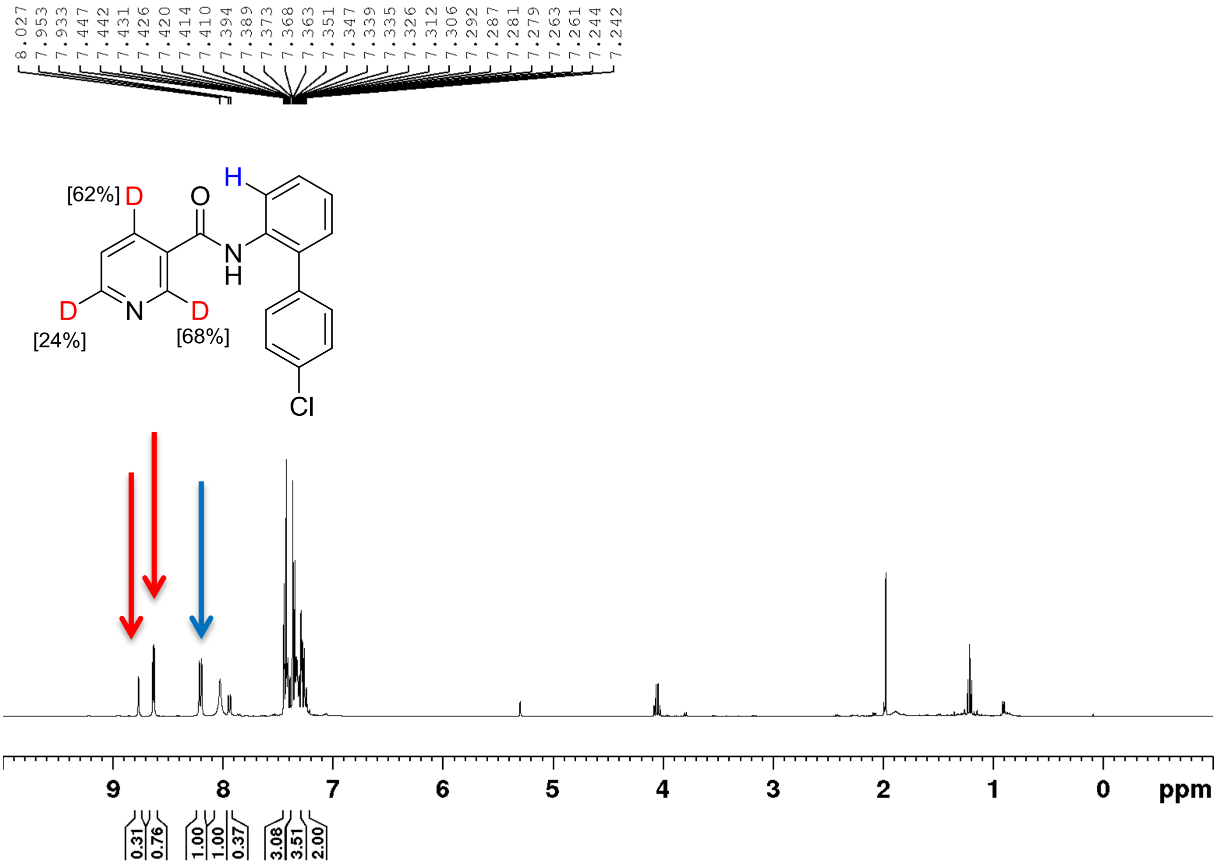

Enlargement of relevant area:

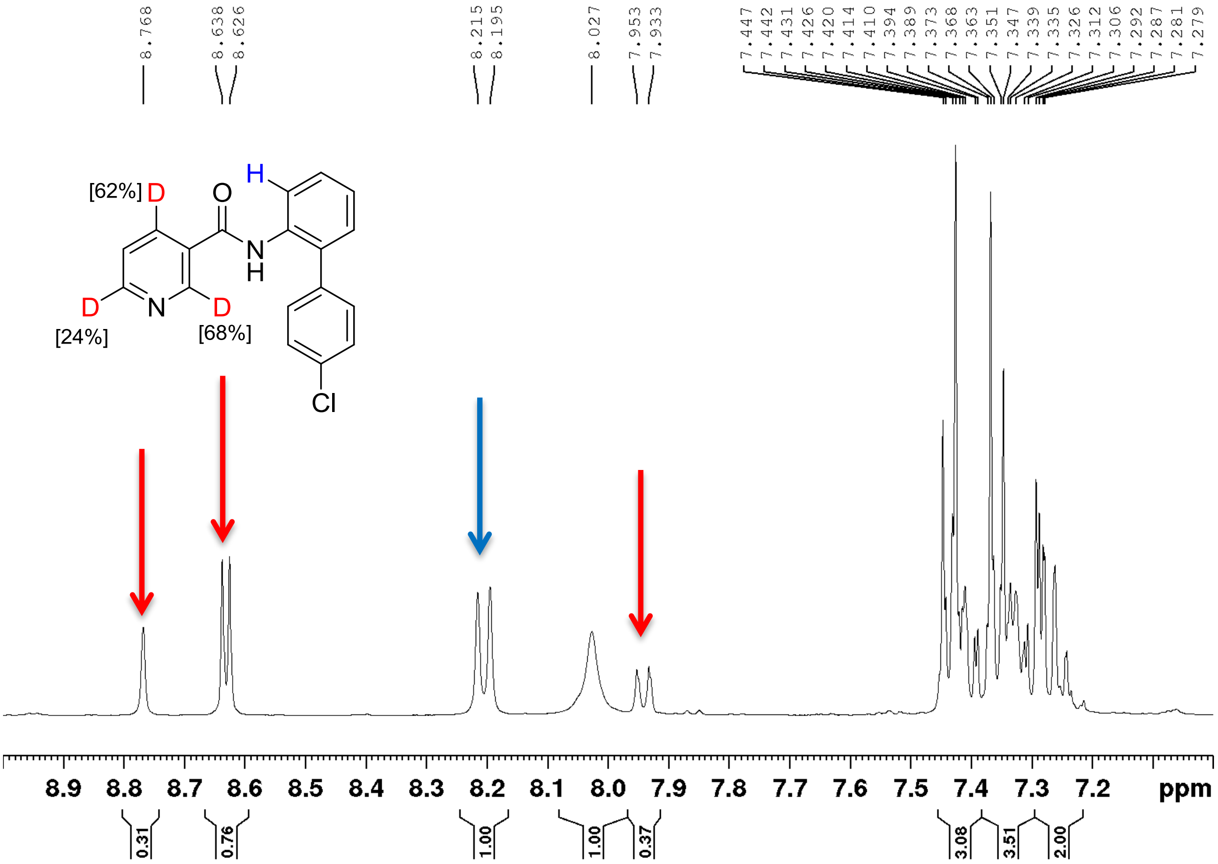

4. Synthesis of literature-known Ru-complexes

^{1}H and ^{31}P NMR-data for known Ru-H-complexes $RuH(OH)(solvent)(PPh_3)_3$ **4**, $Ru_2(H)_2(\mu$-$OH)_2(solvent)_2(PPh_3)_4$ **48**, $RuH_2(H_2)(PPh_3)_3$ **3** and $RuHI(PPh_3)_3$ **2** were in accordance with those given in the literature.[11-14] Characteristic hydride-signals, as well as ^{31}P-signals for the given complexes in THF-d_8 or CD_2Cl_2 were recorded as reference and are listed in table 1.

Table 1: Hydride- and ^{31}P-signals of ruthenium hydride-complexes in THF-d_8.

Complex	*Hydride* (ppm)	^{31}P (ppm)
**$RuH(OH)(solvent)(PPh_3)_3$ 4** [11]	-23.8 (t, *J* = 33.8 Hz)	73.8 (s)
	0.91 (s, 1H, Ru-OH)	
**$Ru_2(H)_2(\mu$-$OH)_2(solvent)_2(PPh_3)_4$ 48** [11]	-18.4 (t, *J* = 33.0 Hz)	43.5 (s)
**$RuH_2(H_2)(PPh_3)_3$ 3** [12]	-7.5 (br)	57.4 (s)
**$RuHI(PPh_3)_3$ 2** (CD_2Cl_2)[13]	-15.3 (q, J = 25.1 Hz)	56.7 (br)
**$(Ph_3P)_3Ru(\mu$-$H)_3RuH(PPh_3)_2$ 49** **[14]**	-10.9 (br)	75.0 (br.)
**(at 25 °C)**	-20.7 (t, *J* = 34.1 Hz)	55.8 (br)
**$(Ph_3P)_3Ru(\mu$-$H)_3RuH(PPh_3)_2$ 49** **[14]**	-9.0 (d, J = 67.7 Hz)	83.7 – 82.1 (m)
**(at -50 °C)**	-10.8 (t, J = 44.3 Hz)	73.3 – 71.7 (m)
	-11.8 (t, J = 46.8 Hz)	63.3 – 60.1 (m)
	-19.8 (t, J = 37.4 Hz)	59.0 (s)

4.1 $Ru_2(H)_2(\mu\text{-}OH)_2(solvent)_2(PPh_3)_4$ 48 and $RuH(OH)(solvent)(PPh_3)_3$ 4[11]

^{1}H-NMR (300 MHz, THF-d_8)

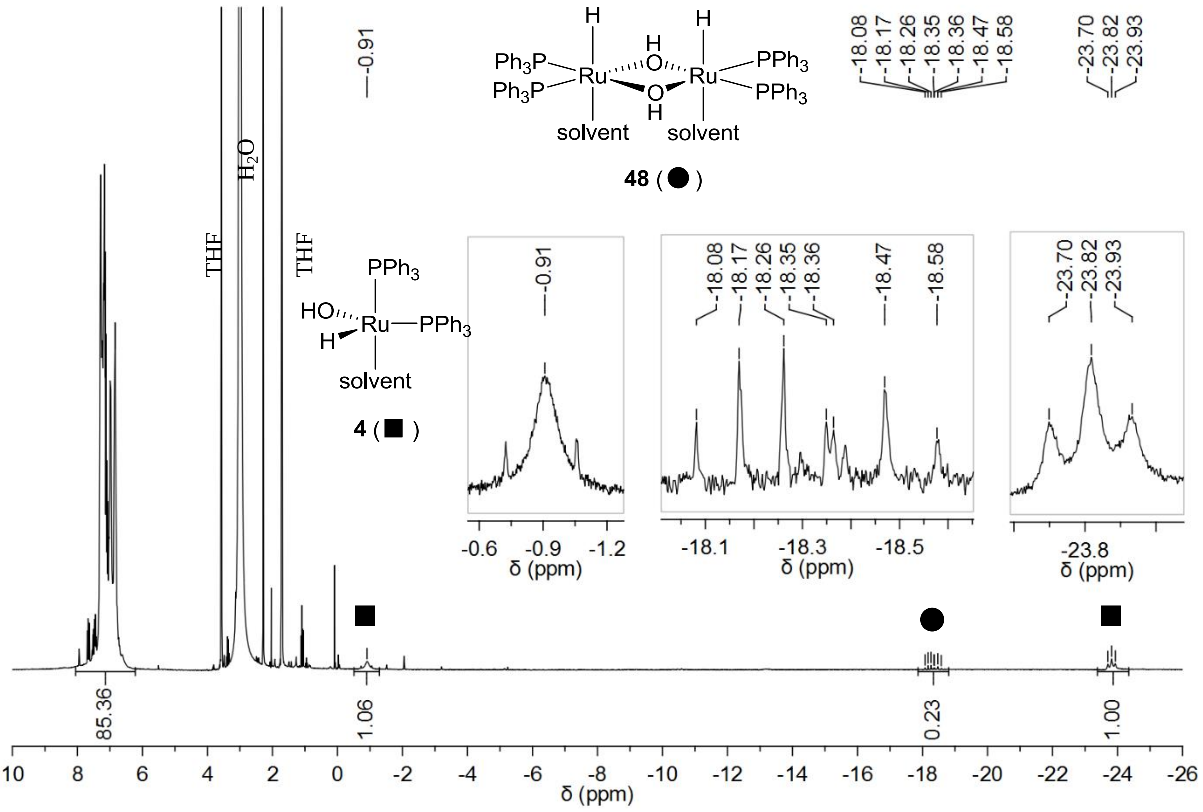

^{31}P-NMR (121 MHz, THF-d_8)

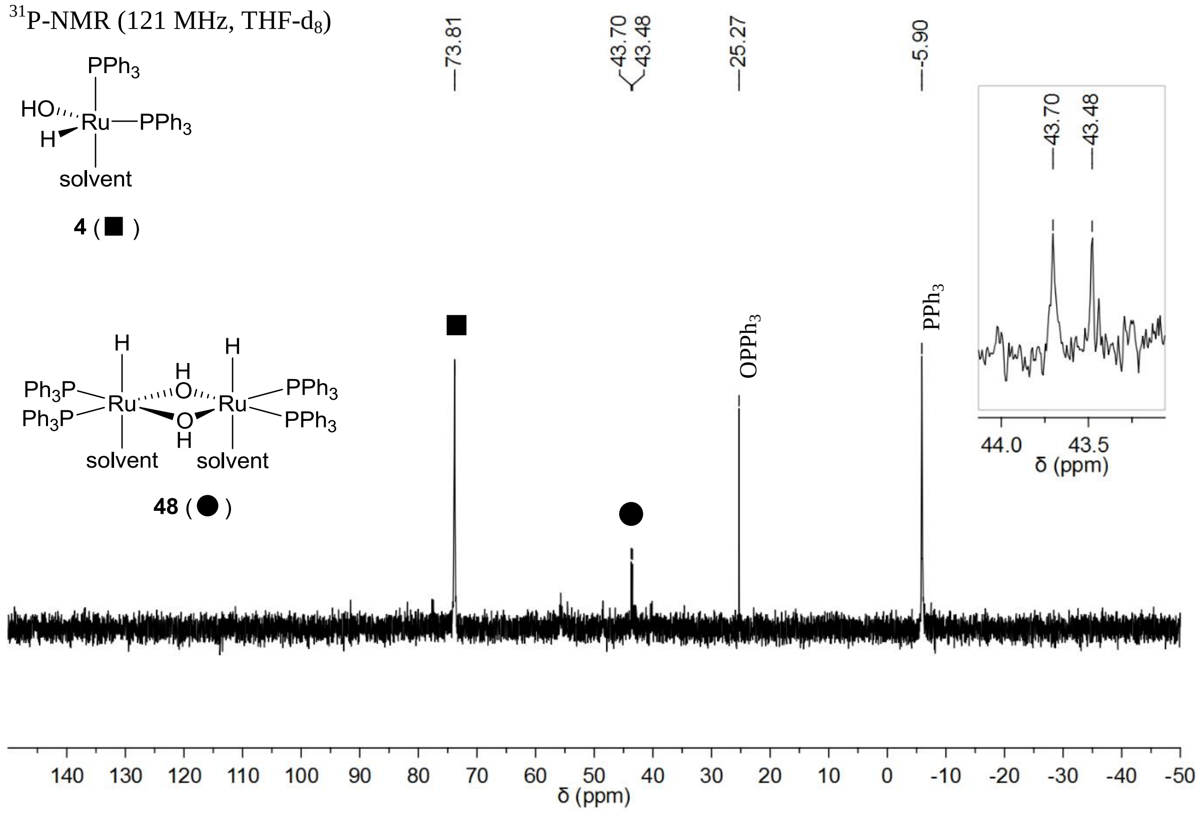

4.2 $RuH_2(H_2)(PPh_3)_3$ 3[12]

^{1}H-NMR (300 MHz, THF-d$_8$)

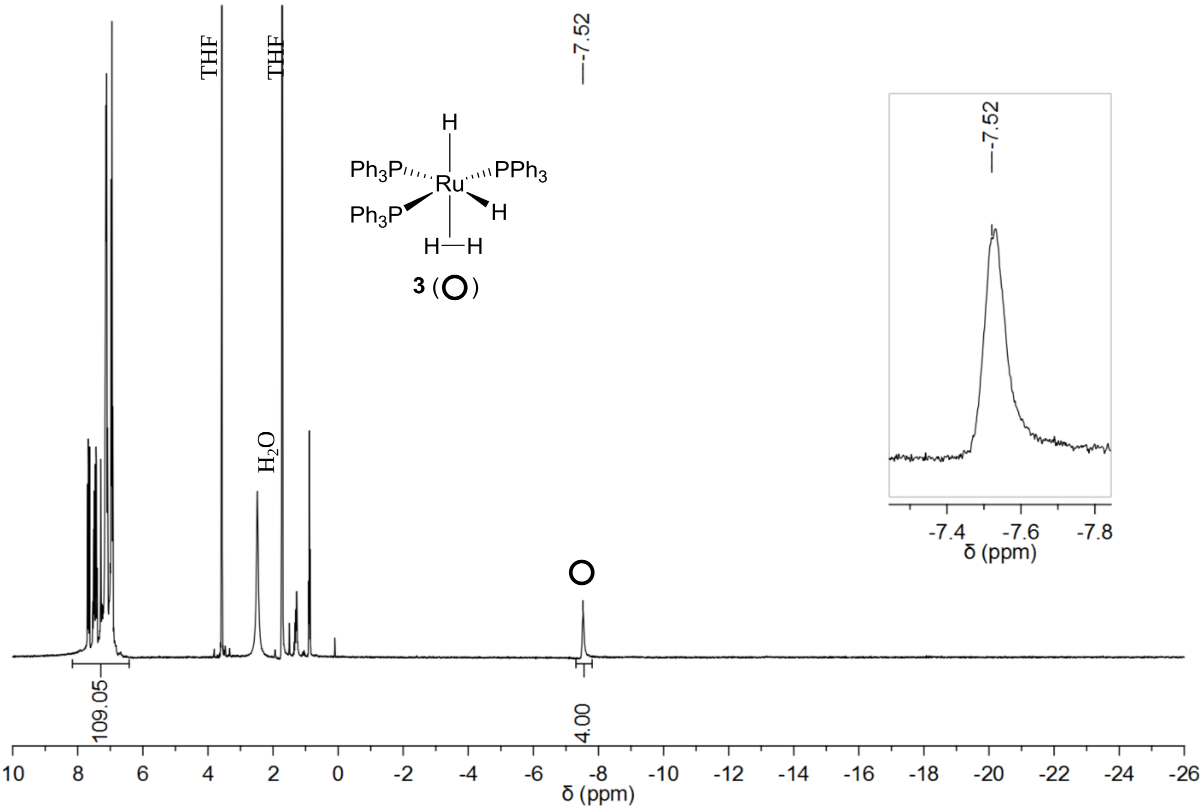

^{31}P-NMR (121 MHz, THF-d$_8$)

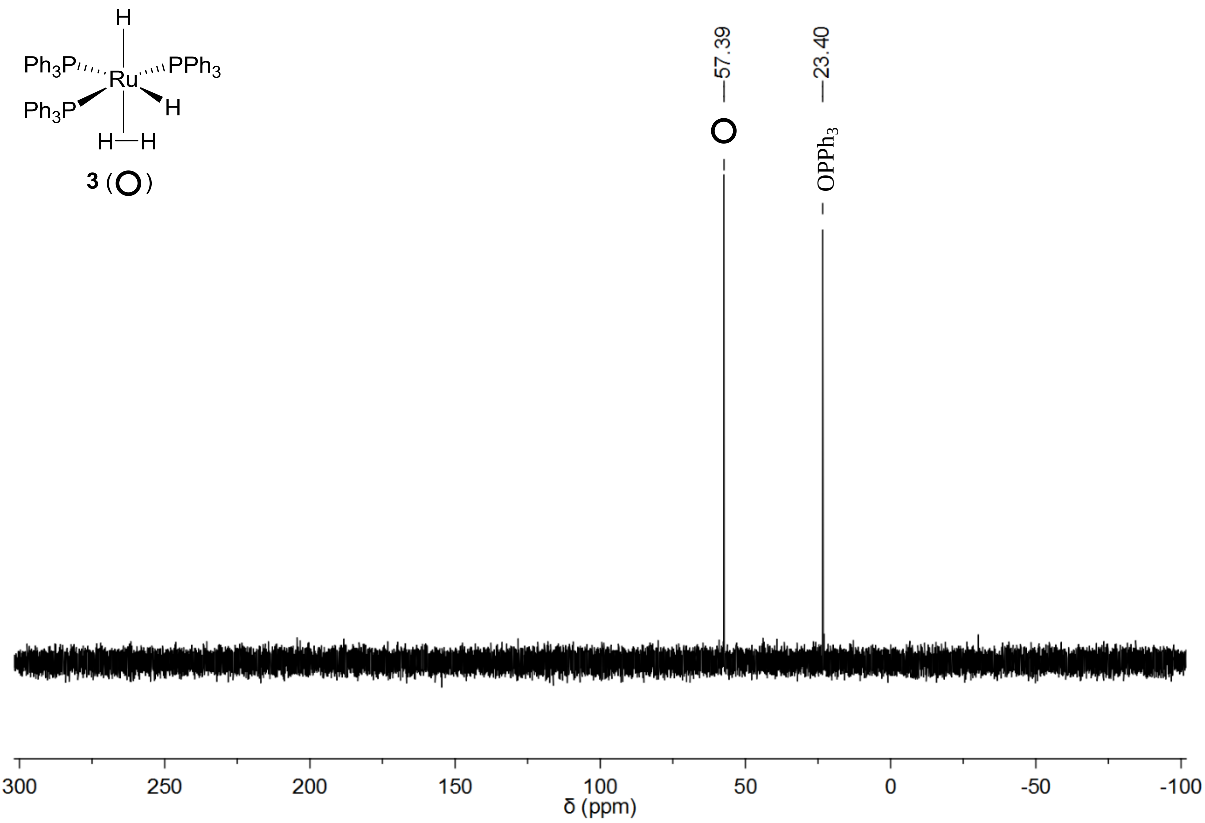

4.3 $RuHI(PPh_3)_3$ 2

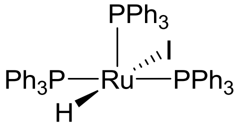

All solvents were degased with argon and the whole reaction was carried out under argon atmosphere. $RuCl_2(PPh_3)_3$ (1.92 g, 2 mmol, 1 eq.), Zn (10.47 g, 160 mmol, 320 eq.) and CuI (1.52 g, 8 mmol, 4 eq.) were dissolved in 1,4-dioxane (160 ml). Deionized H_2O (12.8 ml, 640 mmol, 320 eq.) was added and the crude mixture was stirred for 2 h at 80 °C. The solid was isolated using a filter frit and washed with 1,4-dioxane (50 ml) and deionized H_2O (30 ml). The residue was dried in high vacuum for 2 h, washed with *n*-pentane (35 ml) and dissolved with DCM (110 ml). The solvent was evaporated using an ether bridge and the resultion purple solid dried in high vacuum for 1 h.

Yield: 1.77 g (1.7 mmol, 87%).

Physical State: purple solid.

^{1}H NMR (Avance 400 MHz, CD_2Cl_2) δ 7.16 – 7.08 (m, 27H), 6.90 (t, J = 7.6 Hz, 18H), -15.38 (q, J = 25.1 Hz, 1H) ppm.

^{31}P NMR (Avance 162 MHz, CD_2Cl_2) δ 56.8 (broad) ppm.

The analytical data were in accordance with the literature.[13]

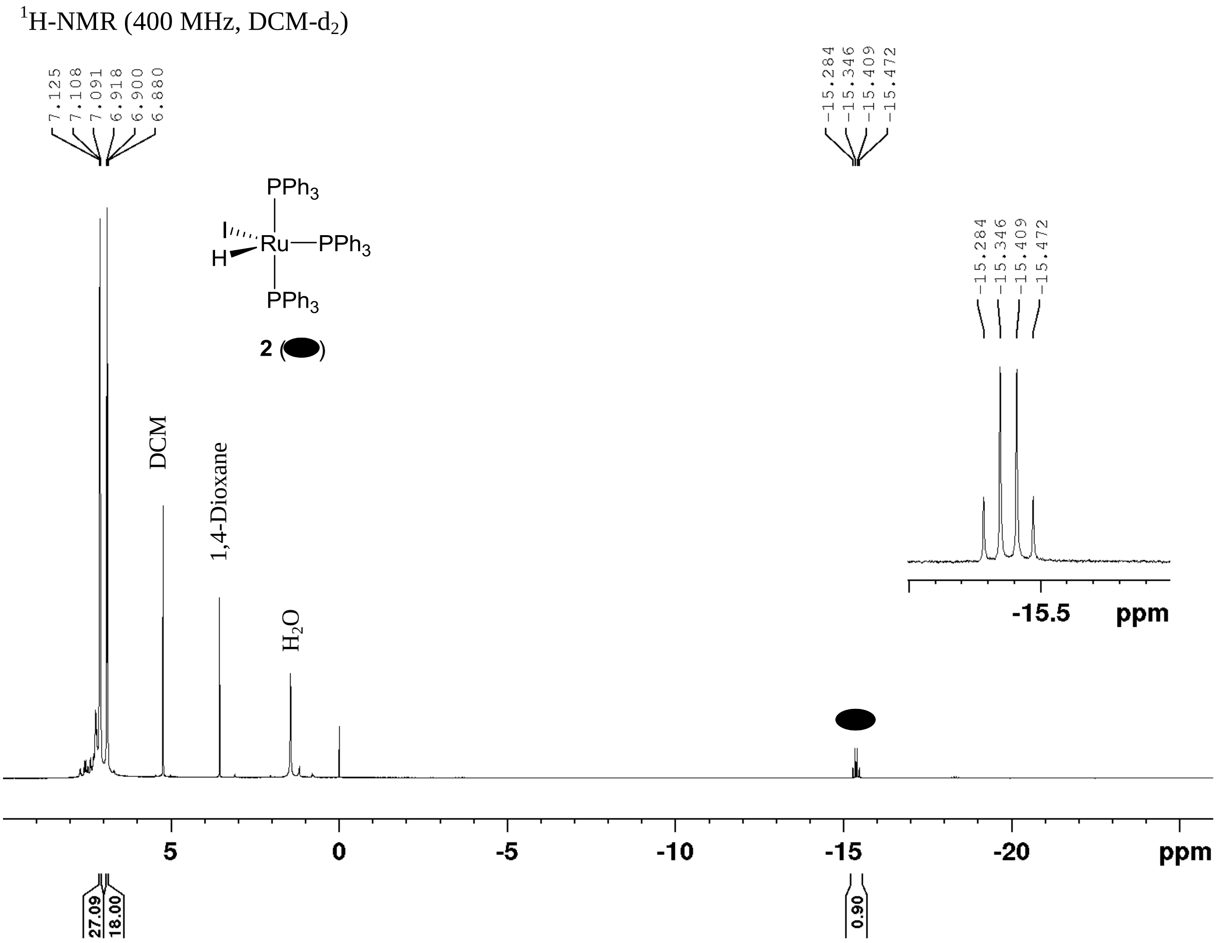
^{1}H-NMR (400 MHz, DCM-d$_2$)
7.125
7.108
7.091
6.918
6.900
6.880
-15.284
-15.346
-15.409
-15.472
PPh3
I
Ru
PPh3
H
PPh3
2
DCM
1,4-Dioxane
H2O
-15.5
ppm
5
0
-5
-10
-15
-20
ppm
27.09
18.00
0.90

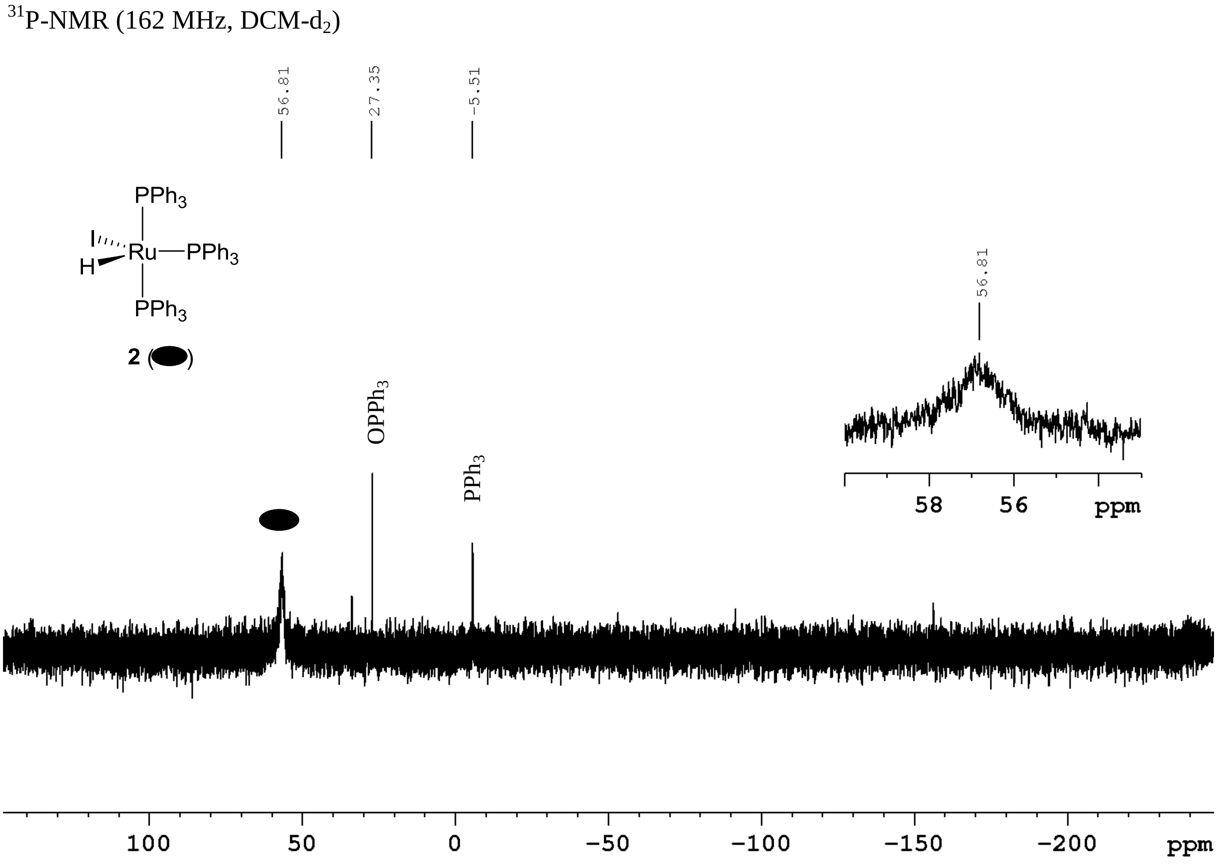
^{31}P-NMR (162 MHz, DCM-d$_2$)
56.81
27.35
-5.51
PPh3
I
Ru
PPh3
H
PPh3
2
OPPh3
PPh3
56.81
58
56
ppm
100
50
0
−50
−100
−150
−200
ppm

5. Mechanistic studies

In order to investigate the additive-dependent chemoselectivity we focused on isolating the hypothetically catalytic active species and subsequent evaluation of their activity in deuteration reactions.

The following scheme shows the additive-dependent pathways that lead to each species.

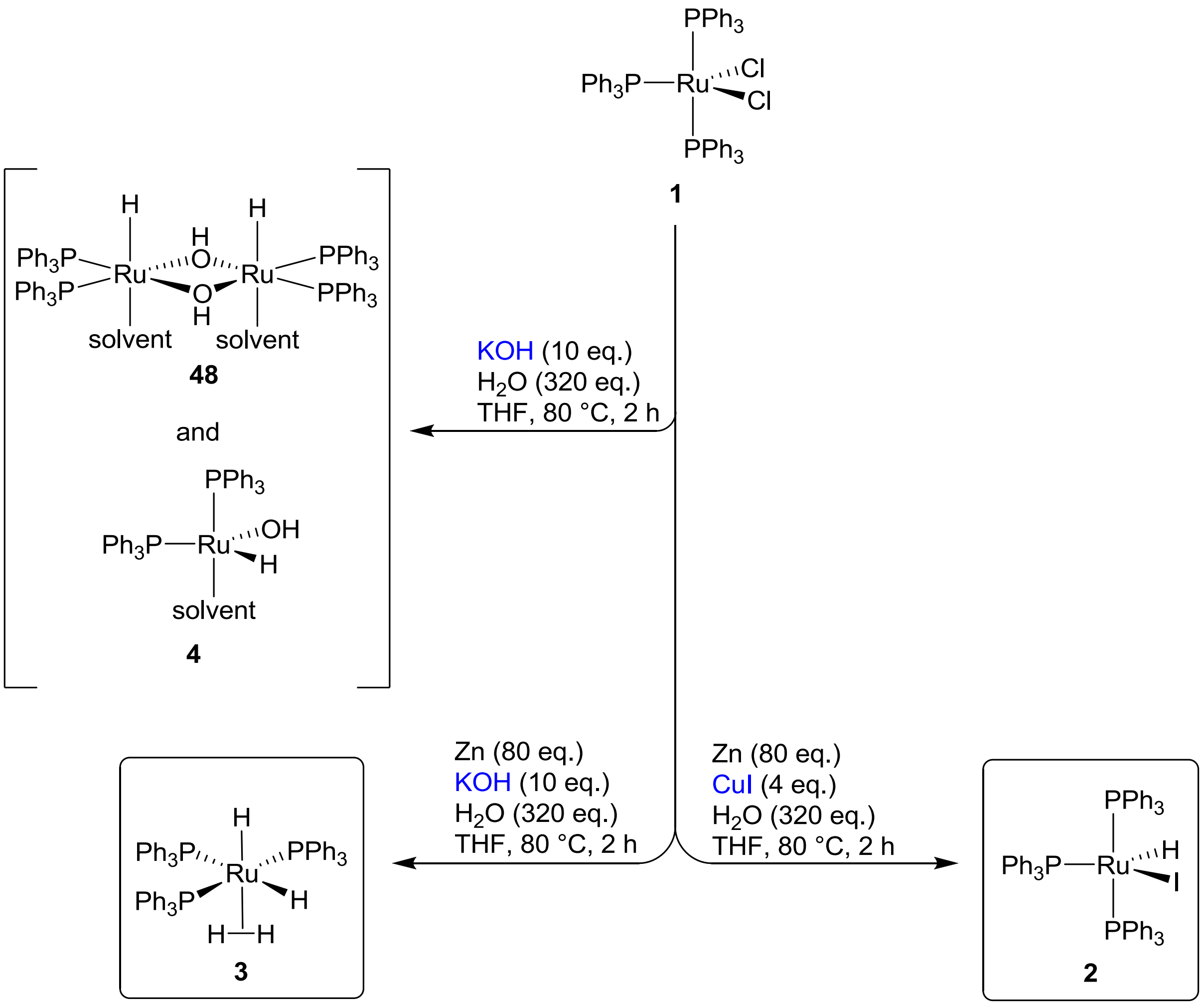

Scheme 1: Overview of hypothetical catalytic species.

5.1 KOH-protocol

We started our studies by subjecting complex **1** to exemplary basic conditions and observed the formation of the dimeric (μ-OH) complex **48** (Figure 1).

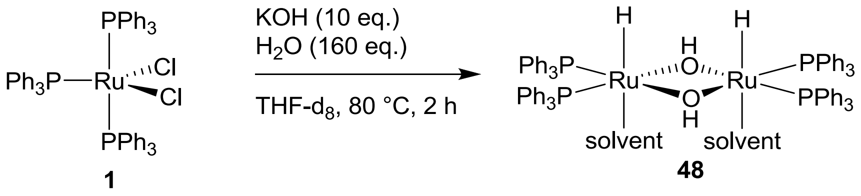

Figure 1: Formation of dimeric ruthenium hydrido-hydroxy complex **48**.

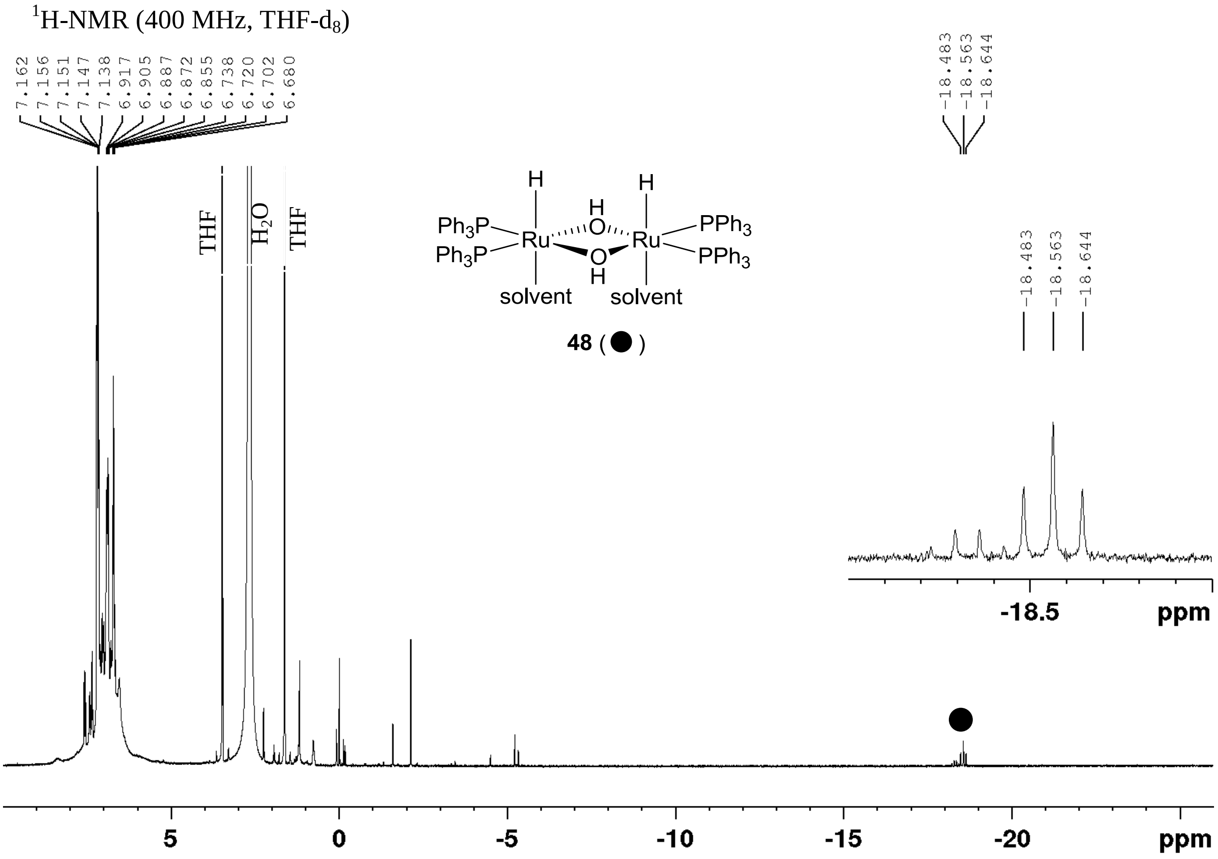

^{31}P-NMR (162 MHz, THF-d_8)

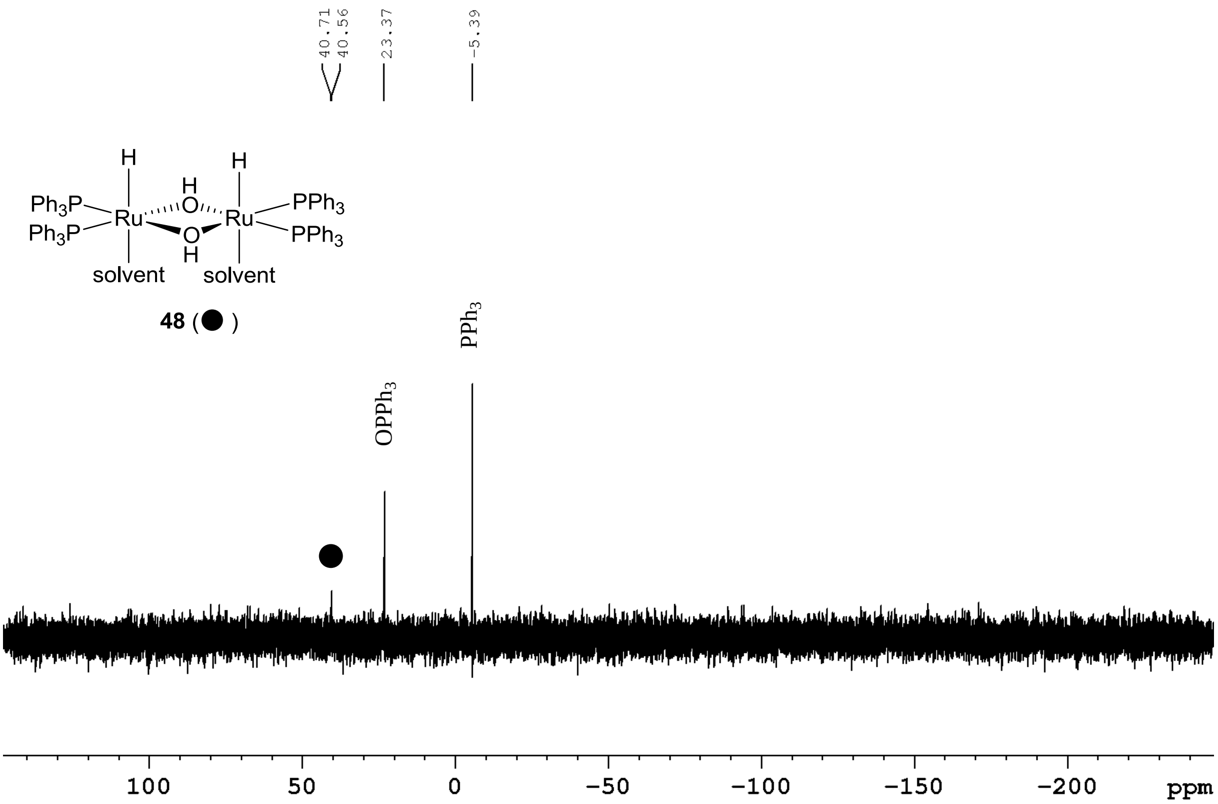

When the equivalents of water were raised to a level similar to the reaction conditions (Figure 2) we observed an increase in the monomeric ruthenium hydroxy species **4**. *Wilkinson* reported the formation of both complexes depending on reaction time and equivalents of water and base.[11] In our case we observed that the ratio of monomeric to dimeric species is highly dependent on the amount of water used.

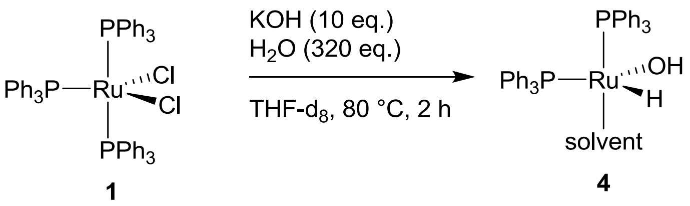

Figure 2: Formation of monomeric ruthenium hydrido-hydroxy species **4**.

^{1}H-NMR (400 MHz, THF-d_8)

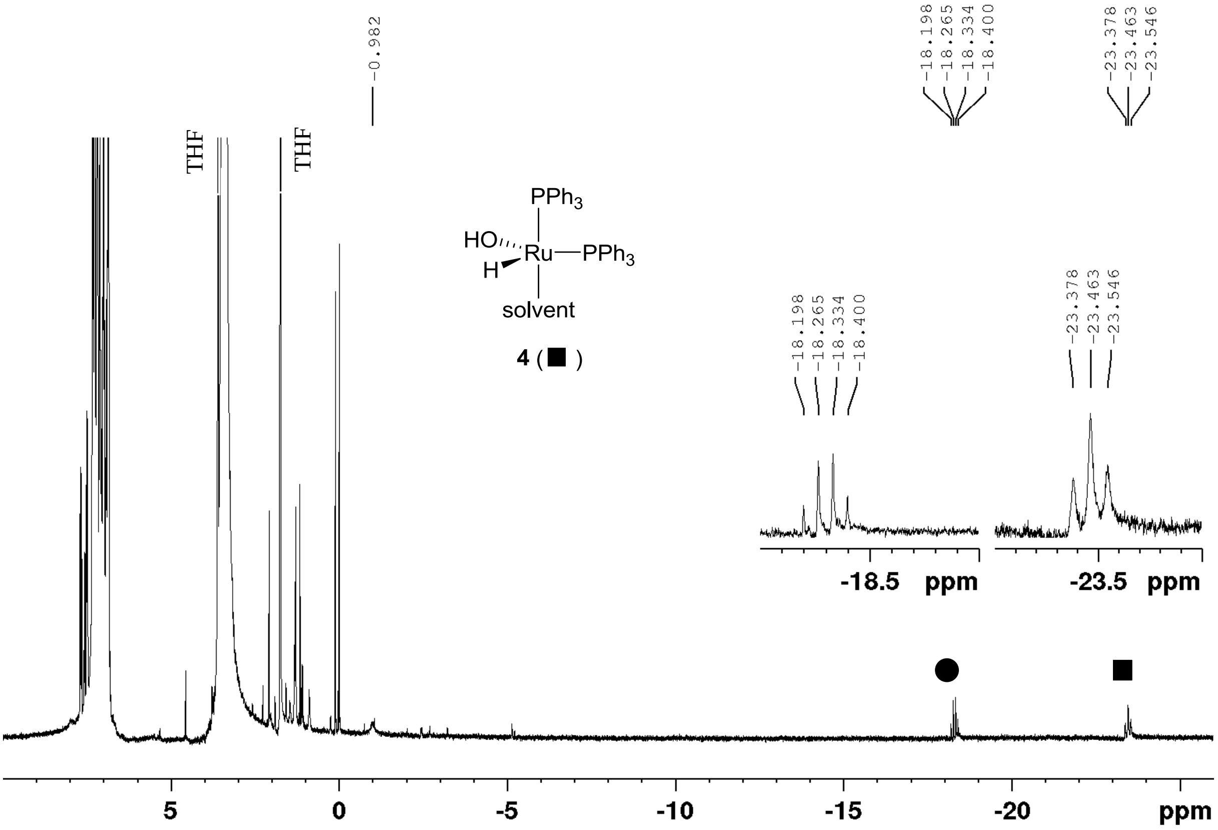

^{31}P-NMR (162 MHz, THF-d_8)

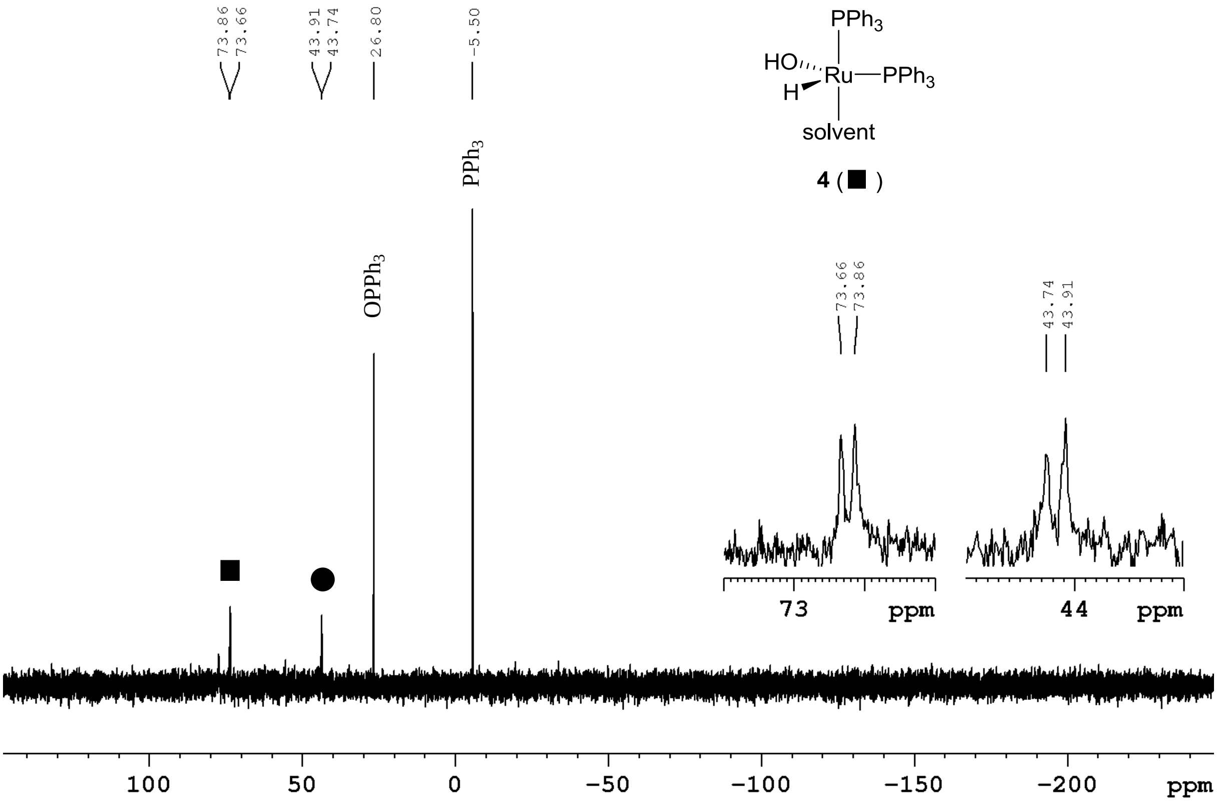

We were not able to test the pure complexes for their respective catalytic activity due to both complexes being instable upon isolation. The aforementioned experiments give us strong

indication that a mixture of both the monomeric complex **4** as well as the dimeric complex **4** are present in our catalysis. Ultimately we assume the monomeric species to be the major component and thus the catalytic active species.

5.2 KOH/Zn-protocol

Under reductive basic conditions the formation of the tetrahydrido ruthenium complex **3** and its closely related dimeric complex **49** is observed.

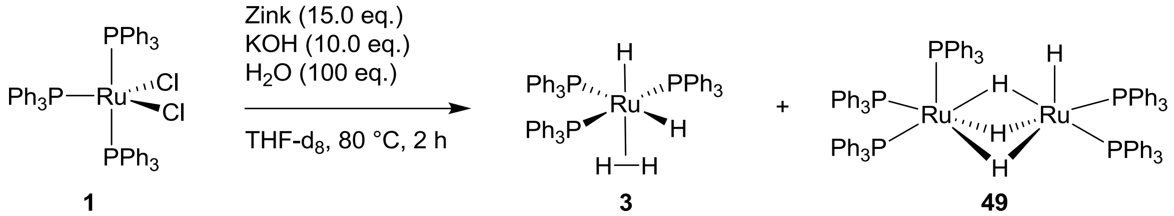

Figure 3: Formation of ruthenium hydride complexes under reductive conditions.

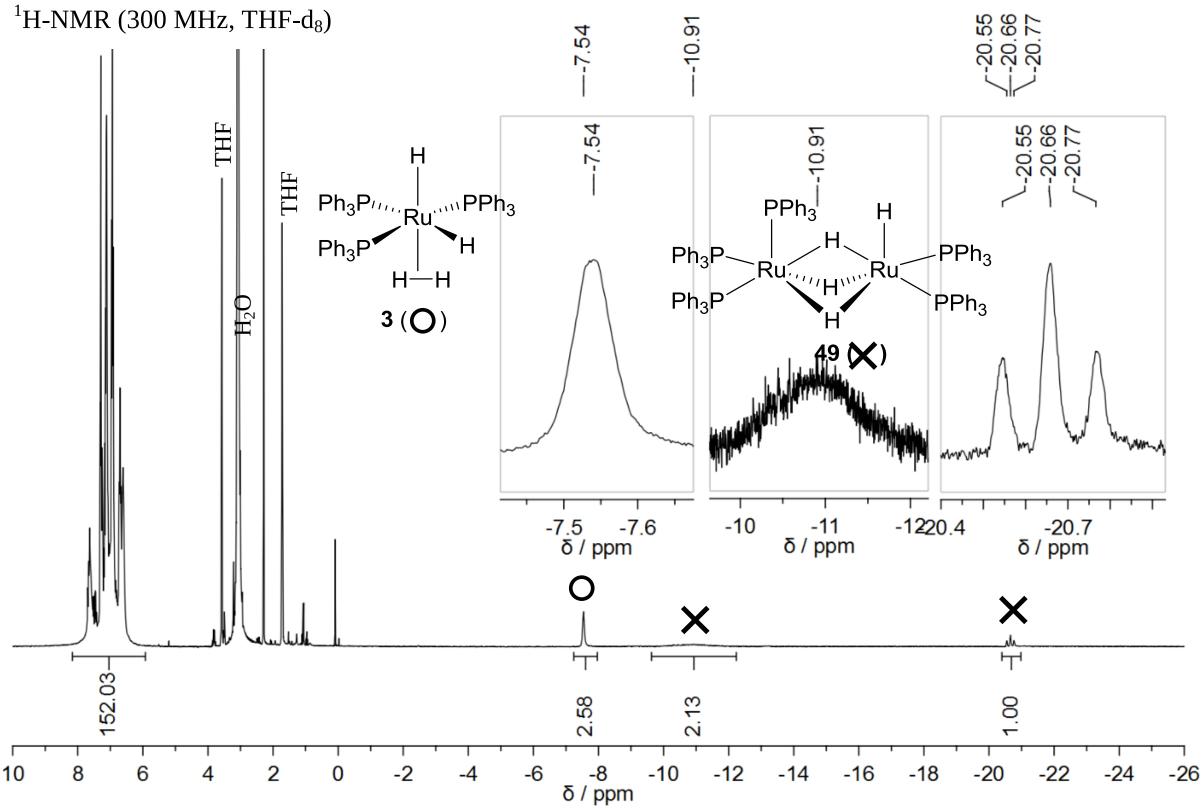

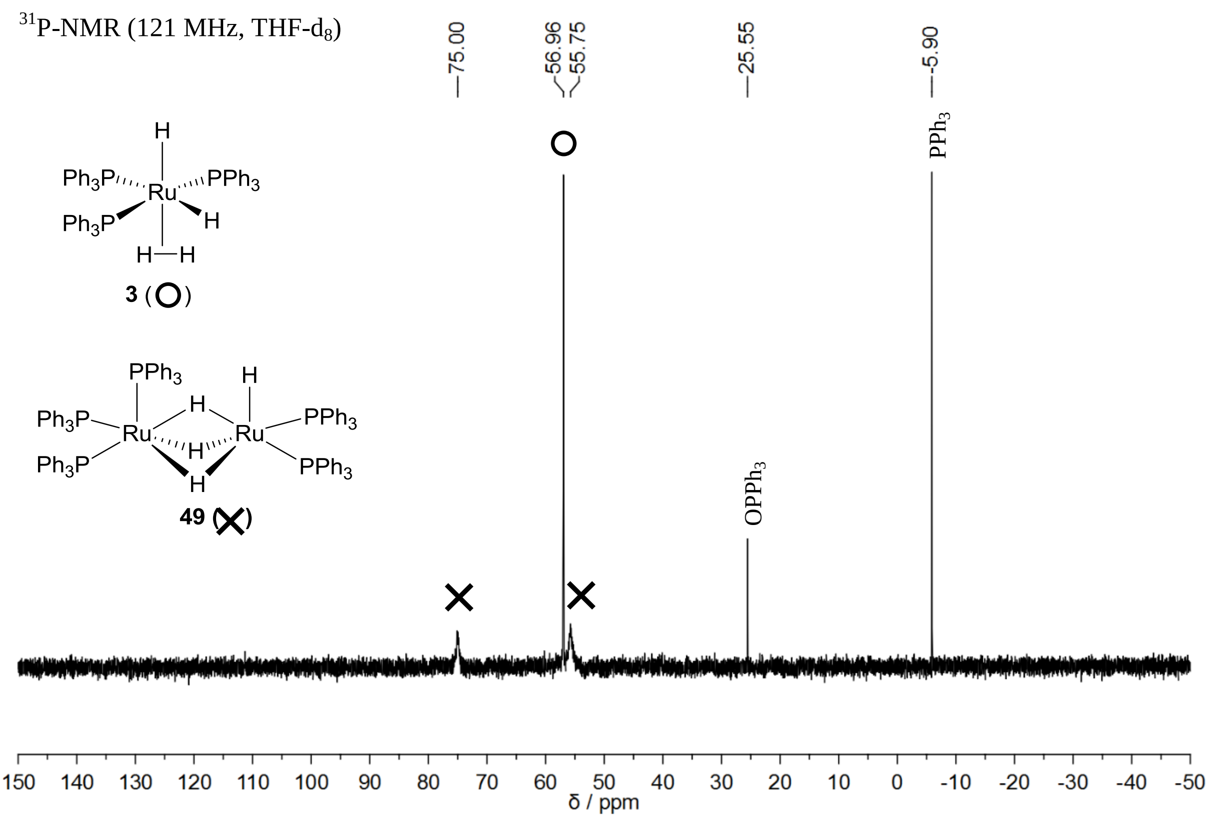

We assigned the signals of the hydride complexes by previously reported literature.[12,14]
By recording the ^{1}H NMR at low temperature (-50 °C) we obtained a better resolution for the (µ-H_3) signals of the dimeric complex **49** (Figure 4).

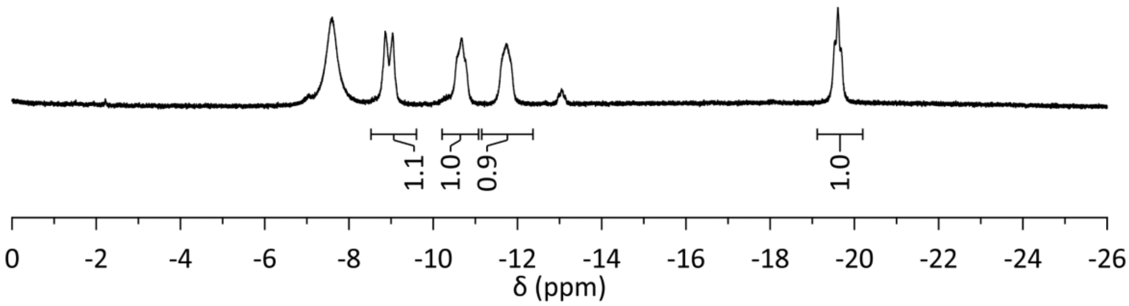

Figure 4: Low-temperature ^{1}H NMR of dimeric hydride complex **49**.

We increased the equivalents of the reagents to match the conditions in our catalytic protocol (Figure 5) but saw no significant change in complex ratio.

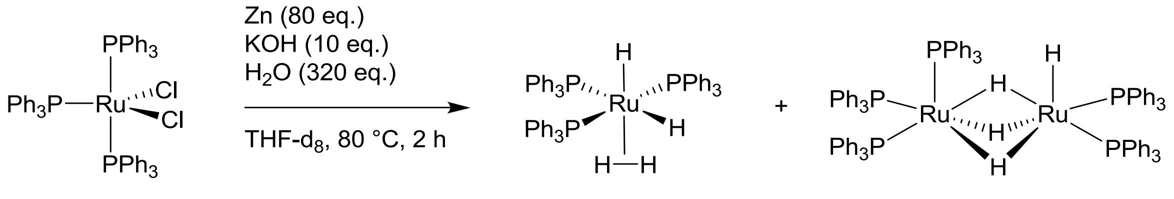

1 **3** **49**

Figure 5: Formation of ruthenium hydride complexes **3** and **49** under catalysis reaction conditions.

We assume that the tetrahydrido ruthenium complex **3** is the active species in our catalysis as there are many reported applications in which complex **3** acts as a powerful reduction catalyst.[15]

5.2.1 Deuteration with $RuH_2(H_2)(PPh_3)_3$

Furthermore, we synthesized the complex **3** following a slightly modified procedure from *Grushin*[12] and employed the complex in an exemplary catalysis (Figure 6).

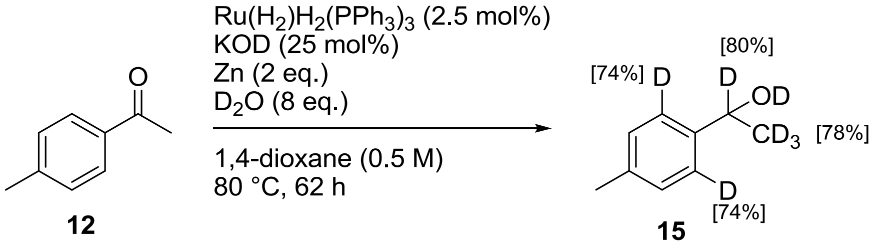

12 **15**

Figure 6: Deuteration of 4-methylacetophenone with $RuH_2(H_2)(PPh_3)_3$ **3** as catalyst.

To our delight, we saw the same degree of deuteration compared to our result when using $RuCl_2(PPh_3)_3$ as precatalyst. This is a strong indication that under these conditions the reactive species of our deuteration is indeed the tetrahydrido species **3**.

^{1}H-NMR (400 MHz, CD_2Cl_2) spectra of deuterated compound **15** following the KOD/Zn procedure for 62 h with $RuH_2(H_2)(PPh_3)_3$ **3** (2.5 mol%): Yield: 72%

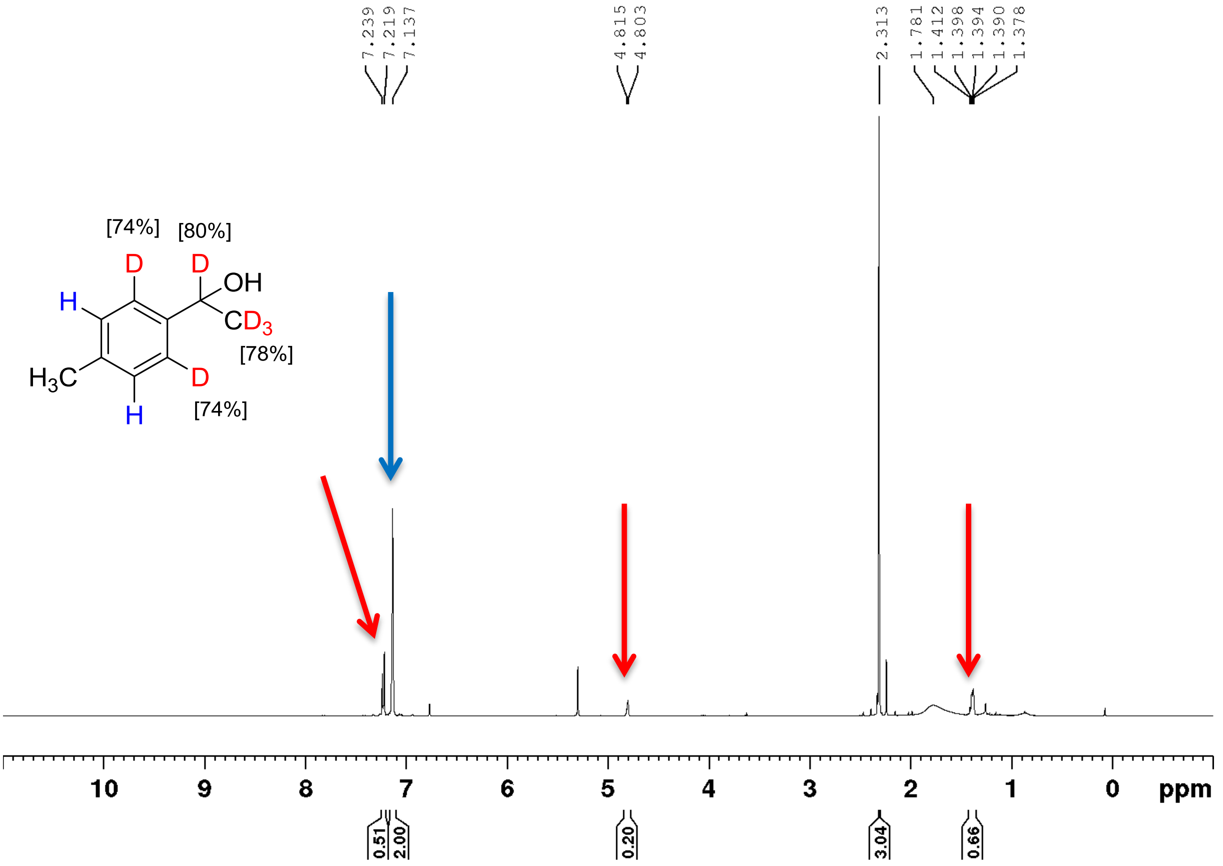

5.3 CuI-protocol

For the CuI protocol, we were able to observe the iodide complex **2** that was smoothly formed under the reaction conditions (Figure 7).

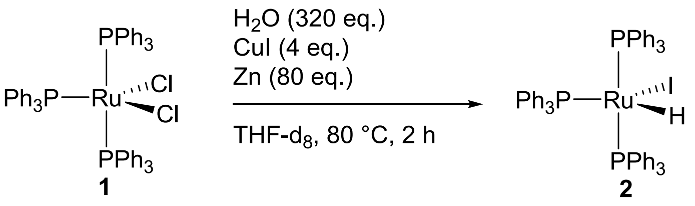

Figure 7: Formation of the iodide complex **2** under conditions A.

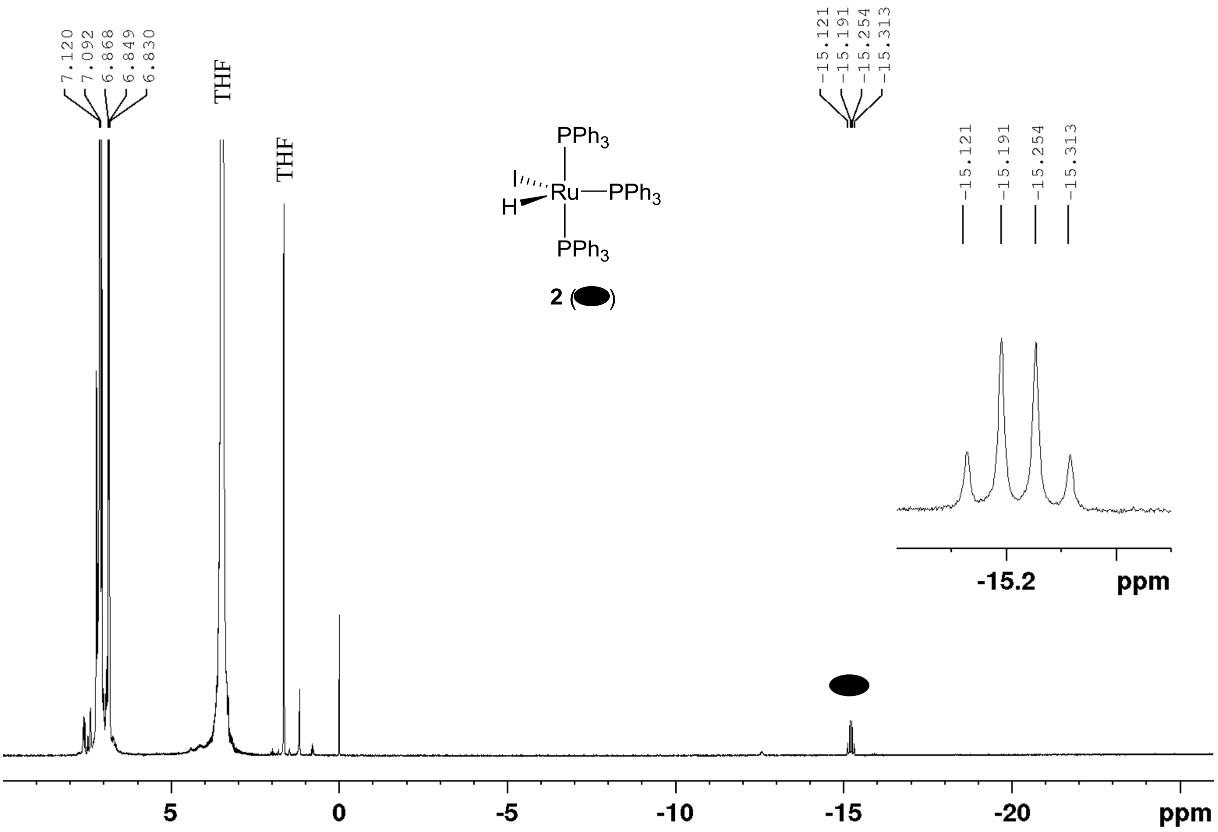
7.120
7.092
6.868
6.849
6.830
THF
THF
-15.121
-15.191
-15.254
-15.313
PPh3
I
H
Ru
PPh3
PPh3
2 ( )
-15.121
-15.191
-15.254
-15.313
-15.2
ppm
5
0
-5
-10
-15
-20
ppm

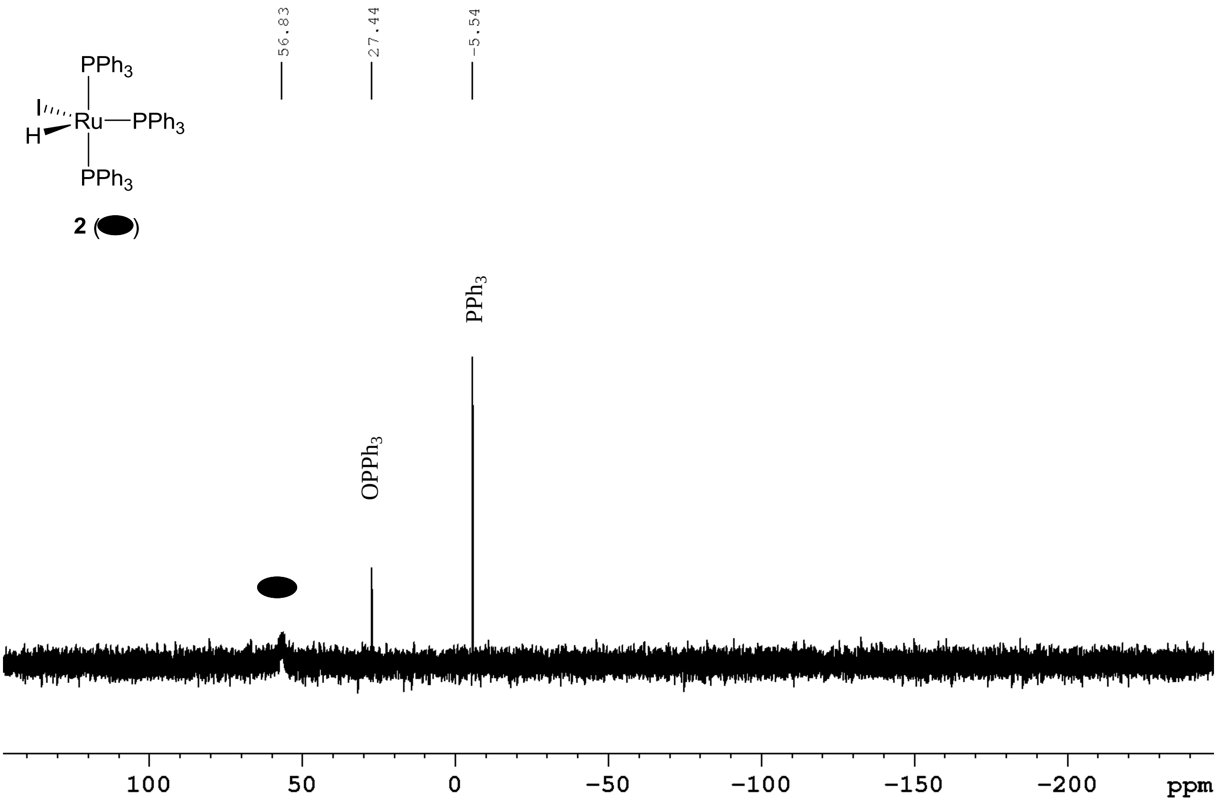
PPh3
I
H
Ru
PPh3
PPh3
2 ( )
56.83
27.44
-5.54
PPh3
OPPh3
100
50
0
-50
-100
-150
-200
ppm

5.3.1 Deuteration with RuHI(PPh$_3$)$_3$ 2

When the iodide catalyst **2** was used in an exemplary deuteration reaction, we were able to surpass the degree of deuteration that we observed when we used the precatalyst **1** (Figure 8). Additionally, we observed the same selectivity and thus conclude that the iodide ruthenium complex **2** is the catalytic active species under conditions A.

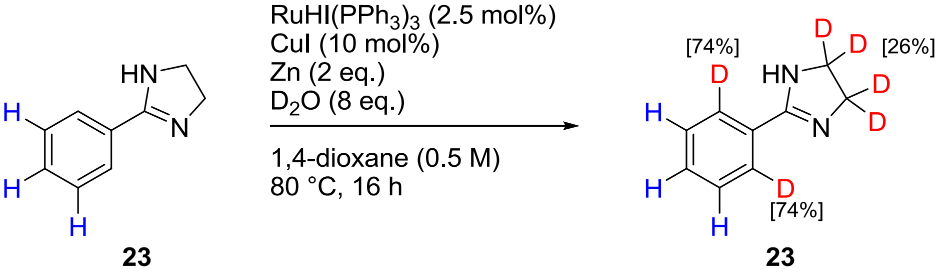

Figure 8: Deuteration of 2-phenyl-2-imidazole **23** with RuHI(PPh$_3$)$_3$ **2** under conditions A.

^{1}H-NMR (400 MHz, CD_2Cl_2) spectra of deuterated compound **23** following the CuI procedure for 16 h with RuHI(PPh$_3$)$_3$ **2** (2.5 mol%): Yield: >99%

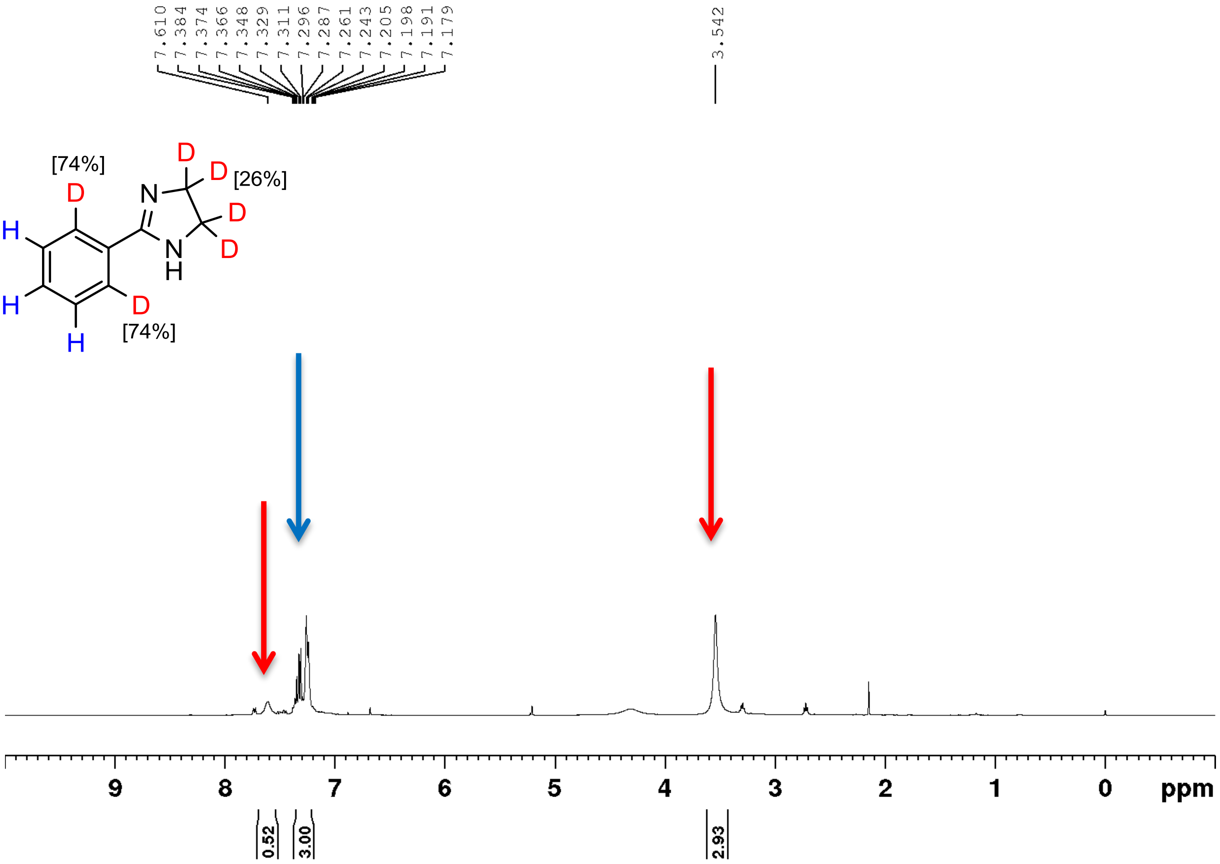

5.3.2 Reduction with RuHI(PPh$_3$)$_3$ 2

Regarding the chemoselectivity we studied the behaviour of complex **2** in the reduction of 4-(phenylethinyl)acetophenone **38**. To our delight we were able to demonstrate the high

selectivity of **2** towards the reduction of alkynes while no reduction of the carbonyl was observed. This further strengthens our hypothesis that **2** is the catalytic active species.

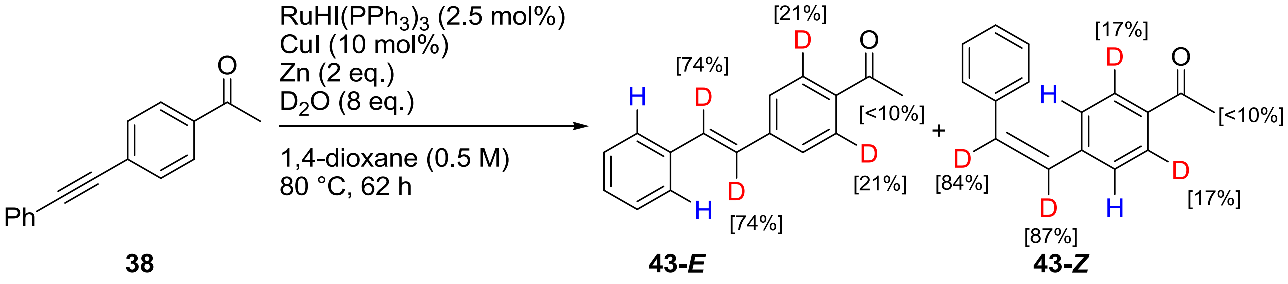

Figure 9: Reduction of 4-(phenylethinyl)acetophenone **38** with RuHI(PPh_3)$_3$ **2** under conditions A.

^{1}H-NMR (400 MHz, $(CD)_2(Cl_2)_2$) Spectra of deuterated compound **43-*Z*** following the CuI procedure for 62 h with RuHI(PPh_3)$_3$ **2** (2.5 mol%): Yield: 20%

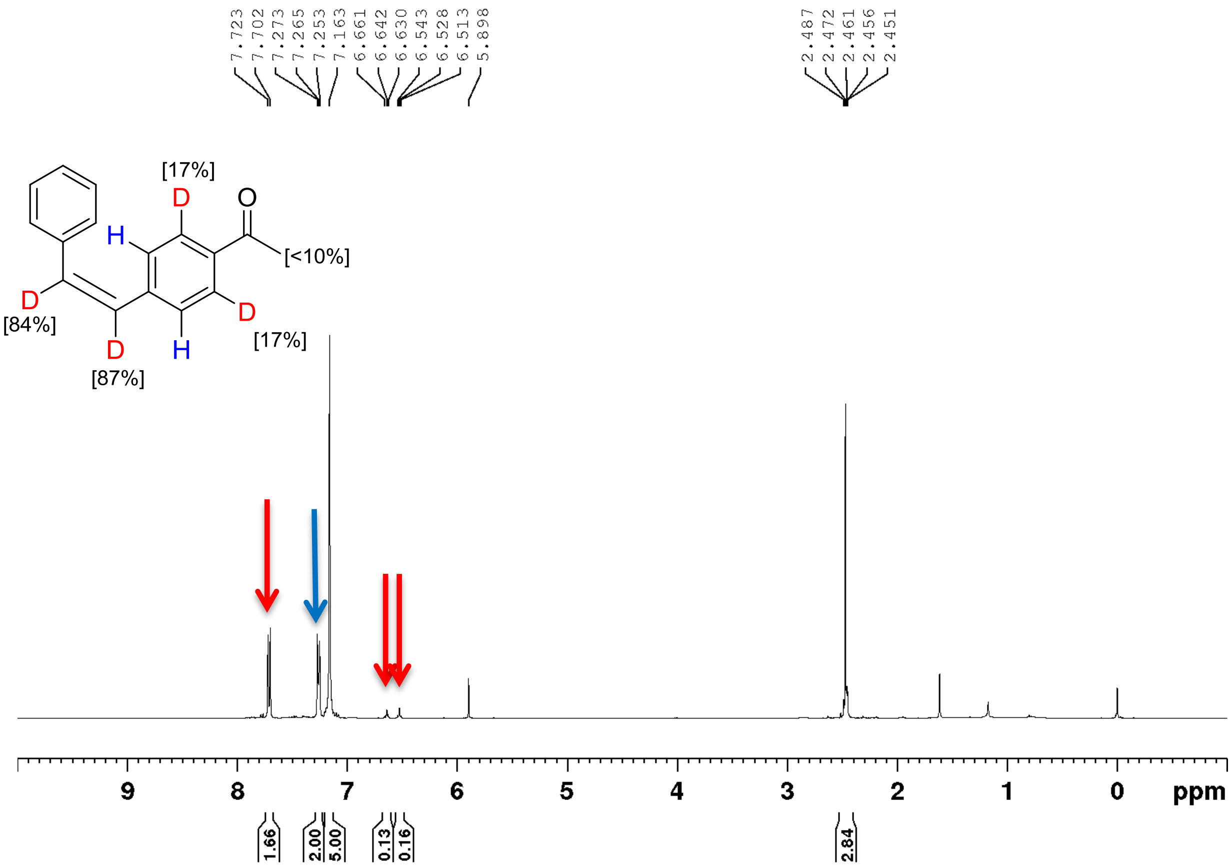

Enlargement of relevant area:

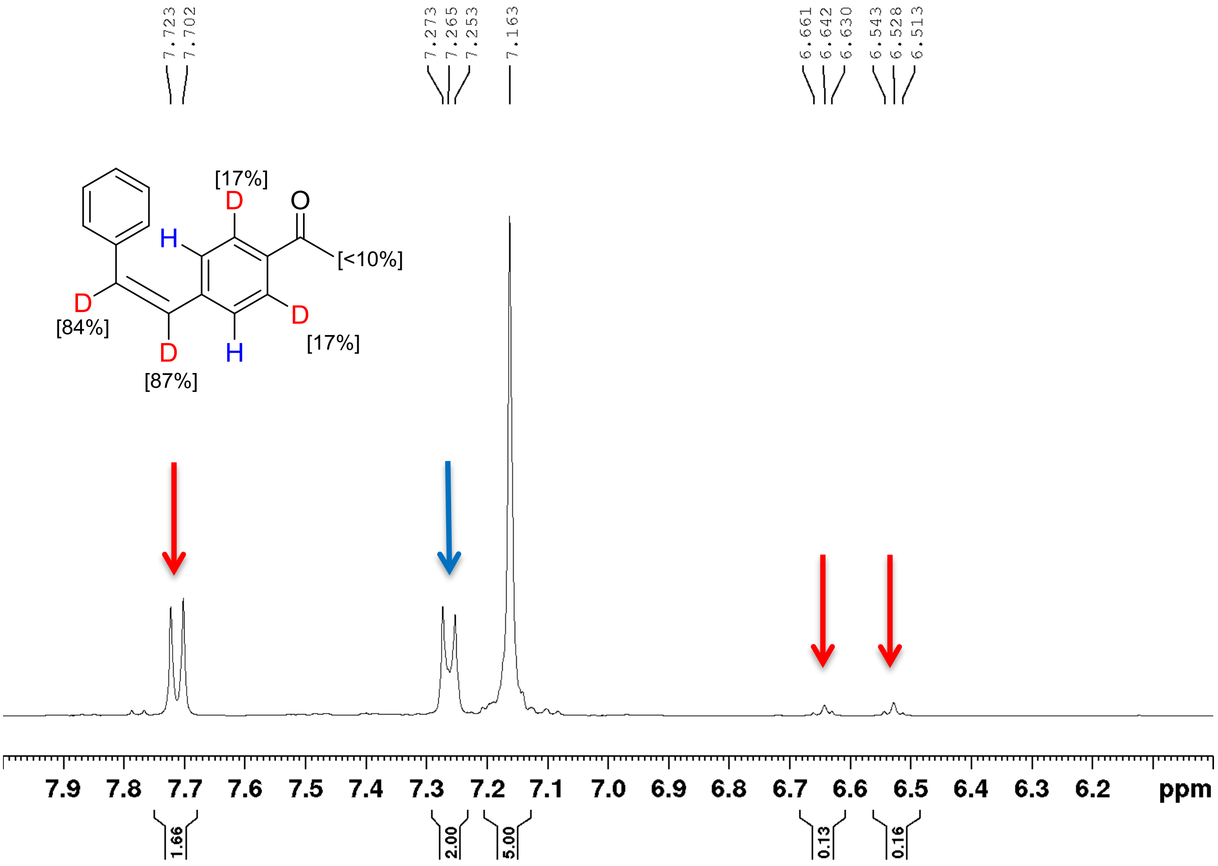

^{1}H-NMR (400 MHz, CD_2Cl_2) Spectra of deuterated compound **43-*E***: Yield: 23%

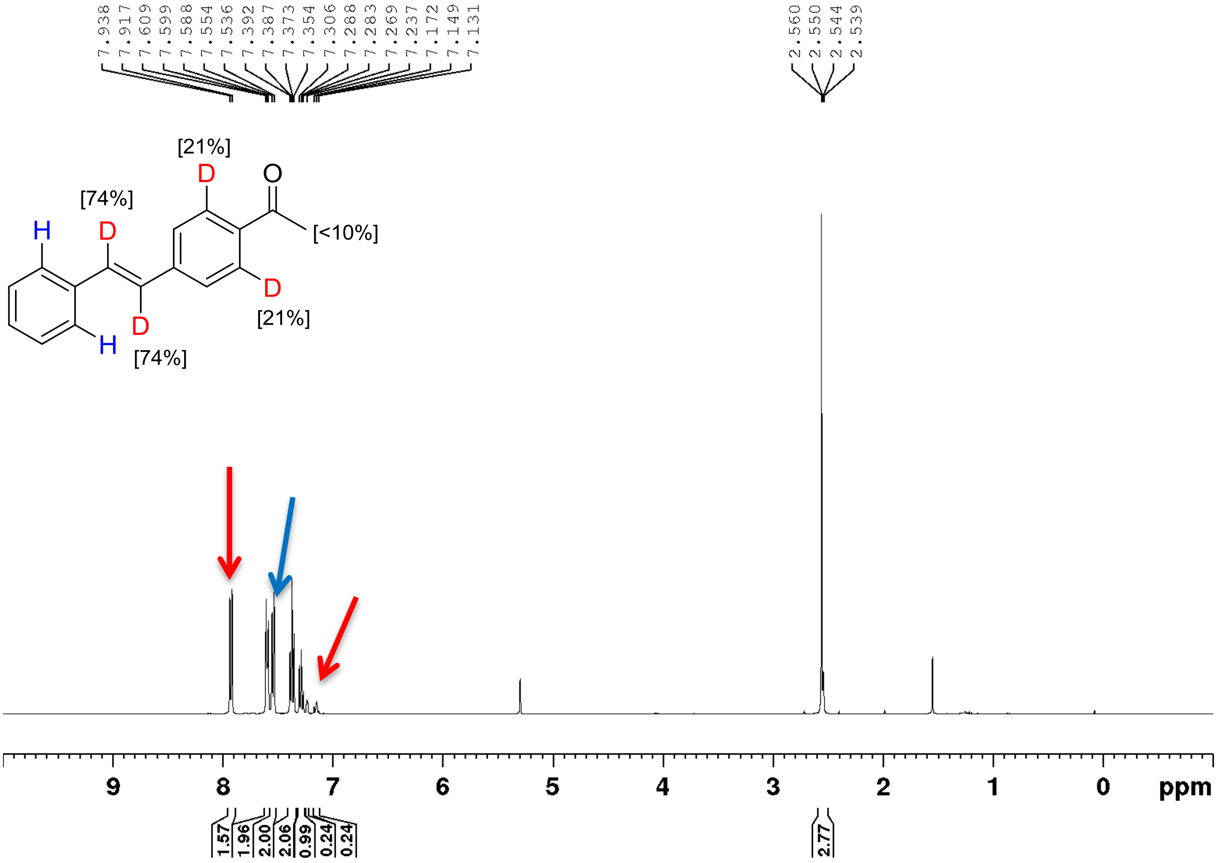

Enlargement of relevant area:

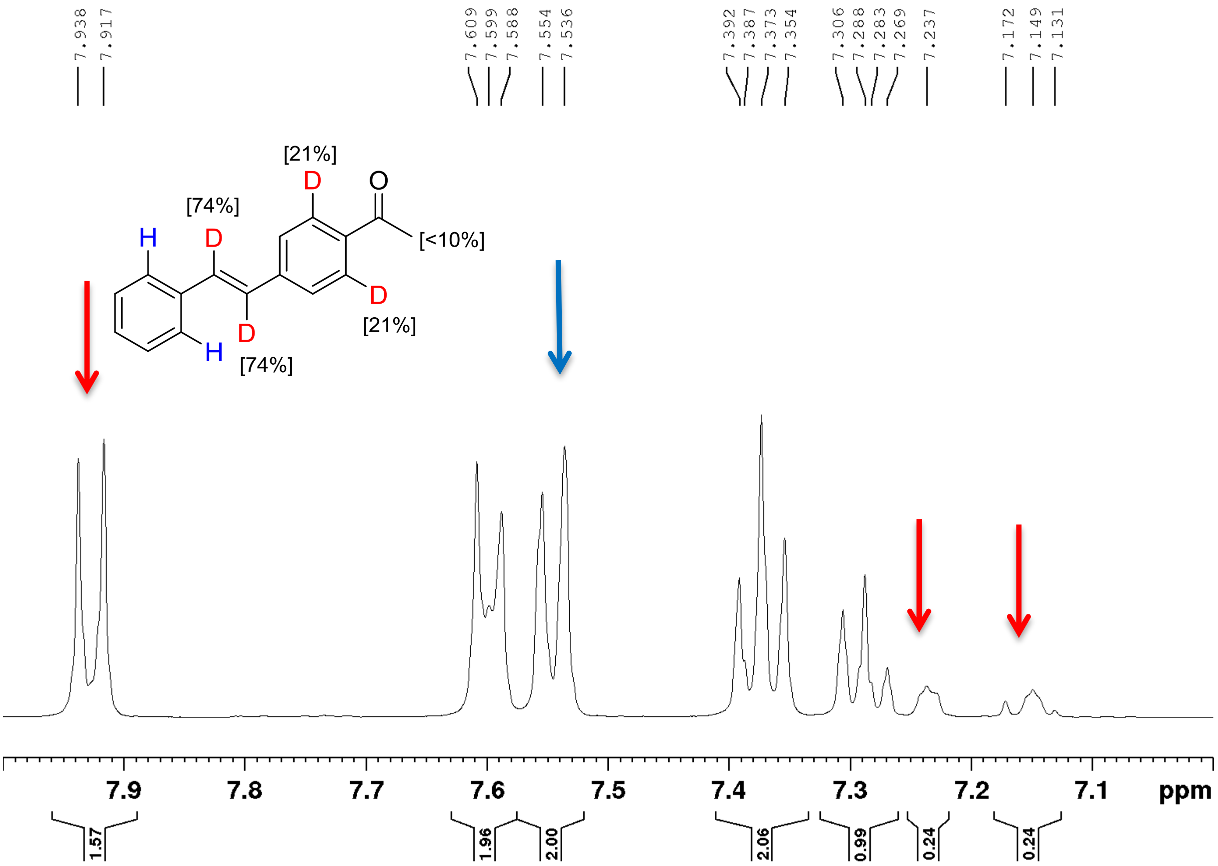

5.4 Verification of D_2 gas formation

To verify the formation of D_2 gas from the deuterium source D_2O when using zinc as additive, we designed a control experiment where the formation of D_2 can take place separated from the deuterium labeling while still sharing the same gas atmosphere. That way, we hope to prove the formation of D_2 gas.

The reactions took place in a two chambered Schlenktube with connected gas atmosphere (Figure 9).

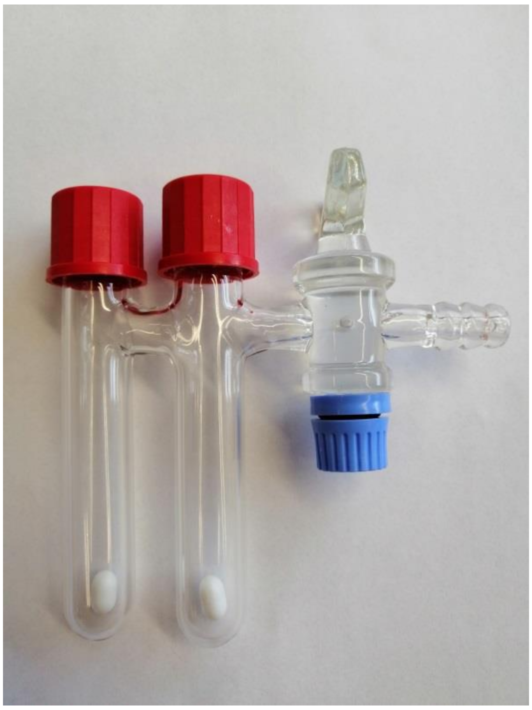

Figure 9: Two-chambered Schlenktube.

For convenience we named one chamber "D_2 chamber" and the other one "deuteration chamber". We performed the control experiments with the following chamber loadings:

**Entry**	**D_2 chamber**				**Deuteration chamber**			
**1**	CuI [mol%]	Zn [eq.]	Ru [mol%]	D_2O [eq.]	CuI [mol%]	Zn [eq.]	Ru [mol%]	Substrate [mmol]
	10	2	2.5	8	10	2	2.5	0.5
**2**	KOD [mol%]	Zn [eq.]	Ru [mol%]	D_2O [eq.]	KOH [mol%]	Zn [eq.]	Ru [mol%]	Substrate [mmol]
	25	2	2.5	8	25	2	2.5	0.5

reaction was run with 1 ml 1,4-dioxane as solvent in each chamber for the given reaction time at 80 °C.

The result of these experiments is summarized in the following scheme (scheme 2):

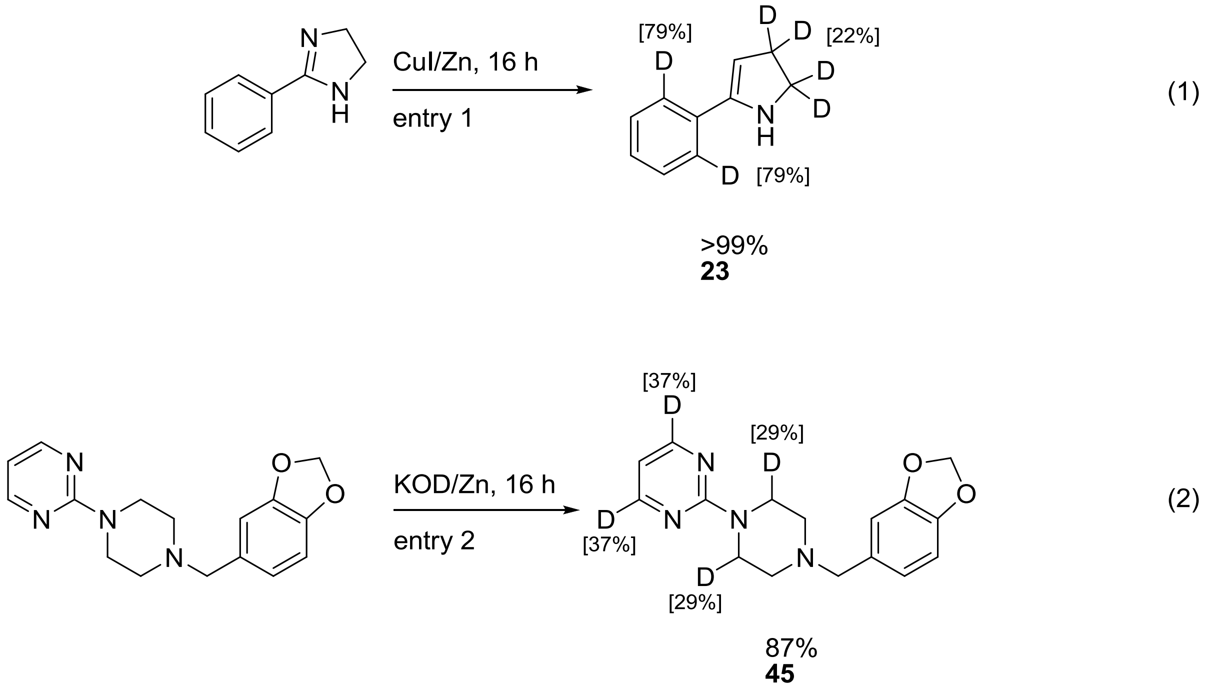

Scheme 2: Overview of deuteration with *in situ* generated D_2 gas.

For the CuI/Zn conditions (eq. (1), scheme 2) we observed approximately the same yield and deuteration degrees as when the one-pot procedure was used. For the KOD/Zn protocol (eq. (2), scheme 2) a slight adjustment had to be made. Since KOD is obtained as a solution in D_2O we used KOH for the "deuteration chamber". Therefore we expected a lower degree of

deuteration since the KIE might lead to a preferential implementation of hydrogen instead of deuterium. Nevertheless we observed deuteration with the same chemoselectivity as when the one-pot procedure was applied. With regard to the experimental setup we interpreted these results as a proof for the formation of D_2 gas when the additive zinc was used.

6. Literature

[1] T. Schabel, C. Belger, B. Plietker, *Org. Lett.* **2013**, *15*, 11, 2858-2861.

[2] D. Nishikawa, K. Hirano, M. Miura, *J. Am. Chem. Soc.* **2015**, *137*, 50, 15620-15623.

[3] R. M. Edkins, A. Wriglesworth, K. Fucke, S. L. Bettington and A. Beeby, *Dalton Trans.* **2011**, *40,* 9672-9678.

[4] A. I. Meyers, R. Gabel, E. D. Mihelich, *J. Org. Chem.* **1978**, *43,* 7, 1372-1379.

[5] A. Srikrishna, R. Viswajanani, *Tetrahedron*, **1995**, *51 (11)*, 3339-3344.

[6] R. Loeppky, W. Cui, *Tetrahedron Letters,* **1998**, *39 (14)*, 1845-1848.

[7] M. Debdab, F. Mongin, J.-P. Bazureau, *Synthesis*, **2006**, *23*, 4046-4052.

[8] A. Rahimi, I. Pápai, Á. Madarász, M. Gjikaj, J. C. Namyslo, A. Schmidt, *Eur. J. Org. Chem.* **2012**, 754-763.

[9] H. Bao, B. Zhou, H. Jin, Y. Liu, *J. Org. Chem.* **2019**, *84*, 3579-3589.

[10] M. Mollo, L. Orelli, *Org. Lett.* **2016,** *18*, 6116-6119.

[11] B. N. Chaudret, D. J. Cole-Hamilton, R. S. Nohr, G. Wilkinson, *J. Chem. Soc. Dalton Trans.* **1977**, 1546-1577.

[12] H. Samouei, F. M. Miloserdov, E. C. Escudero-Adán, V. V. Grushin, *Organometallics* **2014**, *33*, 7279-7283.

[13] F. M. Miloserdov, D. McKay, B. K. Muñoz, H. Samouei, S. A. Macgregor, V. V. Grushin, *Angew. Chem. Int. Ed.* **2015**, 8466-8470.

[14] L. S. van der Sluys, G. J. Kubas, K. G. Caulton, *Organometallics* **1991**, *10*, 1033–1038.

[15] a) L. Xu, G. Ou, Y. Yuan, *J. Organomet. Chem.* **2008**, *693*, 3000-3006; b) D. Linn Jr., J. Halpern, *J. Organomet. Chem.* **1987,** *330*, 155-159; c) Y. Lin, Y. Zhou, *J. Organomet. Chem.* **1990**, *381*, 135-138.
